# Metal-free deoxygenative coupling of alcohol-derived benzoates and pyridines for small molecules and DNA-encoded libraries synthesis†

**DOI:** 10.1039/d2sc01621d

**Published:** 2022-05-10

**Authors:** Sai Rohini Narayanan Kolusu, Manuel Nappi

**Affiliations:** Centro Singular de Investigación en Química Biolóxica e Materiais Moleculares (CiQUS), Departamento de Química Orgánica, Universidade de Santiago de Compostela Rúa de Jenaro de la Fuente, s/n, 15705 Santiago de Compostela A Coruña Spain manuel.nappi@usc.es https://nappichem.com

## Abstract

Alcohols are among the most widely occurring functional groups found in naturally abundant, biologically relevant organic compounds, which in many cases are considered feedstock chemicals. Herein, we report a metal-free method for the deoxygenative coupling of alcohol-derived benzoates and pyridines promoted by visible light. Given the practical, mild and water-compatible conditions, small molecules and DNA headpieces can be successfully functionalized with a range of primary, secondary and tertiary alcohols. This protocol is distinguished by its wide substrate scope and broad applicability, even in the context of late-stage functionalization and DNA–drug coupling reactions.

## Abstract 2

A visible-light-mediated metal-free method for the deoxygenative coupling of alcohol-derived benzoates and pyridines. Given the mild and water-compatible conditions, small molecules and DNA headpieces are functionalized with a wide range of alcohols.

## Graphical abstract

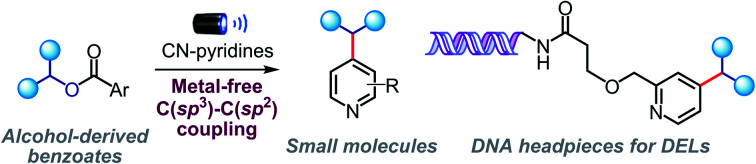

## Introduction

Carbon-centered sp^3^ radicals are useful intermediates for the installation of complex fragments in the synthesis of new organic molecules.^1^ These alkyl radicals are commonly generated from halide precursors; however, competing elimination and rearrangement reactions can often preclude the preparation of structurally complex halides. In contrast, alcohols are ideal precursors for alkyl radicals because they are stable, cheap, and feature prominently among feedstocks and naturally abundant organic compounds such as sugars and amino acids.^2,3^

**Fig. 1:**
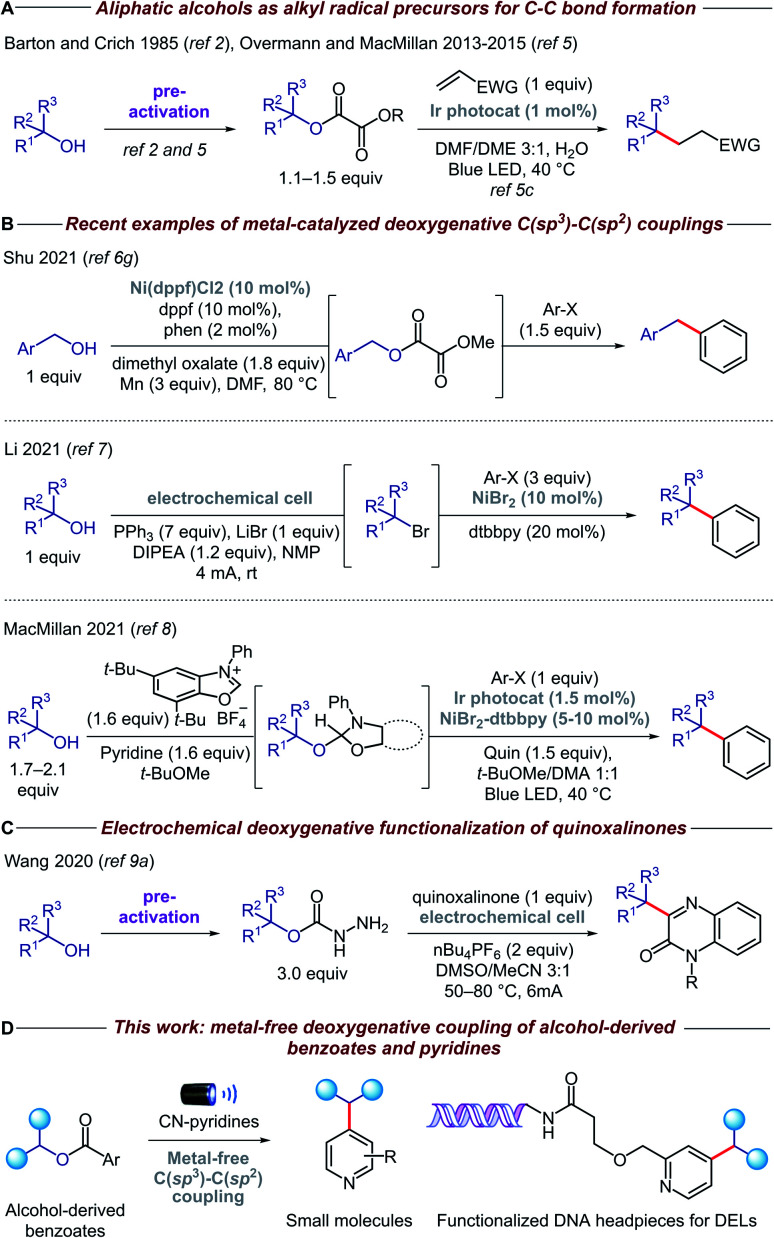
Alcohols as sp^3^ synthons in C(sp^3^)–C(sp^2^) couplings.

Recently, the advent of visible-light photoredox catalysis has offered a unique opportunity through which alkyl radicals can be generated from a range of organic molecules under mild reaction conditions.^4^ Based on the pioneering work of Overmann and MacMillan (Fig. 1A),^5^ several methodologies have been developed for the deoxygenative activation of alcohols, thereby enabling the use of open-shell species in a variety of C–C forming reactions.^6^ Particularly relevant among these are the methods that forge C(sp^3^)–C(sp^2^) bonds, allowing, for the first time, cross-coupling reactions between alcohols and heteroarenes (Fig. 1B). In addition, electrochemistry has emerged as a viable alternative for the deoxygenative formation of alkyl radicals, which has been coupled with nickel catalysis to enable C–C bond arylation.^7^ Very recently, the MacMillan group reported a powerful methodology for *in situ* activation and arylation of alcohols enabled by metallaphotoredox catalysis.^8^

Despite the advances in this area, the majority of deoxygenative methods for the synthesis of C(sp^3^)–C(sp^2^) bonds with heteroarenes rely on the use of transition metal complexes, either as coupling catalysts and/or as photocatalysts. Arguably, the price and toxicity of transition-metal complexes/ligands, and the challenging removal of metal residues from desired products, have driven recent efforts to develop metal-free organic transformations. Surprisingly, there is only an isolated example of metal-free deoxygenative functionalization of heteroarenes, albeit with limited functional group tolerance due to a combination of highly oxidizing conditions and use of activated heteroarenes such as quinoxalinone (Fig. 1C).^9^ Therefore, a general, mild, and practical metal-free deoxygenative method for the heteroarylation of aliphatic alcohols as sp^3^ synthons is highly desirable.

Herein, we describe the development of a general metal-free platform for the deoxygenative coupling of alcohol-derived benzoates and pyridines promoted by visible light (Fig. 1D). Feedstock and complex alcohols can be readily converted to the desired products in high yields, even in the case of top-selling pharmaceuticals such as haloperidol and ezetimibe. Given the mild reaction conditions and compatibility with diluted and aqueous conditions, our chemistry can be also utilized to functionalize DNA headpieces, allowing, for the first time, the use of alcohols as building blocks for the synthesis of DNA-encoded libraries (DELs).

## Design plan

**Fig. 2:**
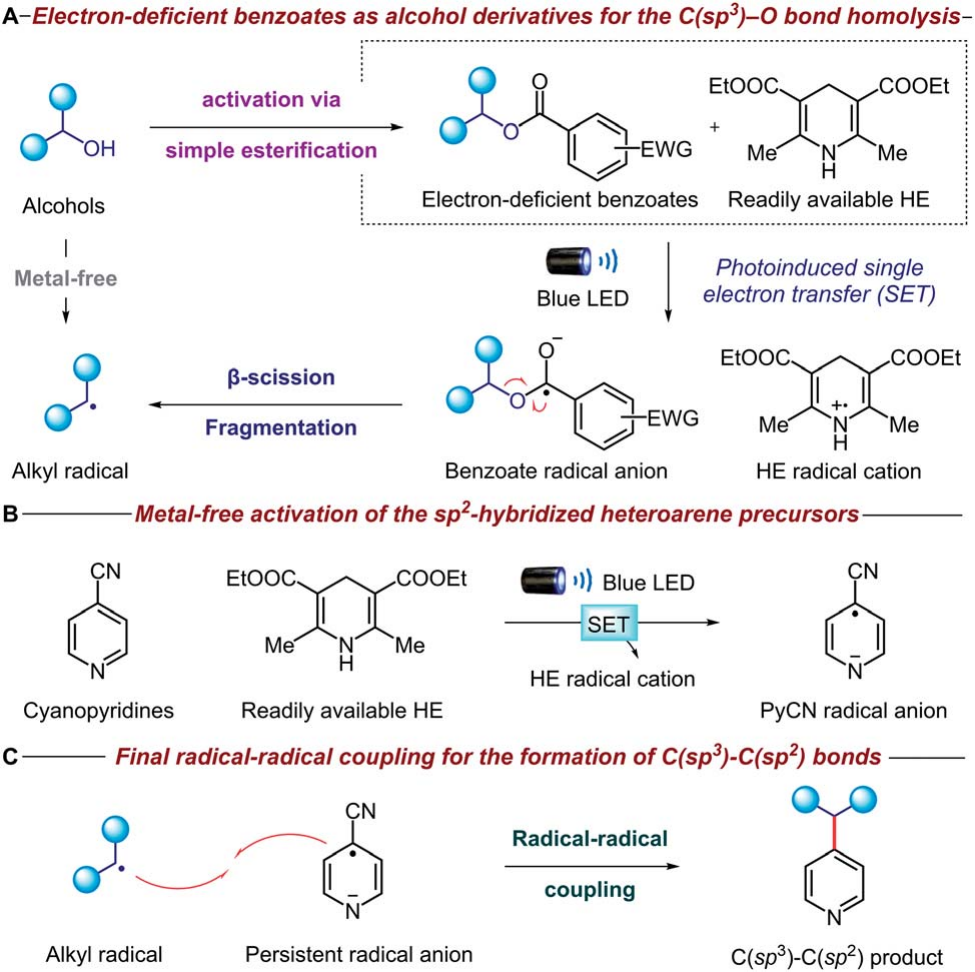
Strategy for a metal-free deoxygenative C(sp^3^)–C(sp^2^) coupling of alcohols and pyridines.

The mechanistic details of our proposed transformation are outlined in Fig. 2. Initially, the aliphatic alcohol is converted to the corresponding electron-deficient benzoate *via* a high-yielding esterification reaction. Under visible light irradiation, the commercially available Hantzsch ester (HE) can donate an electron (SET) to the benzoate ester *via* direct visible-light excitation or an electron donor–acceptor complex (Fig. 2A).^10^ The resulting benzoate radical anion undergoes β-scission fragmentation, providing the desired alkyl radical along with a benign carboxylate byproduct.^11^ It is noteworthy that the alcohol is activated through a new metal-free reductive process, which is complementary to many existing methodologies that oxidatively generate radicals from oxalate salts,^5*c*^ triphenylphosphonium salts,^7^ NHC^8^ and alkyl carbazates.^9*a*^ Concurrently, another equivalent of HE activates the pyridine precursor through a second photoinduced single electron transfer (Fig. 2B); cyano-substituted pyridines are known to undergo facile single electron reduction to the corresponding radical anions,^12^ although HE is rarely used.^13^ Remarkably, both the alcohol and pyridine metal-free activation steps occur simultaneously without any compatibility issues. Finally, radical–radical coupling between the alkyl radical derived from the initial alcohol and the persistent cyanopyridine radical anion forges the desired C(sp^3^)–C(sp^2^) bond (Fig. 2C). Given the strongly reducing conditions, we anticipated that oxidatively sensitive moieties such as tertiary amines and guanosines would be unaffected, thereby ensuring broad functional group tolerance and DNA compatibility.

## Results and discussion

### Optimization of the reaction conditions

We began our investigations by evaluating the efficiency of the alkyl radical formation from different 1-phenylpropanol benzoate ester derivatives 1–8 in the presence of 4-cyanopyridine 9, Hantzsch ester, sodium acetate, and DMSO under blue light irradiation (Table 1). While a color change was observed when nitro derivatives 1 or 2 were mixed with Hantzsch ester, indicating the formation of possible EDA complexes, no coupling product was formed under the reaction conditions. The nitro-benzoate esters were exclusively converted to the corresponding *N*-hydroxyanilines through the reduction of the nitro group. These results indicated that a photoinduced single electron transfer was operative under the reaction conditions, though the negative charge was mainly localized on the nitro group and not at the ester functionality required to promote the desired β-scission fragmentation.

**Table 1:** Evaluation of the benzoate ester structure^a^

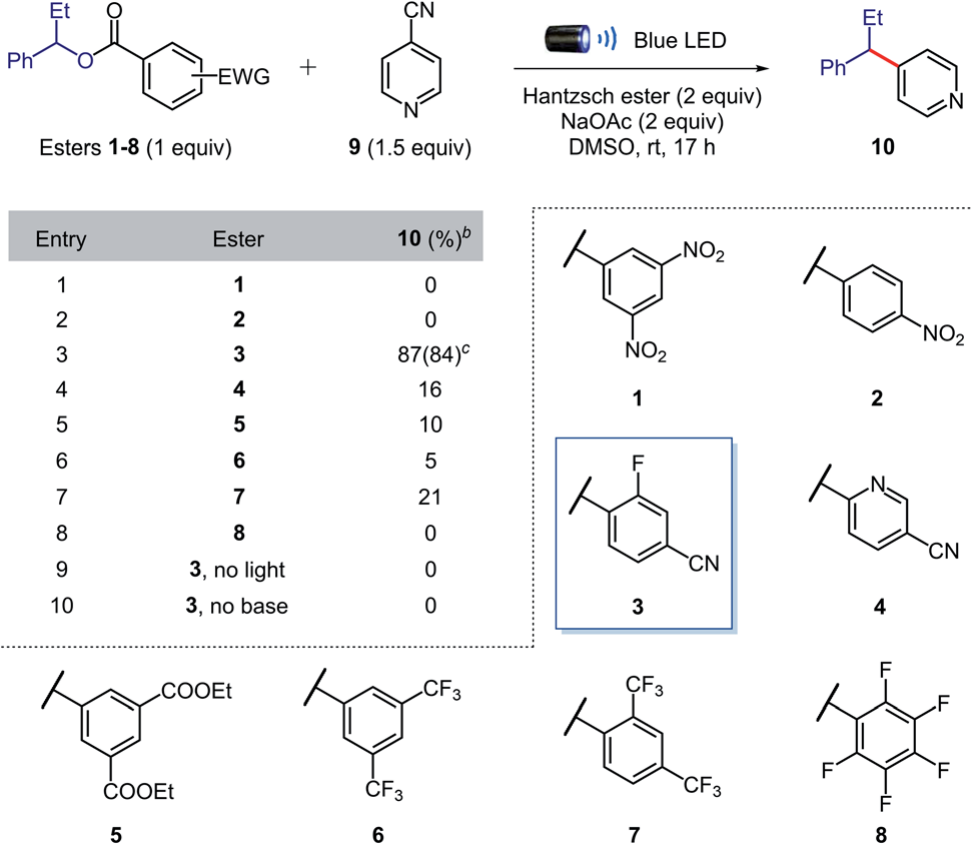

a Reaction conditions: 1–8 (0.1 mmol), 9 (0.15 mmol), Hantzsch ester (0.2 mmol), NaOAc (0.2 mmol) in DMSO (0.05 M) at rt for 17 h.

b NMR yields using 1,1,2,2-tetrachloroethane as the internal standard.

c In brackets isolated yield on 0.2 mmol scale.

Arguably, a balance between the electron-deficiency of the benzoate ester and localization of the negative charge in the carbonyl group was found with the 4-cyano-2-fluorobenzoate derivative 3. Indeed, we were pleased to find that benzoate ester 3 provided the desired coupled product 10 in 84% yield.

Importantly, in contrast to many existing methods,^2,5,6,8,9^ high yields of cross-coupled product were obtained using only 1 equivalent of preactivated alcohol. When other esters (4–8) were tested, the coupling product was obtained in generally low yields, confirming the superior reactivity of the 4-cyano-2-fluorobenzoate ester. Control experiments showed that light and sodium acetate were crucial for the formation of the desired product; the starting benzoate ester 3 was completely recovered in both cases, while 4-cyanopyridine 9 was intact only in the former. Therefore, we propose that sodium acetate favours β-scission fragmentation by deprotonating the radical cation of the Hantzsch ester (p*K*_a,calc_ in DMSO = 3.0)^14^ after SET or through proton-coupled electron transfer (PCET),^15^ thus inhibiting unproductive back electron transfer.

### Generality of the reaction for small molecules synthesis

**Fig. 3:**
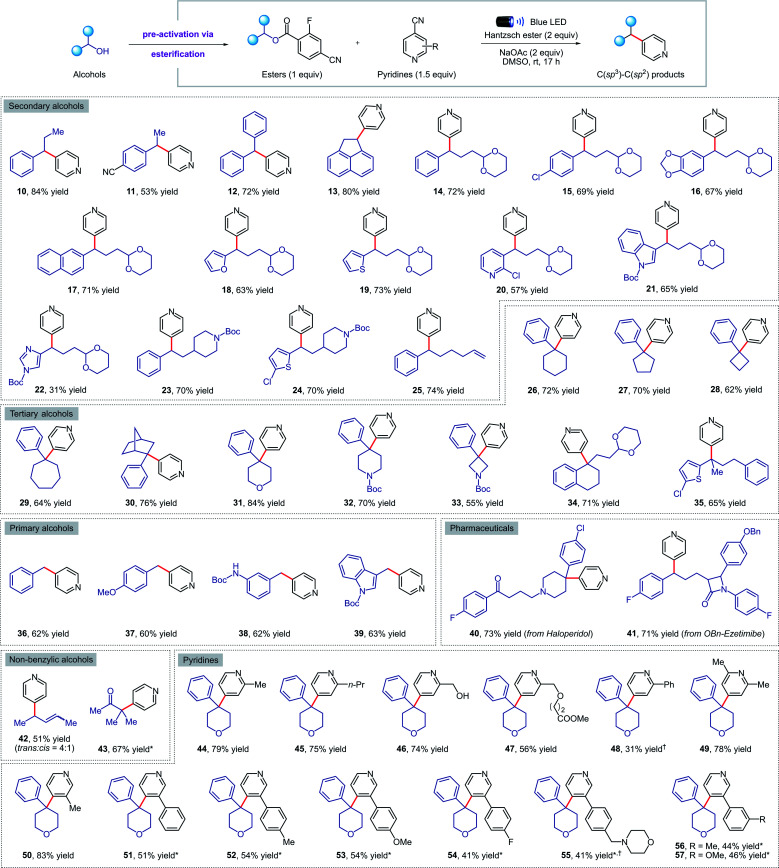
Metal-free deoxygenative coupling of alcohols and pyridines for small molecules synthesis. Reaction conditions as in Table 1 (entry 3). All yields are isolated on 0.2 mmol scale. ^*a*^ NMR yield using 1,1,2,2-tetrachloroethane as the internal standard. *See the ESI† for experimental details.

Next, we turned our attention to exploring the scope of our transformation for small molecule synthesis. As evident from the results compiled in Fig. 3, our visible-light-mediated metal-free deoxygenative coupling could be conducted with a wide variety of 4-cyano-2-fluorobenzoate esters derived from primary, secondary, and tertiary alcohols. Both electron withdrawing (11, 15, 41) and donating (16, 37, 38) substituents on the benzylic alcohols were well tolerated, delivering the corresponding products in good yields. Viable motifs in this transformation include furan (18), thiophene (19), pyridine (20), indole (21, 39), imidazole (22), 1,3-dioxanes (14–22, 34) and *N*-Boc protected amines (23, 24, 32, 33). Cyclic (26–34, 40) and acyclic (35) tertiary alcohols were readily converted to the coupling products despite their steric hindrance; such molecules would be hard to access using transition-metal catalysis. Importantly, we were able to apply our methodology for the late-stage functionalization of top-selling alcohol-containing pharmaceuticals such as haloperidol (40) and ezetimibe (41), obtaining the desired cross-coupled products in 73% and 71% yield respectively. The presence of a tertiary amine in haloperidol, which is normally not compatible with other photocatalytic conditions due to oxidative degradation, was not affected under our reaction conditions. Finally, we were delighted to find that this method is also amenable to non-benzylic substrates such as allylic (42) and tertiary α-keto alcohols (43); the latter represent the first example of a deoxygenative cross-coupling reaction with tertiary α-keto alcohols.

Pleasingly, our metal-free cross-coupling methodology was found to be applicable with a wide array of cyanopyridine derivatives. Substituents in the *ortho* position, including alkyl groups (44, 45, 49), free alcohols (46), and methyl esters (47) posed no problems, while the lower yield observed when using a 2-phenylpyridine derivative (48) could be explained by the highly stabilized nature of the radical anion, resulting in sluggish reactivity. On the other hand, alkyl and aryl meta-substituents were well tolerated under the reaction conditions, forming a variety of cross-coupled products in good yields (50–57). Notably, an electron-rich morpholine-containing pyridine (55) was found to be compatible, highlighting the broad functional group tolerance of our methodology.

### Mechanistic experiments

**Fig. 4:**
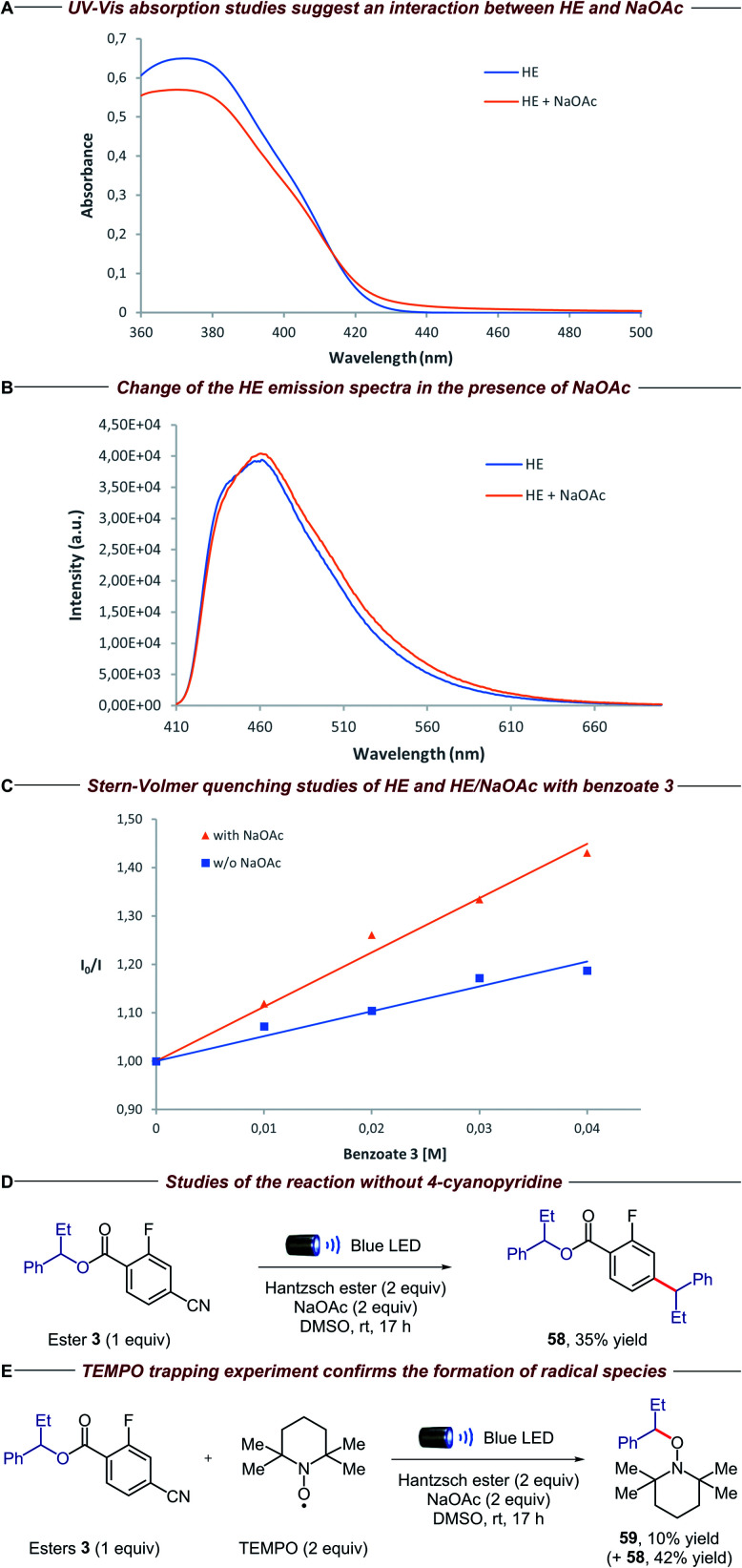
Mechanistic experiments: (A) [HE] = 1 × 10^−4^ M, [NaOAc] = saturated in DMSO (please see the ESI for details†); (B) excitation wavelength = 400 nm, [HE] = 1 × 10^−3^ M, [NaOAc] = saturated in DMSO; (C) excitation wavelength = 373 nm, [HE] = 1 × 10^−4^ M, [NaOAc] = saturated in DMSO; (D) isolated yield on 0.02 mmol scale; (E) NMR yields using 1,1,2,2-tetrachloroethane as the internal standard (product 59 was isolated to confirm the structure).

To gain insight into the mechanistic proposal detailed in Fig. 2, we conducted a series of preliminary experiments summarized in Fig. 4. Firstly, we recorded the UV-Vis absorption spectra of single reaction components and their combination to identify the formation of possible electron donor–acceptor complexes. While the mixture of the electron-rich Hantzsch ester (HE) and electron-deficient dinitro benzoate 1 clearly showed a new absorption band in the visible region, suggesting the formation of a charge-transfer complex, no additional band was observed when HE and 4-cyano-2-fluorobenzoate derivative 3 were mixed (please see the ESI† for details). Surprisingly, the HE absorption spectra clearly changed in the presence of NaOAc, suggesting a ground-state interaction between these two species (Fig. 4A). Emission studies showed a small but appreciable change in the spectra of HE after the addition of NaOAc (Fig. 4B), consistent with the UV-Vis experiments. In light of this, we conducted Stern–Volmer quenching studies for the HE and the HE/NaOAc mixture. In both cases we found a linear correlation between the amounts of benzoate 3 and the ratio *I*_0_/*I*, with a higher Stern–Volmer quenching constant for the HE/NaOAc system (Fig. 4C). Further UV-Vis absorption studies excluded a static quenching and therefore a ground-state ternary association between HE, NaOAc and benzoate 3 (please see the ESI for details†). Finally, when the standard reaction was conducted in the presence of 1,4-dinitrobenzene, a known competitor for single electron transfer processes due to its strong tendency to accept an electron (*E*_red_ = −0.64 V *vs.* SCE),^16^ ester 3 and 4-cyanopyridine 9 were completely recovered.

These findings, together with the control experiments in the absence of light and NaOAc (Table 1, entries 9 and 10), suggest that the HE, in combination with NaOAc, is the main photoactive species in the reaction:^15^ upon absorption of visible light, the excited HE–NaOAc complex (*E*_red_ = −2.28 V *vs.* SCE for HE)^17^ donates an electron to 4-cyano-2-fluorobenzoate 3, while 4-cyanopyridine 9 (*E*_red_ = −1.87 V *vs.* SCE)^18^ can also be reduced by excited HE alone (Table 1, entry 10).^13*b*,*c*^ The resulting radical anion of benzoate 3 is proposed to undergo β-scission fragmentation, providing the desired alkyl radical along with a benign carboxylate byproduct (Fig. 2A). Interestingly, when we conducted the reaction in the absence of 4-cyanopyridine 9, we observed the formation of compound 58 (Fig. 4D). Arguably, the latter is obtained *via* radical–radical coupling of 1-phenylpropyl radical and the radical anion of benzoate 3, before the desired β-scission fragmentation can occur. The formation of 1-phenylpropyl radical was confirmed when the same experiment was performed in the presence of TEMPO: trapping product 59 was detected along with adduct 58 (Fig. 4E).

### Applications in DNA-encoded libraries synthesis

Given the mild reaction conditions, we set out to explore the aqueous compatibility of our system for its application in the synthesis of DNA-encoded libraries (DELs). DELs are a powerful technology that has found widespread application in medicinal chemistry as a time- and cost-effective platform for the discovery of new therapeutic candidates.^19^ The key aspect is the conjugation of chemical compounds or building blocks to short DNA fragments that serve as identification bar codes, and in some cases also direct and control the chemical synthesis. To develop DELs platforms, on-DNA chemistries are required to incorporate multifunctional building-blocks from readily available chemicals under mild, dilute, and aqueous conditions. Recently, pioneering studies from the Baran, Flanagan, and Molander groups^20^ have highlighted the power of radical-based reactivity for DELs synthesis through visible-light photochemistry^21^ and RASS (reversible adsorption to solid support) technology.^22^*N*-(Acyloxy)phthalimides,^20*a*,21*d*,*f*,22*a*^ amino acids,^20*b*,*c*,21*a*,*e*^ carboxylic acids,^20*c*^ 4-alkyldihydropyridines,^20*c*^ silicates,^20*c*^ alkyl halides,^21*b*,*c*,*f*^ and α-TMS amines^21*c*^ have all been employed as alkyl radical precursors. However, to date, no general strategy for the deoxygenative coupling of alcohols and DNA substrates has been reported. Given the importance of DELs in medicinal chemistry and the paucity of on-DNA reactions available from abundant building blocks, a new method for the deoxygenative cross-coupling of alcohols on DNA headpieces would be of great value.

**Fig. 5:**
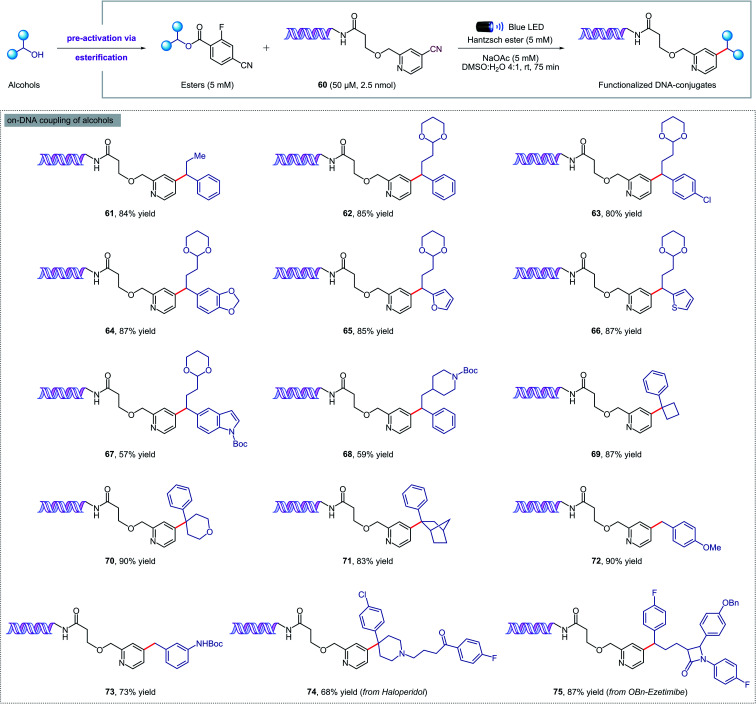
Metal-free deoxygenative coupling of alcohols and DNA–pyridine conjugate 60 for DELs synthesis. Starting DNA headpiece: 5′d Phos-GAGTCA-Spacer 9-Amino C7-Spacer 9-TGACTCCC 3′.

We were delighted to find that our new protocol could be used to functionalize DNA–pyridine conjugate 60 (50 μM, 2.5 nmol) with a variety of pre-activated alcohols (Fig. 5). Despite the diluted aqueous conditions, the key coupling process took place in just 75 minutes under visible light irradiation. The reactions can be set up under air, although flushing with an inert gas was required at the closure of the vial to ensure reproducible results. Pharmaceutical alcohols such as haloperidol and ezetimibe were successfully installed into the DNA-conjugate in high yields (74, 75), revealing that our methodology can also be used for DNA–drug coupling reactions. Remarkably, the DNA remained intact during the photochemical process and no oxidation was observed by mass spectrometry. Finally, a commercial DNA headpiece devoid of the cyanopyridine group was unaffected when exposed to the reaction conditions, confirming the excellent chemoselectivity of our methodology.

## Conclusions

In summary, we have developed a new, practical, metal-free platform for the deoxygenative coupling of alcohol-derived benzoates and pyridines promoted by visible light. Given the mild and water-compatible reaction conditions, our chemistry can be used to successfully functionalize small molecules and DNA headpieces. This protocol is distinguished by its wide substrate scope and broad applicability, even in the context of late-stage functionalization and DNA–drug coupling reactions. Overall, we believe that the flexibility and simplicity of the newly developed method will make this procedure of interest to chemists in both industrial and academic environments and, in particular, to practitioners of medical chemistry.

## Data availability

All the data supporting this article have been included in the main text and the ESI.†

## Author contributions

M. N. conceived the project and designed the experiments. M. N. did most of the experimental work: reaction optimization, alcohol scope, fluorescence quenching studies and DNA experiments. S. R. N. K. finished the alcohol scope, explored the reactivity of pyridines and carried out most of the mechanistic experiments. M. N. wrote the manuscript.

## Conflicts of interest

There are no conflicts to declare.

## Supplementary Material

SC-013-D2SC01621D-s001
