# Supplementary material for: Metal-free deoxygenative coupling of alcohol-derived benzoates and pyridines for small molecules and DNA-encoded libraries synthesis
Source: Chem Sci. 2022 May 10;13(23):6982–9. doi: 10.1039/d2sc01621d (PMC9200129; doi:10.1039/d2sc01621d)
Supplement: SC-013-D2SC01621D-s001 [file SC-013-D2SC01621D-s001.pdf]

Electronic Supplementary Material (ESI) for Chemical Science.
This journal is © The Royal Society of Chemistry 2022

Metal-free Deoxygenative Coupling of Alcohol-Derived Benzoates and Pyridines for Small Molecules and DNA-Encoded Libraries Synthesis

Sai Rohini Narayanan Kolusu and Manuel Nappi*

Email: manuel.nappi@usc.es

Centro Singular de Investigación en Química Biolóxica e Materiais Moleculares (CiQUS), Departamento de Química Orgánica, Universidade de Santiago de Compostela, Rúa de Jenaro de la Fuente, s/n, 15705 Santiago de Compostela, A Coruña, Spain.

Supporting Information

Table of Contents

1. General Information 3

2. Synthesis of starting materials 4

3. Metal-free deoxygenative coupling of alcohol-derived benzoates and pyridines for small molecules synthesis 41

4. Mechanistic experiments 68

5. Metal-free deoxygenative coupling of alcohol-derived benzoates and DNA headpieces for DEL synthesis 76

6. NMR traces 101

1. General Information

Proton nuclear magnetic resonance (^{1}H NMR) spectra were recorded at ambient temperature on Varian Mercury 300 MHz or Bruker AVIII 500 MHz spectrometers. Chemical shifts (δ) are reported in ppm and quoted to the nearest 0.01 ppm relative to the residual protons in $CDCl_3$ (7.26 ppm) and coupling constants (*J*) are quoted in Hertz (Hz). Data are reported as follows: Chemical shift (multiplicity, coupling constants, number of protons). Coupling constants were quoted to the nearest 0.1 Hz and multiplicity reported according to the following convention: s = singlet, d = doublet, t = triplet, q = quartet, qn = quintet, sp = septet, m = multiplet, br = broad. Where coincident coupling constants have been observed, the apparent (app) multiplicity of the proton resonance has been reported.

Carbon nuclear magnetic resonance (^{13}C NMR) spectra were recorded at ambient temperature on Varian Mercury 300 MHz or Bruker AVIII 500 MHz spectrometers. Chemical shift (δ) was measured in ppm and quoted to the nearest 0.1 ppm relative to the residual solvent peaks in $CDCl_3$ (77.16 ppm). DEPT135, nOe experiments and 2-dimensional experiments (COSY, HMBC and HSQC) were used to support assignments where appropriate.

High-resolution mass spectra (HRMS) were measured on Bruker micrOTOF spectrometer using direct injection APCI mode at the CACTUS facility of the University of Santiago de Compostela.

Analytical thin layer chromatography (TLC) was performed using pre-coated Merck glass backed silica gel plates (Silica gel 60 F254). Flash column chromatography was undertaken on silica gel (40-60 μm) under a positive pressure of air unless otherwise stated. Visualization was achieved using ultraviolet light (254 nm) and chemical staining with basic potassium permanganate solutions as appropriate.

Dichloromethane (DCM), Tetrahydrofuran (THF), dimethylformamide (DMF), Acetonitrile (MeCN) were dried and dispensed using solvent purification system. Dimethylsulfoxide (DMSO) was purchased from Sigma-Aldrich chemical company. All

reagents were purchased at the highest commercial quality and used without further purification. Diethylether (Et_2O) used as an eluent in flash column contained 200ppm of 2,6-Di-tert-butyl-4-methylphenol (BHT) as a stabilizer.

Reactions were carried out under an atmosphere of Argon unless otherwise stated. All reactions were monitored by TLC, ^{1}H NMR spectra taken from reaction samples (NMR yields determined by ^{1}H NMR with reference to 1,1,2,2-tetrachloroethane as an internal standard) or gas chromatography mass spectrometry (GC-MS) using Agilent 8890 (mass detector: Agilent 5977B GC/MSD).

Photochemical reactions were irradiated using Kessil A160WE LED Aquarium Light - Tuna Blue (40 W) as light source (settings: maximum blue and maximum intensity).

DNA headpiece **Sx** (5'd Phos-GAGTCA-Spacer 9-Amino C7-Spacer 9-TGACTCCC 3') was purchased from LGC Biosearch Technologies

2. Synthesis of starting materials

General procedure A: synthesis of benzoate esters via the acyl chloride

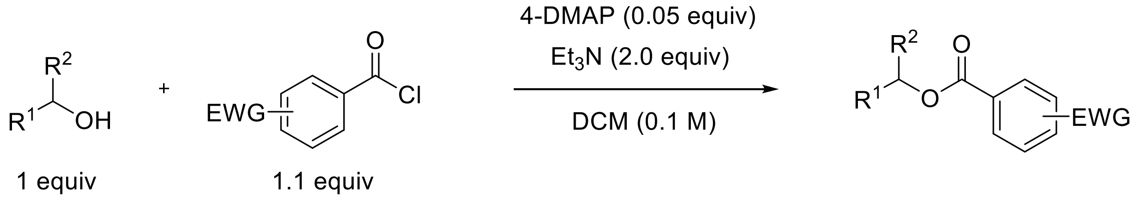

In a 100 mL round bottom flask equipped with stir bar, the appropriate alcohol (5.0 mmol, 1 equiv) was dissolved in dry DCM (50 mL) under argon atmosphere, followed by the addition of 4-(Dimethylamino)pyridine (4-DMAP) (18 mg, 0.15 mmol, 0.05 equiv) and triethylamine (Et_3N) (1.4 mL, 10 mmol, 2 equiv). The resulting suspension was cooled at 0 °C and the acyl chloride (5.5 mmol, 1.1 equiv) was slowly added. The reaction mixture was stirred at room temperature for 2 hours, solvent was evaporated under reduced pressure and the residue was purified by flash chromatography (silica gel) under the stated conditions to provide the pure alcohol derivative.

General procedure B: synthesis of benzoate esters via the carboxylic acid

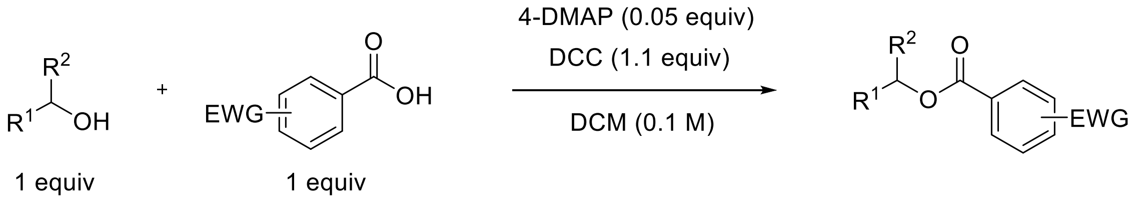

In a 100 mL round bottom flask equipped with stir bar, the appropriate alcohol (3.0 mmol, 1 equiv) was dissolved in dry DCM (30 mL) under argon atmosphere, followed by the addition of the benzoic acid (3.0 mmol, 1 equiv) and 4-(Dimethylamino)pyridine (4-DMAP) (18 mg, 0.15 mmol, 0.05 equiv). The resulting suspension was cooled at 0 °C and N,N-Dicyclohexylcarbodiimide (DCC) (681 mg, 3.3 mmol, 1.1 equiv) was added. The reaction mixture was stirred at room temperature for 17 hours, diluited with 20 mL of DCM and washed with: 5% HCl aqueous solution (20 mL), $NaHCO_3$ saturated aqueous solution (20 mL) and NaCl saturated aqueous solution (20 mL). The organic extract was dried ($MgSO_4$ or Na_2SO_4), filtered and concentrated *in vacuo*. The crude product was purified by flash column chromatography (silica gel) under the stated conditions to provide the pure alcohol derivative.

General procedure C: synthesis of benzoate esters from tertiary alcohols

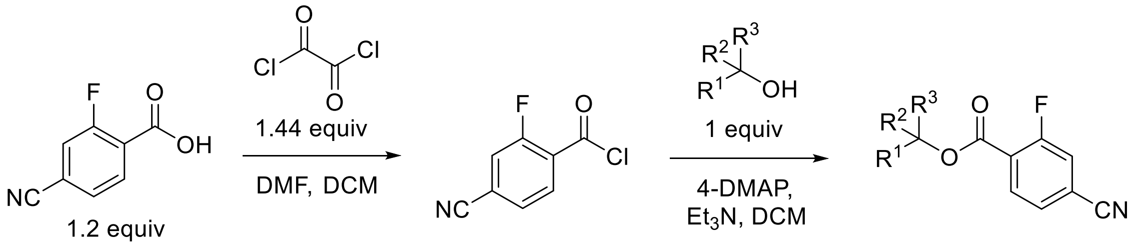

In a 100 mL round bottom flask equipped with stir bar, 4-cyano-2-fluorobenzoic acid (990 mg, 6.0 mmol, 1.2 equiv) and dry DCM (15 mL) were charged under argon atmosphere, followed by the addition of oxalyl chloride (0.610 mL, 7.2 mmol, 1.44 equiv) and 7 drops of DMF at rt. The resulting suspension was stirred for 1 hour (until CO_2 evolution stopped and the solution became omogeneous), solvent was evaporated under reduced pressure and the acyl chloride redissolved in 2 mL of dry DCM. Meanwhile, in a 100 mL round bottom flask equipped with stir bar, the appropriate tertiary alcohol (5.0 mmol, 1 equiv) was dissolved in dry DCM (13 mL) under argon

atmosphere, followed by the addition of 4-(Dimethylamino)pyridine (4-DMAP) (306 mg, 2.5 mmol, 0.5 equiv) and triethylamine (Et_3N) (0.836 uL, 6.0 mmol, 1.2 equiv). The resulting solution was cooled at 0 °C and the freshly prepared acyl chloride solution was added dropwise. The reaction mixture was stirred at room temperature for 24 hours, diluited with 20 mL of DCM and washed with: water (15 mL) and NaCl saturated aqueous solution (15 mL). The organic extract was dried ($MgSO_4$), filtered and concentrated *in vacuo*. The crude product was purified by flash column chromatography (silica gel) under the stated conditions to provide the pure alcohol derivative.

General procedure D: synthesis of secondary alcohols from aldehydes

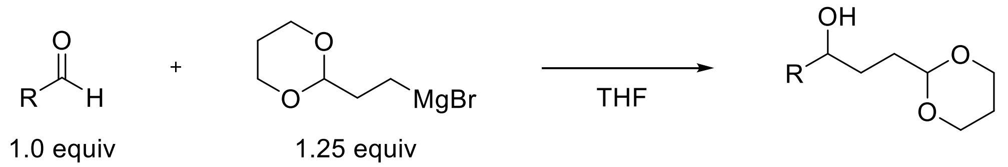

In a 25 mL round bottom flask equipped with stir bar, the appropriate aldehyde (2.0 mmol, 1 equiv) was dissolved in dry THF (4 mL) under argon atmosphere. The resulting solution was cooled at 0 °C and the (1,3-Dioxan-2-ylethyl)magnesium bromide solution (0.5 M in THF, 4.4 mL, 2.2 mmol, 1.1 equiv) was added dropwise. The reaction mixture was stirred at room temperature for 1 hour (until TLC shows the complete conversion of the aldehyde), quenched with NH_4Cl saturated aqueous solution at 0 °C (1 mL), diluted with water (10 mL) and Et_2O (10 mL). The organic phase was separated and the aqueous phase extracted with Et_2O (10 mL). The combined organic phases were washed with NaCl saturated aqueous solution (10 mL), dried over Na_2SO_4, filtered and concentrated *in vacuo*. The crude alcohol was used in the next step without additional purification unless otherwise stated.

General procedure E: synthesis of tertiary alcohols from ketones

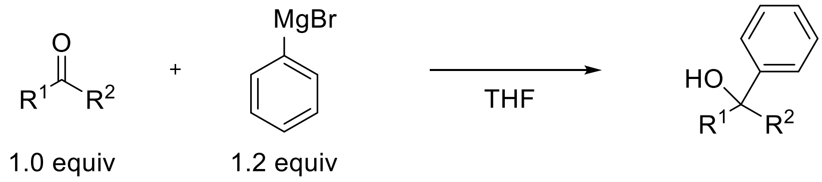

In a 100 mL round bottom flask equipped with stir bar, the appropriate ketone (10.0 mmol, 1 equiv) was dissolved in dry THF (10 mL) under argon atmosphere. The resulting solution was cooled at 0 °C and the phenylmagnesium bromide solution (3M in Et_2O, 4 mL, 12.0 mmol, 1.2 equiv) was added dropwise. The reaction mixture was stirred at room temperature for 1 hour (until TLC shows the complete conversion of the ketone), quenched with NH_4Cl saturated aqueous solution at 0 °C (3 mL), diluted with water (20 mL) and Et_2O (20 mL). The organic phase was separated and the aqueous phase extracted with Et_2O (20 mL). The combined organic phases were washed with NaCl saturated aqueous solution (15 mL), dried over $MgSO_4$, filtered and concentrated *in vacuo*. The crude alcohol was used in the next step without additional purification unless otherwise stated.

General procedure F: synthesis of 3-Aryl-4-cyanopyridine via Suzuki-Miyaura cross-coupling reaction

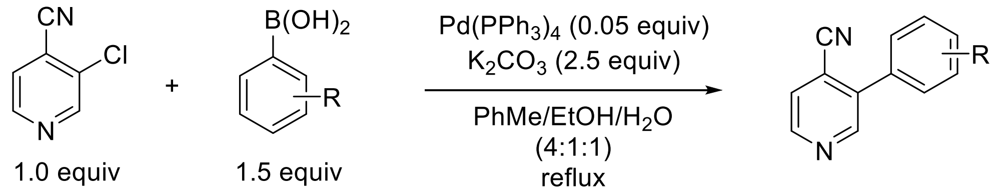

3-chloroisonicotinonitrile (1.0 equiv) was dissolved in toluene/ethanol/water (4:1:1, 10 mL). Arylboronic acid (1.5 equiv) and K_2CO_3 (2.50 equiv) were subsequently added. The mixture was purged with argon for 30 min, $Pd(PPh_3)_4$ (0.05equiv) was added and the reaction was refluxed overnight. The yellow solution was then allowed to cool down to rt and poured into water (40 mL). The aqueous phase was extracted with EtOAc (3 x 40 mL), then the combined extracts were washed with 1.0 M NaOH (50 mL), brine (50 mL) and dried over $MgSO_4$ and then concentrated in vacuo. The residue was then puried by flash chromatography (silica gel) under the stated conditions to provide the desired compound.

1-phenylpropyl 3,5-dinitrobenzoate (1)

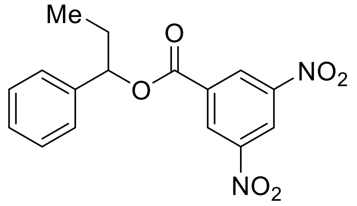

Prepared according to general procedure A using 1-phenyl-1-propanol (0.684 mL, 5.0 mmol) and 3,5-dinitrobenzoyl chloride (1.268 g, 5.5 mmol). The crude product was purified by flash column chromatography (95/5 to 80/20 Hexane/Et_2O) to provide the title compound as a white solid (1.290 mg, 78% yield). ^1H NMR (300 MHz, $CDCl_3$) δ: 9.24 – 9.19 (s, 1H), 9.19 – 9.12 (s, 2H), 7.49 – 7.29 (m, 5H), 5.99 (t, *J* = 7.0 Hz, 1H), 2.27 – 1.96 (m, 2H), 0.99 (t, *J* = 7.4 Hz, 3H). ^{13}C NMR (75 MHz, $CDCl_3$) δ: 162.05, 148.86, 139.23, 134.42, 129.54, 128.89, 128.77, 126.92, 122.46, 80.77, 29.22, 10.20; HRMS (APCI) found $[M]^+$ 330.0838, $C_{16}H_{14}N_2O_6$ requires 330.0846.

1-phenylpropyl 4-nitrobenzoate (2)

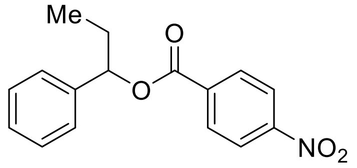

Prepared according to general procedure A using 1-phenyl-1-propanol (0.684 mL, 5.0 mmol) and 4-nitrobenzoyl chloride (1.021 g, 5.5 mmol). The crude product was purified by flash column chromatography (95/5 to 80/20 Hexane/Et_2O) to provide the title compound as a viscous oil (1.070 g, 75% yield). ^1H NMR (300 MHz, $CDCl_3$) δ: 8.29 (d, *J* = 9.1 Hz, 2H), 8.24 (d, *J* = 9.1 Hz, 2H), 7.46 – 7.28 (m, 5H), 5.95 (t, *J* = 6.9 Hz, 1H), 2.21 – 1.91 (m, 2H), 0.98 (t, *J* = 7.4 Hz, 3H); ^{13}C NMR (75 MHz, $CDCl_3$) δ: 164.14, 150.68, 139.97, 136.06, 130.84, 128.71, 128.35, 126.69, 123.65, 79.33, 29.46, 10.07; m/z HRMS (APCI) found $[M]^+$ 285.0986, $C_{16}H_{15}NO_4$ requires 285.0996.

1-phenylpropyl 4-cyano-2-fluorobenzoate (3)

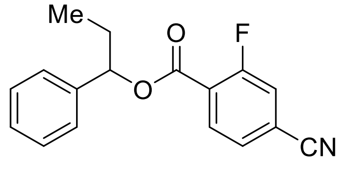

Prepared according to general procedure B using 1-phenyl-1-propanol (0.411 mL, 3.0 mmol) and 4-cyano-2-fluorobenzoic acid (495 mg, 3.0 mmol). The crude product was purified by flash column chromatography (90/10 to 70/30 Hexane/Et_2O) to provide the title compound as a white solid (654 mg, 77% yield). ^1H NMR (300 MHz, $CDCl_3$) δ: 8.04 (t, *J* = 7.3 Hz, 1H), 7.50 (dd, *J* = 8.1, 1.2 Hz, 1H), 7.45 (dd, *J* = 9.8, 1.2 Hz, 1H), 7.43 – 7.27 (m, 5H), 5.94 (t, *J* = 6.8 Hz, 1H), 2.16 – 2.02 (m, 1H), 2.02 – 1.88 (m, 1H), 0.96 (t, *J* = 7.4 Hz, 4H) ^{13}C NMR (75 MHz, $CDCl_3$) δ: 162.48 (d, *J* = 3.9 Hz), 161.39 (d, *J* = 263.6 Hz), 139.84, 133.34 (d, *J* = 1.4 Hz), 128.69, 128.34, 127.83 (d, *J* = 4.5 Hz), 126.75, 123.77 (d, *J* = 10.7 Hz), 121.04 (d, *J* = 26.2 Hz), 117.61 (d, *J* = 9.6 Hz), 116.80 (d, *J* = 2.5 Hz), 79.75, 29.58, 9.97; ^{19}F NMR (282 MHz, $CDCl_3$) δ: -105.9; HRMS (APCI) found $[M]^+$ 283.1005, $C_{17}H_{14}FNO_2$ requires 283.1003.

1-phenylpropyl 5-cyanopicolinate (4)

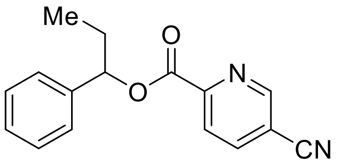

Prepared according to general procedure B using 1-phenyl-1-propanol (0.411 mL, 3.0 mmol) and 5-Cyanopyridine-2-carboxylic acid (444 mg, 3.0 mmol). The crude product was purified by flash column chromatography (90/10 to 70/30 Hexane/Et_2O) to provide the title compound as a white solid (606 mg, 76% yield). ^1H NMR (300 MHz, $CDCl_3$) δ: 9.06 – 8.94 (m, 1H), 8.27 – 8.17 (m, 1H), 8.16 – 8.03 (m, 1H), 7.48 – 7.40 (m, 2H), 7.40 – 7.26 (m, 3H), 5.98 (t, *J* = 7.0 Hz, 1H), 2.25 – 1.93 (m, 2H), 0.97 (t, *J* = 7.4 Hz, 3H); ^{13}C NMR (75 MHz, $CDCl_3$) δ: 163.21, 152.47, 151.14, 140.58, 139.66, 128.70, 128.43, 126.87, 124.83, 115.97, 112.82, 80.13, 29.28, 10.16; m/z HRMS (APCI) found $[M]^+$ 266.1044, $C_{16}H_{14}N_2O_2$ requires 266.1050.

1,3-diethyl 5-(1-phenylpropyl) benzene-1,3,5-tricarboxylate (5)

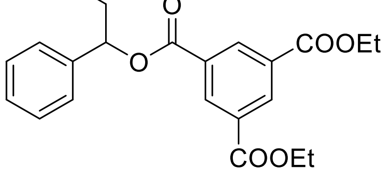

Prepared according to general procedure B using 1-phenyl-1-propanol (0.411 mL, 3.0 mmol) and diethyl 1,3,5-benzenetricarboxylate (799 mg, 3.0 mmol). The crude product

was purified by flash column chromatography (90/10 to 70/30 Hexane/Et_2O) to provide the title compound as a viscous oil (818 mg, 71% yield). ^{1}H NMR (300 MHz, $CDCl_3$) δ: 8.90 – 8.86 (m, 2H), 8.86 – 8.83 (m, 1H), 7.47 – 7.40 (m, 2H), 7.40 – 7.33 (m, 2H), 7.33 – 7.26 (m, 1H), 5.95 (t, *J* = 6.9 Hz, 1H), 4.44 (q, *J* = 7.1 Hz, 4H), 2.22 – 1.89 (m, 2H), 1.43 (t, *J* = 7.1 Hz, 6H), 0.98 (t, *J* = 7.4 Hz, 3H); ^{13}C NMR (75 MHz, $CDCl_3$) δ: 165.19, 164.57, 140.23, 134.62, 134.58, 131.66, 128.65, 128.22, 126.75, 79.09, 61.82, 29.51, 14.42, 10.14; m/z HRMS (APCI) found $[M]^+$ 384.1563, $C_{22}H_{24}O_6$ requires 384.1567.

1-phenylpropyl 3,5-bis(trifluoromethyl)benzoate (6)

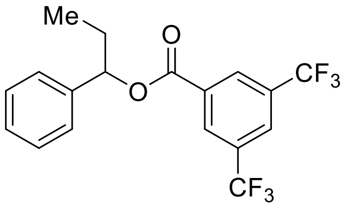

Prepared according to general procedure A using 1-phenyl-1-propanol (0.684 mL, 5.0 mmol) and 3,5-bis(trifluoromethyl)benzoyl chloride (1.521 g, 5.5 mmol). The crude product was purified by flash column chromatography (95/5 to 90/10 Hexane/Et_2O) to provide the title compound as a viscous oil (1.466 g, 78% yield). ^{1}H NMR (300 MHz, $CDCl_3$) δ: 8.51 (s, 2H), 8.07 (s, 1H), 7.47 – 7.28 (m, 5H), 5.97 (t, *J* = 7.0 Hz, 1H), 2.26 – 1.93 (m, 2H), 0.99 (t, *J* = 7.4 Hz, 3H); ^{13}C NMR (75 MHz, $CDCl_3$) δ: 163.45, 139.77, 132.92, 132.38 (q, *J* = 34.0 Hz), 130.03 – 129.59 (m), 128.79, 128.51, 126.82, 126.67 – 126.26 (m), 123.06 (q, *J* = 272.9 Hz), 79.81, 29.35, 10.16; ^{19}F NMR (282 MHz, $CDCl_3$) δ: -63.00; m/z HRMS (APCI) found $[M]^+$ 376.0903, $C_{18}H_{14}F_6O_2$ requires 376.0893.

1-phenylpropyl 2,4-bis(trifluoromethyl)benzoate (7)

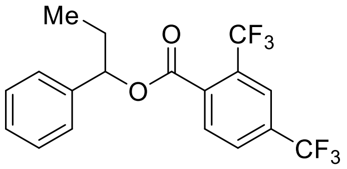

Prepared according to general procedure B using 1-phenyl-1-propanol (0.411 mL, 3.0 mmol) and 2,4-Bis(trifluoromethyl)benzoic acid (774 mg, 3.0 mmol). The crude product was purified by flash column chromatography (95/5 to 90/10 Hexane/Et_2O) to provide the title compound as a viscous oil (868 mg, 77% yield). ^{1}H NMR (300 MHz, $CDCl_3$) δ:

8.00 (s, 1H), 7.94 – 7.78 (m, 2H), 7.44 – 7.28 (m, 5H), 5.94 (t, *J* = 7.0 Hz, 1H), 2.22 – 1.88 (m, 2H), 0.95 (t, *J* = 7.4 Hz, 3H); ^{13}C NMR (75 MHz, $CDCl_3$) δ: 165.12, 139.38, 135.25, 133.34 (q, *J* = 33.9 Hz), 131.04, 129.71 (q, *J* = 33.8 Hz), 129.05 – 128.75 (m), 128.69, 128.49, 127.03, 124.36 – 123.76 (m), 123.04 (q, *J* = 272.7 Hz), 122.75 (q, *J* = 274.0 Hz), 80.51, 29.04, 9.94; ^{19}F NMR (282 MHz, $CDCl_3$) δ: -59.5, -63.3; m/z HRMS (APCI) found $[M]^+$ 376.0897, $C_{18}H_{14}F_6O_2$ requires 376.0893.

1-phenylpropyl 2,3,4,5,6-pentafluorobenzoate (8)

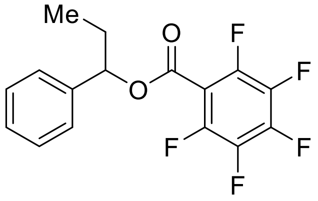

Prepared according to general procedure B using 1-phenyl-1-propanol (0.411 mL, 3.0 mmol) and pentafluorobenzoic acid (636 mg, 3.0 mmol). The crude product was purified by flash column chromatography (95/5 to 90/10 Hexane/Et_2O) to provide the title compound as a colorless oil (723 mg, 73% yield). ^{1}H NMR (300 MHz, $CDCl_3$) δ: 7.43 – 7.28 (m, 5H), 5.94 (t, *J* = 6.8 Hz, 1H), 2.17 – 1.87 (m, 2H), 0.97 (t, *J* = 7.4 Hz, 3H); ^{13}C NMR (75 MHz, $CDCl_3$) δ: 158.53, 139.35, 128.71, 128.48, 126.80, 80.73, 29.45, 9.92 (*note: some carbon peaks are not reported due to the multiple coupling with fluorine atoms in the pentafluorobenzene system*); ^{19}F NMR (282 MHz, $CDCl_3$) δ: -138.2, -149.0, -160.5; m/z HRMS (APCI) found $[M]^+$ 330.0669, $C_{16}H_{11}F_5O_2$ requires 330.0674.

1-(4-cyanophenyl)ethyl 4-cyano-2-fluorobenzoate (S1)

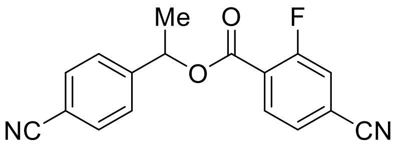

Prepared according to the general procedure B using 4-(1-hydroxyethyl)benzonitrile (441 mg, 3.0 mmol) and 4-cyano-2-fluorobenzoic acid (545 mg, 3.3 mmol). The crude product was purified by flash column chromatography (80/20 to 60/40 hexane/Et_2O) to provide the title compound as a white solid (706 mg, 80% yield). ^{1}H NMR (300 MHz, $CDCl_3$) δ 8.16 – 7.97 (t, *J* = 7.2 Hz 1H), 7.79 – 7.62 (m, 2H), 7.60 – 7.45 (m, 4H), 6.17 (q, *J* = 6.7 Hz, 1H), 1.70 (d, *J* = 6.6 Hz, 3H). ^{13}C NMR (75 MHz, $CDCl_3$) δ 162.18 (d, *J*

= 3.7 Hz), 161.35 (d, J = 263.9 Hz) 146.17, 133.33 (d, J = 1.6 Hz), 132.71, 127.93 (d, J = 4.5 Hz), 126.83, 123.05 (d, J = 10.4 Hz), 121.10 (d, J = 26.2 Hz), 118.54, 118.01 (d, J = 9.7 Hz), 116.62 (d, J = 2.6 Hz), 112.27. ^{19}F NMR (282 MHz, $CDCl_3$) δ -105.73 (dd, J = 9.8, 7.0 Hz). HRMS (APCI) $[M+H]^+$ found 295.0874, $C_{17}H_{11}FN_2O_2$ requires 295.0877.

Benzhydryl 4-cyano-2-fluorobenzoate (S2)

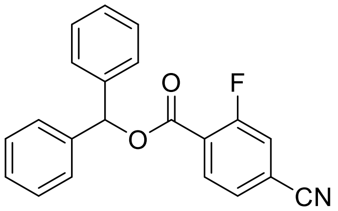

Prepared according to general procedure B using diphenylmethanol (553 mg, 3.0 mmol) and 4-cyano-2-fluorobenzoic acid (495 mg, 3.0 mmol). The crude product was purified by flash column chromatography (95/5 to 80/20 Hexane/Et_2O) to provide the title compound as a white solid (716 mg, 72% yield). ^{1}H NMR (300 MHz, $CDCl_3$) δ: 8.11(dd, J = 8.0, 7.0 Hz, 1H), 7.55-7.42 (m, 6H), 7.42-7.27 (m, 6H), 7.15 (s, 1H); ^{13}C NMR (75 MHz, $CDCl_3$) δ: 162.13 (d, J = 3.9 Hz), 161.47 (d, J = 263.9 Hz), 139.65, 133.50 (d, J = 1.7 Hz), 128.80, 128.37, 127.88 (d, J = 4.4 Hz), 127.22, 123.33 (d, J = 10.3 Hz), 121.10 (d, J = 26.2 Hz), 117.87 (d, J = 9.5 Hz), 116.73 (d, J = 2.6 Hz), 79.06; ^{19}F NMR (282 MHz, $CDCl_3$) δ: -105.3; HRMS (APCI) found $[M]^+$ 331.1002, $C_{21}H_{14}FNO_2$ requires 331.1003.

1,2-dihydroacenaphthylen-1-yl 4-cyano-2-fluorobenzoate (S3)

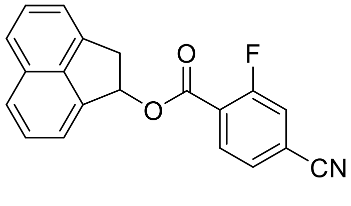

Prepared according to general procedure B using 1-Acenaphthenol (511 mg, 3.0 mmol) and 4-cyano-2-fluorobenzoic acid (495 mg, 3.0 mmol). The crude product was purified by flash column chromatography (95/5 to 85/15 Hexane/Et_2O) to provide the title compound as a white solid (638 mg, 67% yield). ^{1}H NMR (500 MHz, $CDCl_3$) δ: 8.04 (app t, J = 7.5 Hz, 1H), 7.82 (d, J = 8.1 Hz, 1H), 7.71 (d, J = 8.3 Hz, 1H), 7.64 (d, J =

6.9 Hz, 1H), 7.57 (app t, J = 7.6 Hz, 1H), 7.53 (app t, J = 7.6 Hz, 1H), 7.48 (app d, J = 8.0 Hz, 1H), 7.43 (app d, J = 9.8, 1H), 7.37 (d, J = 6.8 Hz, 1H), 6.91 (dd, J = 7.4, 2.3 Hz, 1H), 3.98 (dd, J = 17.9, 7.4 Hz, 1H), 3.54 – 3.49 (d, J = 17.9 Hz,1H).; ^{13}C NMR (125 MHz, $CDCl_3$) δ: 162.98 (d, J = 3.9 Hz), 161.46 (d, J = 264.5 Hz), 141.29, 140.85, 138.07, 133.25 (d, J = 1.7 Hz), 131.35, 128.45, 128.25, 127.80 (d, J = 4.5 Hz), 125.99, 123.44 (d, J = 9.9 Hz), 123.24, 122.28, 121.02 (d, J = 25.9 Hz), 120.14, 117.76 (d, J = 9.9 Hz), 116.74 (d, J = 2.7 Hz), 77.77, 38.84; ^{19}F NMR (470 MHz, $CDCl_3$) δ: -106.1; HRMS (APCI) found $[M+1]^+$ 318.0925, $C_{20}H_{12}FNO_2$ requires 318.0925.

3-(1,3-dioxan-2-yl)-1-phenylpropyl 4-cyano-2-fluorobenzoate (S4)

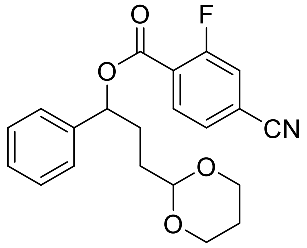

1st step. 3-(1,3-dioxan-2-yl)-1-phenylpropan-1-ol: prepared according to general procedure D using benzaldehyde (0.203 mL, 2.0 mmol) and (1,3-Dioxan-2-ylethyl)magnesium bromide solution (0.5 M in THF, 4.4 mL, 2.2 mmol, 1.1 equiv.). The crude alcohol was used in the next step without additional purification.

2nd step. Prepared according to general procedure B using crude 3-(1,3-dioxan-2-yl)-1-phenylpropan-1-ol and 4-cyano-2-fluorobenzoic acid (330 mg, 2.0 mmol). The crude product was purified by flash column chromatography (80/20 to 60/40 Hexane/Et_2O) to provide the title compound as a white solid (465 mg, 63% overall yield). ^{1}H NMR (300 MHz, $CDCl_3$) δ: 8.04 (app t, J = 7.6 Hz, 1H), 7.56-7.24 (m, 7H), 6.03 (app t, J = 6.9 Hz, 1H), 4.55 (m, 1H), 4.08 (dd, J = 11.6, 4.8 Hz, 2H), 3.73 (td, J = 11.6, 2.1 Hz, 2H), 2.27-1.94 (m, 3H), 1.81-1.60 (m, 2H), 1.32 (d, J = 13.5 Hz, 1H); ^{13}C NMR (75 MHz, $CDCl_3$) δ: 162.31 (d, J = 3.9 Hz), 161.37 (d, J = 263.7 Hz), 139.81, 133.35, 128.73, 128.37, 127.80 (d, J = 4.4 Hz), 126.66, 123.63 (d, J = 10.3 Hz), 121.02 (d, J = 26.2 Hz), 117.62 (d, J = 9.5 Hz), 116.78 (d, J = 2.6 Hz), 101.61, 78.13, 67.00, 31.18, 30.87, 25.86; ^{19}F NMR (282 MHz, $CDCl_3$) δ: -105.8; HRMS (APCI) found $[M-1]^+$ 368.1302, $C_{21}H_{19}FNO_4$ requires 368.1293.

1-(4-chlorophenyl)-3-(1,3-dioxan-2-yl)propyl 4-cyano-2-fluorobenzoate (S5)

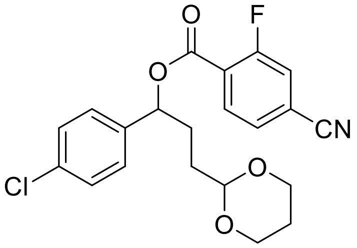

1st step. 1-(4-chlorophenyl)-3-(1,3-dioxan-2-yl)propan-1-ol: prepared according to general procedure D using 4-chlorobenzaldehyde (281 mg, 2.0 mmol) and (1,3-Dioxan-2-ylethyl)magnesium bromide solution (0.5 M in THF, 4.4 mL, 2.2 mmol, 1.1 equiv.). The crude alcohol was used in the next step without additional purification.

2nd step. Prepared according to general procedure B using crude 1-(4-chlorophenyl)-3-(1,3-dioxan-2-yl)propan-1-ol and 4-cyano-2-fluorobenzoic acid (533 mg, 2.0 mmol). The crude product was purified by flash column chromatography (80/20 to 60/40 Hexane/Et_2O) to provide the title compound as a white solid (465 mg, 66% overall yield). ^1H NMR (300 MHz, $CDCl_3$) δ: δ 8.02 (t, *J* = 7.5 Hz, 1H), 7.50 (dd, *J* = 8.1, 0.9 Hz, 1H), 7.45 (dd, *J* = 9.8, 1.1 Hz, 1H), 7.38 – 7.28 (m, 4H), 5.99 (dd, *J* = 7.3, 6.2 Hz, 1H), 4.55 (t, *J* = 4.9 Hz, 1H), 4.07 (dd, *J* = 10.9, 4.9 Hz, 2H), 3.72 (td, *J* = 12.2, 2.1 Hz, 2H), 2.24 – 1.94 (m, 3H), 1.78 – 1.55 (m, 2H), 1.32 (d, *J* = 13.5 Hz, 1H); ^{13}C NMR (75 MHz, $CDCl_3$) δ: 162.19 (d, *J* = 3.8 Hz), 161.26 (d, *J* = 263.8 Hz), 138.31, 134.08, 133.24, 128.85, 128.03, 127.78 (d, *J* = 4.4 Hz), 123.29 (d, *J* = 10.4 Hz), 120.97 (d, *J* = 26.2 Hz), 117.67 (d, *J* = 9.7 Hz), 116.66 (d, *J* = 2.5 Hz), 101.37, 77.29, 66.90, 30.97, 30.61, 25.76; ^{19}F NMR (282 MHz, $CDCl_3$) δ: -105.8; HRMS (APCI) found $[M-1]^+$ 402.0908, $C_{21}H_{18}ClFNO_4$ requires 402.0903.

1-(benzo[d][1,3]dioxol-5-yl)-3-(1,3-dioxan-2-yl)propyl 4-cyano-2-fluorobenzoate (S6)

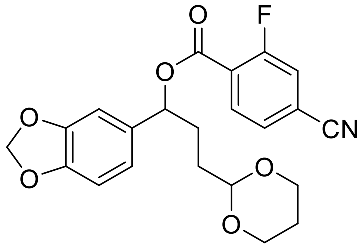

1st step. 1-(benzo[d][1,3]dioxol-5-yl)-3-(1,3-dioxan-2-yl)propan-1-ol: prepared according to general procedure D using piperonal (300 mg, 2.0 mmol) and (1,3-Dioxan-2-ylethyl)magnesium bromide solution (0.5 M in THF, 4.4 mL, 2.2 mmol, 1.1 equiv.). The crude alcohol was used in the next step without additional purification.

2nd step. Prepared according to general procedure B using crude 1-(benzo[d][1,3]dioxol-5-yl)-3-(1,3-dioxan-2-yl)propan-1-ol and 4-cyano-2-fluorobenzoic acid (330 mg, 2.0 mmol). The crude product was purified by flash column chromatography (70/30 to 50/50 Hexane/Et_2O) to provide the title compound as a white solid (604 mg, 73% overall yield). ^{1}H NMR (300 MHz, $CDCl_3$) δ: 8.01 (app t, *J* = 7.5 Hz, 1H), 7.49 (d, *J* = 8.1 Hz, 1H), 7.43 (d, *J* = 9.7 Hz, 1H), 6.89 (s, 1H), 6.87 (d, *J* = 7.8 Hz, 1H), 6.75 (d, *J* = 7.8 Hz, 1H), 5.93 (s, 2H), 4.54 (t, *J* = 4.9 Hz, 1H), 4.07 (dd, *J* = 11.3, 4.5 Hz, 2H), 3.72 (t, *J* = 11.3 Hz, 2H), 2.19-1.92 (m, 3H), 1.74-1.56 (m, 2H), 1.31 (d, *J* = 13.5 Hz, 1H); ^{13}C NMR (75 MHz, $CDCl_3$) δ: 162.25 (d, *J* = 3.8 Hz), 161.34 (d, *J* = 263.8 Hz), 147.99, 147.65, 133.60, 133.29, 127.78 (d, *J* = 4.4 Hz), 123.61 (d, *J* = 10.5 Hz), 120.99 (d, *J* = 26.1 Hz), 120.65, 117.59 (d, *J* = 9.7 Hz), 116.77 (d, *J* = 2.6 Hz), 108.32, 107.10, 101.56, 101.26, 78.01, 66.98, 31.19, 30.75, 25.84; ^{19}F NMR (282 MHz, $CDCl_3$) δ: -105.9; HRMS (APCI) found $[M]^+$ 413.1285, $C_{22}H_{20}FNO_6$ requires 413.1275.

3-(1,3-dioxan-2-yl)-1-(naphthalen-2-yl)propyl 4-cyano-2-fluorobenzoate (S7)

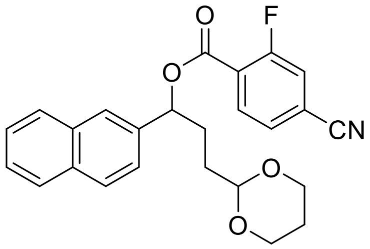

1st step. 3-(1,3-dioxan-2-yl)-1-(naphthalen-2-yl)propan-1-ol: prepared according to general procedure D using 2-Naphthaldehyde (312 mg, 2.0 mmol) and (1,3-Dioxan-2-ylethyl)magnesium bromide solution (0.5 M in THF, 4.4 mL, 2.2 mmol, 1.1 equiv.). The crude alcohol was used in the next step without additional purification.

2nd step. Prepared according to general procedure B using crude 3-(1,3-dioxan-2-yl)-1-(naphthalen-2-yl)propan-1-ol and 4-cyano-2-fluorobenzoic acid

(330 mg, 2.0 mmol). The crude product was purified by flash column chromatography (80/20 to 60/40 Hexane/Et_2O) to provide the title compound as a resin-like solid (570 mg, 68% overall yield). ^{1}H NMR (300 MHz, $CDCl_3$) δ: 8.01 (app t, *J* = 7.6 Hz, 1H), 7.91 – 7.72 (m, 4H), 7.54 (dd, *J* = 8.5, 1.1 Hz, 1H), 7.50 – 7.35 (m, 4H), 6.21 (t, *J* = 6.8 Hz, 1H), 4.56 (t, *J* = 4.9 Hz, 1H), 4.06 (dd, *J* = 11.2, 4.5 Hz, 2H), 3.71 (td, *J* = 12.0, 2.1 Hz, 2H), 2.37 – 1.93 (m, 3H), 1.85 – 1.63 (m, 2H), 1.29 (d, *J* = 13.3 Hz, 1H); ^{13}C NMR (125 MHz, $CDCl_3$) δ: 162.18, 161.11 (d, *J* = 260.8 Hz), 137.00, 133.12, 133.05, 128.50, 128.03, 127.66, 127.63, 126.29, 126.20, 125.86, 124.05, 123.33 (d, *J* = 10.4 Hz), 120.81 (d, *J* = 26.1 Hz), 117.37 (d, *J* = 9.7 Hz), 116.64 (d, *J* = 2.4 Hz), 101.39, 78.07, 66.78, 31.06, 30.60, 25.68; ^{19}F NMR (470 MHz, $CDCl_3$) δ: -105.8; HRMS (APCI) found $[M]^+$ 419.1529, $C_{25}H_{22}FNO_4$ requires 419.1527.

3-(1,3-dioxan-2-yl)-1-(furan-2-yl)propyl 4-cyano-2-fluorobenzoate (S8)

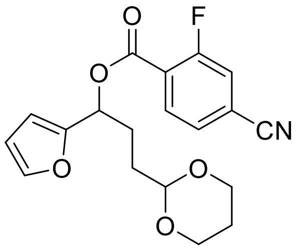

1st step. 3-(1,3-dioxan-2-yl)-1-(furan-2-yl)propan-1-ol: prepared according to general procedure D using furfural (0.166 mL, 2.0 mmol) and (1,3-Dioxan-2-ylethyl)magnesium bromide solution (0.5 M in THF, 4.4 mL, 2.2 mmol, 1.1 equiv.). The crude alcohol was used in the next step without additional purification.

2nd step. Prepared according to general procedure B using crude 3-(1,3-dioxan-2-yl)-1-(furan-2-yl)propan-1-ol and 4-cyano-2-fluorobenzoic acid (330 mg, 2.0 mmol). The crude product was purified by flash column chromatography (80/20 to 60/40 Hexane/Et_2O) to provide the title compound as viscous oil (474 mg, 66% overall yield). ^{1}H NMR (300 MHz, $CDCl_3$) δ: 8.01 (app t, *J* = 7.4 Hz, 1H), 7.48 (d, *J* = 8.1 Hz, 1H), 7.42 (d, *J* = 9.7 Hz, 1H), 7.38 (app s, 1H), 6.42 (d, *J* = 3.1 Hz, 1H), 6.34 (dd, *J* = 3.1, 1.5 Hz, 1H), 6.12 (t, *J* = 7.1 Hz, 1H), 4.56 (t, *J* = 4.9 Hz, 1H), 4.08 (dd, *J* = 11.0, 4.9 Hz, 2H), 3.76 (app t, *J* = 11.0 Hz, 2H), 2.30 – 1.96 (m, 3H), 1.80 – 1.57 (m, 2H), 1.32 (d, *J* = 13.4 Hz, 1H); ^{13}C NMR (75 MHz, $CDCl_3$) δ: 162.14 (d, *J* = 3.7 Hz),

161.40 (d, *J* = 264.2 Hz), 151.75, 142.91, 133.28, 127.76 (d, *J* = 4.6 Hz), 123.49 (d, *J* = 9.9 Hz), 120.98 (d, *J* = 26.0 Hz), 117.64 (d, *J* = 9.7 Hz), 116.79 (d, *J* = 2.6 Hz), 110.44, 109.42, 101.48, 70.52, 67.01, 31.00, 27.06, 25.86; ^{19}F NMR (282 MHz, $CDCl_3$) δ: -106.0; HRMS (APCI) found $[M]^+$ 359.1168, $C_{19}H_{18}FNO_5$ requires 359.1164.

3-(1,3-dioxan-2-yl)-1-(thiophen-2-yl)propyl 4-cyano-2-fluorobenzoate (S9)

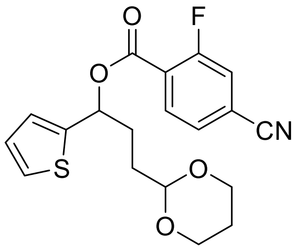

1st step. 3-(1,3-dioxan-2-yl)-1-(thiophen-2-yl)propan-1-ol: prepared according to general procedure D using thiophene-2-carbaldehyde (0.187 mL, 2.0 mmol) and (1,3-Dioxan-2-ylethyl)magnesium bromide solution (0.5 M in THF, 4.4 mL, 2.2 mmol, 1.1 equiv.). The crude alcohol was used in the next step without additional purification.

2nd step. Prepared according to general procedure B using crude 3-(1,3-dioxan-2-yl)-1-(thiophen-2-yl)propan-1-ol and 4-cyano-2-fluorobenzoic acid (330 mg, 2.0 mmol). The crude product was purified by flash column chromatography (80/20 to 60/40 Hexane/Et_2O) to provide the title compound as viscous oil (481 mg, 63% overall yield). ^{1}H NMR (300 MHz, $CDCl_3$) δ: 8.02 (app t, *J* = 7.5 Hz, 1H), 7.49 (d, *J* = 8.1 Hz, 1H), 7.43 (d, *J* = 9.7 Hz, 1H), 7.28 (d, *J* = 5.0 Hz, 1H), 7.14 (d, *J* = 3.2 Hz, 1H), 6.97 (dd, *J* = 5.0, 3.2 Hz, 1H), 6.32 (app t, *J* = 7.0 Hz, 1H), 4.57 (t, *J* = 4.9 Hz, 1H), 4.09 (dd, *J* = 10.9, 4.8 Hz, 2H), 3.74 (app t, *J* = 11.2 Hz, 2H), 2.32 – 1.99 (m, 3H), 1.73 (m, 2H), 1.33 (d, *J* = 13.5 Hz, 1H); ^{13}C NMR (125 MHz, $CDCl_3$) δ: 162.13 (d, *J* = 3.7 Hz), 161.40 (d, *J* = 264.3 Hz), 142.33, 133.31, 127.79 (d, *J* = 4.5 Hz), 126.80, 126.67, 125.86, 123.46 (d, *J* = 10.3 Hz), 121.01 (d, *J* = 26.0 Hz), 117.67 (d, *J* = 9.7 Hz), 116.79 (d, *J* = 2.6 Hz), 101.44, 73.22, 67.02, 31.27, 30.86, 25.85; ^{19}F NMR (470 MHz, $CDCl_3$) δ: -105.9; HRMS (APCI) found $[M]^+$ 375.0936, $C_{19}H_{18}FNO_4S$ requires 375.0935.

1-(2-chloropyridin-3-yl)-3-(1,3-dioxan-2-yl)propyl 4-cyano-2-fluorobenzoate (S10)

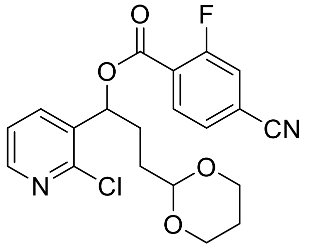

1st step. 1-(2-chloropyridin-3-yl)-3-(1,3-dioxan-2-yl)propan-1-ol: prepared according to general procedure D using 2-Chloro-3-pyridinecarboxaldehyde (283 mg, 2.0 mmol) and (1,3-Dioxan-2-ylethyl)magnesium bromide solution (0.5 M in THF, 4.4 mL, 2.2 mmol, 1.1 equiv.). The crude alcohol was used in the next step without additional purification.

2nd step. Prepared according to general procedure B using crude 1-(2-chloropyridin-3-yl)-3-(1,3-dioxan-2-yl)propan-1-ol and 4-cyano-2-fluorobenzoic acid (330 mg, 2.0 mmol). The crude product was purified by flash column chromatography (70/30 to 50/50 Hexane/Et_2O) to provide the title compound as viscous oil (510 mg, 63% overall yield). ^{1}H NMR (300 MHz, $CDCl_3$) δ: 8.34 (app d, *J* = 4.8 Hz, 1H), 8.06 (t, *J* = 7.5 Hz, 1H), 7.79 (d, *J* = 7.6 Hz, 1H), 7.53 (d, *J* = 8.1 Hz, 1H), 7.47 (d, *J* = 9.7 Hz, 1H), 7.26 (dd, *J* = 7.6, 4.8 Hz, 1H), 6.39 (app t, *J* = 6.0 Hz, 1H), 4.58 (t, *J* = 4.8 Hz, 1H), 4.07 (dd, *J* = 11.4, 4.0 Hz, 2H), 3.73 (app t, *J* = 11.0 Hz, 2H), 2.20 – 1.98 (m, 3H), 1.75 (app q, *J* = 8.2, 7.8 Hz, 2H), 1.32 (d, *J* = 13.5 Hz, 1H; ^{13}C NMR (75 MHz, $CDCl_3$) δ: 162.09 (d, *J* = 3.9 Hz), 161.35 (d, *J* = 264.6 Hz), 149.25, 149.19, 136.21, 134.66, 133.50, 127.98 (d, *J* = 4.4 Hz), 122.97, 122.89 (d, *J* = 9.9 Hz), 121.11 (d, *J* = 26.2 Hz), 118.07 (d, *J* = 9.7 Hz), 116.63 (d, *J* = 2.5 Hz), 101.33, 74.01, 67.00, 30.72, 29.38, 25.82; ^{19}F NMR (282 MHz, $CDCl_3$) δ: -105.9; HRMS (APCI) found $[M+1]^+$ 405.1004, $C_{20}H_{19}ClFN_2O_4$ requires 405.1012.

tert-butyl3-(1-((4-cyano-2-fluorobenzoyl)oxy)-3-(1,3-dioxan-2-yl)propyl)-1H-indole-1-carboxylate (S11)

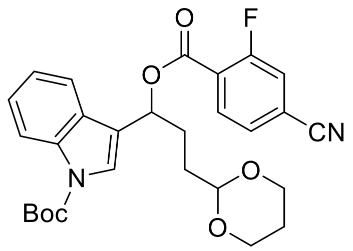

1st step. *tert*-butyl 3-(3-(1,3-dioxan-2-yl)-1-hydroxypropyl)-1H-indole-1-carboxylate: prepared according to general procedure D using N-Boc-indole-3-carboxaldehyde (491 mg, 2.0 mmol) and (1,3-Dioxan-2-ylethyl)magnesium bromide solution (0.5 M in THF, 4.0 mL, 2.0 mmol, 1.0 equiv.). The crude alcohol was purified by flash column chromatography (60/40 to 40/60 Hexane/Et_2O) to provide the title compound as viscous oil (535 mg, 74% overall yield). ^{1}H NMR (300 MHz, $CDCl_3$) δ: 8.14 (d, *J* = 8.1 Hz, 1H), 7.64 (d, *J* = 7.8 Hz, 1H), 7.54 (s, 1H), 7.30 (app t, *J* = 7.9 Hz, 1H), 7.22 (app t, *J* = 7.2 Hz, 1H), 5.00 (dt, *J* = 8.0, 3.8 Hz, 2H), 4.61 (t, *J* = 4.7 Hz, 1H), 4.12 (dd, *J* = 11.6, 4.4 Hz, 2H), 3.77 (t, *J* = 11.8 Hz, 2H), 2.76 (d, *J* = 3.8 Hz, 1H), 2.20 – 1.95 (m, 3H), 1.87 – 1.77 (m, 2H), 1.66 (s, 9H), 1.34 (d, *J* = 13.5 Hz, 1H).

2nd step. Prepared according to general procedure B using *tert*-butyl 3-(3-(1,3-dioxan-2-yl)-1-hydroxypropyl)-1H-indole-1-carboxylate (535mg, 1.48 mmol) and 4-cyano-2-fluorobenzoic acid (244 mg, 1.48 mmol). The crude product was purified by flash column chromatography (70/30 to 50/50 Hexane/Et_2O) to provide the title compound as resin-like solid (384 mg, 51% overall yield). ^{1}H NMR (300 MHz, $CDCl_3$) δ: 8.14 (d, *J* = 8.0 Hz, 1H), 8.02 (t, *J* = 7.5 Hz, 1H), 7.73 (d, *J* = 7.7 Hz, 1H), 7.66 (s, 1H), 7.48 (d, *J* = 8.7 Hz, 1H), 7.44 (d, *J* = 9.8 Hz, 1H), 7.32 (app t, *J* = 7.5 Hz, 1H), 7.24 (app t, *J* = 7.3 Hz, 1H), 6.34 (t, *J* = 7.0 Hz, 1H), 4.57 (t, *J* = 5.0 Hz, 1H), 4.08 (dd, *J* = 11.4, 4.4 Hz, 2H), 3.72 (app td, *J* = 12.4, 1.9 Hz, 2H), 2.42 – 2.16 (m, 2H), 2.16 – 1.95 (m, 1H), 1.84 – 1.60 (m, 2H), 1.67 (s, 9H), 1.32 (d, *J* = 13.5 Hz, 1H); ^{13}C NMR (75 MHz, $CDCl_3$) δ: 162.41 (d, *J* = 3.9 Hz), 161.35 (d, *J* = 264.0 Hz), 149.39, 136.57, 133.36, 129.38, 127.82 (d, *J* = 4.4 Hz), 124.88, 124.42, 123.57 (d, *J* = 10.0 Hz), 122.94, 121.04 (d, *J* = 26.1 Hz), 120.00, 119.02, 117.66 (d, *J* = 9.6 Hz), 116.79 (d, *J* = 2.5 Hz),

115.63, 101.57, 84.18, 72.11, 67.02, 31.41, 29.13, 28.33, 25.88; ^{19}F NMR (282 MHz, $CDCl_3$) δ: -105.8; HRMS (APCI) found $[M]^+$ 508.1998, $C_{28}H_{29}FN_2O_6$ requires 508.2004.

***tert*-butyl 4-(1-((4-cyano-2-fluorobenzoyl)oxy)-3-(1,3-dioxan-2-yl)propyl)-1H-imidazole-1-carboxylate (S12)**

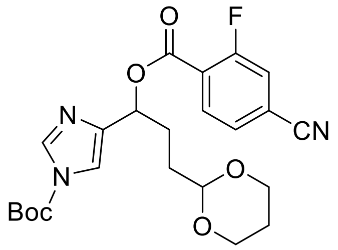

1st step. *tert*-butyl 4-(3-(1,3-dioxan-2-yl)-1-hydroxypropyl)-1H-imidazole-1-carboxylate: prepared according to general procedure D using N-Boc-imidazole-4-carbaldehyde (392 mg, 2.0 mmol) and (1,3-Dioxan-2-ylethyl)magnesium bromide solution (0.5 M in THF, 4.0 mL, 2.0 mmol, 1.0 equiv.). The crude alcohol was used in the next step without additional purification.

2nd step. Prepared according to general procedure B using crude *tert*-butyl 4-(3-(1,3-dioxan-2-yl)-1-hydroxypropyl)-1H-imidazole-1-carboxylate and 4-cyano-2-fluorobenzoic acid (330 mg, 2.0 mmol). The crude product was purified by flash column chromatography (60/40 to 40/60 Hexane/Et_2O) to provide the title compound as a resin-like solid (551 mg, 60% overall yield). ^{1}H NMR (300 MHz, $CDCl_3$) δ: 8.03 (t, J = 7.5 Hz, 1H), 8.00 (s, 1H), 7.48 (d, J = 8.1 Hz, 1H), 7.42 (d, J = 9.8 Hz, 1H), 7.38 (s, 1H), 6.06 (t, J = 6.8 Hz, 1H), 4.56 (t, J = 5.0 Hz, 1H), 4.07 (dd, J = 11.5, 5.0 Hz, 2H), 3.73 (t, J = 11.3 Hz, 2H), 2.30 – 2.12 (m, 2H), 2.12 – 1.97 (m, 1H), 1.74 – 1.63 (m, 2H), 1.60 (s, 9H), 1.31 (d, J = 13.5 Hz, 1H); ^{13}C NMR (75 MHz, $CDCl_3$) δ: 162.26 (d, J = 4.1 Hz), 161.42 (d, J = 264.0 Hz), 146.94, 141.27, 137.26, 133.35, 127.77 (d, J = 4.5 Hz), 123.58 (d, J = 10.0 Hz), 120.96 (d, J = 26.0 Hz), 117.63 (d, J = 9.8 Hz), 116.79 (d, J = 2.6 Hz), 115.64, 101.69, 86.06, 71.85, 67.00, 30.89, 28.01, 27.95, 25.88; ^{19}F NMR (282 MHz, $CDCl_3$) δ: -106.1; HRMS (APCI) found $[M+1]^+$ 460.1868, $C_{23}H_{27}FN_3O_6$ requires 460.1878.

***tert*-butyl 4-(2-((4-cyano-2-fluorobenzoyl)oxy)-2-phenylethyl)piperidine-1-carboxylate (S13)**

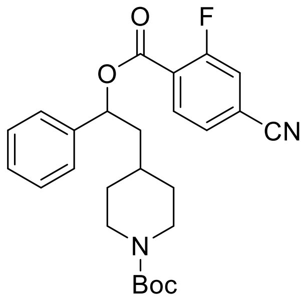

1st step. *tert*-butyl 4-(2-hydroxy-2-phenylethyl)piperidine-1-carboxylate: prepared according to general procedure D using N-Boc-4-piperidineacetaldehyde (455 mg, 2.0 mmol) and Phenylmagnesium bromide solution (3.0 M in Et_2O, 0.66 mL, 2.0 mmol, 1.0 equiv.). The crude alcohol was used in the next step without additional purification.

2nd step. Prepared according to general procedure B using crude *tert*-butyl 4-(2-hydroxy-2-phenylethyl)piperidine-1-carboxylate and 4-cyano-2-fluorobenzoic acid (330 mg, 2.0 mmol). The crude product was purified by flash column chromatography (90/10 to 70/30 Hexane/Et_2O) to provide the title compound as a viscous oil (462 mg, 51% overall yield). ^{1}H NMR (300 MHz, $CDCl_3$) δ: 8.01 (t, *J* = 7.4 Hz, 1H), 7.50 (d, *J* = 8.1 Hz, 1H), 7.40 (m, 6H), 6.10 (dd, *J* = 8.1, 5.8 Hz, 1H), 4.06 (d, *J* = 12.6 Hz, 2H), 2.64 (app q, *J* = 10.5 Hz, 2H), 2.08 (m, 1H), 1.84 – 1.65 (m, 3H), 1.55 – 1.37 (m, 1H), 1.44 (s, 9H), 1.19 (m, 2H); ^{13}C NMR (75 MHz, $CDCl_3$) δ: 162.45 (d, *J* = 3.6 Hz), 161.39 (d, *J* = 264.3 Hz), 155.57, 140.04, 133.35, 128.89, 128.58, 127.89 (d, *J* = 4.4 Hz), 126.71, 123.57 (d, *J* = 10.6 Hz), 121.09 (d, *J* = 26.0 Hz), 117.77 (d, *J* = 9.8 Hz), 116.76 (d, *J* = 2.8 Hz), 80.48, 76.13, 43.92, 43.35, 32.80, 32.46, 31.99, 28.61; ^{19}F NMR (282 MHz, $CDCl_3$) δ: -105.9; HRMS (APCI) found $[M]^+$ 452.2099, $C_{26}H_{29}FN_2O_4$ requires 452.2106.

***tert*-butyl 4-(2-(5-chlorothiophen-2-yl)-2-((4-cyano-2-fluorobenzoyl)oxy)ethyl) piperidine-1-carboxylate (S14)**

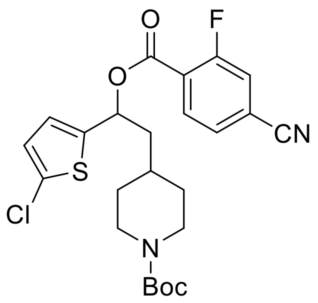

1st step. *tert*-butyl 4-(2-(5-chlorothiophen-2-yl)-2-hydroxyethyl)piperidine-1-carboxylate: prepared according to general procedure D using N-Boc-4-piperidineacetaldehyde (455 mg, 2.0 mmol) and 5-Chloro-2-thienylmagnesium bromide solution (0.5 M in THF, 4.0 mL, 2.0 mmol, 1.0 equiv.). The crude alcohol was used in the next step without additional purification.

2nd step. Prepared according to general procedure B using crude *tert*-butyl 4-(2-(5-chlorothiophen-2-yl)-2-hydroxyethyl)piperidine-1-carboxylate and 4-cyano-2-fluorobenzoic acid (330 mg, 2.0 mmol). The crude product was purified by flash column chromatography (90/10 to 70/30 Hexane/Et_2O) to provide the title compound as a viscous oil (542 mg, 55% overall yield). ^{1}H NMR (300 MHz, $CDCl_3$) δ: 8.00 (t, *J* = 7.5 Hz, 1H), 7.51 (d, *J* = 8.1 Hz, 1H), 7.45 (d, *J* = 9.7 Hz, 1H), 6.93 (d, *J* = 3.7 Hz, 1H), 6.79 (d, *J* = 3.7 Hz, 1H), 6.25 (t, *J* = 7.2 Hz, 1H), 4.07 (d, *J* = 11.6 Hz, 2H), 2.65 (t, *J* = 12.6 Hz, 2H), 2.16 – 2.02 (m, 1H), 1.94 – 1.81 (m, 1H), 1.71 (d, *J* = 12.7 Hz, 2H), 1.58 – 1.49 (m, 1H), 1.44 (s, 9H), 1.31 – 1.09 (m, 2H); ^{13}C NMR (75 MHz, $CDCl_3$) δ: 162.19 (d, *J* = 3.8 Hz), 161.40 (d, *J* = 264.3 Hz), 154.90, 141.06, 133.27, 131.02, 127.90 (d, *J* = 4.5 Hz), 126.38, 125.89, 123.11 (d, *J* = 10.2 Hz), 121.09 (d, *J* = 26.0 Hz), 117.98 (d, *J* = 9.7 Hz), 116.65 (d, *J* = 2.5 Hz), 80.07, 71.07, 44.45, 42.23, 33.23, 32.18, 31.51, 28.58; ^{19}F NMR (282 MHz, $CDCl_3$) δ: -105.8; HRMS (APCI) found $[M]^+$ 492.1279, $C_{24}H_{26}ClFN_2O_4S$ requires 492.1280.

1-phenylhex-5-en-1-yl 4-cyano-2-fluorobenzoate (S15)

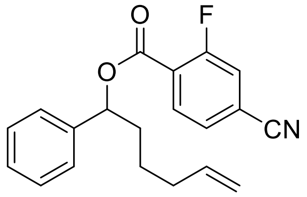

1st step. 1-phenylhex-5-en-1-ol: prepared according to general procedure D using benzaldehyde (0.406 mL, 4.0 mmol) and pent-4-en-1-ylmagnesium bromide solution (0.7 M in THF, 7.15 mL, 5.0 mmol, 1.25 equiv. Prepared according to literature procedure: *J. Am. Chem. Soc.* **2016**, *138*, 15798–15800). The crude alcohol was purified by flash column chromatography (80/20 to 60/40 Hexane/Et_2O) to provide the title compound as a colorless oil (367 mg, 52% overall yield). ^{1}H NMR (300 MHz, $CDCl_3$) δ: 7.39 – 7.31 (m, 4H), 7.31 – 7.23 (m, 1H), 5.87 – 5.70 (m, 1H), 5.05 – 4.90 (m, 2H), 4.68 (dd, *J* = 7.4, 5.8 Hz, 1H), 2.08 (q, *J* = 7.3 Hz, 2H), 1.90 – 1.65 (m, 2H), 1.62 – 1.47 (m, 1H), 1.45 – 1.30 (m, 1H).

2nd step. Prepared according to general procedure B using 1-phenylhex-5-en-1-ol (144 mg, 0.82 mmol), 4-cyano-2-fluorobenzoic acid (165 mg, 1.0 mmol, 1.22 equiv), DCC (206 mg, 1.0 mmol, 1.22 equiv), DMAP (10 mg, 0.08 mmol, 10 mol%) and 5 mL of DCM. The crude product was purified by flash column chromatography (90/10 to 80/20 Hexane/Et_2O) to provide the title compound as a colorless oil (264 mg, quantitative). ^{1}H NMR (300 MHz, $CDCl_3$) δ: 8.03 (t, *J* = 7.5 Hz, 1H), 7.50 (dd, *J* = 8.1, 1.1 Hz, 1H), 7.45 (dd, *J* = 9.7, 1.1 Hz, 1H), 7.43 – 7.30 (m, 5H), 6.01 (t, *J* = 6.8 Hz, 1H), 5.85 – 5.68 (m, 1H), 5.06 – 4.91 (m, 2H), 2.16 – 2.00 (m, 3H), 2.00 – 1.84 (m, 1H), 1.61 – 1.33 (m, 2H); ^{13}C NMR (75 MHz, $CDCl_3$) δ: 162.44 (d, *J* = 4.0 Hz), 161.38 (d, *J* = 263.7 Hz), 140.01, 138.19, 133.35 (d, *J* = 1.4 Hz), 128.75, 128.39, 127.83 (d, *J* = 4.5 Hz), 126.70, 123.72 (d, *J* = 10.7 Hz), 121.05 (d, *J* = 26.1 Hz), 117.64 (d, *J* = 9.7 Hz), 116.80 (d, *J* = 2.1 Hz), 115.21, 78.39, 35.91, 33.41, 24.81; ^{19}F NMR (282 MHz, $CDCl_3$) δ: -105.9; HRMS (APCI) found $[M]^+$ 323.1310, $C_{20}H_{18}FNO_2$ requires 323.1316.

1-phenylcyclohexyl 4-cyano-2-fluorobenzoate (S16)

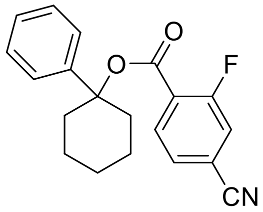

Prepared according to general procedure C using 1-phenylcyclohexan-1-ol (881 mg, 5.0 mmol) and 4-cyano-2-fluorobenzoic acid (990 mg, 6.0 mmol, 1.2 equiv.). The crude product was purified by flash column chromatography (95/5 to 80/20 Hexane/Et_2O) to provide the title compound as a white solid (825 mg, 51% overall yield). ^{1}H NMR (300 MHz, $CDCl_3$) δ: 8.01 (t, *J* = 7.4 Hz, 1H), 7.56 – 7.42 (m, 4H), 7.38 (t, *J* = 7.4 Hz, 2H), 7.34 – 7.26 (m, 1H), 2.70 (d, *J* = 12.7 Hz, 2H), 2.00 – 1.70 (m, 7H), 1.49 – 1.27 (m, 1H). ^{13}C NMR (75 MHz, $CDCl_3$) δ: 161.47 (d, *J* = 3.7 Hz), 161.25 (d, *J* = 262.7 Hz), 144.77, 133.31 (d, *J* = 1.7 Hz), 128.58, 127.84 (d, *J* = 4.4 Hz), 127.55, 124.79, 124.67 (d, J = 10.3 Hz), 121.01 (d, *J* = 26.5 Hz), 117.28 (d, *J* = 9.6 Hz), 116.90 (d, *J* = 2.5 Hz), 85.77, 36.33, 25.34, 22.15; ^{19}F NMR (282 MHz, $CDCl_3$) δ: -106.0; HRMS (APCI) found $[M]^+$ 323.1319, $C_{20}H_{18}FNO_2$ requires 323.1316.

1-phenylcyclopentyl 4-cyano-2-fluorobenzoate (S17)

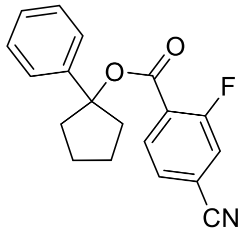

1st step. 1-phenylcyclopentan-1-ol: prepared according to general procedure E using cyclopentanone (0.880 mL, 10.0 mmol) and Phenylmagnesium bromide solution (3 M in THF, 4.0 mL, 12.0 mmol, 1.2 equiv.). The crude alcohol was purified by flash column chromatography (90/10 to 70/30 Hexane/Et_2O) to provide the title compound as a white solid (730 mg, 45% overall yield). ^{1}H NMR (300 MHz, $CDCl_3$) δ: 7.55 – 7.46 (m, 2H), 7.35 (t, *J* = 7.5 Hz, 2H), 7.26 (t, *J* = 3.6 Hz, 2H), 2.08 – 1.78 (m, 8H).

2nd step. Prepared according to general procedure C using 1-phenylcyclopentan-1-ol (487 mg, 3.0 mmol) and 4-cyano-2-fluorobenzoic acid (594 mg, 3.6 mmol, 1.2 equiv.). The crude product was purified by flash column chromatography (95/5 to 80/20

Hexane/Et_2O) to provide the title compound as a white solid (380 mg, 41% overall yield). ^1H NMR (300 MHz, $CDCl_3$) δ: 7.95 (t, *J* = 7.4 Hz, 1H), 7.55 – 7.41 (m, 4H), 7.41 – 7.24 (m, 3H), 2.77 – 2.57 (m, 2H), 2.36 – 2.14 (m, 2H), 2.09 – 1.80 (m, 4H). ^{13}C NMR (75 MHz, $CDCl_3$) δ: 161.90 (d, *J* = 3.6 Hz), 161.22 (d, *J* = 262.9 Hz), 142.46, 133.20, 128.42, 127.77 (d, *J* = 4.4 Hz), 127.67, 125.68, 124.67 (d, *J* = 10.9 Hz), 120.94 (d, *J* = 26.4 Hz), 117.24 (d, *J* = 9.6 Hz), 116.87 (d, *J* = 2.4 Hz), 94.56, 38.50, 23.30; ^{19}F NMR (282 MHz, $CDCl_3$) δ: -106.5; HRMS (APCI) found $[M]^+$ 309.1150, $C_{19}H_{16}FNO_2$ requires 309.1160.

1-phenylcyclobutyl 4-cyano-2-fluorobenzoate (S18)

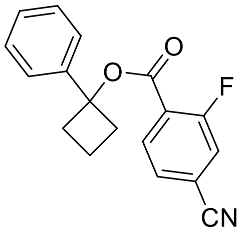

1st step. 1-phenylcyclobutan-1-ol: prepared according to general procedure E using cyclobutanone (0.747 mL, 10.0 mmol) and Phenylmagnesium bromide solution (3 M in THF, 4.0 mL, 12.0 mmol, 1.2 equiv.). The crude alcohol was used in the next step without additional purification.

2nd step. Prepared according to general procedure C using 1-phenylcyclobutan-1-ol (741 mg, 5.0 mmol) and 4-cyano-2-fluorobenzoic acid (990 mg, 6.0 mmol, 1.2 equiv.). The crude product was purified by flash column chromatography (95/5 to 80/20 Hexane/Et_2O) to provide the title compound as a white solid (1.122 g, 76% overall yield). ^1H NMR (300 MHz, $CDCl_3$) δ: 7.96 (t, *J* = 7.5 Hz, 1H), 7.61 – 7.52 (m, 2H), 7.47 (dd, *J* = 7.9, 1.2 Hz, 1H), 7.45 – 7.34 (m, 3H), 7.33 – 7.26 (m, 1H), 2.90 – 2.68 (m, 4H), 2.14 – 1.98 (m, 1H), 1.90 – 1.73 (m, 1H). ^{13}C NMR (75 MHz, $CDCl_3$) δ: 161.40 (d, *J* = 3.8 Hz), 161.35 (d, *J* = 263.3 Hz), 141.75, 133.25 (d, *J* = 1.4 Hz), 128.55, 127.93, 127.76 (d, *J* = 4.5 Hz), 125.99, 124.11 (d, *J* = 10.6 Hz), 120.97 (d, *J* = 26.2 Hz), 117.43 (d, *J* = 9.7 Hz), 116.82 (d, *J* = 2.5 Hz), 84.57, 34.95, 14.32; ^{19}F NMR (282 MHz, $CDCl_3$) δ: -106.2; HRMS (APCI) found $[M]^+$ 295.1011, $C_{18}H_{14}FNO_2$ requires 295.1003.

1-phenylcycloheptyl 4-cyano-2-fluorobenzoate (S19)

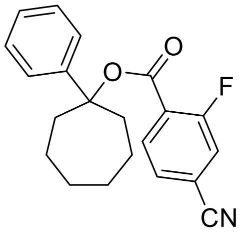

1st step. 1-phenylcycloheptan-1-ol: prepared according to general procedure E using cycloheptanone (1.18 mL, 10.0 mmol) and Phenylmagnesium bromide solution (3 M in THF, 4.0 mL, 12.0 mmol, 1.2 equiv.). The crude alcohol was purified by flash column chromatography (90/10 to 70/30 Hexane/Et_2O) to provide the title compound as a white solid (818 mg, 43% overall yield). ^{1}H NMR (300 MHz, $CDCl_3$) δ: 7.51 (d, *J* = 7.5 Hz, 2H), 7.34 (t, *J* = 7.6 Hz, 2H), 7.28 – 7.19 (m, 1H), 2.07 (dd, *J* = 14.4, 10.3 Hz, 2H), 1.98 – 1.51 (m, 10H).

2nd step. Prepared according to general procedure C using 1-phenylcycloheptan-1-ol (571 mg, 3.0 mmol) and 4-cyano-2-fluorobenzoic acid (594 mg, 3.6 mmol, 1.2 equiv.). The crude product was purified by flash column chromatography (95/5 to 80/20 Hexane/Et_2O) to provide the title compound as a white solid (506 mg, 50% overall yield). ^{1}H NMR (300 MHz, $CDCl_3$) δ: δ 7.98 (t, *J* = 7.5 Hz, 1H), 7.53 – 7.42 (m, 2H), 7.41 – 7.29 (m, 4H), 7.28 – 7.21 (m, 1H), 2.54 – 2.29 (m, 4H), 1.91 – 1.78 (m, 2H), 1.78 – 1.58 (m, 6H). ^{13}C NMR (75 MHz, $CDCl_3$) δ: 161.64 (d, *J* = 3.9 Hz), 161.27 (d, *J* = 262.7 Hz), 146.09, 133.29 (d, *J* = 1.6 Hz), 128.56, 127.84 (d, *J* = 4.5 Hz), 127.26, 124.75 (d, *J* = 11.1 Hz), 124.38, 121.01 (d, *J* = 26.5 Hz), 117.26 (d, *J* = 9.6 Hz), 116.89 (d, *J* = 2.5 Hz), 90.00, 40.94, 29.54, 23.40. ^{19}F NMR (282 MHz, $CDCl_3$) δ: -106.1; HRMS (APCI) found $[M]^+$ 337.1469, $C_{21}H_{20}FNO_2$ requires 337.1473.

2-phenylbicyclo[2.2.1]heptan-2-yl 4-cyano-2-fluorobenzoate (S20)

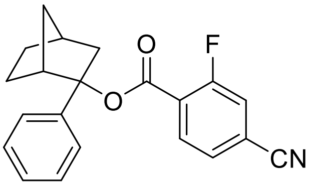

1st step. 2-phenylbicyclo[2.2.1]heptan-2-ol: prepared according to general procedure E using bicyclo[2.2.1]heptan-2-one (1.102 g, 10.0 mmol) and Phenylmagnesium bromide

solution (3 M in THF, 4.0 mL, 12.0 mmol, 1.2 equiv.). The crude alcohol was used in the next step without additional purification.

2nd step. Prepared according to general procedure C using 2-phenylbicyclo[2.2.1]heptan-2-ol (942 mg, 5.0 mmol) and 4-cyano-2-fluorobenzoic acid (990 mg, 6.0 mmol, 1.2 equiv.). The crude product was purified by flash column chromatography (95/5 to 80/20 Hexane/Et_2O) to provide the title compound as a white solid (1.358 g, 81% overall yield). ^1H NMR (300 MHz, $CDCl_3$) δ: 7.93 (t, J = 7.6 Hz, 1H), 7.53 – 7.40 (m, 4H), 7.37 – 7.28 (m, 2H), 7.27 – 7.19 (m, 1H), 3.27 (app s, 1H), 2.48 (ddd, J = 13.8, 4.6, 2.3 Hz, 1H), 2.39 (app s, 1H), 1.95 (dd, J = 13.8, 3.6 Hz, 1H), 1.92 – 1.81 (m, 1H), 1.65 (m, 3H), 1.50 – 1.37 (m, 2H). ^{13}C NMR (75 MHz, $CDCl_3$) δ: 162.10 (d, J = 3.8 Hz), 161.31 (d, J = 262.9 Hz), 144.87, 133.26 (d, J = 1.5 Hz), 128.45, 127.80 (d, J = 4.4 Hz), 127.31, 125.98, 124.33 (d, J = 10.9 Hz), 120.98 (d, J = 26.4 Hz), 117.33 (d, J = 9.7 Hz), 116.86 (d, J = 2.5 Hz), 91.33, 46.11, 45.51, 37.74, 36.75, 28.96, 23.26. ^{19}F NMR (282 MHz, $CDCl_3$) δ: -106.4; HRMS (APCI) found $[M]^+$ 335.1303, $C_{21}H_{18}FNO_2$ requires 335.1316.

4-phenyltetrahydro-2H-pyran-4-yl 4-cyano-2-fluorobenzoate (S21)

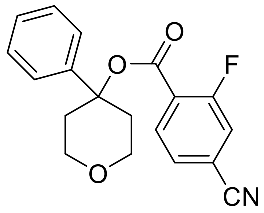

1st step. 4-phenyltetrahydro-2H-pyran-4-ol: prepared according to general procedure E using tetrahydro-4H-pyran-4-one (0.923 mL, 10.0 mmol) and Phenylmagnesium bromide solution (3 M in THF, 4.0 mL, 12.0 mmol, 1.2 equiv.). The crude alcohol was used in the next step without additional purification.

2nd step. Prepared according to general procedure C using 4-phenyltetrahydro-2H-pyran-4-ol (891 mg, 5.0 mmol) and 4-cyano-2-fluorobenzoic acid (990 mg, 6.0 mmol, 1.2 equiv.). The crude product was purified by flash column chromatography (60/40 to 50/50 Hexane/Et_2O) to provide the title compound as a white solid (943 mg, 58% overall yield). ^1H NMR (300 MHz, $CDCl_3$) δ: 7.97 (t, J = 7.6 Hz, 1H), 7.56 – 7.26 (m, 7H), 4.04 – 3.84 (m, 4H), 2.62 (d, J = 12.6 Hz, 2H), 2.35 – 2.20 (m,

2H). ^{13}C NMR (75 MHz, $CDCl_3$) δ: 161.49 (d, *J* = 4.0 Hz), 161.15 (d, *J* = 262.6 Hz), 143.10, 133.24 (d, *J* = 1.5 Hz), 128.74, 128.00, 127.89 (d, *J* = 4.4 Hz), 124.73, 124.08 (d, *J* = 11.2 Hz), 121.02 (d, *J* = 26.6 Hz), 117.56 (d, *J* = 9.7 Hz), 116.68 (d, *J* = 2.5 Hz). ^{19}F NMR (282 MHz, $CDCl_3$) δ: -105.8; HRMS (APCI) found $[M]^+$ 325.1105, $C_{19}H_{16}FNO_3$ requires 325.1109.

***tert*-butyl 4-((4-cyano-2-fluorobenzoyl)oxy)-4-phenylpiperidine-1-carboxylate (S22)**

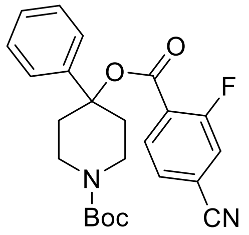

1st step. *tert*-butyl 4-hydroxy-4-phenylpiperidine-1-carboxylate: prepared according to general procedure E using *tert*-butyl 4-oxopiperidine-1-carboxylate (1.993 g, 10.0 mmol) and Phenylmagnesium bromide solution (3 M in THF, 3.33 mL, 10.0 mmol, 1.0 equiv.). The crude alcohol was purified by flash column chromatography (70/30 to 50/50 Hexane/Et_2O) to provide the title compound as a white solid (693 mg, 25% overall yield). ^{1}H NMR (300 MHz, $CDCl_3$) δ: 7.48 (app d, *J* = 7.2 Hz, 2H), 7.36 (app t, *J* = 7.4 Hz, 2H), 7.27 (app t, *J* = 7.1 Hz, 1H), 4.01 (bs, 2H), 3.24 (t, *J* = 11.7 Hz, 2H), 1.98 (t, *J* = 10.7 Hz, 2H), 1.79 – 1.67 (m, 2H), 1.48 (d, *J* = 3.3 Hz, 9H).

2nd step. Prepared according to general procedure C using *tert*-butyl 4-hydroxy-4-phenylpiperidine-1-carboxylate (702 mg, 2.53 mmol) and 4-cyano-2-fluorobenzoic acid (502 mg, 3.04 mmol, 1.2 equiv.). The crude product was purified by flash column chromatography (70/30 to 60/40 Hexane/Et_2O) to provide the title compound as a white solid (698 mg, 65% overall yield). ^{1}H NMR (300 MHz, $CDCl_3$) δ: 7.94 (dd, *J* = 8.2, 7.0 Hz, 1H), 7.52 – 7.25 (m, 7H), 4.21 – 3.98 (m, 2H), 3.20 (t, *J* = 12.5 Hz, 2H), 2.65 (app d, *J* = 12.5 Hz, 2H), 2.06 (td, *J* = 13.7, 4.8 Hz, 2H), 1.48 (s, 9H). ^{13}C NMR (75 MHz, $CDCl_3$) δ: 161.50 (d, *J* = 4.0 Hz), 161.20 (d, *J* = 262.7 Hz), 154.89, 143.07, 133.32 (d, *J* = 1.4 Hz), 128.80, 128.07, 127.94 (d, *J* = 4.3 Hz), 124.71, 124.03 (d, *J* = 11.3 Hz), 121.07 (d, *J* = 26.6 Hz), 117.67 (d, *J* = 9.7 Hz), 116.69 (d, *J* = 2.5 Hz), 83.53, 79.93, 39.75, 35.50, 28.55. ^{19}F NMR (282 MHz, $CDCl_3$) δ: -105.8; HRMS (APCI) found $[M+1]^+$ 425.1881, $C_{24}H_{26}FN_2O_4$ requires 425.1871.

tert-butyl 3-((4-cyano-2-fluorobenzoyl)oxy)-3-phenylazetidine-1-carboxylate (S23)

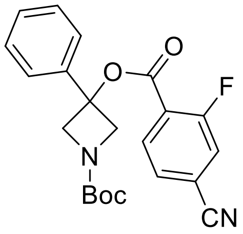

1st step. _tert_-butyl 3-hydroxy-3-phenylazetidine-1-carboxylate: prepared according to general procedure E using _tert_-butyl 3-oxoazetidine-1-carboxylate (1.712 g, 10.0 mmol) and Phenylmagnesium bromide solution (3 M in THF, 3.33 mL, 10.0 mmol, 1.0 equiv.). The crude alcohol was used in the next step without additional purification.

2nd step. Prepared according to general procedure C using _tert_-butyl 3-hydroxy-3-phenylazetidine-1-carboxylate (1.247 g, 5.0 mmol) and 4-cyano-2-fluorobenzoic acid (990 mg, 6.0 mmol, 1.2 equiv.). The crude product was purified by flash column chromatography (70/30 to 60/40 Hexane/Et_2O) to provide the title compound as a white solid (1.546 g, 78% overall yield). ^{1}H NMR (300 MHz, $CDCl_3$) δ: 8.04 (app t, _J_ = 7.5 Hz, 1H), 7.55 – 7.29 (m, 7H), 4.50 (s, 4H), 1.46 (s, 9H). ^{13}C NMR (75 MHz, $CDCl_3$) δ: 161.52 (d, _J_ = 264.7 Hz), 161.43 (d, _J_ = 4.1 Hz), 156.32, 139.06, 133.45, 128.92, 128.65, 127.93 (d, _J_ = 4.5 Hz), 125.37, 122.78 (d, _J_ = 10.3 Hz), 121.18 (d, _J_ = 26.1 Hz), 118.25 (d, _J_ = 9.7 Hz), 116.61 (d, _J_ = 2.4 Hz), 80.43, 78.17, 61.32, 28.46. ^{19}F NMR (282 MHz, $CDCl_3$) δ: -105.3; HRMS (APCI) found $[M+1]^+$ 397.1543, $C_{22}H_{22}FN_2O_4$ requires 397.1558.

1-(2-(1,3-dioxan-2-yl)ethyl)-1,2,3,4-tetrahydronaphthalen-1-yl-4-cyano-2-fluorobenzoate (S24)

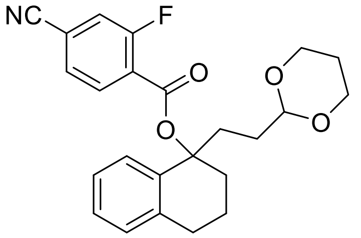

1st step. 1-(2-(1,3-dioxan-2-yl)ethyl)-1,2,3,4-tetrahydronaphthalen-1-ol: prepared according to general procedure E using α-tetralone (0.665 mL, 5.0 mmol) and (1,3-Dioxan-2-ylethyl)magnesium bromide solution (0.5 M in THF, 10 mL, 5.0 mmol, 1.0 equiv.). The crude alcohol was purified by flash column chromatography (50/50 to

30/70 Hexane/Et_2O) to provide the title compound as a viscous oil (328 mg, 25% overall yield). ^1H NMR (300 MHz, $CDCl_3$) δ: 7.54 (d, *J* = 6.4 Hz, 1H), 7.23 – 7.10 (m, 2H), 7.05 (d, *J* = 8.0 Hz, 1H), 4.53 (t, *J* = 5.0 Hz, 1H), 4.16 – 4.02 (m, 2H), 3.82 – 3.68 (m, 2H), 2.87 – 2.64 (m, 2H), 2.15 – 1.63 (m, 8H), 1.39 – 1.18 (m, 2H).

2nd step. Prepared according to general procedure C using 1-(2-(1,3-dioxan-2-yl)ethyl)-1,2,3,4-tetrahydronaphthalen-1-ol (328 mg, 1.25 mmol) and 4-cyano-2-fluorobenzoic acid (248 mg, 1.5 mmol, 1.2 equiv.). The crude product was purified by flash column chromatography (80/20/3 to 70/30/3 Hexane/Et_2O/Et_3N) to provide the title compound as a yellow viscous oil (159 mg, 31% overall yield). *Note: Product unstable over time and/or acidic media; used immediately for the photochemical coupling.* ^1H NMR (300 MHz, $CDCl_3$) δ. 7.94 (t, *J* = 7.5 Hz, 1H), 7.46 (d, *J* = 8.1 Hz, 1H), 7.41 (d, *J* = 9.6 Hz, 1H), 7.38 – 7.31 (m, 1H), 7.18 – 7.06 (m, 3H), 4.54 (t, *J* = 5.0 Hz, 1H), 4.14 – 4.01 (m, 2H), 3.81 – 3.65 (m, 2H), 3.02 – 2.86 (m, 1H), 2.83 – 2.60 (m, 2H), 2.33 – 1.97 (m, 5H), 1.97 – 1.65 (m, 3H), 1.33 (d, *J* = 13.5 Hz, 1H). ^{13}C NMR (75 MHz, $CDCl_3$) δ: 161.45 (d, *J* = 3.6 Hz), 161.20 (d, *J* = 262.9 Hz), 138.68, 137.12, 133.19, 129.04, 127.76 (d, *J* = 4.4 Hz), 127.51, 125.65, 124.84 (d, *J* = 10.6 Hz), 122.82, 120.94 (d, *J* = 26.4 Hz), 117.17 (d, *J* = 9.7 Hz), 116.85 (d, *J* = 2.5 Hz), 102.21, 85.83, 67.02, 31.33, 29.93, 29.50, 26.93, 25.86, 20.70; ^{19}F NMR (282 MHz, $CDCl_3$) δ: -106.2.

2-(5-chlorothiophen-2-yl)-4-phenylbutan-2-yl 4-cyano-2-fluorobenzoate (S25)

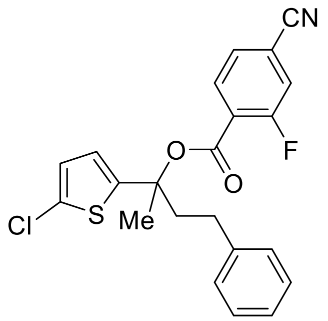

1st step. 2-(5-chlorothiophen-2-yl)-4-phenylbutan-2-ol: prepared according to general procedure E using 4-phenyl-2-butanone (0.750 mL, 10.0 mmol) and 5-Chloro-2-thienylmagnesium bromide solution (0.5 M in THF, 10 mL, 10.0 mmol, 1.0 equiv.). The crude alcohol was used in the next step without additional purification.

2nd step. Prepared according to general procedure C using 2-(5-chlorothiophen-2-yl)-4-phenylbutan-2-ol (1.33 g, 5.0 mmol) and 4-cyano-2-fluorobenzoic acid (990 mg, 6.0 mmol, 1.2 equiv.). The crude product was purified by flash column chromatography (80/20/3 Hexane/Et_2O/Et_3N) to provide the title compound as a yellow viscous oil (1.035 g, 50% overall yield). *Note: Product unstable over time and/or acidic media; used immediately for the photochemical coupling.* ^{1}H NMR (300 MHz, $CDCl_3$) δ: 7.95 (t, *J* = 7.5 Hz, 1H), 7.50 (d, *J* = 8.2 Hz, 1H), 7.46 (d, *J* = 9.7 Hz, 1H), 7.34 – 7.11 (m, 5H), 6.88 (d, *J* = 3.8 Hz, 1H), 6.81 (d, *J* = 3.8 Hz, 1H), 2.73 – 2.60 (m, 2H), 2.60 – 2.47 (m, 2H), 2.07 (s, 3H). ^{13}C NMR (75 MHz, $CDCl_3$) δ: 161.43 (d, *J* = 3.7 Hz), 161.27 (d, *J* = 263.6 Hz), 146.20, 141.02, 133.24, 129.76, 128.65, 128.53, 127.86 (d, *J* = 4.5 Hz), 126.27, 125.92, 124.11 (d, *J* = 10.4 Hz), 123.75, 121.05 (d, *J* = 26.3 Hz), 117.63 (d, *J* = 9.5 Hz), 116.78 (d, *J* = 2.5 Hz), 85.01, 44.30, 30.51, 25.72; ^{19}F NMR (282 MHz, $CDCl_3$) δ: -105.9.

Benzyl 4-cyano-2-fluorobenzoate (S26)

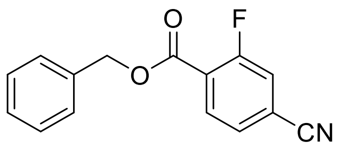

Prepared according to general procedure B using benzyl alcohol (0.312 mL, 3.0 mmol) and 4-cyano-2-fluorobenzoic acid (495 mg, 3.0 mmol). The crude product was purified by flash column chromatography (95/5 to 85/15 Hexane/Et_2O) to provide the title compound as a white solid (536 mg, 70% yield). ^{1}H NMR (500 MHz, $CDCl_3$) δ: 8.06 (dd, *J* = 8.1, 7.1 Hz, 1H), 7.51 (dd, *J* = 8.1, 1.3 Hz, 1H), 7.48 – 7.43 (m, 3H), 7.43 – 7.34 (m, 3H), 5.41 (s, 2H); ^{13}C NMR (125 MHz, $CDCl_3$) δ: 162.78 (d, *J* = 3.8 Hz), 161.38 (d, *J* = 264.1 Hz), 135.17, 133.30 (d, *J* = 1.4 Hz), 128.82, 128.71, 128.40, 127.83 (d, *J* = 4.5 Hz), 123.27 (d, *J* = 10.3 Hz), 121.03 (d, *J* = 26.0 Hz), 117.75 (d, *J* = 9.8 Hz), 116.73 (d, *J* = 2.6 Hz), 67.87; ^{19}F NMR (470 MHz, $CDCl_3$) δ: -106.0; HRMS (APCI) found $[M+1]^+$ 256.0762, $C_{15}H_{11}FNO_2$ requires 256.0768.

4-methoxybenzyl 4-cyano-2-fluorobenzoate (S27)

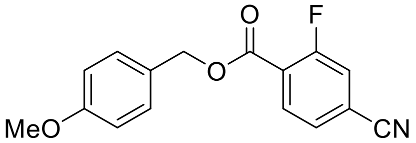

Prepared according to general procedure B using 4-methoxybenzyl alcohol (0.372 mL, 3.0 mmol) and 4-cyano-2-fluorobenzoic acid (495 mg, 3.0 mmol). The crude product was purified by flash column chromatography (95/5 to 85/15 Hexane/Et_2O) to provide the title compound as a white solid (565 mg, 66% yield). ^{1}H NMR (500 MHz, $CDCl_3$) δ: 8.03 (dd, *J* = 8.0, 7.1 Hz, 1H), 7.49 (dd, *J* = 8.0, 1.1 Hz, 1H), 7.43 (dd, *J* = 9.7, 1.1 Hz, 1H), 7.41 – 7.35 (m, 2H), 6.94 – 6.89 (m, 2H), 5.33 (s, 2H), 3.82 (s, 3H); ^{13}C NMR (125 MHz, $CDCl_3$) δ: 162.85 (d, *J* = 3.8 Hz), 161.37 (d, *J* = 264.1 Hz), 160.05, 133.27 (d, *J* = 1.4 Hz), 130.39, 127.80 (d, *J* = 4.5 Hz), 127.29, 123.46 (d, *J* = 10.3 Hz), 121.00 (d, *J* = 26.1 Hz), 117.65 (d, *J* = 9.7 Hz), 116.76 (d, *J* = 2.5 Hz), 114.21, 67.78, 55.43; ^{19}F NMR (470 MHz, $CDCl_3$) δ: -106.2; HRMS (APCI) found $[M]^+$ 285.0795, $C_{16}H_{12}FNO_3$ requires 285.0796.

3-((*tert*-butoxycarbonyl)amino)benzyl 4-cyano-2-fluorobenzoate (S28)

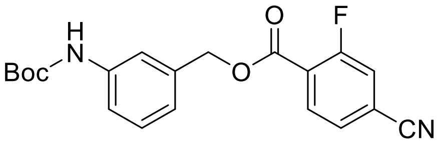

Prepared according to general procedure B using N-Boc-3-aminobenzyl alcohol (670 mg, 3.0 mmol) and 4-cyano-2-fluorobenzoic acid (495 mg, 3.0 mmol). The crude product was purified by flash column chromatography (90/10 to 70/30 Hexane/Et_2O) to provide the title compound as a white solid (711 mg, 64% yield). ^{1}H NMR (300 MHz, $CDCl_3$) δ: 8.05 (t, *J* = 7.5 Hz, 1H), 7.52 (s, 1H), 7.49 (d, *J* = 8.0, Hz, 1H), 7.44 (d, *J* = 9.7, Hz, 1H), 7.29 (app d, *J* = 4.3 Hz, 2H), 7.15 – 7.05 (m, 1H), 6.61 (s, 1H), 5.35 (s, 2H), 1.51 (s, 9H); ^{13}C NMR (75 MHz, $CDCl_3$) δ: 162.69 (d, *J* = 3.7 Hz), 161.38 (d, *J* = 264.2 Hz), 152.79, 138.93, 136.09, 133.32, 129.44, 127.81 (d, *J* = 4.5 Hz), 123.26 (d, *J* = 10.1 Hz), 122.84, 120.99 (d, *J* = 26.0 Hz), 118.71, 118.33, 117.72 (d, *J* = 9.7 Hz), 116.72 (d, *J* = 2.4 Hz) 80.83, 67.70, 28.43; ^{19}F NMR (282 MHz, $CDCl_3$) δ: -106.0; HRMS (APCI) found $[M]^+$ 370.1336, $C_{20}H_{19}FN_2O_4$ requires 370.1323.

tert-butyl 3-(((4-cyano-2-fluorobenzoyl)oxy)methyl)-1H-indole-1-carboxylate (S29)

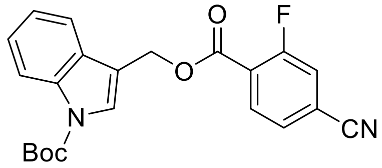

Prepared according to general procedure B using N-Boc-indole-3-carbinol (742 mg, 3.0 mmol) and 4-cyano-2-fluorobenzoic acid (495 mg, 3.0 mmol). The crude product was purified by flash column chromatography (90/10 to 70/30 Hexane/Et_2O) to provide the title compound as a white solid (805 mg, 68% yield). ^{1}H NMR (300 MHz, $CDCl_3$) δ: 8.16 (d, *J* = 8.2 Hz, 1H), 8.02 (app t, *J* = 7.5 Hz, 1H), 7.75 (s, 1H), 7.68 (d, *J* = 7.5 Hz, 1H), 7.47 (app d, *J* = 8.1 Hz, 1H), 7.42 (dd, *J* = 9.8, 1.2 Hz, 1H), 7.40 – 7.33 (m, 1H), 7.33 – 7.25 (m, 1H), 5.56 (s, 2H), 1.68 (s, 9H); ^{13}C NMR (75 MHz, $CDCl_3$) δ: 162.86 (d, J = 3.7 Hz), 161.38 (d, J = 264.3 Hz), 149.57, 135.76, 133.28, 129.28, 127.80 (d, J = 4.4 Hz), 126.33, 125.03, 123.28 (d, J = 10.3 Hz), 123.13, 121.01 (d, J = 26.0 Hz), 119.26, 117.75 (d, J = 9.7 Hz), 116.71 (d, J = 2.5 Hz), 115.57, 114.83, 84.30, 59.78, 28.30; ^{19}F NMR (282 MHz, $CDCl_3$) δ: -106.1; HRMS (APCI) found $[M]^+$ 394.1327, $C_{22}H_{19}FN_2O_4$ requires 394.1323.

4-(4-chlorophenyl)-1-(4-(4-fluorophenyl)-4-oxobutyl)piperidin-4-yl-4-cyano-2-fluorobenzoate (Haloperidol ester) (S30)

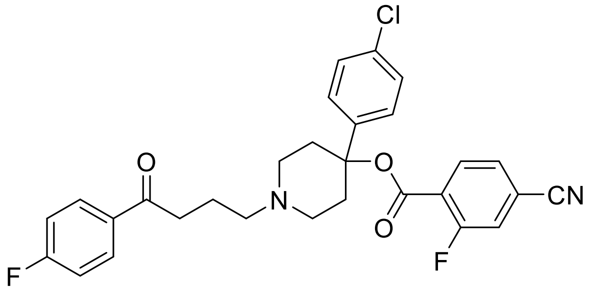

Prepared according to general procedure C using Haloperidol (1.88 g, 5.0 mmol) and 4-cyano-2-fluorobenzoic acid (990 mg, 6.0 mmol, 1.2 equiv.). The crude product was purified by flash column chromatography (40/60 to 30/70 Hexane/Et_2O) to provide the title compound as a white solid (1.674 g, 64% overall yield). ^{1}H NMR (300 MHz, $CDCl_3$) δ: 8.03 – 7.96 (m, 2H), 7.93 (app t, *J* = 7.4 Hz, 1H), 7.53 – 7.42 (m, 2H), 7.31 (app s, 4H), 7.12 (t, *J* = 8.6 Hz, 2H), 2.99 (t, *J* = 6.9 Hz, 2H), 2.92 – 2.82 (m, 2H), 2.58

(d, J = 12.7 Hz, 2H), 2.53 – 2.38 (m, 4H), 2.14 – 1.93 (m, 4H). ^{13}C NMR (75 MHz, $CDCl_3$) δ: 165.78 (d, J = 254.5 Hz), 161.46 (d, J = 3.9 Hz), 161.23 (d, J = 262.9 Hz), 142.03, 133.77, 133.31, 130.75 (d, J = 9.2 Hz), 128.91, 127.96 (d, J = 4.4 Hz), 126.31, 124.09 (d, J = 11.1 Hz), 121.09 (d, J = 26.4 Hz), 117.67 (d, J = 9.6 Hz), 116.76 (d, J = 2.5 Hz), 115.76 (d, J = 21.8 Hz), 82.94, 57.66, 49.12, 36.22, 35.43, 21.85. ^{19}F NMR (282 MHz, $CDCl_3$) δ: -105.6; HRMS (APCI) found $[M+1]^+$ 523.1583, $C_{29}H_{26}ClF_2N_2O_3$ requires 523.1595.

3-(2-(4-(benzyloxy)phenyl)-1-(4-fluorophenyl)-4-oxoazetidin-3-yl)-1-(4-fluorophenyl)propyl 4-cyano-2-fluorobenzoate (OBn-Ezetimibe ester) (S31)

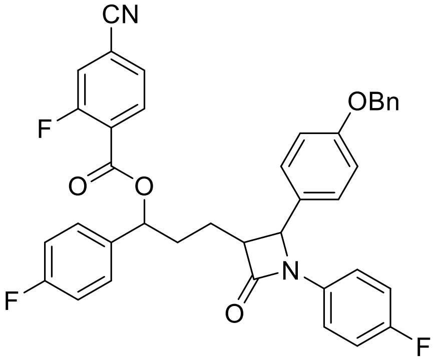

Prepared according to general procedure B using OBn-Ezetimibe (340 mg, 0.68 mmol) and 4-cyano-2-fluorobenzoic acid (116 mg, 0.7 mmol, 1.03 equiv.). The crude product was purified by flash column chromatography (60/40 to 50/50 Hexane/Et_2O) to provide the title compound as a white solid (361 mg, 82% overall yield). ^{1}H NMR (300 MHz, $CDCl_3$) δ: 8.04 (t, J = 7.5 Hz, 1H), 7.53 (d, J = 7.9 Hz, 1H), 7.50 – 7.33 (m, 8H), 7.31 – 7.21 (m, 4H), 7.07 (t, J = 8.6 Hz, 2H), 7.00 (d, J = 8.6 Hz, 2H), 6.95 (t, J = 8.7 Hz, 2H), 6.02 (t, J = 6.6 Hz, 1H), 5.09 (s, 2H), 4.59 (d, J = 2.0 Hz, 1H), 3.13 (td, J = 7.6, 2.0 Hz, 1H), 2.31 – 2.16 (m, 2H), 2.05 – 1.91 (m, 2H). ^{13}C NMR (75 MHz, $CDCl_3$) δ: 166.98, 162.74 (d, J = 247.4 Hz), 162.30 (d, J = 3.9 Hz), 161.88 (d, J = 175.6 Hz), 159.26, 158.52 (d, J = 155.6 Hz), 136.73, 135.08 (d, J = 3.2 Hz), 133.96 (d, J = 2.6 Hz), 133.36 (d, J = 0.9 Hz), 129.57, 128.75, 128.47 (d, J = 8.2 Hz), 128.23, 127.88 (d, J = 4.4 Hz), 127.55, 127.28, 123.18 (d, J = 10.5 Hz), 121.05 (d, J = 26.2 Hz), 118.49 (d, J = 7.8 Hz), 117.84 (d, J = 9.7 Hz), 116.65 (d, J = 2.6 Hz), 116.02 (d, J = 6.8 Hz), 115.73 (d, J = 5.7 Hz), 115.73, 77.10, 70.24, 61.06, 60.06, 33.80, 24.93. ^{19}F NMR (282 MHz, $CDCl_3$) δ: -105.4, -112.8, -117.7; HRMS (APCI) found $[M]^+$ 646.2079, $C_{39}H_{29}F_3N_2O_4$ requires

646.2074.

Pent-3-en-2-yl 4-cyano-2-fluorobenzoate (predominantly trans) (S32)

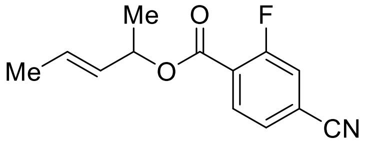

Prepared according to general procedure B using 3-penten-2-ol (predominantly trans, 0.306 mL, 3.0 mmol) and 4-cyano-2-fluorobenzoic acid (495 mg, 3.0 mmol). The crude product was purified by flash column chromatography (90/10 to 70/30 Hexane/Et_2O) to provide the title compound as a white solid (532 mg, 76% yield). ^{1}H NMR (300 MHz, $CDCl_3$) δ: 8.01 (t, *J* = 7.7 Hz, 1H), 7.50 (dd, *J* = 8.1, 1.3 Hz, 1H), 7.43 (dd, *J* = 9.7, 1.3 Hz, 1H), 5.91 – 5.76 (m, 1H), 5.64 – 5.50 (m, 2H), 1.72 (d, *J* = 6.6 Hz, 3H), 1.43 (d, *J* = 6.0 Hz, 3H). ^{13}C NMR (75 MHz, $CDCl_3$) δ: 162.30 (d, *J* = 3.7 Hz), 161.32 (d, *J* = 263.6 Hz), 133.17 (d, *J* = 1.6 Hz), 130.16, 129.49, 127.76 (d, *J* = 4.4 Hz), 124.14 (d, *J* = 10.4 Hz), 120.96 (d, *J* = 26.2 Hz), 117.40 (d, *J* = 9.6 Hz), 116.85 (d, *J* = 2.6 Hz), 73.63, 20.51, 17.82; ^{19}F NMR (282 MHz, $CDCl_3$) δ: -106.5; HRMS (APCI) found $[M]^+$ 233.0845, $C_{13}H_{12}FNO_2$ requires 233.0847.

2-methyl-3-oxobutan-2-yl 4-cyano-2-fluorobenzoate (S33)

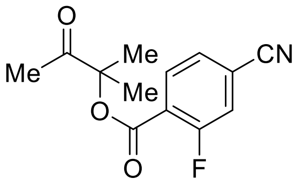

Prepared according to general procedure C using 3-hydroxy-3-methylbutan-2-one (1.46 mL, 8.0 mmol) and 4-cyano-2-fluorobenzoic acid (1.58 g, 9.6 mmol). The crude product was purified by flash column chromatography (90/10 to 85/15 hexane/EtOAc) to provide the title compound as a white solid (1.69 g, 85% yield). ^{1}H NMR (300 MHz, $CDCl_3$) δ 8.03 (t, *J* = 7.5 Hz, 1H), 7.53 (d, *J* = 8.1 Hz, 1H), 7.47 (d, *J* = 9.8 Hz, 1H), 2.21 (s, 3H), 1.63 (s, 6H). ^{13}C NMR (75 MHz, $CDCl_3$) δ 205.76, 162.26, 161.39 (d, *J* = 263.8 Hz), 133.34, 127.92 (d, *J* = 4.3 Hz), 123.24 (d, *J* = 10.7 Hz), 121.09 (d, *J* = 26.0 Hz), 118.02 (d, *J* = 9.5 Hz), 116.69, 86.10, 24.06, 23.64. HRMS (APCI) $[M+H]^+$ found 250.0865, $C_{13}H_{13}FNO_3$ requires 250.0874.

methyl 3-((4-cyanopyridin-2-yl)methoxy)propanoate (S34)

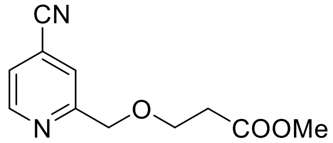

To a stirred solution of 2-(hydroxymethyl)pyridine-4-carbonitrile (402 mg, 3.0 mmol) in THF (6.0 mL) was added NaH (60% in mineral oil, 144 mg, 3.6 mmol) in small portions under argon atmosphere and at 0 °C followed by methyl 3-bromopropionate (0.327 mL, 3.0 mmol). The reaction temperature was slowly raised to room temperature and stirred for 3 h. The reaction mixture was cooled to 10 °C, treated with ice water (20 mL) and extracted with EtOAc (2 x 25 mL). The organic layer was washed with brine (30 mL), dried over anhydrous Na_2SO_4 and concentrated. The crude was purified by flash column chromatography (80/20 to 50/50 Hexane/AcOEt) to provide the title compound as a white solid (370 mg, 56% yield). ^{1}H NMR (300 MHz, $CDCl_3$) δ: 8.75 – 8.65 (m, 1H), 7.74 – 7.65 (m, 1H), 7.45 – 7.36 (m, 1H), 4.70 (s, 2H), 3.88 (t, *J* = 6.2 Hz, 2H), 3.73 (s, 3H), 2.69 (t, *J* = 6.2 Hz, 2H); ^{13}C NMR (75 MHz, $CDCl_3$) δ: 171.87, 160.68, 149.95, 123.89, 122.95, 121.31, 116.75, 73.15, 66.84, 51.98, 34.98; HRMS (APCI) found $[M+1]^+$ 221.0919, $C_{11}H_{13}N_2O_3$ requires 221.0921.

3-phenylisonicotinonitrile (S35)

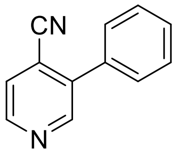

Prepared according to the general procedure F using 3-chloroisonicotino nitrile (693 mg, 5.00 mmol), phenylboronic acid (915 mg, 7.50 mmol), $Pd(PPh_3)_4$ (289 mg, 0.25 mmol) and K_2CO_3 (1.73 g, 12.50 mmol). The crude product was purified by flash column chromatography (95/5 to 80/20 Hexane/EtOAc) to provide the title compound as a white solid (802 mg, 89% yield). ^{1}H NMR (300 MHz, $CDCl_3$) δ 8.87 (s, 1H), 8.76 (d, *J* = 5.1 Hz, 1H), 7.68 – 7.46 (m, 6H). ^{13}C NMR (75 MHz, $CDCl_3$) δ 151.14, 148.86, 138.86, 134.59, 129.70, 129.29, 128.94, 126.16, 118.99, 116.44. HRMS (APCI) found $[M+H]^+$ 181.0758, $C_{12}H_9N_2$ requires 181.0760.

3-(*p*-tolyl)isonicotinonitrile (S36)

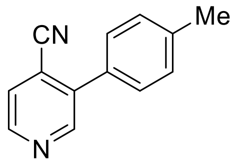

Prepared according to the general procedure F using 3-chloroisonicotino nitrile (339 mg, 2.45 mmol), *p*-tolylboronic acid (500 mg, 3.7 mmol), $Pd(PPh_3)_4$ (142 mg, 0.12 mmol) and K_2CO_3 (848 mg, 6.12 mmol). The crude product was purified by flash column chromatography (95/5 to 80/20 Hexane/EtOAc) to provide the title compound as a white solid (300 mg, 63% yield). ^{1}H NMR (300 MHz, $CDCl_3$) δ 8.87 (s, 1H), 8.74 (d, *J* = 5.0 Hz, 1H), 7.62 (d, *J* = 5.0 Hz, 1H), 7.50 (d, *J* = 8.1 Hz, 2H), 7.37 (d, *J* = 8.1 Hz, 2H), 2.46 (s, 3H). ^{13}C NMR (75 MHz, $CDCl_3$) δ 151.12, 148.55, 139.89, 138.91, 131.71, 130.01, 128.81, 126.14, 118.82, 116.61, 21.43. HRMS (APCI) $[M+H]^+$ found 195.0919, $C_{13}H_{11}N_2$ requires 195.0917.

3-(4-methoxyphenyl)isonicotinonitrile (S37)

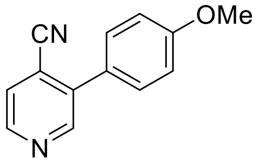

Prepared according to the general procedure F using 3-chloroisonicotino nitrile (303 mg, 2.19 mmol), *p*-tolylboronic acid (500 mg, 3.29 mmol), $Pd(PPh_3)_4$ (126 mg, 0.11 mmol) and K_2CO_3 (756 mg, 5.47 mmol). The crude product was purified by flash column chromatography (95/5 to 80/20 Hexane/EtOAc) to provide the title compound as a white solid (290 mg, 63% yield). ^{1}H NMR (300 MHz, $CDCl_3$) δ 8.85 (s, 1H), 8.70 (d, *J* = 5.0 Hz, 1H), 7.60 (d, *J* = 5.0 Hz, 1H), 7.55 (d, *J* = 8.8 Hz, 2H), 7.07 (d, *J* = 8.8 Hz, 2H), 3.89 (s, 3H). ^{13}C NMR (75 MHz, $CDCl_3$) δ 160.92, 150.94, 148.11, 138.65, 130.26, 126.79, 126.19, 118.65, 116.69, 114.83, 55.56. HRMS (APCI) $[M+H]^+$ found 211.0866, $C_{13}H_{11}N_2O$ requires 211.0866.

3-(4-fluorophenyl)isonicotinonitrile (S38)

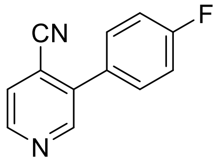

Prepared according to the general procedure F using 3-chloroisonicotino nitrile (330 mg, 2.38 mmol), (4-fluorophenyl)boronic acid (500 mg, 3.57 mmol), $Pd(PPh_3)_4$ (137 mg, 0.12 mmol) and K_2CO_3 (822 mg, 5.95 mmol). The crude product was purified by flash column chromatography (95/5 to 80/20 Hexane/EtOAc) to provide the title compound as a white solid (431 mg, 91% yield). ^{1}H NMR (300 MHz, $CDCl_3$) δ 8.86 (s, 1H), 8.77 (d, *J* = 5.0 Hz, 1H), 7.69 – 7.52 (m, 3H), 7.34 – 7.18 (m, 2H). ^{13}C NMR (75 MHz, $CDCl_3$) δ 163.77 (d, *J* = 250.5 Hz), 151.00, 149.03, 137.86, 130.90 (d, *J* = 8.6 Hz), 130.65 (d, *J* = 3.4 Hz), 126.13, 119.00, 116.52 (d, *J* = 22.0 Hz), 116.31. HRMS (APCI) $[M+H]^+$ found 199.0667, $C_{12}H_8FN_2$ requires 199.0666.

3-(4-(morpholinomethyl)phenyl)isonicotinonitrile (S39)

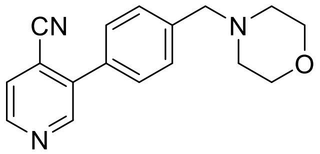

Prepared according to the general procedure F using 3-chloroisonicotino nitrile (208 mg, 1.50 mmol), (4-(morpholinomethyl)phenyl)boronic acid (500 mg, 2.26 mmol), $Pd(PPh_3)_4$ (87 mg, 0.08 mmol) and K_2CO_3 (520 mg, 3.76 mmol). The crude product was purified by flash column chromatography (20/80 to 10/90 Hexane/EtOAc) to provide the title compound as a yellow oil (226 mg, 54% yield). ^{1}H NMR (300 MHz, $CDCl_3$) δ 8.86 (s, 1H), 8.74 (d, *J* = 5.1 Hz, 1H), 7.62 (d, *J* = 5.1 Hz, 1H), 7.59 – 7.47 (m, 4H), 3.81 – 3.67 (m, 4H), 3.58 (s, 2H), 2.53 – 2.44 (m, 4H). ^{13}C NMR (75 MHz, $cdcl_3$) δ 151.04, 148.70, 139.84, 138.58, 133.34, 129.85, 128.81, 126.13, 118.78, 116.46, 67.09, 63.01, 53.78. HRMS (APCI) $[M+H]^+$ found 280.1449, $C_{17}H_{18}N_3O$ requires 280.1444.

3-(m-tolyl)isonicotinonitrile (S40)

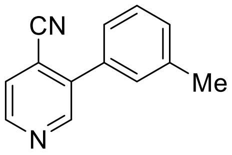

Prepared according to the general procedure F using 3-chloroisonicotino nitrile (340 mg,

2.45 mmol), *m*-tolylboronic acid (500 mg, 3.67 mmol), $Pd(PPh_3)_4$ (141 mg, 0.12 mmol) and K_2CO_3 (846 mg, 6.12 mmol). The crude product was purified by flash column chromatography (95/5 to 80/20 Hexane/EtOAc) to provide the title compound as a white solid (434 mg, 91% yield). ^{1}H NMR (300 MHz, $CDCl_3$) δ 8.87 (s, 1H), 8.75 (d, *J* = 5.0 Hz, 1H), 7.63 (d, *J* = 5.0 Hz, 1H), 7.51 – 7.31 (m, 4H), 2.47 (s, 3H). ^{13}C NMR (75 MHz, $CDCl_3$) δ 151.00, 148.57, 139.11, 134.50, 130.47, 129.55, 129.18, 126.19, 126.05, 119.07, 116.44, 21.56. HRMS (APCI) $[M+H]^+$ found 195.0916, $C_{13}H_{11}N_2$ requires 195.0917.

3-(3-methoxyphenyl)isonicotinonitrile (S41)

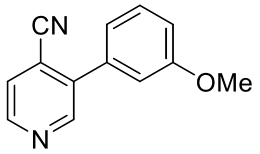

Prepared according to the general procedure F using 3-chloroisonicotino nitrile (303 mg, 2.19 mmol), (3-methoxyphenyl)boronic acid (500 mg, 3.29 mmol), $Pd(PPh_3)_4$ (126 mg, 0.11 mmol) and K_2CO_3 (756 mg, 5.47 mmol). The crude product was purified by flash column chromatography (95/5 to 80/20 Hexane/EtOAc) to provide the title compound as a white solid (403 mg, 88% yield). ^{1}H NMR (300 MHz, $CDCl_3$) δ 8.86 (s, 1H), 8.74 (d, *J* = 5.0 Hz, 1H), 7.61 (d, *J* = 5.0 Hz, 1H), 7.45 (t, *J* = 8.0 Hz, 1H), 7.21 – 7.00 (m, 3H). ^{13}C NMR (75 MHz, $CDCl_3$) δ 160.14, 151.06, 148.91, 138.71, 135.83, 130.39, 126.16, 121.26, 119.00, 116.41, 115.41, 114.48, 55.58. HRMS (APCI) $[M+H]^+$ found 211.0865, $C_{13}H_{11}N_2O$ requires 211.0866.

3-((4-cyanopyridin-2-yl)methoxy)propanoic acid (S42)

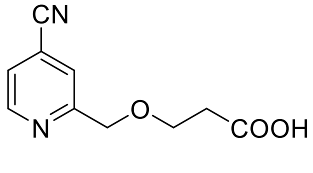

3-((4-cyanopyridin-2-yl)methoxy)propanoic acid was synthesized according to literature procedure (*Tetrahedron Letters* **2007**, *48*, 2497−2499). Methyl 3-((4-cyanopyridin-2-yl)methoxy)propanoate (100 mg, 0.45 mmol) was dissolved in 2 mL of acetonitrile containing water (2 vol%). Triethylamine (0.188 mL, 1.35 mmol, 3 equiv.) and lithium bromide (390 mg, 4.5 mmol, 10 equiv.) were added and the reaction

was stirred vigorously at room temperature for 3 hours. The liquids were removed under reduced pressure and the residue redissolved in water (10 mL) and AcOEt (10 mL). The organic phase was discarded and the aqueous phase was washed with AcOEt (2 x 10 mL), acidified with aqueous HCl (1 M) until pH=2-3 and extracted with AcOEt (3 x 10 mL). The organic phases recovered after the acidification were combined, dried over Na_2SO_4, filtered and concentrated *in vacuo* to provide the title compound as a white solid (80 mg, 86% yield, 95% purity). ^{1}H NMR (300 MHz, $CDCl_3$) δ: 8.72 (d, *J* = 4.8 Hz, 1H), 7.73 (s, 1H), 7.44 (d, *J* = 4.8 Hz, 1H), 6.09 (bs, 1H), 4.72 (s, 2H), 3.89 (t, *J* = 6.1 Hz, 2H), 2.74 (t, *J* = 6.1 Hz, 2H); ^{13}C NMR (75 MHz, $CDCl_3$) δ: 176.13, 160.43, 149.80, 124.09, 123.18, 121.52, 116.63, 72.91, 66.60, 34.92; HRMS (APCI) found $[M-1]^+$ 207.0764, $C_{10}H_{11}N_2O_3$ requires 207.0764.

3. Metal-free deoxygenative coupling of alcohol-derived benzoates and pyridines for small molecules synthesis

General procedure G

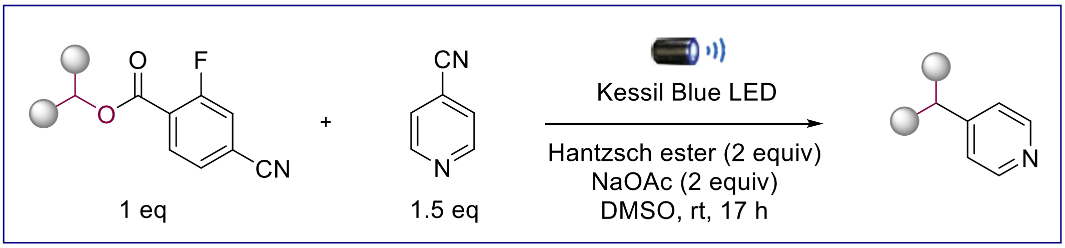

In a 10 mL Schlenk tube equipped with stir bar, the appropriate alcohol derivative (0.1 mmol, 1 equiv.), 4-cyanopyridine (15.6 mg, 0.15 mmol, 1.5 equiv.), Hantzsch ester (50.6 mg, 0.2 mmol, 2 equiv.) and sodium acetate (0.2 mmol, 2 equiv.) were dissolved in anhydrous DMSO (2 mL) under air. *The use of anhydrous DMSO serves to maximize the reproducibility of the reaction, although a small amount of water is tolerated*. The solution was degassed via freeze pump thaw method (3 cycles), backfilled with Argon and irradiated with visible light. *The Schlenk tube was placed at 3 cm distance from the Kessil LED lamp and the reaction temperature was kept contast using a fan placed on top of the reaction at 20 cm distance*. After 17 hours, two equal reactions (0.1 mmol scale) were joined and placed in a separation funnel; DCM (20 mL) and 5% Na_2CO_3 aqueous solution (4 mL) were added. The organic phase was separated and the aqueous phase was extracted once with DCM (15 mL). The combined organic phases were washed twice with saturated Brine solution (40 mL), dried over sodium sulfate and concentrated under reduced pressure. The reaction crude was purified by flash column chromatography (silica gel) under the stated conditions to provide the pure coupling product. *All isolated yields are given on 0.2 mmol scale*.

Important note: The Schlenk tube and the freeze pump thaw method were chosen for maximize the reproducibility of the reaction, given its sensitivity to oxygen. However, traces of oxygen can be tolerated, and similar yields were obtained for selected substrates when the reactions were conducted in microwave vials and degassed via

Argon purging for 10 minutes.

4-(1-phenylpropyl)pyridine (10)

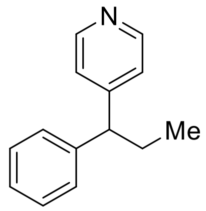

Prepared according to general procedure G using 1-phenylpropyl 4-cyano-2-fluorobenzoate **3** (28.3 mg, 0.1 mmol) and 4-cyanopyridine (15.6 mg, 0.15 mmol); 2 x reaction of 0.1 mmol scale. The crude product was purified by flash column chromatography (50/50 to 30/70 Hexane/Et_2O) to provide the title compound as a colorless viscous oil (33 mg, 84% yield). ^{1}H NMR (300 MHz, $CDCl_3$) δ: 8.49 (d, *J* = 4.6 Hz, 2H), 7.34 – 7.27 (m, 2H), 7.25 – 7.17 (m, 3H), 7.15 (d, *J* = 5.9 Hz, 2H), 3.77 (t, *J* = 7.7 Hz, 1H), 2.08 (p, *J* = 7.4 Hz, 2H), 0.91 (t, *J* = 7.3 Hz, 3H). ^{13}C NMR (75 MHz, $CDCl_3$) δ: 154.12, 149.96, 143.32, 128.77, 128.06, 126.79, 123.43, 52.78, 28.06, 12.67. HRMS (APCI) found $[M+1]^+$ 198.1271, $C_{14}H_{16}N$ requires 198.1277.

4-(1-(pyridin-4-yl)ethyl)benzonitrile (11)

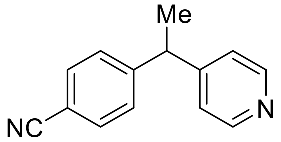

Prepared according to general procedure G using 1-(4-cyanophenyl)ethyl 4-cyano-2-fluorobenzoate **S1** (29.5 mg, 0.1 mmol) and 4-cyanopyridine (15.6 mg, 0.15 mmol), Hantzsch ester (50.6 mg, 0.2 mmol) and NaOAc (16.4 mg, 0.2 mmol); 2 x reaction of 0.1 mmol scale. The crude product was purified by flash column chromatography (25/75 to 20/80 hexane/Et_2O) to provide the title compound as a colorless oil (23 mg, 53% yield). ^{1}H NMR (300 MHz, $CDCl_3$) δ 8.62 – 8.48 (d, *J* = 5.8 Hz, 2H), 7.62 (d, *J* = 8.4 Hz, 2H), 7.39 – 7.27 (m, 2H), 7.11 (d, *J* = 6.1 Hz, 2H), 4.19 (q, *J* = 7.2 Hz, 1H), 1.67 (d, *J* = 7.2 Hz, 3H). ^{13}C NMR (75 MHz, $CDCl_3$) δ 153.45, 150.29, 149.95, 132.65, 128.60, 122.95, 118.80, 110.89, 44.45, 20.86. HRMS (APCI) $[M+H]^+$ found 209.1075, $C_{14}H_{12}N_2$ requires 209.1073.

4-benzhydrylpyridine (12)

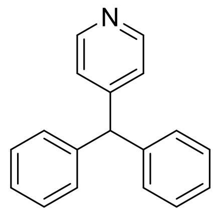

Prepared according to general procedure G using benzhydryl 4-cyano-2-fluorobenzoate **S2** (33 mg, 0.1 mmol) and 4-cyanopyridine (15.6 mg, 0.15 mmol); 2 x reaction of 0.1 mmol scale. The crude product was purified by flash column chromatography (50/50 to 30/70 Hexane/Et_2O) to provide the title compound as a white solid (35 mg, 72% yield). ^{1}H NMR (300 MHz, $CDCl_3$) δ: 8.52 (d, *J* = 5.2 Hz, 2H), 7.39 – 7.19 (m, 6H), 7.10 (d, *J* = 7.1 Hz, 4H), 7.05 (d, *J* = 5.6 Hz, 2H), 5.51 (s, 1H). ^{13}C NMR (75 MHz, $CDCl_3$) δ: 152.93, 149.94, 142.24, 129.48, 128.73, 127.01, 124.78, 56.42. HRMS (APCI) found $[M+1]^+$ 246.1274, $C_{18}H_{16}N$ requires 246.1277.

4-(1,2-dihydroacenaphthylen-1-yl)pyridine (13)

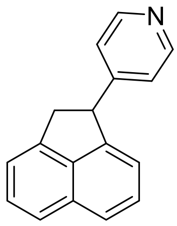

Prepared according to general procedure G using 1,2-dihydroacenaphthylen-1-yl 4-cyano-2-fluorobenzoate **S3** (31.7 mg, 0.1 mmol) and 4-cyanopyridine (15.6 mg, 0.15 mmol); 2 x reaction of 0.1 mmol scale. The crude product was purified by flash column chromatography (50/50 to 30/70 Hexane/Et_2O) to provide the title compound as a colorless viscous oil (37 mg, 80% yield). ^{1}H NMR (300 MHz, $CDCl_3$) δ: 8.51 (d, *J* = 5.6 Hz, 2H), 7.69 (d, *J* = 8.2 Hz, 2H), 7.52 (app t, *J* = 7.3 Hz, 1H), 7.47 (app t, *J* = 7.5 Hz, 1H), 7.33 (d, *J* = 6.9 Hz, 1H), 7.10 (d, *J* = 6.0 Hz, 3H), 4.85 (dd, *J* = 8.7, 3.7 Hz, 1H), 3.98 (dd, *J* = 17.5, 8.8 Hz, 1H), 3.37 (dd, *J* = 17.5, 3.8 Hz, 1H). ^{13}C NMR (125 MHz, $CDCl_3$) δ: 154.60, 150.25, 146.96, 143.33, 138.86, 131.72, 128.43, 128.23, 123.67, 123.13, 123.00, 120.35, 119.73, 48.89, 41.14. HRMS (APCI) found $[M+1]^+$ 232.1120, $C_{17}H_{14}N$ requires 232.1121.

4-(3-(1,3-dioxan-2-yl)-1-phenylpropyl)pyridine (14)

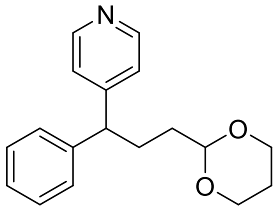

Prepared according to general procedure G using 3-(1,3-dioxan-2-yl)-1-phenylpropyl 4-cyano-2-fluorobenzoate **S4** (37 mg, 0.1 mmol) and 4-cyanopyridine (15.6 mg, 0.15 mmol); 2 x reaction of 0.1 mmol scale. The crude product was purified by flash column chromatography (40/60 to 20/80 Hexane/Et_2O) to provide the title compound as a colorless viscous oil (41 mg, 72% yield). ^{1}H NMR (300 MHz, $CDCl_3$) δ: 8.50 (bs, 2H), 7.36 – 7.25 (m, 2H), 7.26 – 7.09 (m, 5H), 4.52 (t, *J* = 4.8 Hz, 1H), 4.09 (dd, *J* = 11.2, 4.6 Hz, 2H), 3.87 (t, *J* = 7.8 Hz, 1H), 3.73 (t, *J* = 11.8 Hz, 2H), 2.17 (q, *J* = 7.9 Hz, 2H), 2.11 – 1.97 (m, 1H), 1.64 – 1.51 (m, 2H), 1.33 (d, *J* = 13.4 Hz, 1H). ^{13}C NMR (75 MHz, $CDCl_3$) δ: 153.91, 149.89, 143.02, 128.81, 128.05, 126.85, 123.49, 101.96, 67.00, 50.73, 33.65, 29.13, 25.89. HRMS (APCI) found $[M+1]^+$ 284.1643, $C_{18}H_{22}NO_2$ requires 284.1645.

4-(1-(4-chlorophenyl)-3-(1,3-dioxan-2-yl)propyl)pyridine (15)

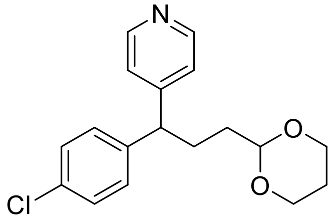

Prepared according to general procedure G using 1-(4-chlorophenyl)-3-(1,3-dioxan-2-yl)propyl 4-cyano-2-fluorobenzoate **S5** (40 mg, 0.1 mmol) and 4-cyanopyridine (15.6 mg, 0.15 mmol); 2 x reaction of 0.1 mmol scale. The crude product was purified by flash column chromatography (40/60 to 10/90 Hexane/Et_2O) to provide the title compound as a yellowish viscous oil (44 mg, 69% yield). ^{1}H NMR (300 MHz, $CDCl_3$) δ: 8.49 (d, *J* = 5.6 Hz, 2H), 7.31 – 7.24 (m, 2H), 7.23 – 7.09 (m, 4H), 4.52 (t, *J* = 4.9 Hz, 1H), 4.08 (dd, *J* = 11.3, 4.9 Hz, 2H), 3.85 (t, *J* = 7.8 Hz, 1H), 3.73 (td, *J* = 12.4, 2.4 Hz, 2H), 2.21 – 1.97 (m, 4H), 1.60 – 1.49 (m, 2H),

1.33 (app d, *J* = 13.3 Hz, 1H). ^{13}C NMR (75 MHz, $CDCl_3$) δ: 153.42, 150.00, 141.54, 132.70, 129.41, 128.96, 123.27, 101.80, 67.01, 50.04, 33.52, 29.02, 25.87. HRMS (APCI) found $[M+1]^+$ 318.1259, $C_{18}H_{21}ClNO_2$ requires 318.1255.

4-(1-(benzo[d][1,3]dioxol-5-yl)-3-(1,3-dioxan-2-yl)propyl)pyridine (16)

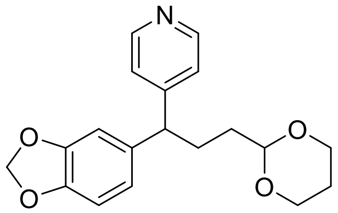

Prepared according to general procedure G using 1-(benzo[d][1,3]dioxol-5-yl)-3-(1,3-dioxan-2-yl)propyl 4-cyano-2-fluorobenzoate **S6** (41.3 mg, 0.1 mmol) and 4-cyanopyridine (15.6 mg, 0.15 mmol); 2 x reaction of 0.1 mmol scale. The crude product was purified by flash column chromatography (40/60 to 10/90 Hexane/Et_2O) to provide the title compound as a yellowish viscous oil (44 mg, 67% yield). ^{1}H NMR (300 MHz, $CDCl_3$) δ: 8.47 (d, *J* = 4.5 Hz, 2H), 7.13 (d, *J* = 5.2 Hz, 2H), 6.76 – 6.61 (m, 3H), 5.90 (s, 2H), 4.51 (t, *J* = 4.9 Hz, 1H), 4.07 (dd, *J* = 11.2, 4.7 Hz, 2H), 3.82 – 3.65 (m, 3H), 2.16 – 1.95 (m, 3H), 1.63 – 1.46 (m, 2H), 1.31 (d, *J* = 13.5 Hz, 1H). ^{13}C NMR (75 MHz, $CDCl_3$) δ: 153.97, 150.00, 148.07, 146.44, 136.96, 123.20, 121.20, 108.43, 108.32, 101.96, 101.12, 67.02, 50.34, 33.62, 29.20, 25.90. HRMS (APCI) found $[M+1]^+$ 328.1545, $C_{19}H_{22}NO_4$ requires 328.1543.

4-(3-(1,3-dioxan-2-yl)-1-(naphthalen-2-yl)propyl)pyridine (17)

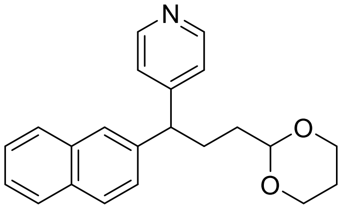

Prepared according to general procedure G using 3-(1,3-dioxan-2-yl)-1-(naphthalen-2-yl)propyl 4-cyano-2-fluorobenzoate **S7** (42 mg, 0.1 mmol) and 4-cyanopyridine (15.6 mg, 0.15 mmol); 2 x reaction of 0.1 mmol scale. The crude product was purified by flash column chromatography (40/60 to 20/80 Hexane/Et_2O) to provide the title compound as a colorless viscous oil (47 mg, 71%

yield). ^{1}H NMR (300 MHz, $CDCl_3$) δ: 8.50 (bs, 2H), 7.85 – 7.65 (m, 4H), 7.53 – 7.38 (m, 2H), 7.33 – 7.14 (m, 3H), 4.54 (t, *J* = 4.9 Hz, 1H), 4.16 – 3.96 (m, 3H), 3.74 (td, *J* = 12.3, 2.0 Hz, 2H), 2.32 – 2.19 (m, 2H), 2.14 – 1.96 (m, 1H), 1.68 – 1.54 (m, 2H), 1.32 (d, *J* = 13.4 Hz, 1H). ^{13}C NMR (125 MHz, $CDCl_3$) δ: 154.03, 149.71, 140.35, 133.61, 132.50, 128.60, 127.86, 127.73, 126.52, 126.40, 126.33, 125.89, 101.93, 67.02, 50.76, 33.67, 28.90, 25.88. HRMS (APCI) found $[M+1]^+$ 334.1803, $C_{22}H_{24}NO_2$ requires 334.1802.

4-(3-(1,3-dioxan-2-yl)-1-(furan-2-yl)propyl)pyridine (18)

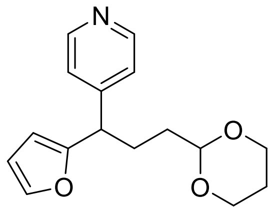

Prepared according to general procedure G using 3-(1,3-dioxan-2-yl)-1-(furan-2-yl)propyl 4-cyano-2-fluorobenzoate **S8** (36 mg, 0.1 mmol) and 4-cyanopyridine (15.6 mg, 0.15 mmol); 2 x reaction of 0.1 mmol scale. The crude product was purified by flash column chromatography (40/60 to 10/90 Hexane/Et_2O) to provide the title compound as a yellowish viscous oil (34 mg, 63% yield). ^{1}H NMR (300 MHz, $CDCl_3$) δ: 8.53 (bs, 2H), 7.31 (s, 1H), 7.16 (bs, 2H), 6.35 – 6.23 (m, 1H), 6.12 (d, *J* = 3.1 Hz, 1H), 4.50 (t, *J* = 5.0 Hz, 1H), 4.08 (dd, *J* = 11.2, 4.6 Hz, 2H), 3.92 (t, *J* = 7.7 Hz, 1H), 3.72 (t, *J* = 11.1 Hz, 2H), 2.28 – 2.11 (m, 1H), 2.11 – 1.91 (m, 2H), 1.67 – 1.45 (m, 2H), 1.32 (d, *J* = 13.5 Hz, 1H). ^{13}C NMR (75 MHz, $CDCl_3$) δ: 155.79, 151.67, 149.94, 141.95, 123.35, 110.28, 106.38, 101.86, 67.03, 44.67, 33.25, 28.48, 25.90. HRMS (APCI) found $[M+1]^+$ 274.1439, $C_{16}H_{20}NO_3$ requires 274.1438.

4-(3-(1,3-dioxan-2-yl)-1-(thiophen-2-yl)propyl)pyridine (19)

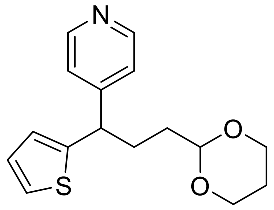

Prepared according to general procedure G using 3-(1,3-dioxan-2-yl)-1-(thiophen-2-yl)propyl 4-cyano-2-fluorobenzoate **S9** (37.5 mg, 0.1 mmol) and 4-cyanopyridine (15.6 mg, 0.15 mmol); 2 x reaction of 0.1 mmol scale. The crude product was purified by flash column chromatography (40/60 to 10/90 Hexane/Et_2O) to provide the title compound as a yellowish viscous oil (42 mg, 73% yield). ^{1}H NMR (300 MHz, $CDCl_3$) δ: 8.50 (d, *J* = 4.3 Hz, 2H), 7.18 (dd, *J* = 4.6, 1.4 Hz, 2H), 7.16 (dd, *J* = 5.1, 1.2 Hz, 1H), 6.92 (dd, *J* = 5.1, 3.5 Hz, 1H), 6.85 (dt, *J* = 3.5, 1.0 Hz, 1H), 4.51 (t, *J* = 5.0 Hz, 1H), 4.13 – 4.04 (m, 3H), 3.76 – 3.68 (m, 2H), 2.26 – 1.98 (m, 3H), 1.66 –1.49 (m, 2H), 1.34 – 1.28 (m, 1H). ^{13}C NMR (125 MHz, $CDCl_3$) δ: 153.24, 150.12, 146.84, 126.86, 124.61, 124.21, 123.10, 101.79, 67.00, 46.17, 33.49, 30.97, 25.86. HRMS (APCI) found $[M+1]^+$ 290.1215, $C_{16}H_{20}NO_2S$ requires 290.1209.

3-(3-(1,3-dioxan-2-yl)-1-(pyridin-4-yl)propyl)-2-chloropyridine (20)

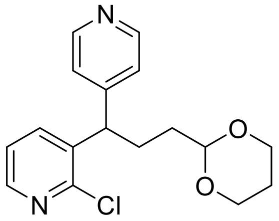

Prepared according to general procedure G using 1-(2-chloropyridin-3-yl)-3-(1,3-dioxan-2-yl)propyl 4-cyano-2-fluorobenzoate **S10** (40.5 mg, 0.1 mmol) and 4-cyanopyridine (15.6 mg, 0.15 mmol); 2 x reaction of 0.1 mmol scale. The crude product was purified by flash column chromatography (40/60 to 20/80 Hexane/AcOEt) to provide the title compound as a yellowish viscous oil (26 mg, 57% yield). ^{1}H NMR (300 MHz, $CDCl_3$) δ: 8.51 (d, *J* = 5.5 Hz, 2H), 8.27 (dd, *J* = 4.6, 1.2 Hz, 1H), 7.63 (dd, *J* = 7.8, 1.2 Hz, 1H), 7.24 (dd, *J* = 7.8, 4.6 Hz, 1H), 7.14 (d, *J* = 5.5 Hz, 2H), 4.54 (t, *J* = 4.8 Hz, 1H), 4.40 (t, *J* = 7.8 Hz, 1H), 4.08 (dd, *J* = 11.4, 4.4 Hz, 2H), 3.73 (t, *J* = 12.0 Hz, 2H), 2.22 – 1.96 (m, 3H), 1.64 – 1.52 (m, 2H), 1.33 (d, *J* = 13.5 Hz, 1H). ^{13}C NMR (75 MHz, $CDCl_3$) δ: 151.75, 151.34, 150.21, 148.02, 137.27, 123.49, 122.98, 101.52, 67.04, 46.11, 33.15, 28.53, 25.86. HRMS (APCI) found $[M+1]^+$ 319.1203, $C_{17}H_{20}ClN_2O_2$ requires 319.1208.

***tert*-butyl 3-(3-(1,3-dioxan-2-yl)-1-(pyridin-4-yl)propyl)-1H-indole-1-carboxylate (21)**

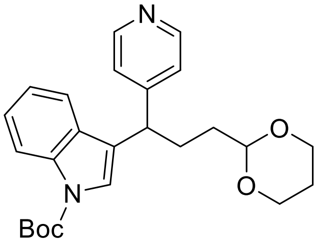

Prepared according to general procedure G using *tert*-butyl 3-(1-((4-cyano-2-fluorobenzoyl)oxy)-3-(1,3-dioxan-2-yl)propyl)-1H-indole-1-carboxylate **S11** (51 mg, 0.1 mmol) and 4-cyanopyridine (15.6 mg, 0.15 mmol); 2 x reaction of 0.1 mmol scale. The crude product was purified by flash column chromatography (40/60 to 10/90 Hexane/Et_2O) to provide the title compound as a yellowish viscous oil (55 mg, 65% yield). ^1H NMR (300 MHz, $CDCl_3$) δ: 8.50 (s, 2H), 8.10 (d, J = 8.2 Hz, 1H), 7.55 (s, 1H), 7.32 – 7.19 (m, 4H), 7.12 (t, J = 7.5 Hz, 1H), 4.55 (t, J = 4.9 Hz, 1H), 4.17 – 4.02 (m, 3H), 3.75 (t, J = 11.5 Hz, 2H), 2.39 – 2.23 (m, 1H), 2.21 – 1.97 (m, 2H), 1.78 – 1.55 (m, 2H), 1.70 (s, 9H), 1.34 (d, J = 13.4 Hz, 1H). ^{13}C NMR (75 MHz, $CDCl_3$) δ: 153.24, 149.92, 149.80, 135.78, 129.81, 124.63, 123.54, 122.98, 122.57, 122.42, 119.47, 115.45, 101.91, 83.94, 67.00, 42.06, 33.43, 29.03, 28.36, 25.88. HRMS (APCI) found $[M+1]^+$ 423.2279, $C_{25}H_{31}N_2O_4$ requires 423.2278.

***tert*-butyl 4-(3-(1,3-dioxan-2-yl)-1-(pyridin-4-yl)propyl)-1H-imidazole-1-carboxylate (22)**

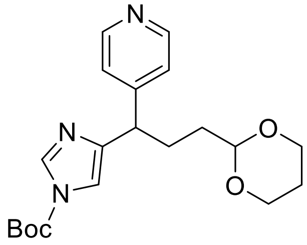

Prepared according to general procedure G using *tert*-butyl 4-(1-((4-cyano-2-fluorobenzoyl)oxy)-3-(1,3-dioxan-2-yl)propyl)-1H-imidazole-1-carboxylate **S12** (46 mg, 0.1 mmol) and 4-cyanopyridine (15.6 mg, 0.15 mmol); 2 x reaction of 0.1 mmol scale. The crude product was purified by flash column chromatography (10/90 to 5/95 Hexane/AcOEt) to provide the title compound as a yellowish viscous oil

(23 mg, 31% yield). ^{1}H NMR (300 MHz, $CDCl_3$) δ: 8.49 (bs, 2H), 7.99 (s, 1H), 7.22 (d, *J* = 4.7 Hz, 2H), 7.15 (s, 1H), 4.51 (t, *J* = 5.0 Hz, 1H), 4.07 (dd, *J* = 10.9, 4.8 Hz, 2H), 3.83 (t, *J* = 7.7 Hz, 1H), 3.72 (t, *J* = 11.1 Hz, 2H), 2.30 – 2.15 (m, 1H), 2.15 – 1.92 (m, 2H), 1.66 – 1.45 (m, 2H), 1.60 (s, 9H), 1.32 (d, *J* = 13.5 Hz, 1H). ^{13}C NMR (75 MHz, $CDCl_3$) δ: 152.57, 149.93, 147.00, 137.18, 123.91, 113.38, 101.99, 85.73, 67.02, 44.51, 33.38, 28.94, 28.05, 25.91. HRMS (APCI) found $[M+1]^+$ 374.2078, $C_{20}H_{28}N_3O_4$ requires 374.2074.

***tert*-butyl 4-(2-phenyl-2-(pyridin-4-yl)ethyl)piperidine-1-carboxylate (23)**

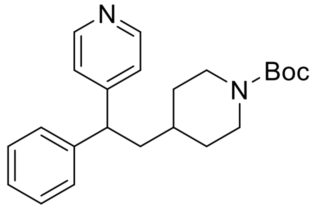

Prepared according to general procedure G using *tert*-butyl 4-(2-((4-cyano-2-fluorobenzoyl)oxy)-2-phenylethyl)piperidine-1-carboxylate **S13** (45 mg, 0.1 mmol) and 4-cyanopyridine (15.6 mg, 0.15 mmol); 2 x reaction of 0.1 mmol scale. The crude product was purified by flash column chromatography (50/50 to 30/70 Hexane/Et_2O) to provide the title compound as a yellowish viscous oil (51 mg, 70% yield). ^{1}H NMR (300 MHz, $CDCl_3$) δ: 8.50 (d, *J* = 3.7 Hz, 2H), 7.31 (t, *J* = 6.9 Hz, 2H), 7.23 (t, *J* = 7.2 Hz, 3H), 7.16 (d, *J* = 5.5 Hz, 2H), 4.11 – 3.96 (m, 3H), 2.57 (t, *J* = 12.2 Hz, 2H), 2.08 – 1.88 (m, 2H), 1.77 – 1.60 (m, 2H), 1.45 (s, 9H), 1.36 – 1.06 (m, 3H). ^{13}C NMR (75 MHz, $CDCl_3$) δ: 154.89, 154.06, 149.91, 142.87, 128.90, 127.91, 126.96, 123.26, 79.39, 47.54, 43.86, 41.93, 33.53, 32.38, 32.10, 30.44, 28.56. HRMS (APCI) found $[M+1]^+$ 367.2395, $C_{23}H_{31}N_2O_2$ requires 367.2380.

***tert*-butyl 4-(2-(5-chlorothiophen-2-yl)-2-(pyridin-4-yl)ethyl)piperidine-1-carboxylate (24)**

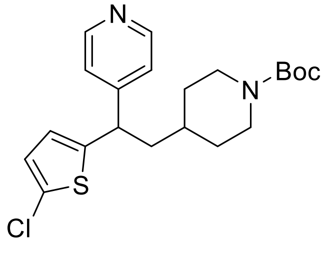

Prepared according to general procedure G using *tert*-butyl 4-(2-(5-chlorothiophen-2-yl)-2-((4-cyano-2-fluorobenzoyl)oxy)ethyl)piperidine-1-carboxylate **S14** (49 mg, 0.1 mmol) and 4-cyanopyridine (15.6 mg, 0.15 mmol); 2 x reaction of 0.1 mmol scale. The crude product was purified by flash column chromatography (50/50 to 10/90 Hexane/Et_2O) to provide the title compound as a yellowish viscous oil (51 mg, 63% yield). ^1H NMR (300 MHz, $CDCl_3$) δ: 8.53 (s, 2H), 7.14 (d, J = 4.4 Hz, 2H), 6.73 (d, J = 3.5 Hz, 1H), 6.60 (d, J = 3.3 Hz, 1H), 4.19 – 3.94 (m, 3H), 2.57 (t, J = 12.5 Hz, 2H), 2.02 – 1.79 (m, 2H), 1.65 (d, J = 11.8 Hz, 2H), 1.43 (s, 9H), 1.37 – 1.04 (m, 3H). ^{13}C NMR (75 MHz, $CDCl_3$) δ: 154.88, 152.55, 150.35, 145.60, 128.71, 125.93, 123.96, 122.83, 79.52, 43.83, 43.48, 43.25, 33.60, 32.09, 30.46, 28.57. HRMS (APCI) found $[M+1]^+$ 407.1562, $C_{21}H_{28}ClN_2O_2S$ requires 407.1555.

4-(1-phenylhex-5-en-1-yl)pyridine (25)

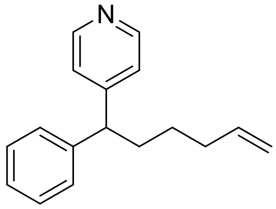

Prepared according to general procedure G using 1-phenylhex-5-en-1-yl 4-cyano-2-fluorobenzoate **S15** (32.3 mg, 0.1 mmol) and 4-cyanopyridine (15.6 mg, 0.15 mmol); 2 x reaction of 0.1 mmol scale. The crude product was purified by flash column chromatography (60/40 to 40/60 Hexane/Et_2O) to provide the title compound as a colorless oil (15 mg, 74% yield). ^1H NMR (300 MHz, $CDCl_3$) δ: 8.51 (d, J = 5.9 Hz, 2H), 7.36 – 7.27 (m, 2H), 7.27 – 7.19 (m, 3H), 7.19 – 7.13 (m, 2H), 5.88 – 5.69 (m, 1H), 5.09 – 4.89 (m, 2H), 3.89 (t, J = 7.8 Hz, 1H), 2.16 – 2.02 (m, 4H), 1.45 – 1.32 (m, 2H). ^{13}C NMR (75 MHz, $CDCl_3$) δ: 154.13, 150.00, 143.31, 138.42, 128.81, 128.01, 126.83, 123.35, 115.02, 50.84, 34.49, 33.69, 27.20. HRMS (APCI) found $[M+1]^+$ 238.1590, $C_{17}H_{20}N$ requires 238.1590.

4-(1-phenylcyclohexyl)pyridine (26)

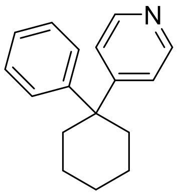

Prepared according to general procedure G using 1-phenylcyclohexyl 4-cyano-2-fluorobenzoate **S16** (32.4 mg, 0.1 mmol) and 4-cyanopyridine (15.6 mg, 0.15 mmol); 2 x reaction of 0.1 mmol scale. The crude product was purified by flash column chromatography (60/40 to 40/60 Hexane/Et_2O) to provide the title compound as a yellowish viscous oil (34 mg, 72% yield). ^{1}H NMR (300 MHz, $CDCl_3$) δ: 8.46 (d, *J* = 5.4 Hz, 2H), 7.33 – 7.21 (m, 4H), 7.16 (d, *J* = 5.3 Hz, 3H), 2.35 – 2.18 (m, 4H), 1.65 – 1.44 (m, 6H). ^{13}C NMR (75 MHz, $CDCl_3$) δ: 157.87, 149.97, 146.75, 128.63, 127.21, 126.15, 122.60, 46.27, 36.51, 26.27, 22.83. HRMS (APCI) found $[M+1]^+$ 238.1585, $C_{17}H_{20}N$ requires 238.1590.

4-(1-phenylcyclopentyl)pyridine (27)

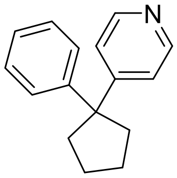

Prepared according to general procedure G using 1-phenylcyclopentyl 4-cyano-2-fluorobenzoate **S17** (31 mg, 0.1 mmol) and 4-cyanopyridine (15.6 mg, 0.15 mmol); 2 x reaction of 0.1 mmol scale. The crude product was purified by flash column chromatography (60/40 to 40/60 Hexane/Et_2O) to provide the title compound as a yellowish viscous oil (31 mg, 70% yield). ^{1}H NMR (300 MHz, $CDCl_3$) δ: 8.59 (bs, 2H), 7.34 – 7.13 (m, 7H), 2.32 (bs, 4H), 1.75 (bs, 4H). ^{13}C NMR (75 MHz, $CDCl_3$) δ: 157.86, 149.72, 147.23, 128.44, 127.12, 126.26, 122.63, 55.76, 38.22, 23.03. HRMS (APCI) found $[M+1]^+$ 224.1433, $C_{16}H_{18}N$ requires 224.1434.

4-(1-phenylcyclobutyl)pyridine (28)

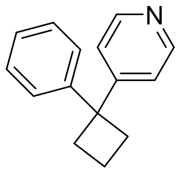

Prepared according to general procedure G using 1-phenylcyclobutyl 4-cyano-2-fluorobenzoate **S18** (29.5 mg, 0.1 mmol) and 4-cyanopyridine (15.6 mg, 0.15 mmol); 2 x reaction of 0.1 mmol scale. The crude product was purified by flash column chromatography (60/40 to 40/60 Hexane/Et_2O) to provide the title compound as a yellowish viscous oil (26 mg, 62% yield). ^{1}H NMR (300 MHz, $CDCl_3$) δ: 8.49 (bs, 2H), 7.35 – 7.25 (m, 4H), 7.24 – 7.15 (m, 3H), 2.86 – 2.64 (m, 4H), 2.00 (p, *J* = 7.4 Hz, 2H). ^{13}C NMR (75 MHz, $CDCl_3$) δ: 158.51, 149.90, 148.03, 128.63, 126.29, 126.22, 121.60, 50.90, 34.73, 16.74. HRMS (APCI) found $[M+1]^+$ 210.1271, $C_{15}H_{16}N$ requires 210.1277.

4-(1-phenylcycloheptyl)pyridine (29)

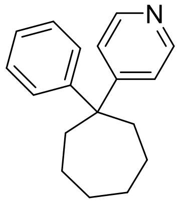

Prepared according to general procedure G using 1-phenylcycloheptyl 4-cyano-2-fluorobenzoate **S19** (34 mg, 0.1 mmol) and 4-cyanopyridine (15.6 mg, 0.15 mmol); 2 x reaction of 0.1 mmol scale. The crude product was purified by flash column chromatography (60/40 to 40/60 Hexane/Et_2O) to provide the title compound as a white solid (32 mg, 64% yield). ^{1}H NMR (300 MHz, $CDCl_3$) δ: 8.48 (bs, 2H), 7.33 – 7.24 (m, 2H), 7.23 – 7.13 (m, 3H), 7.09 (d, J = 5.8 Hz, 2H), 2.42 – 2.21 (m, 4H), 1.83 – 1.67 (m, 4H), 1.67 – 1.54 (m, 4H). ^{13}C NMR (75 MHz, $CDCl_3$) δ: 160.09, 149.74, 149.16, 128.39, 127.33, 126.01, 122.69, 50.15, 39.71, 30.11, 24.30. HRMS (APCI) found $[M+1]^+$ 252.1744, $C_{18}H_{22}N$ requires 252.1747.

4-(2-phenylbicyclo[2.2.1]heptan-2-yl)pyridine (30)

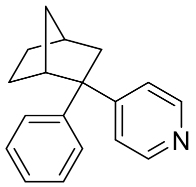

Prepared according to general procedure G using 2-phenylbicyclo[2.2.1]heptan-2-yl 4-cyano-2-fluorobenzoate **S20** (33.5 mg, 0.1 mmol) and 4-cyanopyridine (15.6 mg, 0.15 mmol); 2 x reaction of 0.1 mmol scale. The crude product was purified by flash column chromatography (60/40 to 40/60 Hexane/Et_2O) to provide the title compound as a white solid (38 mg, 76% yield). ^{1}H NMR (300 MHz, $CDCl_3$) δ: 8.45 (bs, 2H), 7.34 – 7.19 (m, 6H), 7.19 – 7.09 (m, 1H), 3.21 (s, 1H), 2.47 – 2.32 (m, 2H), 2.26 (ddd, *J* = 12.9, 4.5, 1.6 Hz, 1H), 1.61 (d, *J* = 9.8 Hz, 1H), 1.57 – 1.48 (m, 2H), 1.48 – 1.38 (m, 1H), 1.30 – 1.15 (m, 2H). ^{13}C NMR (75 MHz, $CDCl_3$) δ: 161.26, 149.75, 146.70, 128.37, 128.02, 125.92, 122.48, 56.33, 44.22, 43.01, 38.32, 38.12, 29.53, 24.60. HRMS (APCI) found $[M+1]^+$ 250.1592, $C_{18}H_{20}N$ requires 250.1590.

4-(4-phenyltetrahydro-2H-pyran-4-yl)pyridine (31)

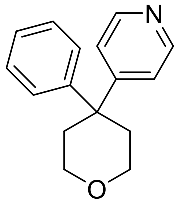

Prepared according to general procedure G using 4-phenyltetrahydro-2H-pyran-4-yl 4-cyano-2-fluorobenzoate **S21** (32.5 mg, 0.1 mmol) and 4-cyanopyridine (15.6 mg, 0.15 mmol); 2 x reaction of 0.1 mmol scale. The crude product was purified by flash column chromatography (20/80 to 5/95 Hexane/Et_2O) to provide the title compound as a white solid (40 mg, 84% yield). ^{1}H NMR (300 MHz, $CDCl_3$) δ: 8.53 (bs, 2H), 7.40 – 7.10 (m, 7H), 3.89 – 3.65 (m, 4H), 2.56 – 2.36 (m, 4H). ^{13}C NMR (75 MHz, $CDCl_3$) δ: 156.60, 150.16, 145.19, 128.94, 127.06, 126.67, 122.29, 64.58, 44.20, 36.21. HRMS (APCI) found $[M+1]^+$ 240.1383, $C_{16}H_{18}NO$ requires 240.1383.

***tert*-butyl 4-phenyl-4-(pyridin-4-yl)piperidine-1-carboxylate (32)**

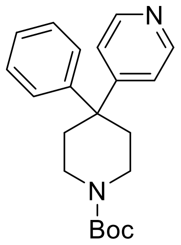

Prepared according to general procedure G using *tert*-butyl 4-((4-cyano-2-fluorobenzoyl)oxy)-4-phenylpiperidine-1-carboxylate **S22** (42.5 mg, 0.1 mmol) and 4-cyanopyridine (15.6 mg, 0.15 mmol); 2 x reaction of 0.1 mmol scale. The crude product was purified by flash column chromatography (30/70 to 10/90 Hexane/Et_2O) to provide the title compound as a viscous oil (47 mg, 70% yield). ^1H NMR (300 MHz, $CDCl_3$) δ: 8.50 (bs, 2H), 7.37 – 7.28 (m, 2H), 7.28 – 7.17 (m, 3H), 7.14 (d, J = 6.1 Hz, 2H), 3.62 – 3.35 (m, 4H), 2.47 – 2.25 (m, 4H), 1.45 (s, 9H). ^{13}C NMR (75 MHz, $CDCl_3$) δ: 156.27, 154.94, 150.17, 144.55, 128.95, 127.13, 126.73, 122.24, 79.71, 44.86, 40.68, 35.37, 28.54. HRMS (APCI) found $[M+1]^+$ 339.2054, $C_{21}H_{27}N_2O_2$ requires 339.2067.

***tert*-butyl 3-phenyl-3-(pyridin-4-yl)azetidine-1-carboxylate (33)**

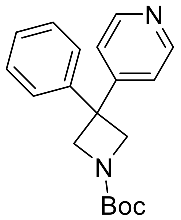

Prepared according to general procedure G using *tert*-butyl 3-((4-cyano-2-fluorobenzoyl)oxy)-3-phenylazetidine-1-carboxylate **S23** (40 mg, 0.1 mmol) and 4-cyanopyridine (15.6 mg, 0.15 mmol); 2 x reaction of 0.1 mmol scale. The crude product was purified by flash column chromatography (30/70 to 10/90 Hexane/Et_2O) to provide the title compound as a viscous oil (34 mg, 55% yield). ^1H NMR (300 MHz, $CDCl_3$) δ: 8.55 (d, *J* = 6.1 Hz, 2H), 7.42 – 7.33 (m, 2H), 7.33 – 7.25 (m, 1H), 7.21 – 7.11 (m, 4H), 4.57 (d, *J* = 8.4 Hz, 2H), 4.45 (d, *J* = 8.4 Hz, 2H), 1.46 (s, 9H). ^{13}C NMR (75 MHz, $CDCl_3$) δ: 156.35, 155.24, 150.25, 144.05, 129.04, 127.39, 126.72, 121.67, 80.28, 62.41, 45.24, 28.49. HRMS (APCI) found $[M+1]^+$ 311.1758,

$C_{19}H_{23}N_2O_2$ requires 311.1754.

4-(1-(2-(1,3-dioxan-2-yl)ethyl)-1,2,3,4-tetrahydronaphthalen-1-yl)pyridine (34)

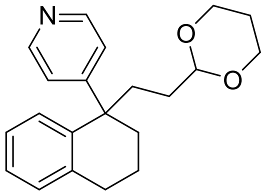

Prepared according to general procedure G using 1-(2-(1,3-dioxan-2-yl)ethyl)-1,2,3,4-tetrahydronaphthalen-1-yl 4-cyano-2-fluoro-benzoate **S24** (41 mg, 0.1 mmol) and 4-cyanopyridine (15.6 mg, 0.15 mmol); 2 x reaction of 0.1 mmol scale. The crude product was purified by flash column chromatography (40/60 to 10/90 Hexane/Et_2O) to provide the title compound as a viscous oil (46 mg, 71% yield). ^{1}H NMR (300 MHz, $CDCl_3$) δ: 8.42 (d, *J* = 5.3 Hz, 2H), 7.20 – 7.05 (m, 3H), 7.03 – 6.92 (m, 3H), 4.49 (t, *J* = 5.0 Hz, 1H), 4.15 – 4.00 (m, 2H), 3.80 – 3.64 (m, 2H), 2.82 – 2.71 (m, 2H), 2.26 – 2.15 (m, 2H), 2.15 – 1.98 (m, 2H), 1.93 (ddd, *J* = 13.5, 5.7, 3.2 Hz, 1H), 1.80 – 1.66 (m, 1H), 1.64 – 1.36 (m, 3H), 1.36 – 1.26 (m, 1H). ^{13}C NMR (75 MHz, $CDCl_3$) δ: 160.17, 149.49, 139.36, 138.43, 129.56, 129.10, 126.45, 126.12, 123.04, 102.51, 67.04, 67.02, 46.03, 36.55, 34.59, 30.99, 30.10, 25.91, 19.11. HRMS (APCI) found $[M+1]^+$ 324.1956, $C_{21}H_{26}NO_2$ requires 324.1958.

4-(2-(5-chlorothiophen-2-yl)-4-phenylbutan-2-yl)pyridine (35)

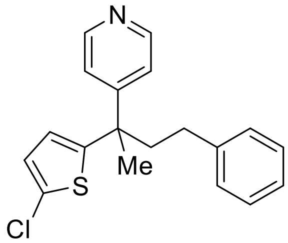

Prepared according to general procedure G using 2-(5-chlorothiophen-2-yl)-4-phenylbutan-2-yl 4-cyano-2-fluorobenzoate **S25** (41.5 mg, 0.1 mmol) and 4-cyanopyridine (15.6 mg, 0.15 mmol); 2 x reaction of 0.1 mmol scale. The crude product was purified by flash column chromatography (60/40 to 40/60 Hexane/Et_2O) to provide the title compound as a viscous oil (43 mg, 66% yield). ^{1}H NMR (300 MHz, $CDCl_3$) δ: 8.57 (d, *J* = 5.5 Hz, 2H), 7.34 – 7.27 (m, 2H), 7.26 – 7.18 (m, 3H), 7.18 – 7.11 (m, 2H), 6.79 (d, *J* = 3.8 Hz, 1H), 6.66 (d, *J* = 3.8 Hz, 1H), 2.63 –

2.26 (m, 4H), 1.79 (s, 3H). ^{13}C NMR (75 MHz, $CDCl_3$) δ: 156.71, 151.50, 150.09, 141.69, 128.87, 128.69, 128.39, 126.25, 125.64, 123.89, 121.85, 45.46, 44.57, 31.16, 27.36. HRMS (APCI) found $[M+1]^+$ 328.0921, $C_{19}H_{19}ClNS$ requires 328.0921.

4-benzylpyridine (36)

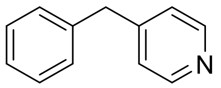

Prepared according to general procedure G using benzyl 4-cyano-2-fluorobenzoate **S26** (25.5 mg, 0.1 mmol) and 4-cyanopyridine (15.6 mg, 0.15 mmol); 2 x reaction of 0.1 mmol scale. The crude product was purified by flash column chromatography (50/50 to 30/70 Hexane/Et_2O) to provide the title compound as a colorless viscous oil (21 mg, 62% yield). ^{1}H NMR (300 MHz, $CDCl_3$) δ: 8.50 (d, J = 5.8 Hz, 2H), 7.32 (t, J = 7.1 Hz, 2H), 7.28 – 7.21 (m, 1H), 7.18 (d, J = 7.0 Hz, 2H), 7.10 (d, J = 5.5 Hz, 2H), 3.97 (s, 2H). ^{13}C NMR (125 MHz, $CDCl_3$) δ: 150.14, 149.95, 138.99, 129.17, 128.86, 126.81, 124.35, 41.38. HRMS (APCI) found $[M+1]^+$ 170.0966, $C_{12}H_{12}N$ requires 170.0964.

4-(4-methoxybenzyl)pyridine (37)

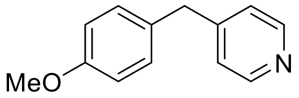

Prepared according to general procedure G using 4-methoxybenzyl 4-cyano-2-fluorobenzoate **S27** (28.5 mg, 0.1 mmol) and 4-cyanopyridine (15.6 mg, 0.15 mmol); 2 x reaction of 0.1 mmol scale. The crude product was purified by flash column chromatography (50/50 to 30/70 Hexane/Et_2O) to provide the title compound as a yellowish viscous oil (24 mg, 60% yield). ^{1}H NMR (500 MHz, $CDCl_3$) δ: 8.48 (d, J = 5.8 Hz, 2H), 7.11 – 7.05 (m, 4H), 6.88 – 6.83 (m, 2H), 3.90 (s, 3H), 3.79 (s, 4H). ^{13}C NMR (125 MHz, $CDCl_3$) δ: 158.52, 150.60, 149.96, 131.07, 130.15, 124.20, 114.26, 55.40, 40.48. HRMS (APCI) found $[M+1]^+$ 200.1068, $C_{13}H_{14}NO$ requires 200.1070.

***tert*-butyl (3-(pyridin-4-ylmethyl)phenyl)carbamate (38)**

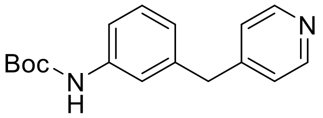

Prepared according to general procedure G using 3-((*tert*-butoxycarbonyl)amino)benzyl

4-cyano-2-fluorobenzoate **S28** (37 mg, 0.1 mmol) and 4-cyanopyridine (15.6 mg, 0.15 mmol); 2 x reaction of 0.1 mmol scale. The crude product was purified by flash column chromatography (50/50 to 30/70 Hexane/Et_2O) to provide the title compound as a yellowish viscous oil (35 mg, 62% yield). ^{1}H NMR (300 MHz, $CDCl_3$) δ: 8.53 (bs, 2H), 7.35 – 7.08 (m, 4H), 6.87 (d, *J* = 6.5 Hz, 1H), 6.61 (s, 1H), 3.97 (s, 2H), 1.54 (s, 9H). ^{13}C NMR (75 MHz, $CDCl_3$) δ: 152.84, 150.12, 149.83, 139.94, 138.98, 129.44, 124.40, 123.81, 119.23, 116.97, 80.73, 41.39, 28.48. HRMS (APCI) found $[M+1]^+$ 285.1601, $C_{17}H_{21}N_2O_2$ requires 285.1598.

***tert*-butyl 3-(pyridin-4-ylmethyl)-1H-indole-1-carboxylate (39)**

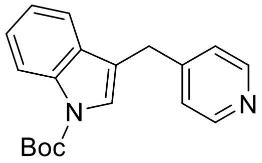

Prepared according to general procedure G using tert-butyl 3-(((4-cyano-2-fluorobenzoyl)oxy)methyl)-1H-indole-1-carboxylate **S29** (39.5 mg, 0.1 mmol) and 4-cyanopyridine (15.6 mg, 0.15 mmol); 2 x reaction of 0.1 mmol scale. The crude product was purified by flash column chromatography (50/50 to 30/70 Hexane/Et_2O) to provide the title compound as a yellowish viscous oil (39 mg, 63% yield). ^{1}H NMR (300 MHz, $CDCl_3$) δ: 8.50 (bs, 2H), 8.13 (d, *J* = 8.1 Hz, 1H), 7.40 (s, 1H), 7.37 – 7.28 (m, 2H), 7.23 – 7.15 (m, 3H), 4.04 (s, 2H), 1.67 (s, 9H). ^{13}C NMR (75 MHz, $CDCl_3$) δ: 149.95, 149.07, 130.21, 124.78, 124.16, 123.62, 122.74, 119.22, 117.93, 115.54, 108.55, 83.93, 30.96, 28.37. HRMS (APCI) found $[M+1]^+$ 309.1609, $C_{19}H_{21}N_2O_2$ requires 309.1598.

4-(4-(4-chlorophenyl)-4-(pyridin-4-yl)piperidin-1-yl)-1-(4-fluorophenyl)butan-1-one (from Haloperidol) (40)

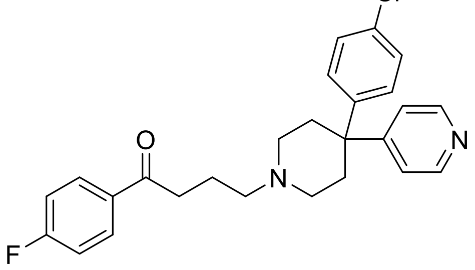

Prepared according to general procedure G using 4-(4-chlorophenyl)-1-(4-(4-fluorophenyl)-4-oxobutyl)piperidin-4-yl 4-cyano-2-fluoro benzoate **S30** (Haloperidol ester, 52.3 mg, 0.1 mmol) and 4-cyanopyridine (15.6 mg, 0.15 mmol); 2 x reaction of 0.1 mmol scale. The crude product was purified by flash column chromatography (98/1/1 to 95/4/1 AcOEt/MeOH/Et_3N) to provide the title compound as a yellowish solid (64 mg, 73% yield). ^{1}H NMR (300 MHz, $CDCl_3$) δ: 8.49 (d, *J* = 6.0 Hz, 2H), 8.05 – 7.93 (m, 2H), 7.31 – 7.23 (m, 2H), 7.18 – 7.07 (m, 6H), 2.95 (t, *J* = 7.0 Hz, 2H), 2.57 – 2.42 (m, 4H), 2.42 – 2.27 (m, 6H), 1.92 (p, *J* = 7.0 Hz, 2H). ^{13}C NMR (75 MHz, $CDCl_3$) δ: 198.40, 165.76 (d, *J* = 254.5 Hz), 156.40, 150.17, 150.04, 133.83 (d, *J* = 3.1 Hz), 132.34, 130.77 (d, *J* = 9.2 Hz), 128.89, 128.72, 122.42, 115.73 (d, *J* = 21.8 Hz), 57.84, 50.21, 44.47, 36.24, 35.62, 21.95. HRMS (APCI) found $[M+1]^+$ 437.1775, $C_{26}H_{27}ClFN_2O$ requires 437.1790.

4-(4-(benzyloxy)phenyl)-1-(4-fluorophenyl)-3-(3-(4-fluorophenyl)-3-(pyridin-4-yl)propyl)azetidin-2-one (from OBn-Ezetimibe) (41)

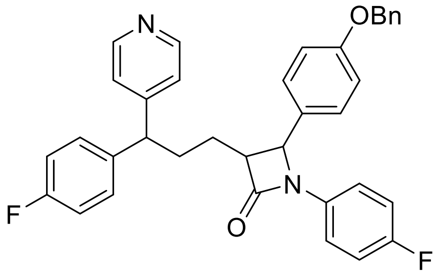

Prepared according to general procedure G using 3-(2-(4-(benzyloxy)phenyl)-1-(4-fluorophenyl)-4-oxoazetidin-3-yl)-1-(4-fluorophenyl)propyl 4-cyano-2-fluorobenzoate **S31** (OBn-Ezetimibe ester, 64.7 mg, 0.1 mmol) and 4-cyanopyridine (15.6 mg, 0.15 mmol); 2 x reaction of 0.1 mmol scale. The crude product was purified by flash column chromatography (30/70 to 10/90 Hex/Et_2O) to provide the title compound as a yellowish solid (81 mg, 72% yield). ^{1}H NMR (300 MHz, $CDCl_3$) δ: 8.61 (bs, 2H), 7.46 – 7.29 (m, 5H), 7.28 – 7.11 (m, 8H), 7.06 – 6.86 (m, 6H), 5.06 (s, 2H), 4.51 (t, *J* = 2.6 Hz, 1H), 3.94 (td, *J* = 7.9, 3.0 Hz, 1H), 3.16 – 3.02 (m, 1H), 2.43 – 2.24 (m, 1H), 2.24 – 2.09 (m, 1H), 1.96 – 1.71 (m, 2H). ^{13}C NMR (75 MHz, $CDCl_3$) δ: 167.14, 162.17 (d, *J* = 213.8 Hz), 159.29, 158.92 (d, *J* = 211.0 Hz), 154.96, 148.64, 137.63 (d, *J* = 3.4 Hz), 136.74, 133.96 (d, *J* = 2.5 Hz), 129.62, 129.44 (d, *J* =

7.8 Hz), 128.77, 128.25, 127.58, 127.30, 123.66, 118.51 (d, *J* = 7.8 Hz), 115.99 (d, *J* = 19.4 Hz), 115.97 (d, *J* = 19.4 Hz), 115.78, 70.27, 61.06, 60.38, 50.09, 32.45, 30.46, 27.29. HRMS (APCI) found $[M+1]^+$ 561.2344, $C_{36}H_{31}F_2N_2O_2$ requires 561.2348.

4-(pent-3-en-2-yl)pyridine (42)

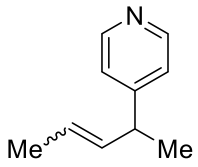

Prepared according to general procedure G using pent-3-en-2-yl 4-cyano-2-fluorobenzoate **S32** (predominantly *trans*, 23.3 mg, 0.1 mmol) and 4-cyanopyridine (15.6 mg, 0.15 mmol); 2 x reaction of 0.1 mmol scale. The crude product (*trans:cis* ratio 4:1) was purified by flash column chromatography (50/50 to 30/70 Hexane/Et_2O) to provide the title compound as a colorless oil (*trans:cis* ratio 5.4:1, 15 mg, 51% yield). ^1H NMR (300 MHz, $CDCl_3$) δ: 8.50 (d, *J* = 5.5 Hz, 2H), 7.16 (d, *J* = 5.5 Hz, 2H), 5.62 – 5.40 (m, 2H), 3.47 – 3.34 (m, 1H), 1.68 (d, *J* = 6.0 Hz, 3H), 1.33 (d, *J* = 7.0 Hz, 3H). ^{13}C NMR (75 MHz, $CDCl_3$) δ: 155.97, 149.40, 134.31, 125.61, 122.91, 41.96, 20.84, 18.01.

3-methyl-3-(pyridin-4-yl)butan-2-one (43)

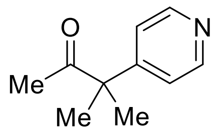

Prepared according to general procedure G using 2-methyl-3-oxobutan-2-yl 4-cyano-2-fluorobenzoate **S33** (75 mg, 0.3 mmol) and 4-pyridinecarbonitrile (10.4 mg, 0.1 mmol), Hantzsch ester (101.2 mg, 0.4 mmol) and NaOAc (32.8 mg, 0.4 mmol); 2 x reaction of 0.1 mmol scale. Reaction time: 20 hours. The crude product was purified by flash column chromatography (30/70 to 20/80 hexane/Et_2O) to provide the title compound as a pale yellow liquid (22 mg, 67% yield). ^1H NMR (300 MHz, $CDCl_3$) δ 8.66 – 8.49 (m, 2H), 7.24 – 7.11 (m, 2H), 1.95 (s, 3H), 1.49 (s, 6H). ^{13}C NMR (75 MHz, $CDCl_3$) δ 209.38, 153.17, 150.44, 121.38, 52.49, 25.80, 24.72. HRMS (APCI) $[M+H]^+$ found 164.1071, $C_{10}H_{14}NO$ requires 164.1070.

2-methyl-4-(4-phenyltetrahydro-2H-pyran-4-yl)pyridine (44)

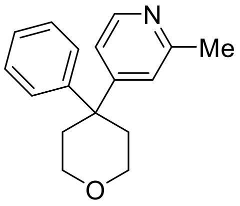

Prepared according to general procedure G using 4-phenyltetrahydro-2H-pyran-4-yl 4-cyano-2-fluorobenzoate **S21** (33 mg, 0.1 mmol) and 4-cyano-2-methylpyridine (17.7 mg, 0.15 mmol); 2 x reaction of 0.1 mmol scale. The crude product was purified by flash column chromatography (10/90 to 5/95 Hexane/Et_2O) to provide the title compound as a white solid (40 mg, 79% yield). ^{1}H NMR (300 MHz, $CDCl_3$) δ 8.40 (d, J = 5.4 Hz, 1H), 7.40 – 7.18 (m, 5H), 7.04 (s, 1H), 6.99 (d, J = 5.4 Hz, 1H), 3.85 – 3.67 (m, 4H), 2.53 (s, 3H), 2.50 – 2.36 (m, 4H). ^{13}C NMR (75 MHz, $CDCl_3$) δ 158.76, 156.74, 149.50, 145.44, 128.89, 126.99, 126.57, 121.62, 119.39, 64.62, 44.03, 36.19, 24.82. HRMS (APCI) $[M+H]^+$ found 254.1539, $C_{17}H_{20}NO$ requires 254.1539.

4-(4-phenyltetrahydro-2H-pyran-4-yl)-2-propylpyridine (45)

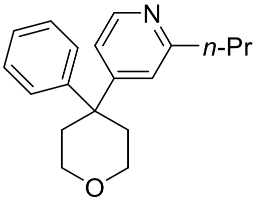

Prepared according to general procedure G using 4-phenyltetrahydro-2H-pyran-4-yl 4-cyano-2-fluorobenzoate **S21** (33 mg, 0.1 mmol) and 2-propylisonicotinonitrile (22 mg, 0.15 mmol); 2 x reaction of 0.1 mmol scale. The crude product was purified by flash column chromatography (50/50 to 45/55 Hexane/Et_2O) to provide the title compound as a colorless oil (42 mg, 75% yield). ^{1}H NMR (300 MHz, $CDCl_3$) δ 8.43 (d, J = 5.3 Hz, 1H), 7.39 – 7.17 (m, 5H), 7.03 (s, 1H), 6.97 (d, J = 5.3 Hz, 1H), 3.87 – 3.57 (m, 4H), 2.73 (t, J = 7.4 Hz, 2H), 2.54 – 2.30 (m, 4H), 1.86 – 1.63 (m, 2H), 0.95 (t, J = 7.3 Hz, 3H). ^{13}C NMR (75 MHz, $CDCl_3$) δ 162.68, 156.67, 149.58, 145.55, 128.88, 127.03, 126.57, 121.16, 119.65, 64.66, 44.17, 40.70, 36.32, 23.25, 13.93. HRMS (APCI) $[M+H]^+$ found 282.1851, $C_{19}H_{24}NO$ requires 282.1852.

(4-(4-phenyltetrahydro-2H-pyran-4-yl)pyridin-2-yl)methanol (46)

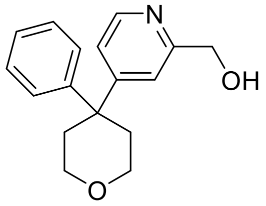

Prepared according to general procedure G using 4-phenyltetrahydro-2H-pyran-4-yl 4-cyano-2-fluorobenzoate **S21** (33 mg, 0.1 mmol) and 2-(hydroxymethyl)isonicotinonitrile (20.1 mg, 0.15 mmol); 2 x reaction of 0.1 mmol scale. The crude product was purified by flash column chromatography (01/99 to 0/100 Hexane/Et_2O) to provide the title compound as a pale yellow solid (40 mg, 74% yield). ^{1}H NMR (300 MHz, $CDCl_3$) δ 8.46 (d, *J* = 5.3 Hz, 1H), 7.38 – 7.19 (m, 5H), 7.14 (s, 1H), 7.09 (d, *J* = 5.3 Hz, 1H), 4.72 (s, 2H), 3.83 – 3.69 (m, 4H), 2.55 – 2.34 (m, 4H). ^{13}C NMR (75 MHz, $CDCl_3$) δ 159.63, 157.65, 148.95, 145.06, 129.02, 127.07, 126.75, 121.08, 118.78, 64.61, 64.53, 44.29, 36.27. HRMS (APCI) $[M+H]^+$ found 270.1487, $C_{17}H_{20}NO_2$ requires 270.1489.

methyl 3-((4-(4-phenyltetrahydro-2*H*-pyran-4-yl)pyridin-2-yl)methoxy)propanoate (47)

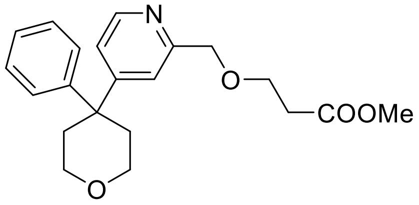

Prepared according to general procedure G using 4-phenyltetrahydro-2*H*-pyran-4-yl 4-cyano-2-fluorobenzoate **S21** (32.5 mg, 0.1 mmol) and methyl 3-((4-cyanopyridin-2-yl)methoxy)propanoate **S34** (33 mg, 0.15 mmol); 2 x reaction of 0.1 mmol scale. The crude product was purified by flash column chromatography (20/80 to 5/95 Hexane/Et_2O) to provide the title compound as a colorless viscous oil (40 mg, 56% yield). ^{1}H NMR (300 MHz, $CDCl_3$) δ: 8.43 (d, *J* = 5.3 Hz, 1H), 7.39 (d, *J* = 1.3 Hz, 1H), 7.36 – 7.24 (m, 4H), 7.24 – 7.16 (m, 1H), 7.04 (dd, *J* = 5.3, 1.8 Hz, 1H), 4.63 (s, 2H), 3.81 (t, *J* = 6.3 Hz, 2H), 3.78 – 3.72 (m, 4H), 3.71 (s, 3H), 2.65 (t, *J* = 6.3

Hz, 2H), 2.53 – 2.40 (m, 4H). ^{13}C NMR (75 MHz, $CDCl_3$) δ: 172.02, 158.62, 157.72, 149.23, 145.29, 128.92, 127.04, 126.62, 121.13, 119.59, 73.88, 66.39, 64.63, 51.81, 44.32, 36.18, 34.99. HRMS (APCI) found $[M+1]^+$ 356.1860, $C_{21}H_{26}NO_4$ requires 356.1856.

2-phenyl-4-(4-phenyltetrahydro-2H-pyran-4-yl)pyridine (48)

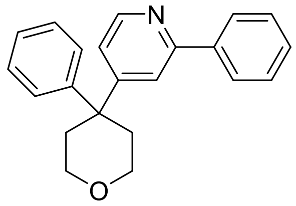

Prepared according to general procedure G using 4-phenyltetrahydro-2H-pyran-4-yl 4-cyano-2-fluorobenzoate **S21** (33 mg, 0.1 mmol) and 2-phenylisonicotinonitrile (27 mg, 0.15 mmol), Hantzsch ester (63.3 mg, 0.25 mmol) and NaOAc (20.5 mg, 0.25 mmol); 2 x reaction of 0.1 mmol scale. Reaction time: 20 hours. The crude product was purified by flash column chromatography (70/30 to 60/40 of hexane/Et_2O) to provide the title compound albeit with some unknown impurities, therefore it is reported considering the NMR yield (31% yield). 1,1,2,2-tetrachloroethane was used as the internal standard. ^{1}H NMR (300 MHz, $CDCl_3$) δ 8.59 (d, J = 5.3 Hz, 1H), 7.90 (d, J = 7.4 Hz, 2H), 7.58 (s, 1H), 7.53 – 7.17 (m, 8H), 7.10 (d, J = 5.3 Hz, 1H), 3.79 (t, J = 5.2 Hz, 4H), 2.50 (m, 4H).

2,6-dimethyl-4-(4-phenyltetrahydro-2H-pyran-4-yl)pyridine (49)

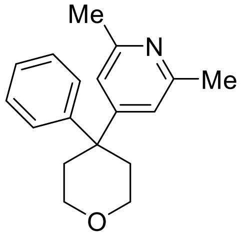

Prepared according to general procedure G using 4-phenyltetrahydro-2H-pyran-4-yl 4-cyano-2-fluorobenzoate **S21** (33 mg, 0.1 mmol) and 2,6-dimethylisonicotinonitrile (19.8 mg, 0.15 mmol); 2 x reaction of 0.1 mmol scale. The crude product was purified by flash column chromatography (25/75 to 20/80 Hexane/Et_2O) to provide the title compound as a pale yellow solid (40 mg, 78% yield). ^{1}H NMR (300 MHz, $CDCl_3$) δ 7.39 – 7.17 (m, 5H), 6.85 (s, 2H), 3.83 – 3.69 (m, 4H), 2.49 (s, 6H), 2.46 – 2.39 (m, 4H).

^{13}C NMR (75 MHz, $CDCl_3$) δ 158.08, 156.97, 145.77, 128.86, 126.99, 126.51, 118.78, 64.70, 43.99, 36.32, 24.83. HRMS (APCI) $[M+H]^+$ found 268.1696, $C_{18}H_{22}NO$ requires 268.1696.

3-methyl-4-(4-phenyltetrahydro-2H-pyran-4-yl)pyridine (50)

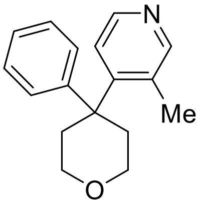

Prepared according to general procedure G using 4-phenyltetrahydro-2H-pyran-4-yl 4-cyano-2-fluorobenzoate **S21** (33 mg, 0.1 mmol) and 4-cyano-3-methylpyridine (17.7 mg, 0.15 mmol); 2 x reaction of 0.1 mmol scale. The crude product was purified by flash column chromatography (10/90 to 5/95 Hexane/Et_2O) to provide the title compound as a colorless oil (42 mg, 83% yield). ^1H NMR (300 MHz, $CDCl_3$) δ 8.50 (d, J = 5.3 Hz, 1H), 8.25 (s, 1H), 7.48 (d, J = 5.3 Hz, 1H), 7.35 – 7.13 (m, 5H), 3.94 – 3.65 (m, 4H), 2.44 (t, J = 5.3 Hz, 4H), 1.90 (s, 3H). ^{13}C NMR (75 MHz, $CDCl_3$) δ 153.81, 153.34, 147.59, 145.89, 132.61, 128.50, 127.43, 126.37, 122.40, 64.67, 45.05, 36.31, 18.85. HRMS (APCI) [M+H]+ found 254.1540, $C_{17}H_{20}NO$ requires 254.1539.

3-phenyl-4-(4-phenyltetrahydro-2H-pyran-4-yl)pyridine (51)

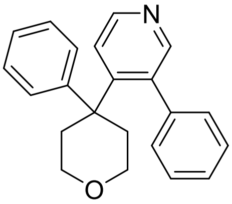

Prepared according to general procedure G using 4-phenyltetrahydro-2H-pyran-4-yl 4-cyano-2-fluorobenzoate **S21** (33 mg, 0.1 mmol), 3-phenylisonicotinonitrile **S35** (27 mg, 0.15 mmol), Hantzsch ester (63.3 mg, 0.25 mmol) and NaOAc (20.5 mg, 0.25 mmol); 2 x reaction of 0.1 mmol scale. Reaction time: 20 hours. The crude product was purified by flash column chromatography (50/50 to 40/60 Hexane/Et_2O) to provide the title compound as a white solid (32 mg, 51% yield). ^1H NMR (300 MHz, $CDCl_3$) δ 8.67 (d, J = 5.4 Hz, 1H), 8.22 (s, 1H), 7.60 (d, J = 5.4 Hz, 1H), 7.31 – 7.08 (m, 6H), 7.01 – 6.88 (m, 2H), 6.51 (d, J = 7.2 Hz, 2H), 3.78 – 3.58 (m, 4H), 2.36 – 2.10 (m, 4H). ^{13}C

NMR (75 MHz, $CDCl_3$) δ 153.46, 152.44, 148.93, 146.55, 139.56, 138.37, 129.82, 128.42, 127.70, 127.58, 127.41, 126.40, 122.63, 64.51, 45.33, 37.13. HRMS (APCI) $[M+H]^+$ found 316.1700, $C_{22}H_{22}NO$ requires 316.1696.

4-(4-phenyltetrahydro-2H-pyran-4-yl)-3-(p-tolyl)pyridine (52)

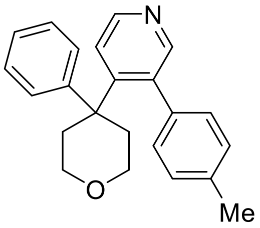

Prepared according to general procedure G using 4-phenyltetrahydro-2H-pyran-4-yl 4-cyano-2-fluorobenzoate **S21** (33 mg, 0.1 mmol), 3-(p-tolyl)isonicotinonitrile **S36** (29.1 mg, 0.15 mmol), Hantzsch ester (63.3 mg, 0.25 mmol) and NaOAc (20.5 mg, 0.25 mmol); 2 x reaction of 0.1 mmol scale. Reaction time: 20 hours. The crude product was purified by flash column chromatography (40/60 to 30/70 Hexane/Et_2O) to provide the title compound as a white solid (35.5 mg, 54% yield). ^1H NMR (300 MHz, $CDCl_3$) δ 8.64 (d, J = 5.5 Hz, 1H), 8.21 (s, 1H), 7.58 (d, J = 5.5 Hz, 1H), 7.26 – 7.15 (m, 3H), 7.04 – 6.90 (m, 4H), 6.39 (d, J = 8.1 Hz, 2H), 3.77 – 3.50 (m, 4H), 2.35 (s, 3H), 2.32 – 2.12 (m, 4H). ^{13}C NMR (75 MHz, $CDCl_3$) δ 153.69, 152.38, 148.75, 146.77, 138.39, 137.15, 136.65, 129.63, 128.40, 128.30, 127.66, 126.36, 122.73, 64.54, 45.38, 37.11, 21.30. HRMS (APCI) $[M+H]^+$ found 330.1857, $C_{23}H_{24}NO$ requires 330.1852.

3-(4-methoxyphenyl)-4-(4-phenyltetrahydro-2H-pyran-4-yl)pyridine (53)

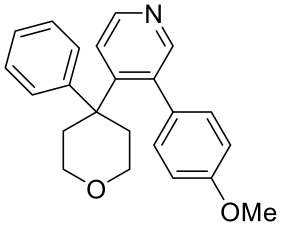

Prepared according to general procedure G using 4-phenyltetrahydro-2H-pyran-4-yl 4-cyano-2-fluorobenzoate **S21** (33 mg, 0.1 mmol) and 3-(4-methoxyphenyl)isonicotinonitrile **S37** (31.5 mg, 0.15 mmol), Hantzsch ester (63.3 mg, 0.25 mmol) and NaOAc (20.5 mg, 0.25 mmol); 2 x reaction of 0.1 mmol scale. Reaction time: 20 hours. The crude product was purified by flash column chromatography (30/70 to 20/80 Hexane/Et_2O) to provide the title compound as a white

solid (37 mg, 54% yield). ^{1}H NMR (300 MHz, $CDCl_3$) δ 8.64 (d, *J* = 5.4 Hz, 1H), 8.21 (s, 1H), 7.58 (d, *J* = 5.4 Hz, 1H), 7.31 – 7.13 (m, 3H), 7.02 – 6.91 (m, 2H), 6.72 – 6.61 (m, 2H), 6.45 – 6.34 (m, 2H), 3.82 (s, 3H), 3.76 – 3.56 (m, 4H), 2.35 – 2.11 (m, 4H). ^{13}C NMR (75 MHz, $CDCl_3$) δ 158.98, 153.89, 152.72, 148.78, 146.76, 138.11, 131.84, 130.89, 128.41, 127.69, 126.37, 122.68, 113.05, 64.53, 55.36, 45.33, 37.10. HRMS (APCI) $[M+H]^+$ found 346.1802, $C_{23}H_{24}NO_2$ requires 346.1802.

3-(4-fluorophenyl)-4-(4-phenyltetrahydro-2H-pyran-4-yl)pyridine (54)

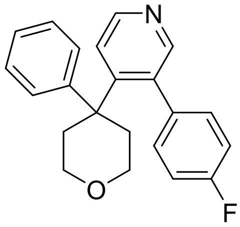

Prepared according to general procedure G using 4-phenyltetrahydro-2H-pyran-4-yl 4-cyano-2-fluorobenzoate **S21** (33 mg, 0.1 mmol) and 3-(4-fluorophenyl)isonicotinonitrile **S38** (29.7 mg, 0.15 mmol), Hantzsch ester (63.3 mg, 0.25 mmol), NaOAc (20.5 mg, 0.25 mmol); 2 x reaction of 0.1 mmol scale. Reaction time: 20 hours. The crude product was purified by flash column chromatography (30/70 to 20/80 Hexane/Et_2O) to provide the title compound as a white solid (27 mg, 40.5% yield). ^{1}H NMR (300 MHz, $CDCl_3$) δ 8.67 (d, *J* = 5.4 Hz, 1H), 8.18 (s, 1H), 7.60 (d, *J* = 5.4 Hz, 1H), 7.26 – 7.15 (m, 3H), 6.97 – 6.88 (m, 2H), 6.87 – 6.77 (m, 2H), 6.49 – 6.35 (m, 2H), 3.78 – 3.60 (m, 4H), 2.34 – 2.13 (m, 4H). ^{13}C NMR (75 MHz, $CDCl_3$) δ 162.07 (d, *J* = 247.2 Hz), 153.39, 152.87, 149.07, 146.19, 137.26, 135.20 (d, *J* = 3.7 Hz), 131.35 (d, *J* = 8.1 Hz), 128.35, 127.61, 126.38, 122.36, 114.40 (d, *J* = 21.4 Hz), 64.30, 45.10, 36.97. ^{19}F NMR (282 MHz, $CDCl_3$) δ -114.72. HRMS (APCI) $[M+H]^+$ found 334.1600, $C_{22}H_{21}FNO$ requires 334.1602.

4-(4-(4-(4-phenyltetrahydro-2H-pyran-4-yl)pyridin-3-yl)benzyl)morpholine (55)

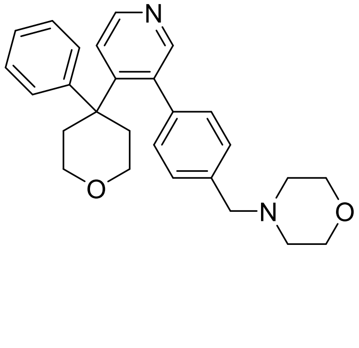

Prepared according to general procedure G using 4-phenyltetrahydro-2H-pyran-4-yl 4-cyano-2-fluorobenzoate **S21** (33 mg, 0.1 mmol) and 3-(4-(morpholinomethyl)phenyl)isonicotinonitrile **S39** (42 mg, 0.15 mmol), Hantzsch ester (63.3 mg, 0.25 mmol) and NaOAc (20.5 mg, 0.25 mmol); 2 x reaction of 0.1 mmol scale. Reaction time: 20 hours. The crude product was purified by flash column chromatography (2% NEt_3 in Et_2O) to provide the title compound albeit with some unknown impurities, therefore it is reported as an NMR yield (43% NMR yield). 1,1,2,2-tetrachloroethane was used as the internal standard. ^{1}H NMR (300 MHz, $CDCl_3$) δ 8.63 (d, *J* = 5.4 Hz, 1H), 8.18 (s, 1H), 7.57 (d, *J* = 5.4 Hz, 1H), 7.19 – 7.12 (m, 3H), 7.06 (d, *J* = 7.7 Hz, 2H), 6.95 – 6.86 (m, 2H), 6.43 (d, *J* = 7.6 Hz, 2H), 3.83 – 3.67 (m, 4H), 3.67 – 3.56 (m, 4H), 3.48 (s, 2H), 2.55 – 2.36 (m, 4H), 2.27 – 2.13 (m, 4H). ^{13}C NMR (75 MHz, $CDCl_3$) δ 153.53, 152.54, 148.91, 146.64, 138.42, 138.25, 137.02, 129.75, 128.40, 128.29, 127.70, 126.42, 122.65, 67.17, 64.52, 63.20, 53.78, 45.36, 37.16. HRMS (APCI) $[M+H]^+$ found 415.2379, $C_{27}H_{31}N_2O_2$ requires 415.2380.

4-(4-phenyltetrahydro-2H-pyran-4-yl)-3-(m-tolyl)pyridine (56)

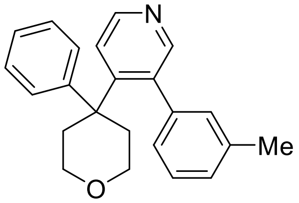

Prepared according to general procedure G using 4-phenyltetrahydro-2H-pyran-4-yl 4-cyano-2-fluorobenzoate **S21** (33 mg, 0.1 mmol) and 3-(m-tolyl)isonicotinonitrile **S40** (29.1 mg, 0.15 mmol), Hantzsch ester (63.3 mg, 0.25 mmol) and NaOAc (20.5 mg, 0.25 mmol); 2 x reaction of 0.1 mmol scale. Reaction time: 20 hours. The crude product was purified by flash column chromatography (30/70 to 20/80 Hexane/Et_2O) to provide the title compound as a white solid (29 mg, 44% yield). ^{1}H NMR (300 MHz, $CDCl_3$) δ 8.66 (d, *J* = 5.4 Hz, 1H), 8.22 (s, 1H), 7.59 (d, *J* = 5.4 Hz, 1H), 7.27 – 7.15 (m, 3H), 7.11 – 7.03 (m, 2H), 7.01 – 6.92 (m, 2H), 6.57 – 6.47 (m, 1H), 6.03 (s, 1H), 3.76 – 3.53 (m, 4H), 2.39 – 2.15 (m, 4H), 2.13 (s, 3H). ^{13}C NMR (75 MHz, $CDCl_3$) δ 153.45, 152.30, 148.81, 146.88, 139.37, 138.48, 137.01, 130.80, 128.34, 128.19, 127.67, 127.52, 126.66, 126.29, 122.63, 64.53, 45.27, 36.03, 21.44. HRMS (APCI) $[M+H]^+$ found 330.1852,

$C_{23}H_{24}NO$ requires 330.1852.

3-(3-methoxyphenyl)-4-(4-phenyltetrahydro-2H-pyran-4-yl)pyridine (57)

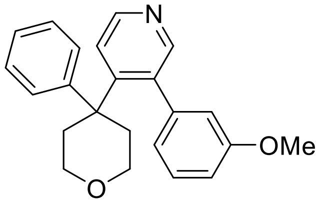

Prepared according to general procedure G using 4-phenyltetrahydro-2H-pyran-4-yl 4-cyano-2-fluorobenzoate **S21** (33 mg, 0.1 mmol) and 3-(3-methoxyphenyl)isonicotinonitrile **S41** (31.5 mg, 0.15 mmol), Hantzsch ester (63.3 mg, 0.25 mmol) and NaOAc (20.5 mg, 0.25 mmol); 2 x reaction of 0.1 mmol scale. Reaction time: 20 hours. The crude product was purified by flash column chromatography (30/70 to 20/80 Hexane/Et_2O) to provide the title compound as a white solid (32 mg, 46% yield). ^{1}H NMR (300 MHz, $CDCl_3$) δ 8.66 (d, *J* = 5.4 Hz, 1H), 8.24 (s, 1H), 7.59 (d, *J* = 5.4 Hz, 1H), 7.27 – 7.14 (m, 3H), 7.09 (t, *J* = 7.9 Hz, 1H), 7.02 – 6.94 (m, 2H), 6.85 – 6.76 (m, 1H), 6.31 – 6.25 (m, 1H), 5.99 – 5.90 (m, 1H), 3.75 – 3.58 (m, 4H), 3.54 (s, 3H), 2.40 – 2.09 (m, 4H). ^{13}C NMR (75 MHz, $CDCl_3$) δ 158.62, 153.24, 151.93, 148.80, 146.65, 140.77, 138.07, 128.54, 128.33, 127.47, 126.25, 122.66, 122.04, 114.79, 113.69, 64.40, 54.89, 45.37, 36.52. HRMS (APCI) $[M+H]^+$ found 346.1802, $C_{23}H_{24}NO_2$ requires 346.1802.

4. Mechanistic experiments

UV-Vis absorption studies

The UV-Vis absorption spectra were recorded using a Jasco V-770 spectrophotometer equipped with photomultiplier detector, monochromator and deuterium/halogen light source. The samples were prepared in a 1mL quartz cuvette with a path length of 1 cm. Final concentration of the single components: HE (0.02 M), ester **1** (0.01 M), PyCN (0.015 M).

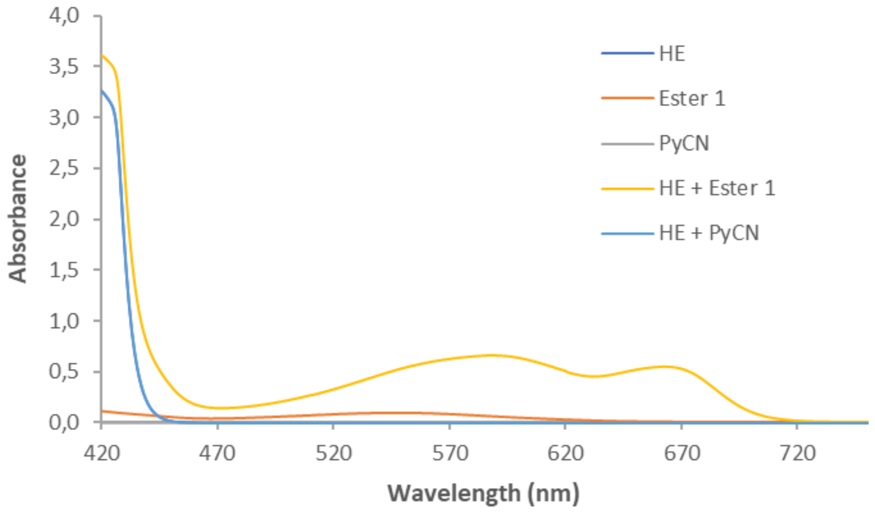

The combination of HE and dinitro ester **1** showed a new absorption band in the visible region (yellow line), indicating the formation of a charge transfer complex. On the other hand, the mixture of HE and PyCN did not shown any difference from the absorption spectra of the single components, excluding a ground-state association between these two species.

We performed the same studies with benzoate **3**; final concentration of the single components: HE (0.02 M), ester **3** (0.01 M).

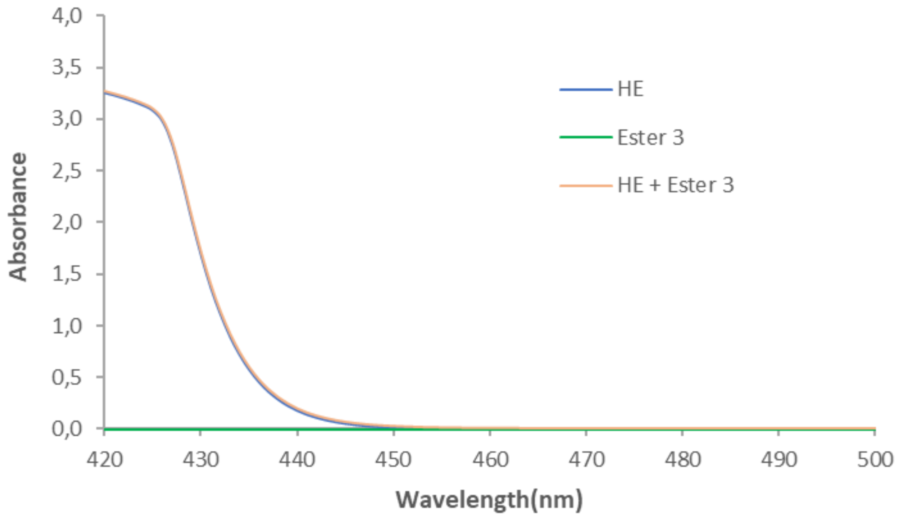

In this case, no appreciable difference in the absorption spectra was observed when HE and ester **3** were mixed, excluding a ground-state association between these two species. We then carefully studied the changing in the absorption spectra of HE ($1.0 \cdot 10^{-4}$ M) in the presence of NaOAc. The sample was prepared in a vial and 2.5 mg of NaOAc were added (0.03 mmol). After sonicating for 60 seconds, the solution was transferred to the cuvette for the experiment. *Note: only a small portion of NaOAc is soluble in DMSO; this method ensures the preparation of a saturated solution of NaOAc.*

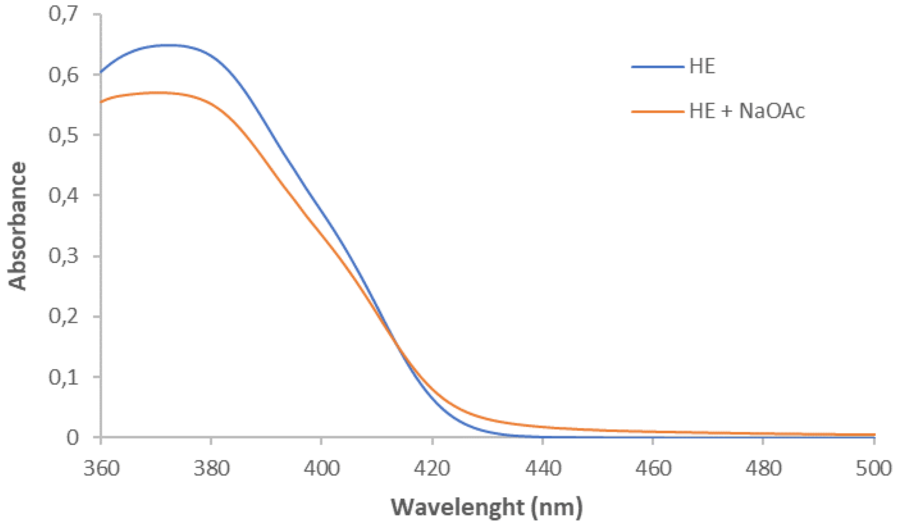

The HE absorption spectra clearly changed in the presence of NaOAc, suggesting an ground-state interaction between these two species.

Next, we repeated the experiment with increasing concentrations of benzoate **3**: 0.01, 0.02, 0.03 and 0.04 M.

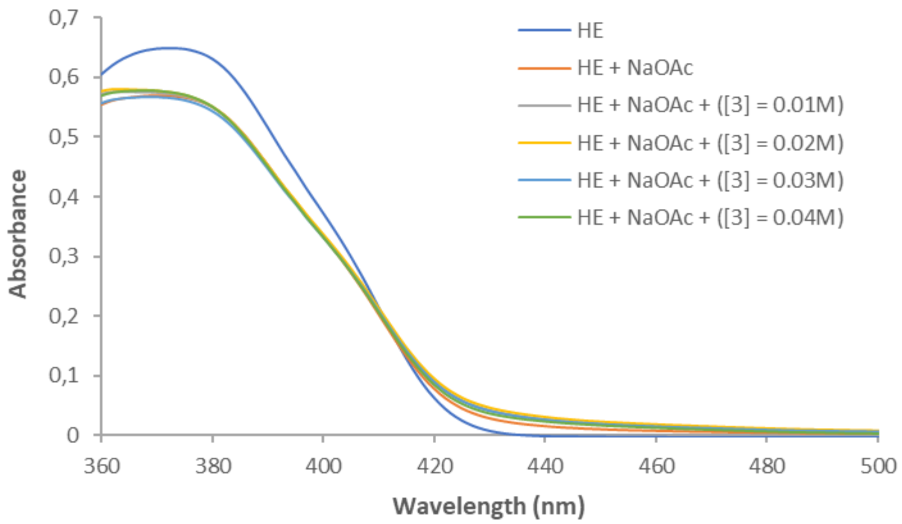

As evident from the graph, the increase in benzoate **3** concentrations did not significantly alter the absorption spectra of the HE/NaOAc mixture with a clear trend, indicating the absence of a ternary association in the ground state (small fluctuations are probably due to slight difference in concentration of NaOAc in each sample).

Emission studies

The emission spectra were recorded using a FS5 Spectrofluorometer equipped with photomultiplier detector, double monochromator and 150 W xenon light source. 3 mL of a $1 \cdot 10^{-3}$ M solution of Hantzsch ester (HE) in DMSO were placed in a quartz fluorescence cuvette (10x10 mm light path) equipped with septum. The sample was degassed with a stream of Argon for 15 min. The excitation wavelength was fixed at 400 nm (bandwidth = 3 nm), while the emission spectra was acquired from 410 nm to 700 nm. The same experiment was repeated in the presence of NaOAc. In this case, the sample was prepared in a vial and 2.5 mg of NaOAc were added (0.03 mmol). After sonicating for 60 seconds, the solution was transferred to the cuvette for degassing. *Note: only a small portion of NaOAc is soluble in DMSO; this method ensures the preparation of a saturated solution of NaOAc.*

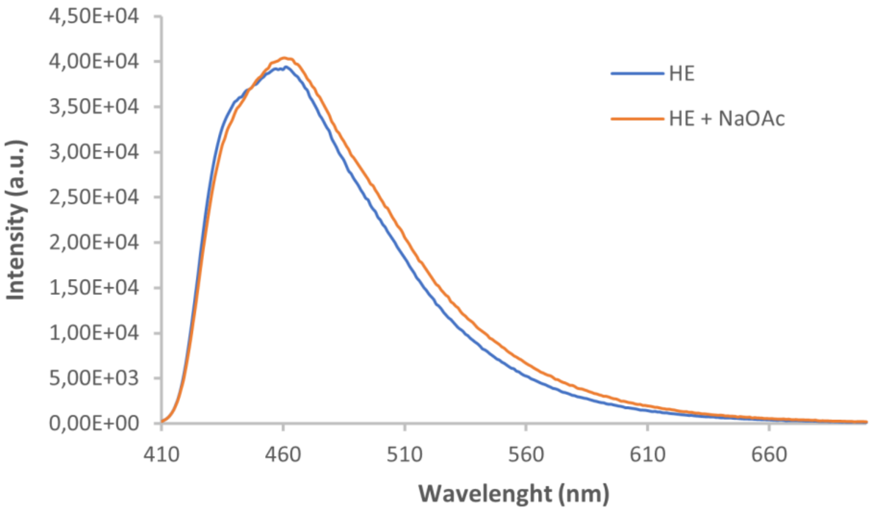

In the presence of NaOAc, we observed a small but appreciable change in the emission spectra of HE, possibly due to a ground state interaction between the two species and consisten with the UV-Vis studies above.

We repeated the same experiment using 373 nm as excitation wavelength (bandwidth = 1 nm) and [HE] = $1{\cdot}10^{-4}$ M. In this case, a small static quenching was visible in the presence of NaOAc.

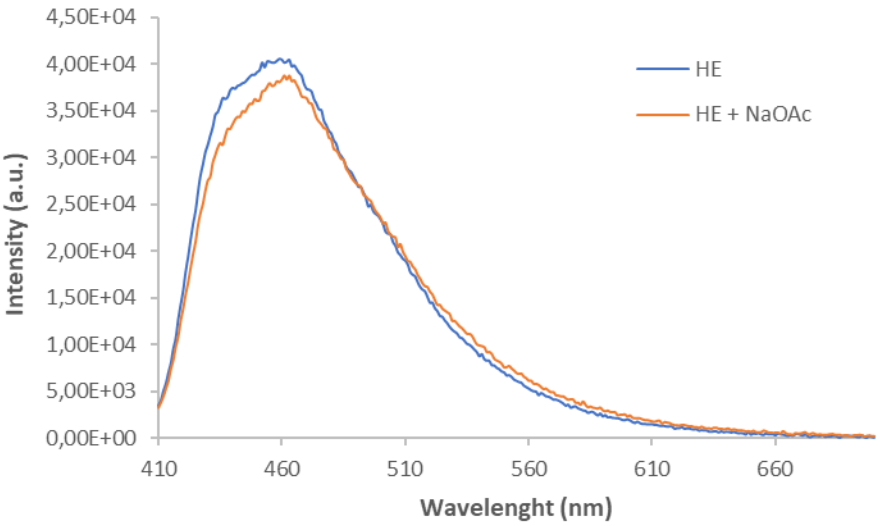

For Stern-Volmer quenching studies, the excitation wavelength was fixed at 373 nm and the emission spectra was acquired from 410 nm to 700 nm. The samples were prepared mixing a HE stock solution (final [HE] = $1.0{\cdot}10^{-4}$ M) with 0.1 M stock solution of benzoate **3** (final [**3**] = 0.01, 0.02, 0.03 and 0.04 M) in a total volume of 3 mL of DMSO in a quartz fluorescence cuvette (10x10 mm light path) equipped with septum. After

degassing the sample with a stream of argon for 15 minutes, the emission spectra of the samples were collected. In case of the quenching studies with NaOAc, the samples were prepared in a vial and 2.5 mg of NaOAc was added (0.03 mmol). After sonicating for 60 seconds, the solutions were transferred to the cuvette for degassing. *Note: only a small portion of NaOAc is soluble in DMSO; this method ensures the preparation of a saturated solution of NaOAc favoring the suggested interaction with HE.*

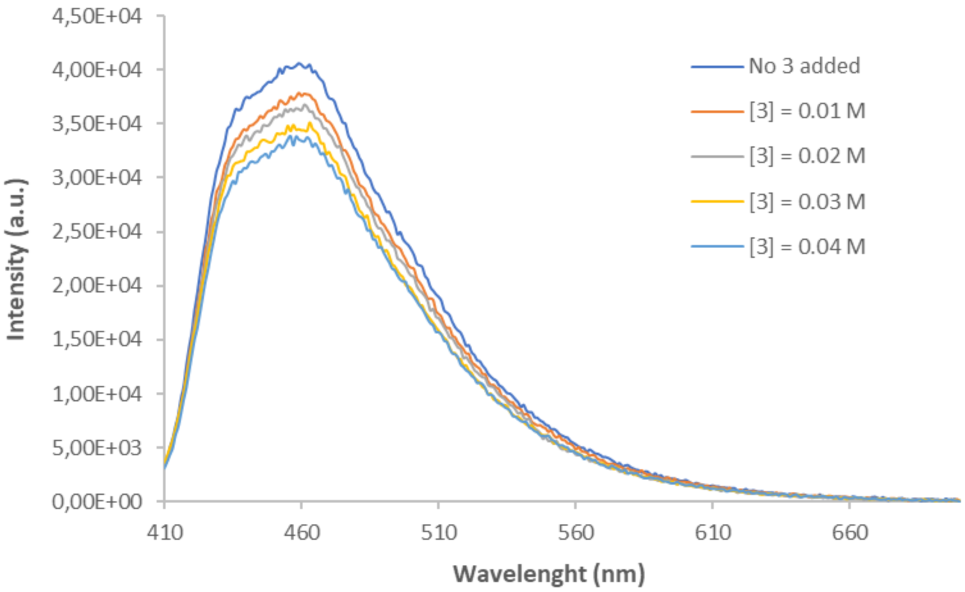

HE emission quenching with increasing concentratios of Benzoate **3**

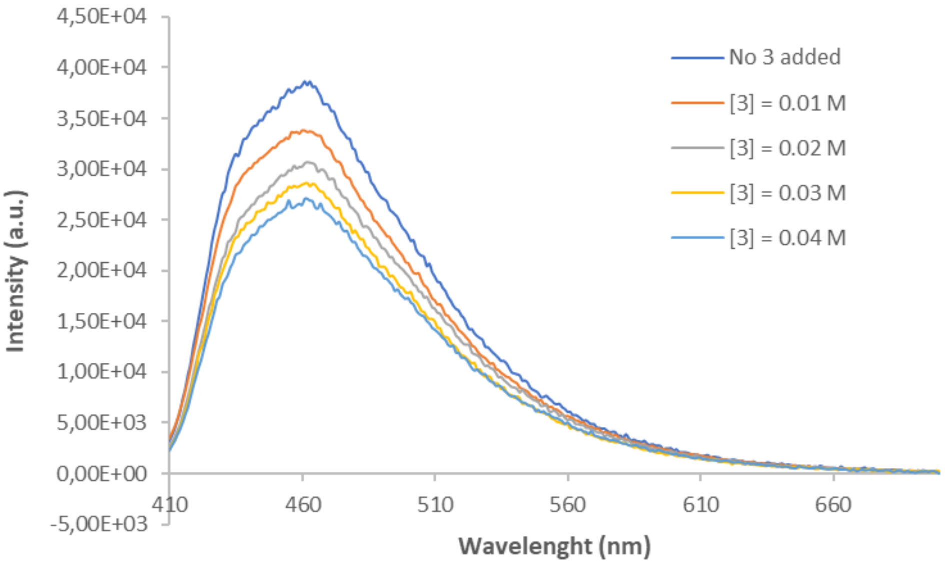

HE/NaOAc emission quenching with increasing concentratios of Benzoate **3**

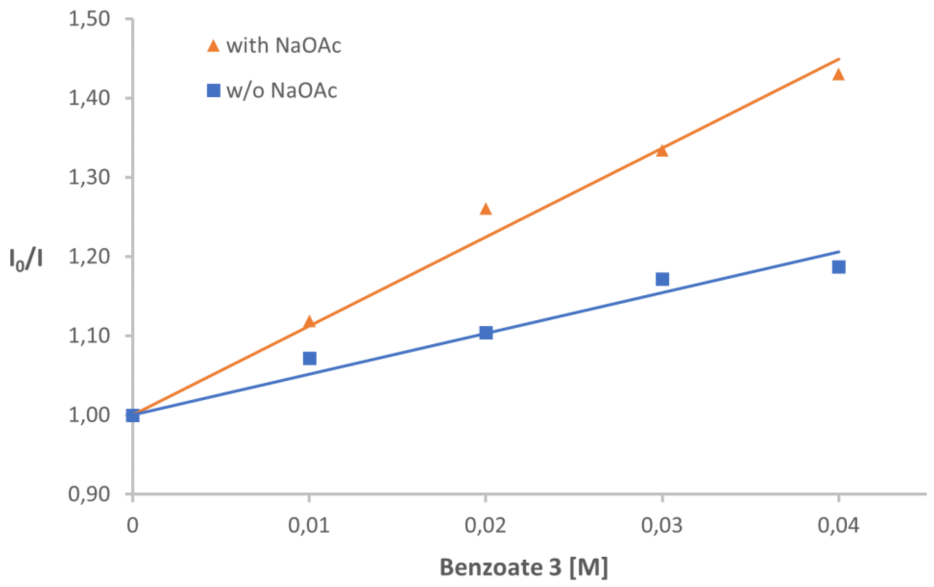

The Stern-Volmer plots show linear correlations between the amounts of **3** and the ratio I_0/I for the quenching of both HE and HE/NaOAc. On the basis of the following equation, it is possible to calculate the Stern-Volmer constants K_{SV} (J. R. Lakowicz *Principles of Fluorescence Spectroscopy*, chap. 3, pp. 52-93, Plenum Press, New York 1983):

$$I_0/I = 1 + K_{SV}[Q]$$

K_{sv} for HE = **5.15**; K_{sv} for HE/NaOAc = **11.22**

SET quencher experiment

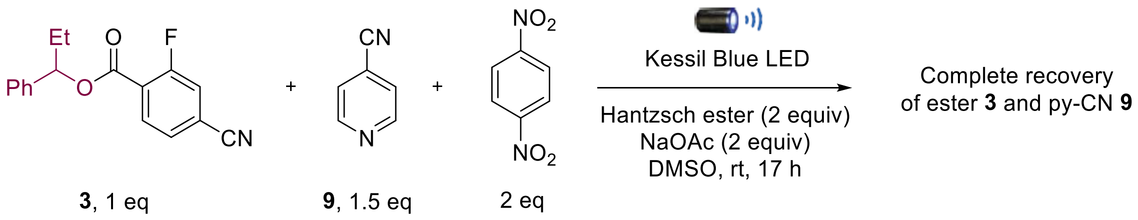

According to the general procedure G, 1-phenylpropyl 4-cyano-2-fluorobenzoate **3** (28.3 mg, 0.1 mmol), 4-cyanopyridine **9** (15.6 mg, 0.15 mmol), Hantzsch ester (50.6 mg, 0.2 mmol), NaOAc (16.4 mg, 0.2 mmol) and 1,4-dinitrobenzene (33.6 mg, 0.2 mmol) were dissolved in DMSO (2 mL) under air. The reaction was degassed via freeze pump thaw method (3 cycles), backfilled with Argon and irradiated with visible light. The reaction crude was analyzed by NMR and no conversion of 1-phenylpropyl 4-cyano-2-fluorobenzoate **3** and 4-pyridinecarbonitrile **9** was observed.

This experiment confirmed that single electron transfer processes are key to the photochemical reaction, since 1,4-dinitrobenzene is a known competitor for SET due to its strong tendency to accept an electron (E_{red} = -0.64 V vs SCE, refs: *Nat. Chem.* **2013**, *5*, 750-756 and *Int. J. Electrochem.* **2011**, Article ID 346043).

Studies of the reaction without 4-cyanopyridine

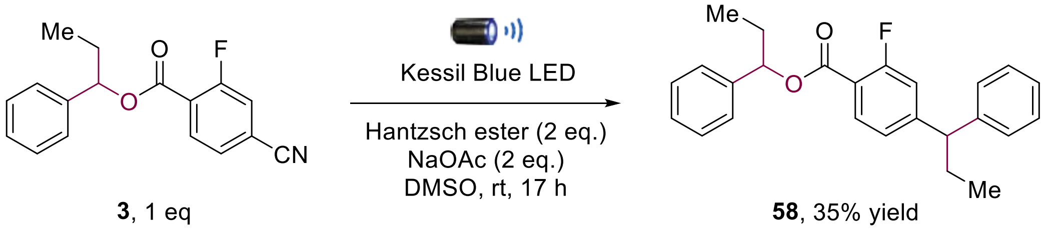

3, 1 eq

58, 35% yield

According to the general procedure G, 1-phenylpropyl 4-cyano-2-fluorobenzoate **3** (28.3 mg, 0.1 mmol), Hantzsch ester (50.6 mg, 0.2 mmol) and NaOAc (16.4 mg, 0.2 mmol) were dissolved in DMSO (2 mL) under air. The reaction was degassed via freeze pump thaw method (3 cycles), backfilled with Argon and irradiated with visible light. Reaction was done in 2 x 0.1 mmol scale. The crude product was purified using flash column chromatography (98/2 to 95/5 hexane/Et_2O) to provide the byproduct **58** as a 4:1 mixture of diastereoisomers and colorless oil (13 mg, 35% yield). Major diasteroisomer: ^{1}H NMR (300 MHz, $CDCl_3$) δ 7.86 (t, *J* = 7.7 Hz, 1H), 7.51 – 7.14 (m, 10H), 7.13 – 6.91 (m, 2H), 5.91 (t, *J* = 6.6 Hz, 1H), 3.82 (t, *J* = 7.7 Hz, 1H), 2.00 (m, 4H), 0.93 (m, 6H). ^{13}C NMR (75 MHz, $CDCl_3$) δ 163.87 (d, *J* = 3.5 Hz), 162.30 (d, *J* = 260.1 Hz), 153.23 (d, *J* = 7.9 Hz), 143.63, 140.65, 132.30, 129.93, 128.77, 128.52, 127.94 (d, *J* = 3.4 Hz), 126.71 (d, *J* = 5.5 Hz), 123.71 (d, *J* = 3.3 Hz), 116.54, 116.24, 78.31, 53.13, 29.75, 28.31, 12.70, 9.97. ^{19}F NMR (282 MHz, $CDCl_3$) δ -108.93. HRMS (APCI) $[M+H]^+$ found 376.1844, $C_{25}H_{25}FO_2$ requires 376.1833.

We propose that product **58** is obtained via radical-radical coupling of 1-phenylpropyl radical and the radical anion of benzoate **3**, before the desired β-scission fragmentation can occur.

TEMPO trapping experiment

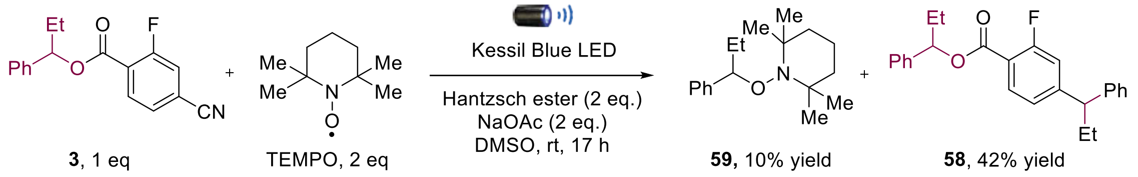

To probe the intermediacy of radical species, a trapping experiment was performed using TEMPO [(2,2,6,6- tetramethylpiperidin-1-yl)oxyl] as a radical scavenger. According to the general procedure G, 1-phenylpropyl 4-cyano-2-fluorobenzoate (28.3 mg, 0.1 mmol), Hantzsch ester (50.6 mg, 0.2 mmol), NaOAc (16.4 mg, 0.2 mmol) and TEMPO (31.2 mg, 0.2 mmol) were dissolved in DMSO (2 mL) under air. The reaction was degassed via freeze pump thaw method (3 cycles), backfilled with Argon and irradiated with visible light. Reaction was done in 2 x 0.1 mmol scale. The crude product was purified using flash column chromatography (98/2 to 95/5 hexane/Et_2O) to provide the TEMPO adduct **59** in 10% yield. The NMR spectral data match with the previously published data (*Nat. Chem.* **2019**, *11*, 1158-1166): ^{1}H NMR (300 MHz, $CDCl_3$) δ 7.27 (m, 5H), 4.53 (dd, *J* = 9.5, 4.0 Hz, 1H), 2.18 – 2.00 (m, 1H), 1.88 – 1.70 (m, 1H), 1.48 (brs, 3H), 1.21 – 1.39 (m, 6H), 1.17 (brs, 3H), 1.01 (brs, 3H), 0.89 – 0.81 (m, 1H), 0.66 (t, *J* = 7.5 Hz, 3H), 0.60 (brs, 2H).

The detection of the TEMPO product **59** confirms the formation of the proposed radical specie, which is obtained after the reduction of benzoate **3** and β-scission of the corresponding radical anion.

5. Metal-free deoxygenative coupling of alcohol-derived benzoates and DNA headpieces for DEL synthesis

LCMS Analysis

Liquid chromatography-mass spectrometry (HPLC-MS) analyses were performed on Thermo Scientific Dionex Ultimate 3000 UHPLC coupled with Bruker Amazon SL using an Electrospray ionization (ESI) ion source.

Column: Reverse phase (Waters XBridge Premier Oligo BEH C18 column 130Å, 2.5 µm, 2.1x50mm); column oven temperature: 30 °C; flow rate: 0.2 mL/min.

Mobile phase: (A). H_2O containing 10mM Et_3N and 100 mM HFIP; (B). MeOH.

UHPLC method: 5% phase B isocratic for 5 min, 5% to 50% phase B gradient 10 min, 50% phase B isocratic for 5 min, 50% to 5% phase B gradient 2 min.

UV detection: 260 nm.

MS method: capillary voltage 4500 V, range: 700-2200 *m/z*; ionization polarity: negative, triply charged mass was observed (M–3)/3.

Injection amount: 20 µL of purified sample (ca. 50 µM).

Important note: normally, the MS signal is delayed by 0.2 min respect to the UV signal.

Synthesis of DNA derivative 60

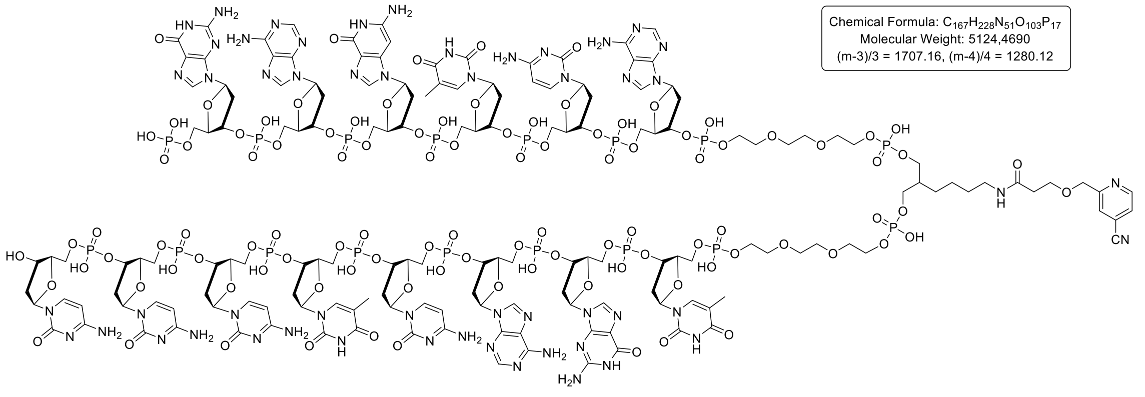

The DNA derivative **60** was synthesized according to the literature procedure (*J. Am. Chem. Soc.* **2020**, *142*, 20143–20151). 3-((4-cyanopyridin-2-yl)methoxy)propanoic acid **S42** (10 µL of 1.0 M stock solution in DMA, 10 µmol, 100 equiv.) and

N,*N*-diisopropylethylamine (10 μL of 1.0 M stock solution in DMA, 10 μmol, 100 equiv.) were added in an Eppendorf tube containing 1-[bis(dimethylamino)methylene]-1*H*-1,2,3-triazolo[4,5-b]pyridinium 3-oxide hexafluorophosphate (HATU; 3.8 mg, 10 μmol, 100 equiv.). The reaction mixture was vortexed and allowed to stand at room temperature for 1 h, followed by the addition to a solution of the DNA headpiece (10 μL of 10 mM stock solution in H2O, 0.1 μmol, 1.0 equiv.) in borate buffer (70 μL, 100 mM, pH 9.5). The mixture was vortexed and allowed to stand at room temperature for 3 h. Sodium chloride (10 μL of 5.0 M stock solution in H2O) and cold ethanol (0.3 mL) were added. The resulting mixture was vortexed and allowed to stand at –20 °C for 30 min. The suspension was centrifuged at 12000 RPM for 3 min. After discarding the supernatant, the pellet was dried under reduced pressure and redissolved in H2O (100 μL) to afford a stock solution of DNA-conjugate **60** (1.0 mM). *Note: small impurities (< 5% by mass) are coeluenting with DNA derivative* ***60****. m-3/3 = 1611 is due to the loss of one C and a phosphate from* ***60****, while m-3/3 = 1769 is due an additional inclusion of acid* ***S42****.*

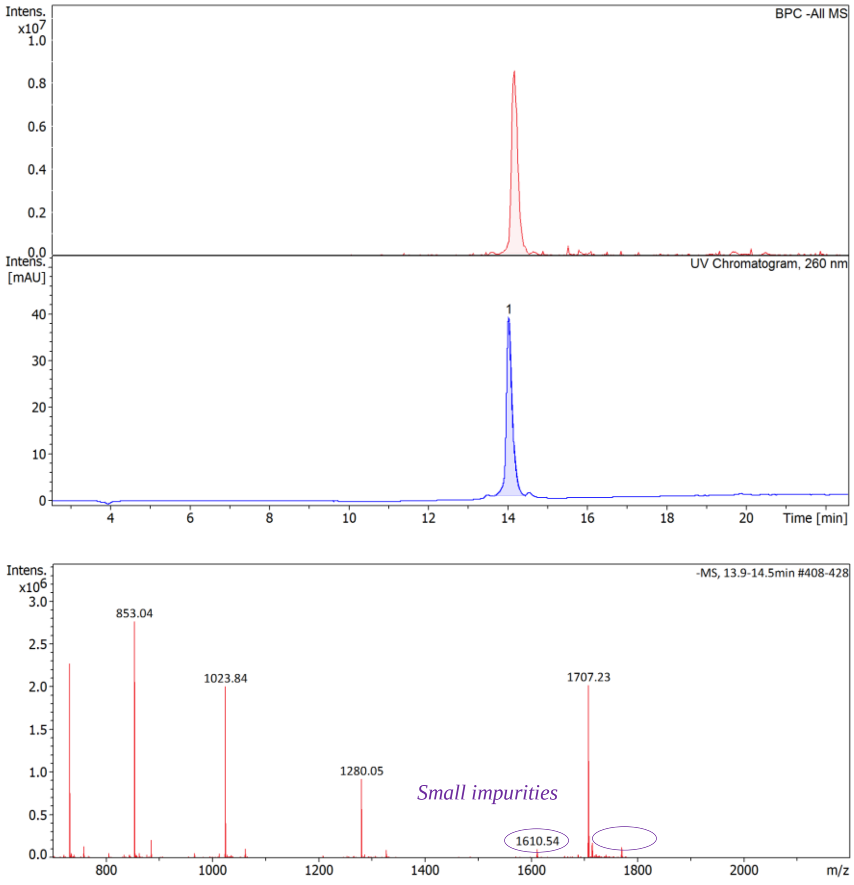

*Conjugate **60** is stable for 5-10 days in the freezer at -20 °C as stock solution. After this time, a new unknown impurity (coeluting with **60**) is found by LC-MS analysis:*

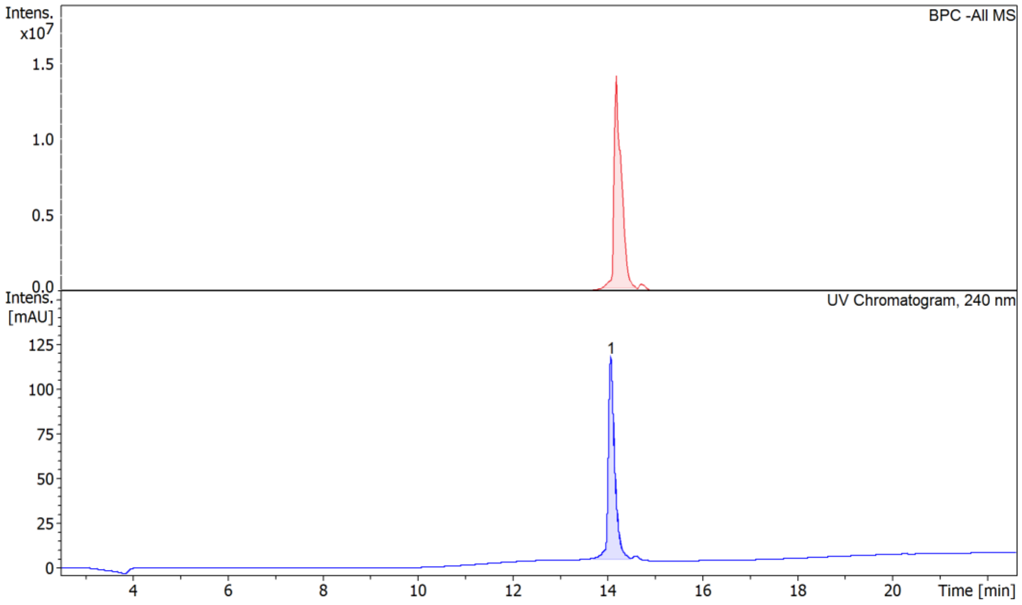

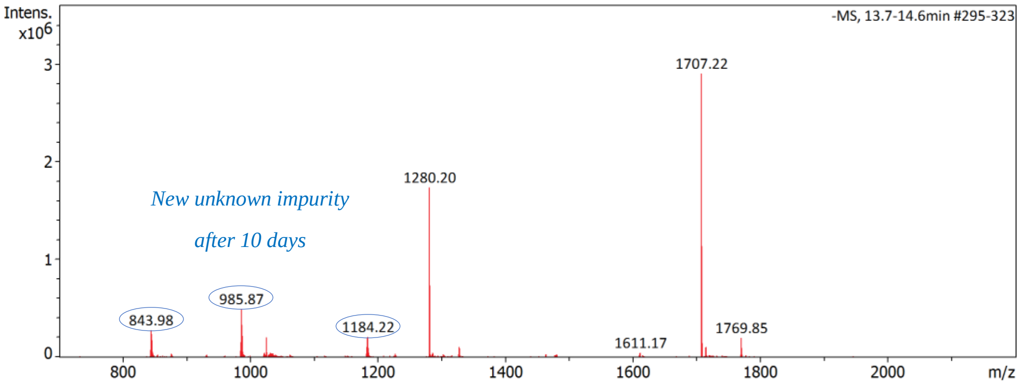

General procedure H

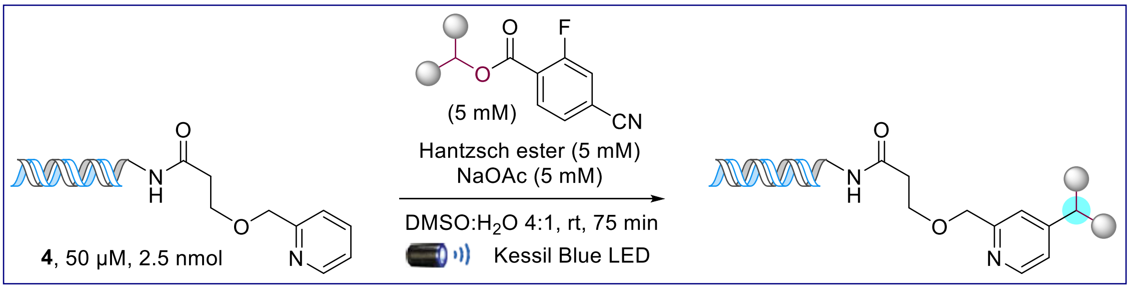

A 0.5-2 mL Microwave vial was charged with the appropriate alcohol derivative (5 μL of 50 mM stock solution in DMSO, 250 nmol, 100 equiv.), Hantzsch ester (5 μL of 50 mM stock solution in DMSO, 250 nmol, 100 equiv.), DMSO (30 μL), H2O (6.5 μL), sodium acetate (1 μL of 250 mM stock solution in H_2O, 250 nmol, 100 equiv.) and DNA derivative

60 (2.5 µL of 1 mM stock solution in H_2O, 2.5 nmol, 1 equiv.). The vial was flushed 15 seconds with Argon and sealed. The reaction was irradiated with visible light for 75 minutes. *Note: the vial was placed at 3 cm distance from the Kessil LED lamp and the reaction temperature was kept contast using a fan placed on top of the reaction at 20 cm distance.* After irradiation, the functionalized DNA was purified using Mini Quick Spin Oligo Column by Roche and analyzed by HPLC-MS.

Yield determination for the DNA functionalization

The yield of the DNA functionalization was determined by LC-MS as reported in the literature for DEL synthesis (*J. Am. Chem. Soc.* **2020**, *142*, 20143–20151 and ref therein) by integration of the UV absorbance at 260 nm, assuming total DNA recovery and that all DNA compounds have similar UV absorbance. To calculate the yield, the area of the product was divided by the summed-up area of all DNA containing compounds. Non-DNA impurities (molecular weight < 1000 g/mol) were not included in the yield calculation.

*Note: During our studies we noticed that most of the UV peaks of the desired coupling products have a small shoulder. we investigated this issue during the optimization phase with alcohol derivative 4-phenyltetrahydro-2H-pyran-4-yl 4-cyano-2-fluorobenzoate **S21**.*

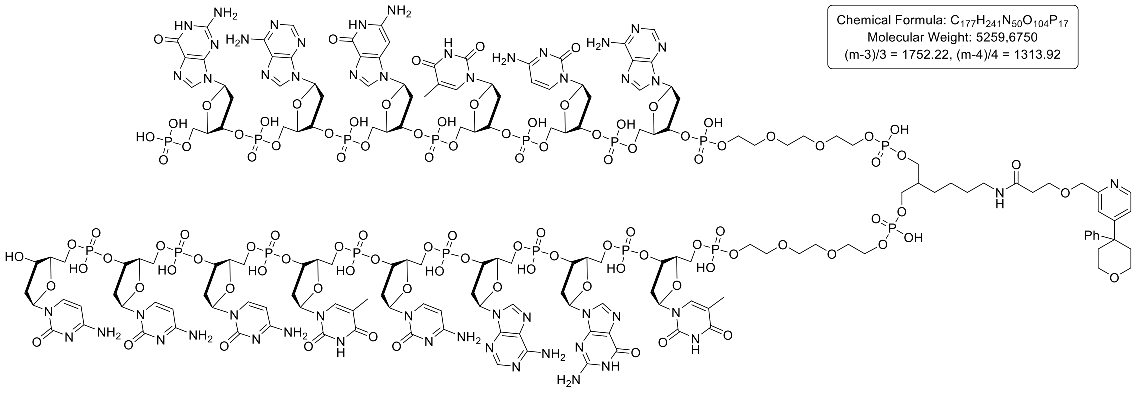

*Integration of UV peak 3 revealed a yield of 88% of the expected DNA-conjugate coupling product with alcohol-derived benzoate **S21**. The mass spectra of* <u>*the entire peak 3 (including the shoulder)*</u> *showed that the desired coupling product mass (m-3/3 = 1752, m-4/4 = 1314, m-5/5 = 1050, m-6/6 = 876, m-7/7 = 750) is the major signal (>*

90%, considering only m-3/3 adducts). We identified the major impurity (m-3/3 = 1656) as the coupling product of the initial impurity found in the starting conjugate ***60*** *(m-3/3 = 1611, which is due to the loss of one C and a phosphate from* ***60****) and alcohol-derived benzoate* ***S21****. Therefore, we assumed that integration of UV peak 3 is a good estimation of the reaction yield despite the small shoulder, which is arguably due to the coupling product of impurity m-3/3 = 1611 found in the starting conjugate* ***60****.*

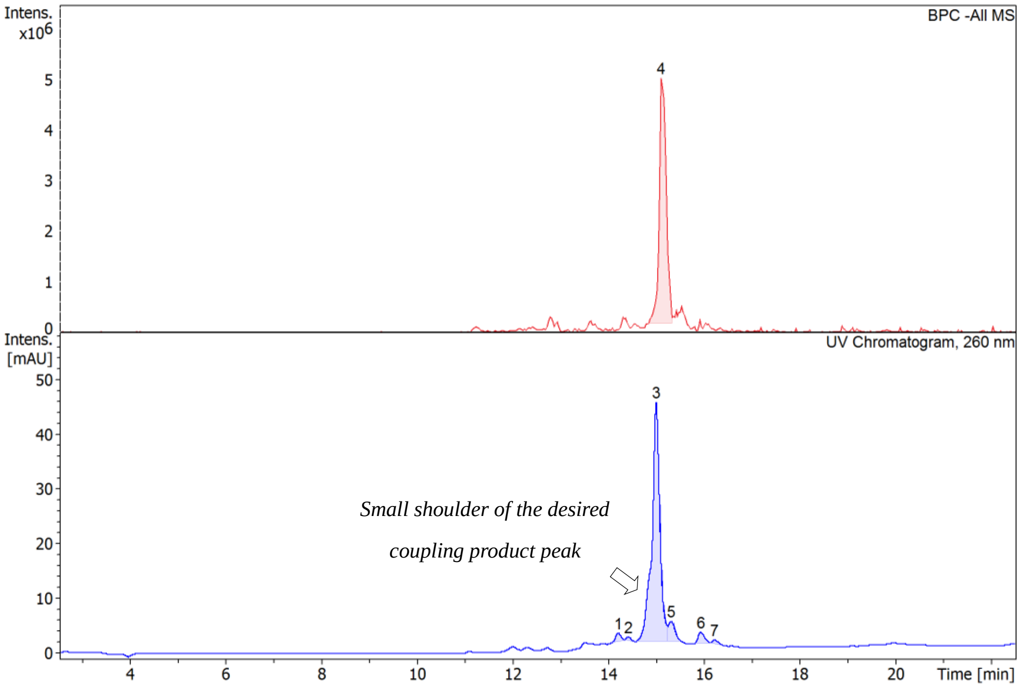

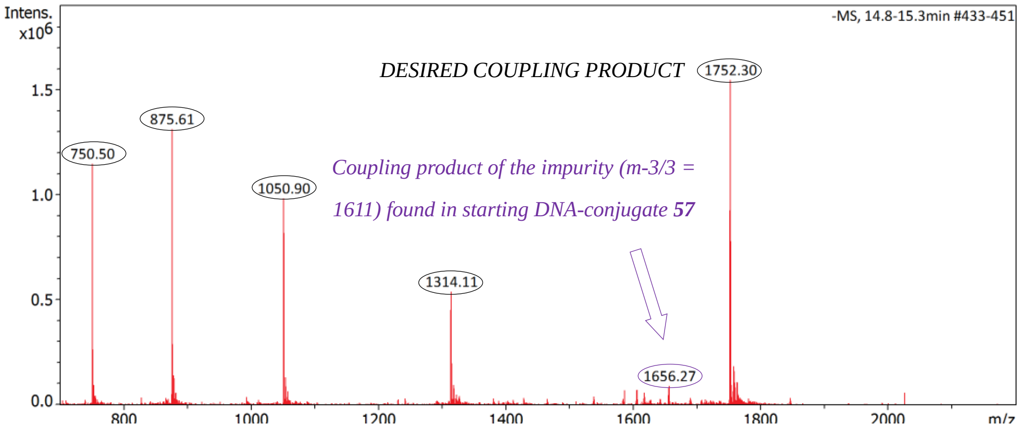

#	RT [min]	Chromatogram	Area	S/N	Area Frac. %
1	14.2	UV Chromatogram, 260 nm	10.8190	6343412.0	1.86120
2	14.4	UV Chromatogram, 260 nm	4.1689	2695434.0	0.71718
3	15.0	UV Chromatogram, 260 nm	509.2200	184011456.0	87.60084
5	15.3	UV Chromatogram, 260 nm	34.0011	15563116.0	5.84920
6	15.9	UV Chromatogram, 260 nm	17.8049	8406527.0	3.06296
7	16.2	UV Chromatogram, 260 nm	5.2818	2761519.5	0.90863

Synthesis of DNA functionalized product 61

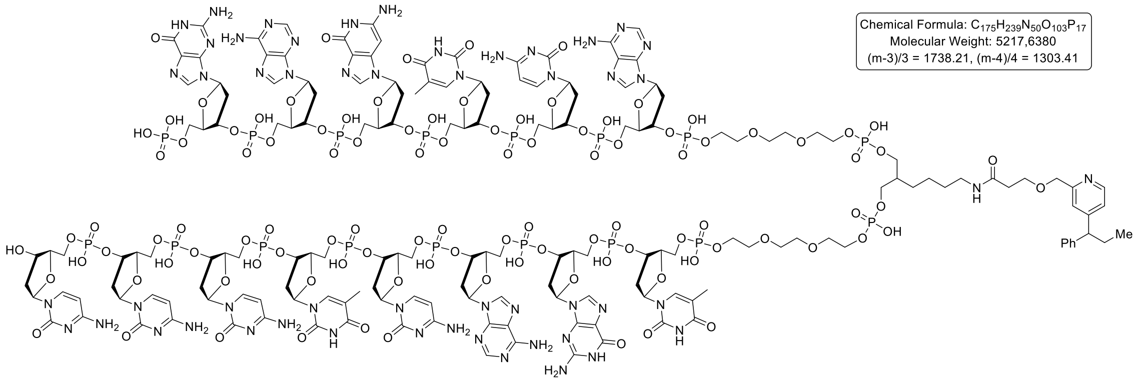

DNA functionalized product **61** was synthesized and purified according to general procedure H using 1-phenylpropyl 4-cyano-2-fluorobenzoate **3** (5 µL of 50 mM stock solution in DMSO, 250 nmol, 100 equiv.), Hantzsch ester (5 µL of 50 mM stock solution in DMSO, 250 nmol, 100 equiv.), DMSO (30 µL), H2O (6.5 µL), sodium acetate (1 µL of 250 mM stock solution in H_2O, 250 nmol, 100 equiv.) and DNA derivative **60** (2.5 µL of 1 mM stock solution in H_2O, 2.5 nmol, 1 equiv.). Yield = 84%

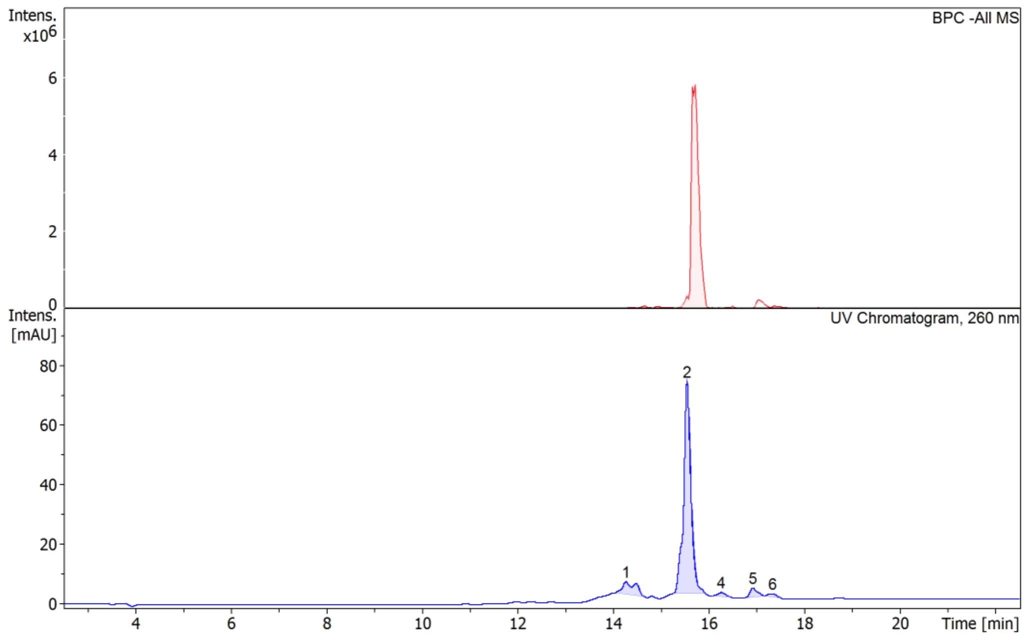

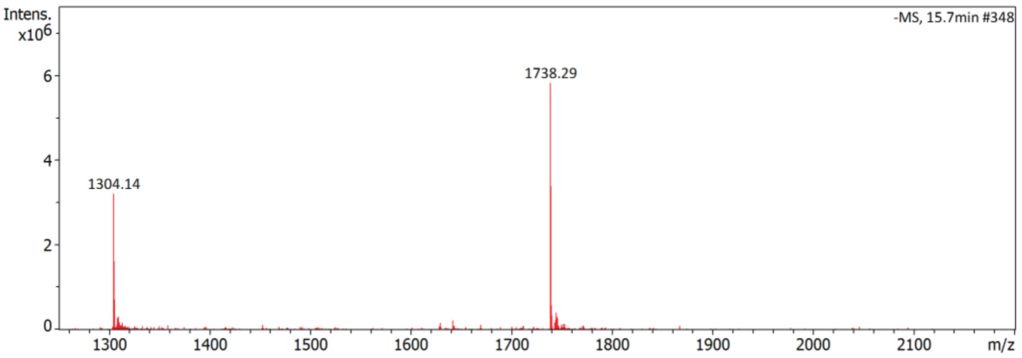

#	RT [min]	Chromatogram	Area	S/N	Area Frac. %
1	14.3	UV Chromatogram, 260 nm	81.411	538453.3	9.03249
2	15.5	UV Chromatogram, 260 nm	757.145	9352835.0	84.00442
4	16.3	UV Chromatogram, 260 nm	17.075	190021.3	1.89446
5	16.9	UV Chromatogram, 260 nm	33.362	407305.8	3.70149
6	17.3	UV Chromatogram, 260 nm	12.322	134175.0	1.36715

Synthesis of DNA functionalized product 62

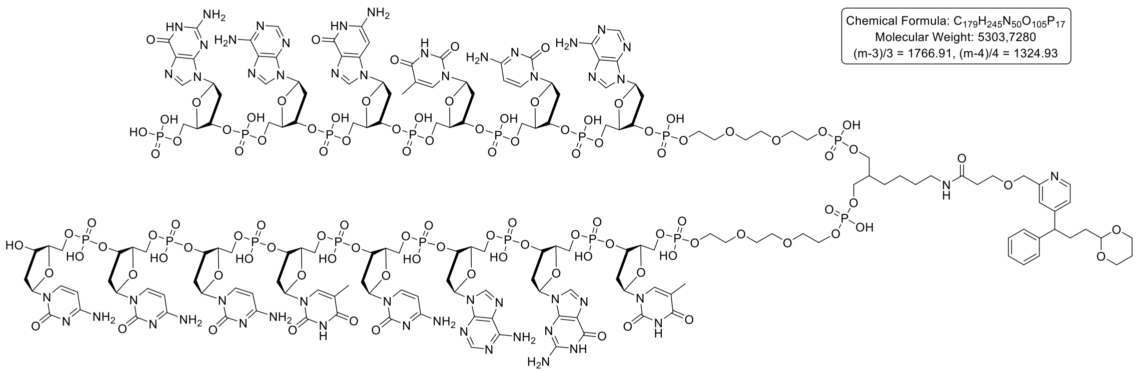

DNA functionalized product **62** was synthesized and purified according to general procedure H using 3-(1,3-dioxan-2-yl)-1-phenylpropyl 4-cyano-2-fluorobenzoate **S4** (5 µL of 50 mM stock solution in DMSO, 250 nmol, 100 equiv.), Hantzsch ester (5 µL of 50 mM stock solution in DMSO, 250 nmol, 100 equiv.), DMSO (30 µL), H2O (6.5 µL), sodium acetate (1 µL of 250 mM stock solution in H_2O, 250 nmol, 100 equiv.) and DNA derivative **60** (2.5 µL of 1 mM stock solution in H_2O, 2.5 nmol, 1 equiv.). Yield = 85%

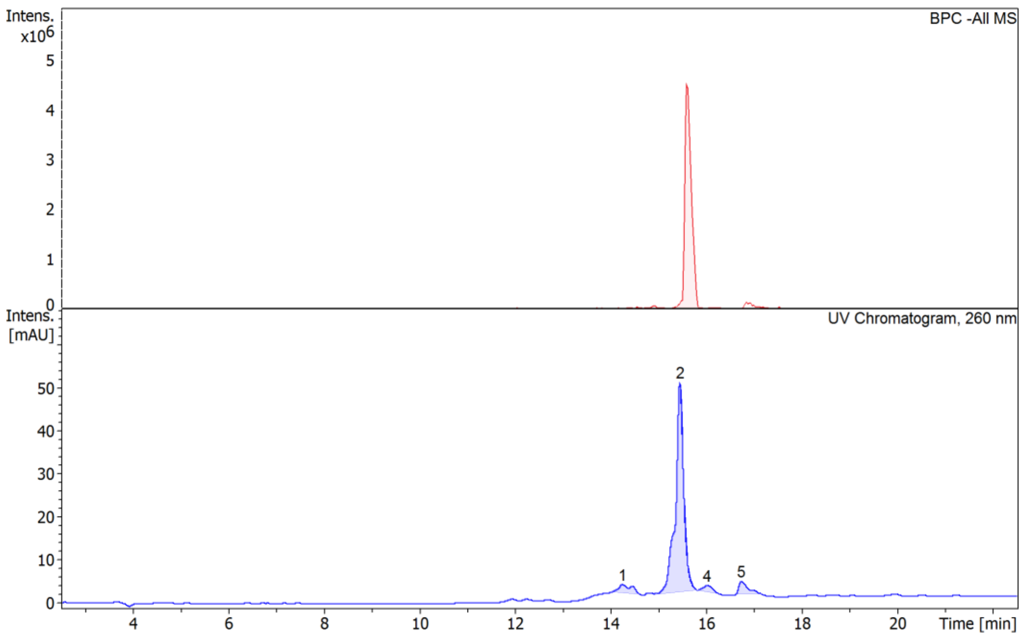

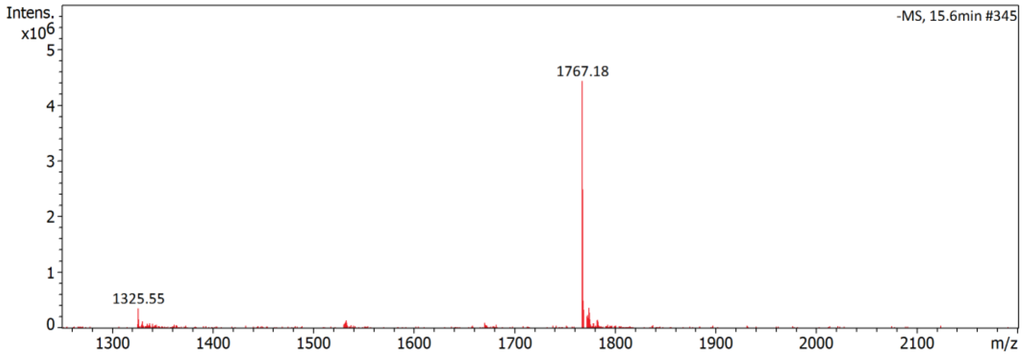

#	RT [min]	Chromatogram	Area	S/N	Area Frac. %
1	14.2	UV Chromatogram, 260 nm	38.642	1964.7	5.63525
2	15.4	UV Chromatogram, 260 nm	585.740	48928.0	85.42010
4	16.0	UV Chromatogram, 260 nm	19.904	1495.5	2.90264
5	16.7	UV Chromatogram, 260 nm	41.431	3013.8	6.04202

Synthesis of DNA functionalized product 63

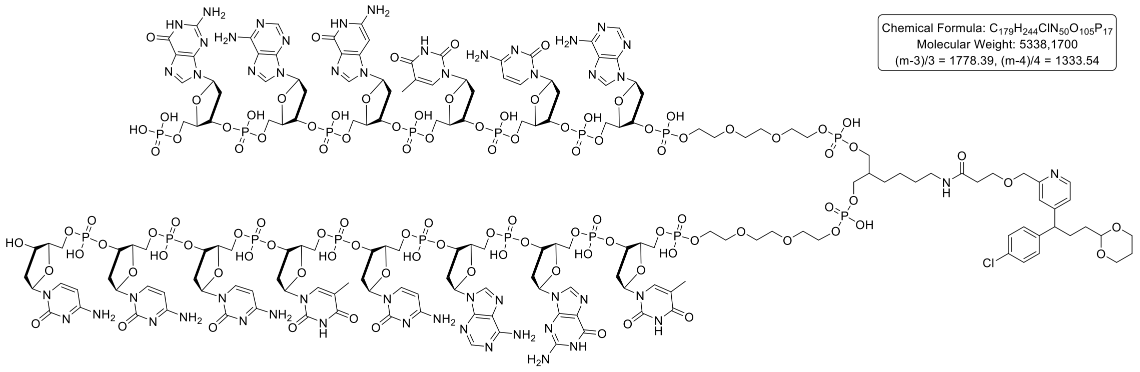

DNA functionalized product **63** was synthesized and purified according to general procedure H using 1-(4-chlorophenyl)-3-(1,3-dioxan-2-yl)propyl 4-cyano-

2-fluorobenzoate **S5** (5 μL of 50 mM stock solution in DMSO, 250 nmol, 100 equiv.), Hantzsch ester (5 μL of 50 mM stock solution in DMSO, 250 nmol, 100 equiv.), DMSO (30 μL), H2O (6.5 μL), sodium acetate (1 μL of 250 mM stock solution in H_2O, 250 nmol, 100 equiv.) and DNA derivative **60** (2.5 μL of 1 mM stock solution in H_2O, 2.5 nmol, 1 equiv.). Yield = 80%

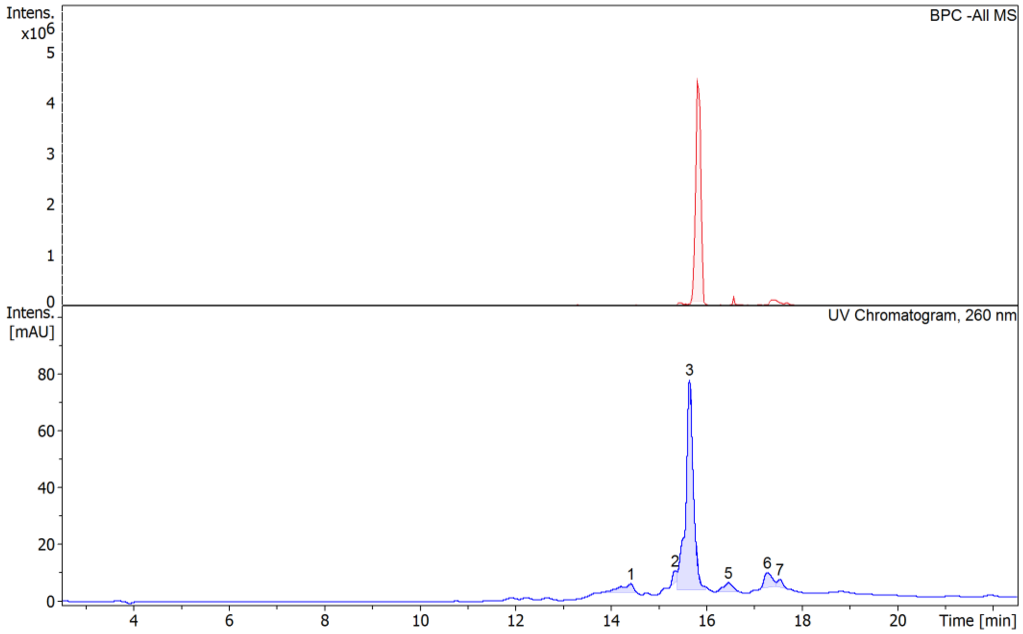

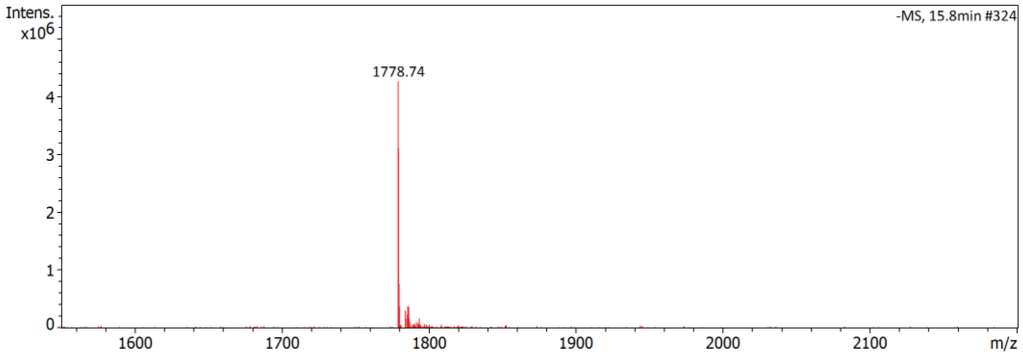

#	RT [min]	Chromatogram	Area	S/N	Area Frac. %
1	14.4	UV Chromatogram, 260 nm	58.897	3096.0	5.6394
2	15.3	UV Chromatogram, 260 nm	25.450	4237.0	2.4368
3	15.6	UV Chromatogram, 260 nm	831.107	74451.7	79.5786
5	16.5	UV Chromatogram, 260 nm	47.385	3313.2	4.5371
6	17.3	UV Chromatogram, 260 nm	59.791	5382.2	5.7250
7	17.5	UV Chromatogram, 260 nm	21.755	2722.4	2.0830

Synthesis of DNA functionalized product 64

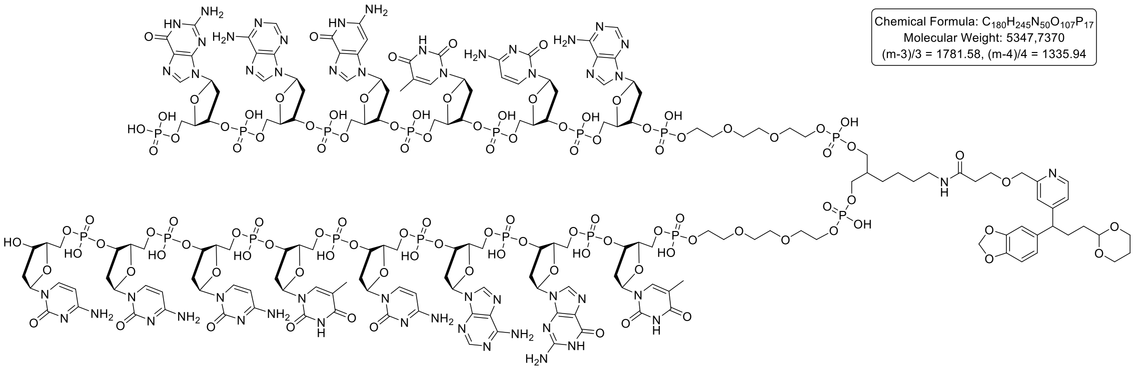

DNA functionalized product **64** was synthesized and purified according to general procedure H using 1-(benzo[d][1,3]dioxol-5-yl)-3-(1,3-dioxan-2-yl)propyl 4-cyano-2-fluorobenzoate **S6** (5 µL of 50 mM stock solution in DMSO, 250 nmol, 100 equiv.), Hantzsch ester (5 µL of 50 mM stock solution in DMSO, 250 nmol, 100 equiv.), DMSO (30 µL), H2O (6.5 µL), sodium acetate (1 µL of 250 mM stock solution in H_2O, 250 nmol, 100 equiv.) and DNA derivative **60** (2.5 µL of 1 mM stock solution in H_2O, 2.5 nmol, 1 equiv.). Yield = 87%

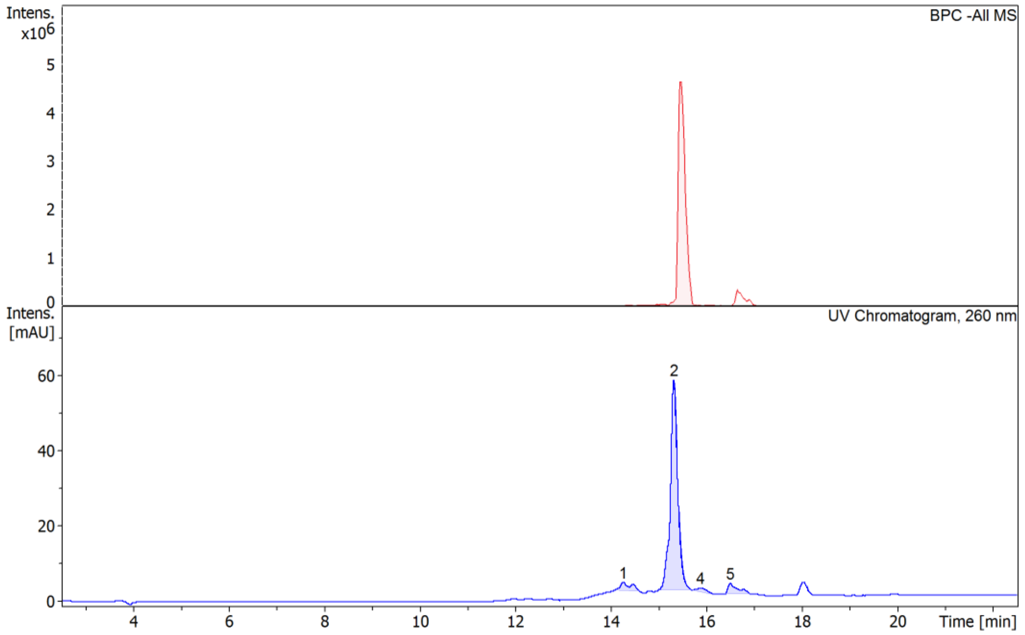

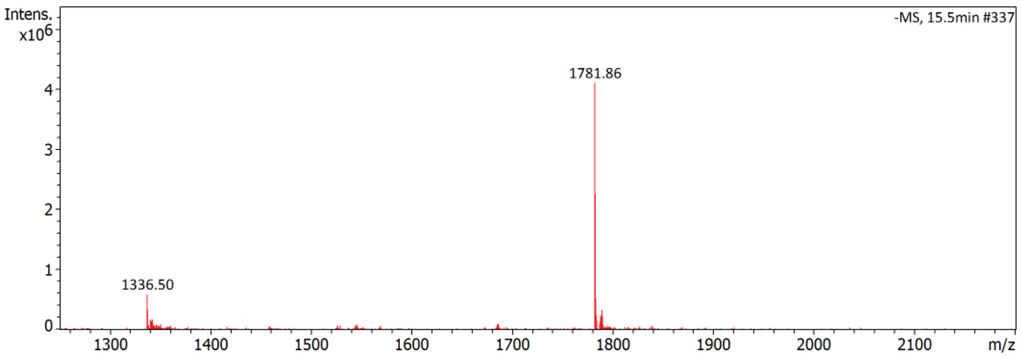

#	RT [min]	Chromatogram	Area	S/N	Area Frac. %
1	14.2	UV Chromatogram, 260 nm	33.301	4447314.5	4.84132
2	15.3	UV Chromatogram, 260 nm	599.825	117526664.0	87.20323
4	15.9	UV Chromatogram, 260 nm	14.368	2236430.8	2.08889
5	16.5	UV Chromatogram, 260 nm	40.353	5723126.0	5.86655

Synthesis of DNA functionalized product 65

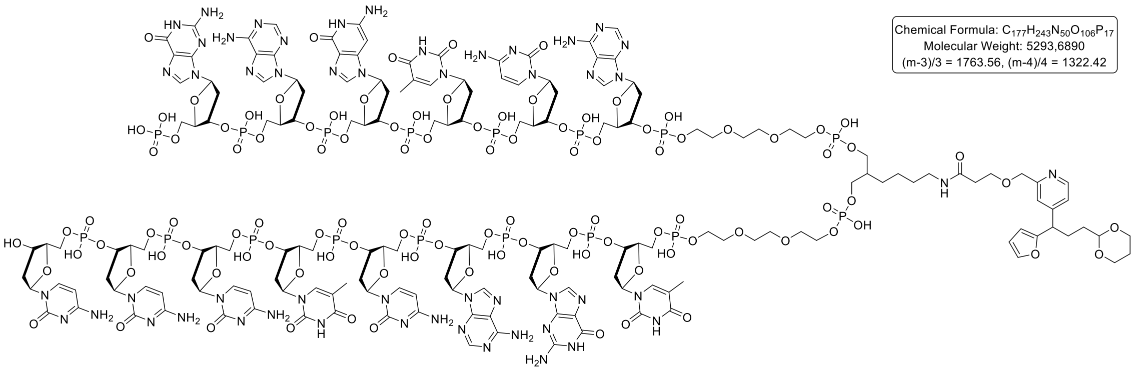

DNA functionalized product **65** was synthesized and purified according to general procedure H using 3-(1,3-dioxan-2-yl)-1-(furan-2-yl)propyl 4-cyano-2-fluorobenzoate **S8** (5 µL of 50 mM stock solution in DMSO, 250 nmol, 100 equiv.), Hantzsch ester (5 µL of 50 mM stock solution in DMSO, 250 nmol, 100 equiv.), DMSO (30 µL), H2O (6.5 µL), sodium acetate (1 µL of 250 mM stock solution in H_2O, 250 nmol, 100 equiv.) and DNA derivative **60** (2.5 µL of 1 mM stock solution in H_2O, 2.5 nmol, 1 equiv.). Yield = 85%.

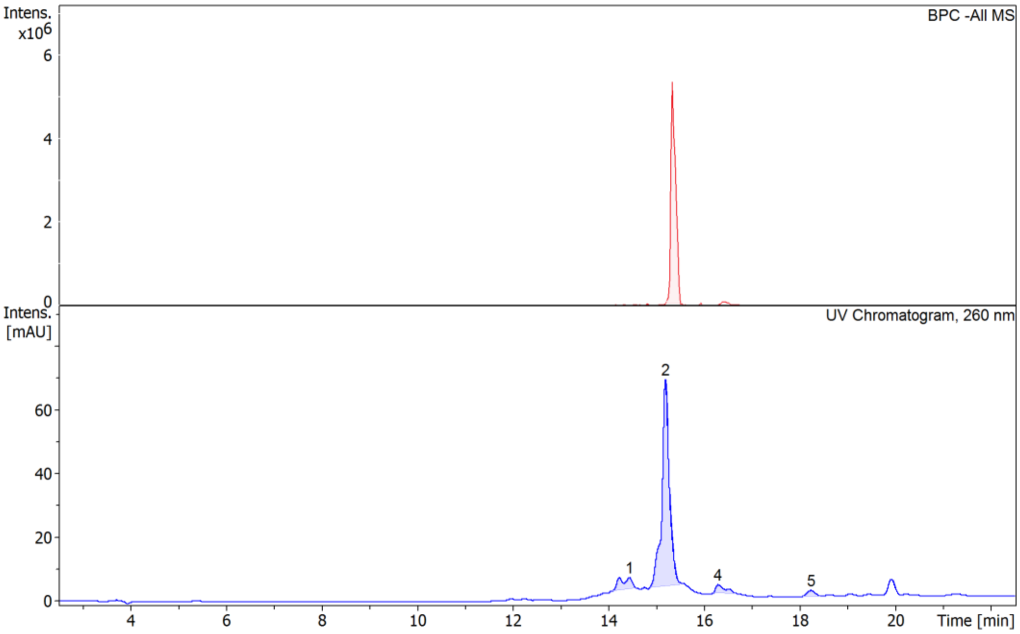

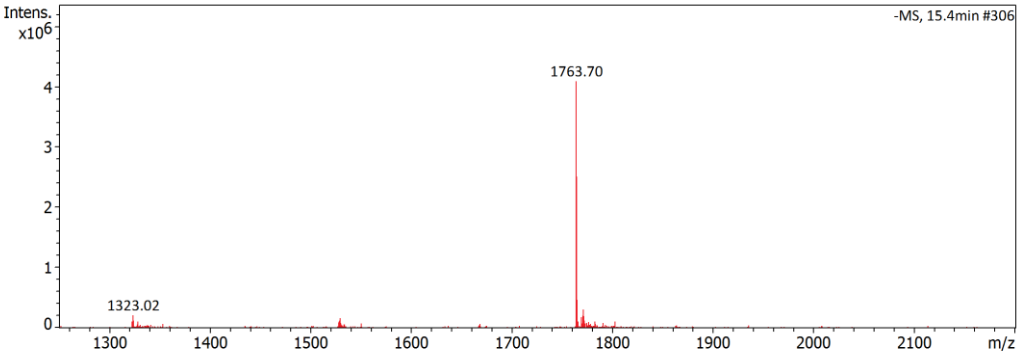

#	RT [min]	Chromatogram	Area	S/N	Area Frac. %
1	14.4	UV Chromatogram, 260 nm	62.282	3622.2	7.5577
2	15.2	UV Chromatogram, 260 nm	703.985	65140.1	85.4269
4	16.3	UV Chromatogram, 260 nm	37.377	2650.8	4.5356
5	18.2	UV Chromatogram, 260 nm	20.435	1840.1	2.4797

Synthesis of DNA functionalized product 66

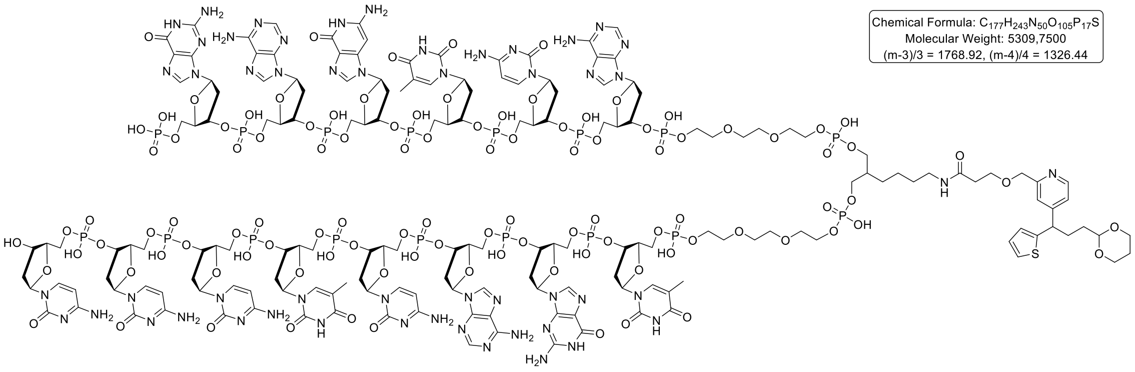

DNA functionalized product **66** was synthesized and purified according to general procedure H using 3-(1,3-dioxan-2-yl)-1-(thiophen-2-yl)propyl

4-cyano-2-fluorobenzoate **S9** (5 μL of 50 mM stock solution in DMSO, 250 nmol, 100 equiv.), Hantzsch ester (5 μL of 50 mM stock solution in DMSO, 250 nmol, 100 equiv.), DMSO (30 μL), H2O (6.5 μL), sodium acetate (1 μL of 250 mM stock solution in H_2O, 250 nmol, 100 equiv.) and DNA derivative **60** (2.5 μL of 1 mM stock solution in H_2O, 2.5 nmol, 1 equiv.). Yield = 87%

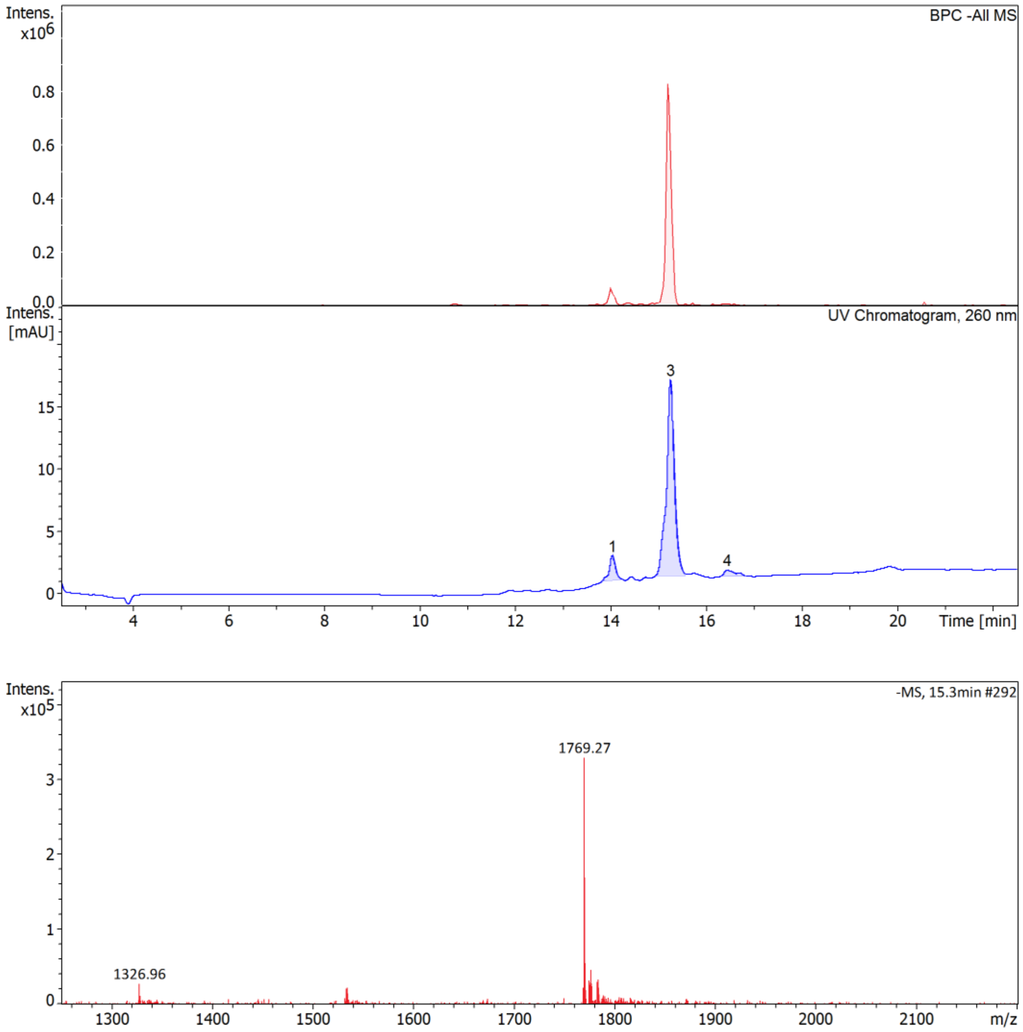

#	RT [min]	Chromatogram	Area	S/N	Area Frac. %
1	14.0	UV Chromatogram, 260 nm	18.0562	8300760.5	8.6912
3	15.2	UV Chromatogram, 260 nm	181.3405	66098280.0	87.2866
4	16.4	UV Chromatogram, 260 nm	8.3562	2212677.5	4.0222

Synthesis of DNA functionalized product 67

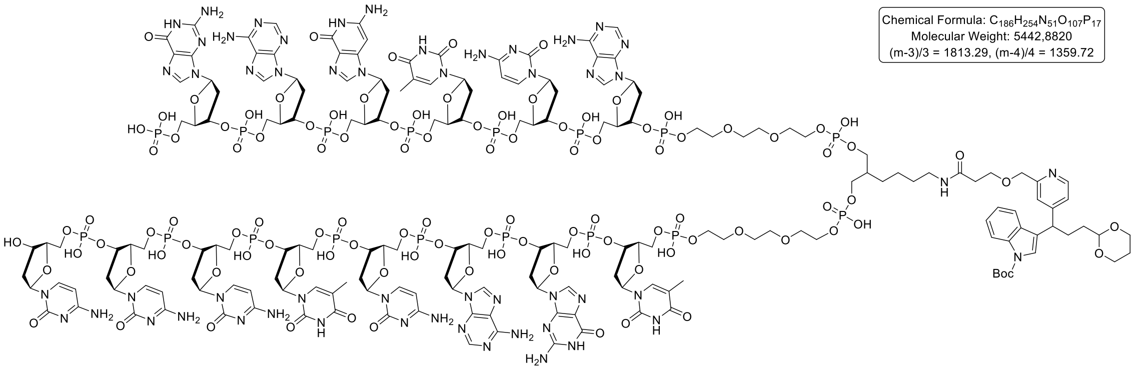

DNA functionalized product **67** was synthesized and purified according to general procedure H using *tert*-butyl 3-(1-((4-cyano-2-fluorobenzoyl)oxy)-3-(1,3-dioxan-2-yl)propyl)-1H-indole-1-carboxylate **S11** (5 µL of 50 mM stock solution in DMSO, 250 nmol, 100 equiv.), Hantzsch ester (5 µL of 50 mM stock solution in DMSO, 250 nmol, 100 equiv.), DMSO (30 µL), H2O (6.5 µL), sodium acetate (1 µL of 250 mM stock solution in H_2O, 250 nmol, 100 equiv.) and DNA derivative **60** (2.5 µL of 1 mM stock solution in H_2O, 2.5 nmol, 1 equiv.). Yield = 57%

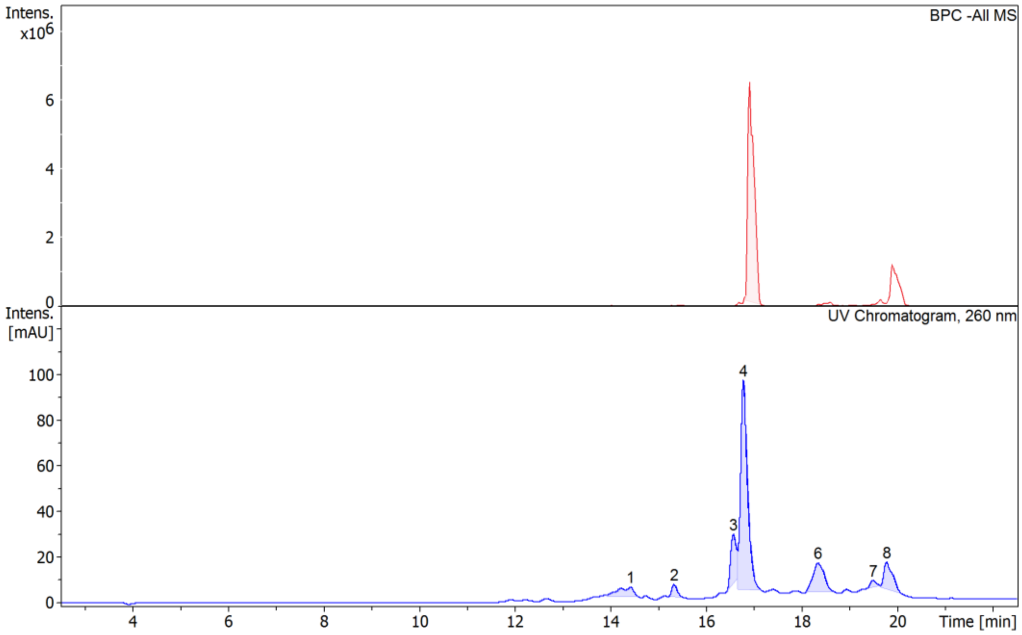

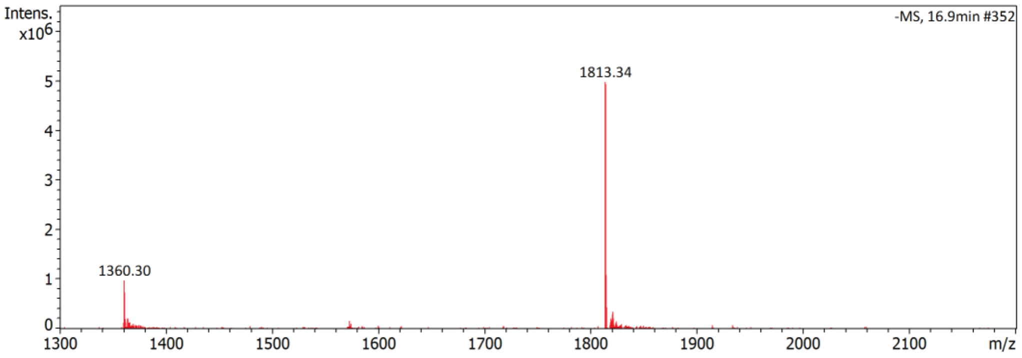

#	RT [min]	Chromatogram	Area	S/N	Area Frac. %
1	14.4	UV Chromatogram, 260 nm	96.366	4376.6	5.96862
2	15.3	UV Chromatogram, 260 nm	40.924	5353.1	2.53474
3	16.6	UV Chromatogram, 260 nm	171.693	21479.4	10.63418
4	16.8	UV Chromatogram, 260 nm	924.677	91883.9	57.27198
6	18.3	UV Chromatogram, 260 nm	209.478	12882.0	12.97451
7	19.5	UV Chromatogram, 260 nm	23.893	2919.1	1.47984
8	19.8	UV Chromatogram, 260 nm	147.506	11807.7	9.13612

Synthesis of DNA functionalized product 68

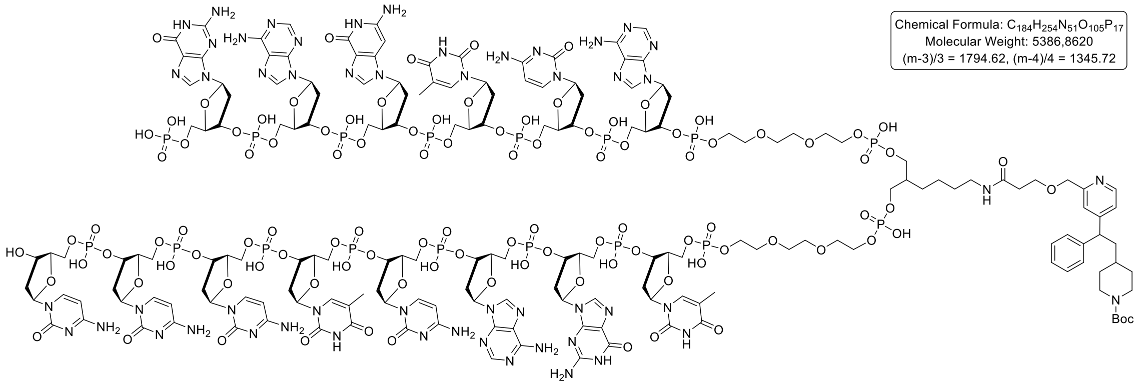

DNA functionalized product **68** was synthesized and purified according to general procedure H using *tert*-butyl 4-(2-((4-cyano-2-fluorobenzoyl)oxy)-2-phenylethyl) piperidine-1-carboxylate **S13** (5 µL of 50 mM stock solution in DMSO, 250 nmol, 100 equiv.), Hantzsch ester (5 µL of 50 mM stock solution in DMSO, 250 nmol, 100 equiv.), DMSO (30 µL), H2O (6.5 µL), sodium acetate (1 µL of 250 mM stock solution in H_2O, 250 nmol, 100 equiv.) and DNA derivative **60** (2.5 µL of 1 mM stock solution in H_2O, 2.5 nmol, 1 equiv.). Yield = 59%

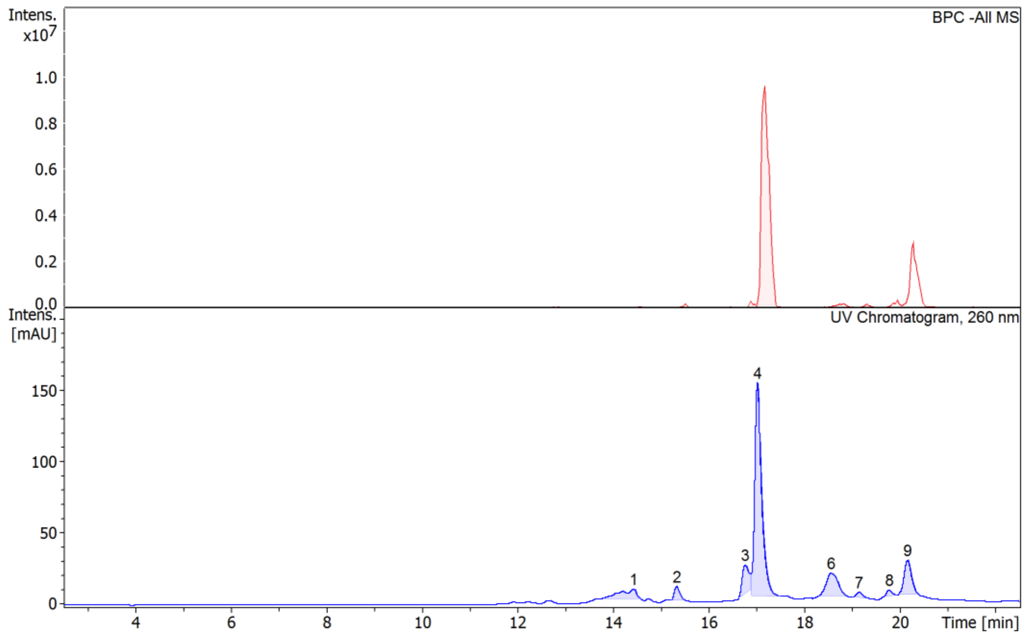

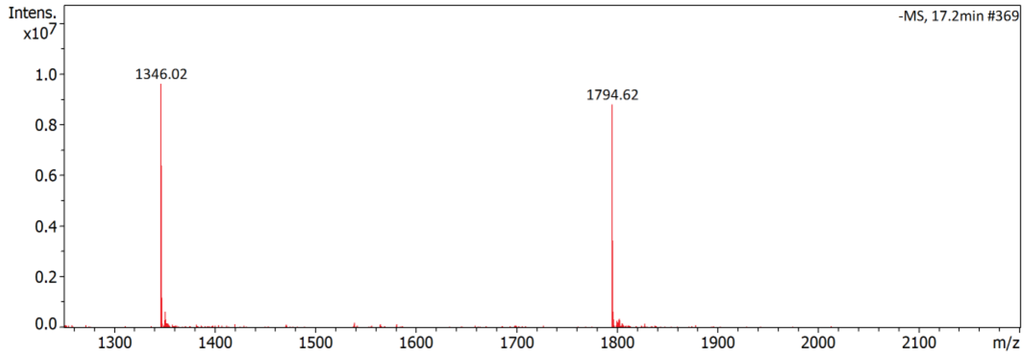

#	RT [min]	Chromatogram	Area	S/N	Area Frac. %
1	14.4	UV Chromatogram, 260 nm	171.334	7297.0	6.76805
2	15.3	UV Chromatogram, 260 nm	74.706	9582.4	2.95104
3	16.8	UV Chromatogram, 260 nm	193.592	20109.4	7.64727
4	17.0	UV Chromatogram, 260 nm	1488.276	149967.3	58.79001
6	18.6	UV Chromatogram, 260 nm	279.503	16388.8	11.04093
7	19.1	UV Chromatogram, 260 nm	33.553	3667.8	1.32542
8	19.8	UV Chromatogram, 260 nm	31.793	4137.2	1.25590
9	20.2	UV Chromatogram, 260 nm	258.755	24275.3	10.22136

Synthesis of DNA functionalized product 69

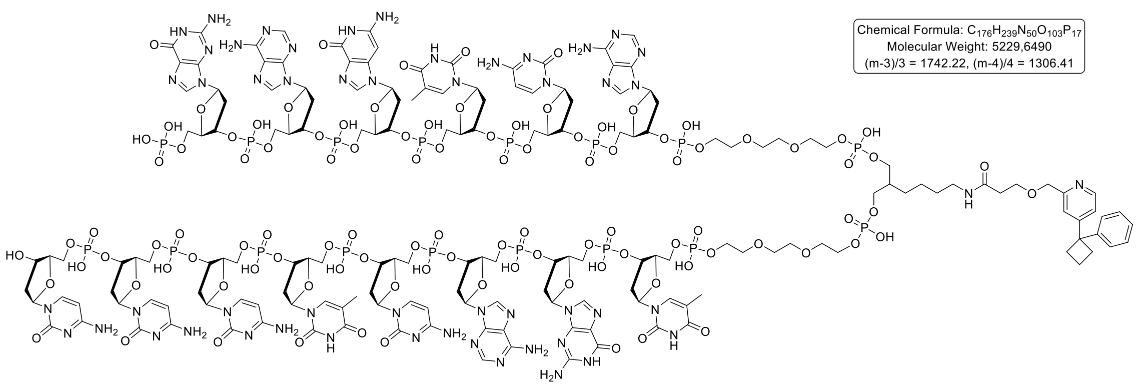

DNA functionalized product **69** was synthesized and purified according to general procedure H using 1-phenylcyclobutyl 4-cyano-2-fluorobenzoate **S18** (5 µL of 50 mM stock solution in DMSO, 250 nmol, 100 equiv.), Hantzsch ester (5 µL of 50 mM stock solution in DMSO, 250 nmol, 100 equiv.), DMSO (30 µL), H2O (6.5 µL), sodium acetate (1 µL of 250 mM stock solution in H_2O, 250 nmol, 100 equiv.) and DNA derivative **60** (2.5 µL of 1 mM stock solution in H_2O, 2.5 nmol, 1 equiv.). Yield = 87%

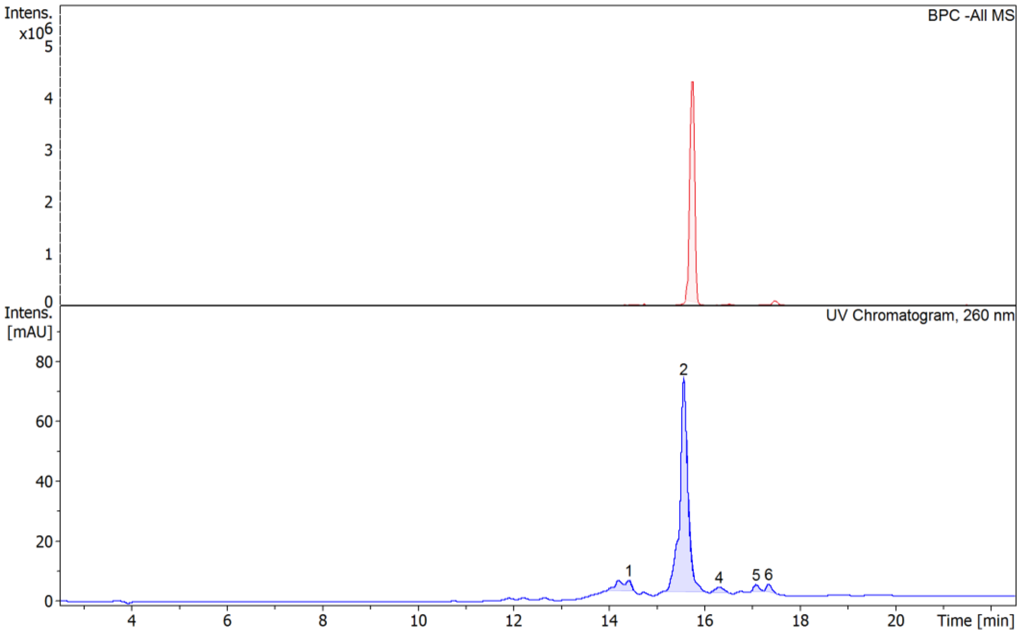

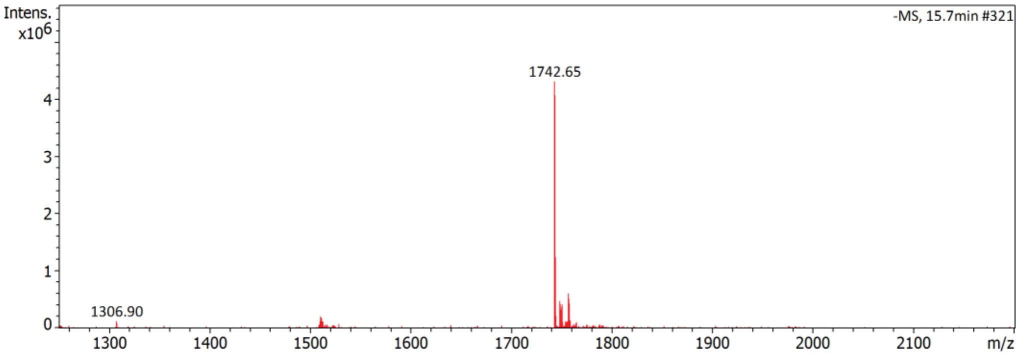

#	RT [min]	Chromatogram	Area	S/N	Area Frac. %
1	14.4	UV Chromatogram, 260 nm	66.887	3593.2	6.9065
2	15.6	UV Chromatogram, 260 nm	838.019	71129.1	86.5305
4	16.3	UV Chromatogram, 260 nm	25.190	1968.0	2.6010
5	17.1	UV Chromatogram, 260 nm	18.163	2263.8	1.8754
6	17.3	UV Chromatogram, 260 nm	20.208	2590.0	2.0866

Synthesis of DNA functionalized product 70

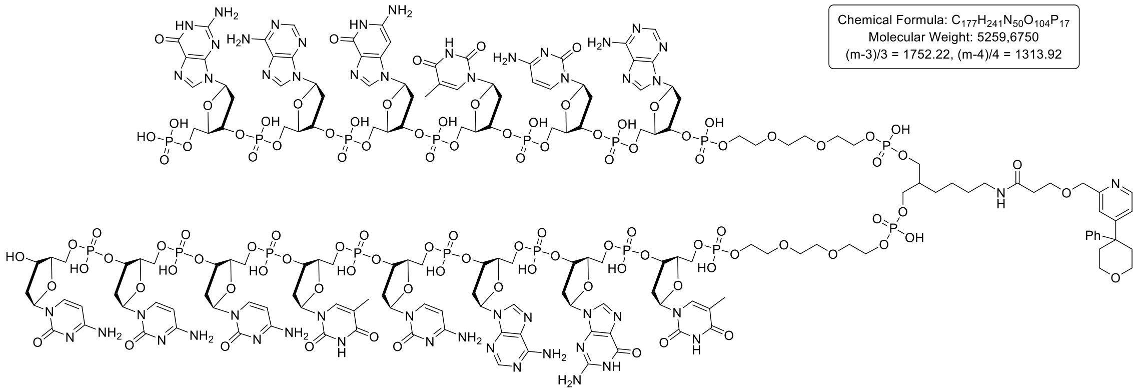

DNA functionalized product **70** was synthesized and purified according to general procedure H using 4-phenyltetrahydro-2*H*-pyran-4-yl 4-cyano-2-fluorobenzoate **S21** (5 μL of 50 mM stock solution in DMSO, 250 nmol, 100 equiv.), Hantzsch ester (5 μL of 50 mM stock solution in DMSO, 250 nmol, 100 equiv.), DMSO (30 μL), H2O (6.5 μL), sodium acetate (1 μL of 250 mM stock solution in H_2O, 250 nmol, 100 equiv.) and DNA derivative **60** (2.5 μL of 1 mM stock solution in H_2O, 2.5 nmol, 1 equiv.). Yield = 90%

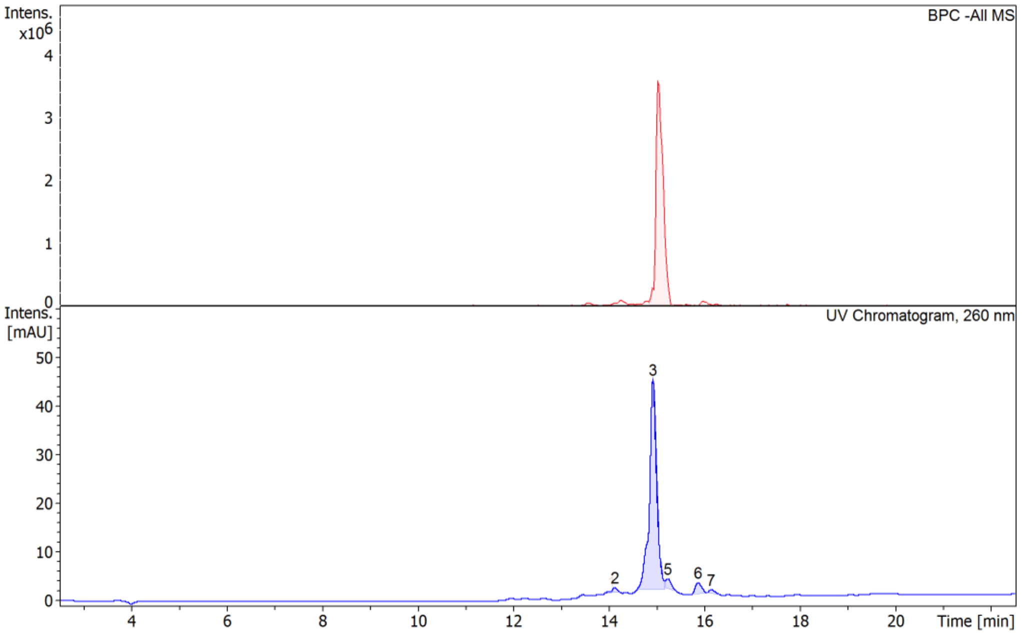

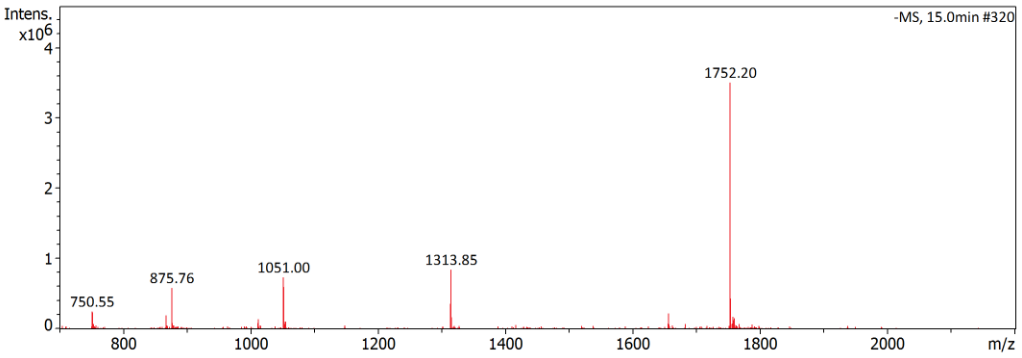

#	RT [min]	Chromatogram	Area	S/N	Area Frac. %
1	14.0	UV Chromatogram, 260 nm	2.3083	650294.1	0.45150
2	14.1	UV Chromatogram, 260 nm	6.6370	2133098.8	1.29820
3	14.9	UV Chromatogram, 260 nm	457.9228	90929304.0	89.56972
5	15.2	UV Chromatogram, 260 nm	16.9635	4320101.5	3.31806
6	15.9	UV Chromatogram, 260 nm	21.0077	4743981.5	4.10910
7	16.1	UV Chromatogram, 260 nm	6.4080	1629171.6	1.25341

Synthesis of DNA functionalized product 71

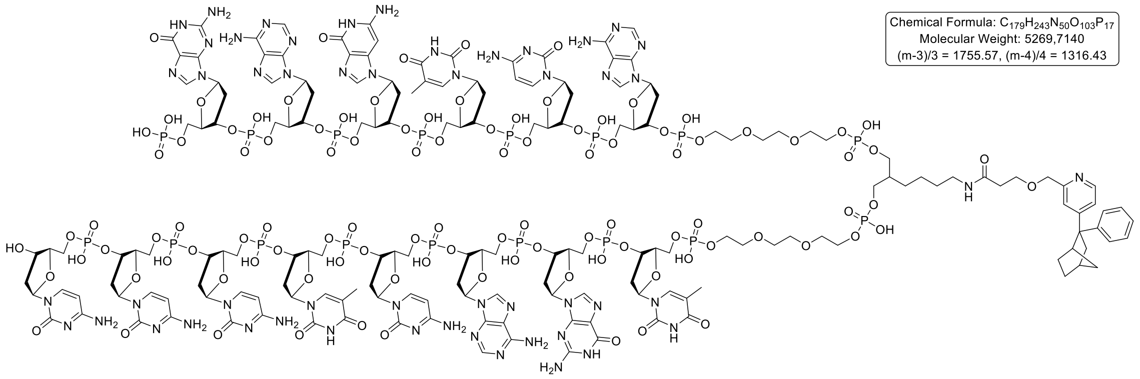

DNA functionalized product **71** was synthesized and purified according to general procedure H using 2-phenylbicyclo[2.2.1]heptan-2-yl 4-cyano-2-fluorobenzoate **S20** (5 µL of 50 mM stock solution in DMSO, 250 nmol, 100 equiv.), Hantzsch ester (5 µL of 50 mM stock solution in DMSO, 250 nmol, 100 equiv.), DMSO (30 µL), H2O (6.5 µL), sodium acetate (1 µL of 250 mM stock solution in H_2O, 250 nmol, 100 equiv.) and DNA derivative **60** (2.5 µL of 1 mM stock solution in H_2O, 2.5 nmol, 1 equiv.). Yield = 83%

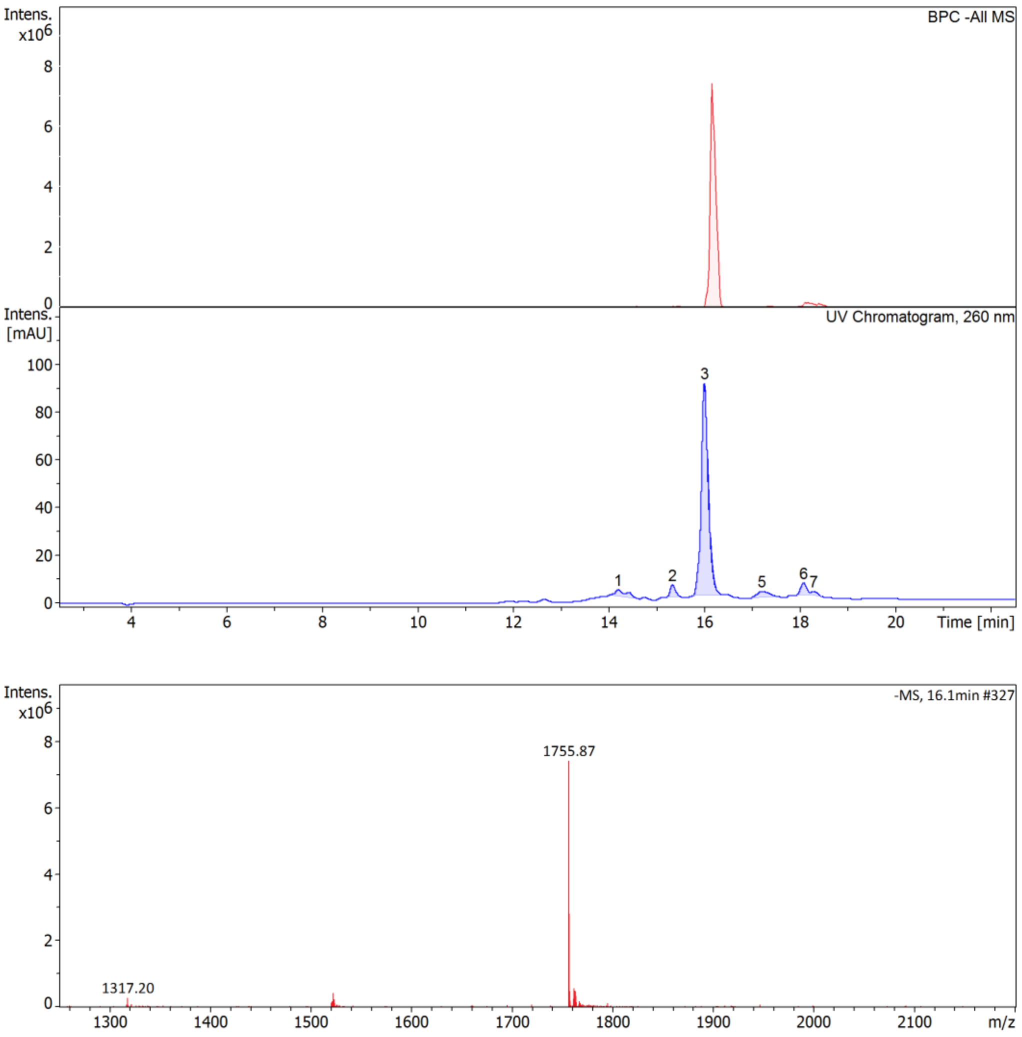

#	RT [min]	Chromatogram	Area	S/N	Area Frac. %
1	14.2	UV Chromatogram, 260 nm	45.236	2627.9	4.11747
2	15.3	UV Chromatogram, 260 nm	37.186	5020.2	3.38468
3	16.0	UV Chromatogram, 260 nm	910.617	89355.7	82.88568
5	17.2	UV Chromatogram, 260 nm	45.220	2681.2	4.11602
6	18.1	UV Chromatogram, 260 nm	44.933	5070.6	4.08986
7	18.3	UV Chromatogram, 260 nm	15.450	1710.9	1.40628

Synthesis of DNA functionalized product 72

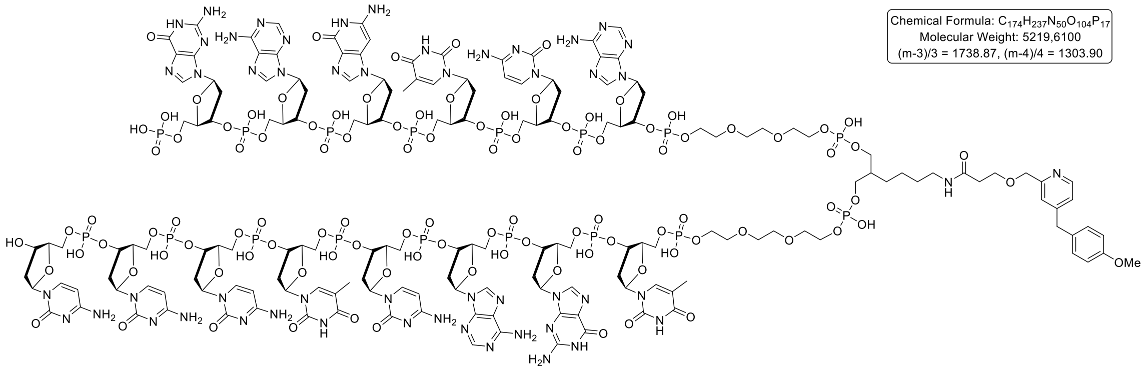

DNA functionalized product **72** was synthesized and purified according to general procedure H using 4-methoxybenzyl 4-cyano-2-fluorobenzoate **S27** (5 µL of 50 mM

stock solution in DMSO, 250 nmol, 100 equiv.), Hantzsch ester (5 μL of 50 mM stock solution in DMSO, 250 nmol, 100 equiv.), DMSO (30 μL), H2O (6.5 μL), sodium acetate (1 μL of 250 mM stock solution in H_2O, 250 nmol, 100 equiv.) and DNA derivative **60** (2.5 μL of 1 mM stock solution in H_2O, 2.5 nmol, 1 equiv.). Yield = 90%

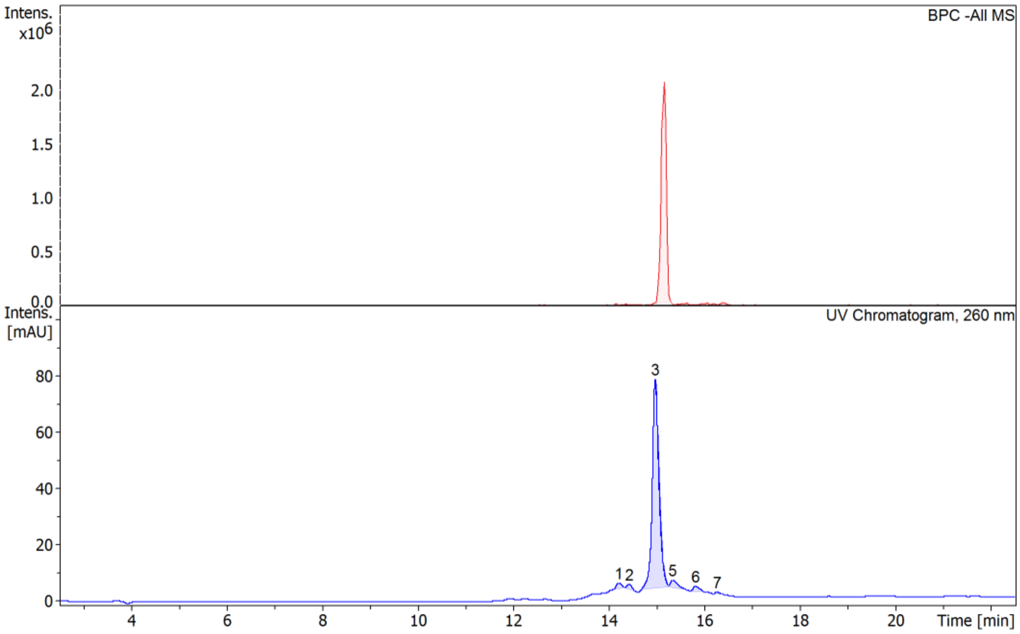

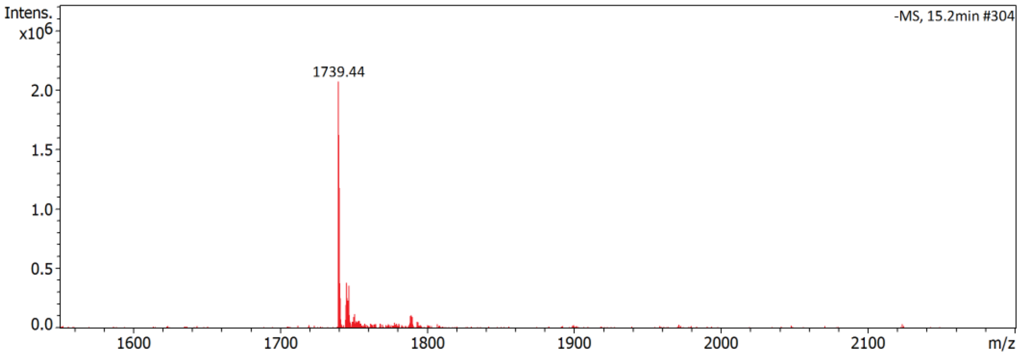

#	RT [min]	Chromatogram	Area	S/N	Area Frac. %
1	14.2	UV Chromatogram, 260 nm	14.3486	2016.6	1.81550
2	14.4	UV Chromatogram, 260 nm	12.8941	1786.8	1.63146
3	15.0	UV Chromatogram, 260 nm	714.0269	74460.5	90.34433
5	15.3	UV Chromatogram, 260 nm	28.2616	2659.1	3.57588
6	15.8	UV Chromatogram, 260 nm	15.8702	1792.7	2.00803
7	16.3	UV Chromatogram, 260 nm	4.9380	690.7	0.62479

Synthesis of DNA functionalized product 73

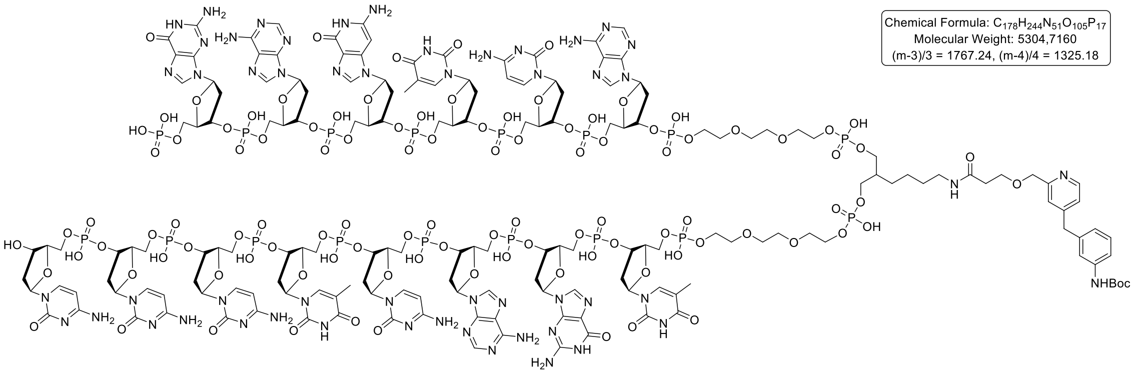

DNA functionalized product **73** was synthesized and purified according to general procedure H using 3-((*tert*-butoxycarbonyl)amino)benzyl 4-cyano-2-fluorobenzoate **S28** (5 µL of 50 mM stock solution in DMSO, 250 nmol, 100 equiv.), Hantzsch ester (5 µL of 50 mM stock solution in DMSO, 250 nmol, 100 equiv.), DMSO (30 µL), H2O (6.5 µL), sodium acetate (1 µL of 250 mM stock solution in H_2O, 250 nmol, 100 equiv.) and DNA derivative **60** (2.5 µL of 1 mM stock solution in H_2O, 2.5 nmol, 1 equiv.). Yield = 73%

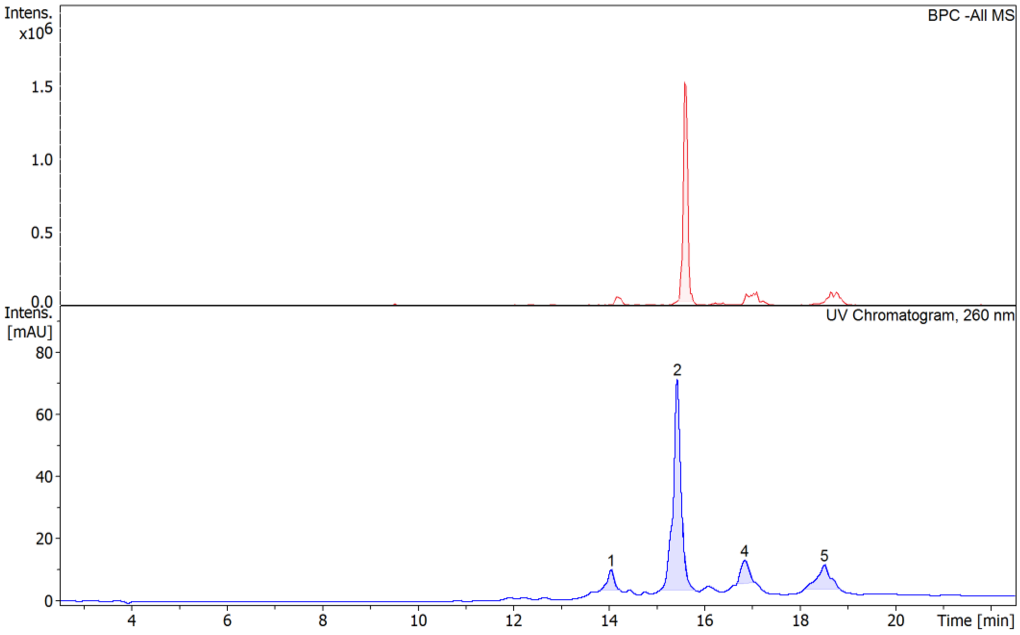

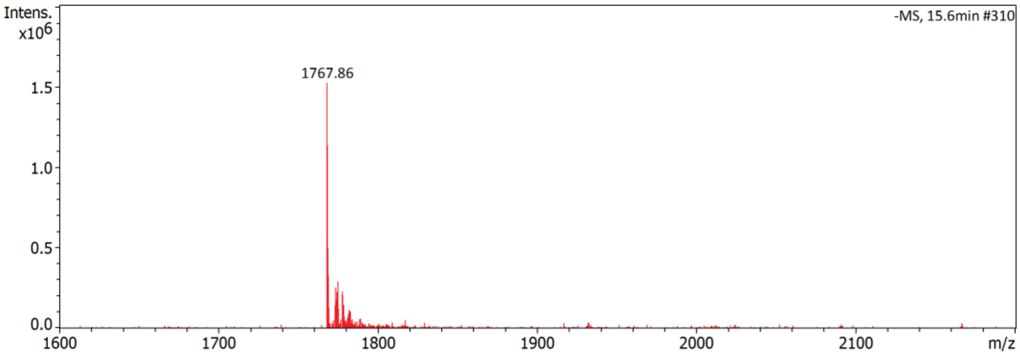

#	RT [min]	Chromatogram	Area	S/N	Area Frac. %
1	14.0	UV Chromatogram, 260 nm	61.797	6583.5	5.720
2	15.4	UV Chromatogram, 260 nm	784.668	68214.2	72.630
4	16.8	UV Chromatogram, 260 nm	85.351	7337.4	7.900
5	18.5	UV Chromatogram, 260 nm	148.550	7752.8	13.750

Synthesis of DNA functionalized product 74 (from Haloperidol)

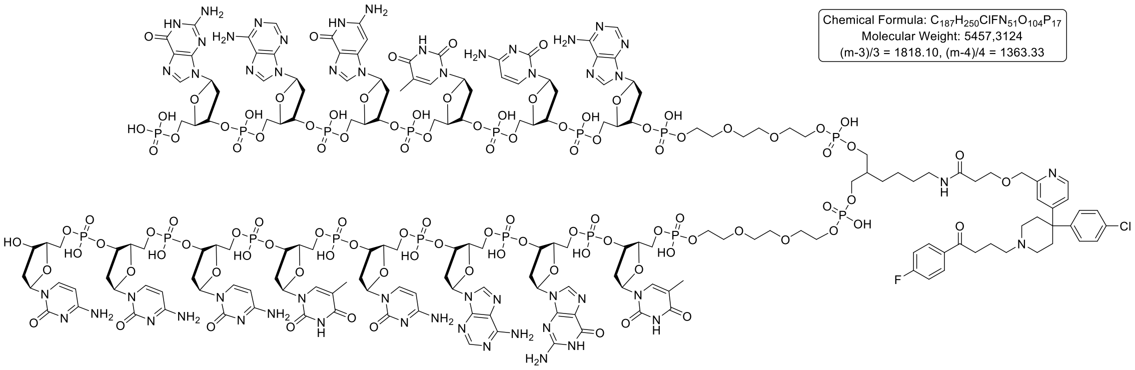

DNA functionalized product **74** was synthesized and purified according to general procedure H using 4-(4-chlorophenyl)-1-(4-(4-fluorophenyl)-4-oxobutyl) piperidin-4-yl-4-cyano-2-fluorobenzoate (Haloperidol ester) **S30** (5 µL of 50 mM stock solution in DMSO, 250 nmol, 100 equiv.), Hantzsch ester (5 µL of 50 mM stock solution in DMSO, 250 nmol, 100 equiv.), DMSO (30 µL), H2O (6.5 µL), sodium acetate (1 µL of 250 mM stock solution in H_2O, 250 nmol, 100 equiv.) and DNA derivative **60** (2.5 µL of 1 mM stock solution in H_2O, 2.5 nmol, 1 equiv.). Yield = 68%

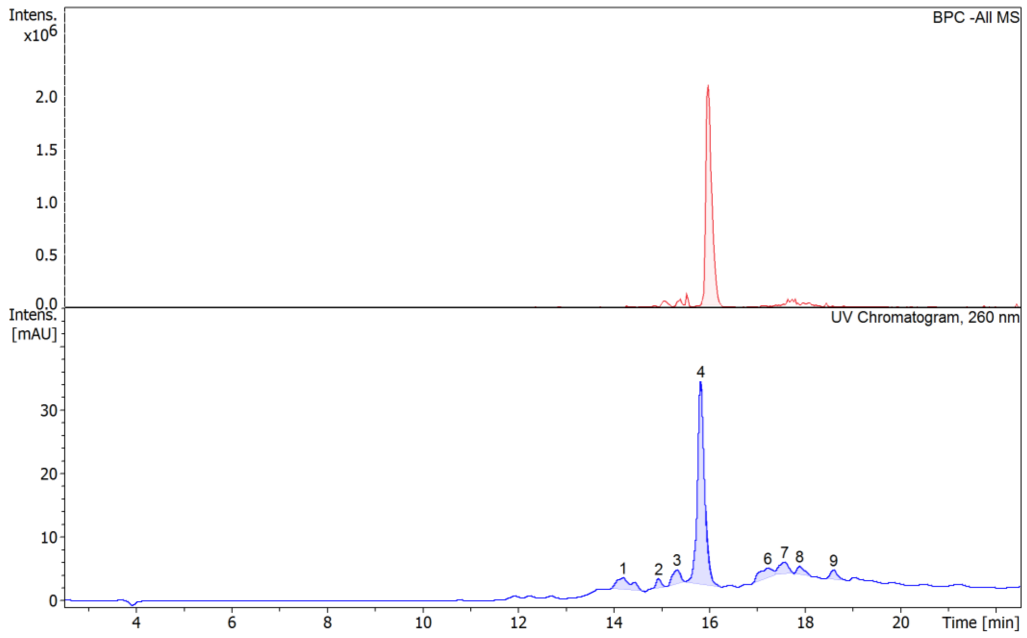

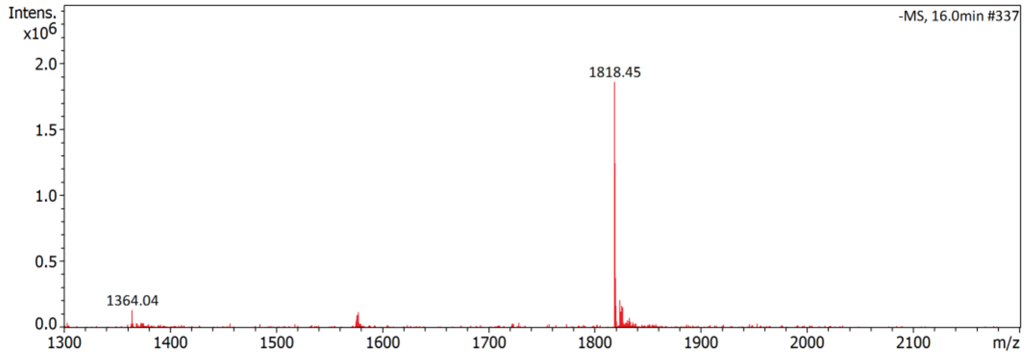

#	RT [min]	Chromatogram	Area	S/N	Area Frac. %
1	14.2	UV Chromatogram, 260 nm	39.634	1806.7	8.12886
2	14.9	UV Chromatogram, 260 nm	10.048	1429.5	2.06090
3	15.3	UV Chromatogram, 260 nm	25.003	2222.3	5.12820
4	15.8	UV Chromatogram, 260 nm	330.429	32044.7	67.77095
6	17.2	UV Chromatogram, 260 nm	30.875	1467.3	6.33254
7	17.6	UV Chromatogram, 260 nm	24.494	1797.8	5.02366
8	17.9	UV Chromatogram, 260 nm	12.976	1254.1	2.66135
9	18.6	UV Chromatogram, 260 nm	14.108	1452.7	2.89353

Synthesis of DNA functionalized product 75 (from OBn-Ezetimibe)

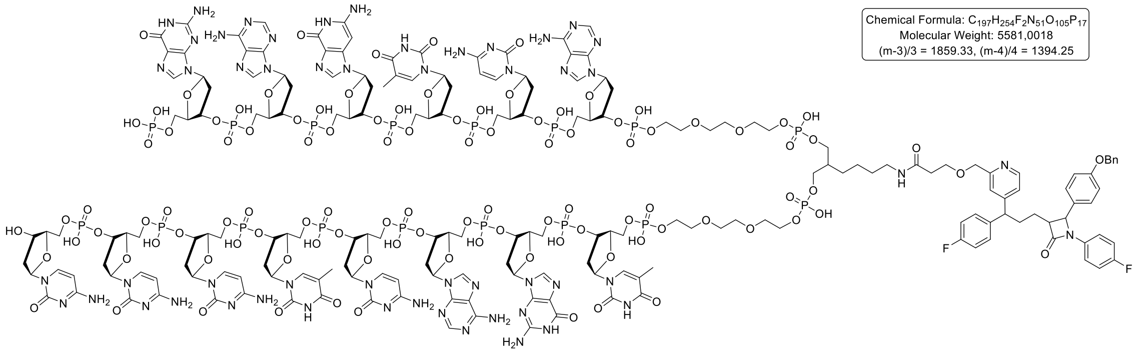

DNA functionalized product **75** was synthesized and purified according to general procedure H using 3-(2-(4-(benzyloxy)phenyl)-1-(4-fluorophenyl)-4-oxoazetidin-3-yl)-1-(4-fluorophenyl)propyl 4-cyano-2-fluorobenzoate (OBn-Ezetimibe ester) **S31** (5 μL of 50 mM stock solution in DMSO, 250 nmol, 100 equiv.), Hantzsch ester (5 μL of 50 mM stock solution in DMSO, 250 nmol, 100 equiv.), DMSO (30 μL), H2O (6.5 μL), sodium acetate (1 μL of 250 mM stock solution in H_2O, 250 nmol, 100 equiv.) and DNA derivative **60** (2.5 μL of 1 mM stock solution in H_2O, 2.5 nmol, 1 equiv.). Yield = 87%

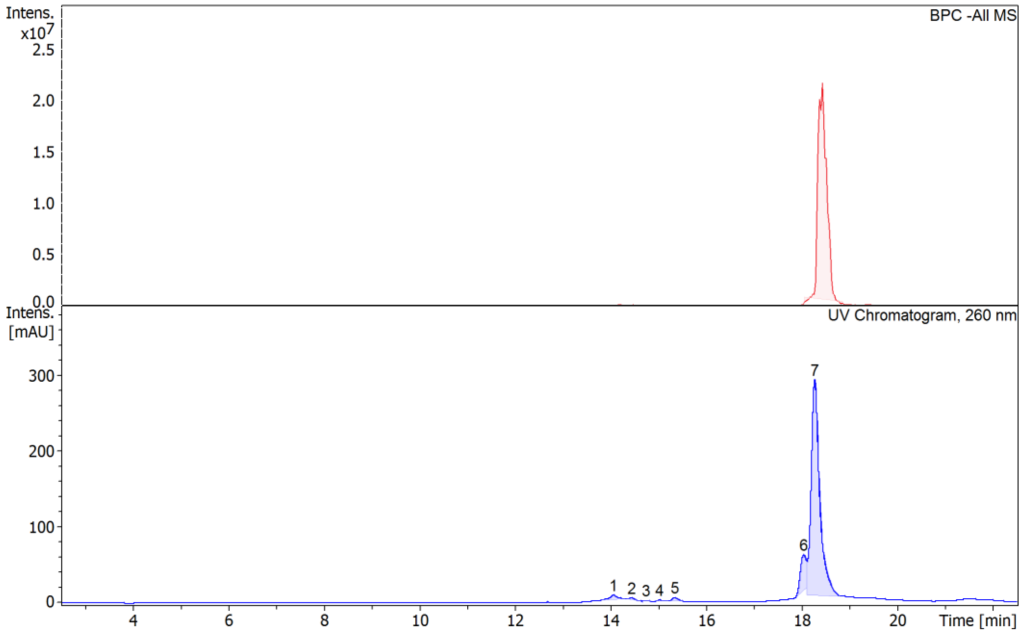

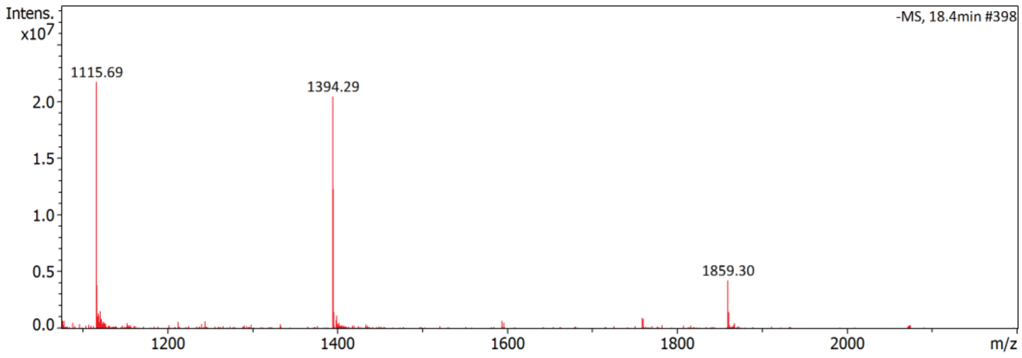

#	RT [min]	Chromatogram	Area	S/N	Area Frac. %
1	14.0	UV Chromatogram, 260 nm	72.5788	6289.2	1.768656
2	14.4	UV Chromatogram, 260 nm	19.5865	2572.0	0.477298
3	14.7	UV Chromatogram, 260 nm	5.9822	1010.3	0.145779
4	15.0	UV Chromatogram, 260 nm	14.2682	1505.6	0.347698
5	15.3	UV Chromatogram, 260 nm	37.3140	4724.4	0.909296
6	18.0	UV Chromatogram, 260 nm	391.3810	47189.4	9.537473
7	18.3	UV Chromatogram, 260 nm	3562.5024	285897.7	86.813800

6. NMR traces

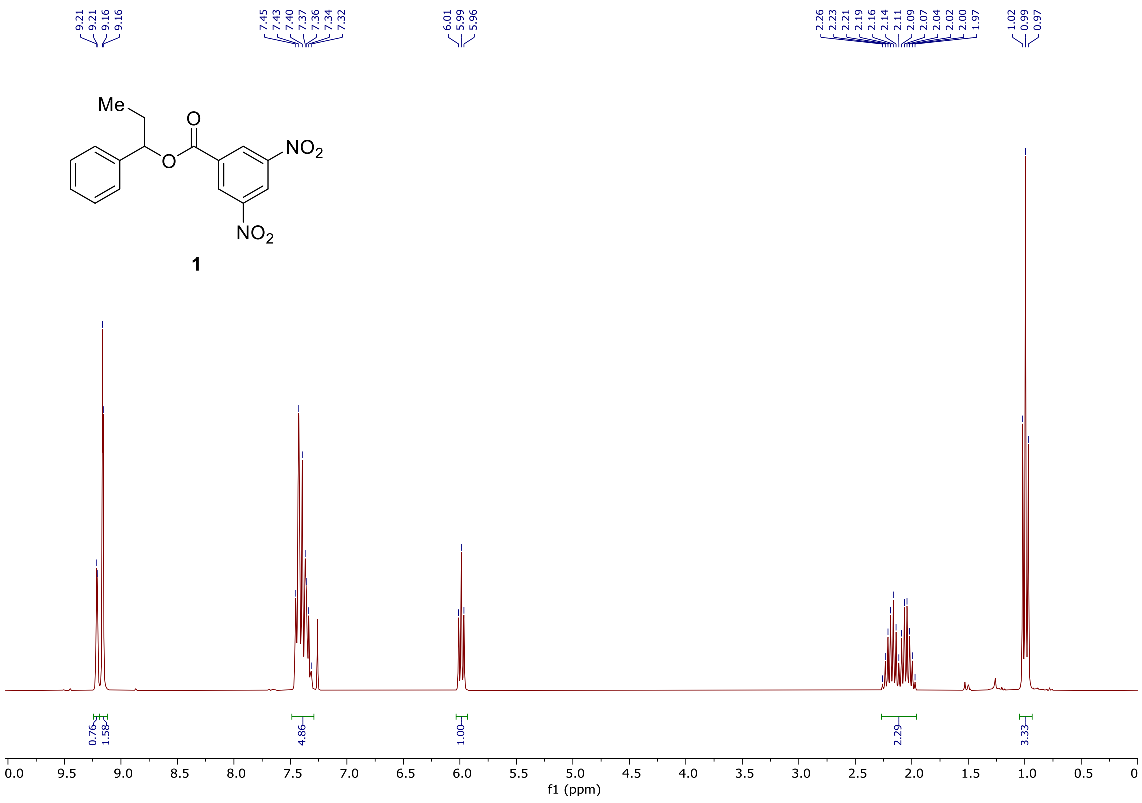
Me
O
O
NO_2
NO_2
1
9.21
9.21
9.16
9.16
7.45
7.43
7.40
7.37
7.36
7.34
7.32
6.01
5.99
5.96
2.26
2.23
2.21
2.19
2.16
2.14
2.11
2.09
2.07
2.04
2.02
2.00
1.97
1.02
0.99
0.97
0.76
1.58
4.86
1.00
2.29
3.33
f1 (ppm)

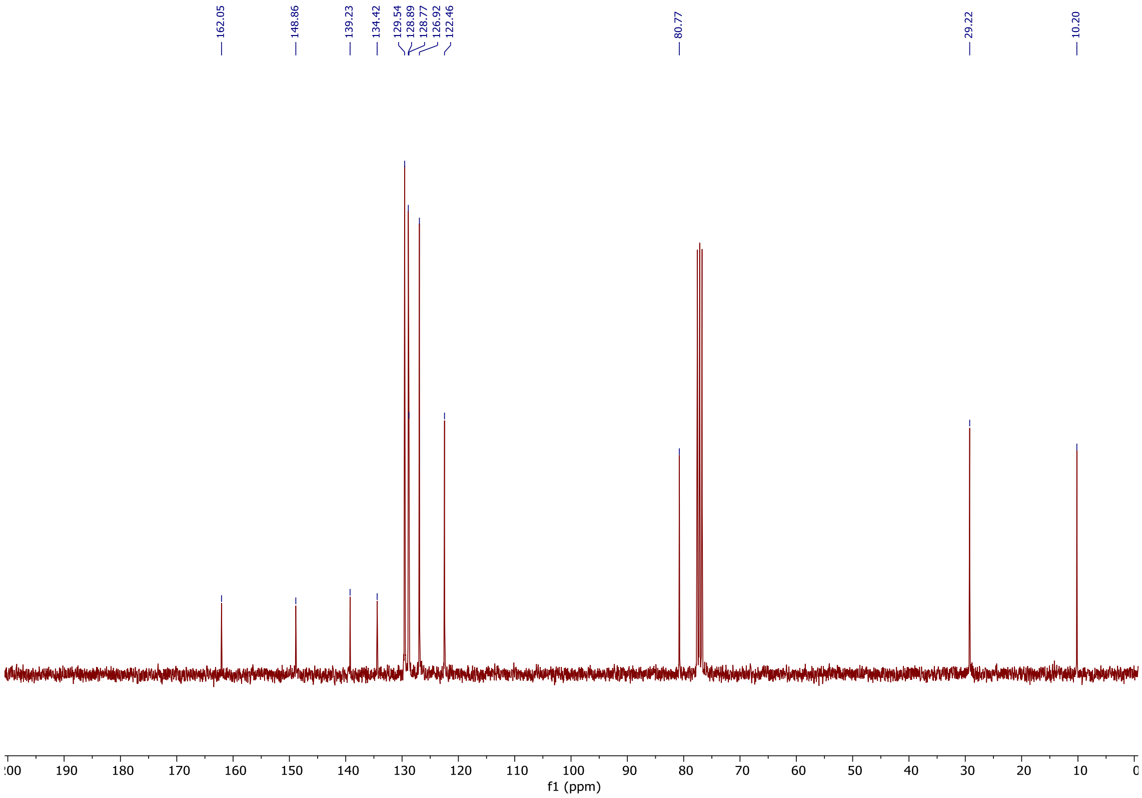
162.05
148.86
139.23
134.42
129.54
128.89
128.77
126.92
122.46
80.77
29.22
10.20
f1 (ppm)

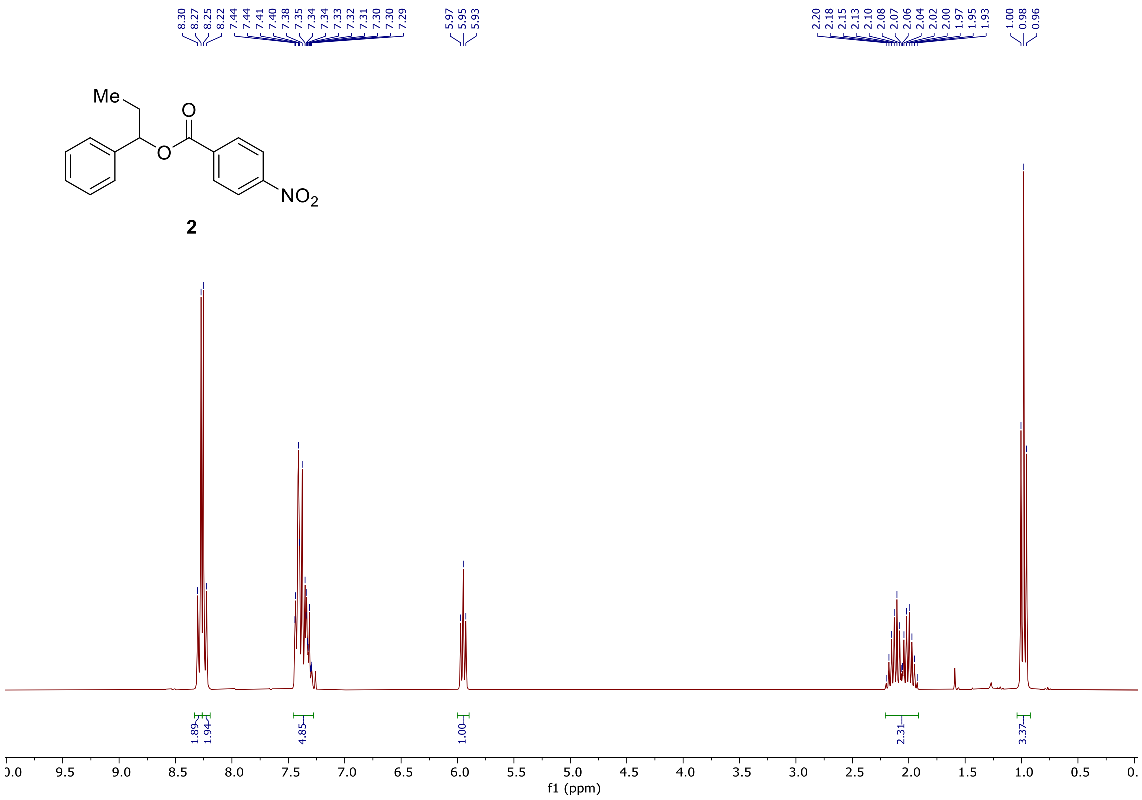

8.30
8.27
8.25
8.22
7.44
7.44
7.41
7.40
7.38
7.35
7.34
7.34
7.33
7.32
7.31
7.30
7.29
5.97
5.95
5.93
2.20
2.18
2.15
2.13
2.10
2.08
2.07
2.06
2.04
2.02
2.00
1.97
1.95
1.93
1.00
0.98
0.96
Me
O
O
NO2
2
1.89
1.94
4.85
1.00
2.31
3.37
9.5
9.0
8.5
8.0
7.5
7.0
6.5
6.0
5.5
5.0
4.5
4.0
3.5
3.0
2.5
2.0
1.5
1.0
0.5
f1 (ppm)

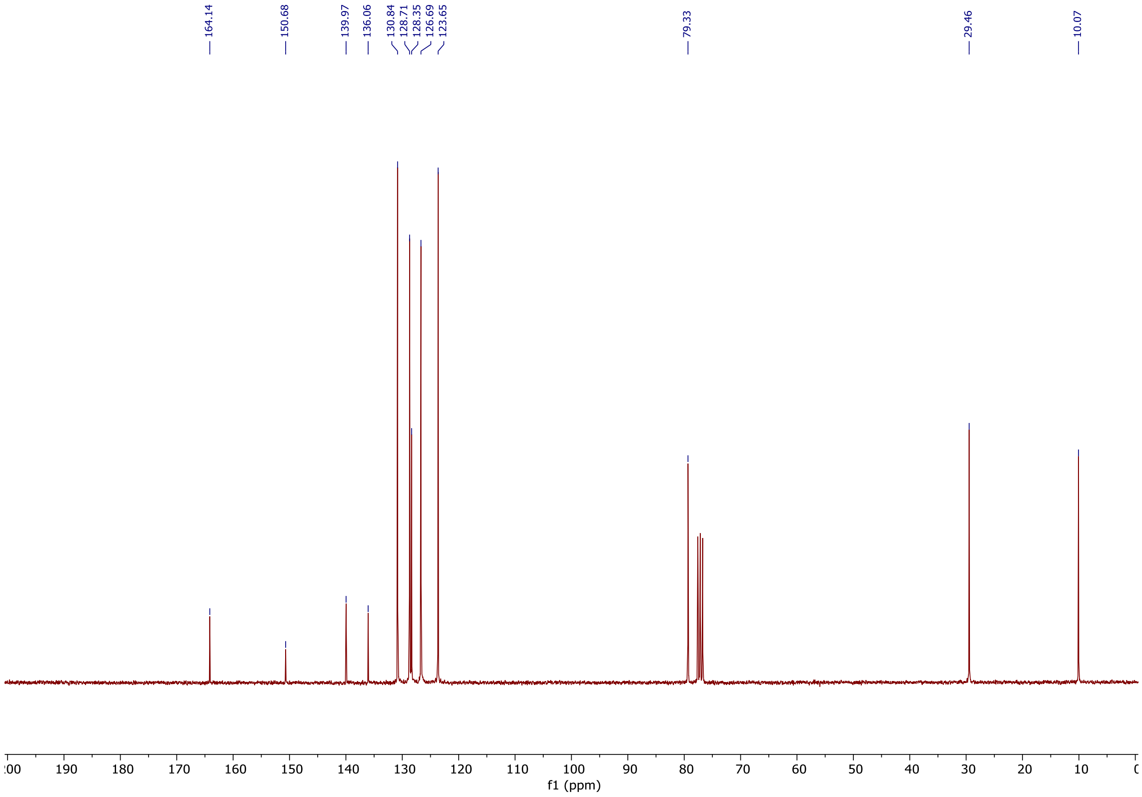

164.14
150.68
139.97
136.06
130.84
128.71
128.35
126.69
123.65
79.33
29.46
10.07
190
180
170
160
150
140
130
120
110
100
90
80
70
60
50
40
30
20
10
f1 (ppm)

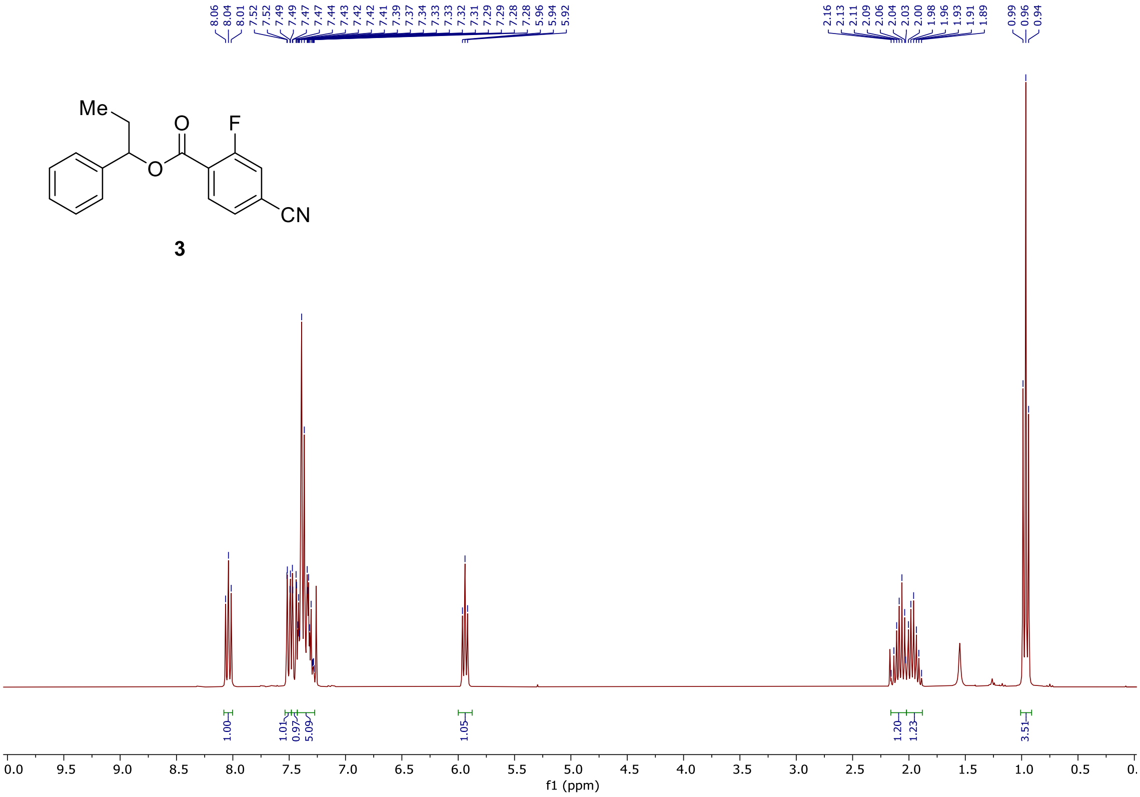
Me
O
F
O
CN
3
8.06
8.04
8.01
7.52
7.52
7.49
7.47
7.47
7.44
7.43
7.42
7.42
7.41
7.39
7.37
7.34
7.33
7.33
7.32
7.31
7.29
7.29
7.28
5.96
5.94
5.92
2.16
2.13
2.11
2.09
2.06
2.04
2.03
2.00
1.98
1.96
1.93
1.91
1.89
0.99
0.96
0.94
1.00
1.01
0.97
5.09
1.05
1.20
1.23
3.51
f1 (ppm)

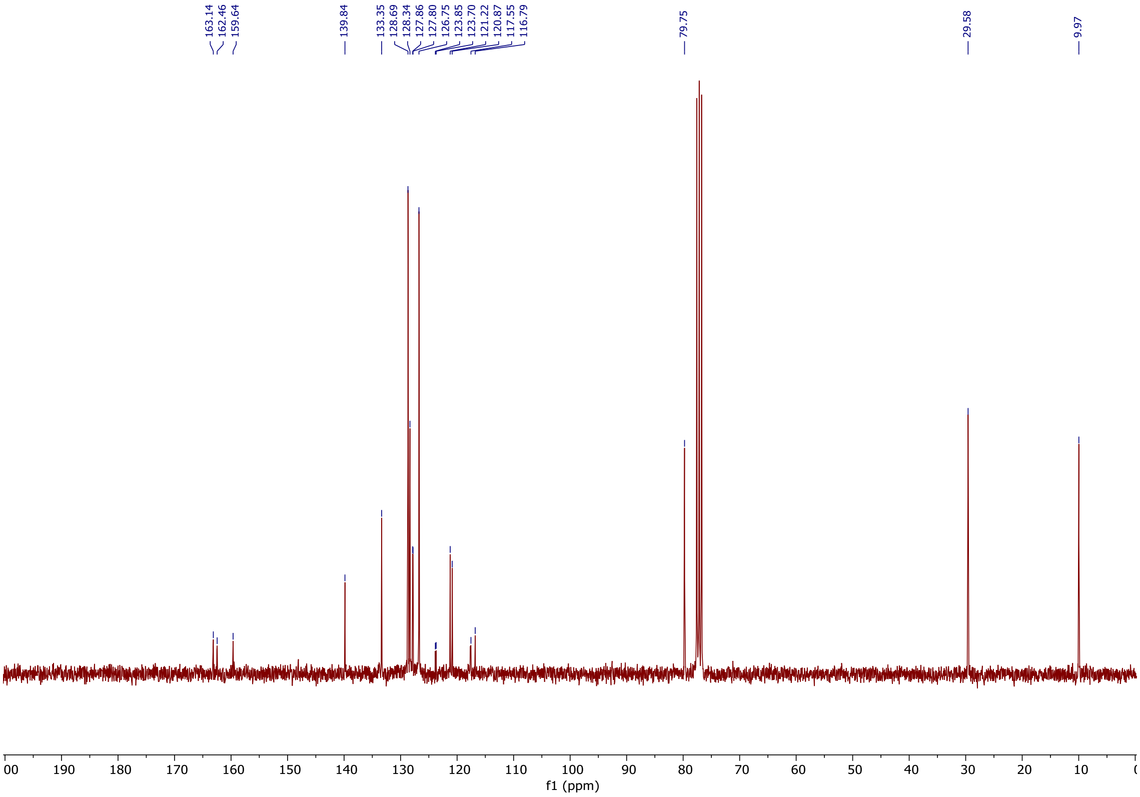
163.14
162.46
159.64
139.84
133.35
128.69
128.34
127.86
127.80
126.75
123.85
123.70
121.22
120.87
117.55
116.79
79.75
29.58
9.97
f1 (ppm)

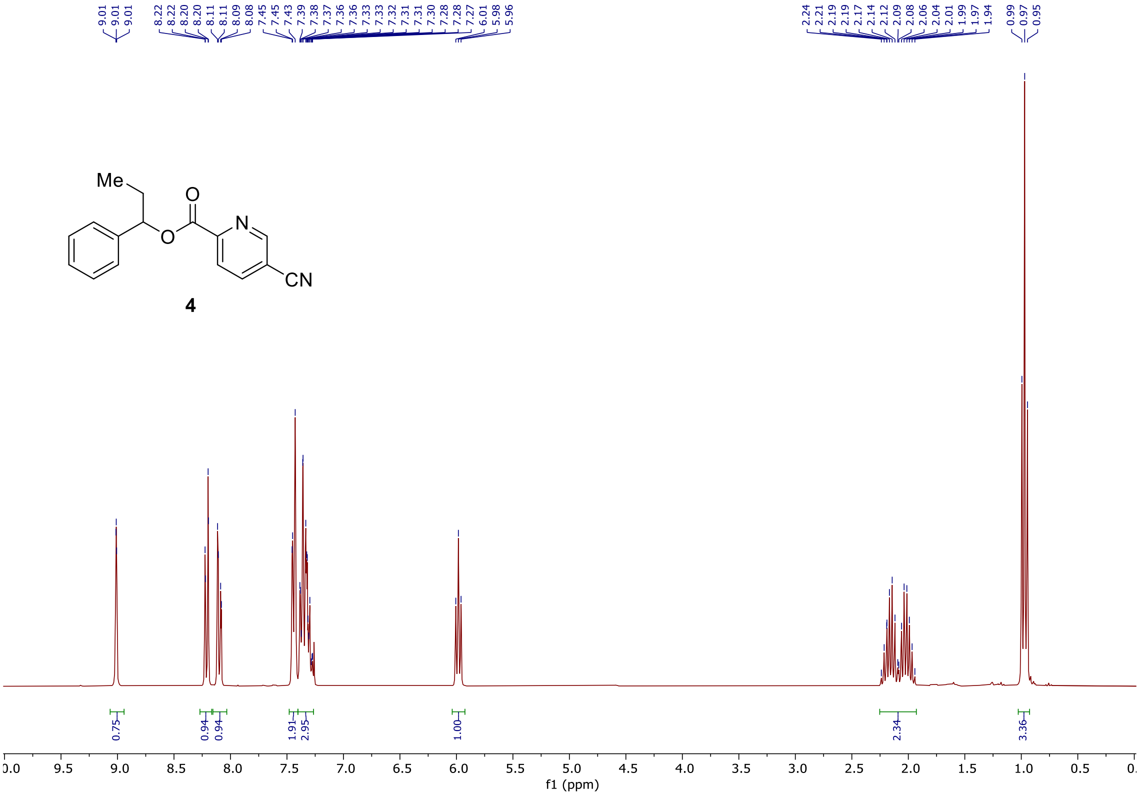
9.01 9.01 9.01 8.22 8.22 8.20 8.20 8.11 8.11 8.09 8.08 7.45 7.45 7.43 7.39 7.38 7.37 7.36 7.36 7.33 7.33 7.32 7.31 7.31 7.30 7.28 7.28 7.27 6.01 5.98 5.96
2.24 2.21 2.19 2.17 2.14 2.12 2.09 2.08 2.06 2.04 2.01 1.99 1.97 1.94 0.99 0.97 0.95
Me
O
N
CN
4
0.75 0.94 0.94 1.91 2.95 1.00 2.34 3.36
9.5 9.0 8.5 8.0 7.5 7.0 6.5 6.0 5.5 5.0 4.5 4.0 3.5 3.0 2.5 2.0 1.5 1.0 0.5
f1 (ppm)

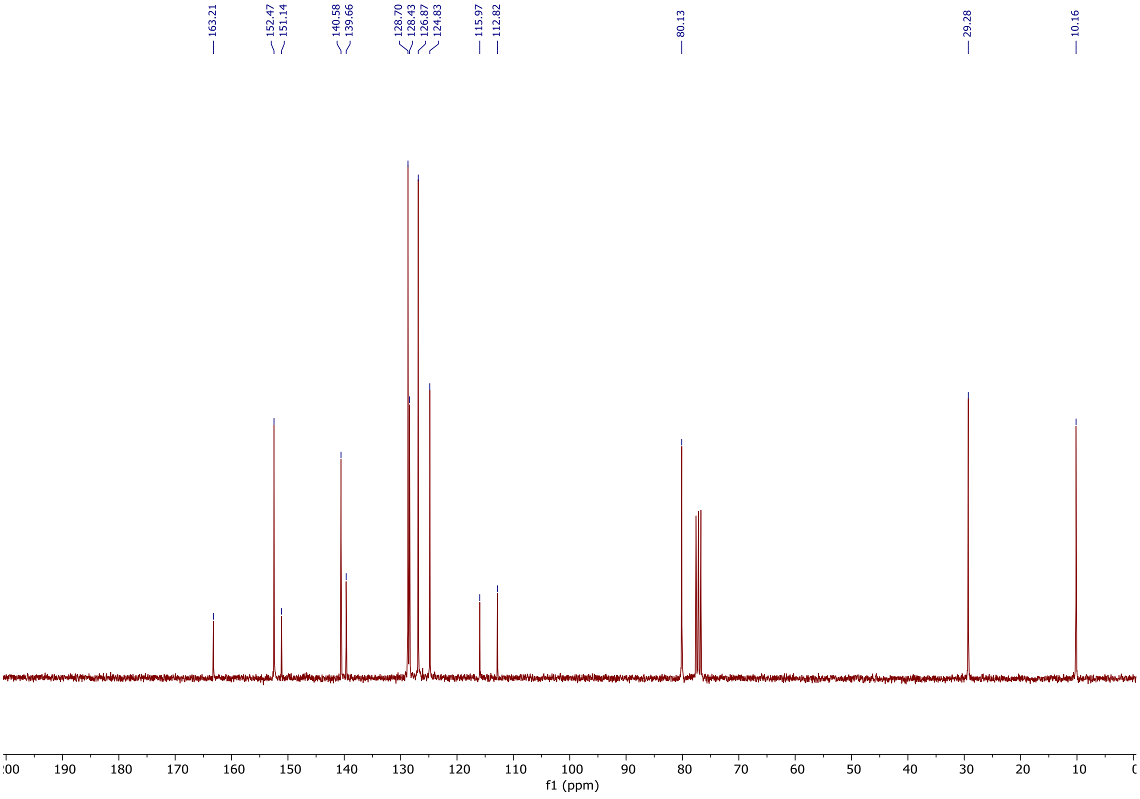
163.21 152.47 151.14 140.58 139.66 128.70 128.43 126.87 124.83 115.97 112.82 80.13 29.28 10.16
190 180 170 160 150 140 130 120 110 100 90 80 70 60 50 40 30 20 10
f1 (ppm)

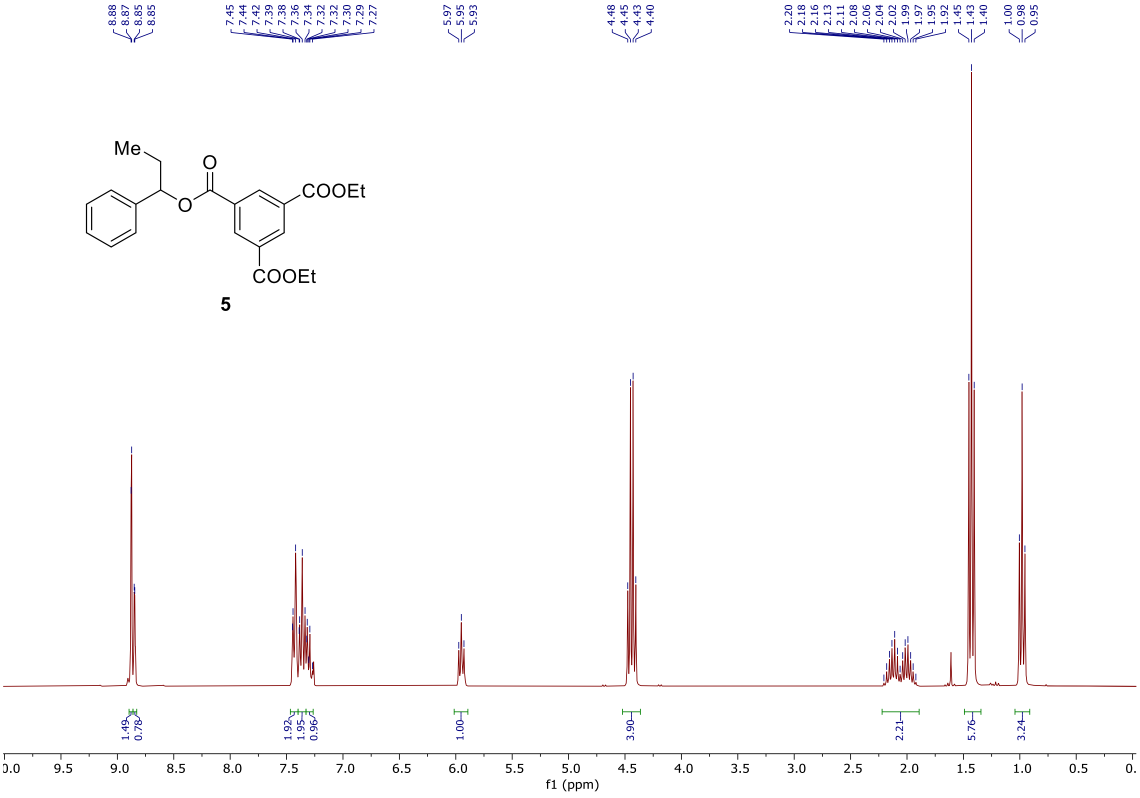
8.88
8.87
8.85
8.85
7.45
7.44
7.42
7.39
7.38
7.36
7.34
7.32
7.32
7.30
7.29
7.27
5.97
5.95
5.93
4.48
4.45
4.43
4.40
2.20
2.18
2.16
2.13
2.11
2.08
2.06
2.04
2.02
1.99
1.97
1.95
1.92
1.45
1.43
1.40
1.00
0.98
0.95
Me
O
O
COOEt
COOEt
5
1.49
0.78
1.92
1.95
0.96
1.00
3.90
2.21
5.76
3.24
f1 (ppm)

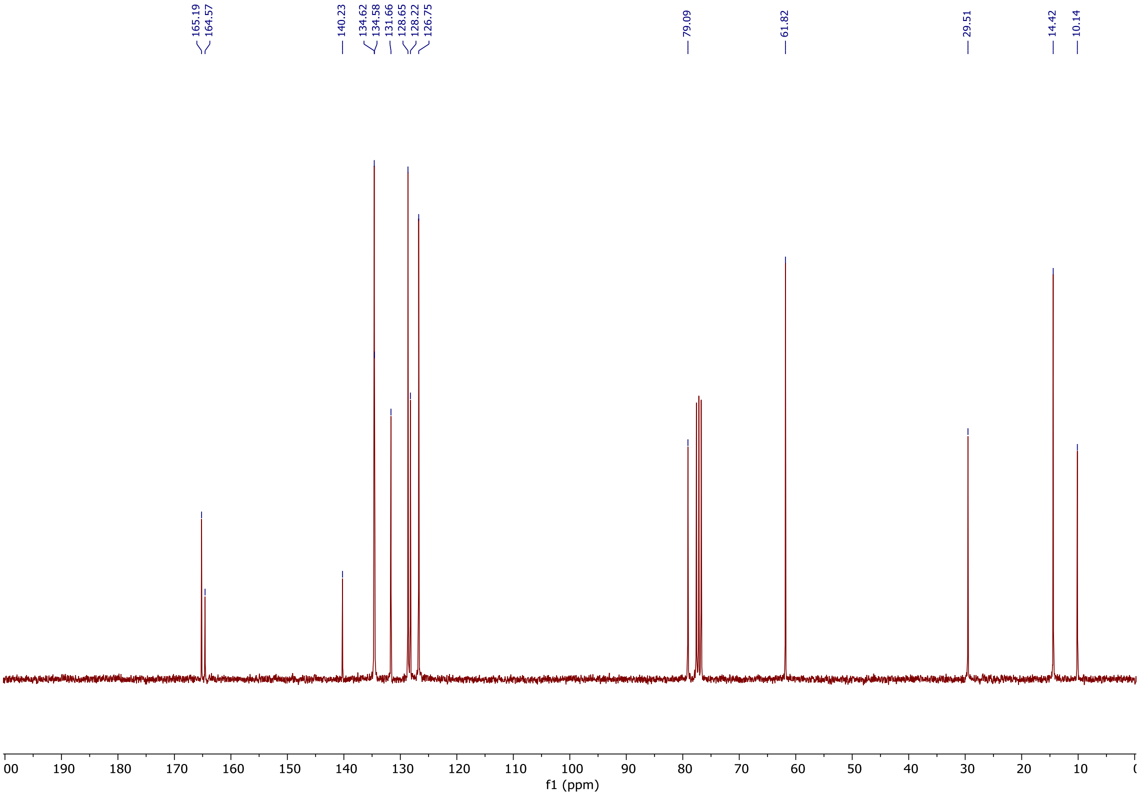
165.19
164.57
140.23
134.62
134.58
131.66
128.65
128.22
126.75
79.09
61.82
29.51
14.42
10.14
f1 (ppm)

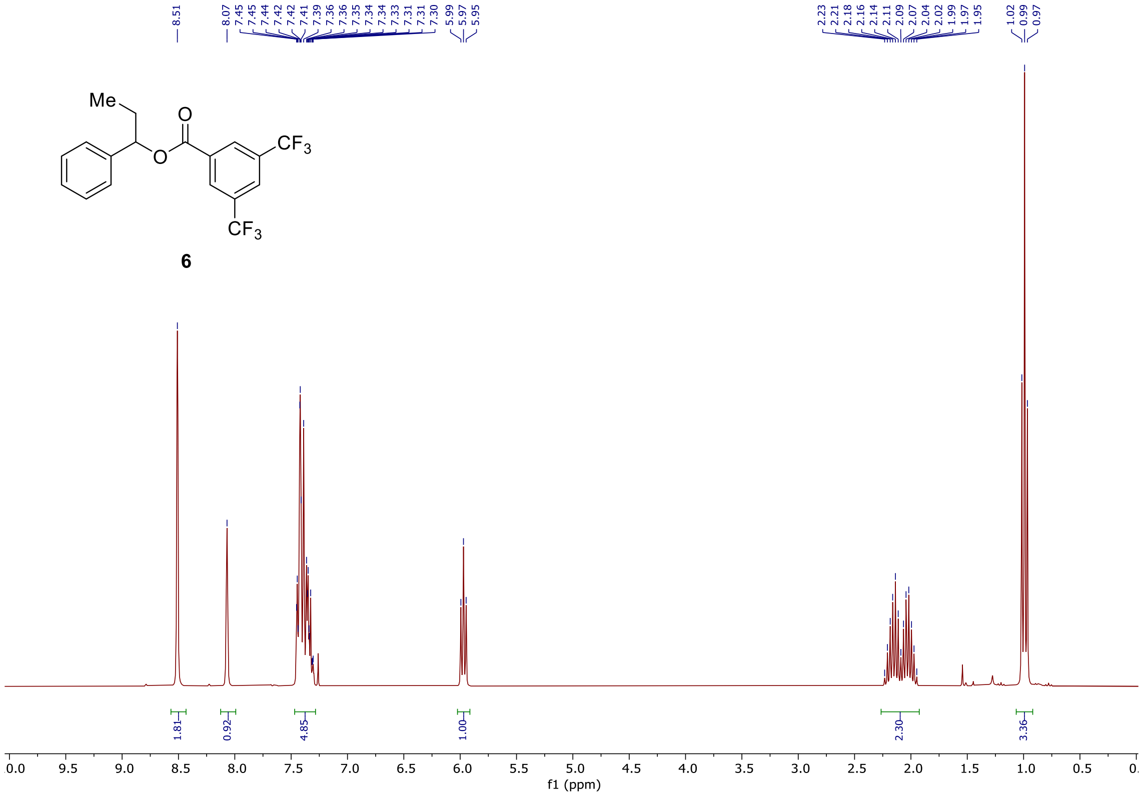
8.51
8.07 7.45 7.44 7.42 7.42 7.41 7.39 7.36 7.36 7.35 7.34 7.34 7.33 7.31 7.31 7.30 5.99 5.97 5.95
2.23 2.21 2.18 2.16 2.14 2.11 2.09 2.07 2.04 2.02 1.99 1.97 1.95
1.02 0.99 0.97
Me
O
O
CF3
CF3
6
1.81 0.92 4.85 1.00 2.30 3.36
f1 (ppm)

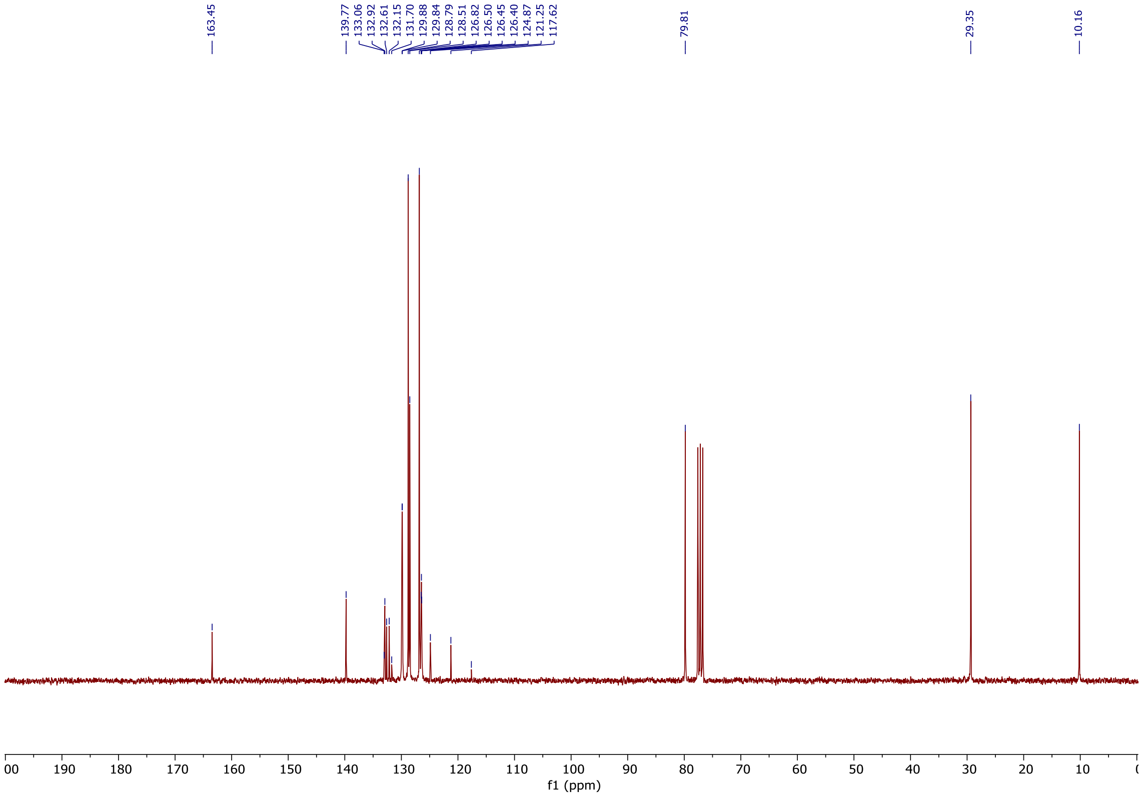
163.45
139.77 133.06 132.92 132.61 132.15 131.70 129.88 129.84 128.79 128.51 126.82 126.50 126.45 126.40 124.87 121.25 117.62
79.81
29.35
10.16
f1 (ppm)

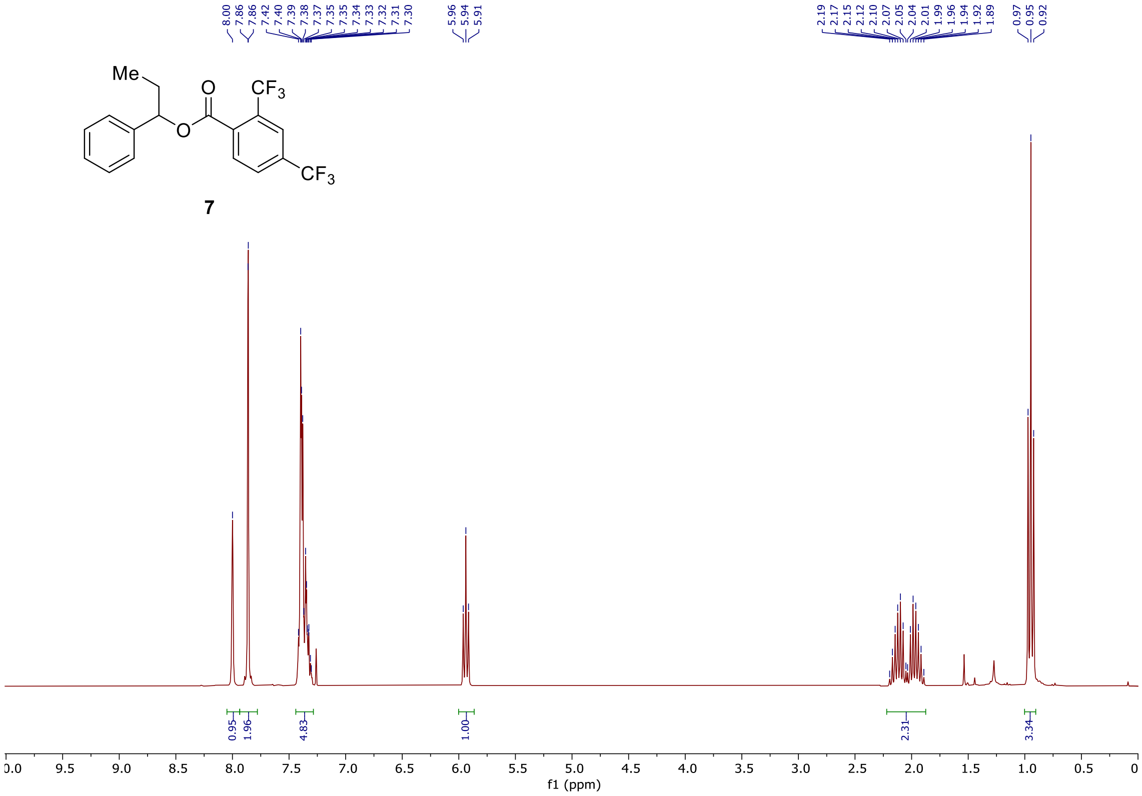
Me
O
CF3
O
CF3
7
8.00
7.86
7.86
7.42
7.40
7.39
7.38
7.37
7.35
7.35
7.34
7.33
7.32
7.31
7.30
5.96
5.94
5.91
2.19
2.17
2.15
2.12
2.10
2.07
2.05
2.04
2.01
1.99
1.96
1.94
1.92
1.89
0.97
0.95
0.92
0.95
1.96
4.83
1.00
2.31
3.34
f1 (ppm)

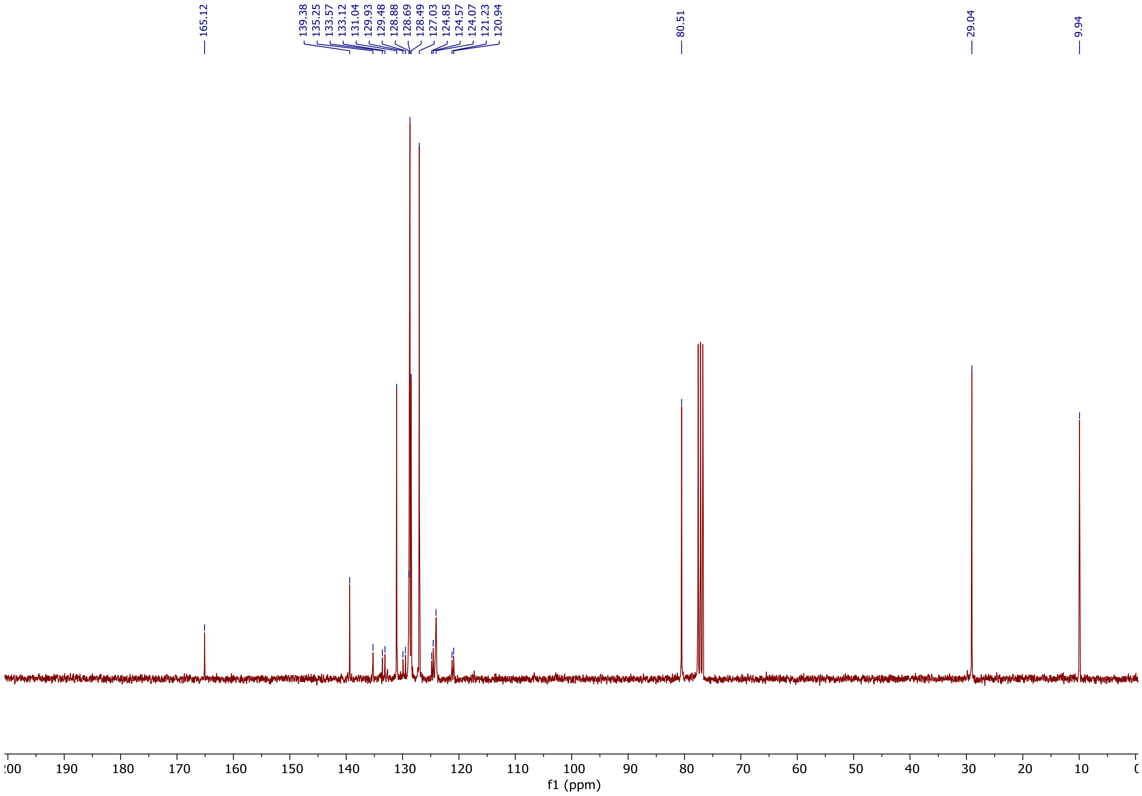
165.12
139.38
135.25
133.57
133.12
131.04
129.93
129.48
128.88
128.69
128.49
127.03
124.85
124.57
124.07
121.23
120.94
80.51
29.04
9.94
f1 (ppm)

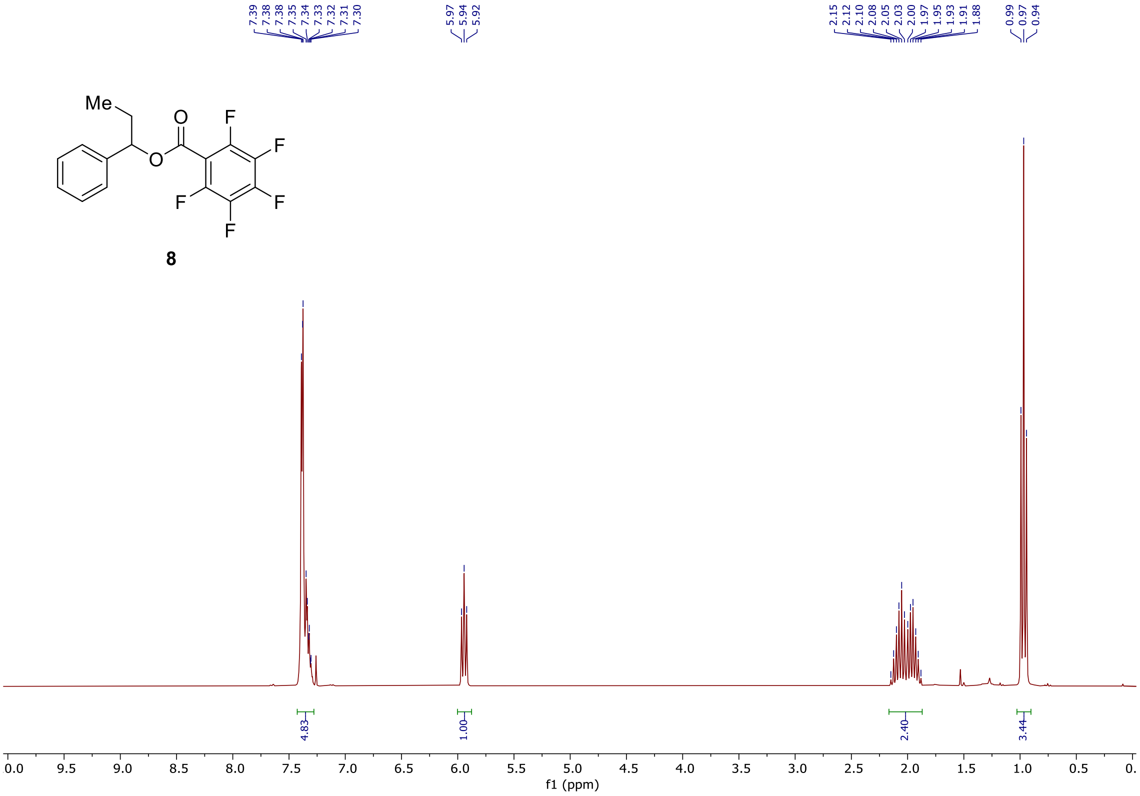
7.39 7.38 7.38 7.35 7.34 7.33 7.32 7.31 7.30
5.97 5.94 5.92
2.15 2.12 2.10 2.08 2.05 2.03 2.00 1.97 1.95 1.93 1.91 1.88
0.99 0.97 0.94
Me
O
O
F
F
F
F
F
8
4.83
1.00
2.40
3.44
f1 (ppm)

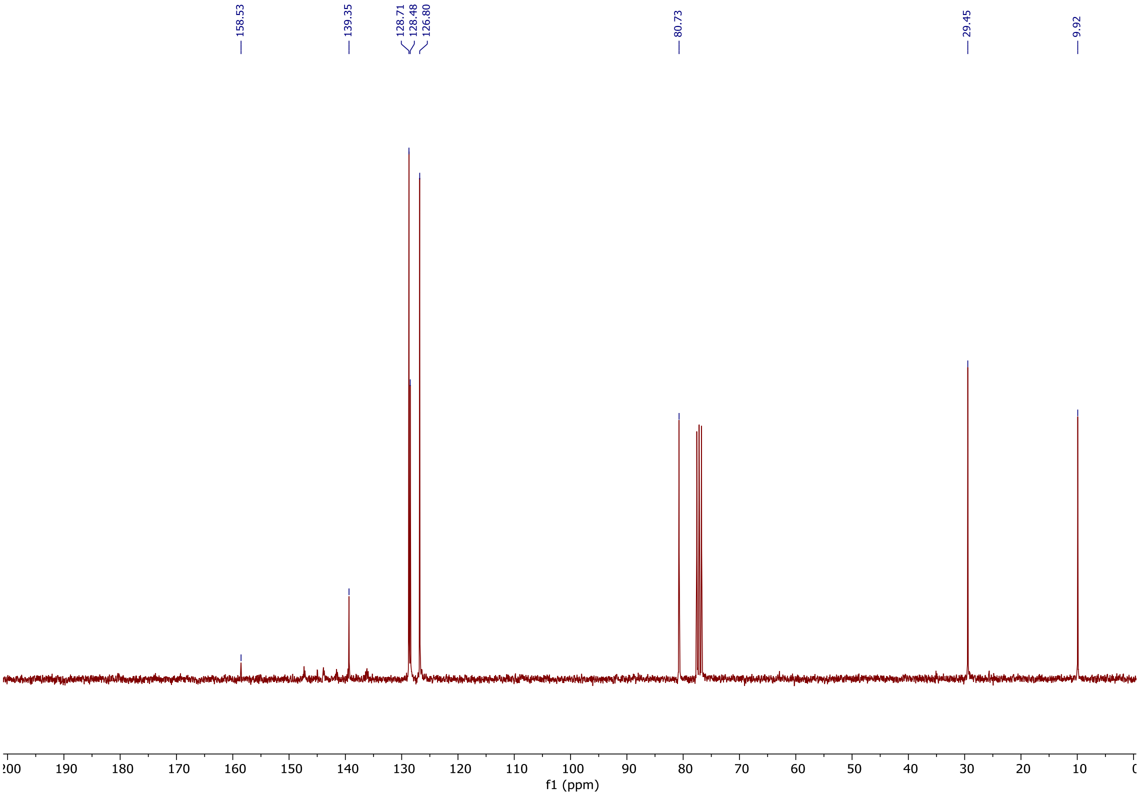
158.53
139.35
128.71 128.48 126.80
80.73
29.45
9.92
f1 (ppm)

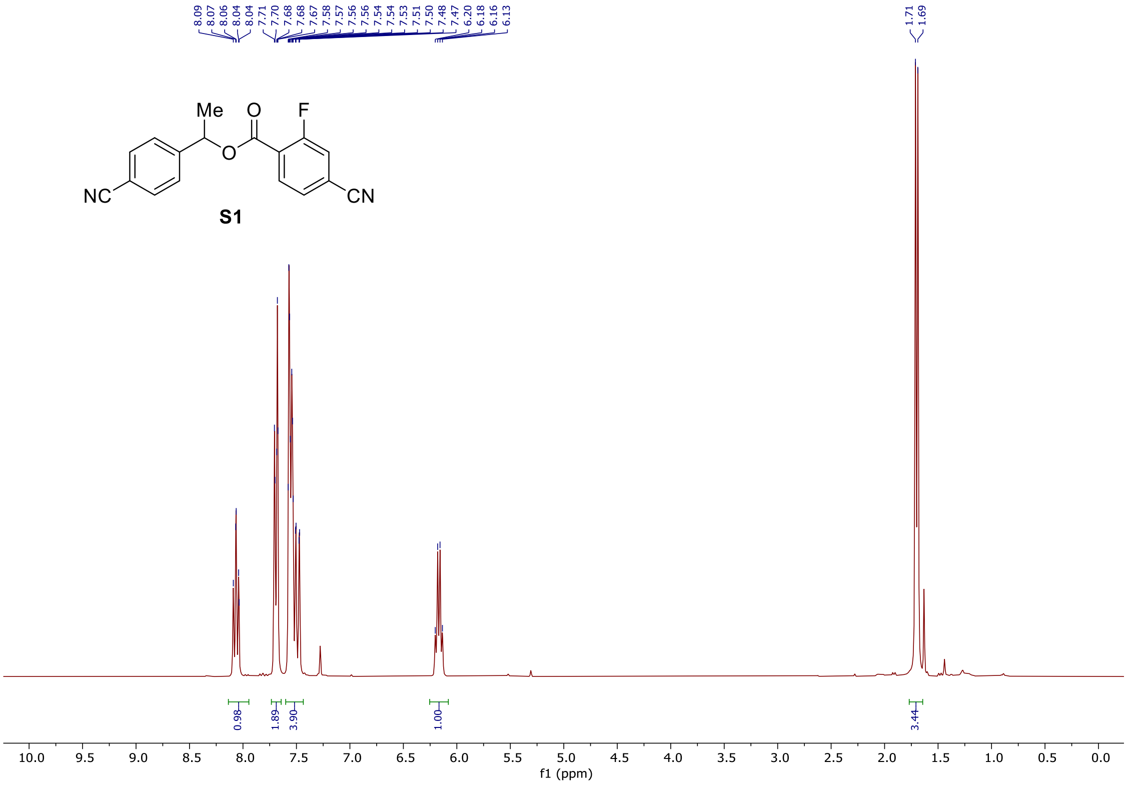

8.09
8.07
8.06
8.04
8.04
7.71
7.70
7.68
7.68
7.67
7.58
7.57
7.56
7.56
7.54
7.54
7.53
7.51
7.50
7.48
7.47
6.20
6.18
6.16
6.13
1.71
1.69
Me
O
F
O
NC
CN
S1
0.98
1.89
3.90
1.00
3.44
10.0
9.5
9.0
8.5
8.0
7.5
7.0
6.5
6.0
5.5
5.0
4.5
4.0
3.5
3.0
2.5
2.0
1.5
1.0
0.5
0.0
f1 (ppm)

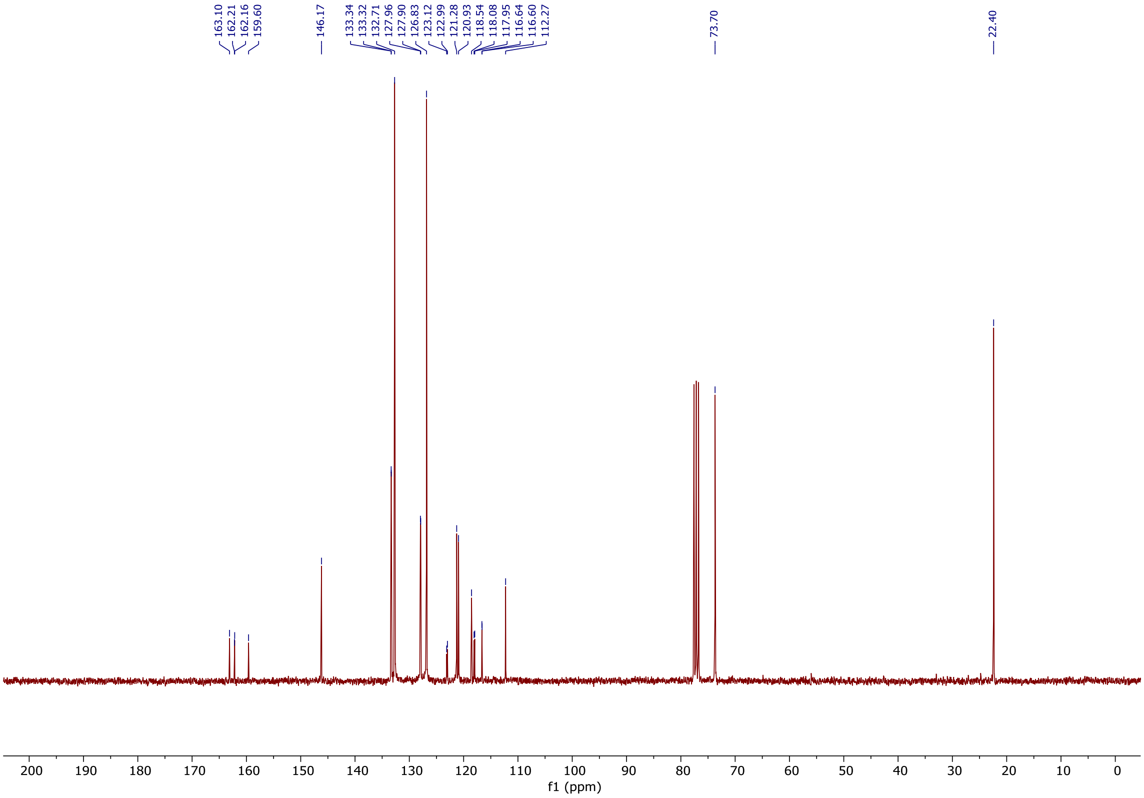

163.10
162.21
162.16
159.60
146.17
133.34
133.32
132.71
127.96
127.90
126.83
123.12
122.99
121.28
120.93
118.54
118.08
117.95
116.64
116.60
112.27
73.70
22.40
200
190
180
170
160
150
140
130
120
110
100
90
80
70
60
50
40
30
20
10
0
f1 (ppm)

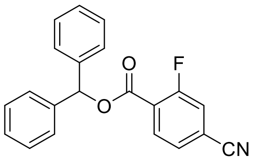
O
F
O
CN

S2

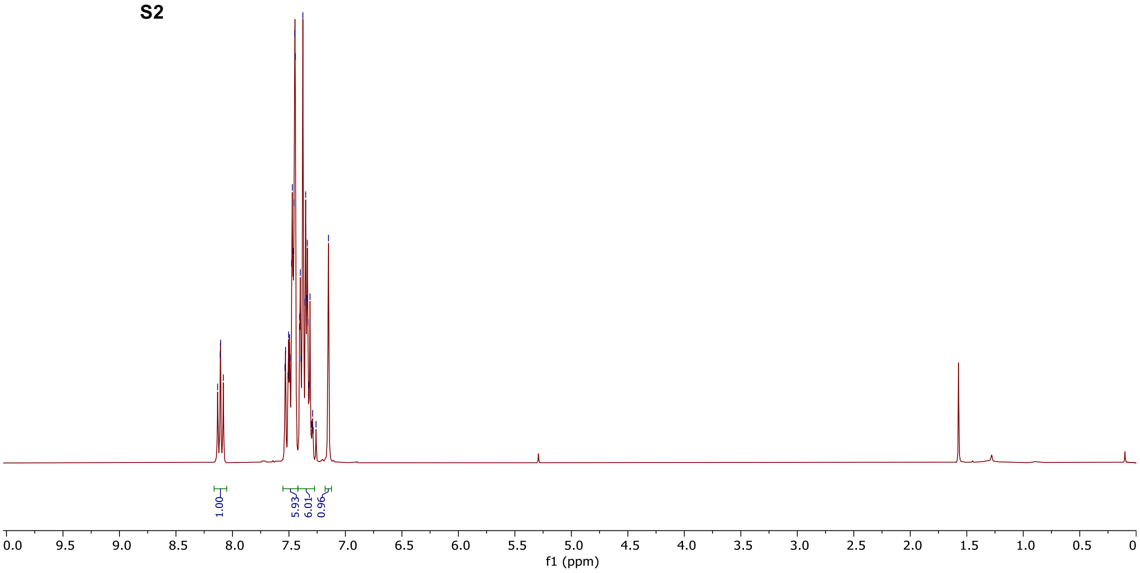
8.13
8.11
8.10
8.08
7.53
7.53
7.51
7.50
7.49
7.49
7.48
7.47
7.46
7.45
7.45
7.44
7.41
7.40
7.39
7.38
7.36
7.35
7.34
7.34
7.33
7.32
7.31
7.30
7.29
7.29
7.29
7.26
7.15
1.00
5.93
6.01
0.96
f1 (ppm)

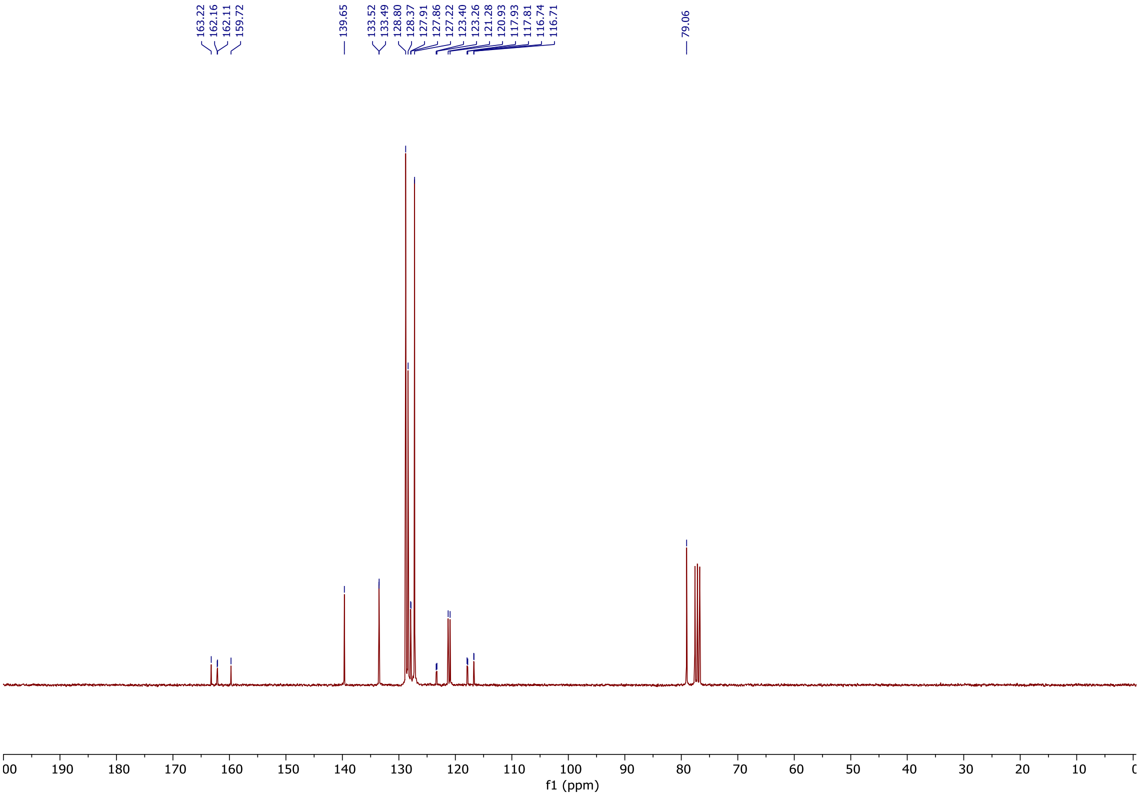
163.22
162.16
162.11
159.72
139.65
133.52
133.49
128.80
128.37
127.91
127.86
127.22
123.40
123.26
121.28
120.93
117.93
117.81
116.74
116.71
79.06
f1 (ppm)

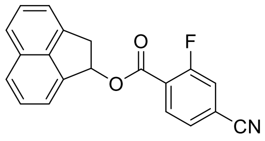
O
F
O
CN

S3

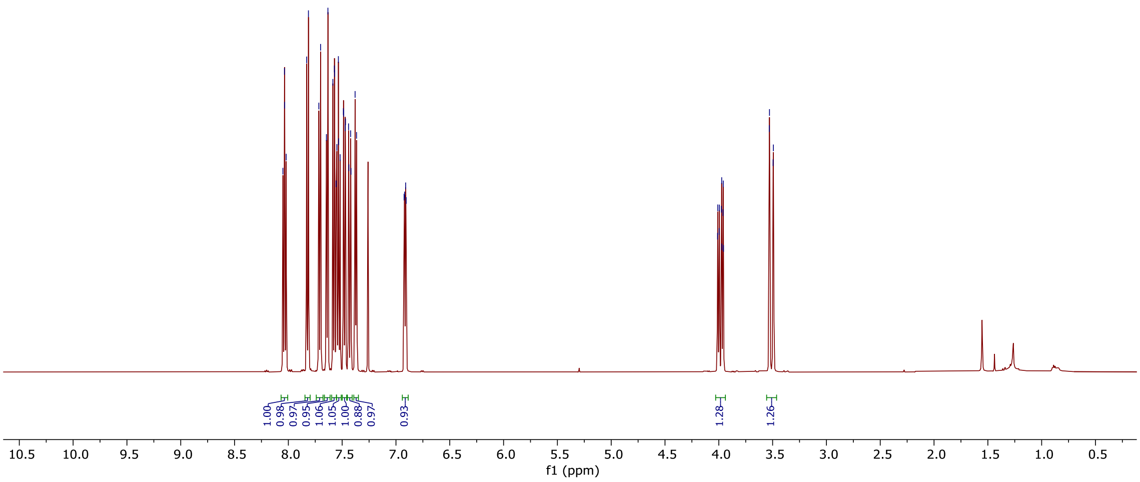
8.05 8.04 8.03 8.02 7.83 7.81 7.72 7.70 7.65 7.63 7.59 7.57 7.57 7.56 7.55 7.54 7.53 7.52 7.49 7.49 7.47 7.47 7.44 7.44 7.42 7.42 7.38 7.37 6.92 6.92 6.91 6.91
4.01 4.01 4.00 3.99 3.98 3.97 3.97 3.96 3.96 3.96 3.53 3.53 3.50 3.49
1.00 0.98 0.97 0.95 1.06 1.05 1.00 0.88 0.97 0.93 1.28 1.26
10.5 10.0 9.5 9.0 8.5 8.0 7.5 7.0 6.5 6.0 5.5 5.0 4.5 4.0 3.5 3.0 2.5 2.0 1.5 1.0 0.5
f1 (ppm)

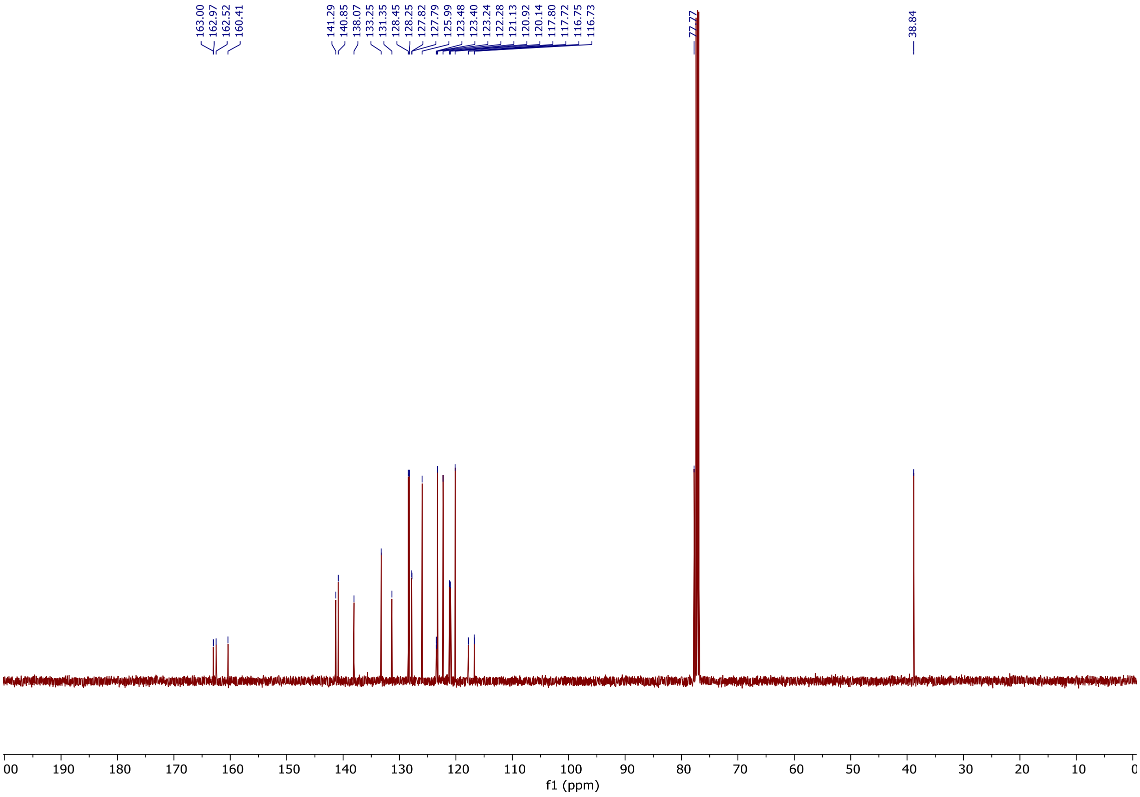
163.00 162.97 162.52 160.41
141.29 140.85 138.07 133.25 131.35 128.45 128.25 127.82 127.79 125.99 123.48 123.40 123.24 122.28 121.13 120.92 120.14 117.80 117.72 116.75 116.73
77.77
38.84
00 190 180 170 160 150 140 130 120 110 100 90 80 70 60 50 40 30 20 10 0
f1 (ppm)

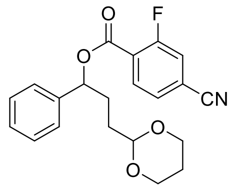
F
O
O
CN
O
O

S4

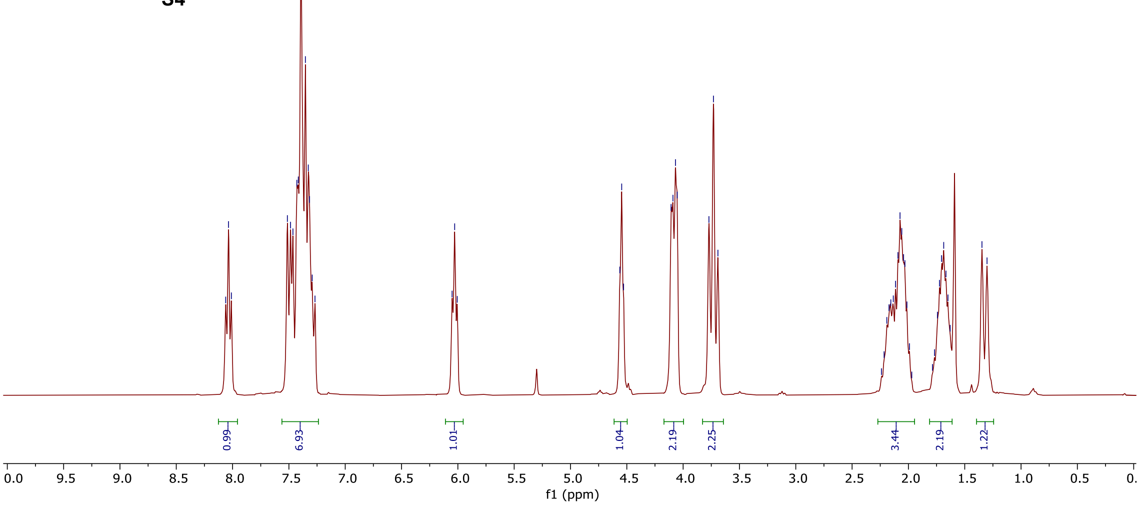
8.06
8.04
8.01
7.51
7.49
7.46
7.43
7.42
7.39
7.35
7.33
7.32
7.29
7.27
6.05
6.03
6.01
4.56
4.55
4.53
4.11
4.09
4.07
4.05
3.77
3.73
3.69
2.24
2.22
2.19
2.17
2.16
2.13
2.11
2.09
2.07
2.06
2.04
2.03
2.01
1.99
1.97
1.79
1.77
1.74
1.72
1.71
1.69
1.67
1.65
1.63
1.35
1.30
0.99
6.93
1.01
1.04
2.19
2.25
3.44
2.19
1.22
f1 (ppm)

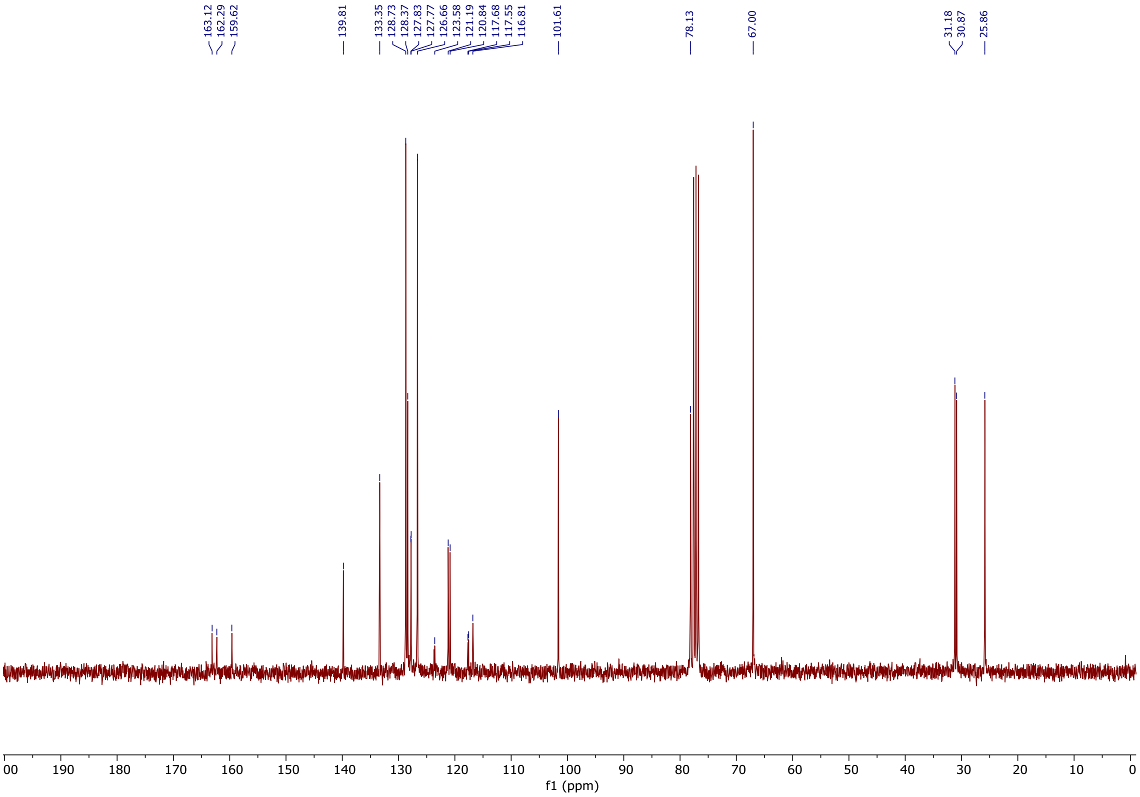
163.12
162.29
159.62
139.81
133.35
128.73
128.37
127.83
127.77
126.66
123.58
121.19
120.84
117.68
117.55
116.81
101.61
78.13
67.00
31.18
30.87
25.86
f1 (ppm)

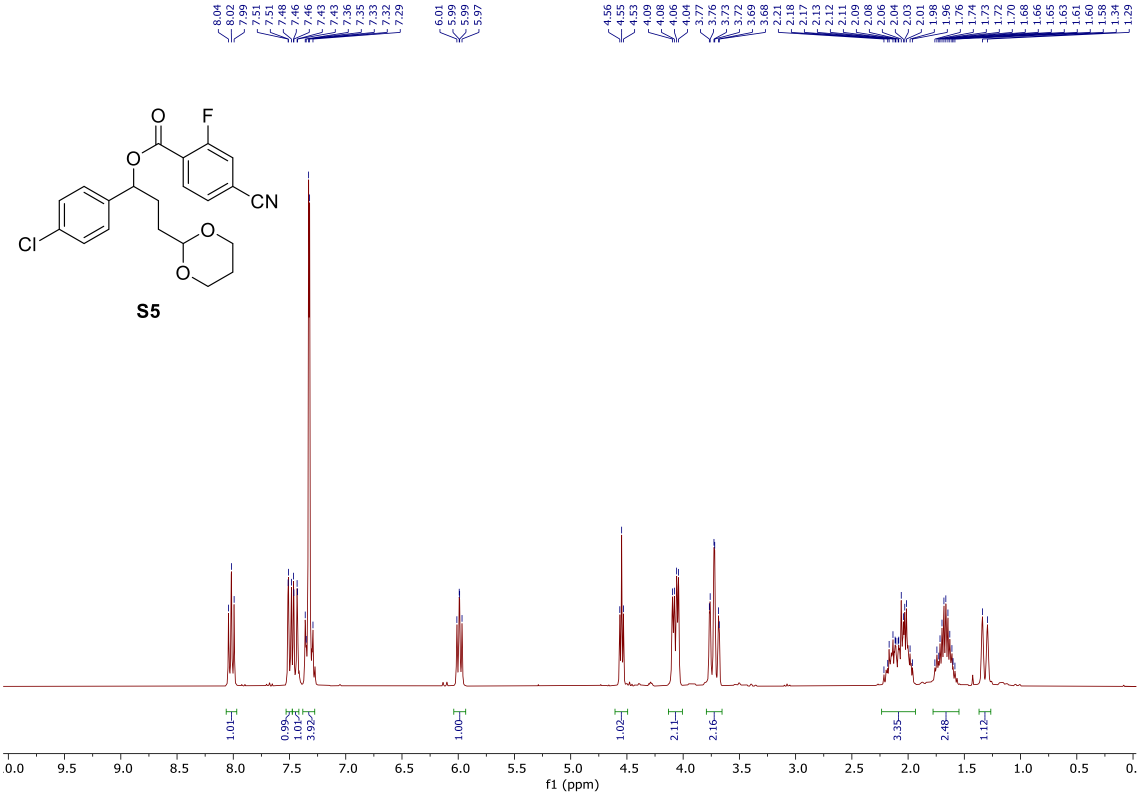

8.04 8.02 7.99 7.51 7.51 7.48 7.46 7.43 7.43 7.36 7.35 7.33 7.32 7.29
6.01 5.99 5.99 5.97
4.56 4.55 4.53 4.09 4.08 4.06 4.04 3.77 3.76 3.73 3.72 3.69 3.68 2.21 2.18 2.17 2.13 2.12 2.11 2.09 2.08 2.06 2.04 2.03 2.01 1.98 1.96 1.76 1.74 1.73 1.72 1.70 1.68 1.66 1.65 1.63 1.61 1.60 1.58 1.34 1.29
O
F
O
CN
Cl
O
O
S5
1.01 0.99 1.01 3.92 1.00 1.02 2.11 2.16 3.35 2.48 1.12
f1 (ppm)

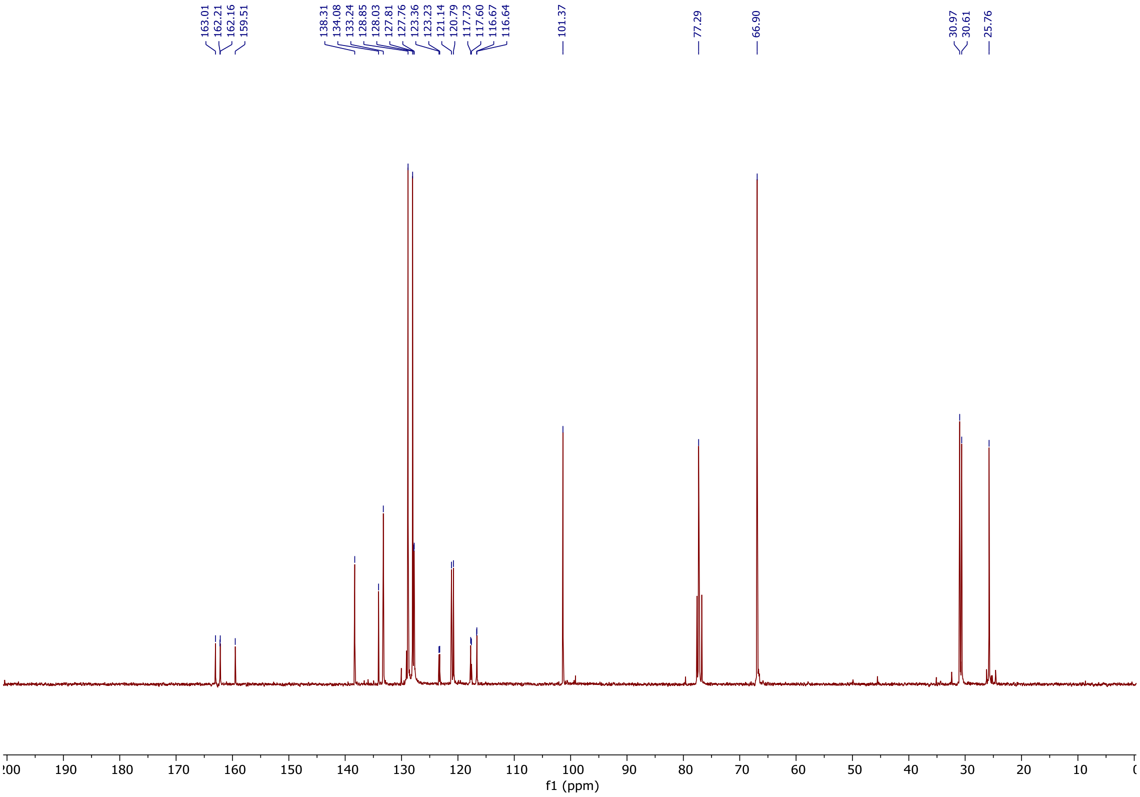

163.01 162.21 162.16 159.51
138.31 134.08 133.24 128.85 128.03 127.81 127.76 123.36 123.23 121.14 120.79 117.73 117.60 116.67 116.64
101.37
77.29
66.90
30.97 30.61 25.76
f1 (ppm)

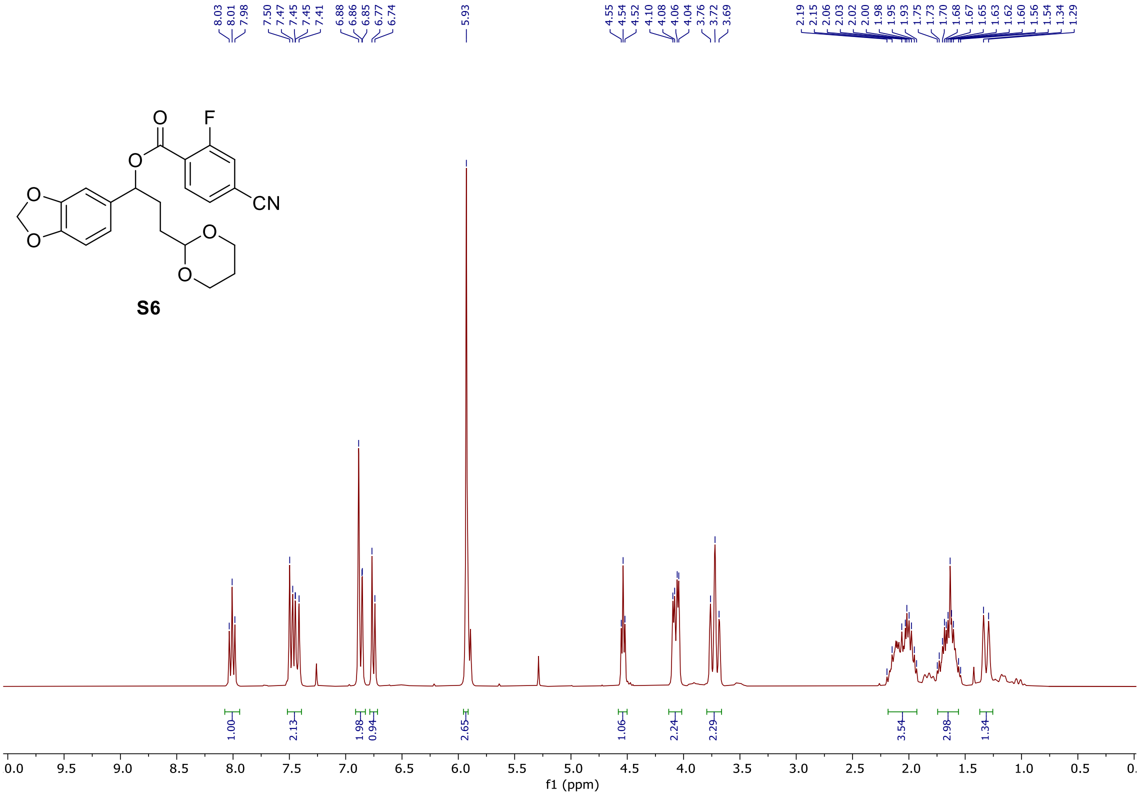

8.03
8.01
7.98
7.50
7.47
7.45
7.45
7.41
6.88
6.86
6.85
6.77
6.74
5.93
4.55
4.54
4.52
4.10
4.08
4.06
4.04
3.76
3.72
3.69
2.19
2.15
2.06
2.03
2.02
2.00
1.98
1.95
1.93
1.75
1.73
1.70
1.68
1.67
1.65
1.63
1.62
1.60
1.56
1.54
1.34
1.29
O
F
O
CN
O
O
O
O
S6
1.00
2.13
1.98
0.94
2.65
1.06
2.24
2.29
3.54
2.98
1.34
f1 (ppm)

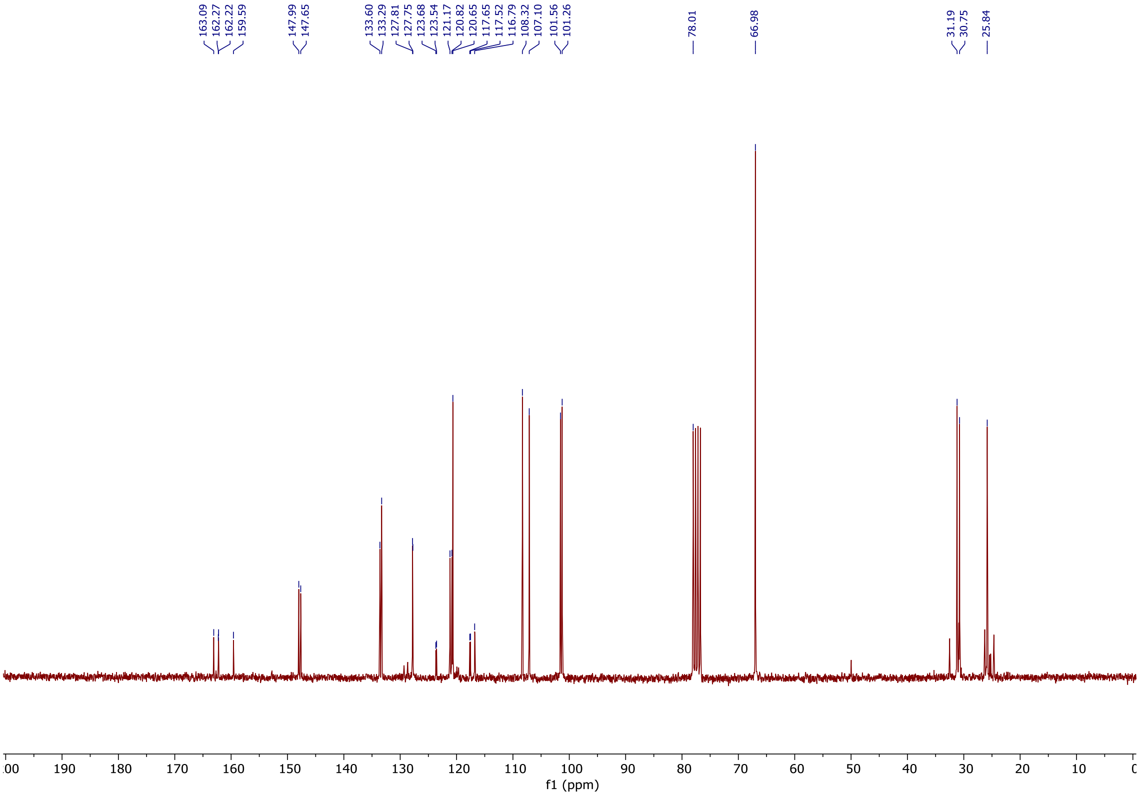

163.09
162.27
162.22
159.59
147.99
147.65
133.60
133.29
127.81
127.75
123.68
123.54
121.17
120.82
120.65
117.65
117.52
116.79
108.32
107.10
101.56
101.26
78.01
66.98
31.19
30.75
25.84
f1 (ppm)

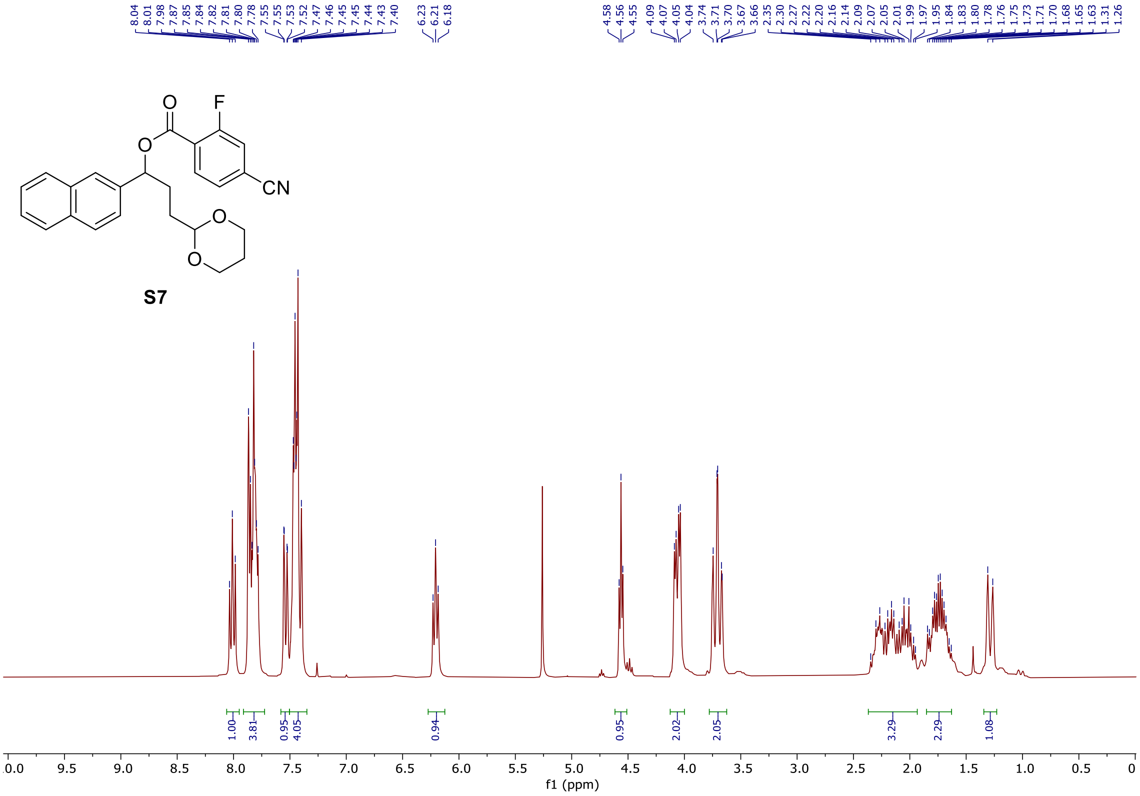
8.04 8.01 7.98 7.87 7.85 7.84 7.82 7.81 7.80 7.78 7.55 7.55 7.53 7.52 7.47 7.46 7.45 7.45 7.44 7.43 7.40
6.23 6.21 6.18
4.58 4.56 4.55 4.09 4.07 4.05 4.04 3.74 3.71 3.70 3.67 3.66 2.35 2.30 2.27 2.22 2.20 2.16 2.14 2.09 2.07 2.05 2.01 1.99 1.97 1.95 1.84 1.83 1.80 1.78 1.76 1.75 1.73 1.71 1.70 1.68 1.65 1.63 1.31 1.26
O
F
O
CN
O
O
S7
1.00 3.81 0.95 4.05 0.94 0.95 2.02 2.05 3.29 2.29 1.08
10.0 9.5 9.0 8.5 8.0 7.5 7.0 6.5 6.0 5.5 5.0 4.5 4.0 3.5 3.0 2.5 2.0 1.5 1.0 0.5 0
f1 (ppm)

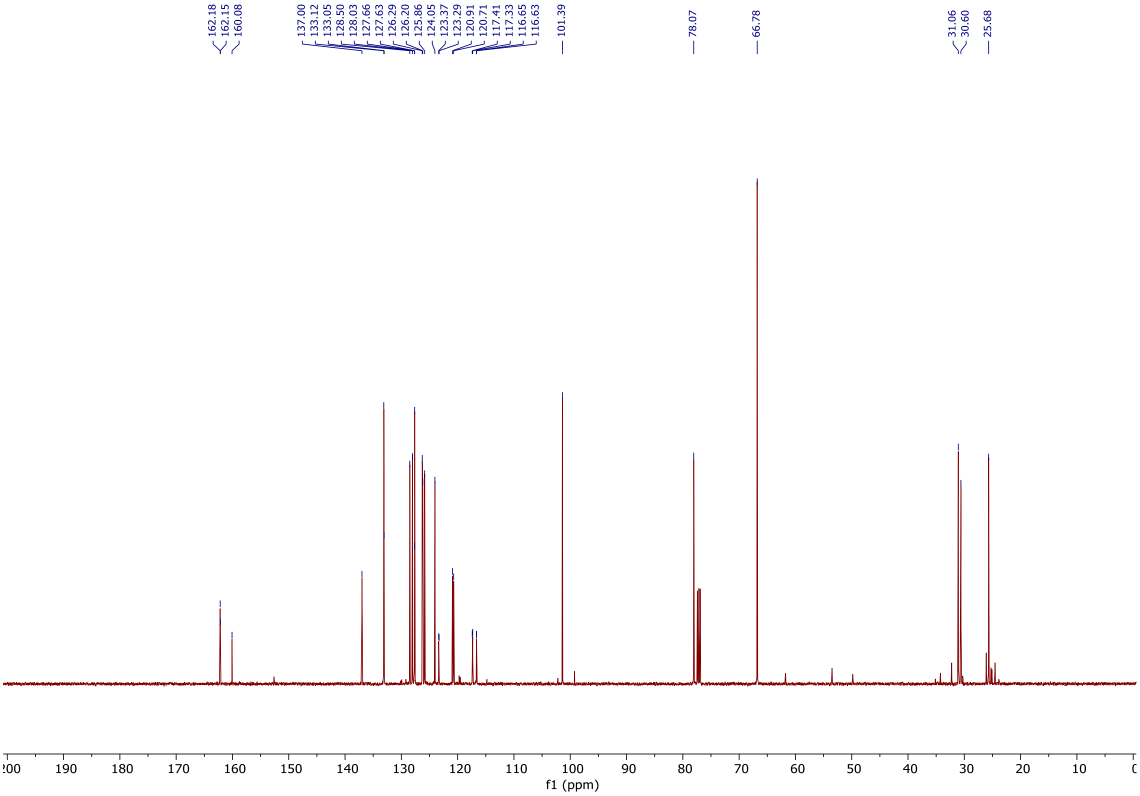
162.18 162.15 160.08
137.00 133.12 133.05 128.50 128.03 127.66 127.63 126.29 126.20 125.86 124.05 123.37 123.29 120.91 120.71 117.41 117.33 116.65 116.63
101.39
78.07
66.78
31.06 30.60 25.68
200 190 180 170 160 150 140 130 120 110 100 90 80 70 60 50 40 30 20 10 0
f1 (ppm)

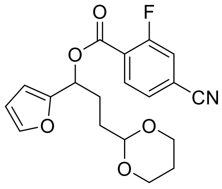
O
F
O
CN
O
O
O

S8

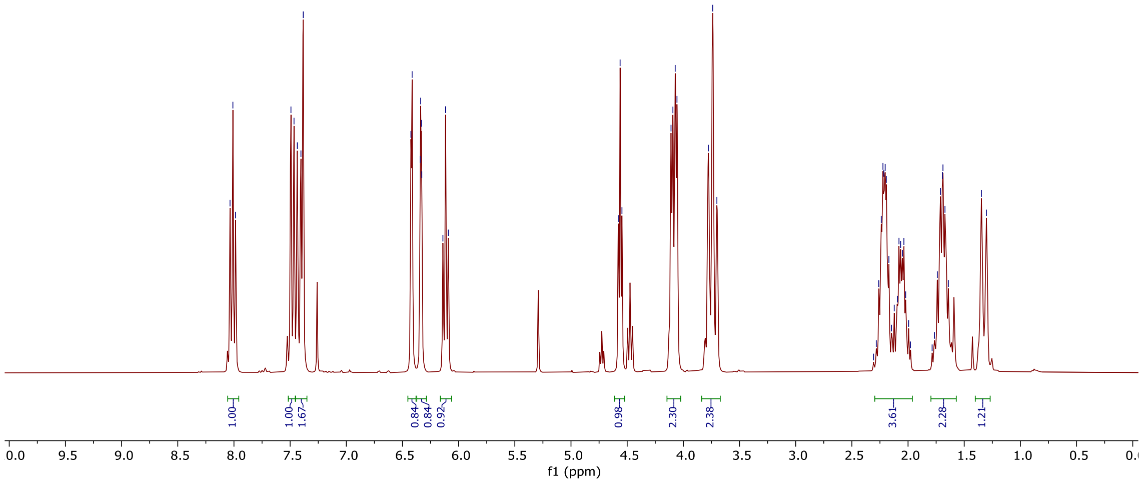
8.04
8.01
7.99
7.49
7.47
7.44
7.40
7.38
6.42
6.41
6.34
6.34
6.33
6.33
6.14
6.12
6.09
4.58
4.56
4.55
4.11
4.09
4.07
4.06
3.78
3.74
3.70
2.31
2.28
2.26
2.24
2.22
2.22
2.20
2.19
2.17
2.15
2.12
2.09
2.08
2.07
2.05
2.04
2.02
1.99
1.98
1.79
1.76
1.74
1.71
1.69
1.69
1.67
1.64
1.35
1.30
1.00
1.00
1.67
0.84
0.84
0.92
0.98
2.30
2.38
3.61
2.28
1.21
0.0 9.5 9.0 8.5 8.0 7.5 7.0 6.5 6.0 5.5 5.0 4.5 4.0 3.5 3.0 2.5 2.0 1.5 1.0 0.5
f1 (ppm)

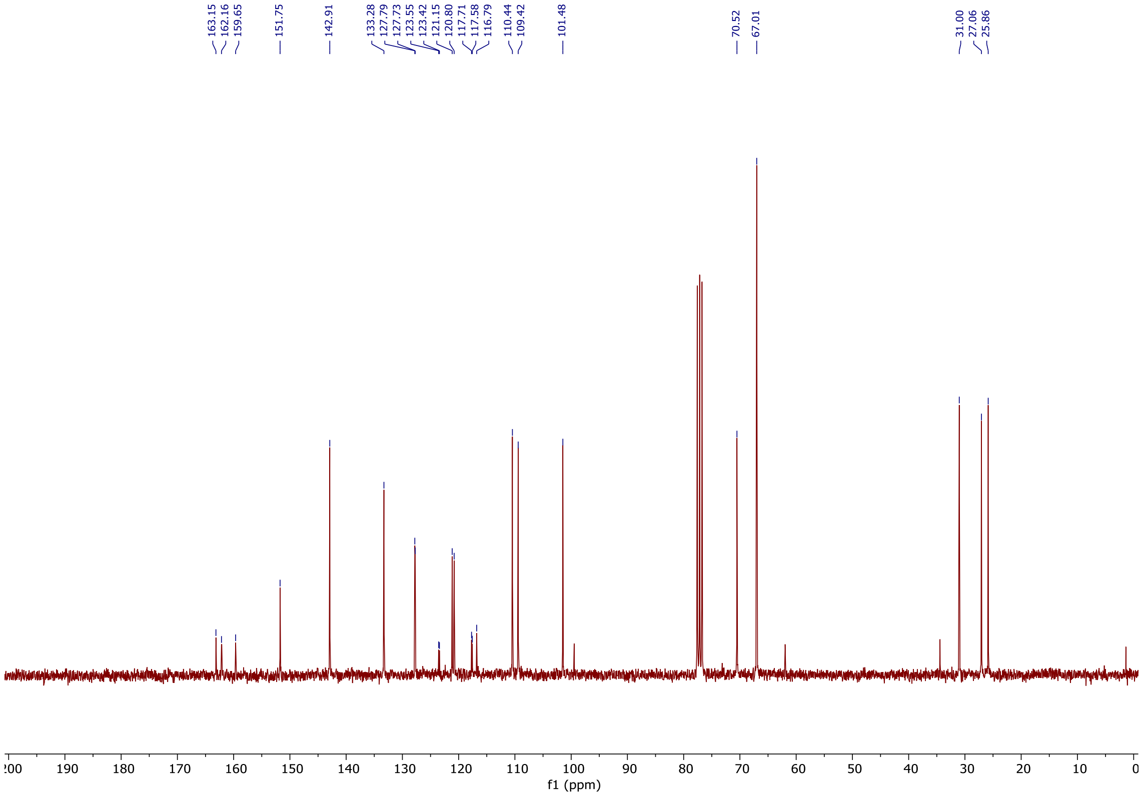
163.15
162.16
159.65
151.75
142.91
133.28
127.79
127.73
123.55
123.42
121.15
120.80
117.71
117.58
116.79
110.44
109.42
101.48
70.52
67.01
31.00
27.06
25.86
190 180 170 160 150 140 130 120 110 100 90 80 70 60 50 40 30 20 10 0
f1 (ppm)

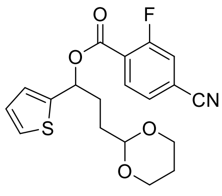
O
F
O
CN
S
O
O

S9

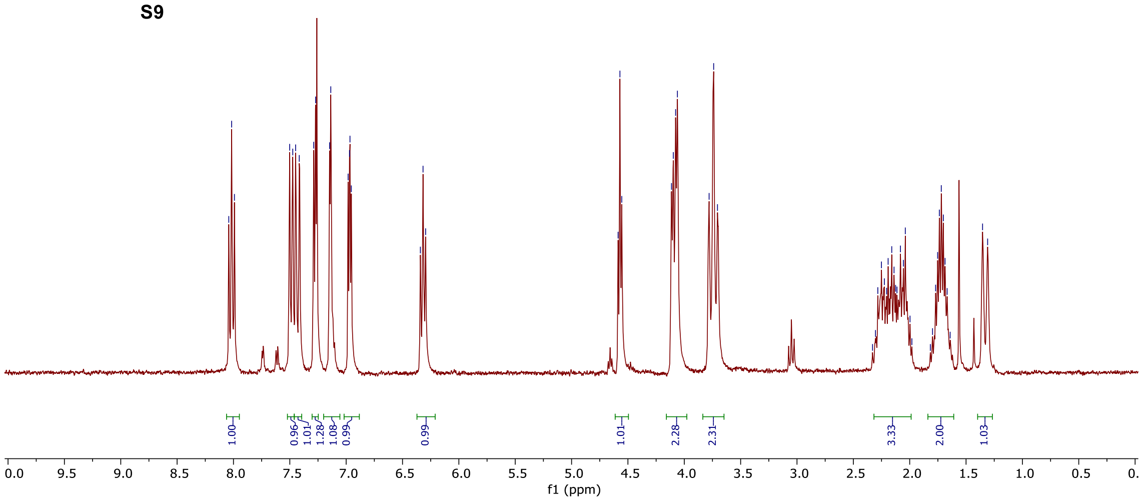
8.04
8.02
7.99
7.50
7.47
7.45
7.42
7.29
7.27
7.15
7.14
6.98
6.97
6.97
6.95
6.34
6.32
6.29
4.59
4.57
4.55
4.11
4.10
4.08
4.06
3.78
3.74
3.71
2.33
2.30
2.28
2.25
2.22
2.20
2.19
2.16
2.14
2.12
2.11
2.08
2.06
2.04
2.00
1.98
1.82
1.80
1.77
1.75
1.74
1.72
1.70
1.69
1.67
1.64
1.35
1.31
1.00
0.96
1.01
1.28
1.08
0.99
0.99
1.01
2.28
2.31
3.33
2.00
1.03
f1 (ppm)

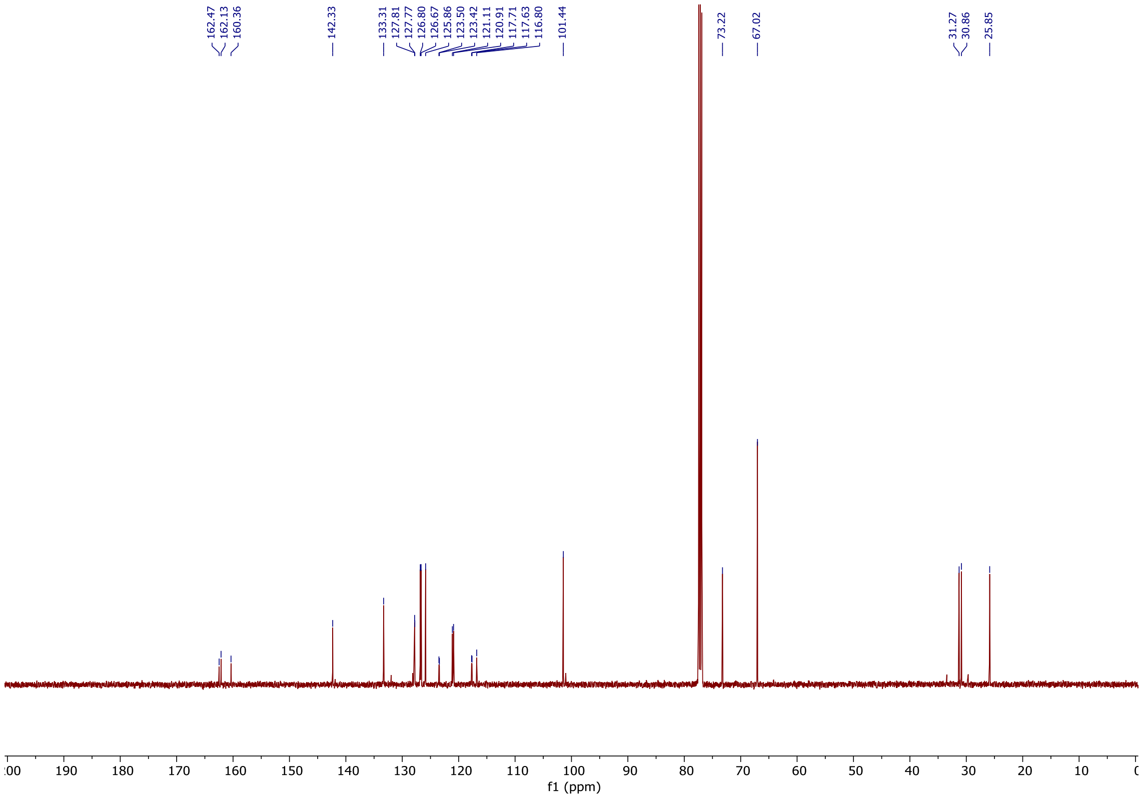
162.47
162.13
160.36
142.33
133.31
127.81
127.77
126.80
126.67
125.86
123.50
123.42
121.11
120.91
117.71
117.63
116.80
101.44
73.22
67.02
31.27
30.86
25.85
f1 (ppm)

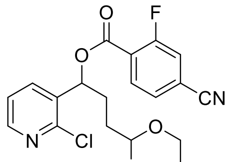
O
F
O
CN
N
Cl
O
O

S10

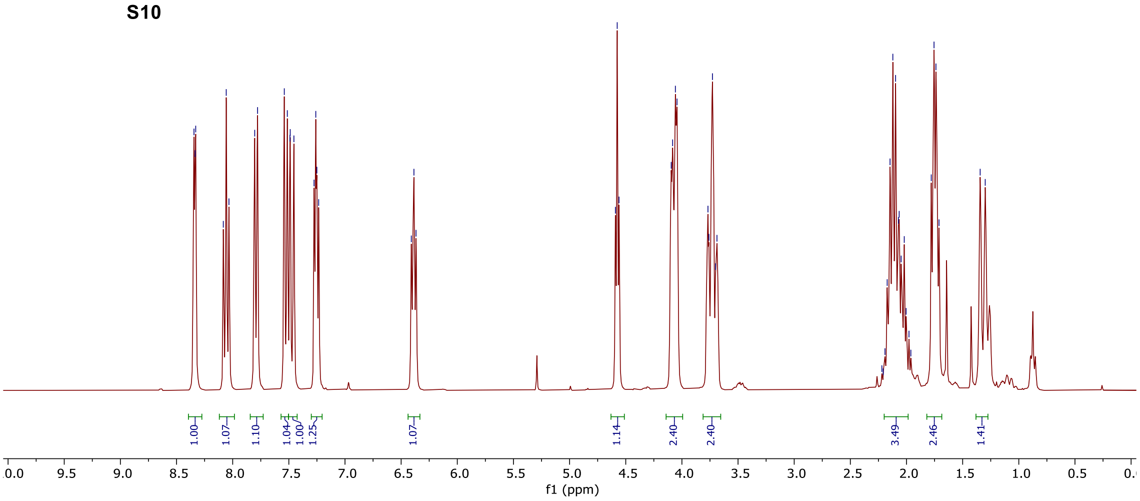
8.34
8.33
8.33
8.08
8.06
8.03
7.80
7.78
7.54
7.51
7.49
7.49
7.45
7.28
7.26
7.25
7.23
6.41
6.39
6.37
4.59
4.58
4.56
4.10
4.08
4.06
4.04
3.77
3.76
3.73
3.70
3.69
2.22
2.21
2.19
2.17
2.15
2.12
2.10
2.07
2.07
2.05
2.02
2.00
1.98
1.96
1.78
1.75
1.74
1.71
1.34
1.30
1.00
1.07
1.10
1.04
1.00
1.25
1.07
1.14
2.40
2.40
3.49
2.46
1.41
f1 (ppm)

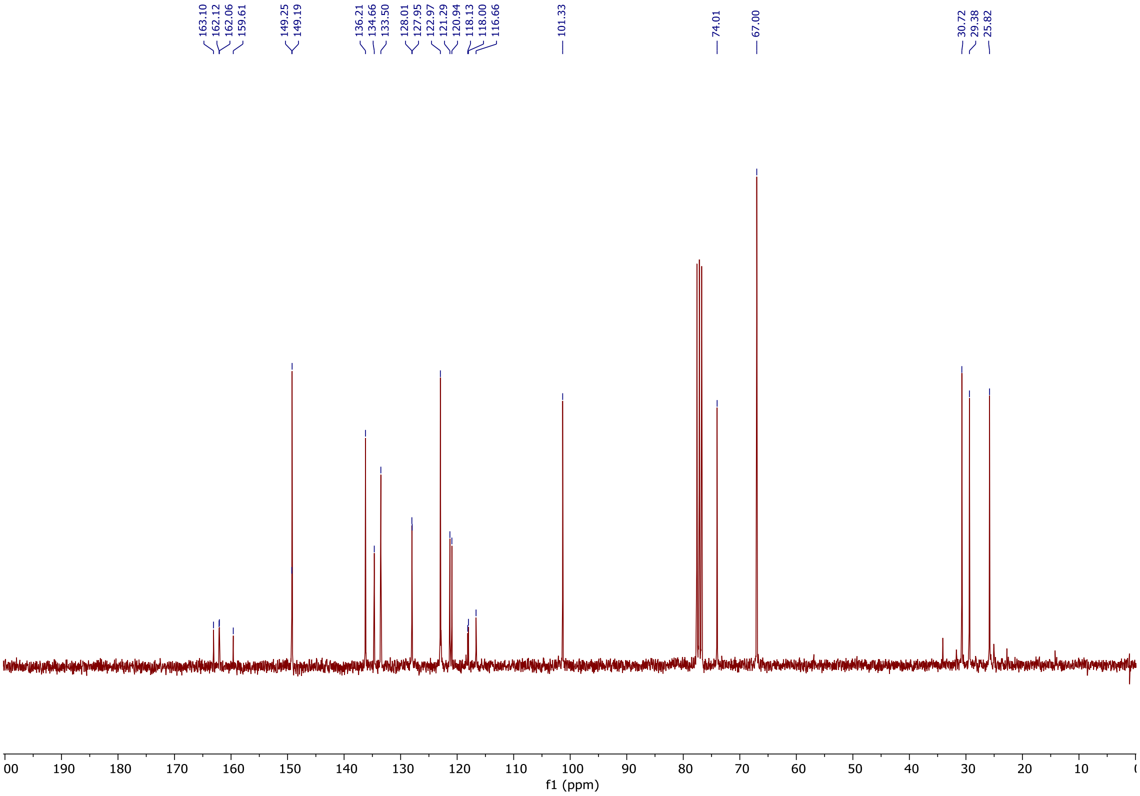
163.10
162.12
162.06
159.61
149.25
149.19
136.21
134.66
133.50
128.01
127.95
122.97
121.29
120.94
118.13
118.00
116.66
101.33
74.01
67.00
30.72
29.38
25.82
f1 (ppm)

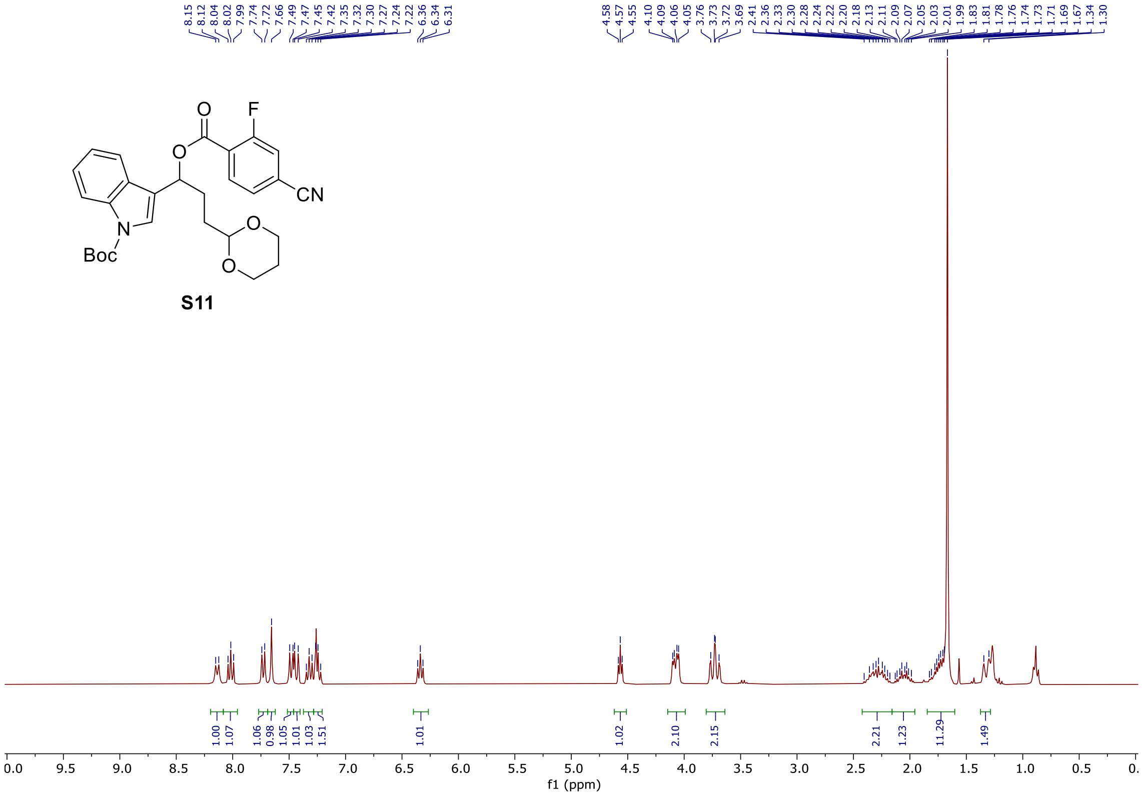

S11

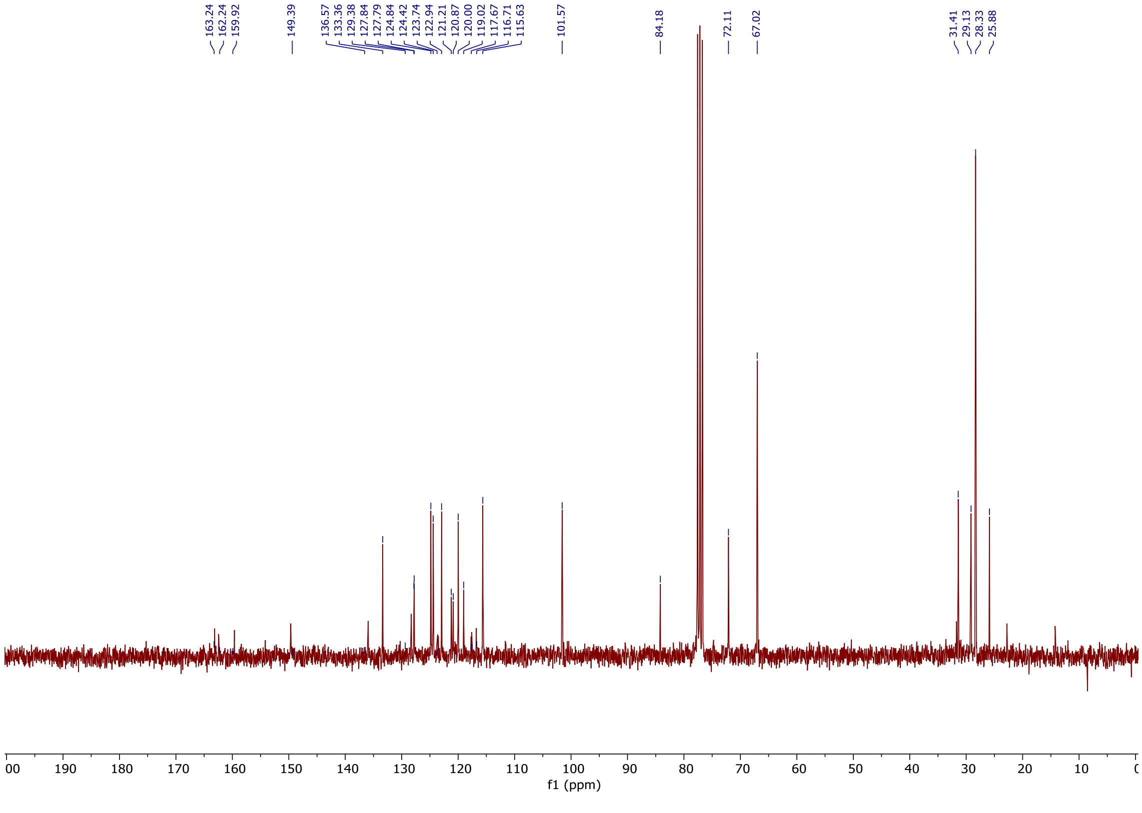

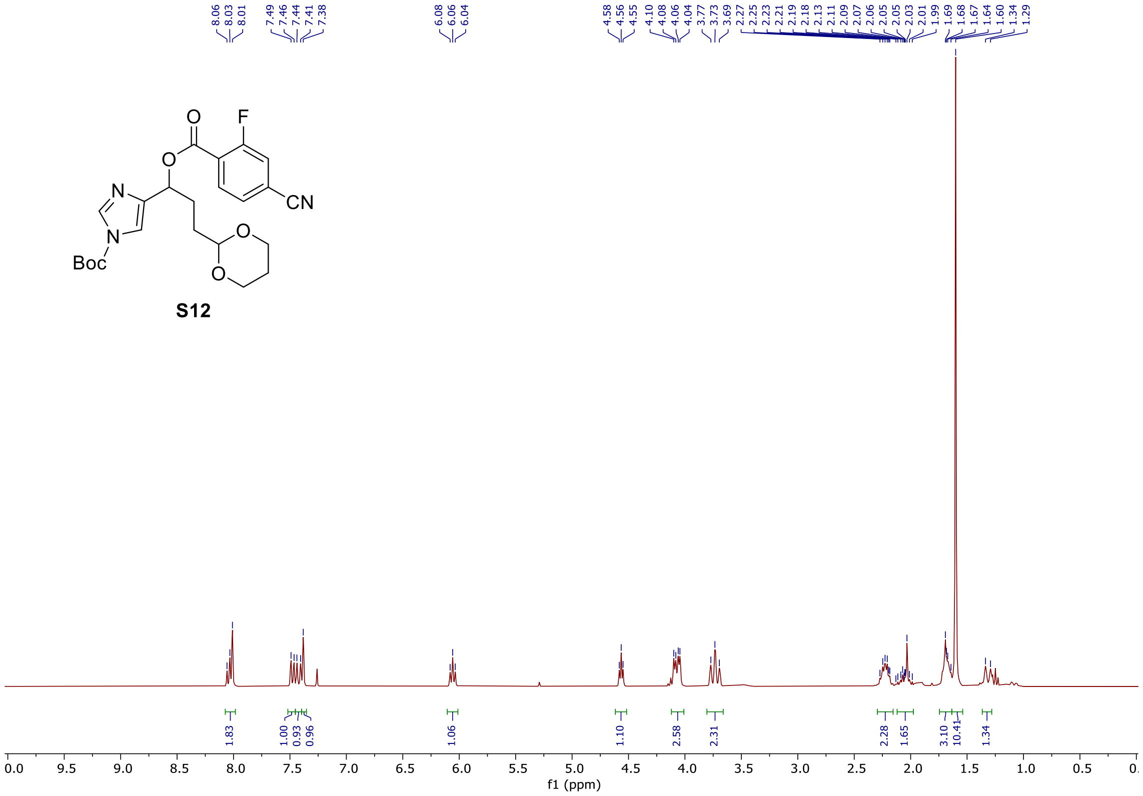
O
F
O
N
CN
N
O
Boc
O

S12

8.06
8.03
8.01
7.49
7.46
7.44
7.41
7.38
6.08
6.06
6.04
4.58
4.56
4.55
4.10
4.08
4.06
4.04
3.77
3.73
3.69
2.27
2.25
2.23
2.21
2.19
2.18
2.13
2.11
2.09
2.07
2.06
2.05
2.05
2.03
2.01
1.99
1.69
1.68
1.67
1.64
1.60
1.34
1.29
1.83
1.00
0.93
0.96
1.06
1.10
2.58
2.31
2.28
1.65
3.10
10.41
1.34
f1 (ppm)

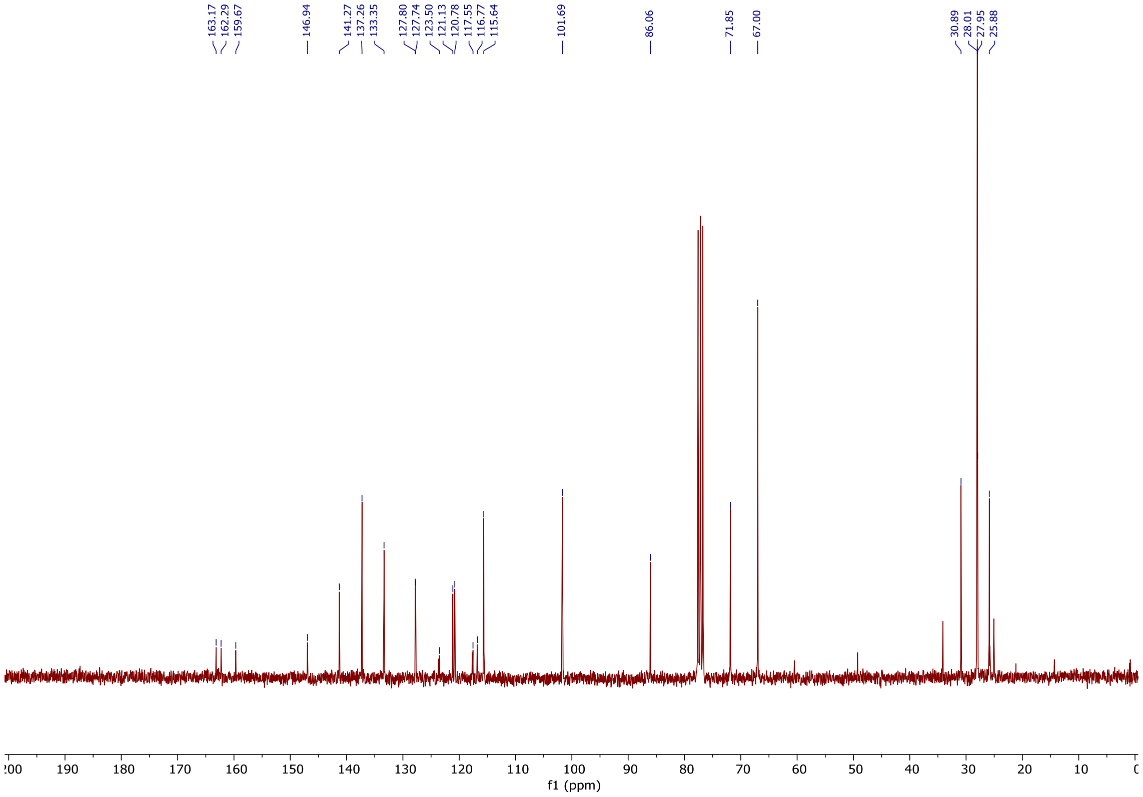
163.17
162.29
159.67
146.94
141.27
137.26
133.35
127.80
127.74
123.50
121.13
120.78
117.55
116.77
115.64
101.69
86.06
71.85
67.00
30.89
28.01
27.95
25.88
f1 (ppm)

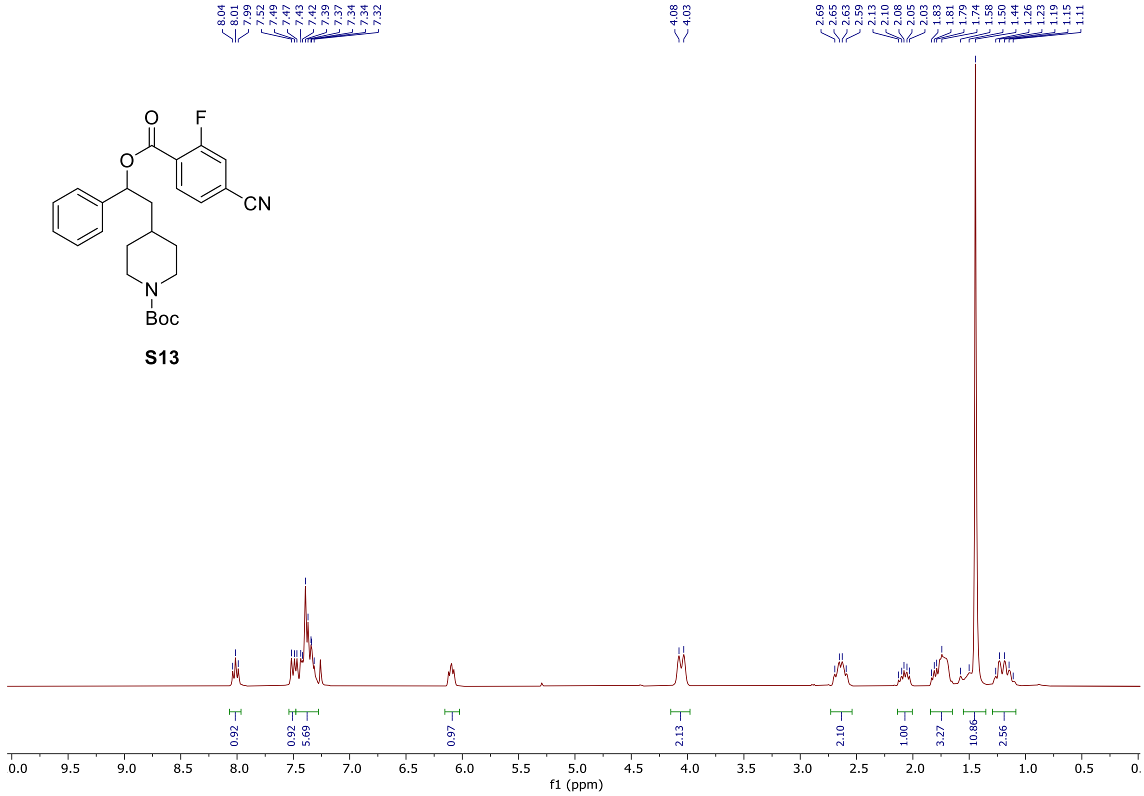
8.04
8.01
7.99
7.52
7.49
7.47
7.43
7.42
7.39
7.37
7.34
7.34
7.32
4.08
4.03
2.69
2.65
2.63
2.59
2.13
2.10
2.08
2.05
2.03
1.83
1.81
1.79
1.74
1.58
1.50
1.44
1.26
1.23
1.19
1.15
1.11
O
F
O
CN
N
Boc
S13
0.92
0.92
5.69
0.97
2.13
2.10
1.00
3.27
10.86
2.56
f1 (ppm)

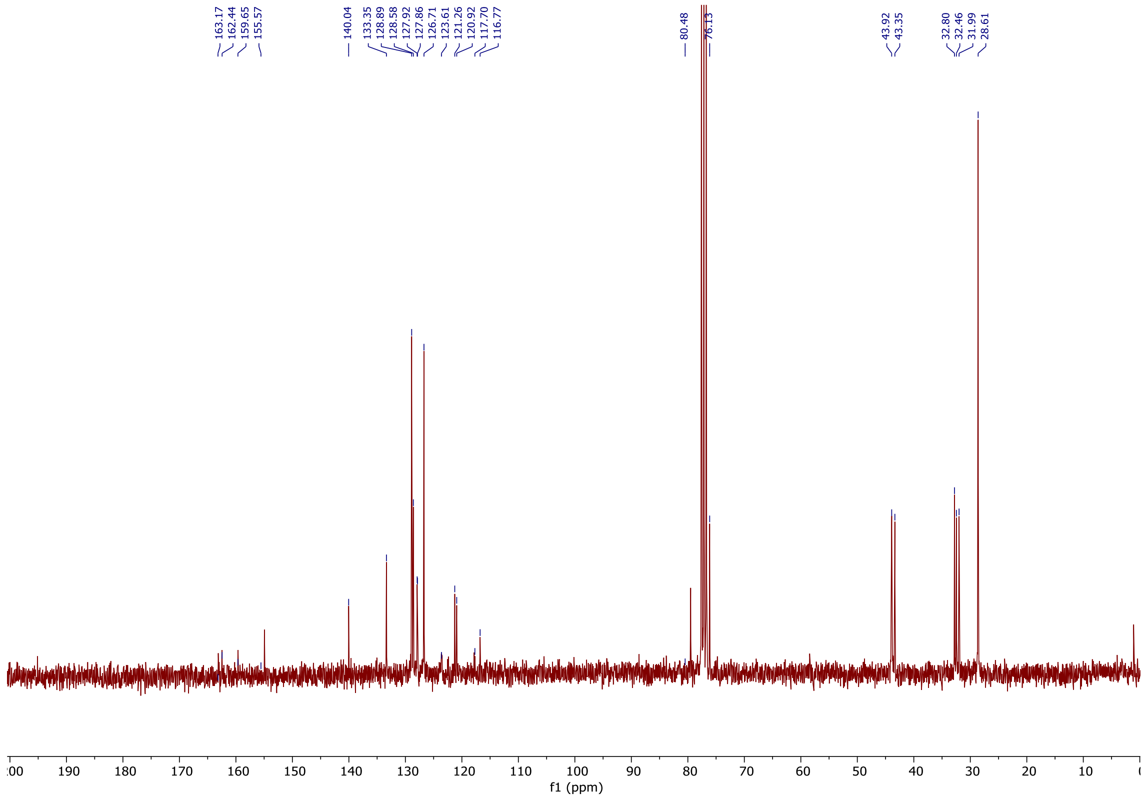
163.17
162.44
159.65
155.57
140.04
133.35
128.89
128.58
127.92
127.86
126.71
123.61
121.26
120.92
117.70
116.77
80.48
76.13
43.92
43.35
32.80
32.46
31.99
28.61
f1 (ppm)

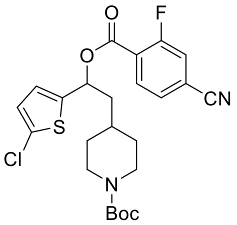

S14

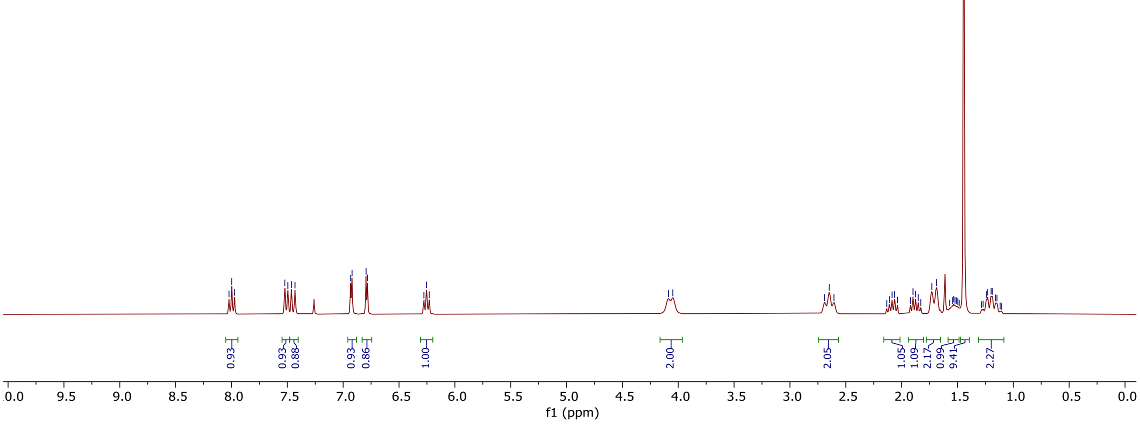

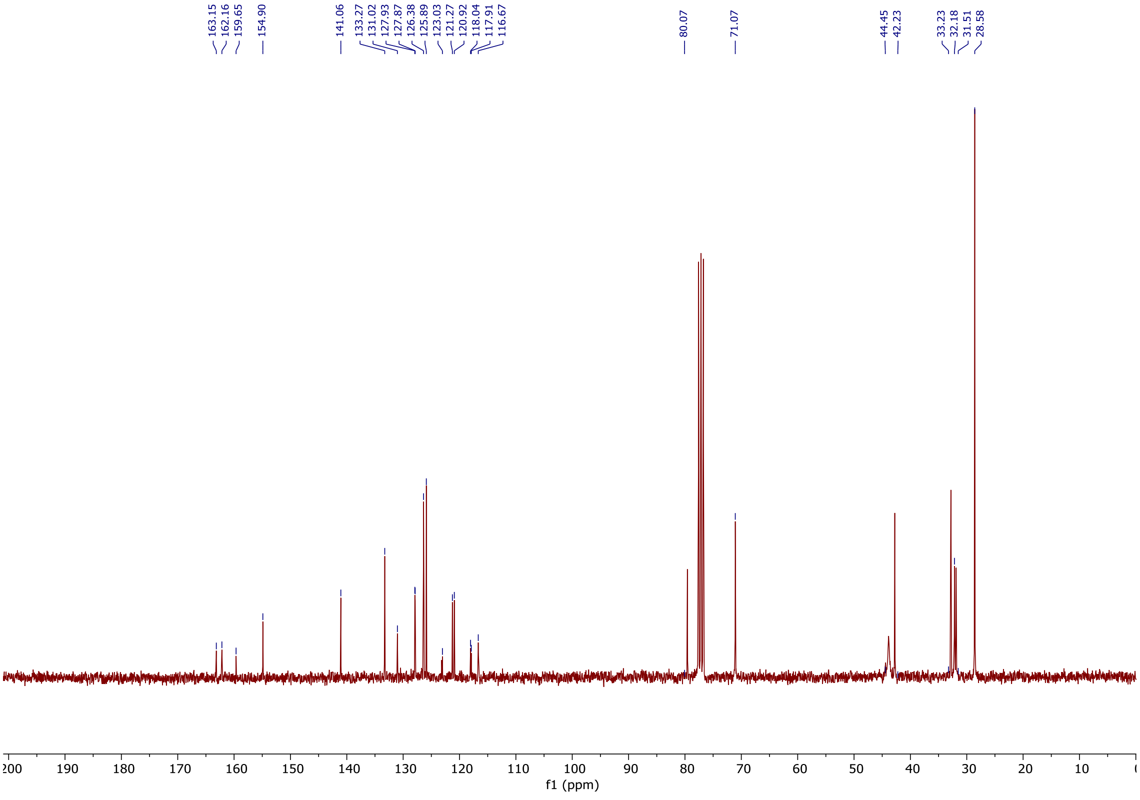

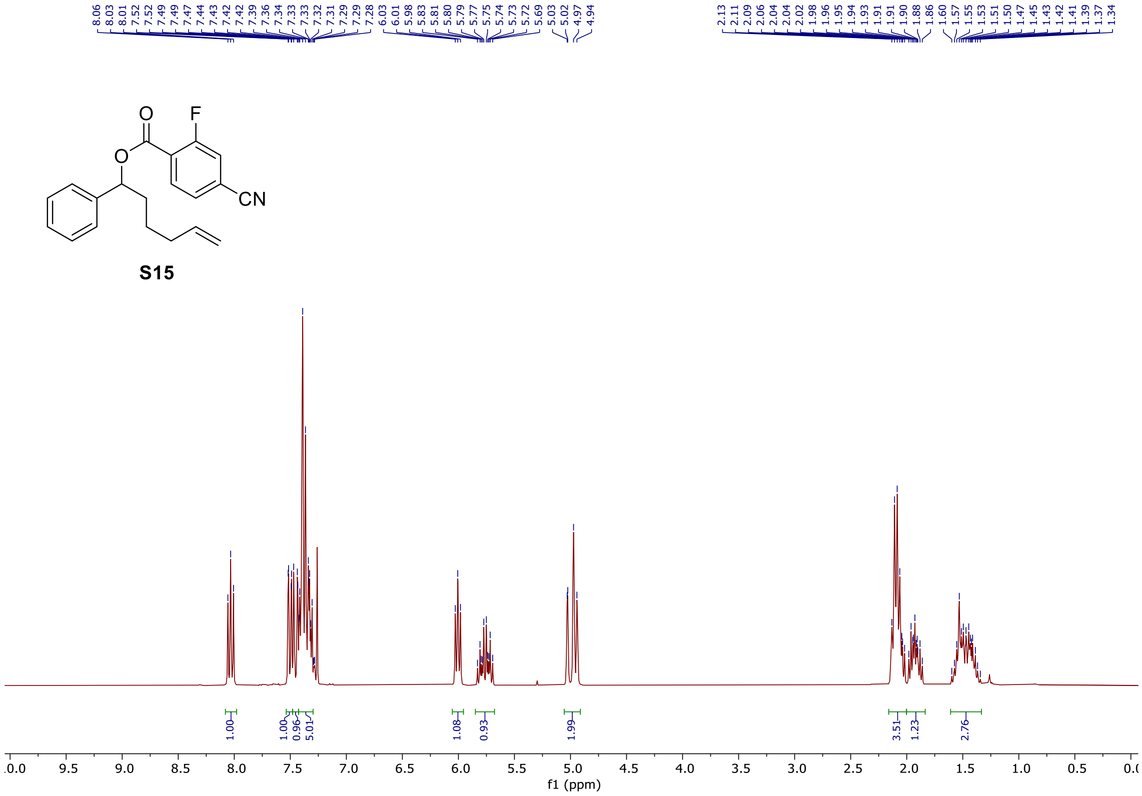
8.06 8.03 8.01 7.52 7.52 7.49 7.49 7.47 7.44 7.43 7.42 7.42 7.39 7.36 7.34 7.33 7.33 7.32 7.31 7.29 7.29 7.28 6.03 6.01 5.98 5.83 5.81 5.80 5.79 5.77 5.75 5.74 5.73 5.72 5.69 5.03 5.02 4.97 4.94
2.13 2.11 2.09 2.06 2.04 2.04 2.02 1.98 1.96 1.95 1.94 1.93 1.91 1.91 1.90 1.88 1.86 1.60 1.57 1.55 1.53 1.51 1.50 1.47 1.45 1.43 1.42 1.41 1.39 1.37 1.34
O
F
O
CN
S15
1.00
1.00
0.96
5.01
1.08
0.93
1.99
3.51
1.23
2.76
f1 (ppm)

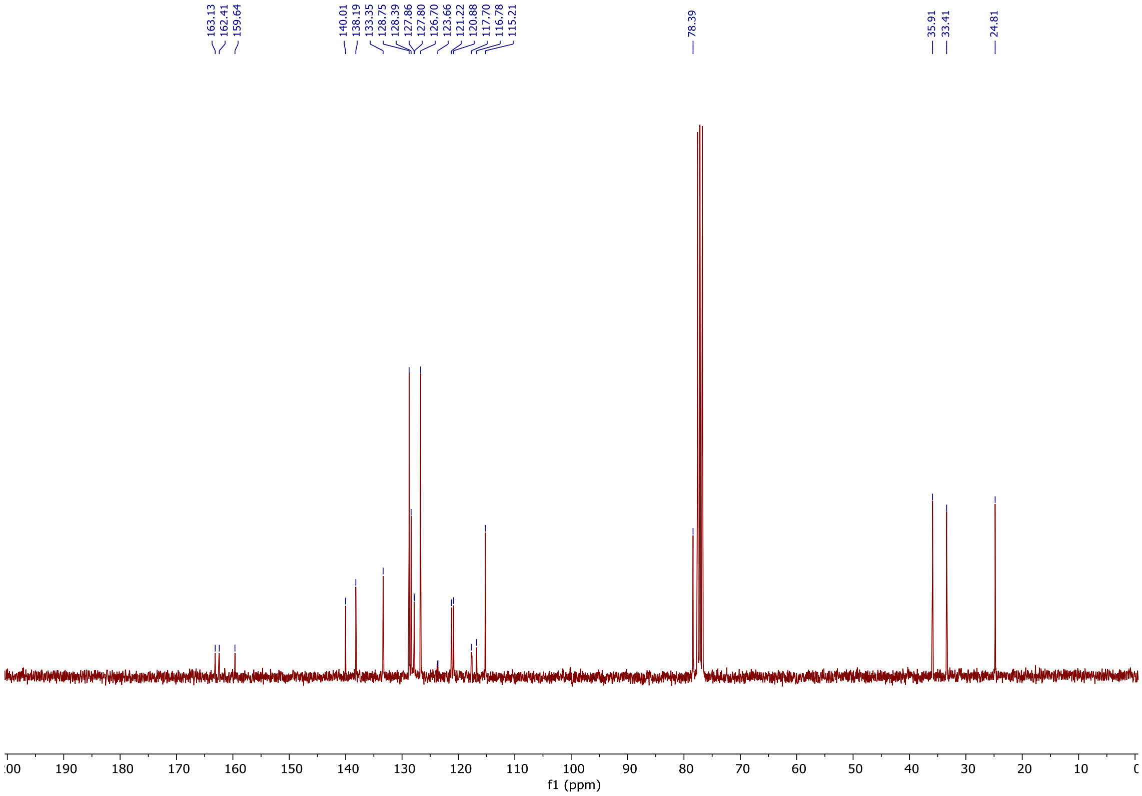
163.13 162.41 159.64
140.01 138.19 133.35 128.75 128.39 127.86 127.80 126.70 123.66 121.22 120.88 117.70 116.78 115.21
78.39
35.91 33.41
24.81
f1 (ppm)

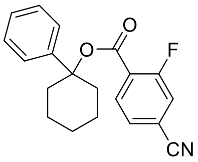
O
F
CN

S16

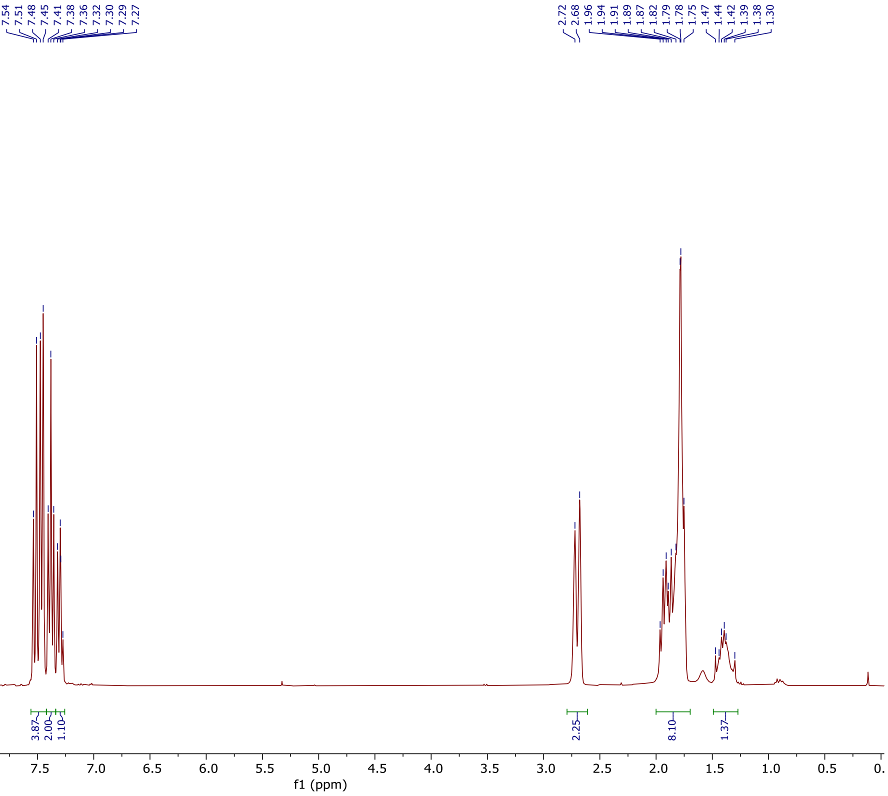
8.03
8.01
7.98
7.54
7.51
7.48
7.45
7.41
7.38
7.36
7.32
7.30
7.29
7.27
2.72
2.68
1.96
1.94
1.91
1.89
1.87
1.82
1.79
1.78
1.75
1.47
1.44
1.42
1.39
1.38
1.30
0.94
3.87
2.00
1.10
2.25
8.10
1.37
f1 (ppm)

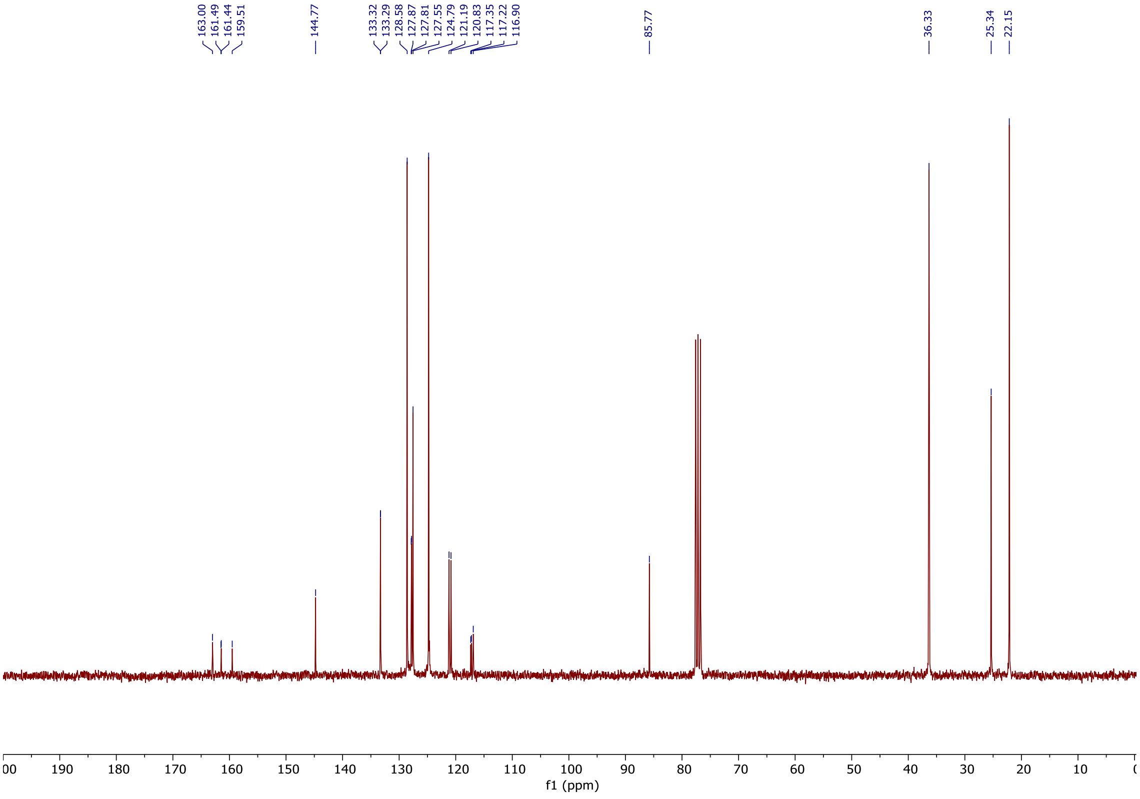
163.00
161.49
161.44
159.51
144.77
133.32
133.29
128.58
127.87
127.81
127.55
124.79
121.19
120.83
117.35
117.22
116.90
85.77
36.33
25.34
22.15
f1 (ppm)

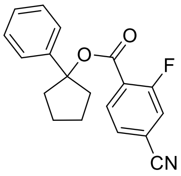
O
F
CN

S17

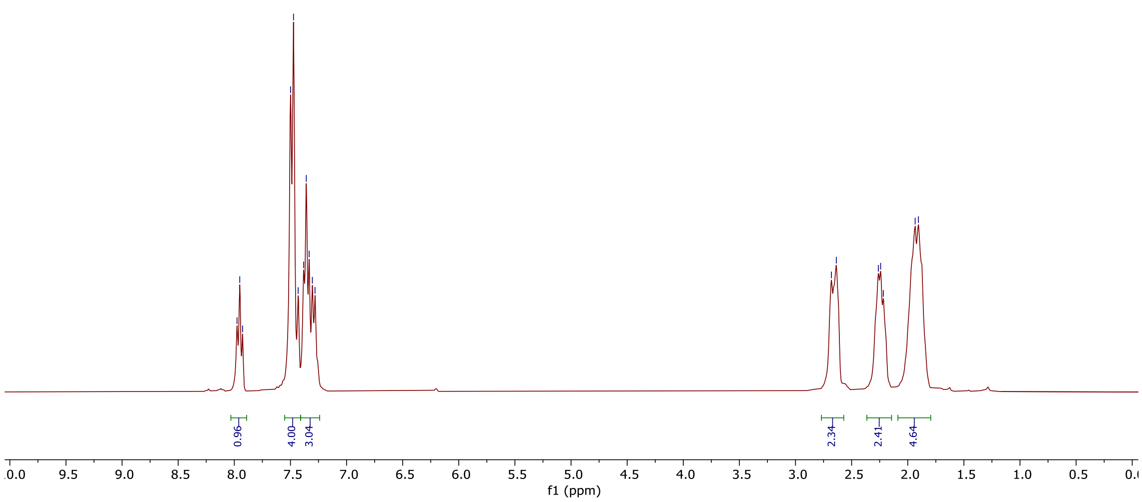
7.98
7.95
7.93
7.50
7.47
7.43
7.38
7.36
7.33
7.30
7.28
2.68
2.64
2.26
2.24
2.22
1.93
1.91
0.96
4.00
3.04
2.34
2.41
4.64
f1 (ppm)

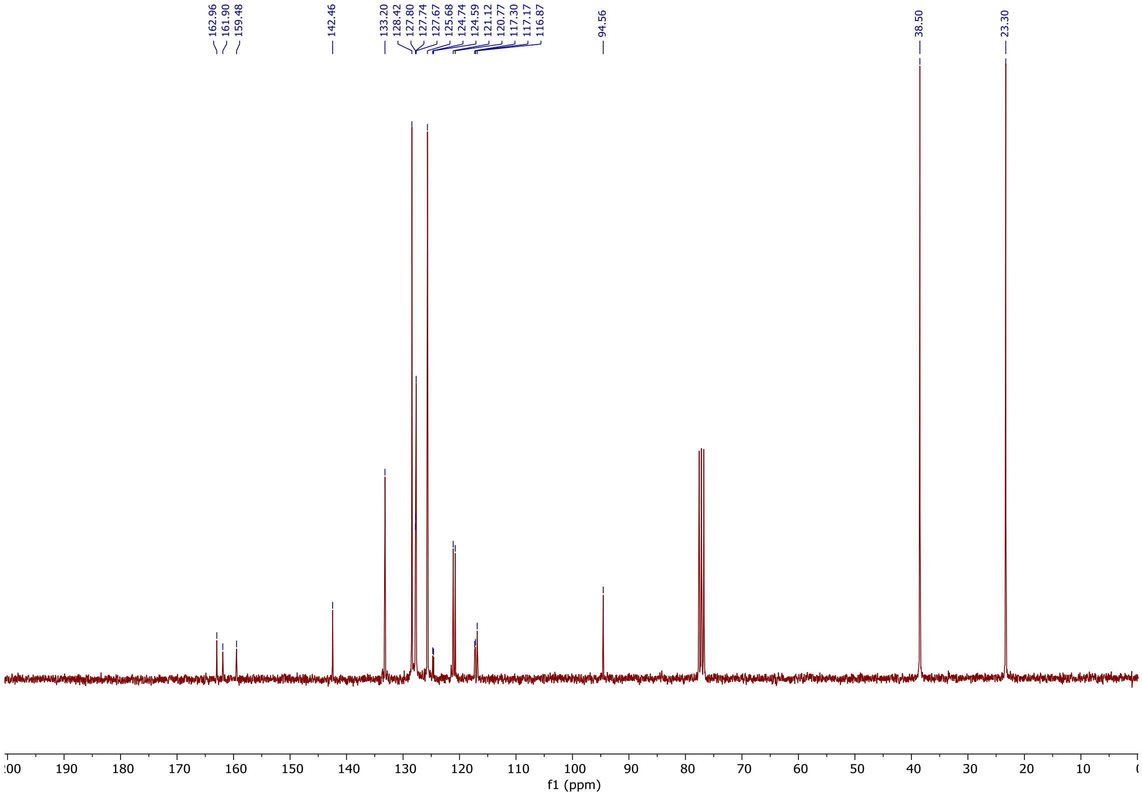
162.96
161.90
159.48
142.46
133.20
128.42
127.80
127.74
127.67
125.68
124.74
124.59
121.12
120.77
117.30
117.17
116.87
94.56
38.50
23.30
f1 (ppm)

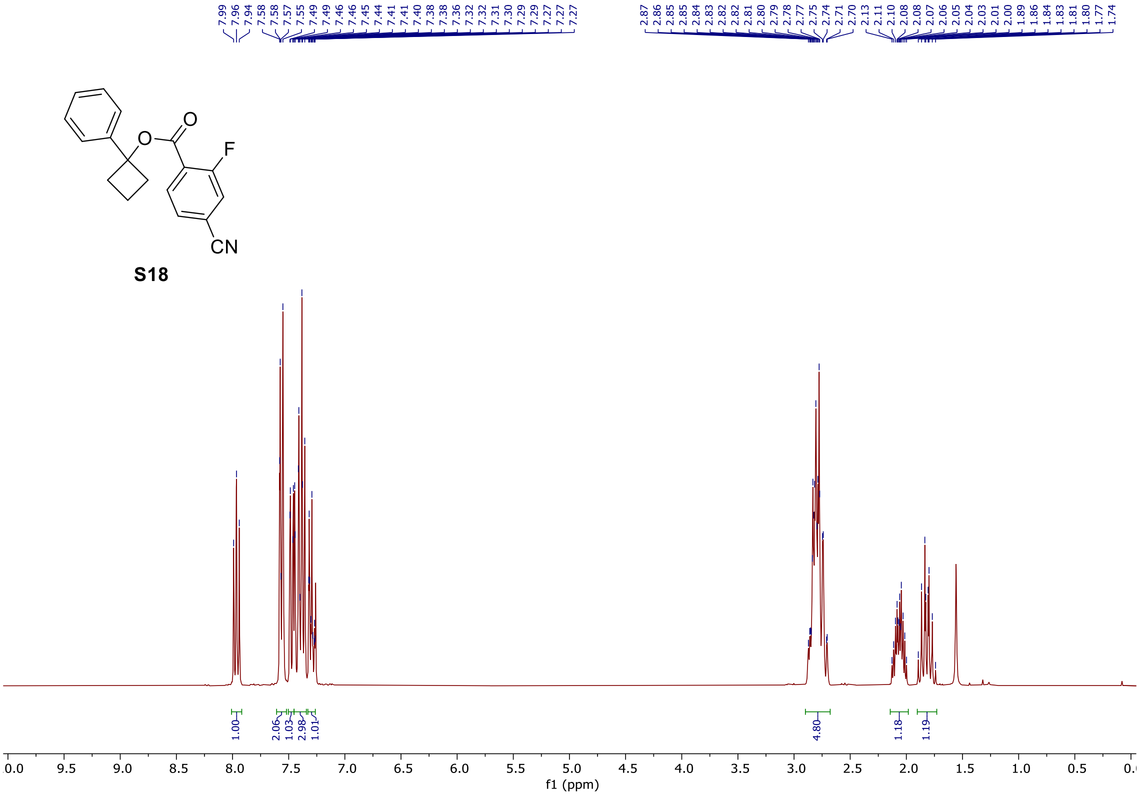

S18
7.99 7.96 7.94 7.58 7.58 7.57 7.55 7.49 7.49 7.46 7.45 7.44 7.41 7.41 7.40 7.38 7.38 7.36 7.32 7.32 7.31 7.30 7.29 7.29 7.27 7.27
2.87 2.86 2.85 2.84 2.83 2.82 2.81 2.80 2.79 2.78 2.77 2.75 2.74 2.71 2.70 2.13 2.11 2.10 2.08 2.08 2.07 2.06 2.05 2.04 2.03 2.01 2.00 1.89 1.86 1.84 1.83 1.81 1.80 1.77 1.74
1.00 2.06 1.03 2.98 1.01 4.80 1.18 1.19
f1 (ppm)

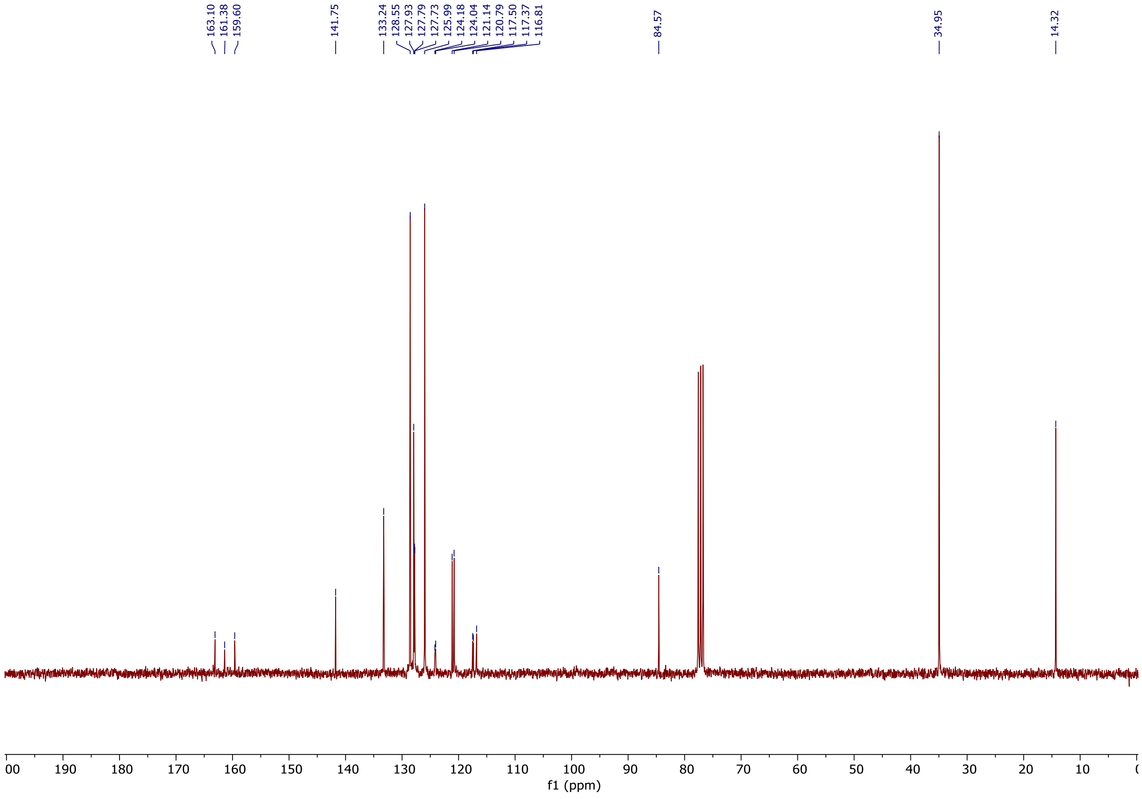
163.10 161.38 159.60
141.75
133.24 128.55 127.93 127.79 127.73 125.99 124.18 124.04 121.14 120.79 117.50 117.37 116.81
84.57
34.95
14.32
f1 (ppm)

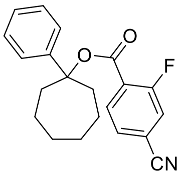

O
F
CN

S19

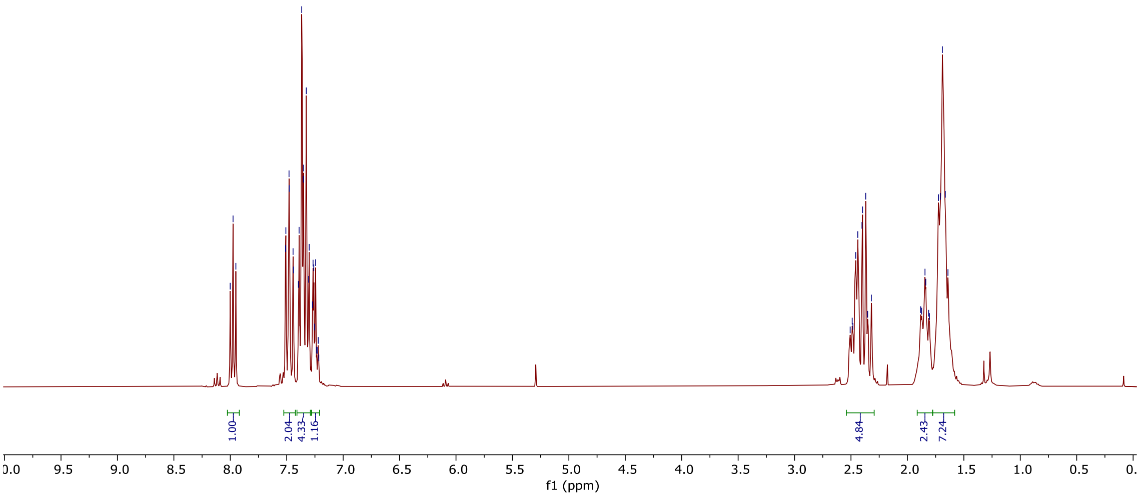

8.00 7.98 7.95 7.51 7.51 7.48 7.48 7.44 7.44 7.40 7.39 7.37 7.35 7.35 7.33 7.31 7.30 7.27 7.27 7.26 7.25 7.24 7.23 7.23 7.22 7.22
2.51 2.49 2.48 2.46 2.44 2.40 2.40 2.37 2.35 2.32 1.88 1.87 1.84 1.84 1.81 1.80 1.72 1.71 1.69 1.66 1.64
1.00 2.04 4.33 1.16 4.84 2.43 7.24
f1 (ppm)

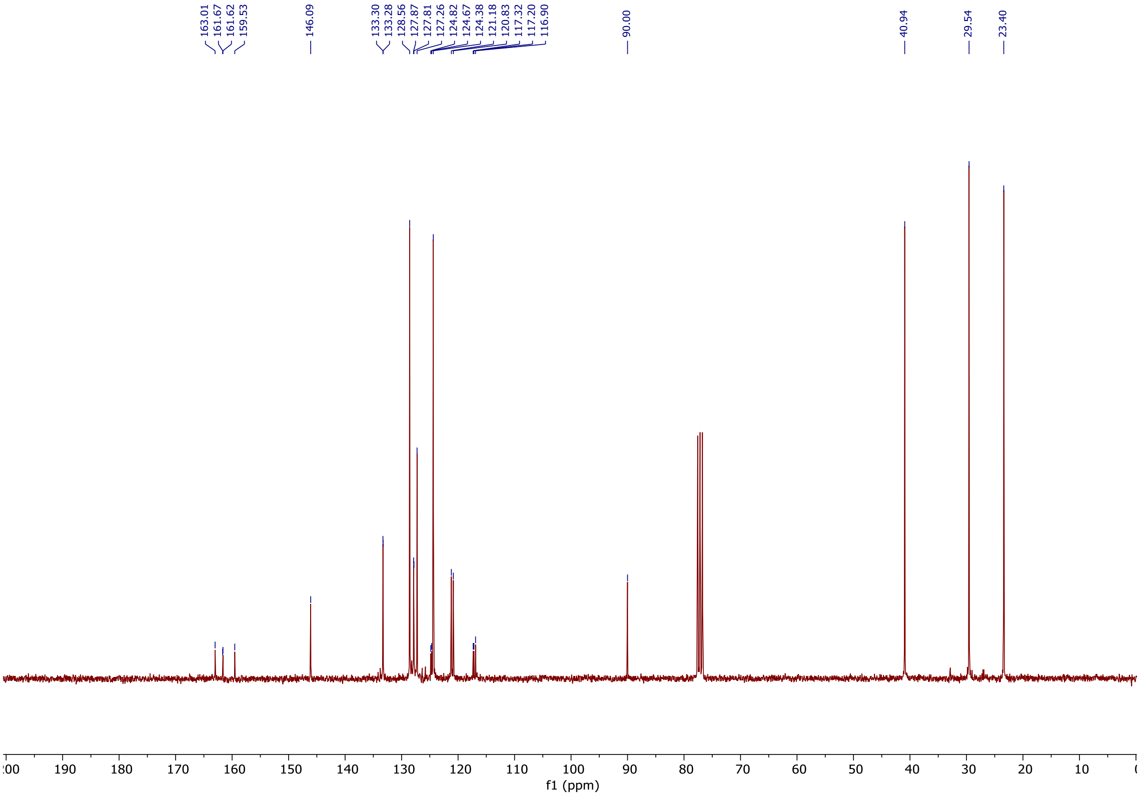

163.01 161.67 161.62 159.53
146.09
133.30 133.28 128.56 127.87 127.81 127.26 124.82 124.67 124.38 121.18 120.83 117.32 117.20 116.90
90.00
40.94
29.54
23.40
f1 (ppm)

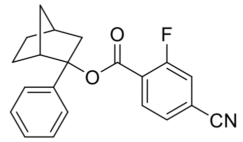
O
F
O
CN

S20

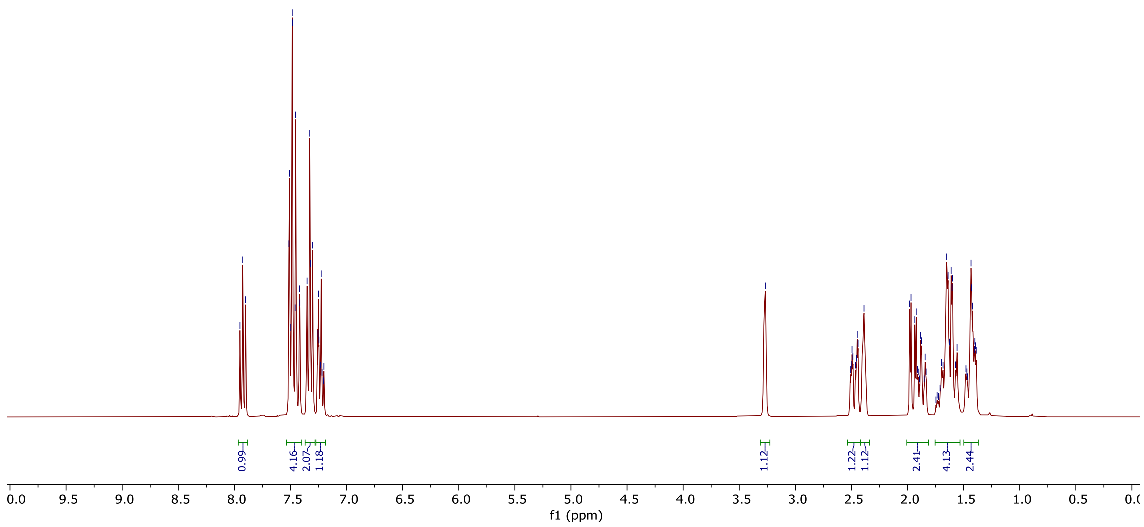
7.95 7.93 7.90 7.51 7.51 7.50 7.48 7.48 7.46 7.45 7.42 7.42 7.35 7.33 7.32 7.30 7.25 7.25 7.25 7.23 7.22 7.21 7.20
3.27 2.51 2.50 2.49 2.49 2.46 2.45 2.44 2.39 1.98 1.97 1.93 1.92 1.91 1.90 1.89 1.88 1.87 1.85 1.84 1.83 1.75 1.74 1.72 1.71 1.70 1.68 1.65 1.64 1.63 1.61 1.60 1.60 1.57 1.56 1.48 1.47 1.46 1.43 1.43 1.42 1.40 1.40 1.39 1.39
0.99
4.16
2.07
1.18
1.12
1.22
1.12
2.41
4.13
2.44
10.0 9.5 9.0 8.5 8.0 7.5 7.0 6.5 6.0 5.5 5.0 4.5 4.0 3.5 3.0 2.5 2.0 1.5 1.0 0.5 0.0
f1 (ppm)

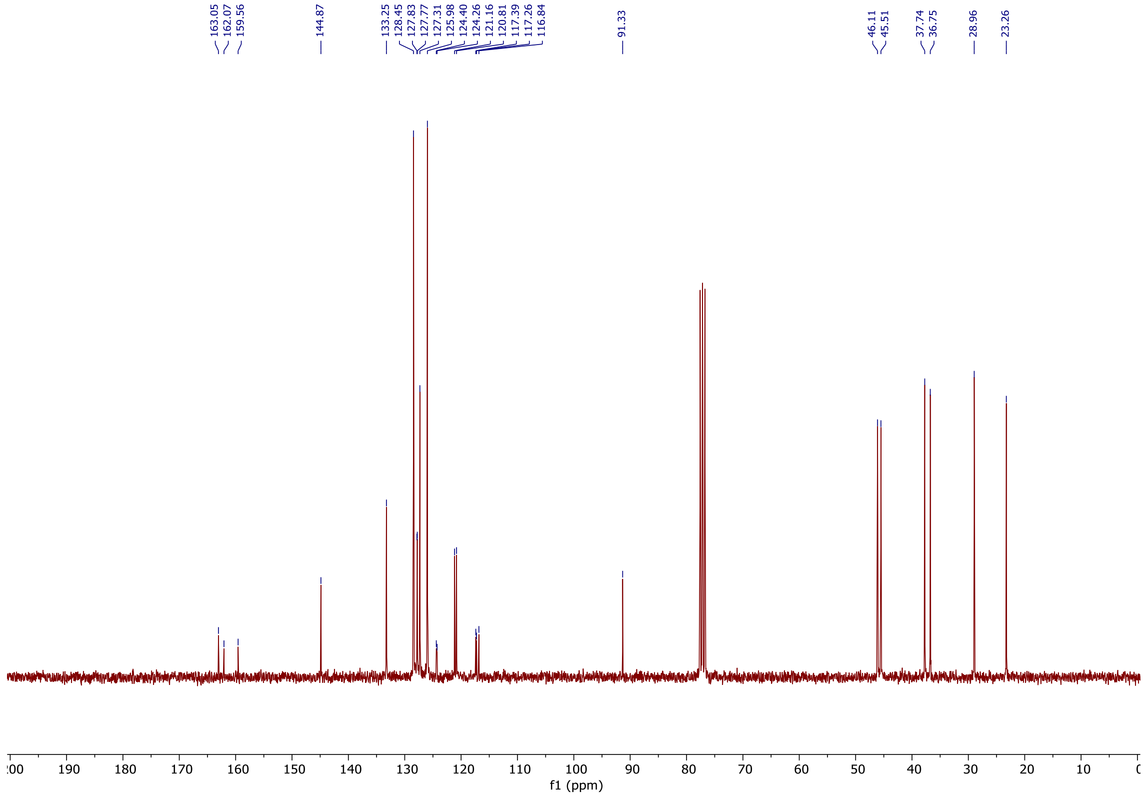
163.05 162.07 159.56
144.87
133.25 128.45 127.83 127.77 127.31 125.98 124.40 124.26 121.16 120.81 117.39 117.26 116.84
91.33
46.11 45.51
37.74 36.75
28.96
23.26
200 190 180 170 160 150 140 130 120 110 100 90 80 70 60 50 40 30 20 10 0
f1 (ppm)

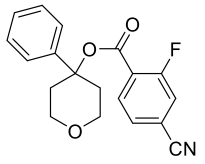
O
F
O
O
CN

S21

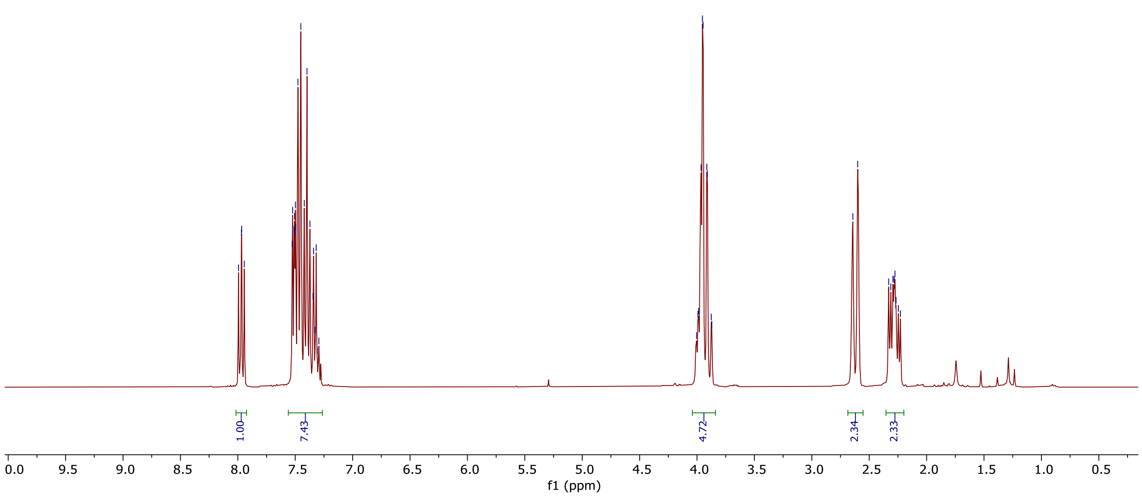
7.99 7.97 7.97 7.94 7.53 7.52 7.51 7.51 7.50 7.50 7.48 7.45 7.42 7.40 7.37 7.35 7.34 7.33 7.32 7.31 7.29
4.00 3.99 3.99 3.96 3.95 3.95 3.91 3.91 3.88 3.87
2.64 2.60 2.33 2.31 2.29 2.28 2.28 2.27 2.25 2.23
1.00
7.43
4.72
2.34
2.33
f1 (ppm)

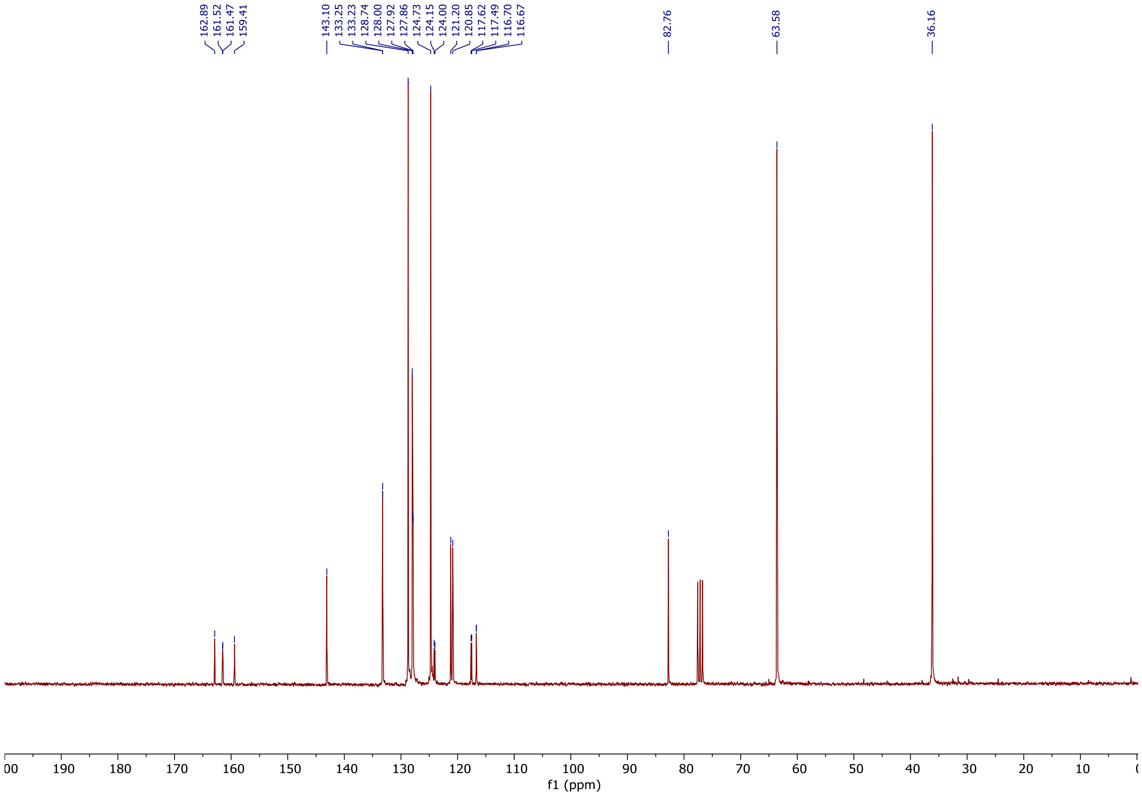
162.89 161.52 161.47 159.41
143.10 133.25 133.23 128.74 128.00 127.92 127.86 124.73 124.15 124.00 121.20 120.85 117.62 117.49 116.70 116.67
82.76
63.58
36.16
f1 (ppm)

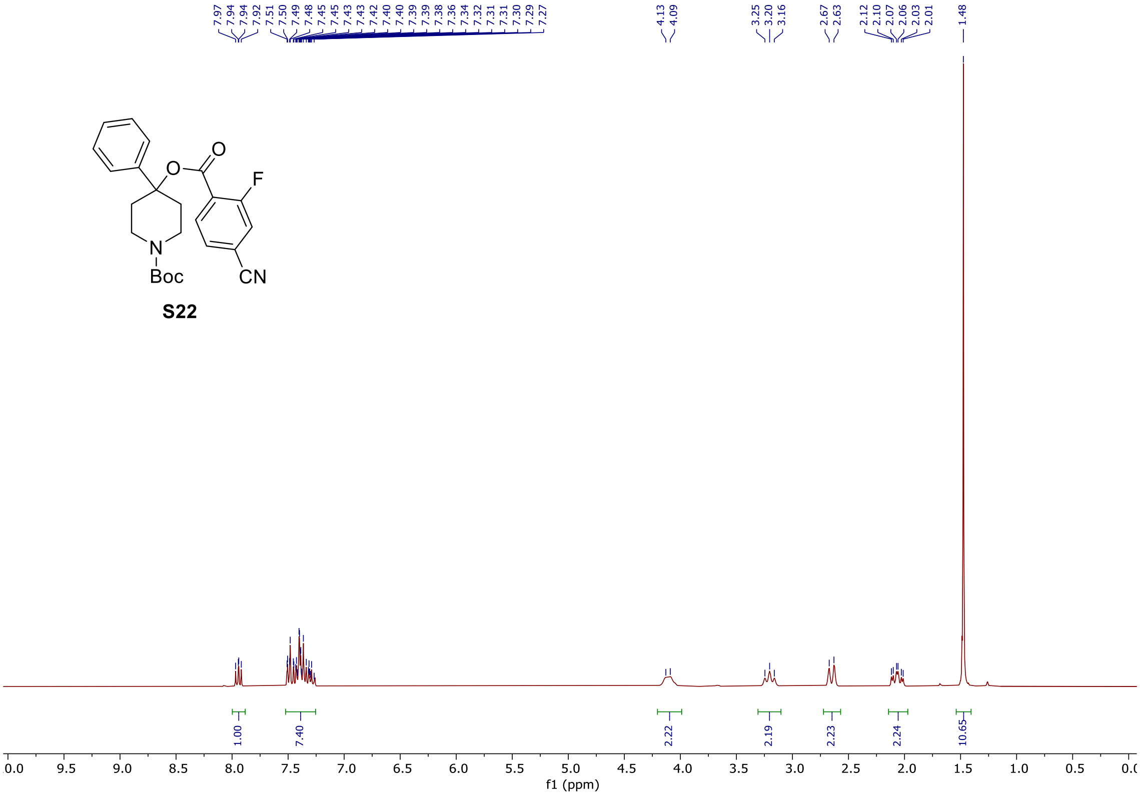
O
F
O
N
Boc
CN

S22

7.97
7.94
7.94
7.92
7.51
7.50
7.49
7.48
7.45
7.45
7.43
7.42
7.40
7.40
7.39
7.39
7.38
7.36
7.34
7.32
7.31
7.31
7.30
7.29
7.27
4.13
4.09
3.25
3.20
3.16
2.67
2.63
2.12
2.10
2.07
2.06
2.03
2.01
1.48
1.00
7.40
2.22
2.19
2.23
2.24
10.65
f1 (ppm)

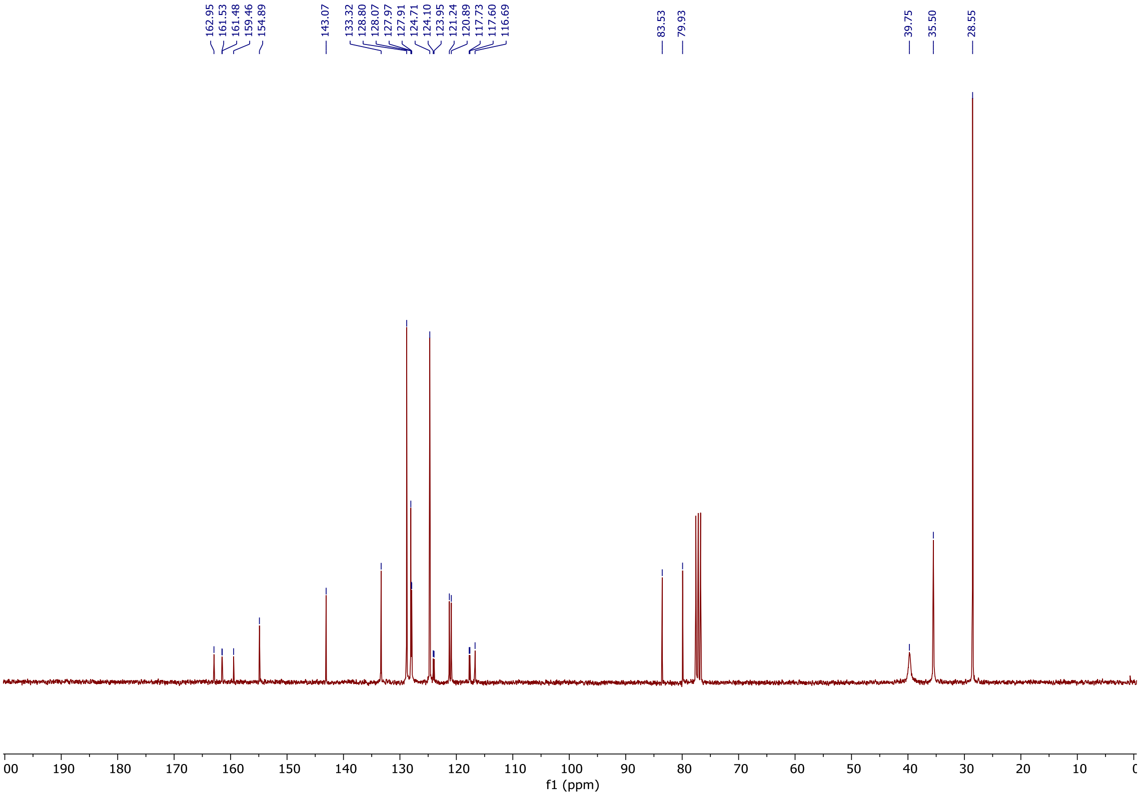
162.95
161.53
161.48
159.46
154.89
143.07
133.32
128.80
128.07
127.97
127.91
124.71
124.10
123.95
121.24
120.89
117.73
117.60
116.69
83.53
79.93
39.75
35.50
28.55
f1 (ppm)

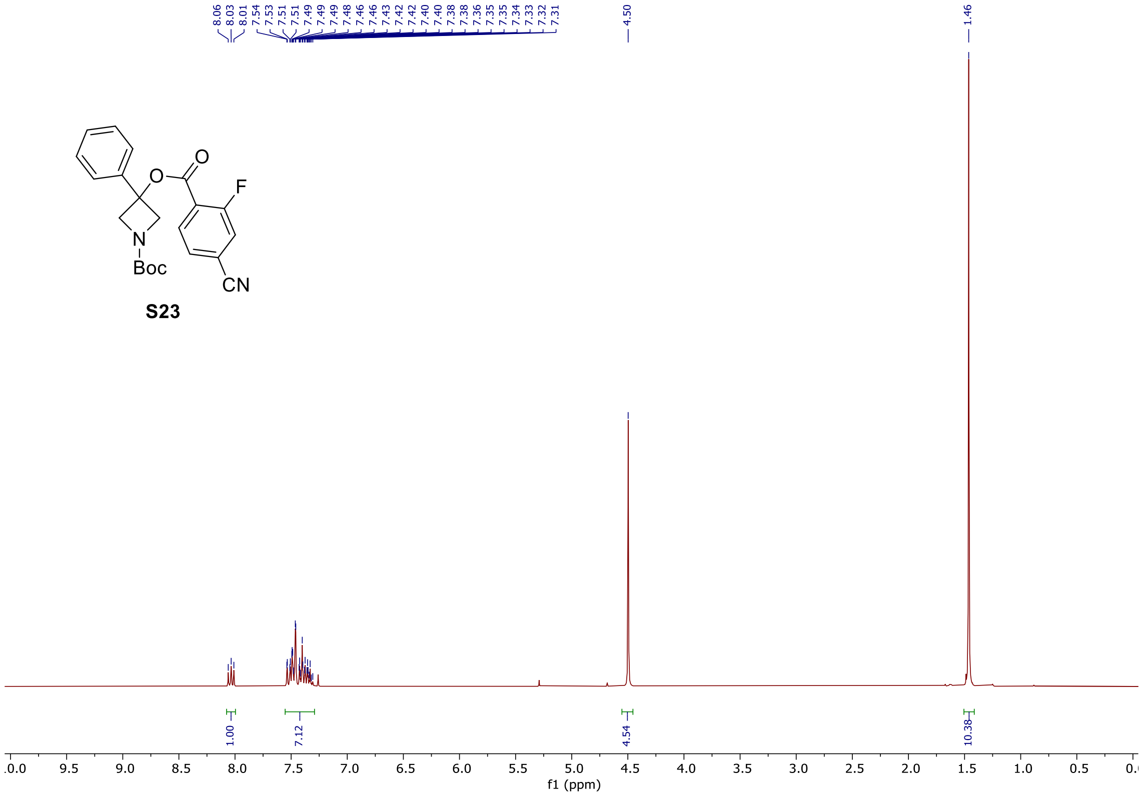
8.06
8.03
8.01
7.54
7.53
7.51
7.51
7.49
7.49
7.48
7.46
7.46
7.43
7.42
7.42
7.40
7.40
7.38
7.38
7.36
7.35
7.35
7.34
7.33
7.32
7.31
4.50
1.46
O
O
F
N
Boc
CN
S23
1.00
7.12
4.54
10.38
f1 (ppm)

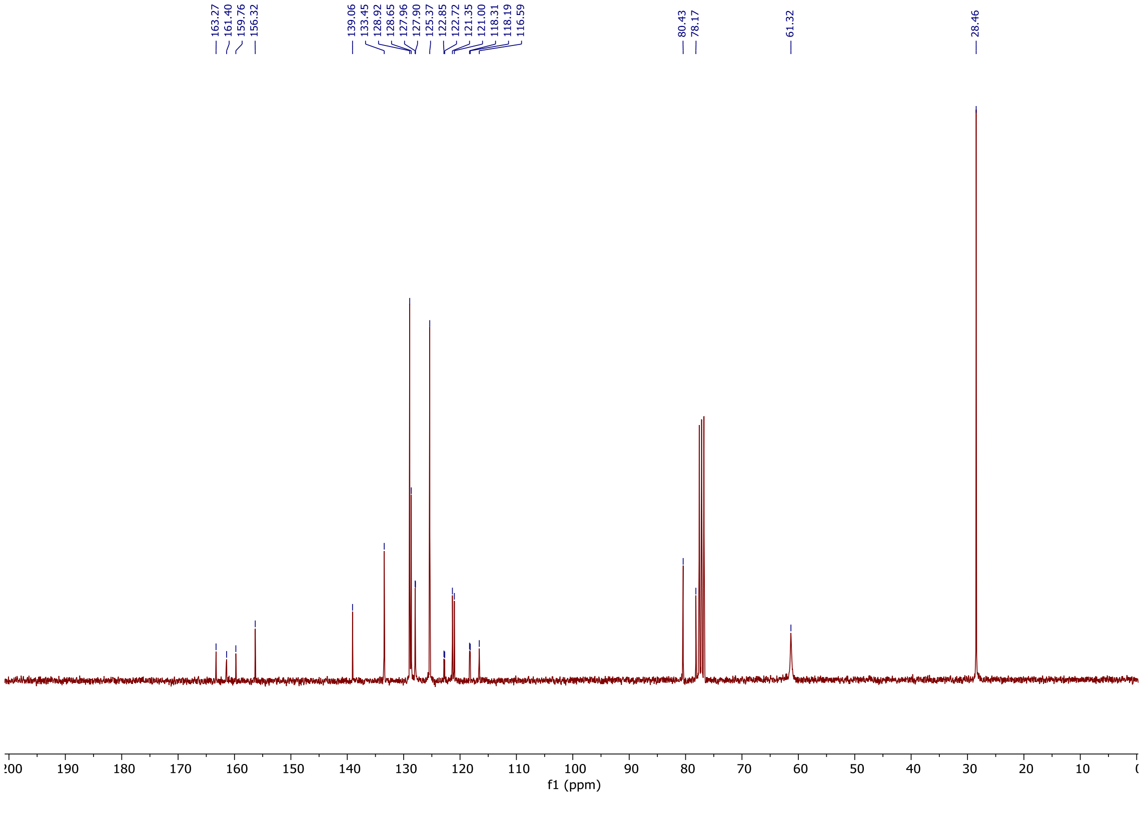
163.27
161.40
159.76
156.32
139.06
133.45
128.92
128.65
127.96
127.90
125.37
122.85
122.72
121.35
121.00
118.31
118.19
116.59
80.43
78.17
61.32
28.46
f1 (ppm)

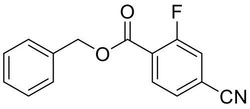

O
F
O
CN

S26

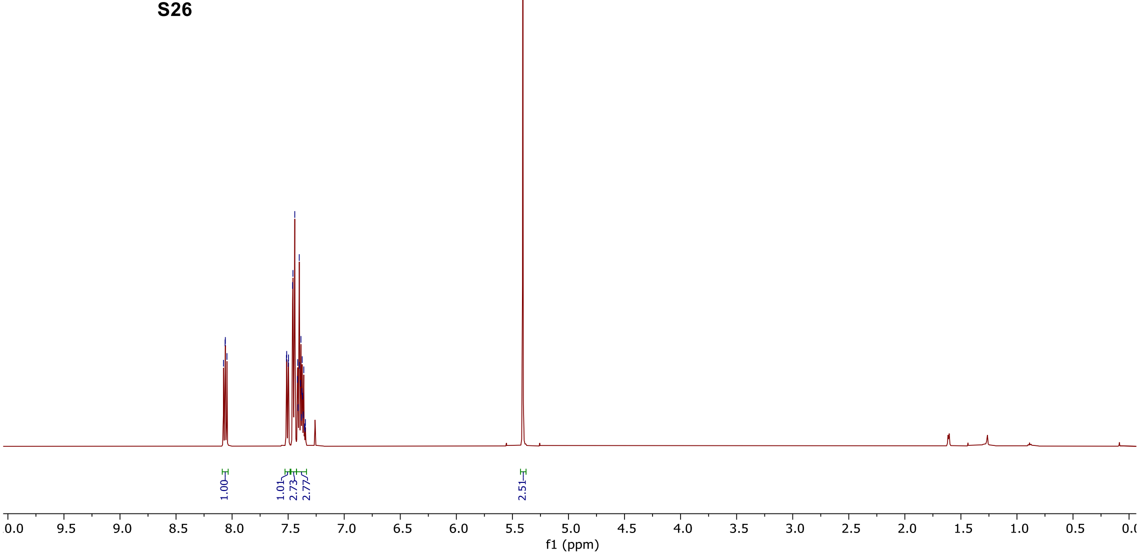

8.08 8.06 8.06 8.05 7.52 7.51 7.50 7.50 7.46 7.46 7.44 7.42 7.41 7.41 7.40 7.40 7.39 7.39 7.38 7.38 7.37 7.37 7.37 7.36 7.35 7.35 7.35 5.41
1.00
1.01
2.73
2.77
2.51
f1 (ppm)

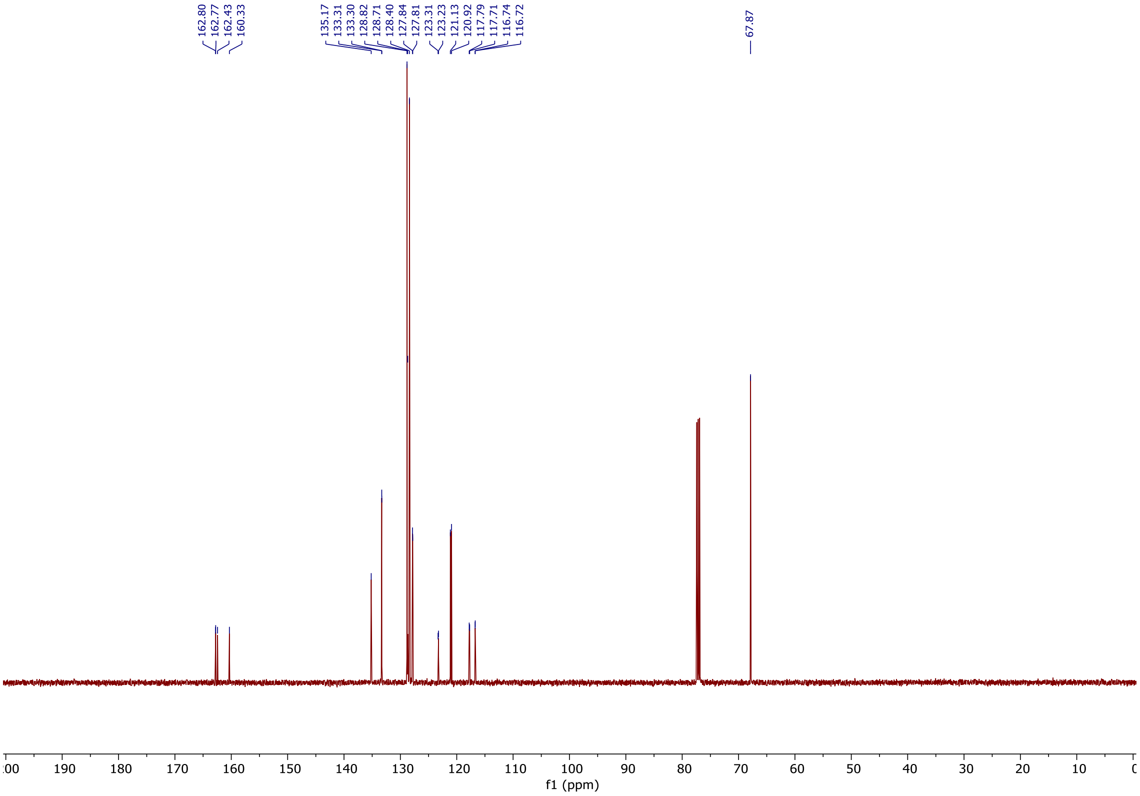

162.80 162.77 162.43 160.33
135.17 133.31 133.30 128.82 128.71 128.40 127.84 127.81 123.31 123.23 121.13 120.92 117.79 117.71 116.74 116.72
67.87
f1 (ppm)

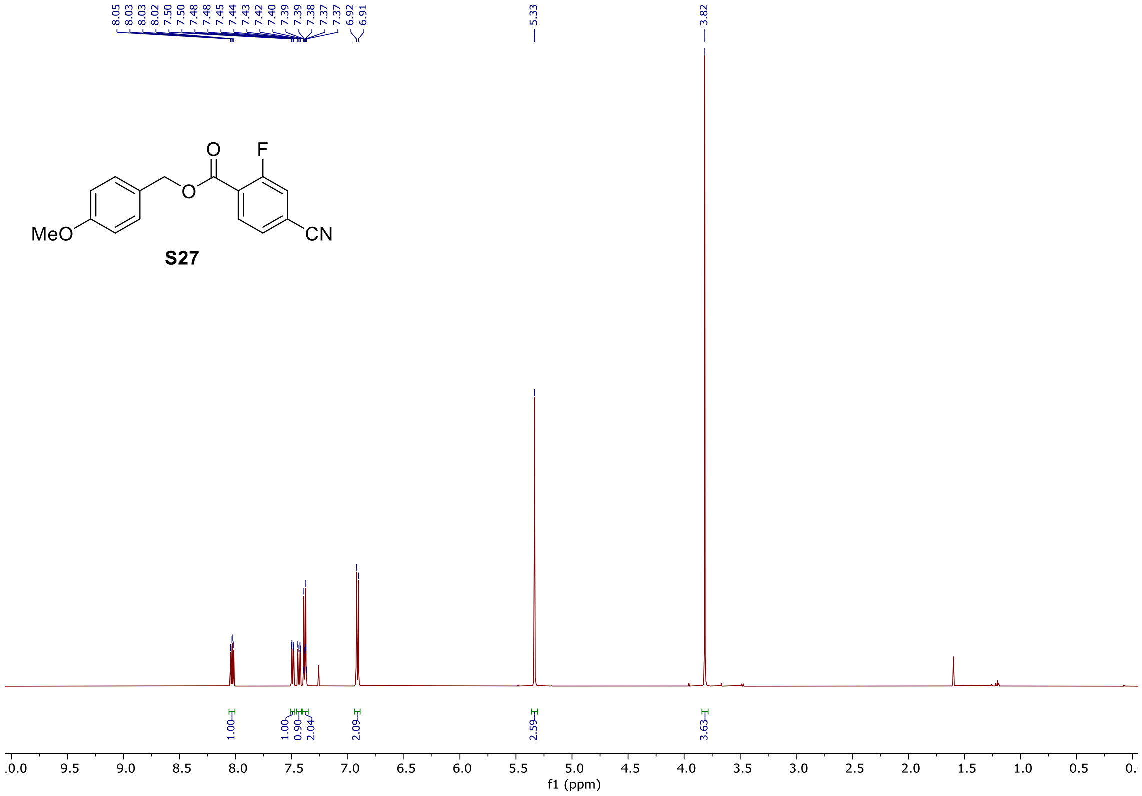
8.05
8.03
8.03
8.02
7.50
7.50
7.48
7.48
7.45
7.44
7.43
7.42
7.40
7.39
7.39
7.38
7.37
7.37
6.92
6.91
5.33
3.82
MeO
O
O
F
CN
S27
1.00
1.00
0.90
2.04
2.09
2.59
3.63
f1 (ppm)

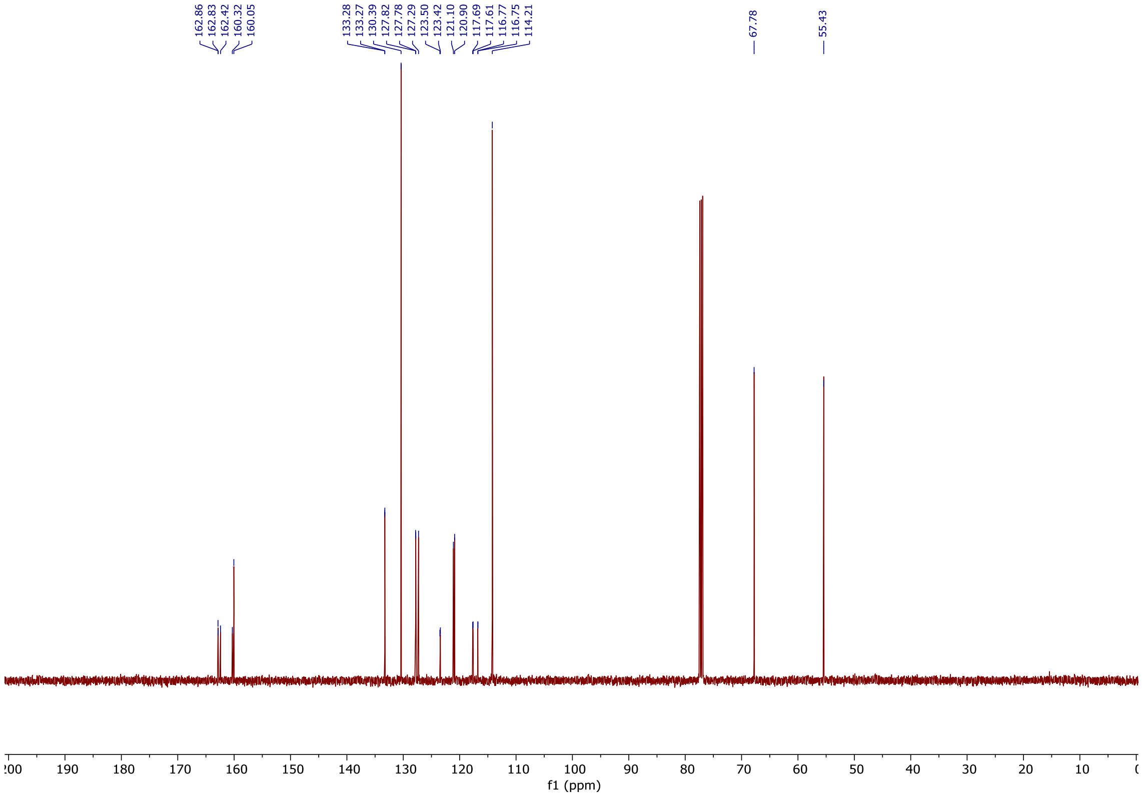
162.86
162.83
162.42
160.32
160.05
133.28
133.27
130.39
127.82
127.78
127.29
123.50
123.42
121.10
120.90
117.69
117.61
116.77
116.75
114.21
67.78
55.43
f1 (ppm)

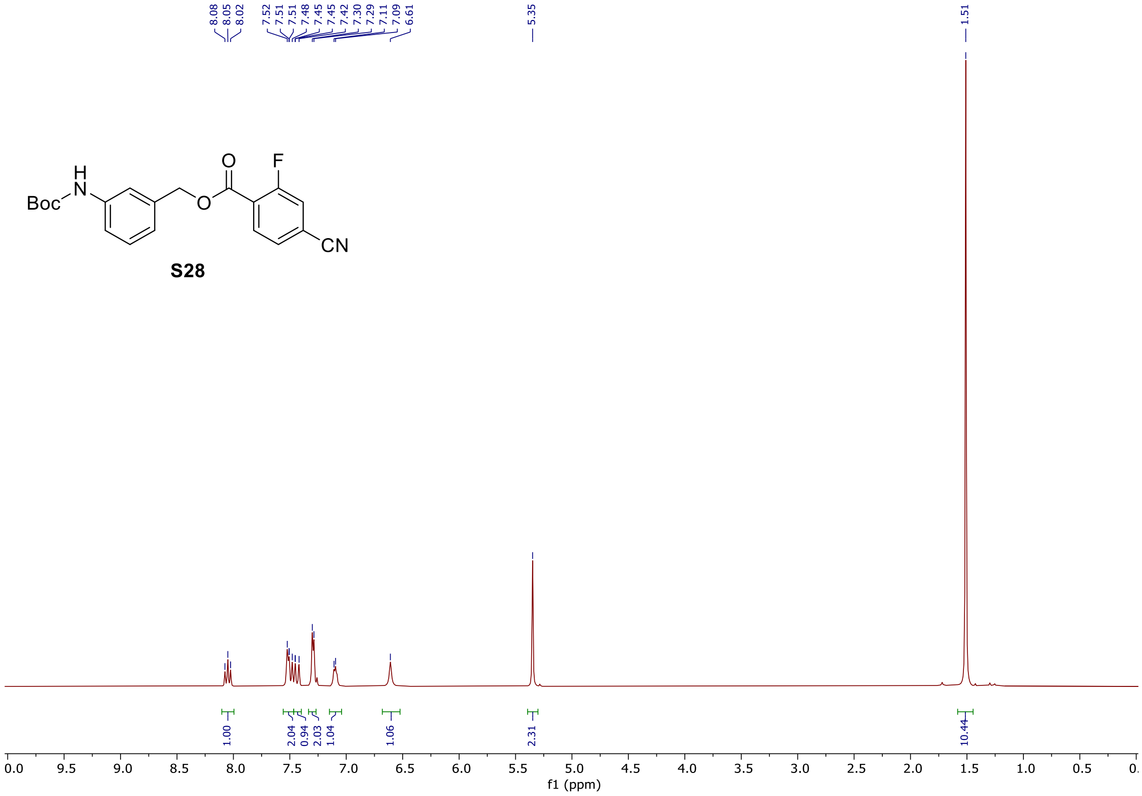
Boc
N
H
O
O
F
CN
8.08
8.05
8.02
7.52
7.51
7.51
7.48
7.45
7.45
7.42
7.30
7.29
7.11
7.09
6.61
5.35
1.51
1.00
2.04
0.94
2.03
1.04
1.06
2.31
10.44
f1 (ppm)

S28

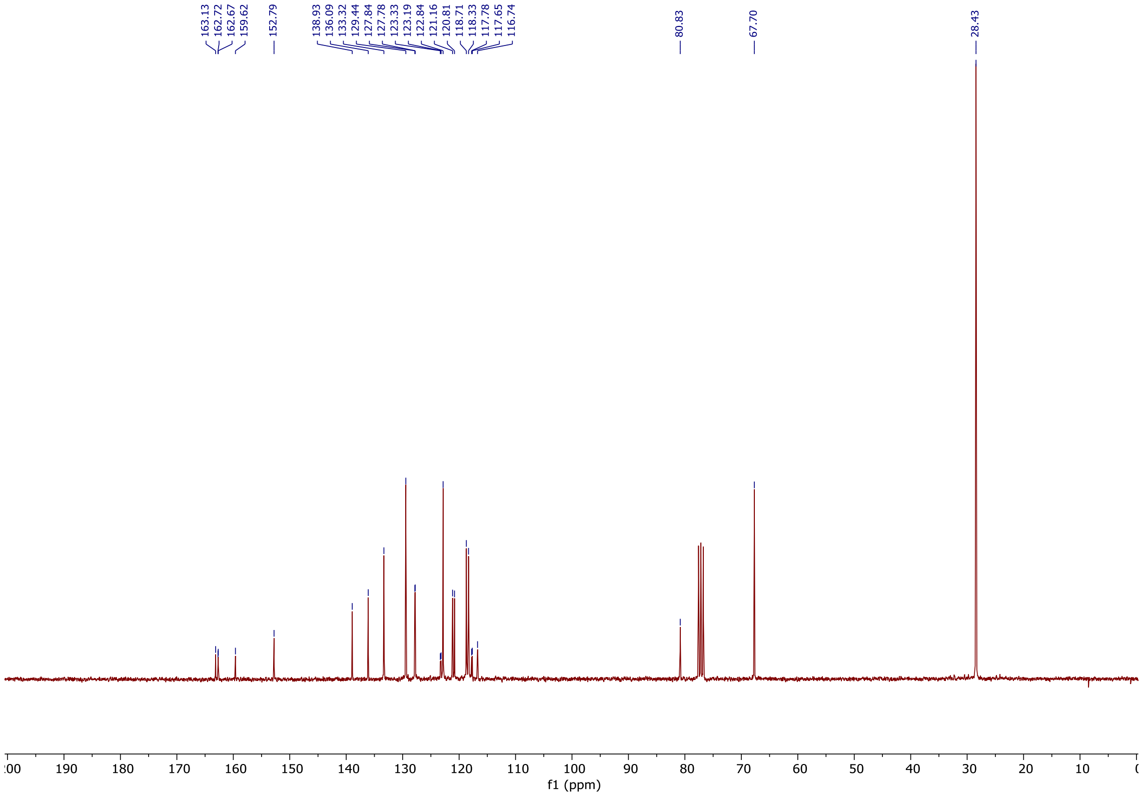
163.13
162.72
162.67
159.62
152.79
138.93
136.09
133.32
129.44
127.84
127.78
123.33
123.19
122.84
121.16
120.81
118.71
118.33
117.78
117.65
116.74
80.83
67.70
28.43
f1 (ppm)

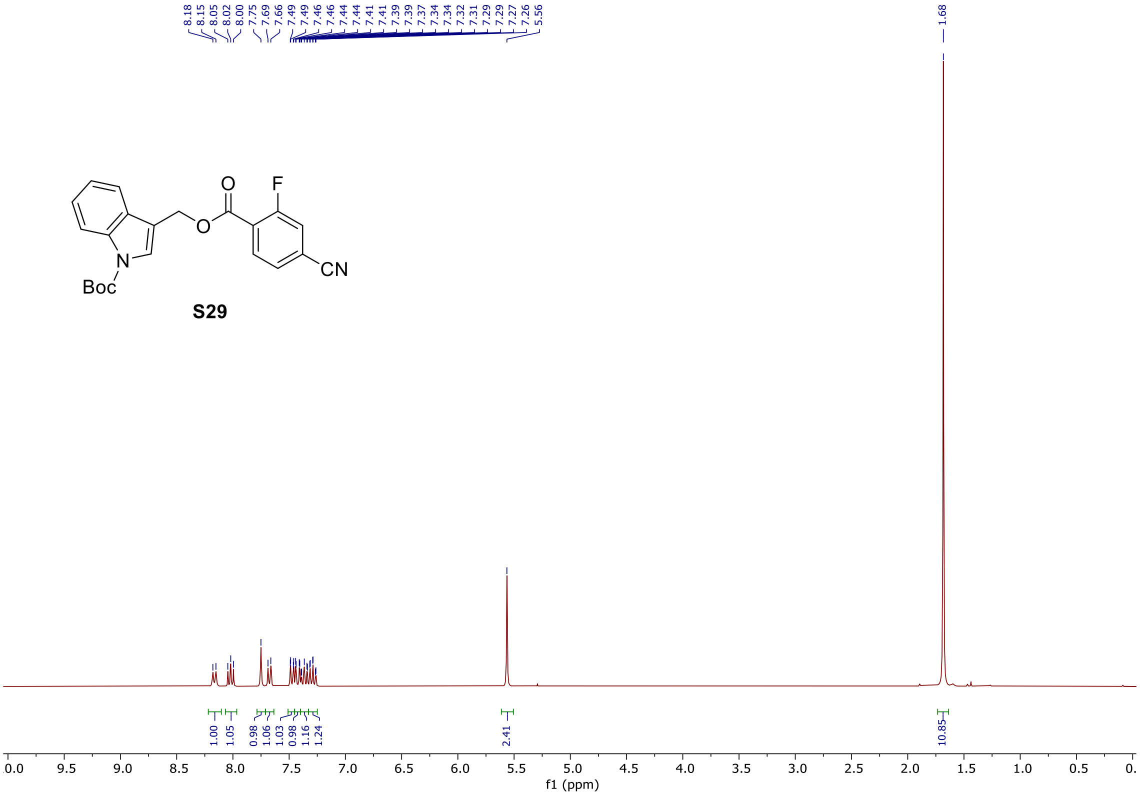
8.18
8.15
8.05
8.02
8.00
7.75
7.69
7.66
7.49
7.49
7.46
7.46
7.44
7.44
7.41
7.41
7.39
7.39
7.37
7.34
7.34
7.32
7.31
7.29
7.29
7.27
7.26
5.56
1.68
O
F
O
N
Boc
CN
1.00
1.05
0.98
1.06
1.03
0.98
1.16
1.24
2.41
10.85
f1 (ppm)

S29

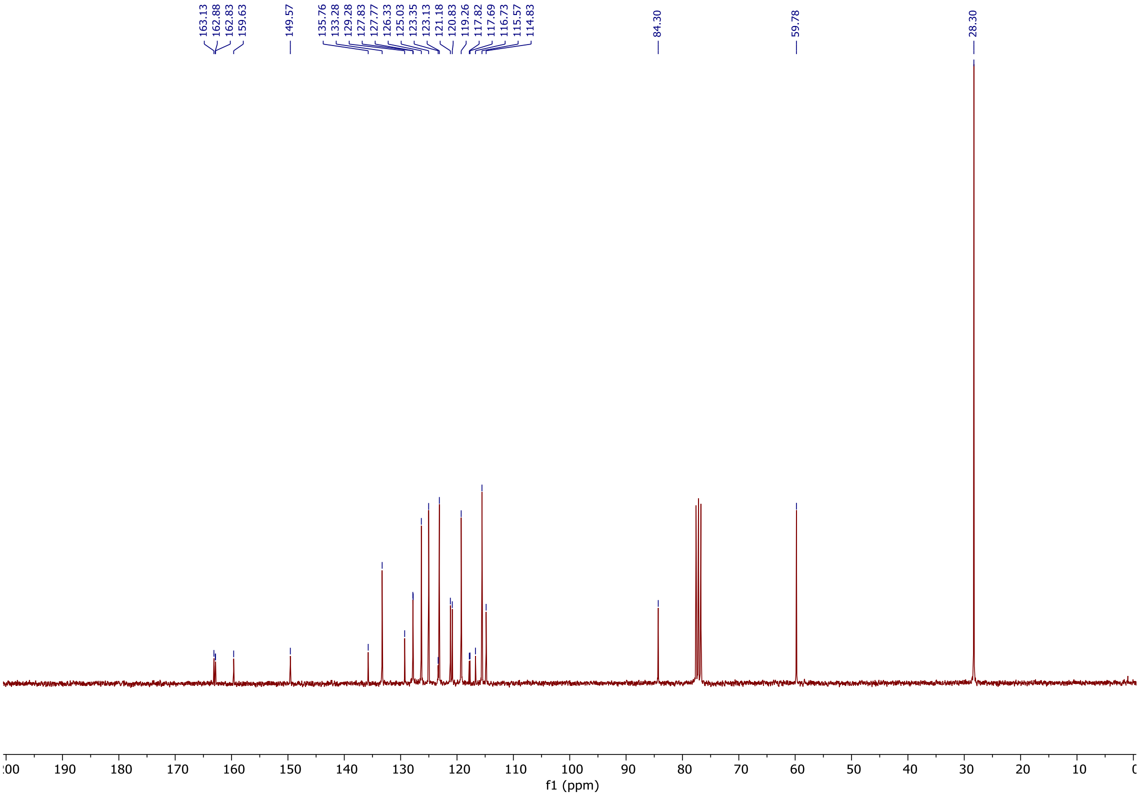
163.13
162.88
162.83
159.63
149.57
135.76
133.28
129.28
127.83
127.77
126.33
125.03
123.35
123.13
121.18
120.83
119.26
117.82
117.69
116.73
115.57
114.83
84.30
59.78
28.30
f1 (ppm)

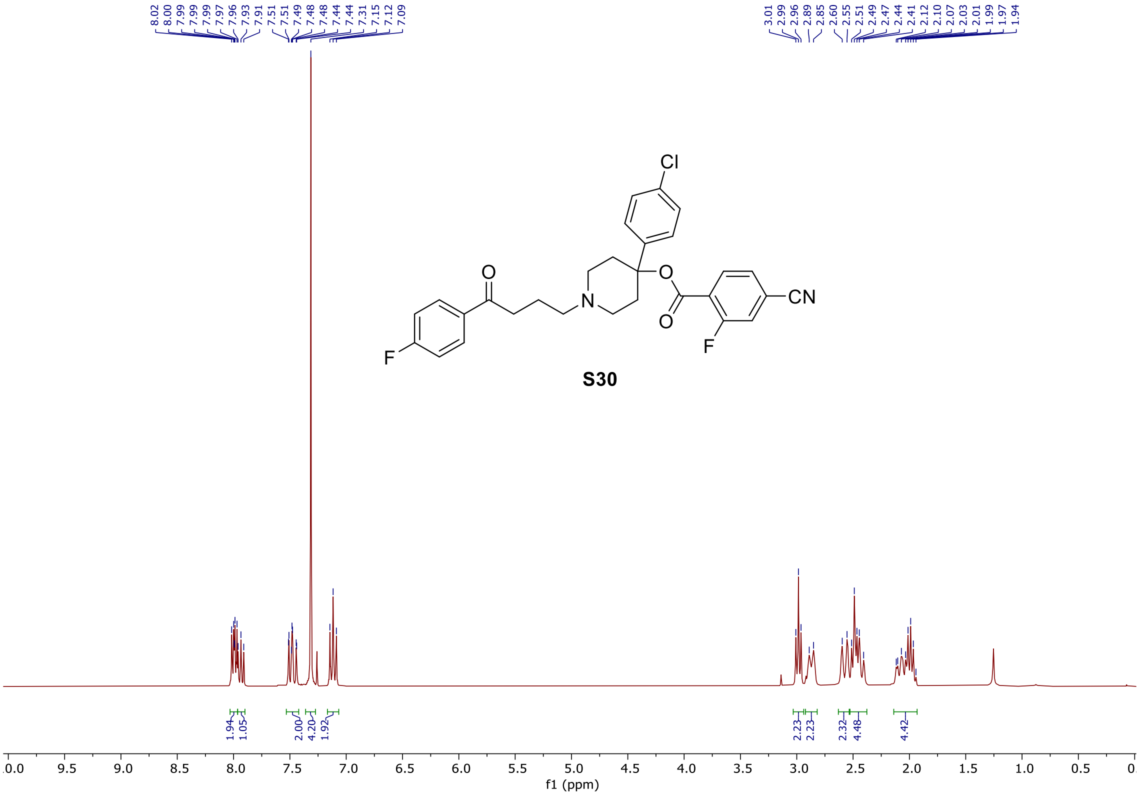
8.02 8.00 7.99 7.99 7.99 7.97 7.96 7.93 7.91 7.51 7.51 7.49 7.48 7.48 7.44 7.44 7.31 7.15 7.12 7.09
3.01 2.99 2.96 2.89 2.85 2.60 2.55 2.51 2.49 2.47 2.44 2.41 2.12 2.10 2.07 2.03 2.01 1.99 1.97 1.94
Cl
O
N
O
CN
O
F
F
S30
1.94 1.05 2.00 4.20 1.92 2.23 2.23 2.32 4.48 4.42
f1 (ppm)

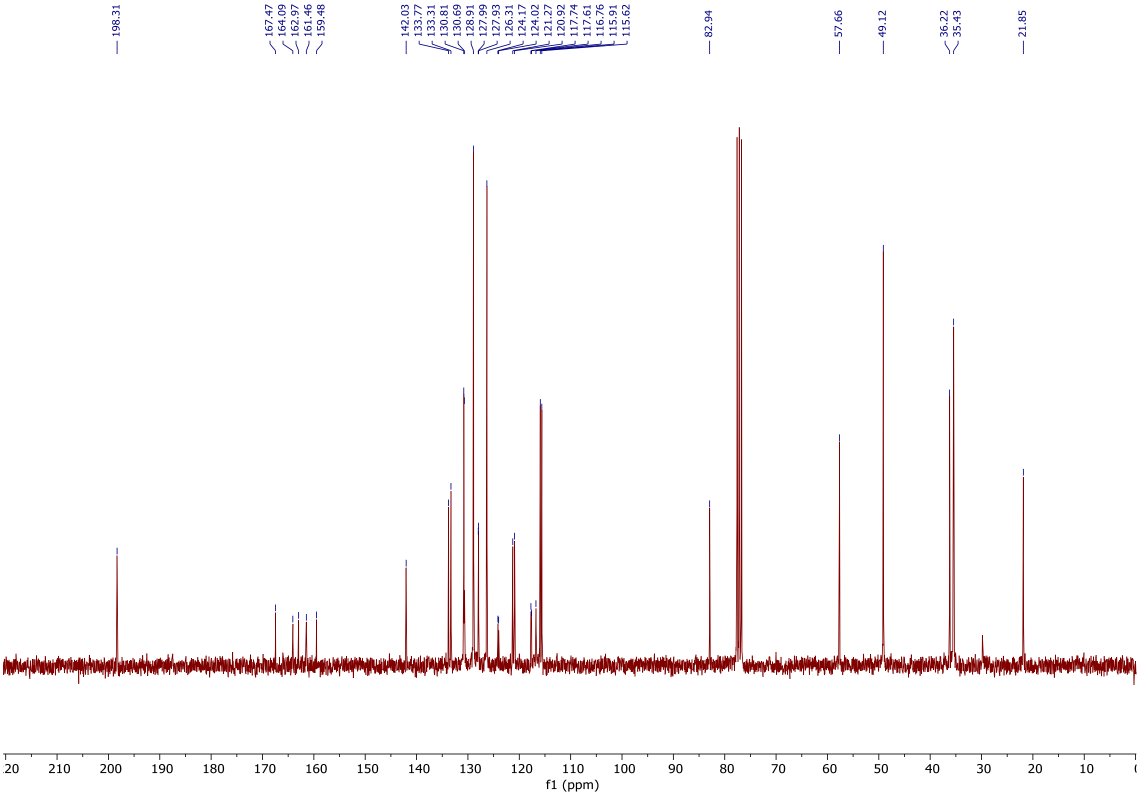
198.31
167.47 164.09 162.97 161.46 159.48
142.03 133.77 133.31 130.81 130.69 128.91 127.99 127.93 126.31 124.17 124.02 121.27 120.92 117.74 117.61 116.76 115.91 115.62
82.94
57.66
49.12
36.22 35.43
21.85
f1 (ppm)

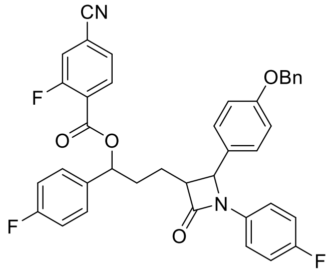
CN
F
OBn
O
O
N
O
F
F

S31

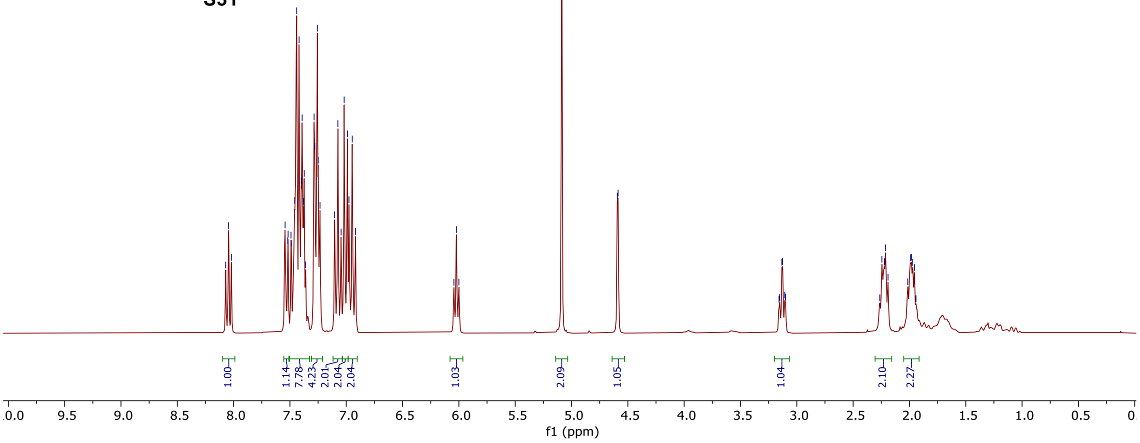
8.07 8.04 8.02 7.54 7.52 7.49 7.49 7.46 7.44 7.42 7.40 7.39 7.38 7.37 7.36 7.29 7.28 7.26 7.25 7.23 7.10 7.07 7.05 7.02 6.99 6.98 6.95 6.92 6.04 6.02 6.00
5.09
4.59 4.59
3.16 3.15 3.13 3.13 3.11 3.10 2.26 2.24 2.22 2.21 2.19 2.01 1.99 1.98 1.97 1.96 1.94
1.00 1.14 7.78 4.23 2.01 2.04 2.04 1.03 2.09 1.05 1.04 2.10 2.27
9.5 9.0 8.5 8.0 7.5 7.0 6.5 6.0 5.5 5.0 4.5 4.0 3.5 3.0 2.5 2.0 1.5 1.0 0.5
f1 (ppm)

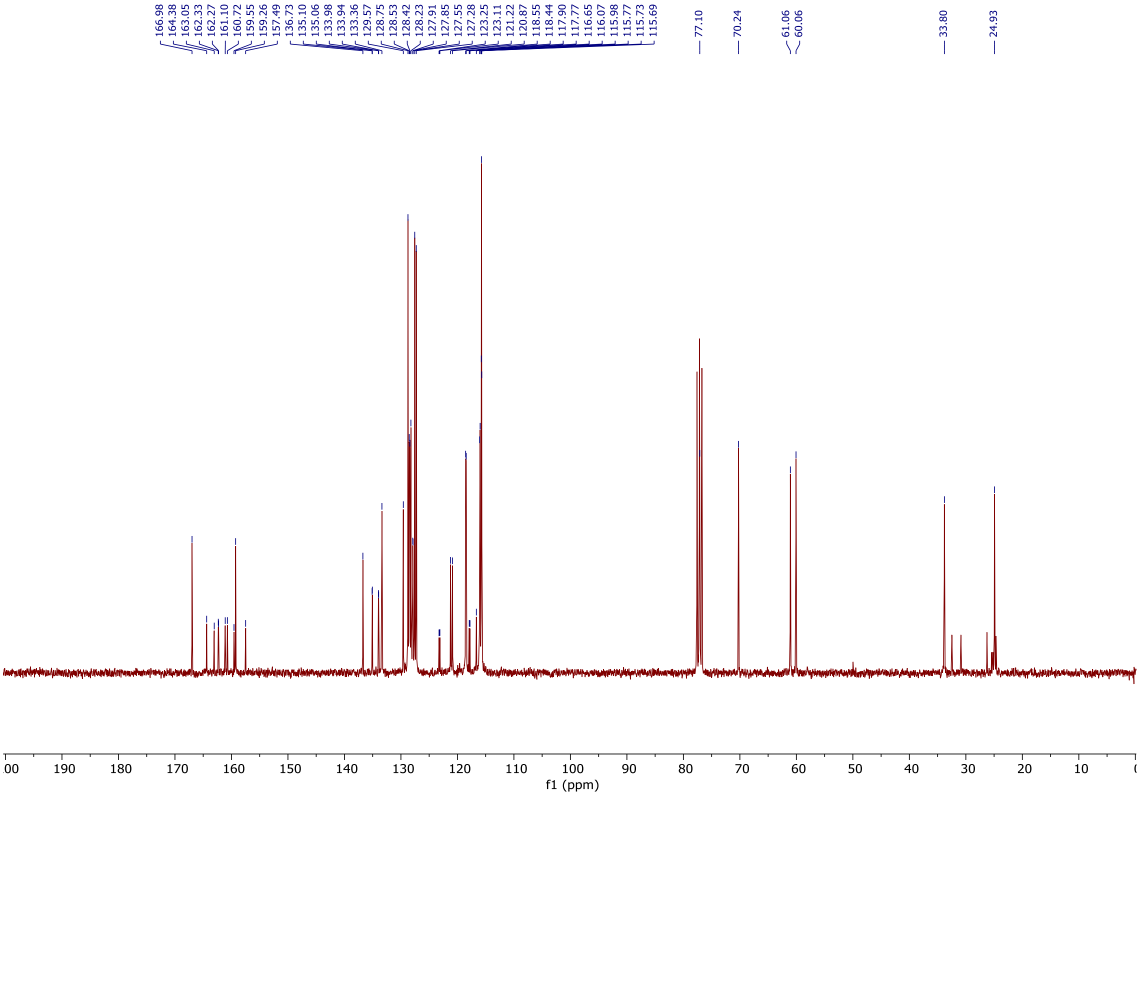
166.98 164.38 163.05 162.33 162.27 161.10 160.72 159.55 159.26 157.49 136.73 135.10 135.06 133.98 133.94 133.36 129.57 128.75 128.53 128.42 128.23 127.91 127.85 127.55 127.28 123.25 123.11 121.22 120.87 118.55 118.44 117.90 117.77 116.65 116.07 115.98 115.77 115.73 115.69
77.10
70.24
61.06 60.06
33.80
24.93
190 180 170 160 150 140 130 120 110 100 90 80 70 60 50 40 30 20 10
f1 (ppm)

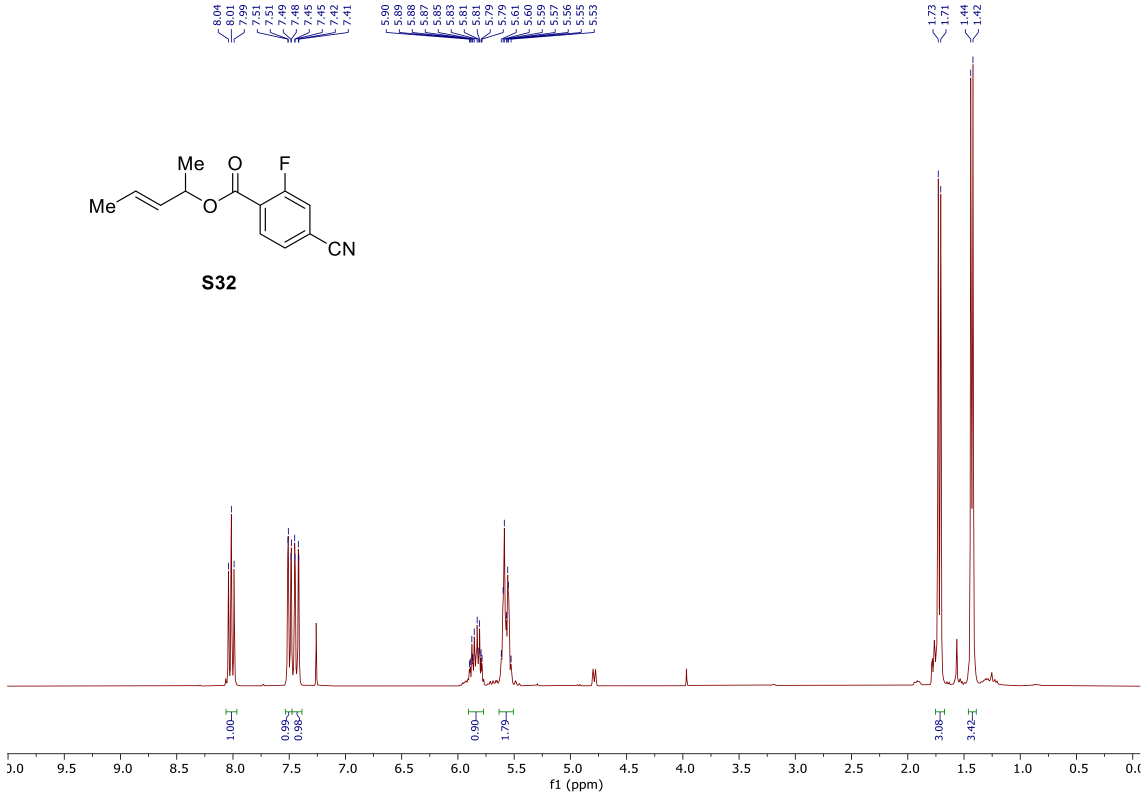
8.04
8.01
7.99
7.51
7.51
7.49
7.48
7.45
7.45
7.42
7.41
5.90
5.89
5.88
5.87
5.85
5.83
5.81
5.81
5.79
5.79
5.61
5.60
5.59
5.57
5.56
5.55
5.53
1.73
1.71
1.44
1.42
Me
O
F
Me
O
CN
S32
1.00
0.99
0.98
0.90
1.79
3.08
3.42
f1 (ppm)

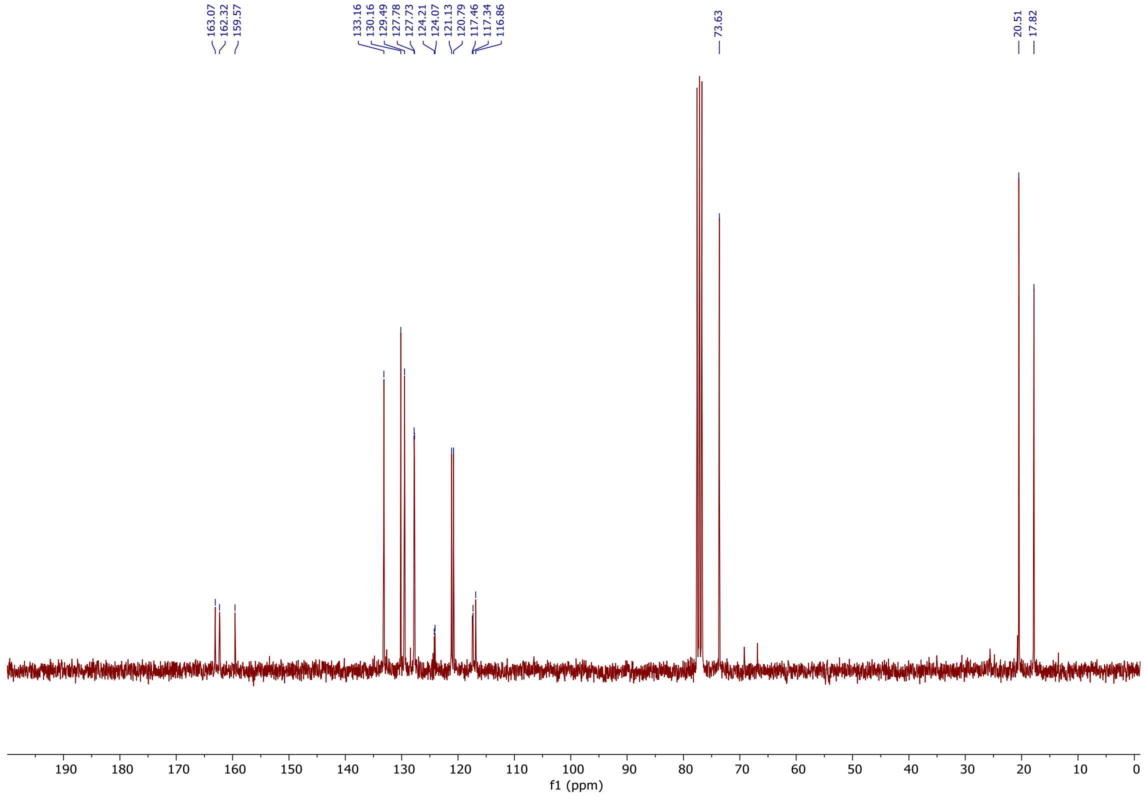
163.07
162.32
159.57
133.16
130.16
129.49
127.78
127.73
124.21
124.07
121.13
120.79
117.46
117.34
116.86
73.63
20.51
17.82
f1 (ppm)

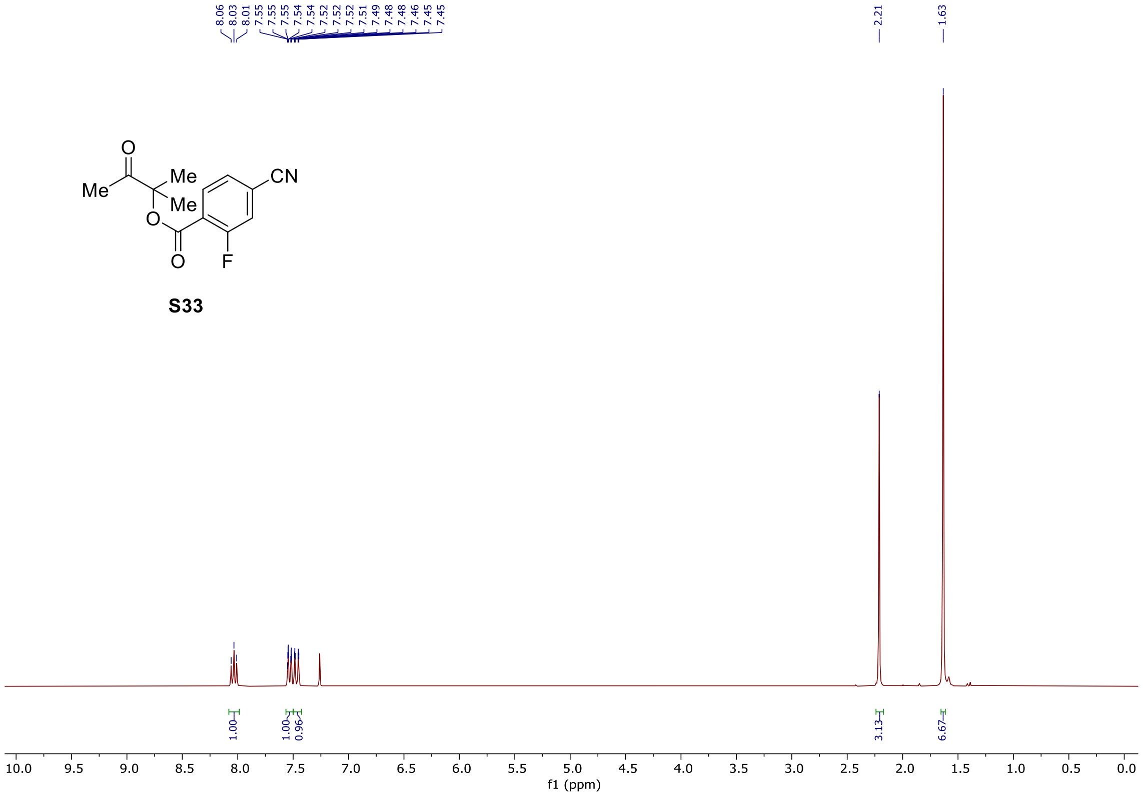

O
Me
Me
Me
CN
O
O
F
S33
8.06
8.03
8.01
7.55
7.55
7.55
7.54
7.54
7.52
7.52
7.52
7.51
7.49
7.48
7.48
7.46
7.45
7.45
2.21
1.63
1.00
1.00
0.96
3.13
6.67
10.0 9.5 9.0 8.5 8.0 7.5 7.0 6.5 6.0 5.5 5.0 4.5 4.0 3.5 3.0 2.5 2.0 1.5 1.0 0.5 0.0
f1 (ppm)

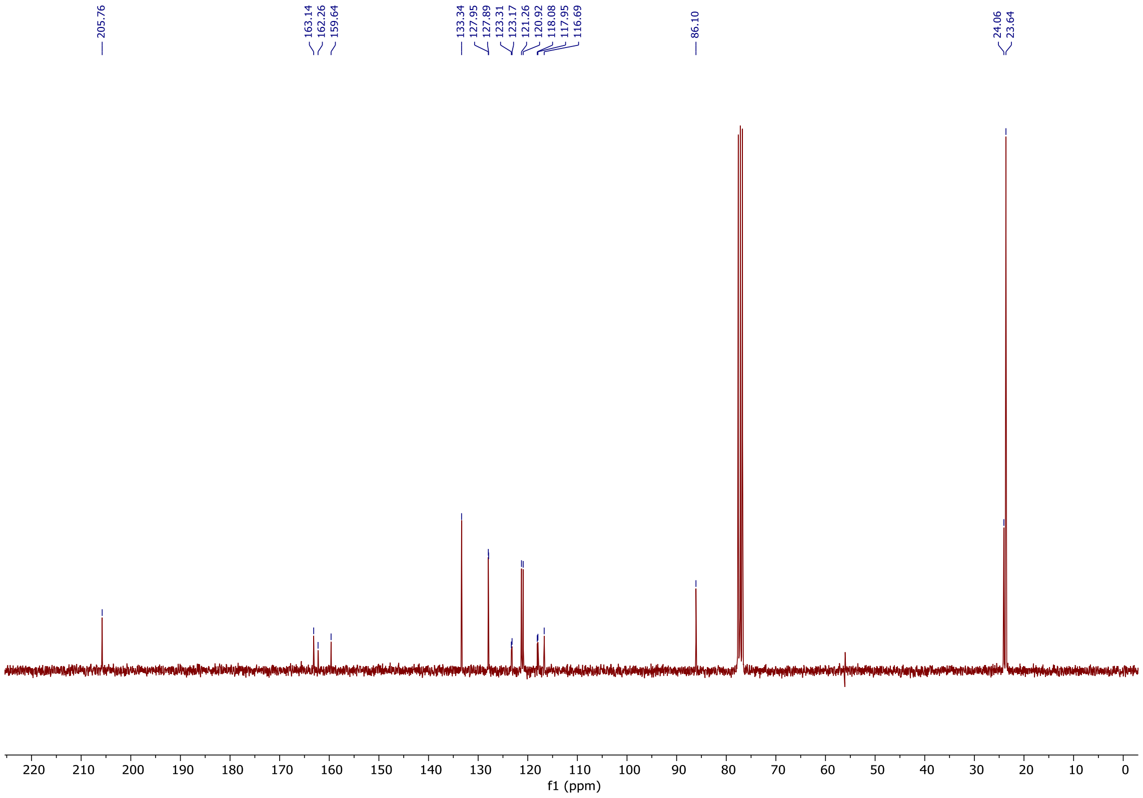

205.76
163.14
162.26
159.64
133.34
127.95
127.89
123.31
123.17
121.26
120.92
118.08
117.95
116.69
86.10
24.06
23.64
220 210 200 190 180 170 160 150 140 130 120 110 100 90 80 70 60 50 40 30 20 10 0
f1 (ppm)

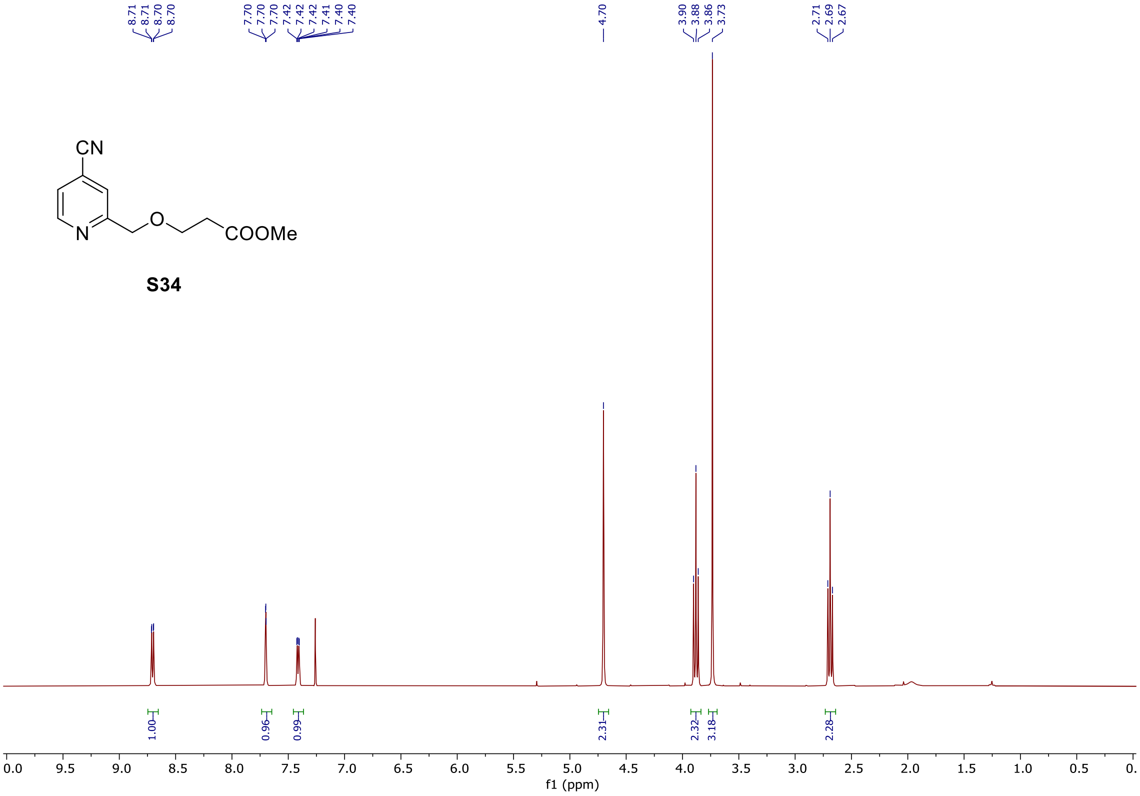
8.71
8.71
8.70
8.70
7.70
7.70
7.70
7.42
7.42
7.42
7.41
7.40
7.40
4.70
3.90
3.88
3.86
3.73
2.71
2.69
2.67
CN
N
O
COOMe
S34
1.00
0.96
0.99
2.31
2.32
3.18
2.28
10.0
9.5
9.0
8.5
8.0
7.5
7.0
6.5
6.0
5.5
5.0
4.5
4.0
3.5
3.0
2.5
2.0
1.5
1.0
0.5
0.
f1 (ppm)

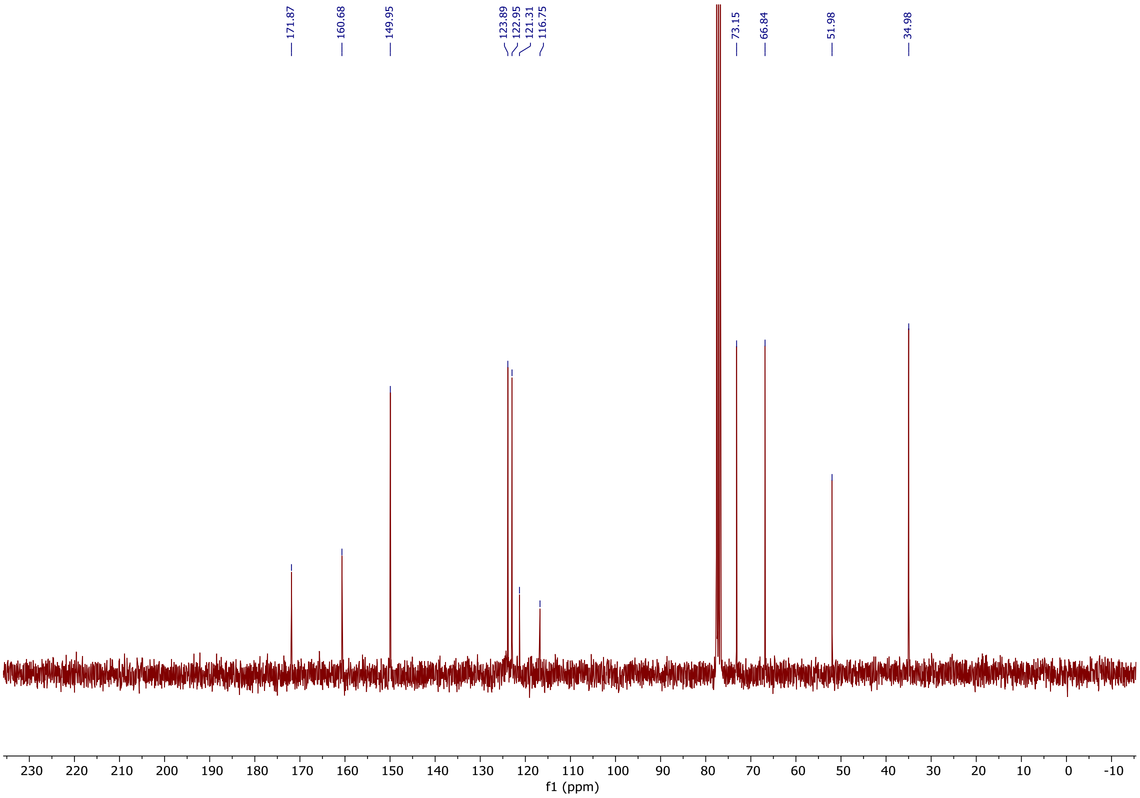
171.87
160.68
149.95
123.89
122.95
121.31
116.75
73.15
66.84
51.98
34.98
230
220
210
200
190
180
170
160
150
140
130
120
110
100
90
80
70
60
50
40
30
20
10
0
-10
f1 (ppm)

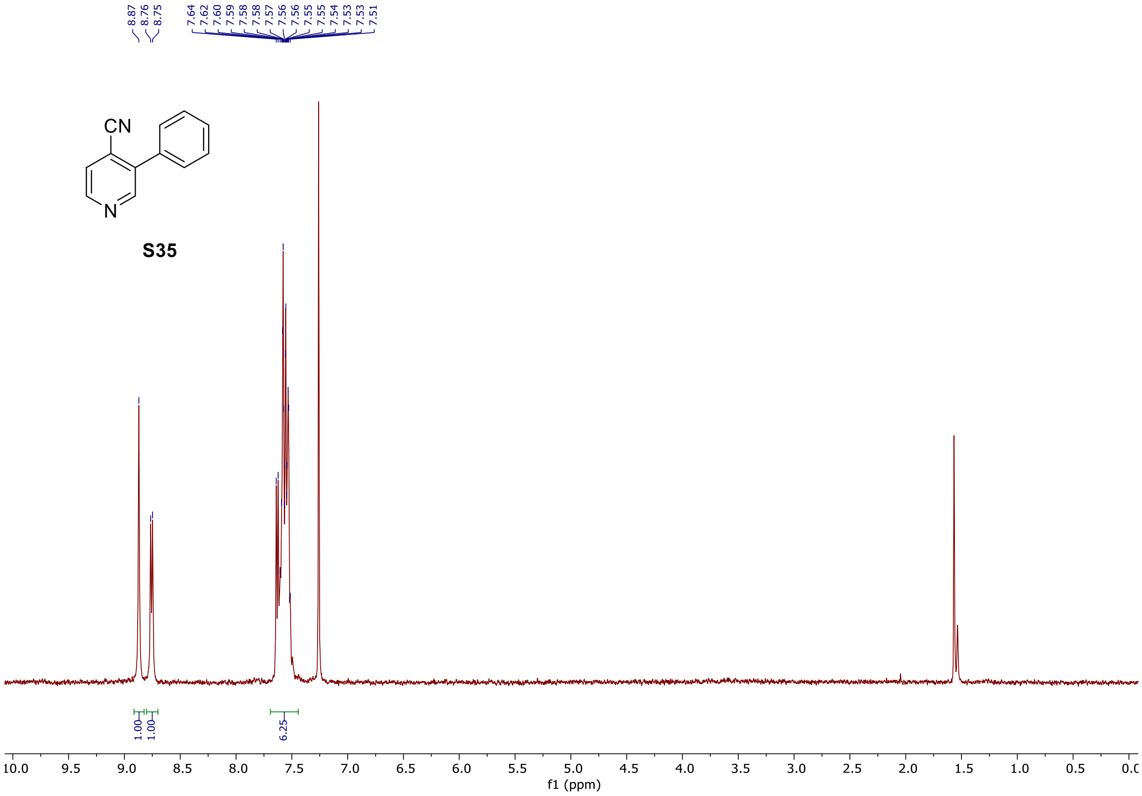
8.87
8.76
8.75
7.64
7.62
7.60
7.59
7.58
7.58
7.57
7.56
7.56
7.55
7.55
7.54
7.53
7.53
7.51
CN
N
S35
1.00
1.00
6.25
f1 (ppm)

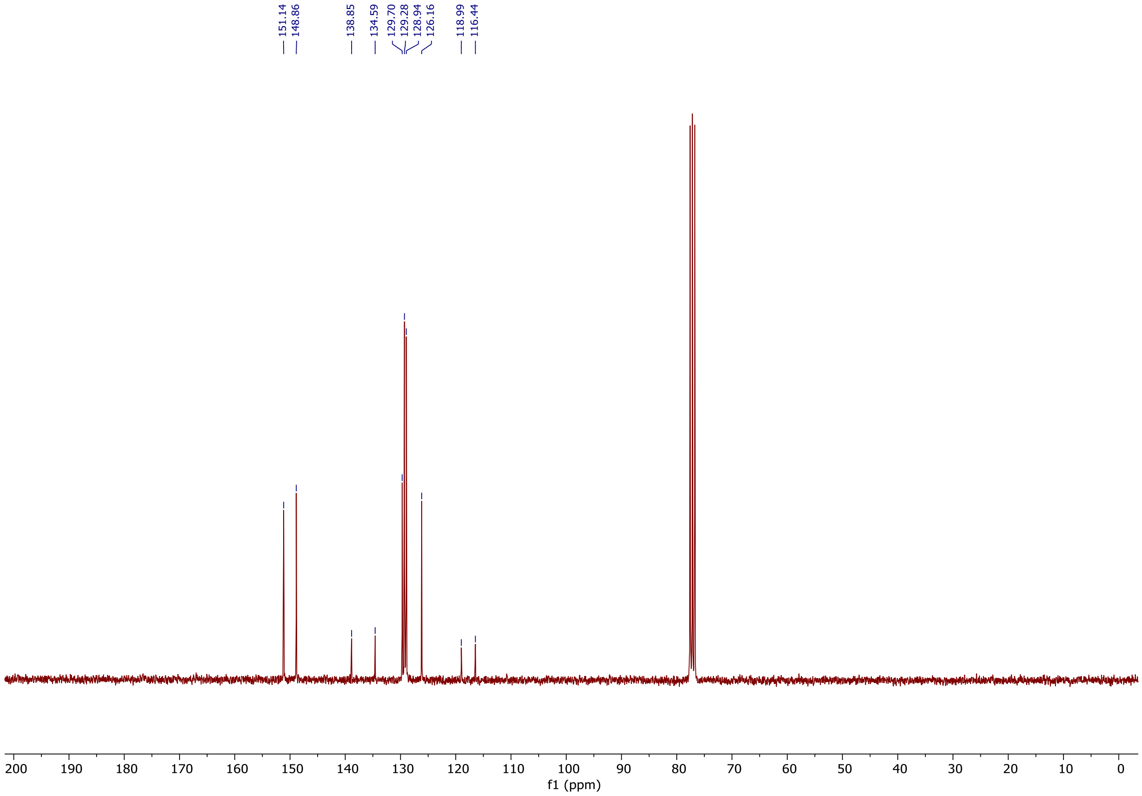
151.14
148.86
138.85
134.59
129.70
129.28
128.94
126.16
118.99
116.44
f1 (ppm)

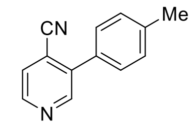
CN
Me
N

S36

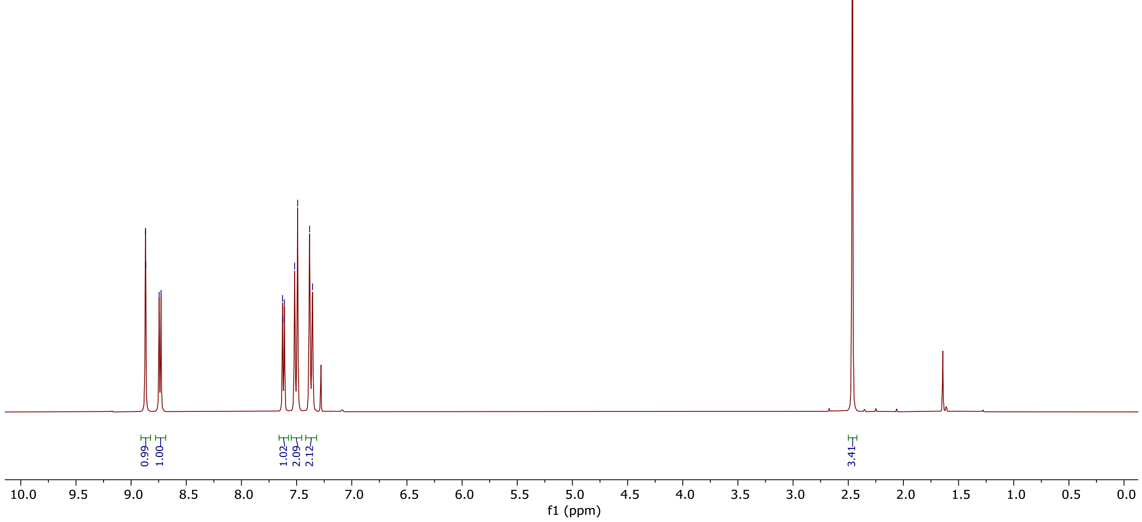
8.87
8.87
8.75
8.73
7.63
7.62
7.61
7.52
7.49
7.38
7.36
2.46
0.99
1.00
1.02
2.09
2.12
3.41
10.0 9.5 9.0 8.5 8.0 7.5 7.0 6.5 6.0 5.5 5.0 4.5 4.0 3.5 3.0 2.5 2.0 1.5 1.0 0.5 0.0
f1 (ppm)

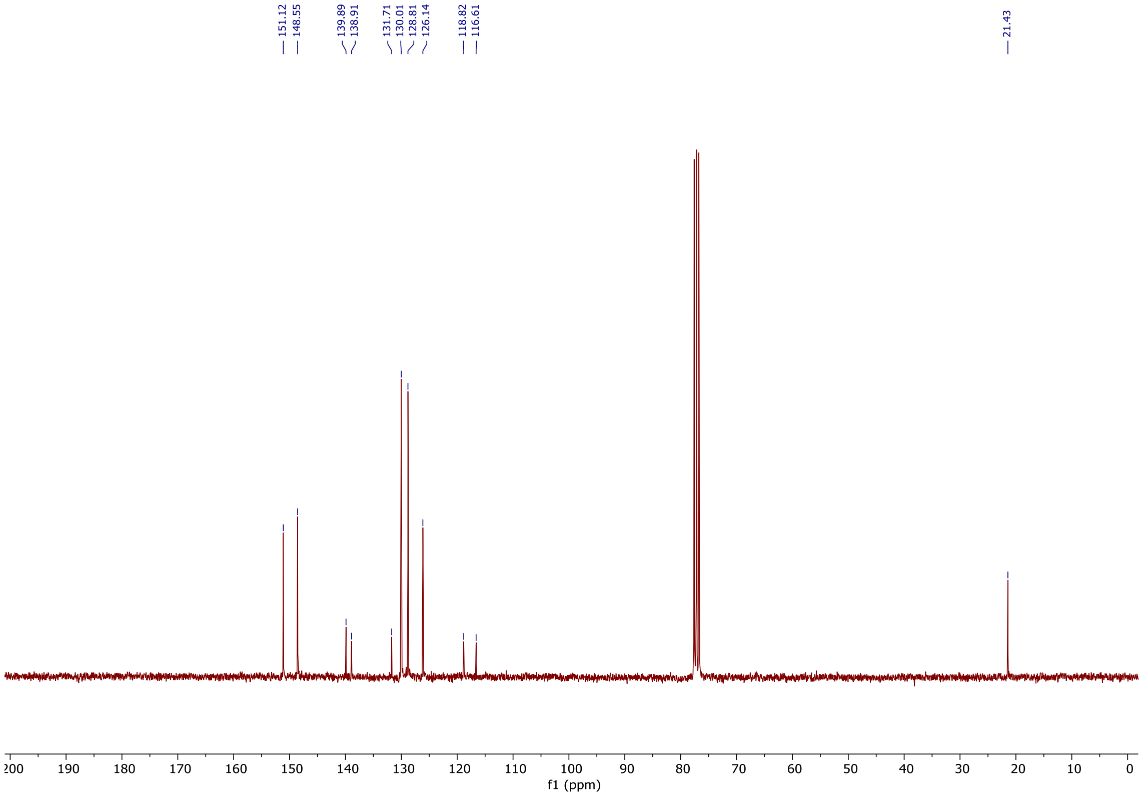
151.12
148.55
139.89
138.91
131.71
130.01
128.81
126.14
118.82
116.61
21.43
200 190 180 170 160 150 140 130 120 110 100 90 80 70 60 50 40 30 20 10 0
f1 (ppm)

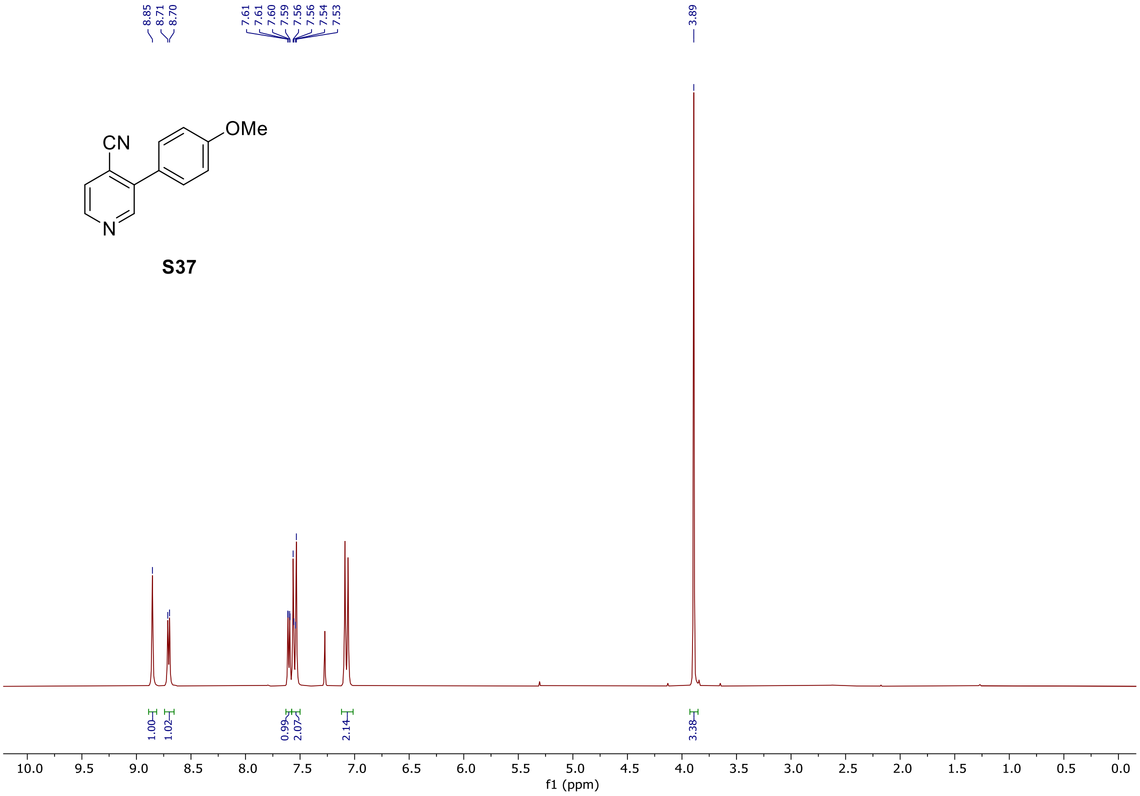

CN
OMe
N
S37
8.85
8.71
8.70
7.61
7.61
7.60
7.59
7.56
7.56
7.54
7.53
3.89
1.00
1.02
0.99
2.07
2.14
3.38
10.0 9.5 9.0 8.5 8.0 7.5 7.0 6.5 6.0 5.5 5.0 4.5 4.0 3.5 3.0 2.5 2.0 1.5 1.0 0.5 0.0
f1 (ppm)

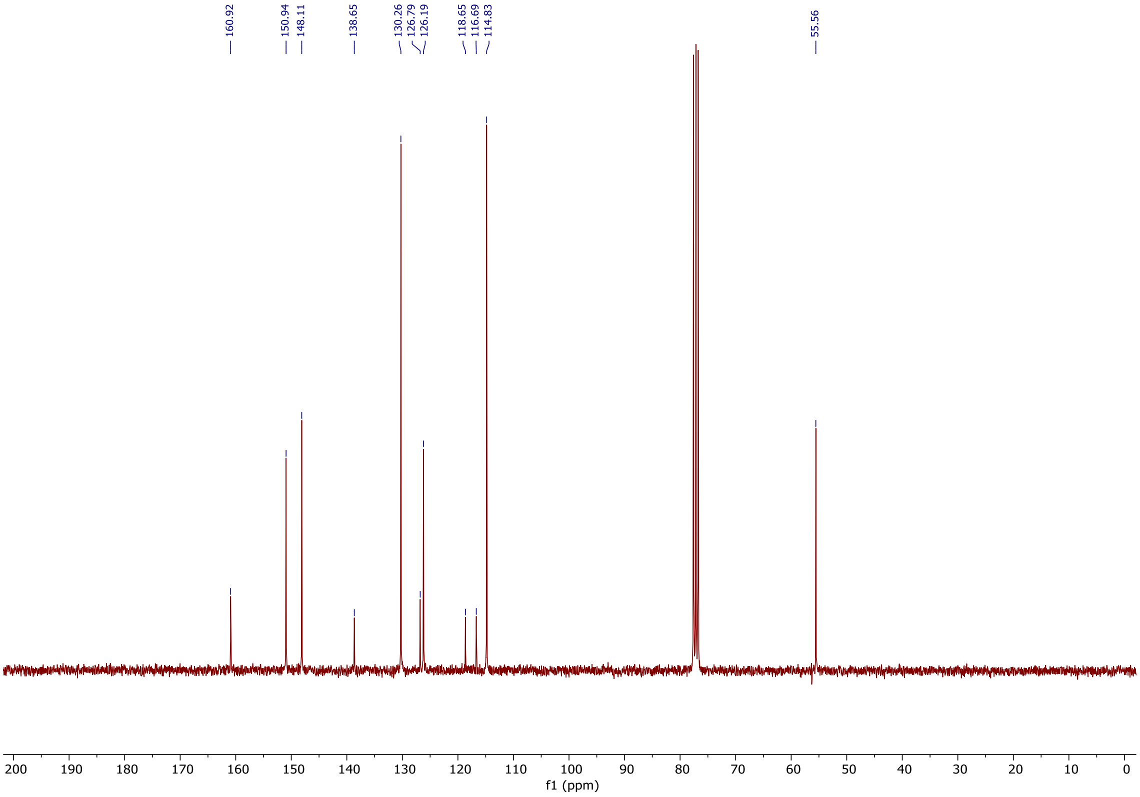

160.92
150.94
148.11
138.65
130.26
126.79
126.19
118.65
116.69
114.83
55.56
200 190 180 170 160 150 140 130 120 110 100 90 80 70 60 50 40 30 20 10 0
f1 (ppm)

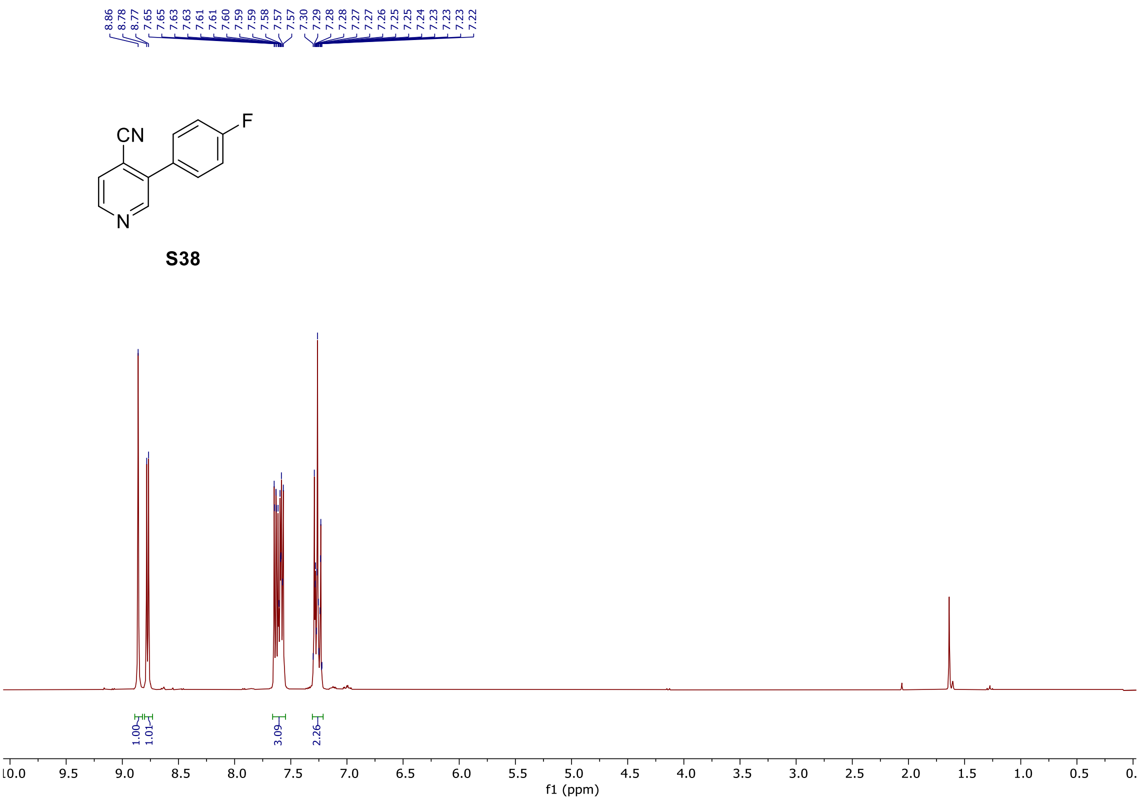
8.86
8.78
8.77
7.65
7.65
7.63
7.63
7.61
7.61
7.60
7.59
7.59
7.58
7.57
7.57
7.30
7.29
7.28
7.28
7.27
7.27
7.26
7.25
7.25
7.24
7.23
7.23
7.23
7.22
CN
F
N
1.00
1.01
3.09
2.26
f1 (ppm)

S38

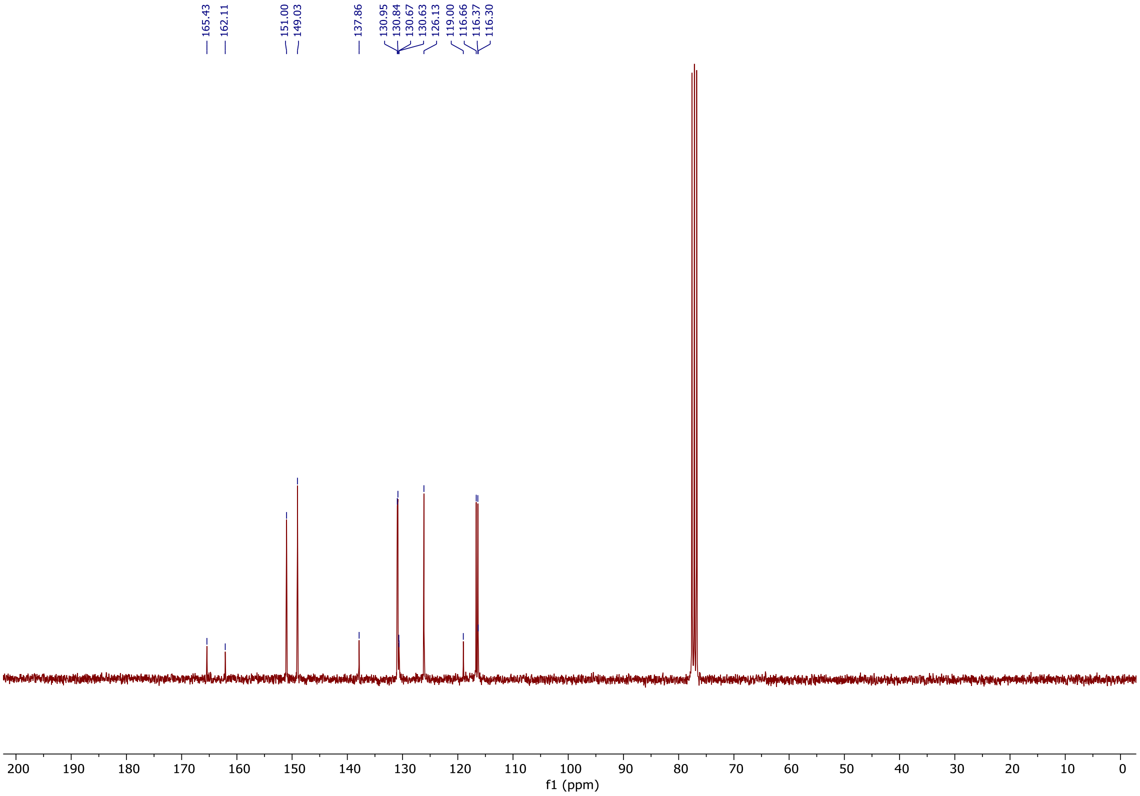
165.43
162.11
151.00
149.03
137.86
130.95
130.84
130.67
130.63
126.13
119.00
116.66
116.37
116.30
f1 (ppm)

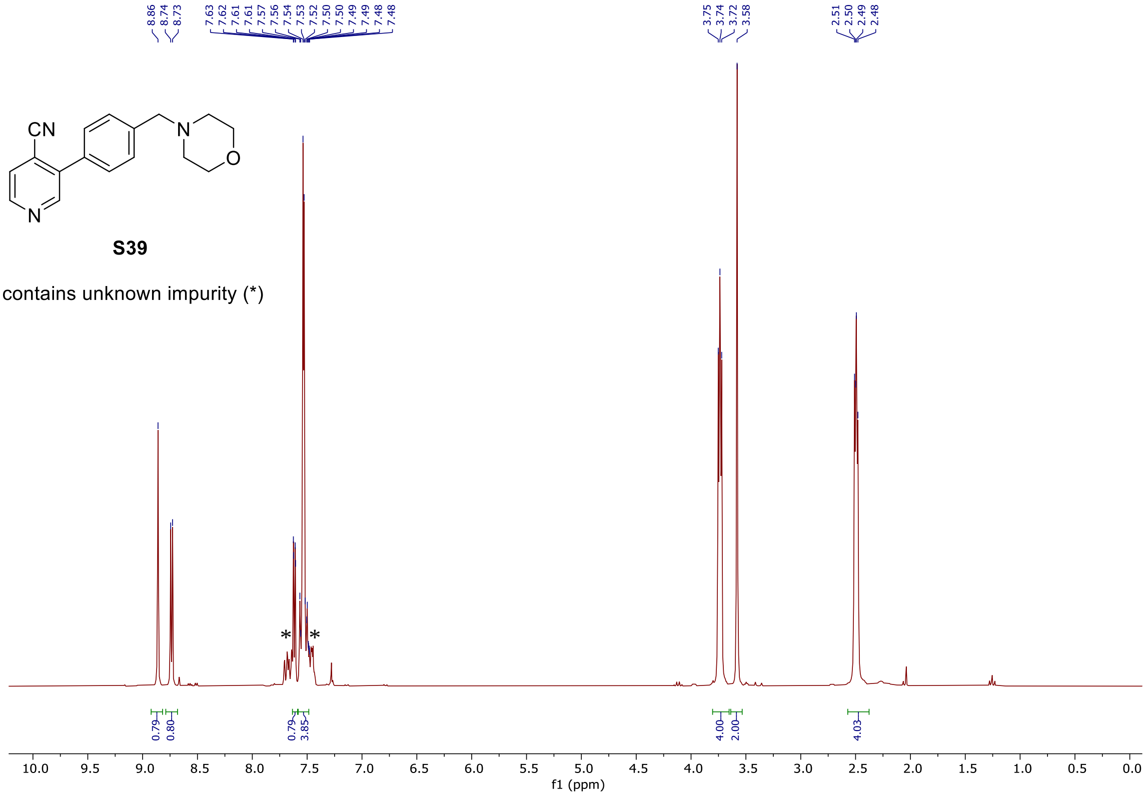

S39

contains unknown impurity (*)

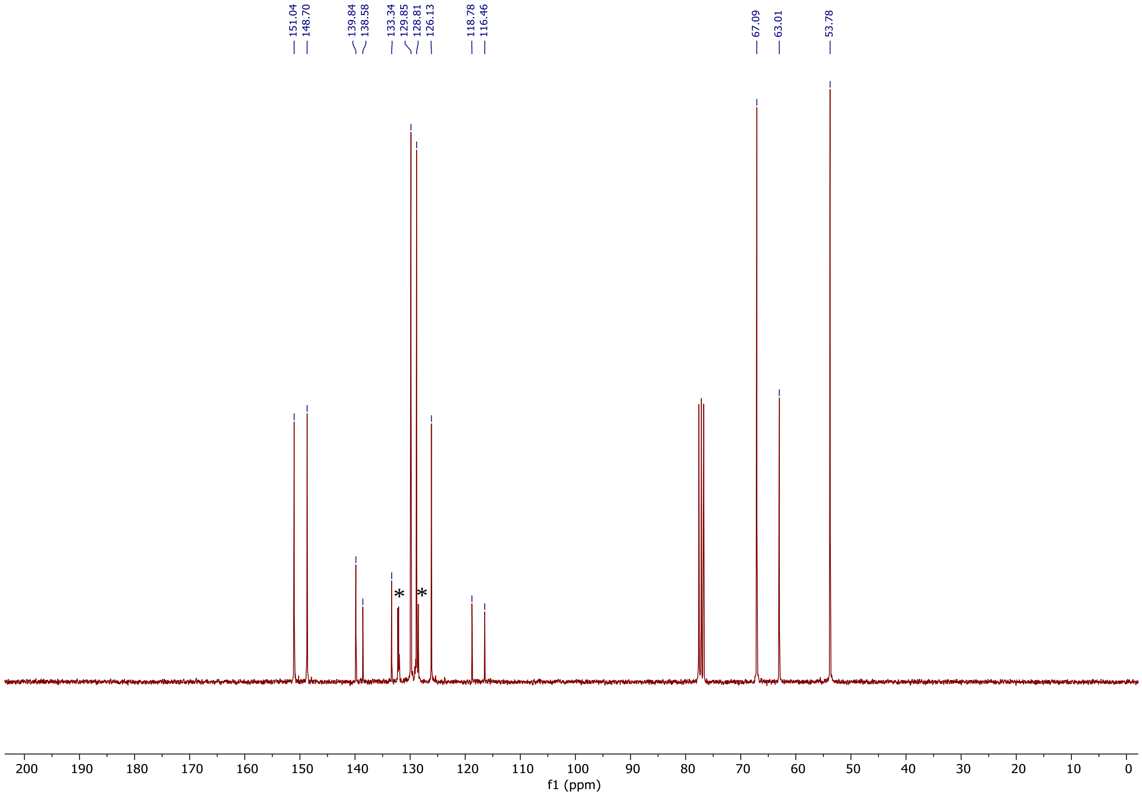

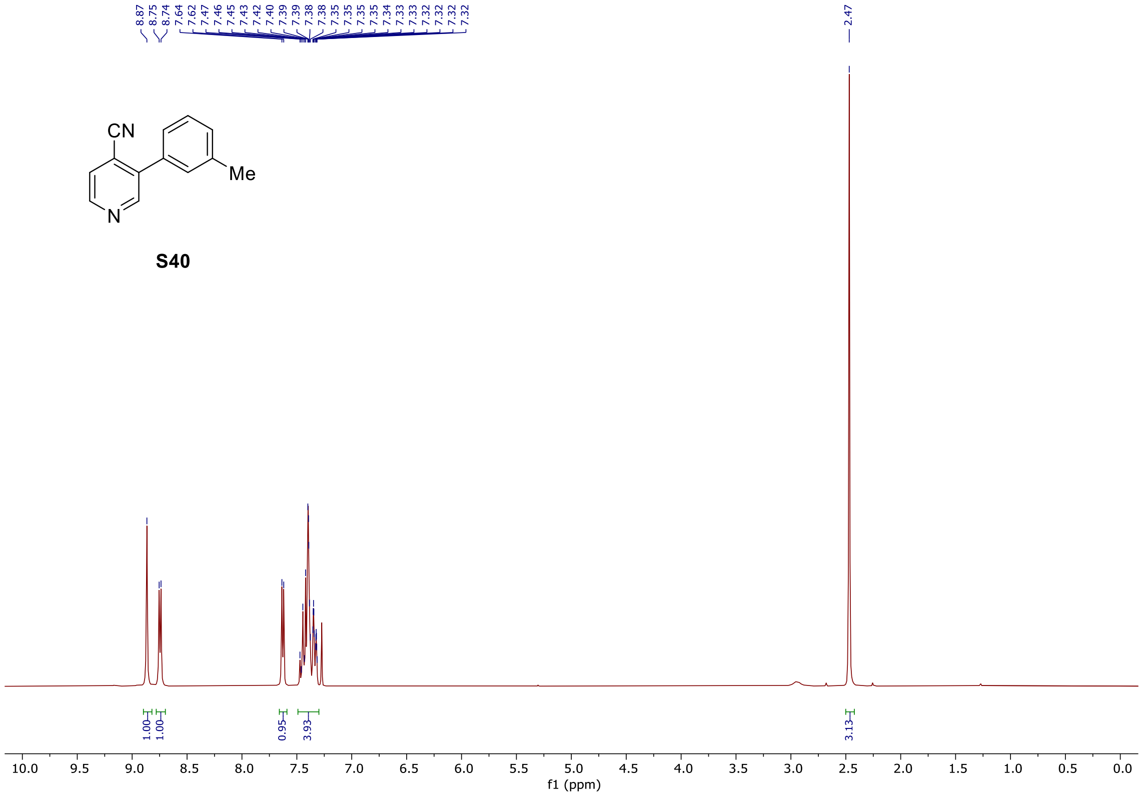
8.87
8.75
8.74
7.64
7.62
7.47
7.46
7.45
7.43
7.42
7.40
7.39
7.39
7.38
7.38
7.35
7.35
7.35
7.35
7.34
7.33
7.33
7.32
7.32
7.32
7.32
2.47
CN
Me
N
S40
1.00
1.00
0.95
3.93
3.13
10.0 9.5 9.0 8.5 8.0 7.5 7.0 6.5 6.0 5.5 5.0 4.5 4.0 3.5 3.0 2.5 2.0 1.5 1.0 0.5 0.0
f1 (ppm)

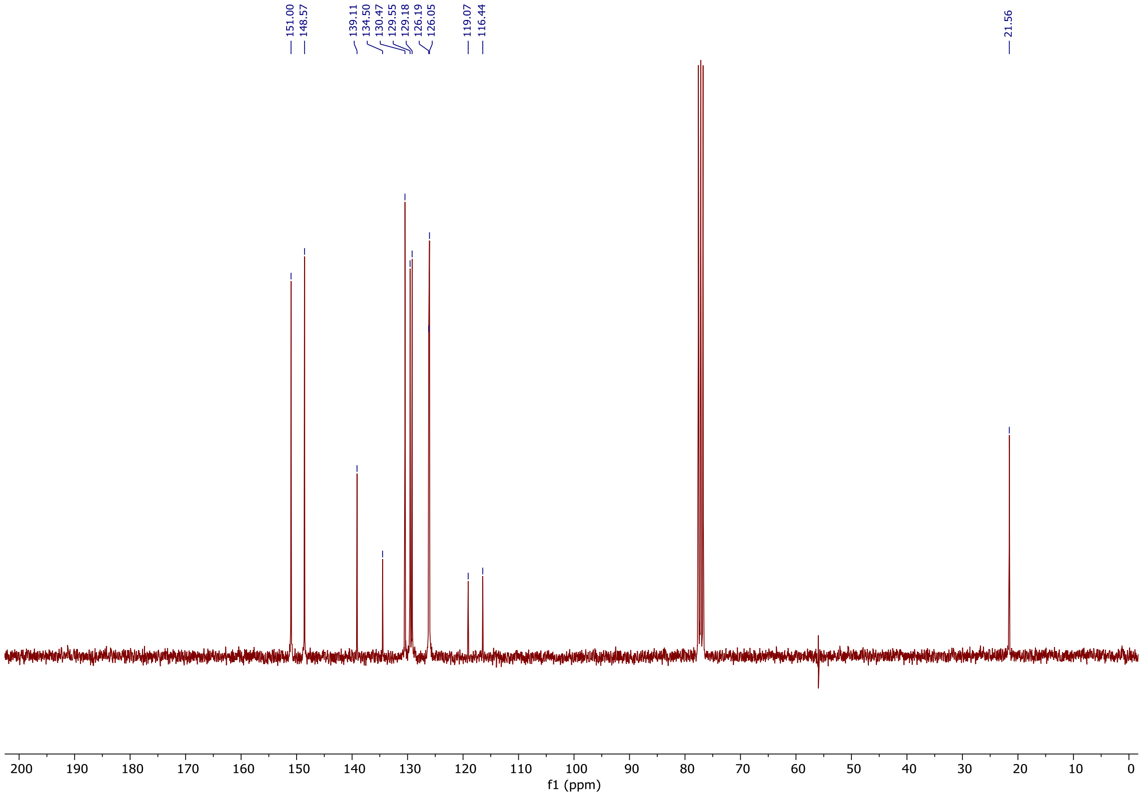
151.00
148.57
139.11
134.50
130.47
129.55
129.18
126.19
126.05
119.07
116.44
21.56
200 190 180 170 160 150 140 130 120 110 100 90 80 70 60 50 40 30 20 10 0
f1 (ppm)

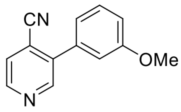
CN
OMe
N

S41

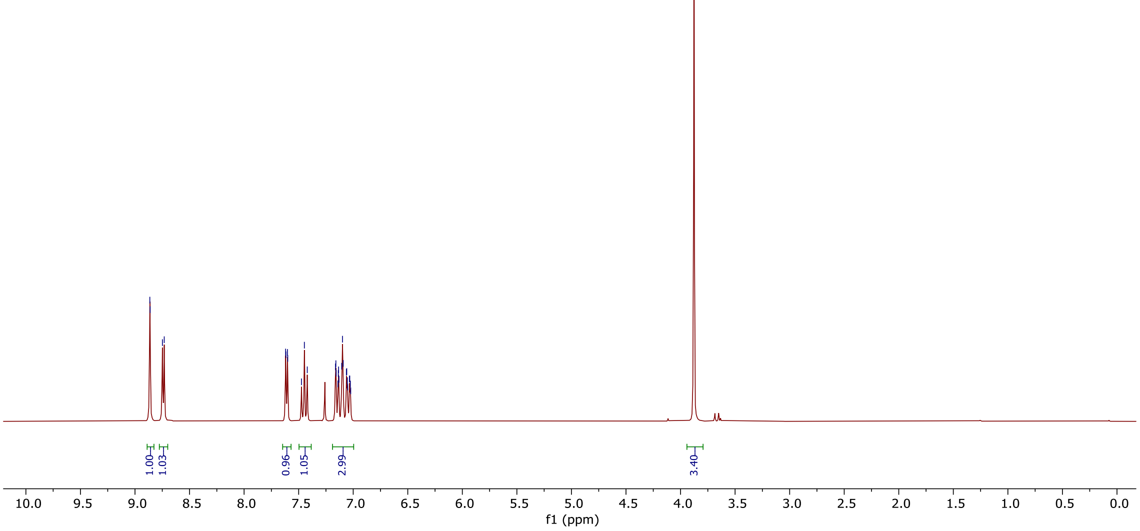
8.86
8.86
8.75
8.73
7.62
7.62
7.60
7.60
7.47
7.45
7.42
7.16
7.16
7.16
7.14
7.14
7.13
7.13
7.11
7.10
7.09
7.06
7.06
7.05
7.05
7.03
7.03
7.02
3.88
1.00
1.03
0.96
1.05
2.99
3.40
10.0 9.5 9.0 8.5 8.0 7.5 7.0 6.5 6.0 5.5 5.0 4.5 4.0 3.5 3.0 2.5 2.0 1.5 1.0 0.5 0.0
f1 (ppm)

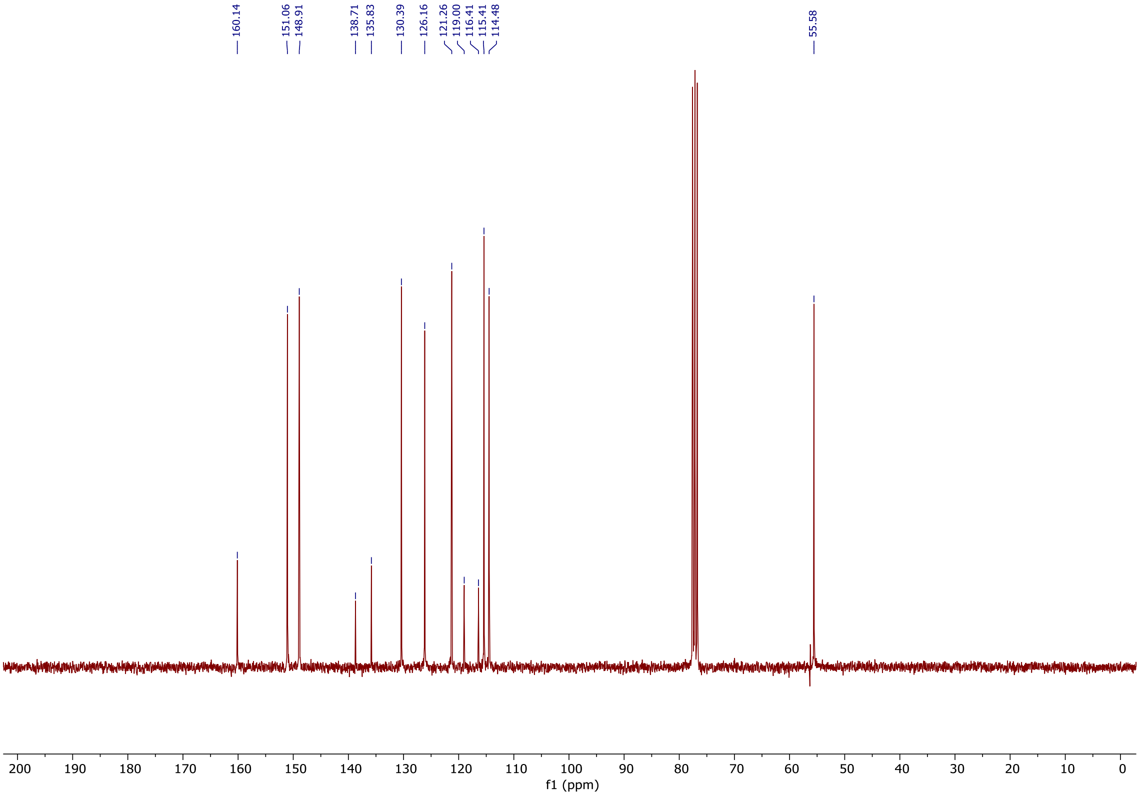
160.14
151.06
148.91
138.71
135.83
130.39
126.16
121.26
119.00
116.41
115.41
114.48
55.58
200 190 180 170 160 150 140 130 120 110 100 90 80 70 60 50 40 30 20 10 0
f1 (ppm)

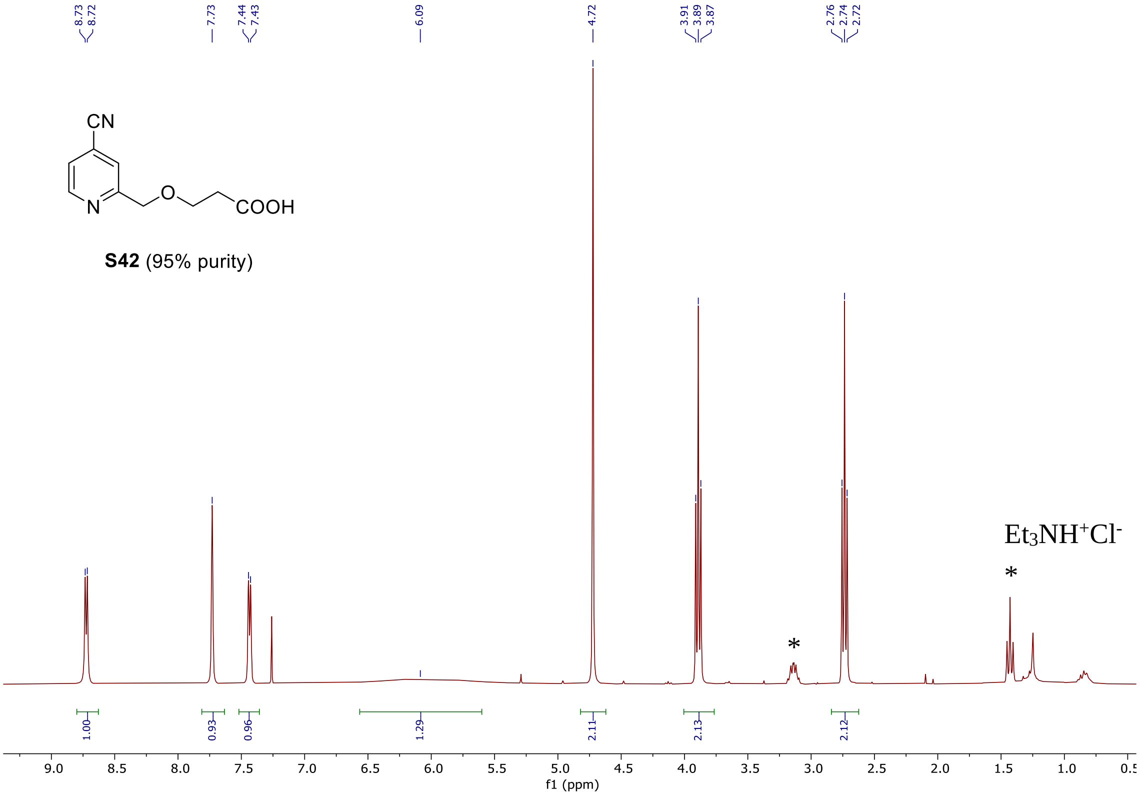

CN
N
O
COOH
S42 (95% purity)
Et3NH+Cl-
*
8.73
8.72
7.73
7.44
7.43
6.09
4.72
3.91
3.89
3.87
2.76
2.74
2.72
1.00
0.93
0.96
1.29
2.11
2.13
2.12
f1 (ppm)

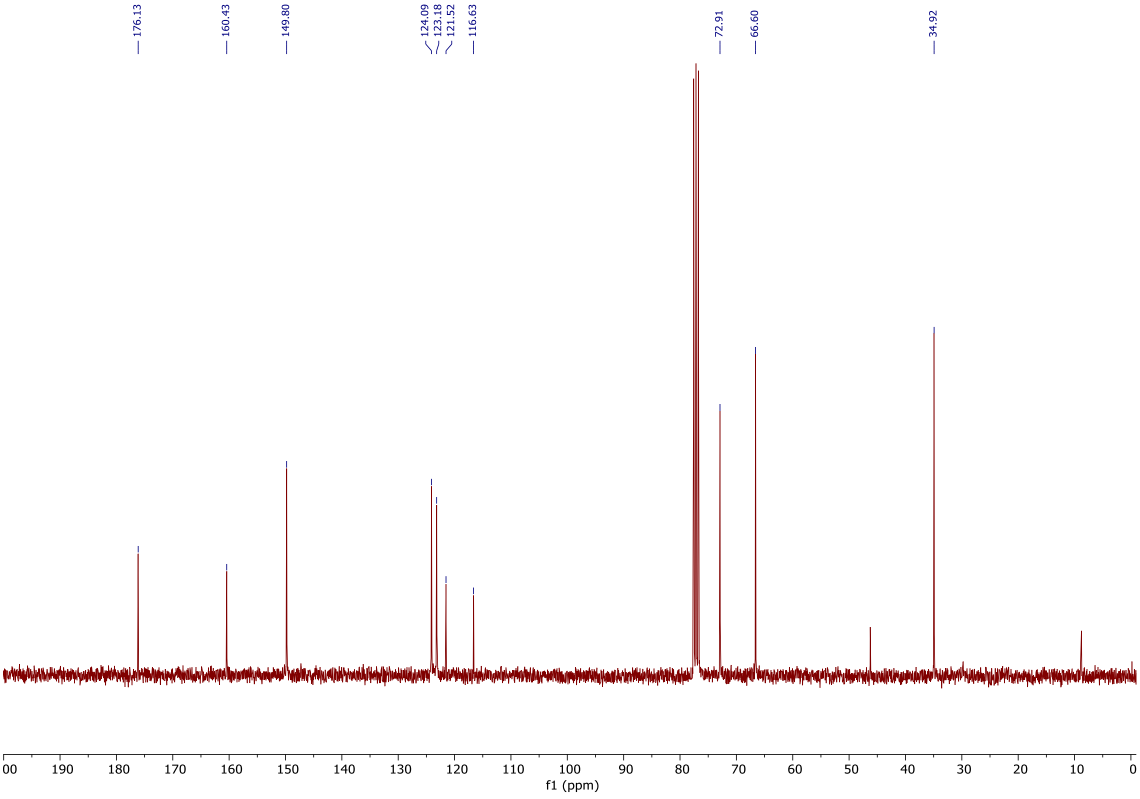

176.13
160.43
149.80
124.09
123.18
121.52
116.63
72.91
66.60
34.92
f1 (ppm)

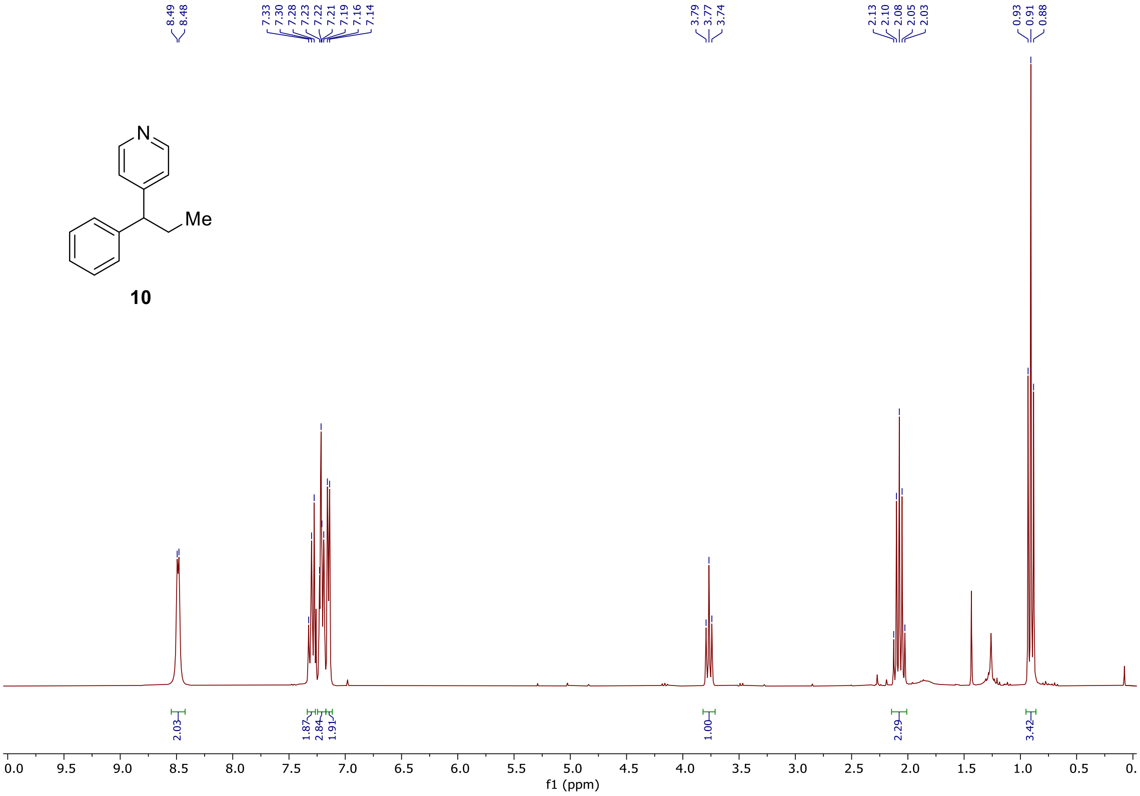
10
8.49
8.48
7.33
7.30
7.28
7.23
7.22
7.21
7.19
7.16
7.14
3.79
3.77
3.74
2.13
2.10
2.08
2.05
2.03
0.93
0.91
0.88
2.03
1.87
2.84
1.91
1.00
2.29
3.42
f1 (ppm)

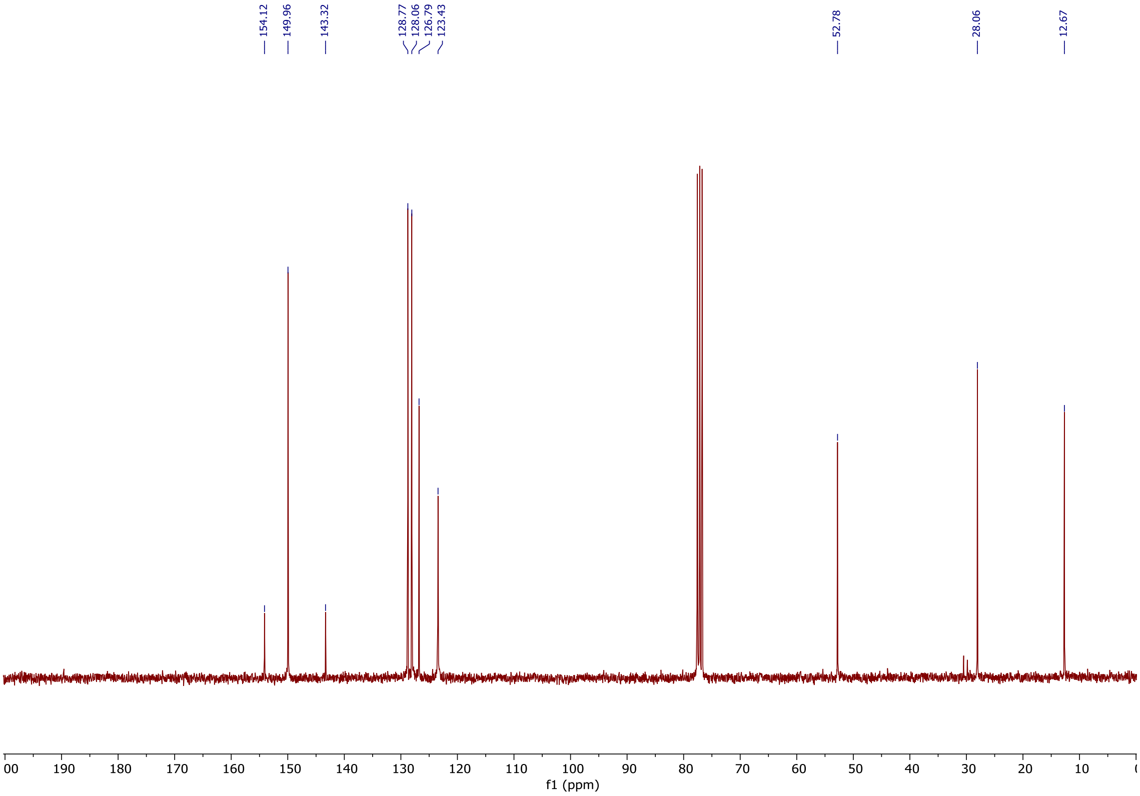
154.12
149.96
143.32
128.77
128.06
126.79
123.43
52.78
28.06
12.67
f1 (ppm)

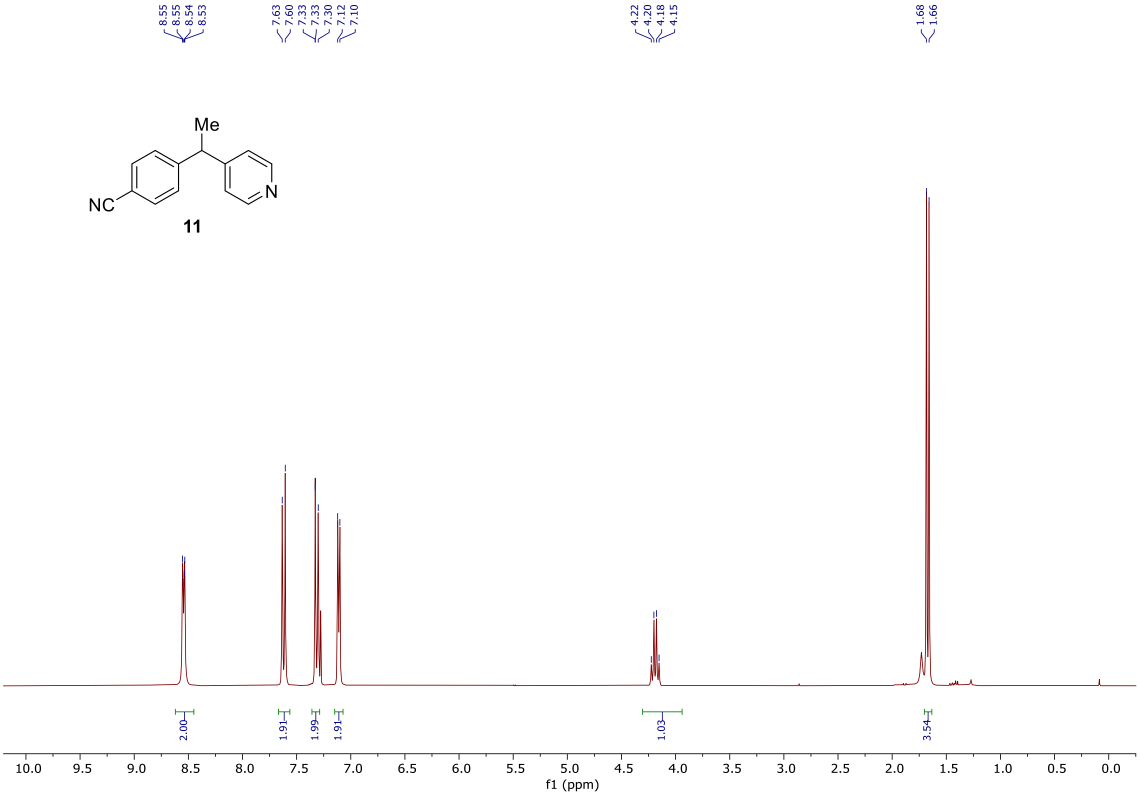
8.55
8.55
8.54
8.53
7.63
7.60
7.33
7.33
7.30
7.12
7.10
4.22
4.20
4.18
4.15
1.68
1.66
Me
NC
N
11
2.00
1.91
1.99
1.91
1.03
3.54
10.0 9.5 9.0 8.5 8.0 7.5 7.0 6.5 6.0 5.5 5.0 4.5 4.0 3.5 3.0 2.5 2.0 1.5 1.0 0.5 0.0
f1 (ppm)

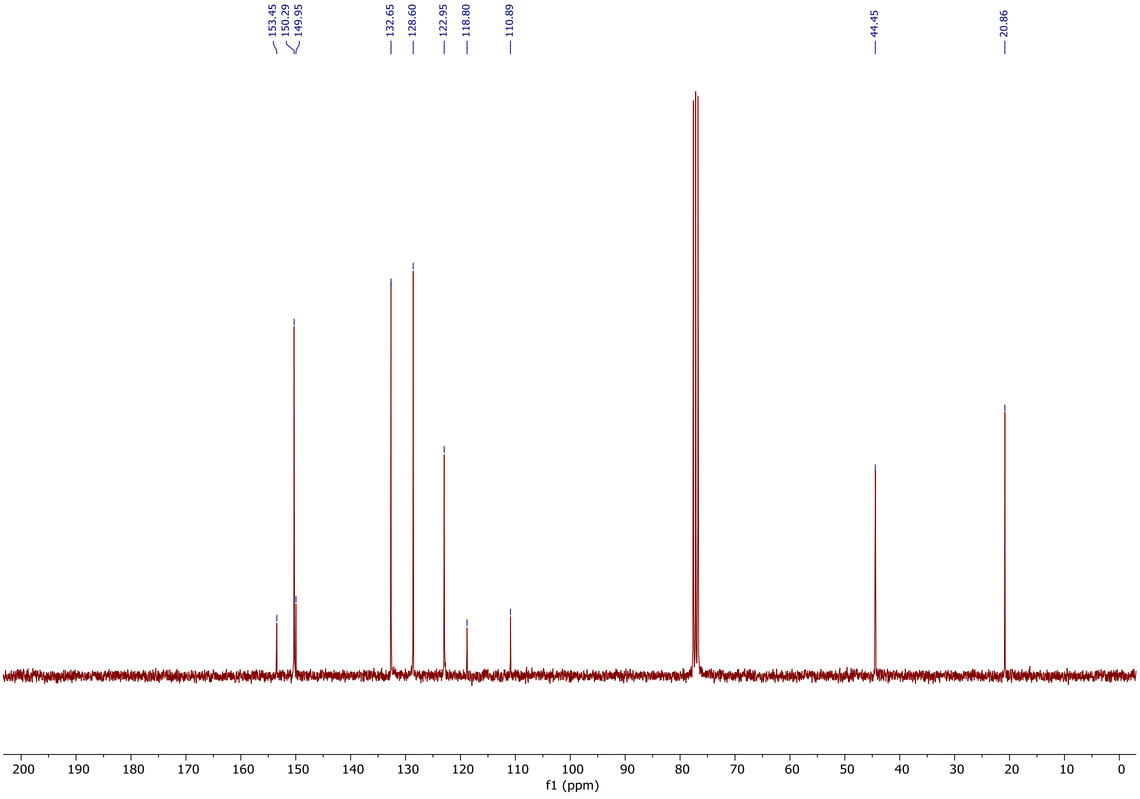
153.45
150.29
149.95
132.65
128.60
122.95
118.80
110.89
44.45
20.86
200 190 180 170 160 150 140 130 120 110 100 90 80 70 60 50 40 30 20 10 0
f1 (ppm)

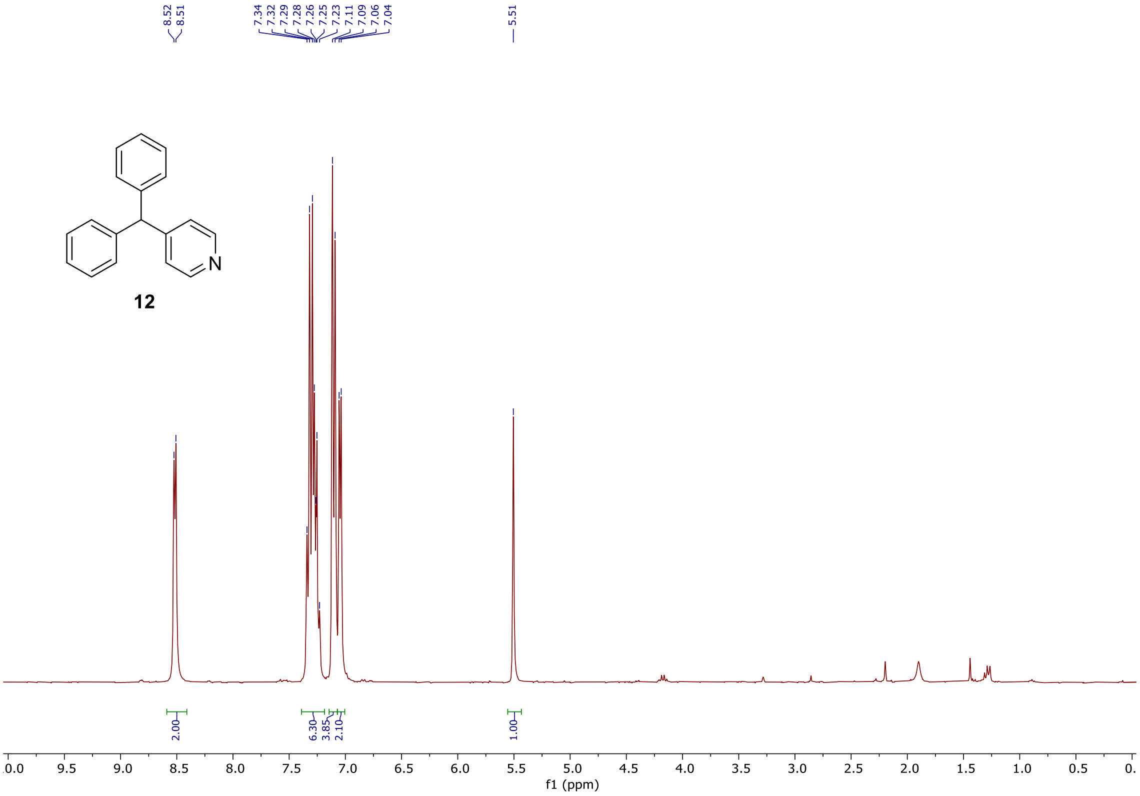
8.52
8.51
7.34
7.32
7.29
7.28
7.26
7.25
7.23
7.11
7.09
7.06
7.04
5.51
N
12
2.00
6.30
3.85
2.10
1.00
f1 (ppm)

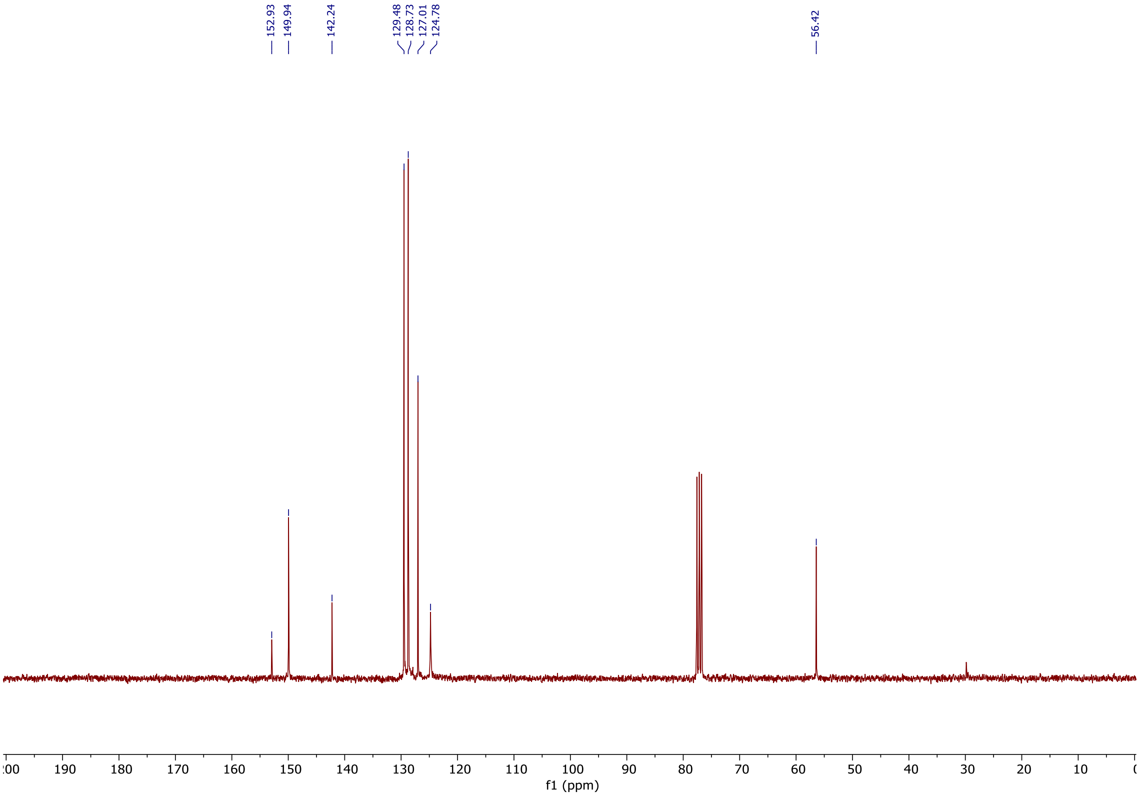
152.93
149.94
142.24
129.48
128.73
127.01
124.78
56.42
f1 (ppm)

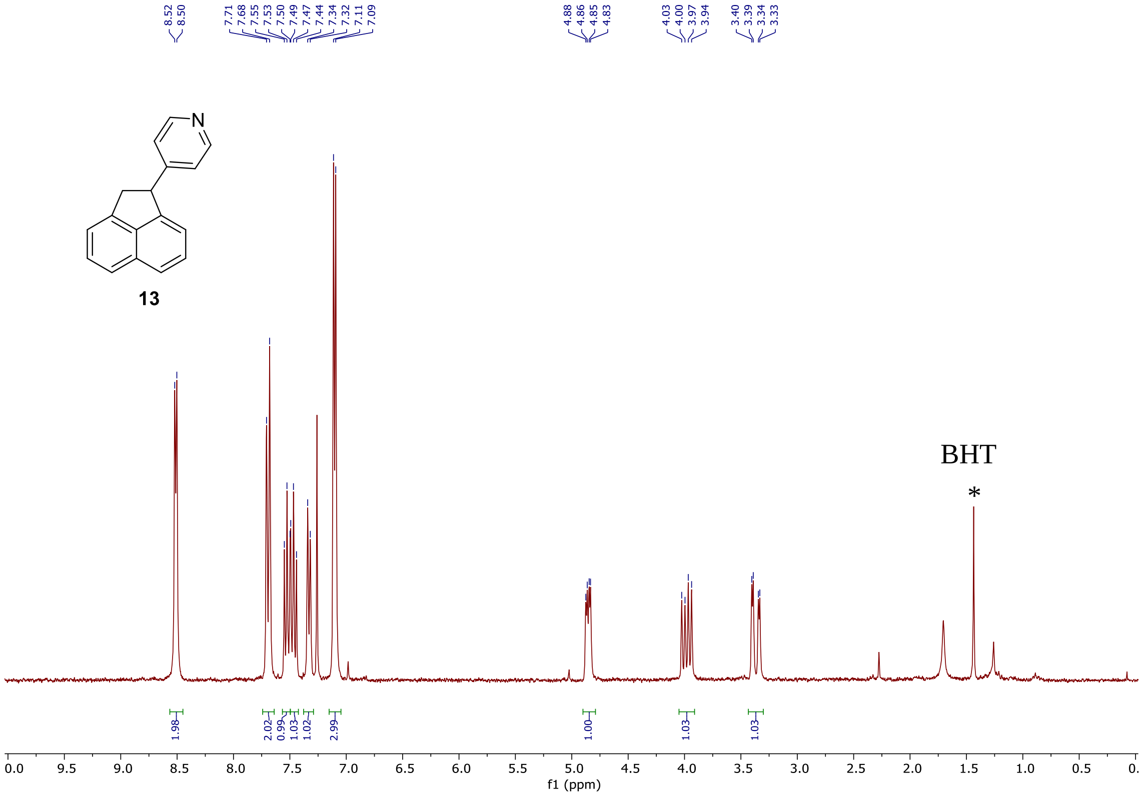
8.52
8.50
7.71
7.68
7.55
7.53
7.50
7.49
7.47
7.44
7.34
7.32
7.11
7.09
4.88
4.86
4.85
4.83
4.03
4.00
3.97
3.94
3.40
3.39
3.34
3.33
N
13
BHT
*
1.98
2.02
0.99
1.03
1.02
2.99
1.00
1.03
1.03
f1 (ppm)

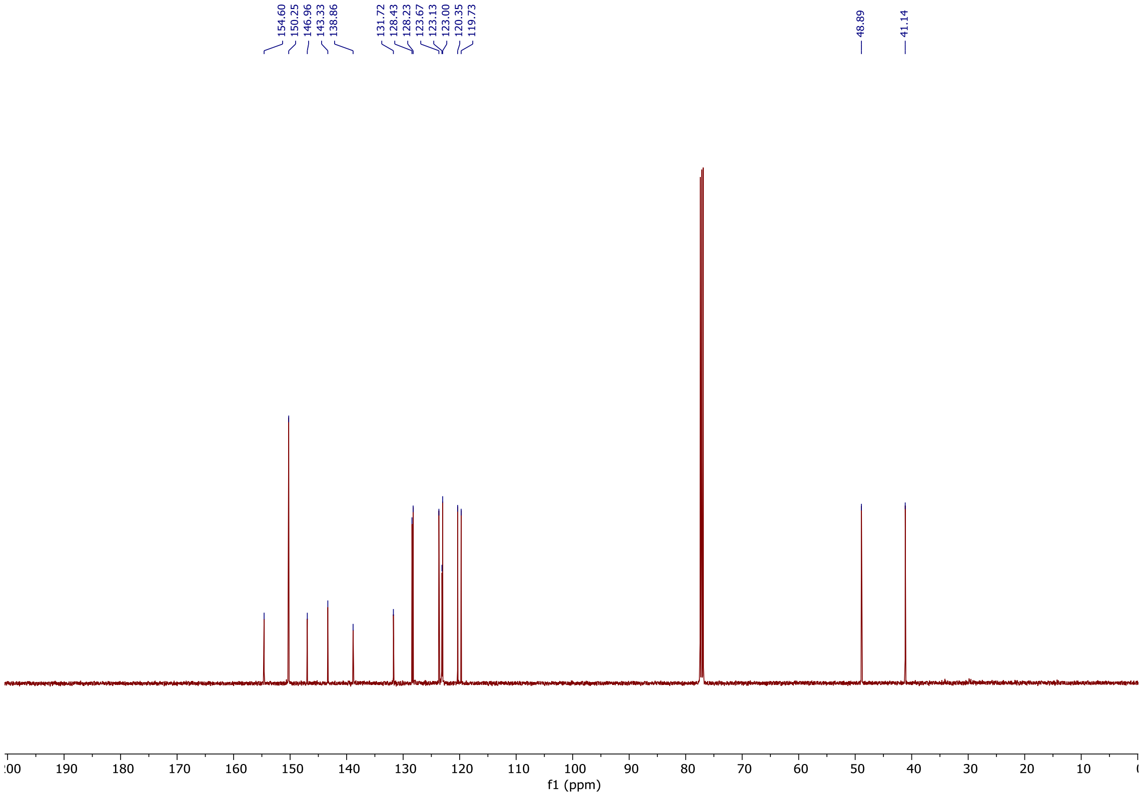
154.60
150.25
146.96
143.33
138.86
131.72
128.43
128.23
123.67
123.13
123.00
120.35
119.73
48.89
41.14
f1 (ppm)

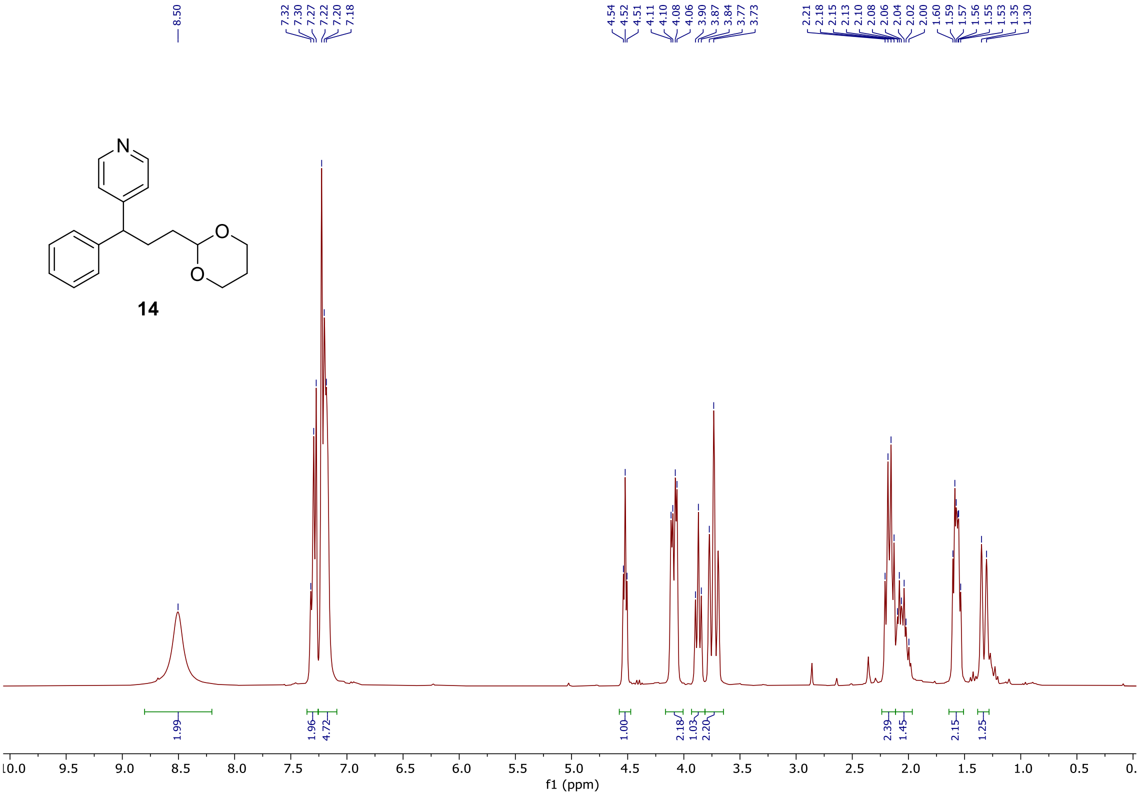

8.50
7.32 7.30 7.27 7.22 7.20 7.18
4.54 4.52 4.51 4.11 4.10 4.08 4.06 3.90 3.87 3.84 3.77 3.73
2.21 2.18 2.15 2.13 2.10 2.08 2.06 2.04 2.02 2.00 1.60 1.59 1.57 1.56 1.55 1.53 1.35 1.30
N
O
O
14
1.99 1.96 4.72 1.00 2.18 1.03 2.20 2.39 1.45 2.15 1.25
f1 (ppm)

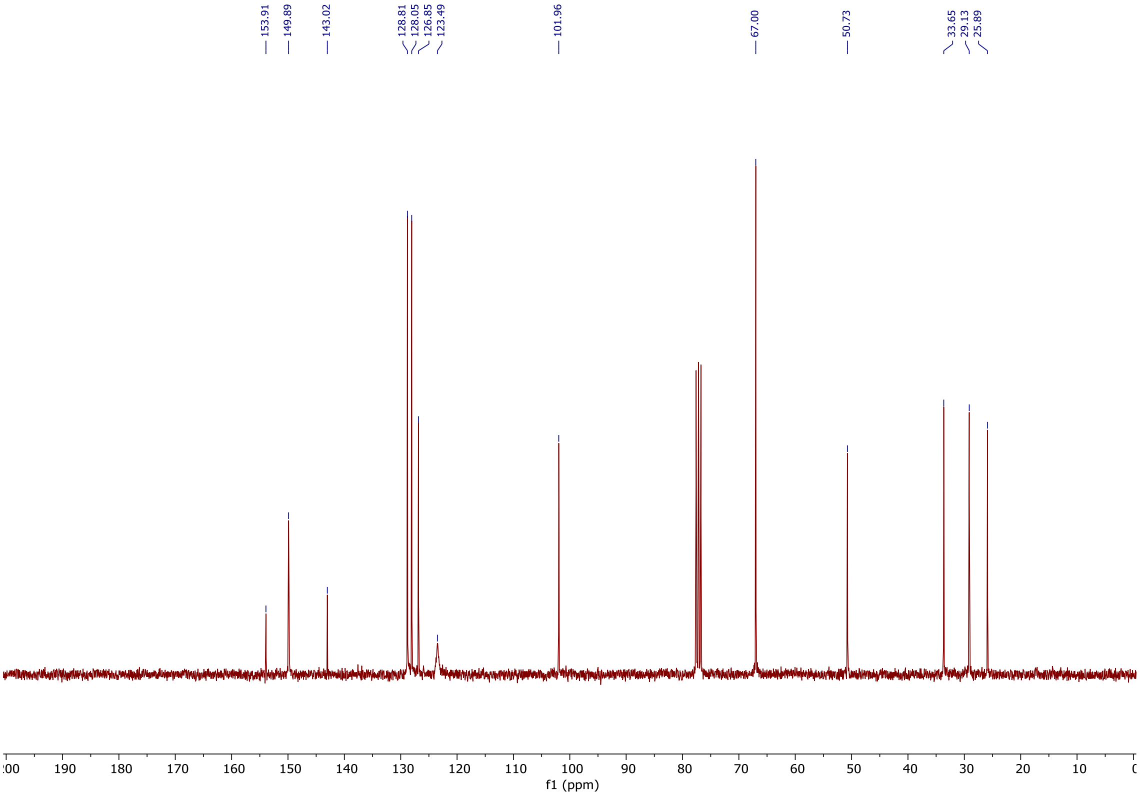

153.91 149.89 143.02 128.81 128.05 126.85 123.49 101.96 67.00 50.73 33.65 29.13 25.89
f1 (ppm)

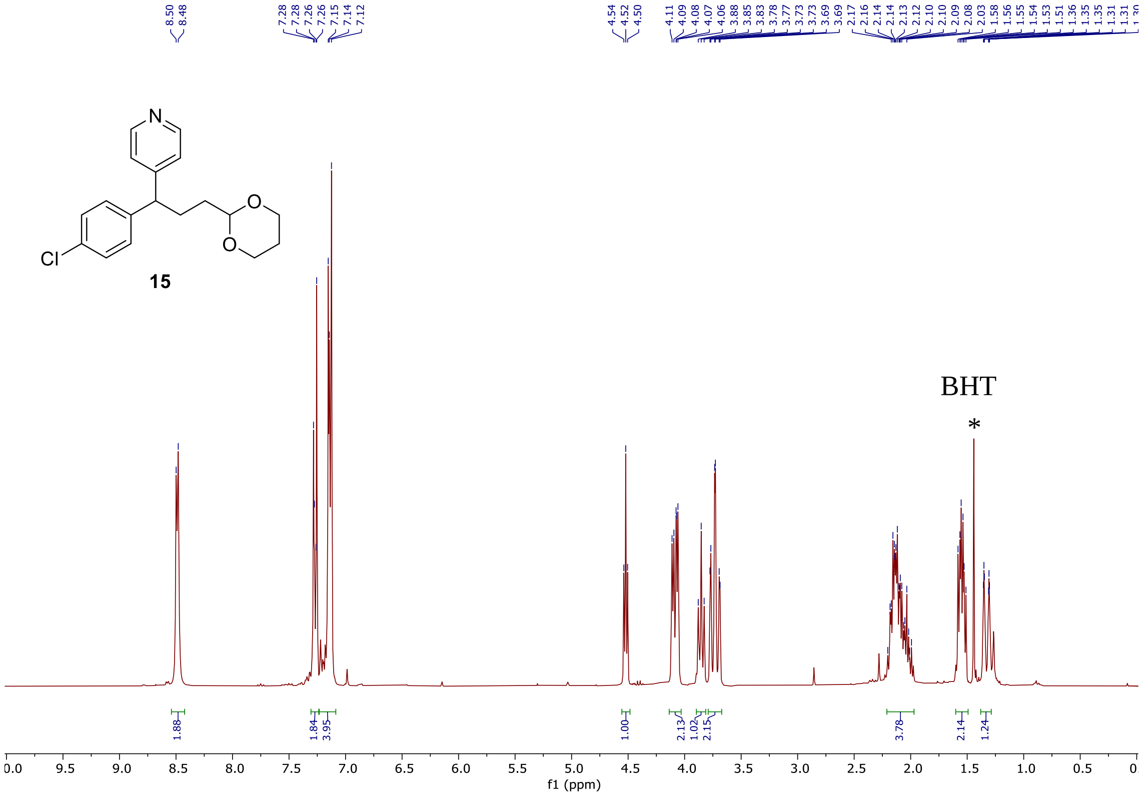
N
O
O
Cl
15
8.50
8.48
7.28
7.28
7.26
7.26
7.15
7.14
7.12
4.54
4.52
4.50
BHT
*
1.88
1.84
3.95
1.00
2.13
1.02
2.15
3.78
2.14
1.24
f1 (ppm)

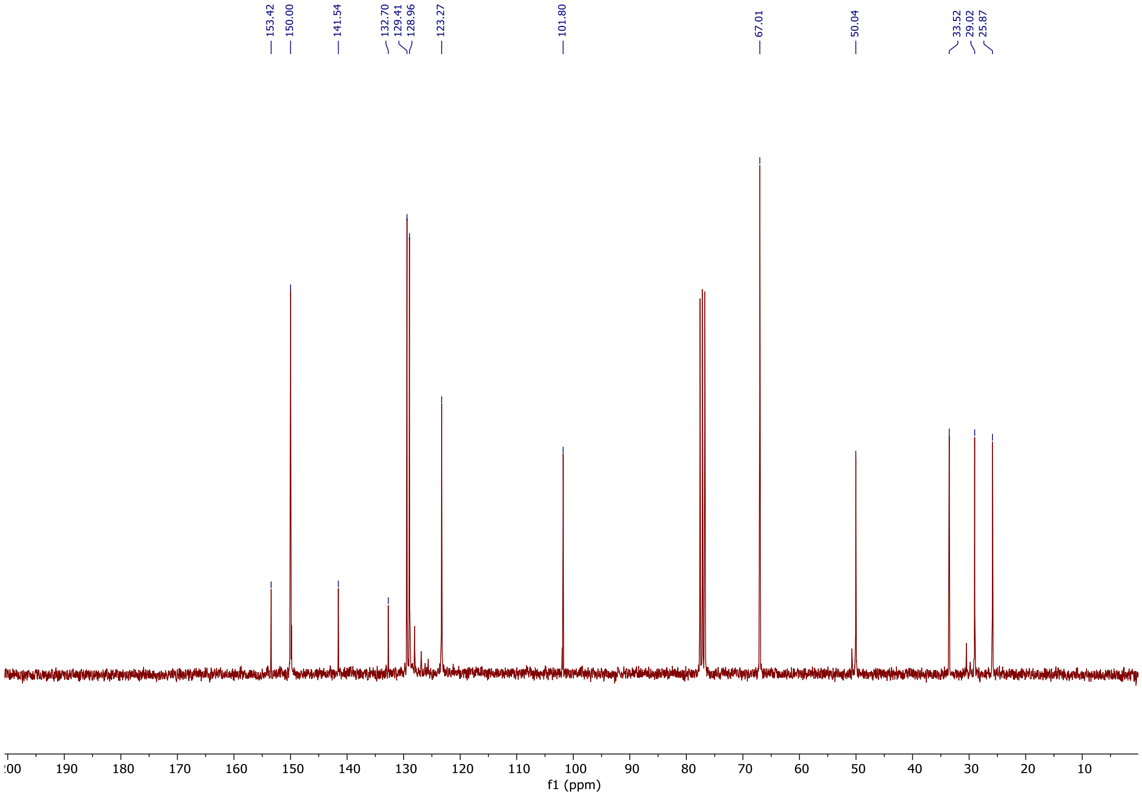
153.42
150.00
141.54
132.70
129.41
128.96
123.27
101.80
67.01
50.04
33.52
29.02
25.87
f1 (ppm)

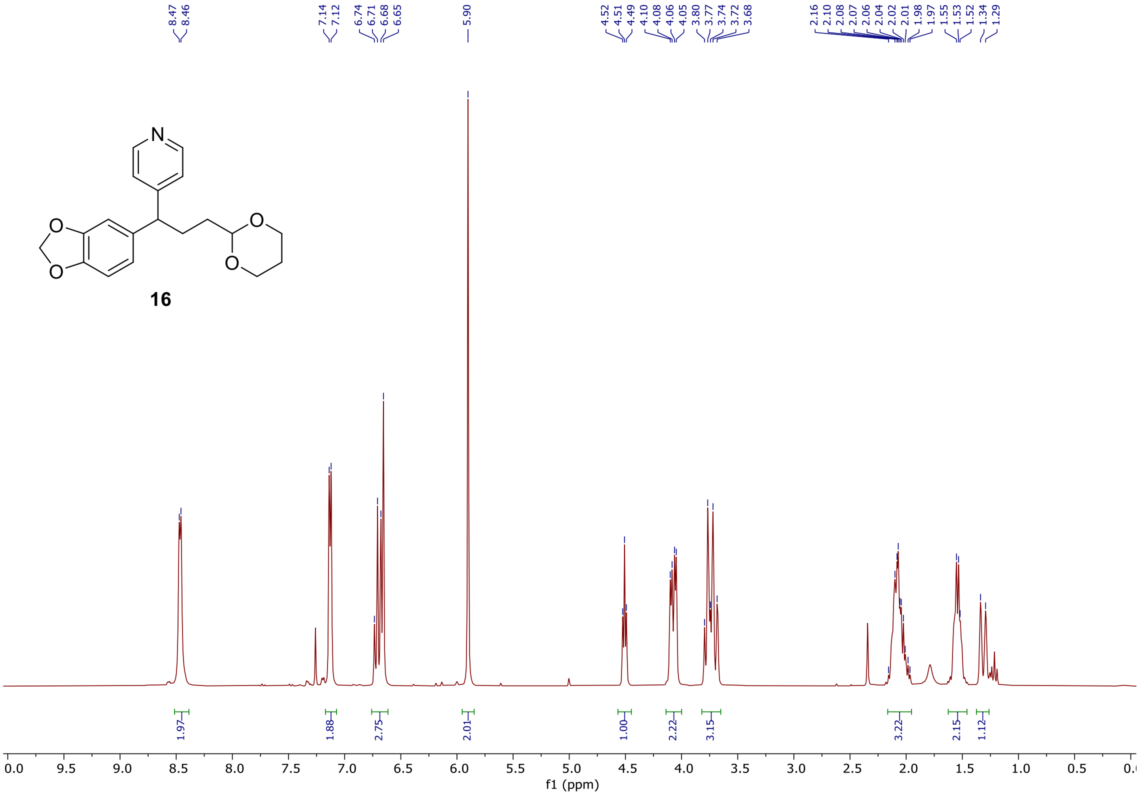
8.47
8.46
7.14
7.12
6.74
6.71
6.68
6.65
5.90
4.52
4.51
4.49
4.10
4.08
4.06
4.05
3.80
3.77
3.74
3.72
3.68
2.16
2.10
2.08
2.07
2.06
2.04
2.02
2.01
1.98
1.97
1.55
1.53
1.52
1.34
1.29
N
O
O
O
O
16
1.97
1.88
2.75
2.01
1.00
2.22
3.15
3.22
2.15
1.12
f1 (ppm)

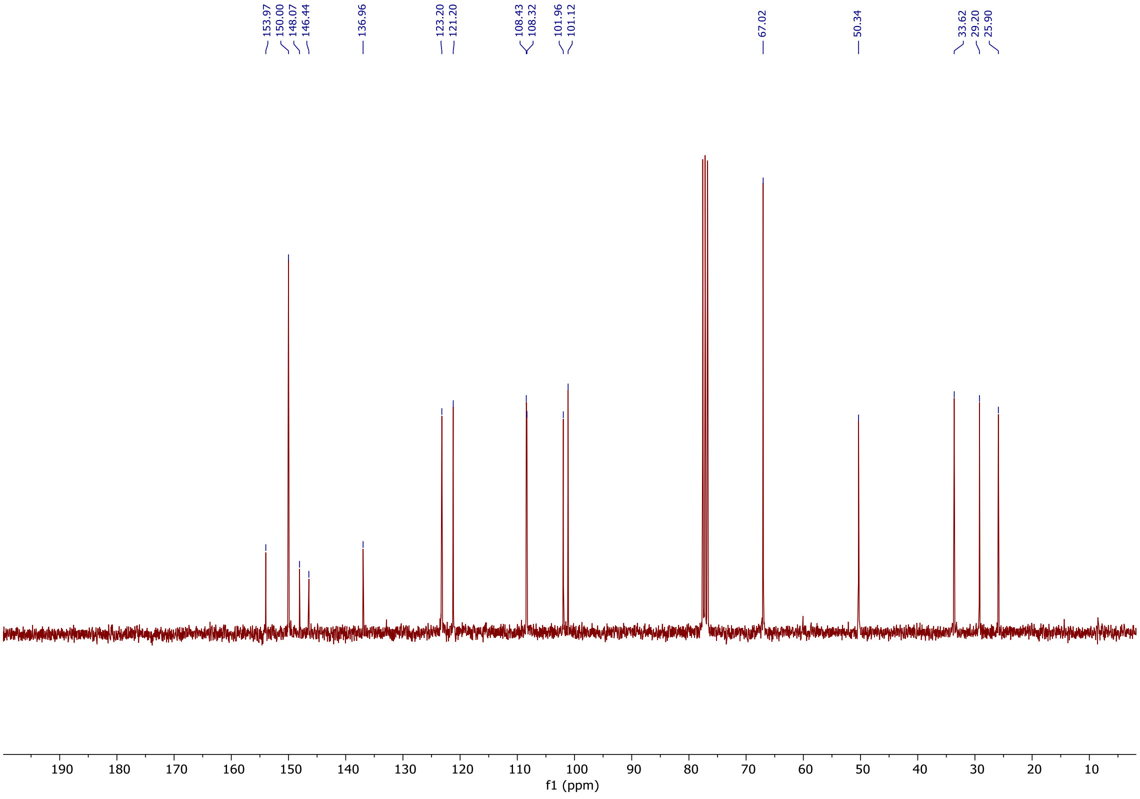
153.97
150.00
148.07
146.44
136.96
123.20
121.20
108.43
108.32
101.96
101.12
67.02
50.34
33.62
29.20
25.90
f1 (ppm)

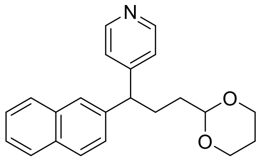

17

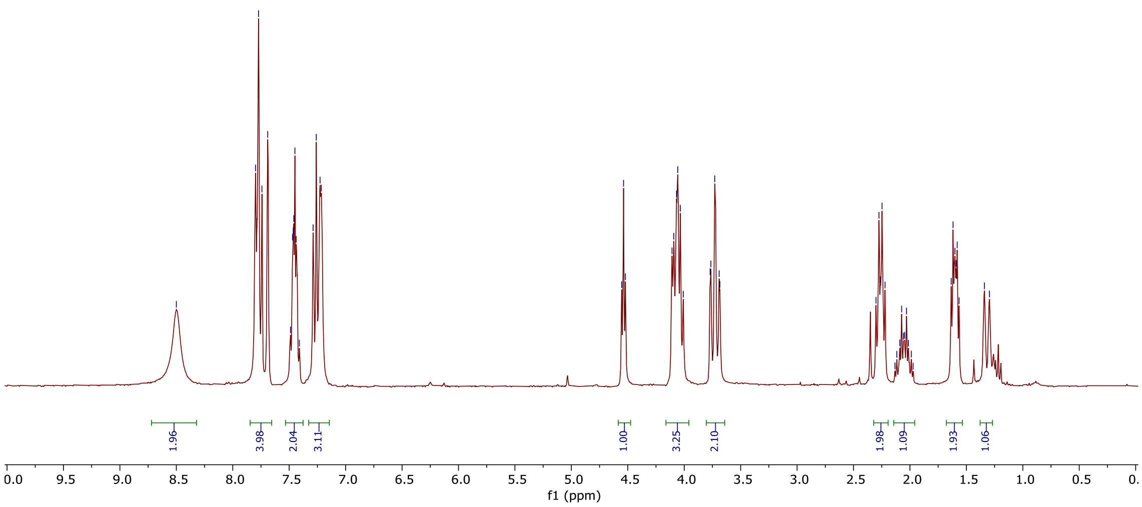
8.50
7.80 7.77 7.74 7.69 7.49 7.47 7.46 7.45 7.44 7.41 7.29 7.26 7.23 7.22
4.55 4.54 4.52 4.11 4.09 4.07 4.06 4.03 4.01 3.77 3.76 3.73 3.69 3.68 2.30 2.27 2.26 2.25 2.22 2.13 2.12 2.09 2.07 2.06 2.05 2.03 2.01 1.99 1.97 1.63 1.62 1.60 1.59 1.58 1.56 1.34 1.30
1.96
3.98
2.04
3.11
1.00
3.25
2.10
1.98
1.09
1.93
1.06
f1 (ppm)

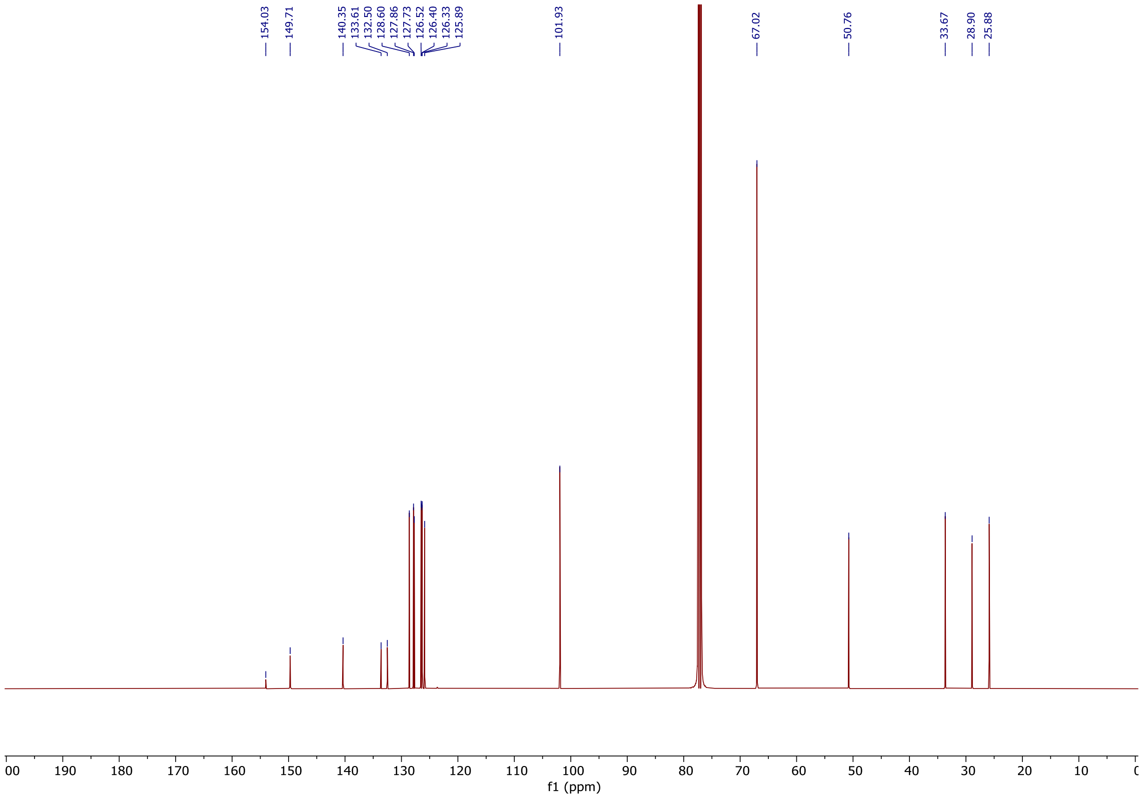
154.03
149.71
140.35 133.61 132.50 128.60 127.86 127.73 126.52 126.40 126.33 125.89
101.93
67.02
50.76
33.67
28.90
25.88
f1 (ppm)

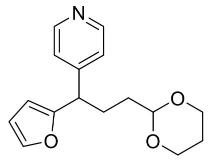
N
O
O
O

18

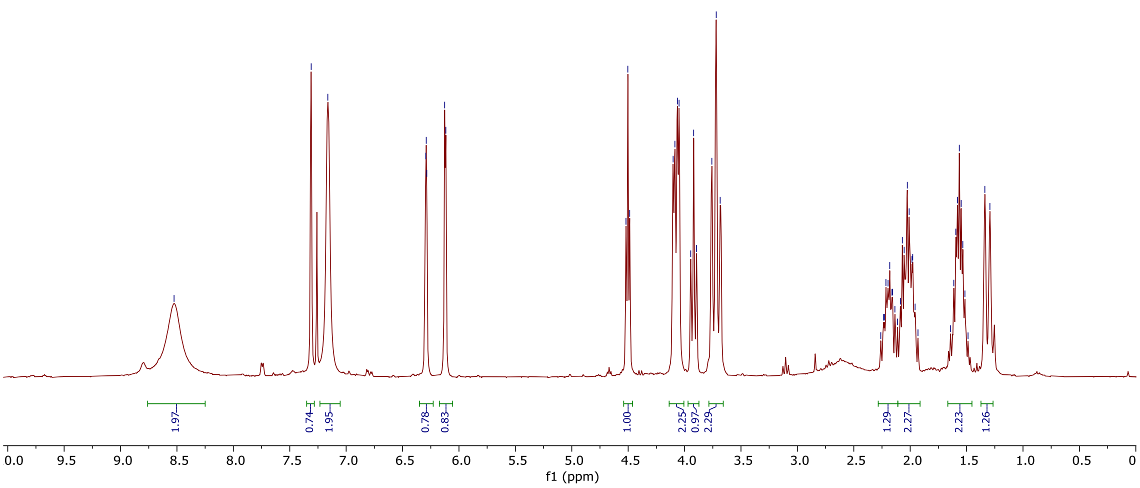
8.53
7.31
7.16
6.29
6.29
6.29
6.13
6.12
4.52
4.50
4.49
4.10
4.09
4.06
4.05
3.95
3.92
3.89
3.76
3.72
3.68
2.24
2.23
2.21
2.20
2.18
2.16
2.16
2.14
2.11
2.09
2.07
2.05
2.03
2.01
1.98
1.98
1.96
1.64
1.61
1.60
1.58
1.56
1.55
1.53
1.51
1.34
1.97
0.74
1.95
0.78
0.83
1.00
2.25
0.97
2.29
1.29
2.27
2.23
1.26
9.5
9.0
8.5
8.0
7.5
7.0
6.5
6.0
5.5
5.0
4.5
4.0
3.5
3.0
2.5
2.0
1.5
1.0
0.5
f1 (ppm)

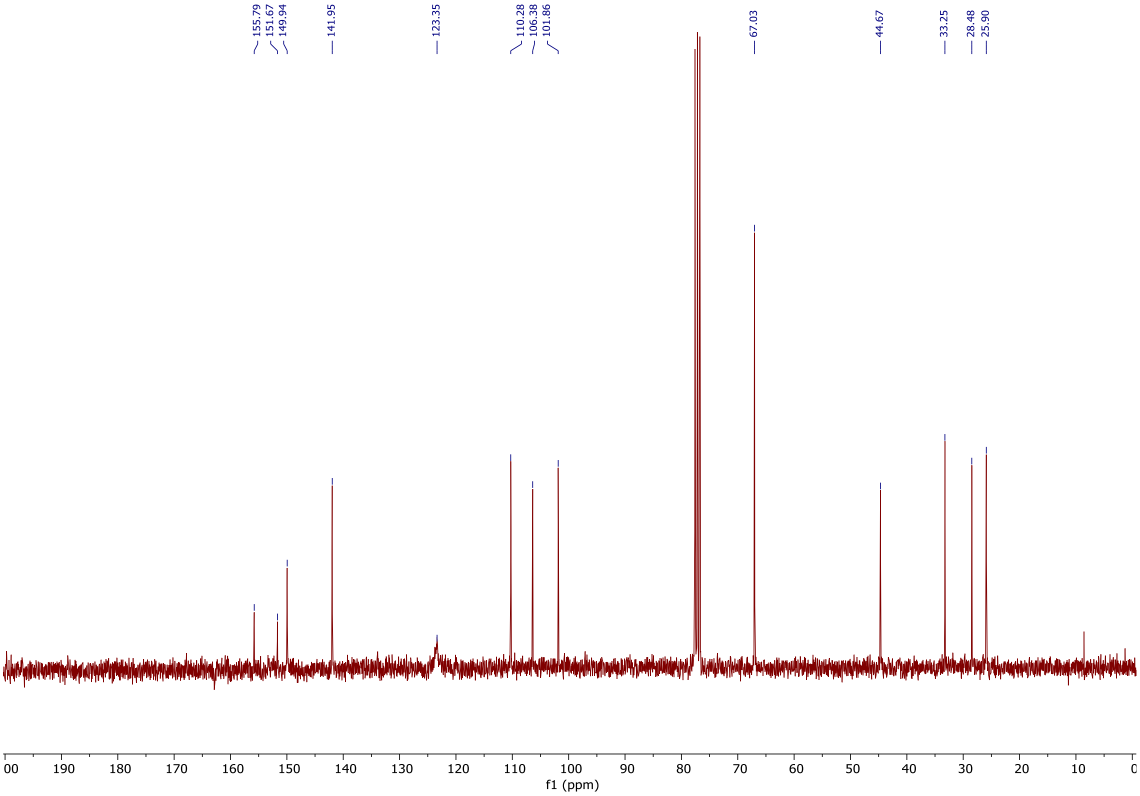
155.79
151.67
149.94
141.95
123.35
110.28
106.38
101.86
67.03
44.67
33.25
28.48
25.90
190
180
170
160
150
140
130
120
110
100
90
80
70
60
50
40
30
20
10
f1 (ppm)

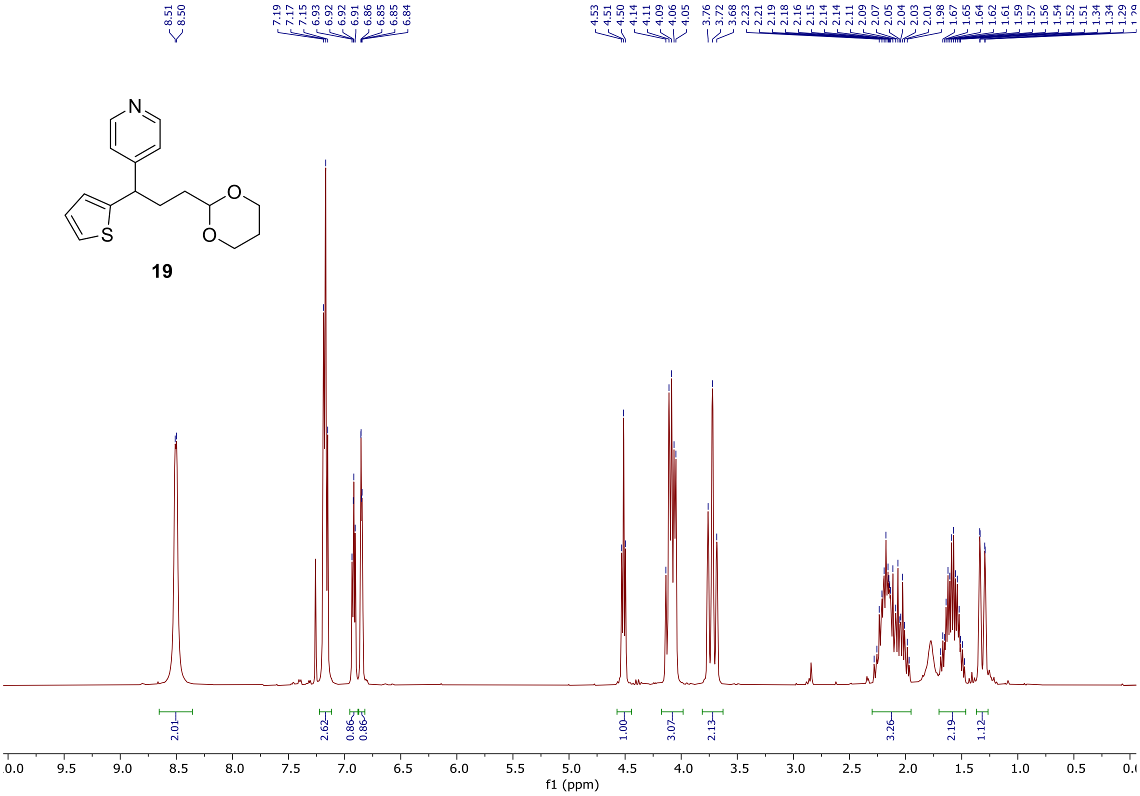
8.51
8.50
7.19
7.17
7.15
6.93
6.92
6.92
6.91
6.86
6.85
6.85
6.84
4.53
4.51
4.50
4.14
4.11
4.09
4.06
4.05
3.76
3.72
3.68
2.23
2.21
2.19
2.18
2.16
2.15
2.14
2.14
2.11
2.09
2.07
2.05
2.04
2.03
2.01
1.98
1.67
1.65
1.64
1.62
1.61
1.59
1.57
1.56
1.54
1.52
1.51
1.34
1.34
1.29
2.01
2.62
0.86
0.86
1.00
3.07
2.13
3.26
2.19
1.12
f1 (ppm)

19

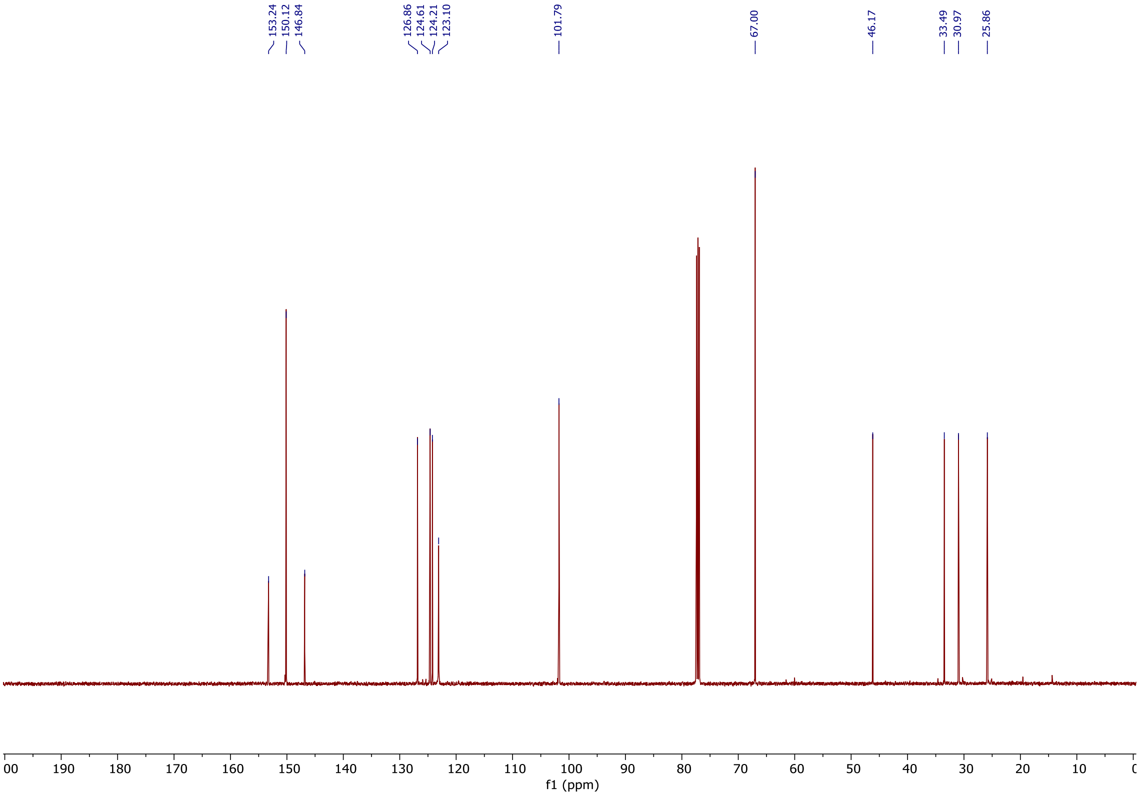
153.24
150.12
146.84
126.86
124.61
124.21
123.10
101.79
67.00
46.17
33.49
30.97
25.86
f1 (ppm)

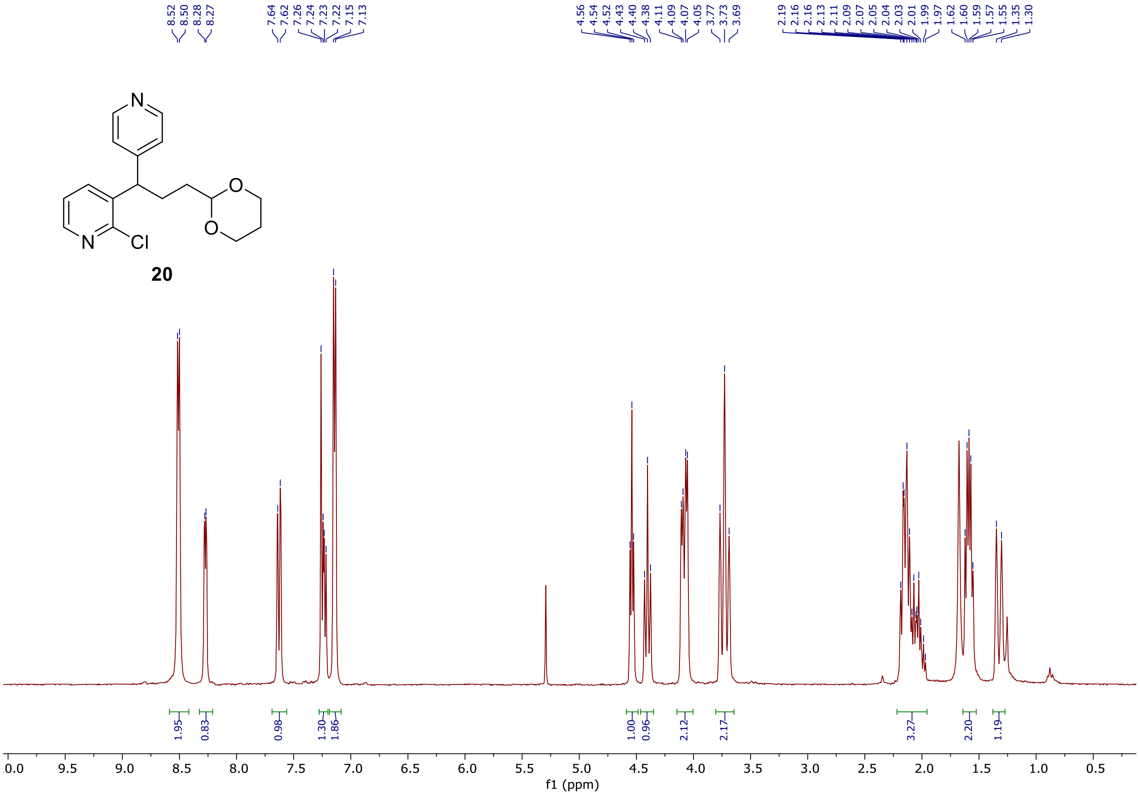

20
f1 (ppm)

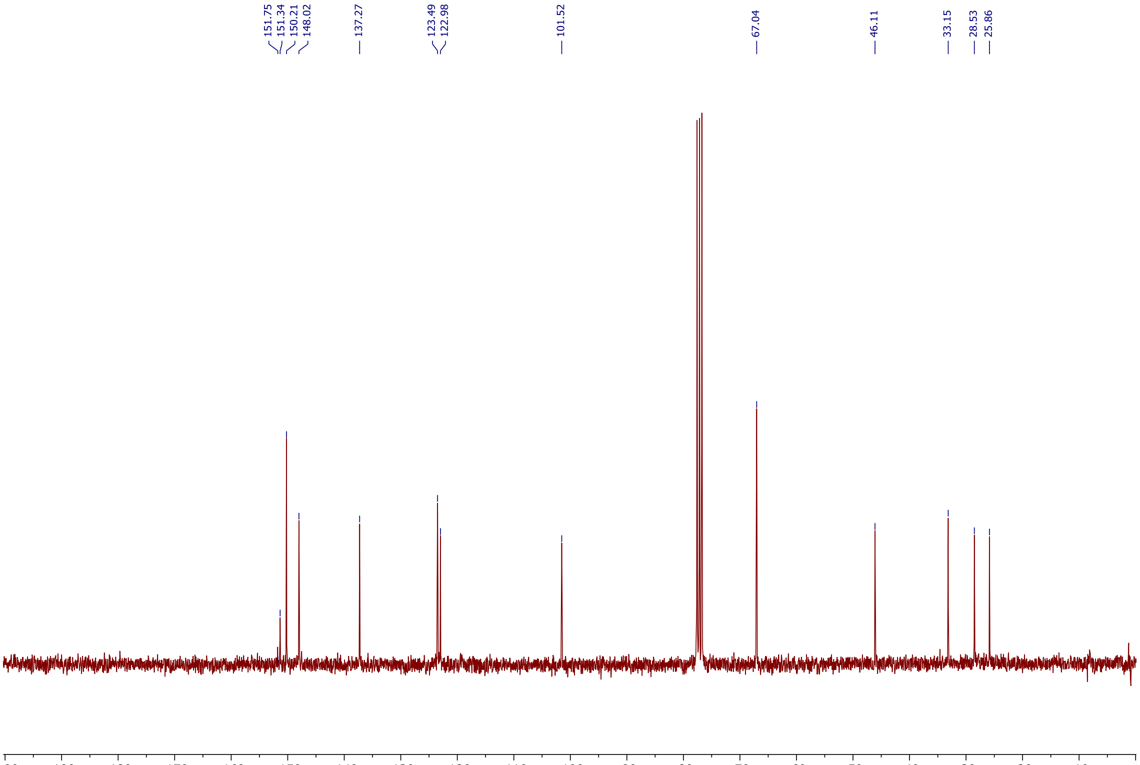

f1 (ppm)

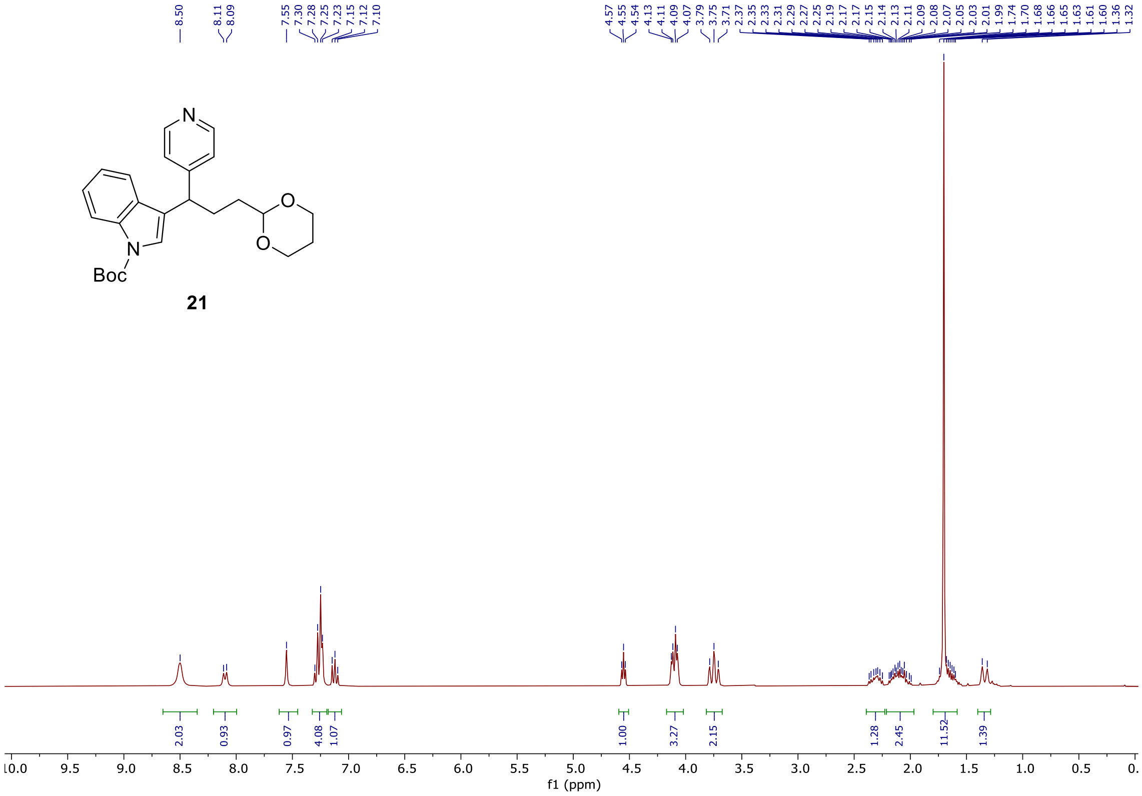

N
O
O
N
Boc
21
8.50
8.11
8.09
7.55
7.30
7.28
7.25
7.23
7.15
7.12
7.10
4.57
4.55
4.54
4.13
4.11
4.09
4.07
3.79
3.75
3.71
2.37
2.35
2.33
2.31
2.29
2.27
2.25
2.19
2.17
2.17
2.15
2.14
2.13
2.11
2.09
2.08
2.07
2.05
2.03
2.01
1.99
1.74
1.70
1.68
1.66
1.65
1.63
1.61
1.60
1.36
1.32
2.03
0.93
0.97
4.08
1.07
1.00
3.27
2.15
1.28
2.45
11.52
1.39
f1 (ppm)

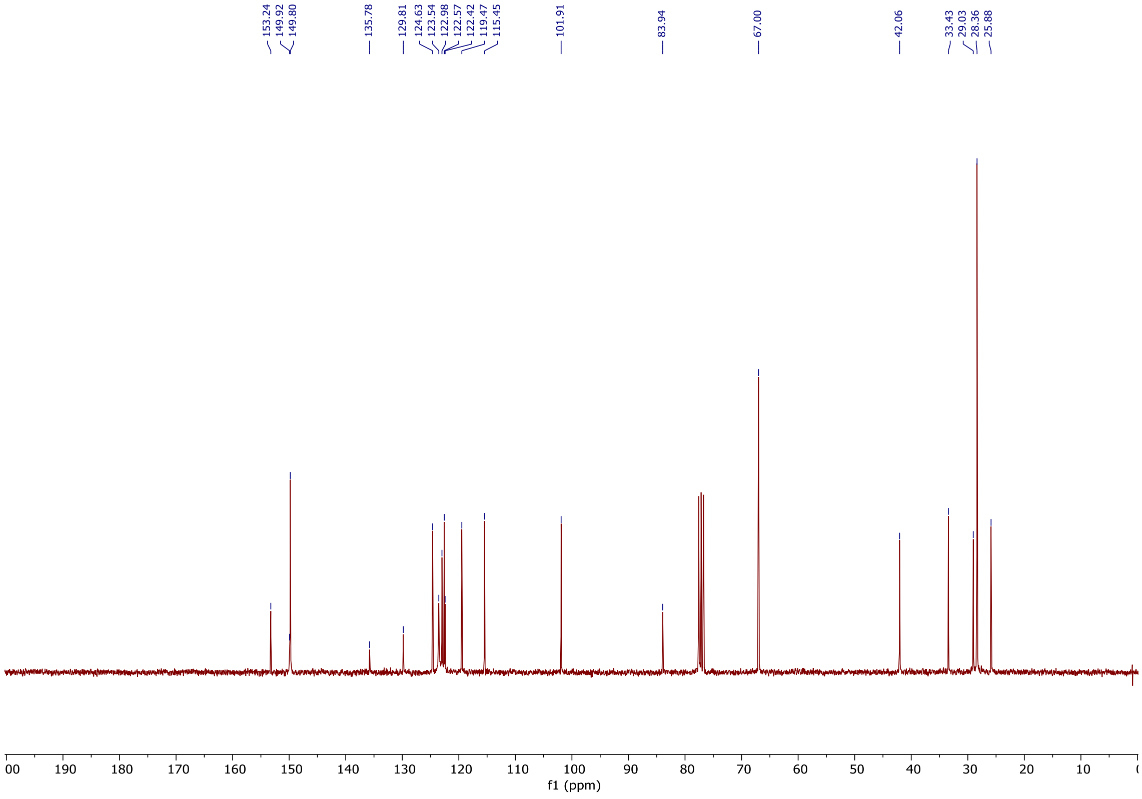

153.24
149.92
149.80
135.78
129.81
124.63
123.54
122.98
122.57
122.42
119.47
115.45
101.91
83.94
67.00
42.06
33.43
29.03
28.36
25.88
f1 (ppm)

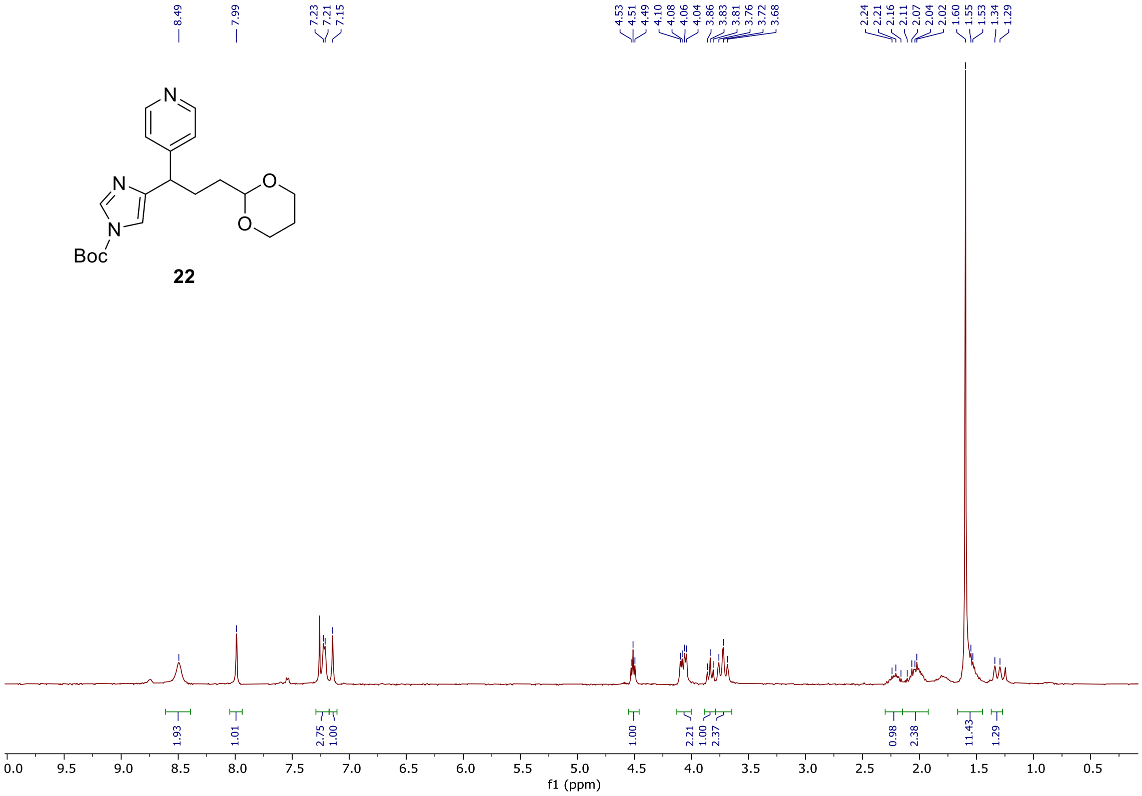

8.49
7.99
7.23
7.21
7.15
4.53
4.51
4.49
4.10
4.08
4.06
4.04
3.86
3.83
3.81
3.76
3.72
3.68
2.24
2.21
2.16
2.11
2.07
2.04
2.02
1.60
1.55
1.53
1.34
1.29
N
N
N
O
O
Boc
22
1.93
1.01
2.75
1.00
1.00
2.21
1.00
2.37
0.98
2.38
11.43
1.29
0.0 9.5 9.0 8.5 8.0 7.5 7.0 6.5 6.0 5.5 5.0 4.5 4.0 3.5 3.0 2.5 2.0 1.5 1.0 0.5
f1 (ppm)

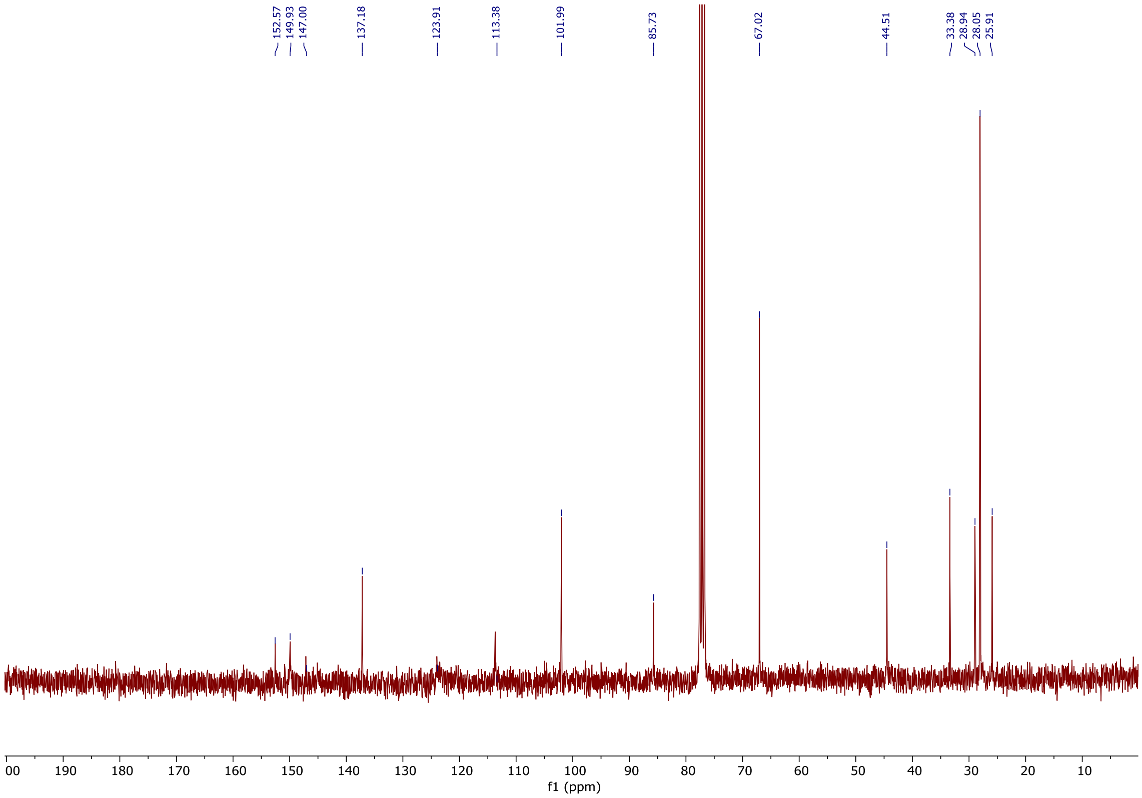

152.57
149.93
147.00
137.18
123.91
113.38
101.99
85.73
67.02
44.51
33.38
28.94
28.05
25.91
00 190 180 170 160 150 140 130 120 110 100 90 80 70 60 50 40 30 20 10
f1 (ppm)

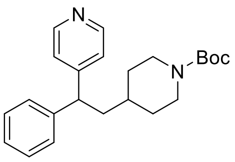
N
Boc

23

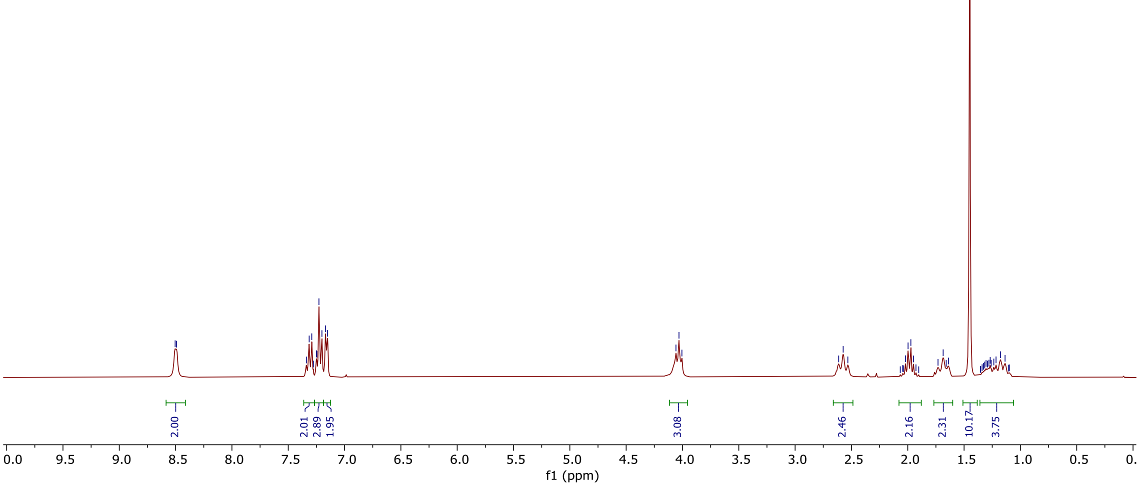
8.50
8.49
7.34
7.31
7.29
7.28
7.25
7.25
7.23
7.20
7.17
7.15
4.06
4.03
4.01
2.61
2.57
2.53
2.07
2.04
2.04
2.02
2.00
1.97
1.95
1.93
1.90
1.73
1.69
1.66
1.64
1.45
1.35
1.35
1.34
1.33
1.32
1.30
1.29
1.28
1.27
1.26
1.24
1.22
1.18
1.14
1.11
1.10
2.00
2.01
2.89
1.95
3.08
2.46
2.16
2.31
10.17
3.75
f1 (ppm)

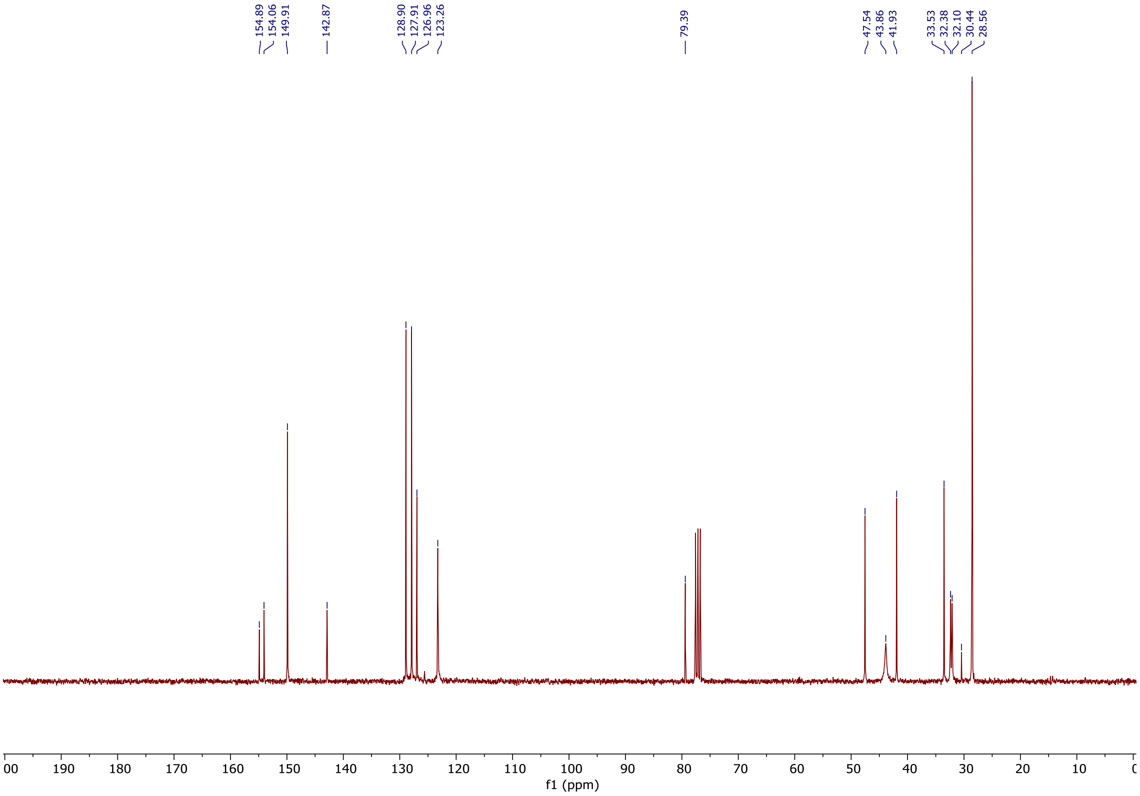
154.89
154.06
149.91
142.87
128.90
127.91
126.96
123.26
79.39
47.54
43.86
41.93
33.53
32.38
32.10
30.44
28.56
f1 (ppm)

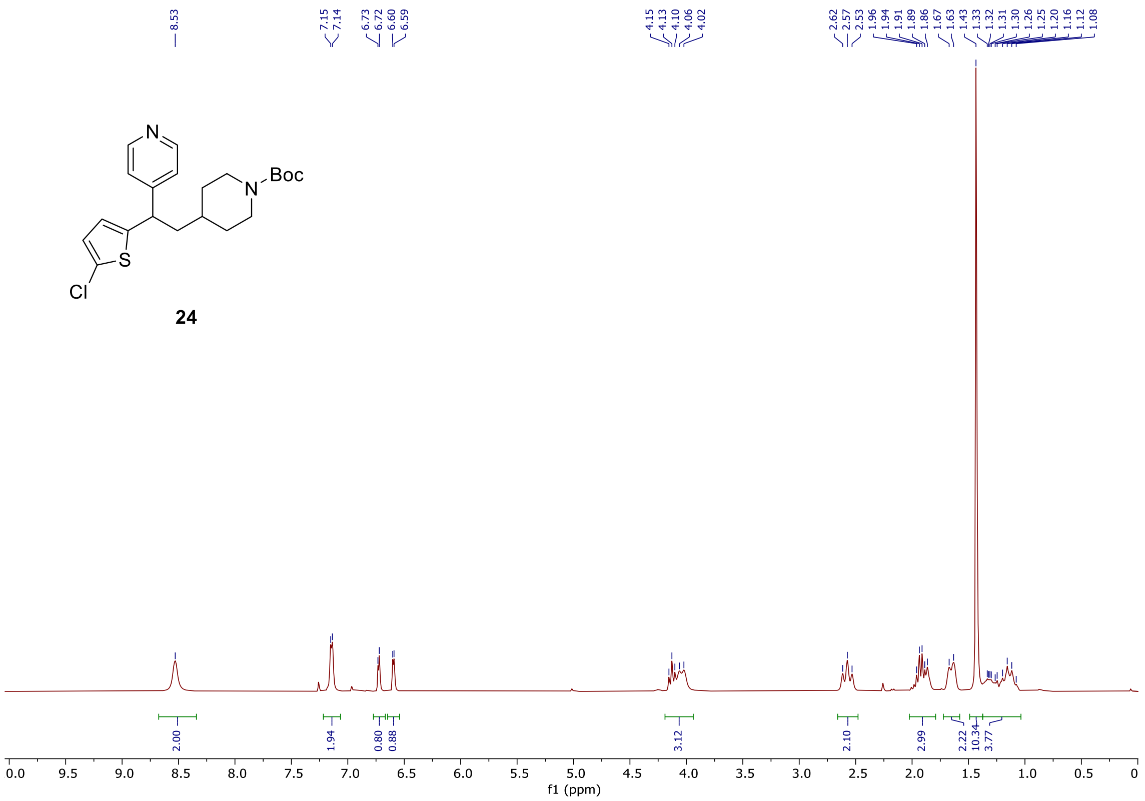

N
Boc
S
Cl
24
f1 (ppm)

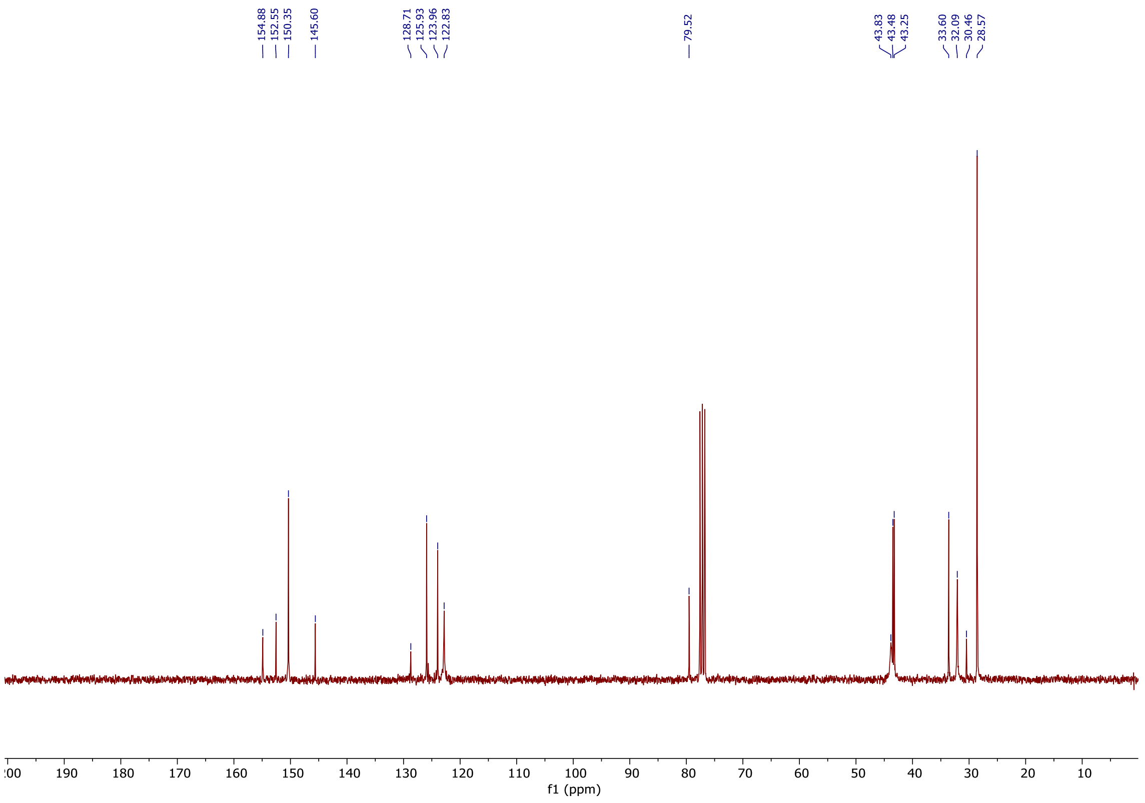

f1 (ppm)

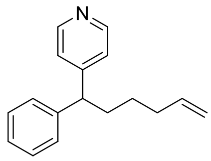

25

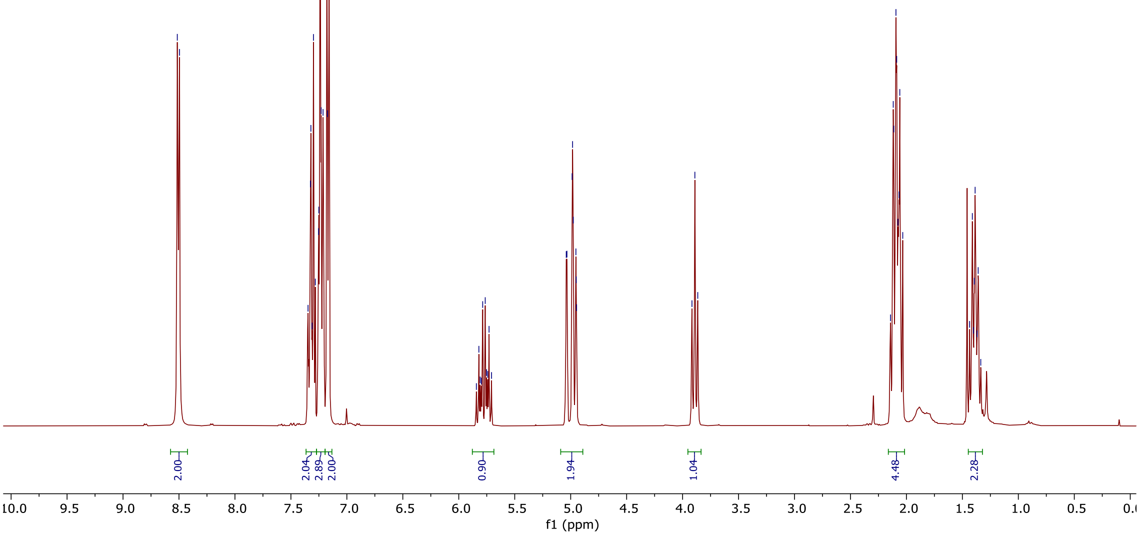

8.52
8.50
7.35
7.33
7.32
7.31
7.30
7.28
7.25
7.25
7.24
7.24
7.23
7.21
7.18
7.17
7.16
5.84
5.82
5.81
5.80
5.79
5.76
5.75
5.74
5.73
5.71
5.04
5.03
4.99
4.98
4.98
4.95
4.95
4.95
3.92
3.89
3.86
2.14
2.12
2.11
2.09
2.09
2.08
2.07
2.06
2.03
1.44
1.41
1.40
1.39
1.39
1.37
1.36
1.34
2.00
2.04
2.89
2.00
0.90
1.94
1.04
4.48
2.28
f1 (ppm)

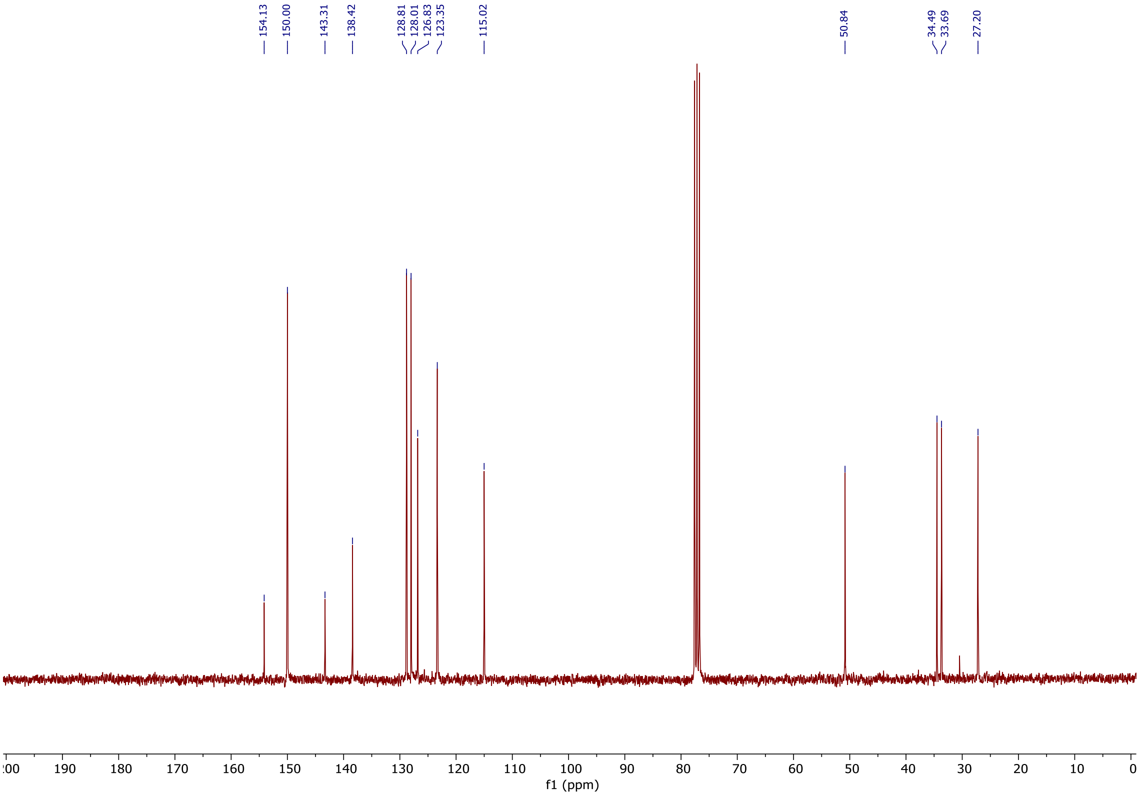

154.13
150.00
143.31
138.42
128.81
128.01
126.83
123.35
115.02
50.84
34.49
33.69
27.20
f1 (ppm)

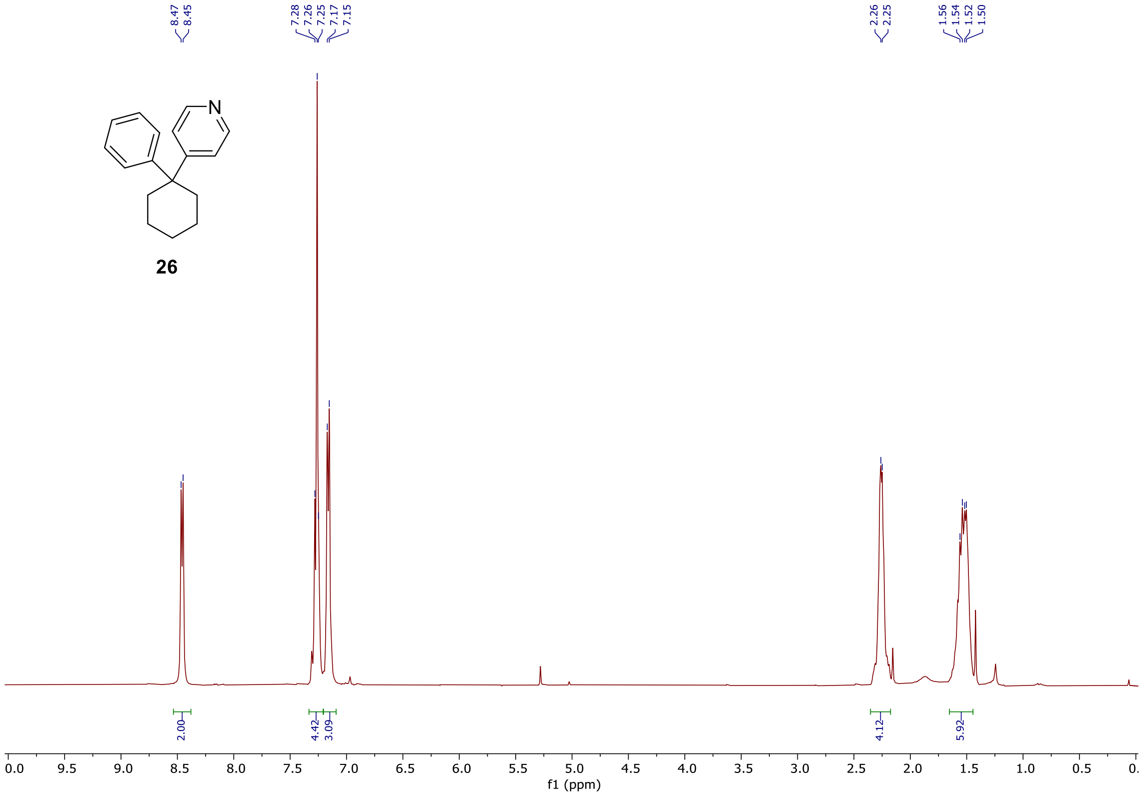
26
8.47
8.45
7.28
7.26
7.25
7.17
7.15
2.26
2.25
1.56
1.54
1.52
1.50
2.00
4.42
3.09
4.12
5.92
0.0 9.5 9.0 8.5 8.0 7.5 7.0 6.5 6.0 5.5 5.0 4.5 4.0 3.5 3.0 2.5 2.0 1.5 1.0 0.5 0.
f1 (ppm)

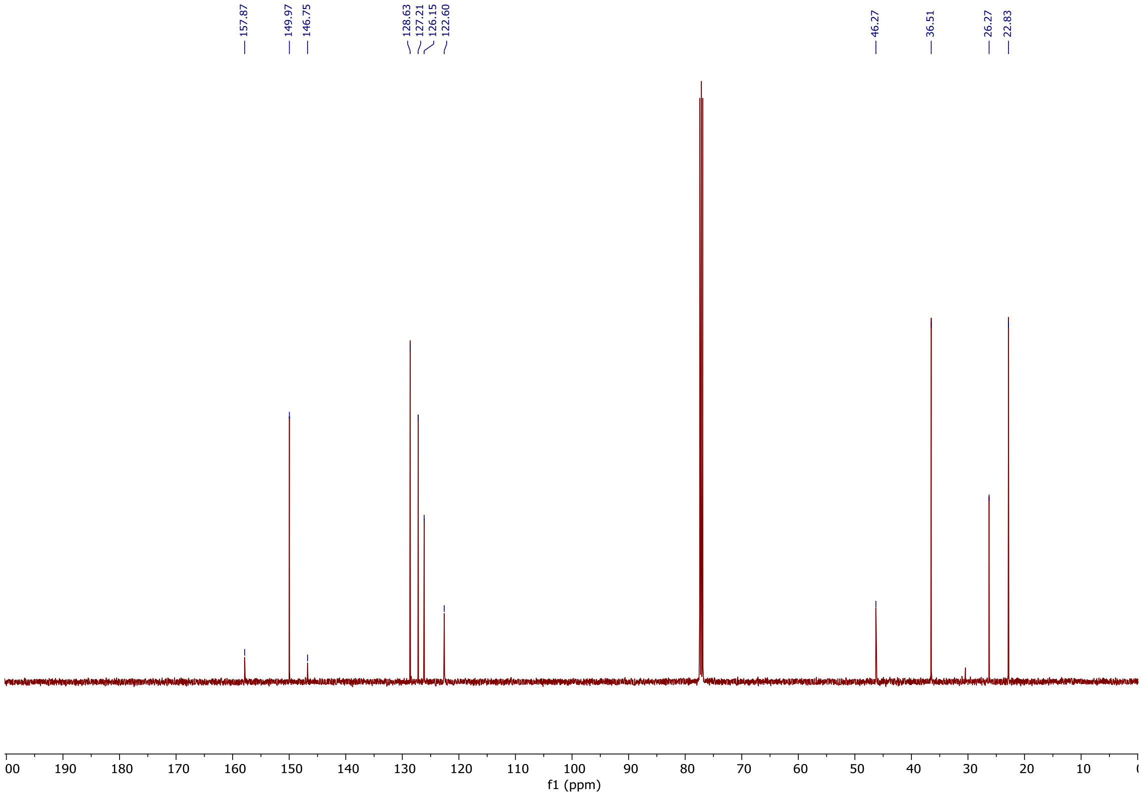
157.87
149.97
146.75
128.63
127.21
126.15
122.60
46.27
36.51
26.27
22.83
00 190 180 170 160 150 140 130 120 110 100 90 80 70 60 50 40 30 20 10
f1 (ppm)

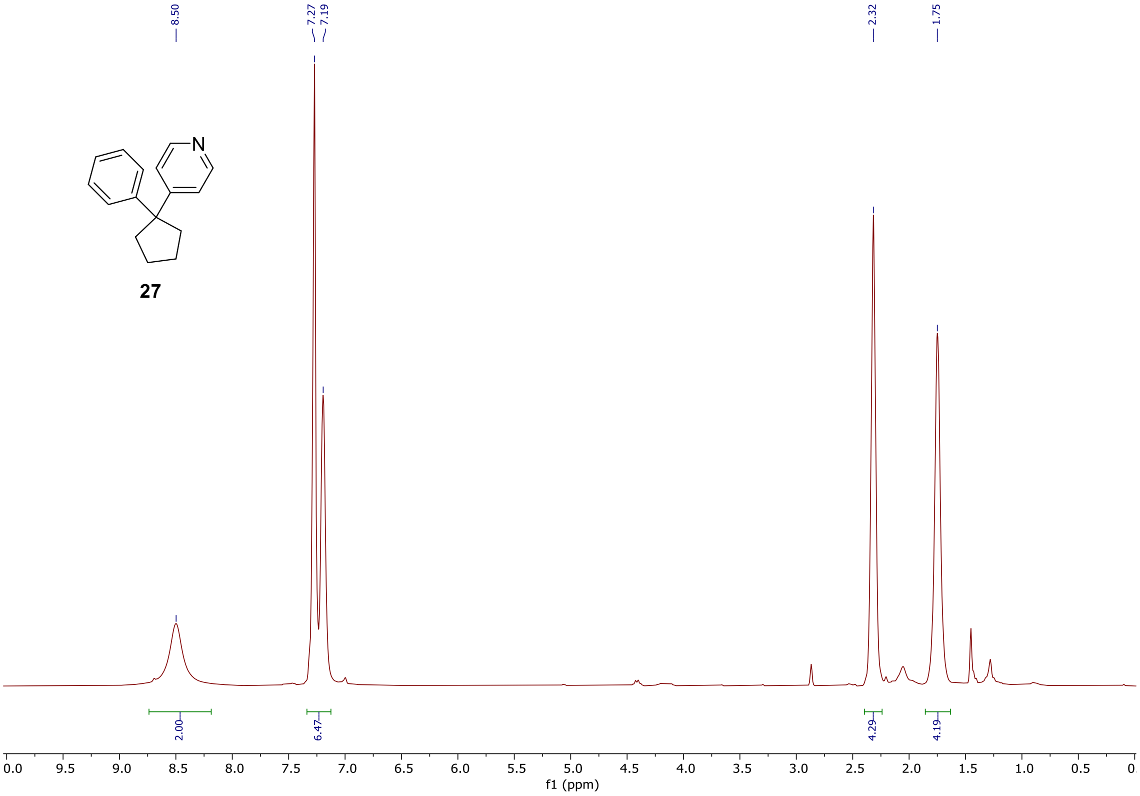
N
27
8.50
7.27
7.19
2.32
1.75
2.00
6.47
4.29
4.19
f1 (ppm)

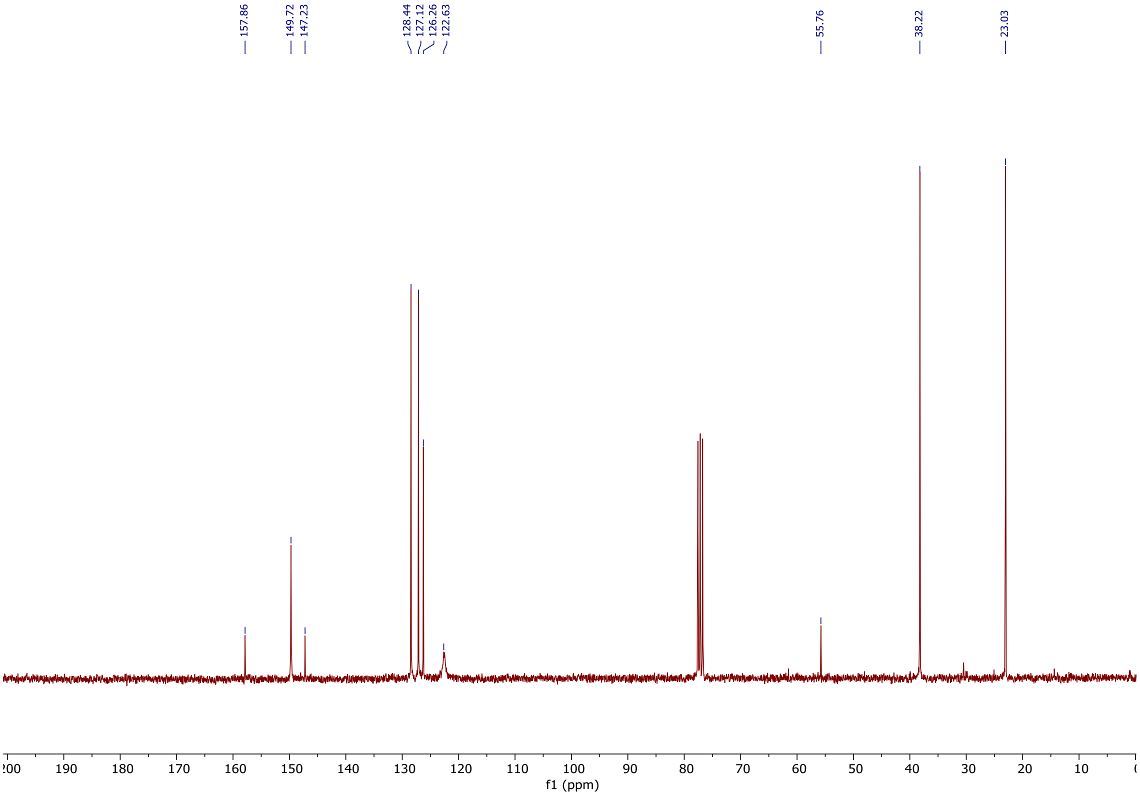
157.86
149.72
147.23
128.44
127.12
126.26
122.63
55.76
38.22
23.03
f1 (ppm)

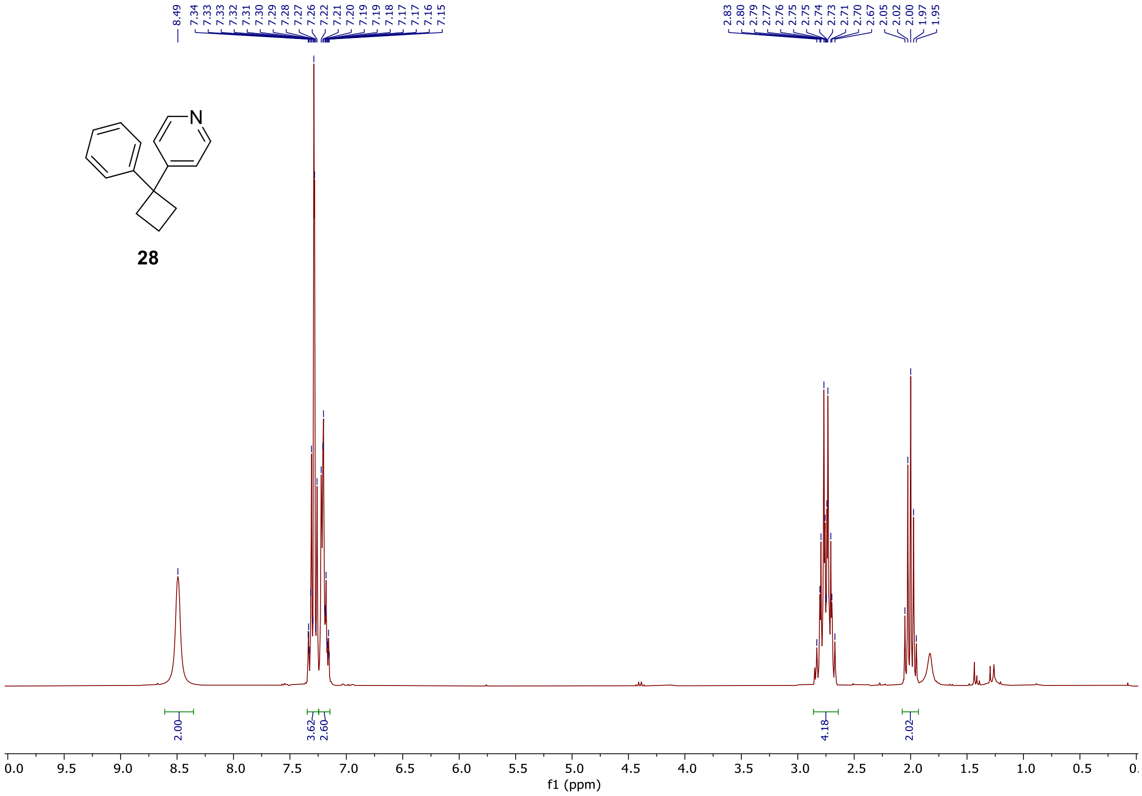
8.49
7.34 7.33 7.33 7.32 7.31 7.30 7.29 7.28 7.27 7.26 7.22 7.21 7.20 7.19 7.19 7.18 7.17 7.17 7.16 7.15
2.83 2.80 2.79 2.77 2.76 2.75 2.75 2.74 2.73 2.71 2.70 2.67 2.05 2.02 2.00 1.97 1.95
N
28
2.00
3.62
2.60
4.18
2.02
f1 (ppm)

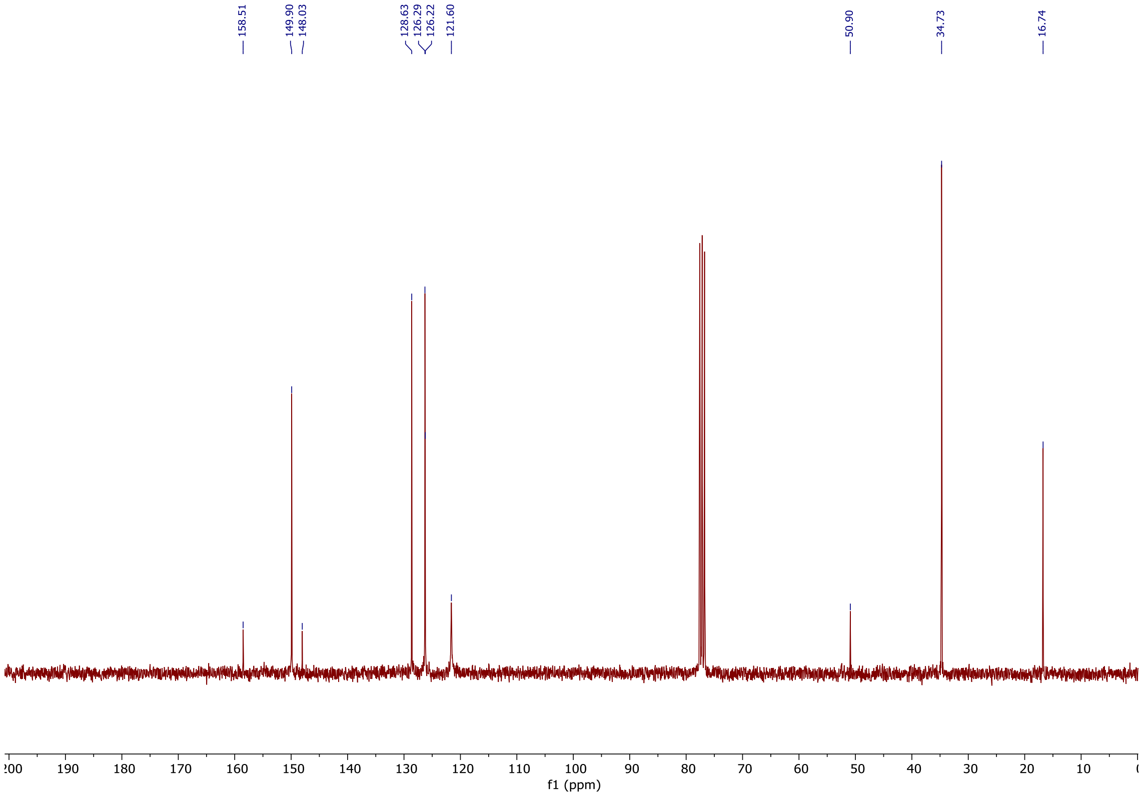
158.51
149.90
148.03
128.63
126.29
126.22
121.60
50.90
34.73
16.74
f1 (ppm)

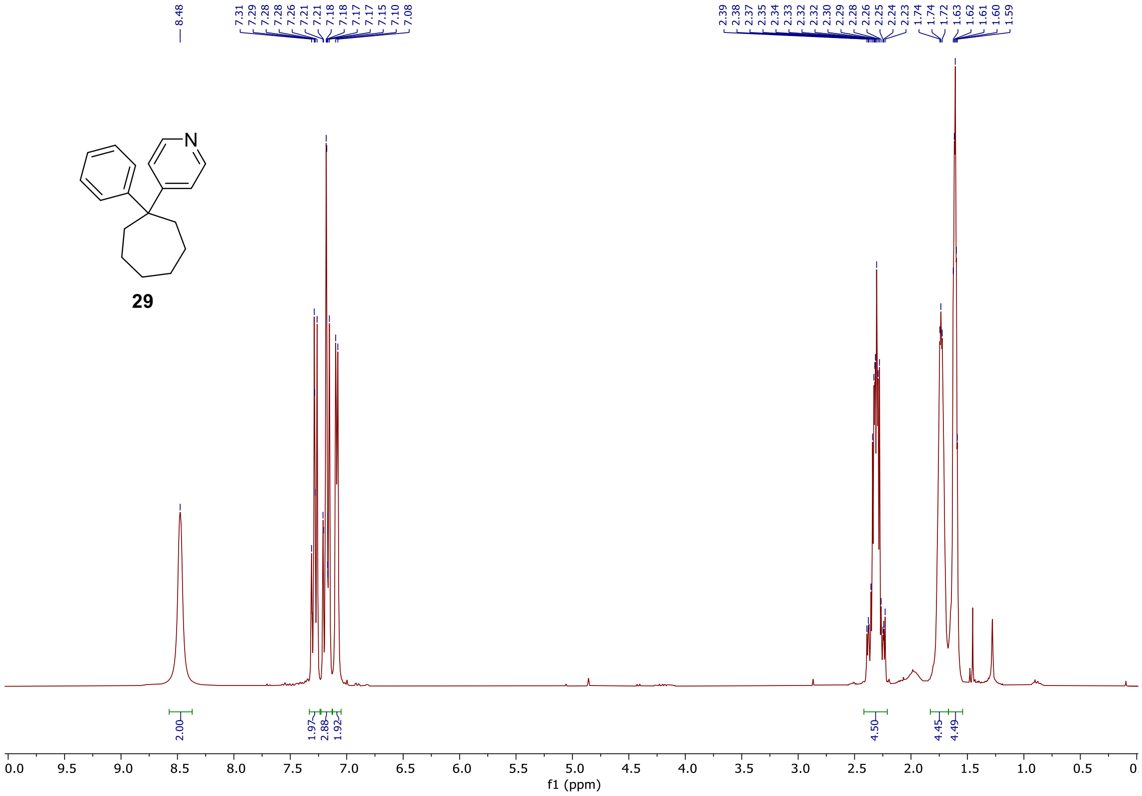
8.48
7.31 7.29 7.28 7.28 7.26 7.21 7.21 7.18 7.18 7.17 7.17 7.15 7.10 7.08
2.39 2.38 2.37 2.35 2.34 2.33 2.32 2.32 2.30 2.29 2.28 2.26 2.25 2.24 2.23 1.74 1.74 1.72 1.63 1.62 1.61 1.60 1.59
2.00
1.97
2.88
1.92
4.50
4.45
4.49
f1 (ppm)

29

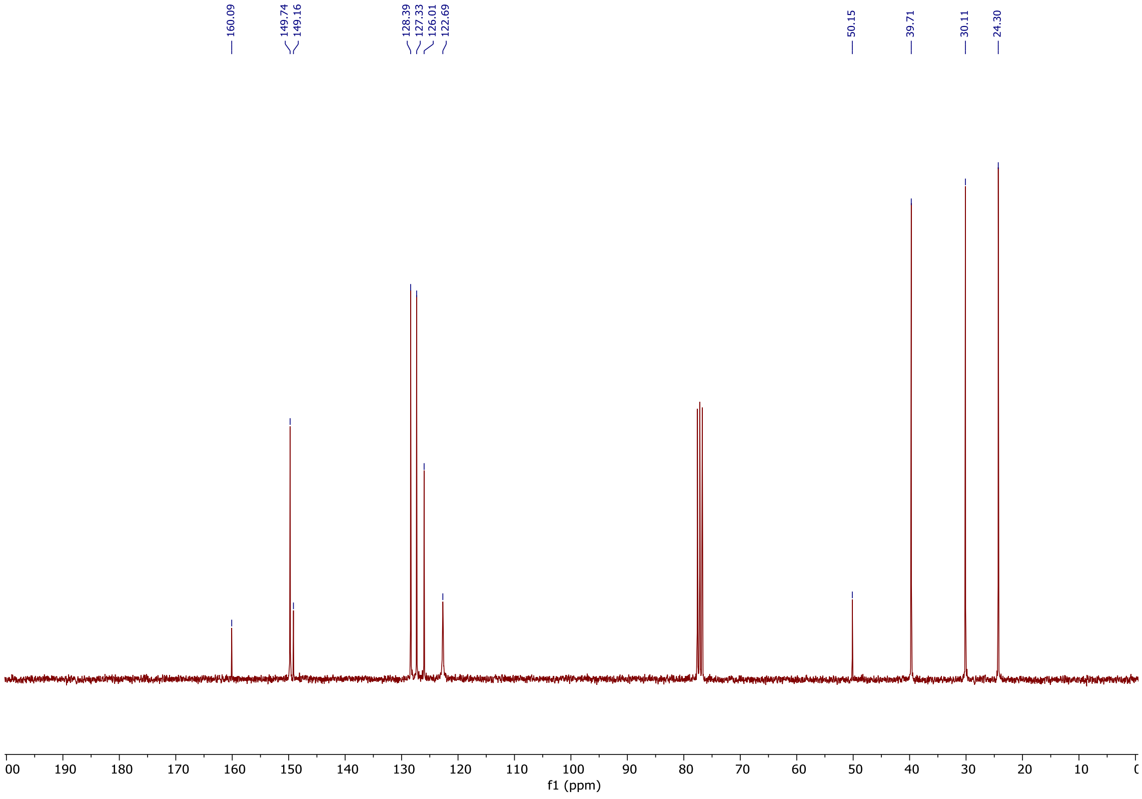
160.09
149.74 149.16
128.39 127.33 126.01 122.69
50.15
39.71
30.11
24.30
f1 (ppm)

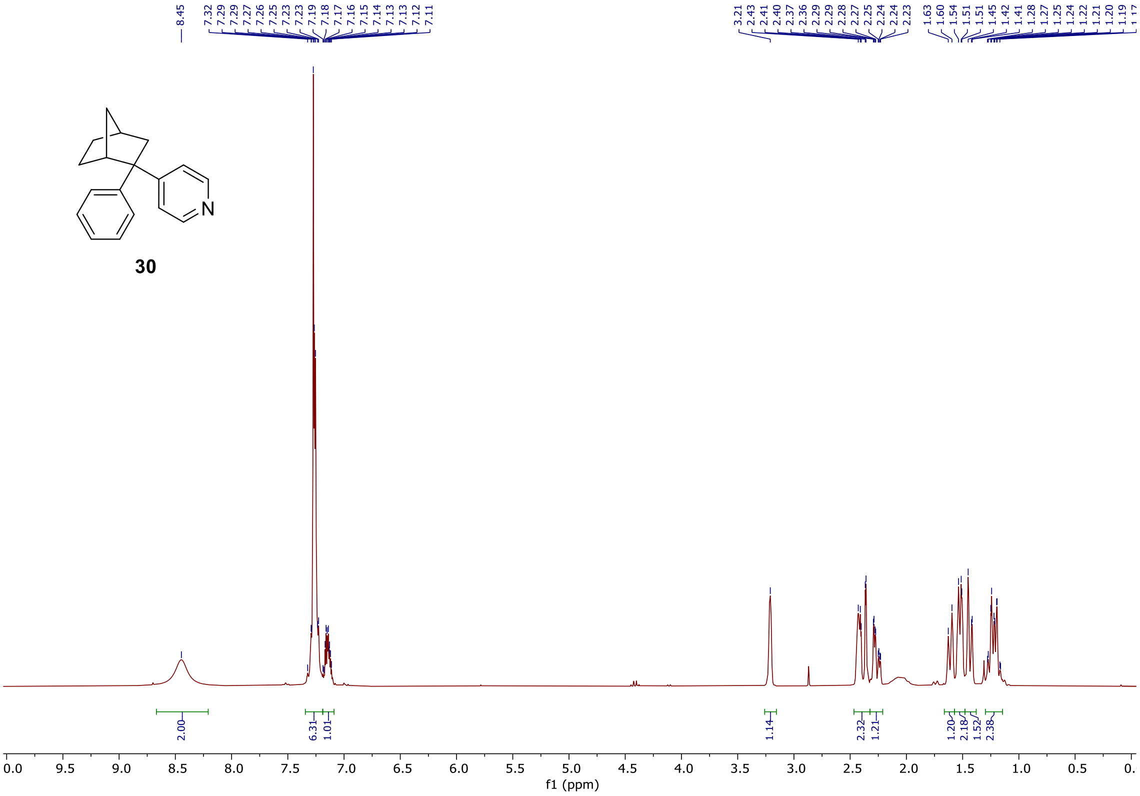

8.45
7.32 7.29 7.29 7.27 7.26 7.25 7.23 7.23 7.19 7.18 7.17 7.16 7.15 7.14 7.13 7.13 7.12 7.11
3.21 2.43 2.41 2.40 2.37 2.36 2.29 2.29 2.28 2.27 2.25 2.24 2.24 2.23
1.63 1.60 1.54 1.51 1.51 1.45 1.42 1.41 1.28 1.27 1.25 1.24 1.22 1.21 1.20 1.19
2.00
6.31
1.01
1.14
2.32
1.21
1.20
2.18
1.52
2.38
0.0 9.5 9.0 8.5 8.0 7.5 7.0 6.5 6.0 5.5 5.0 4.5 4.0 3.5 3.0 2.5 2.0 1.5 1.0 0.5 0.
f1 (ppm)
N

30

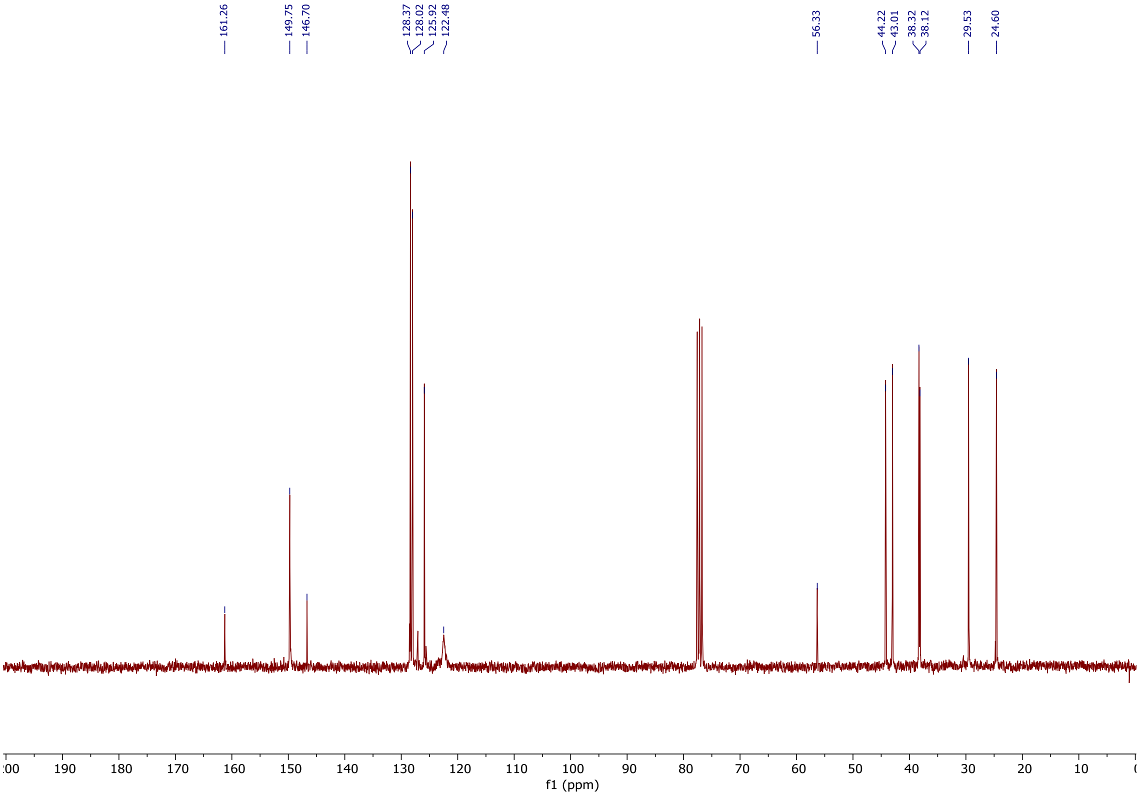

161.26
149.75
146.70
128.37 128.02 125.92 122.48
56.33
44.22 43.01
38.32 38.12
29.53
24.60
00 190 180 170 160 150 140 130 120 110 100 90 80 70 60 50 40 30 20 10
f1 (ppm)

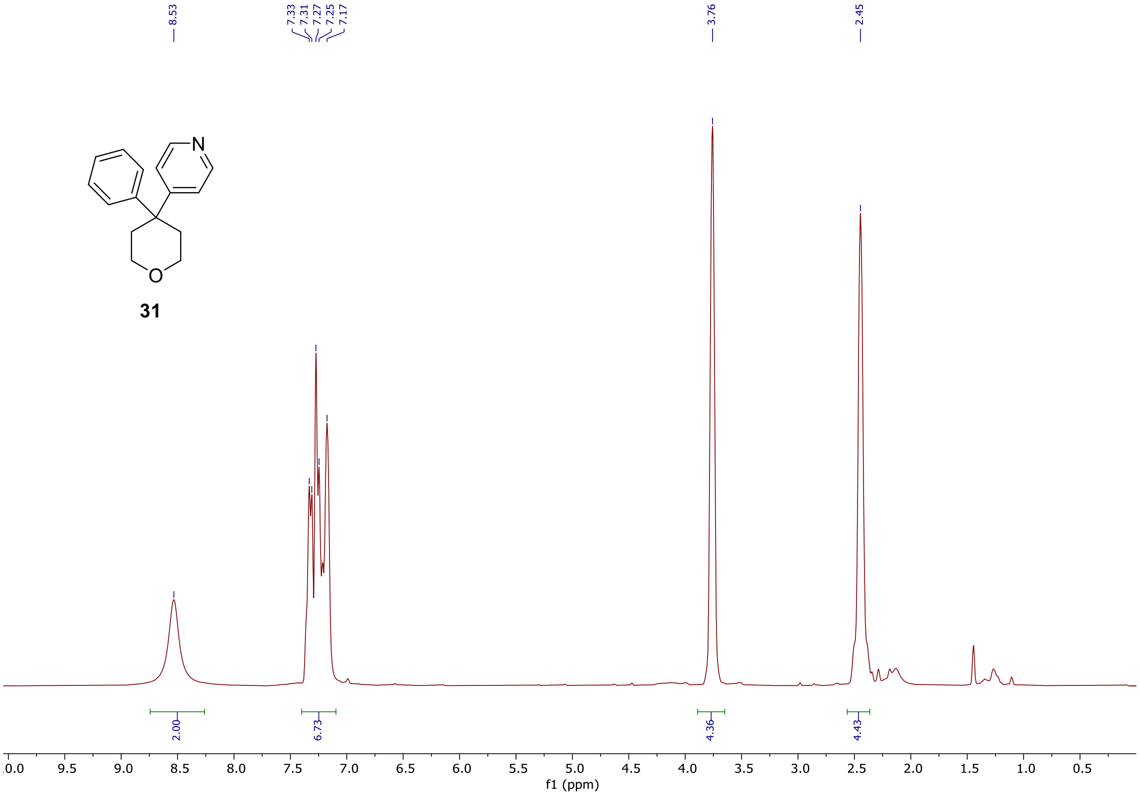

8.53
7.33
7.31
7.27
7.25
7.17
3.76
2.45
N
O
31
2.00
6.73
4.36
4.43
10.0 9.5 9.0 8.5 8.0 7.5 7.0 6.5 6.0 5.5 5.0 4.5 4.0 3.5 3.0 2.5 2.0 1.5 1.0 0.5
f1 (ppm)

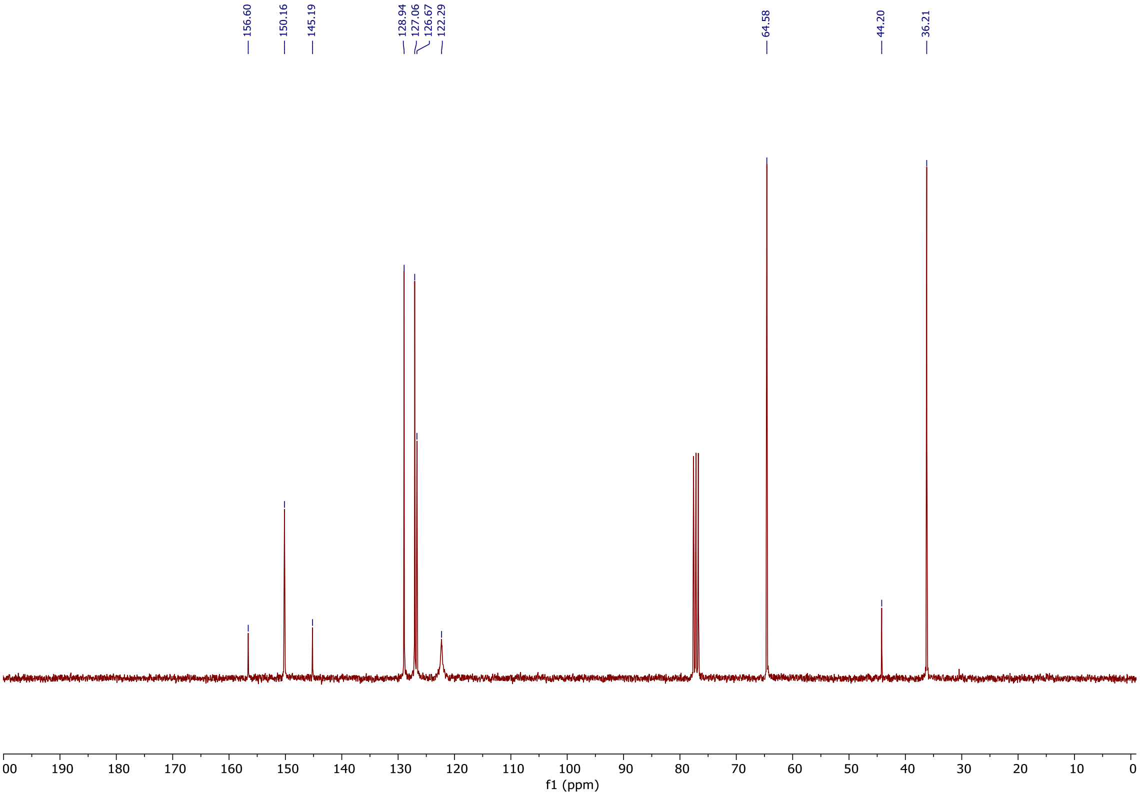

156.60
150.16
145.19
128.94
127.06
126.67
122.29
64.58
44.20
36.21
200 190 180 170 160 150 140 130 120 110 100 90 80 70 60 50 40 30 20 10 0
f1 (ppm)

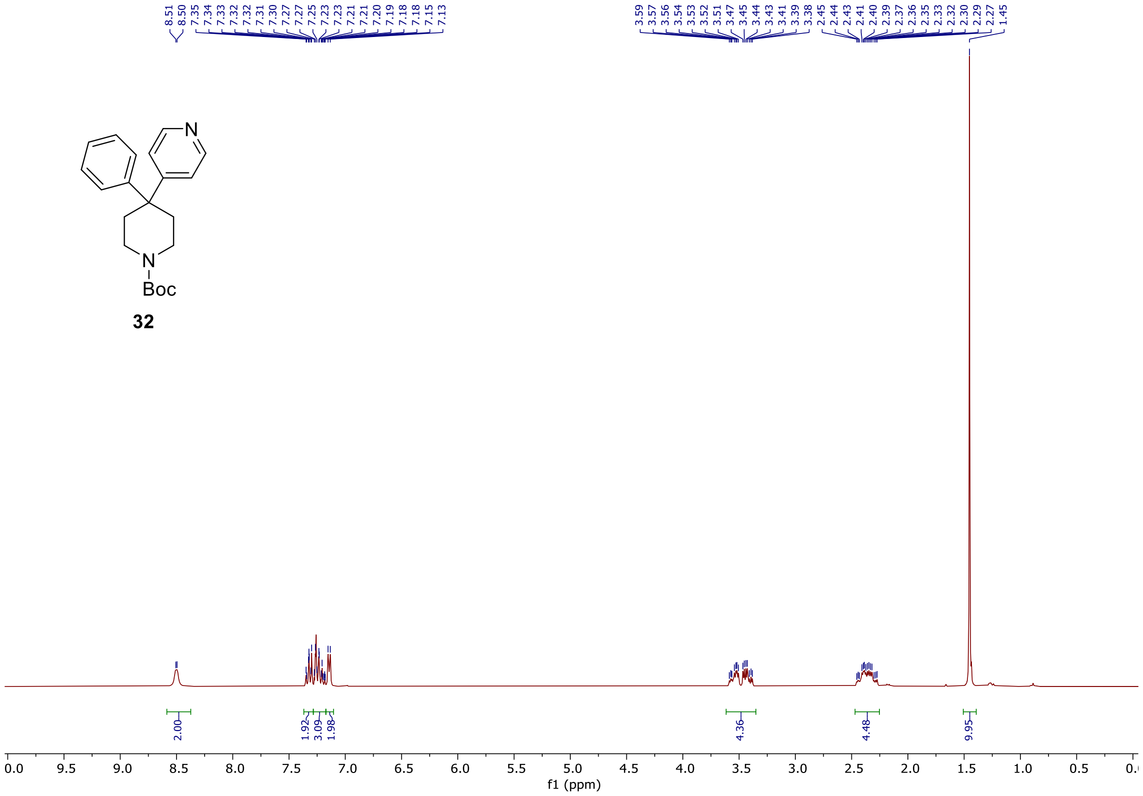
8.51
8.50
7.35
7.34
7.33
7.32
7.32
7.31
7.30
7.27
7.27
7.25
7.23
7.23
7.21
7.21
7.20
7.19
7.18
7.15
7.13
3.59
3.57
3.56
3.54
3.53
3.52
3.51
3.47
3.45
3.44
3.43
3.41
3.39
3.38
2.45
2.44
2.43
2.41
2.40
2.39
2.37
2.36
2.35
2.33
2.32
2.30
2.29
2.27
1.45
N
N
Boc
2.00
1.92
3.09
1.98
4.36
4.48
9.95
f1 (ppm)

32

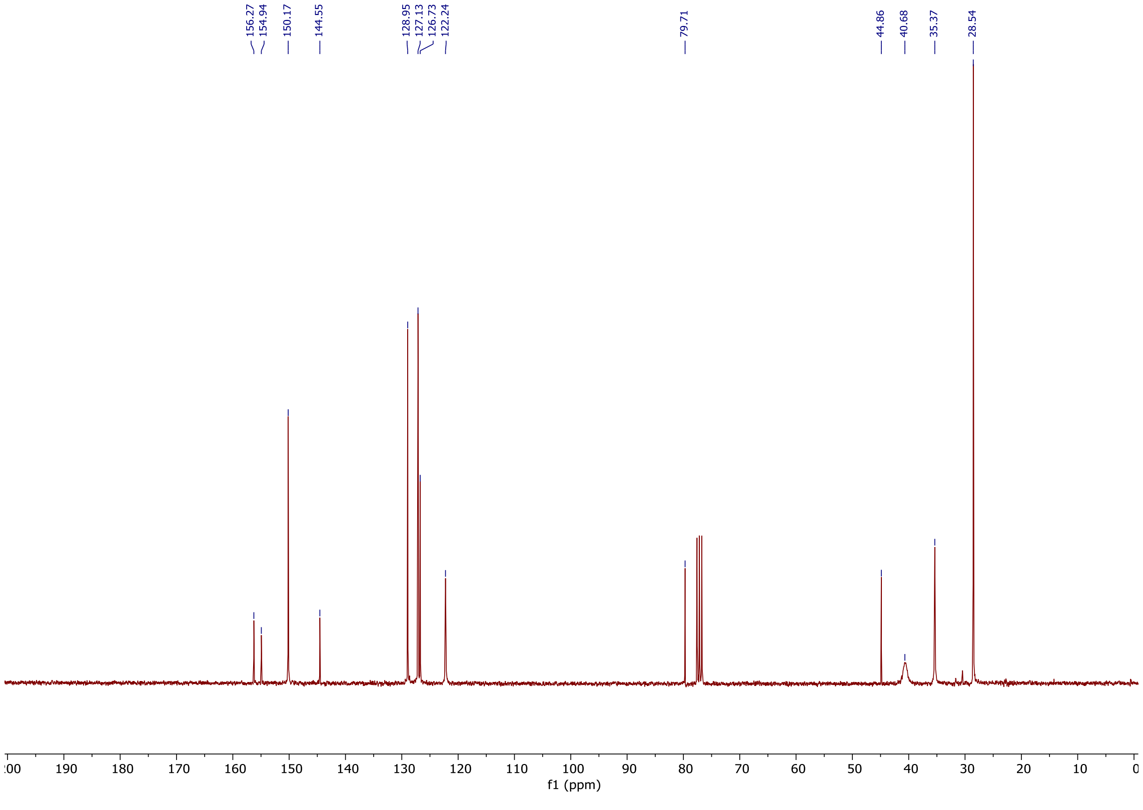
156.27
154.94
150.17
144.55
128.95
127.13
126.73
122.24
79.71
44.86
40.68
35.37
28.54
f1 (ppm)

33

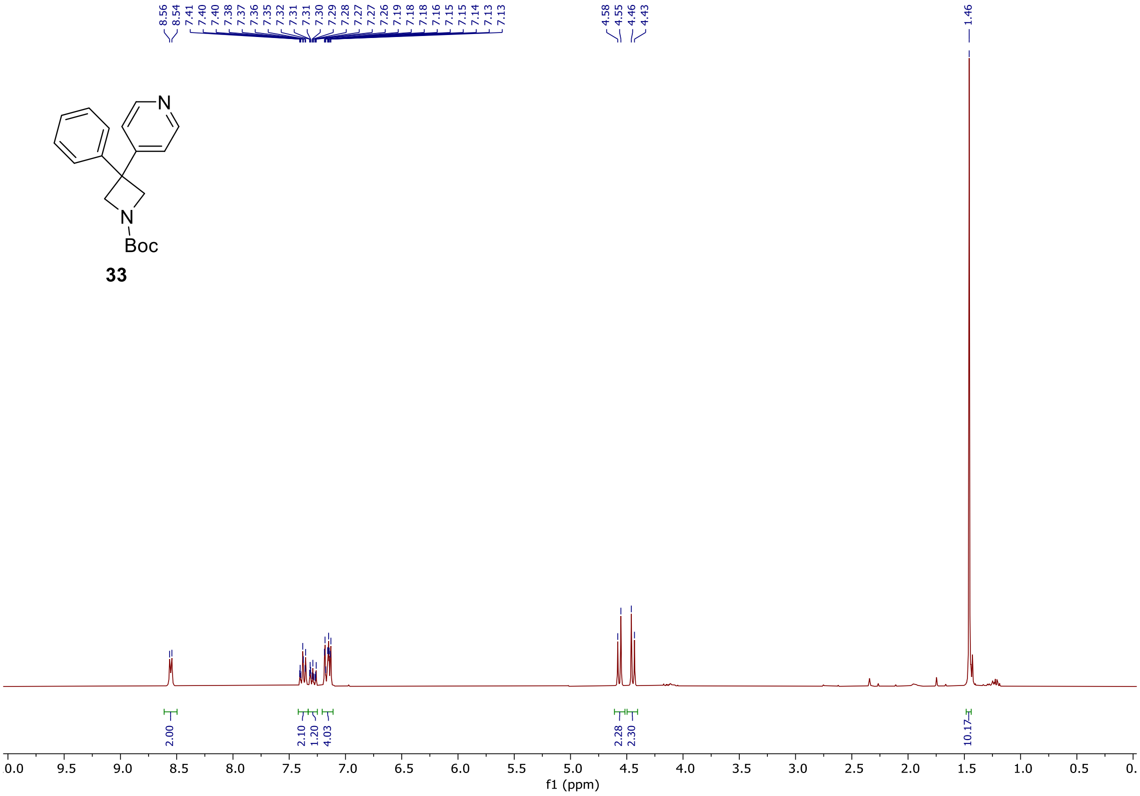
N
N
Boc
8.56
8.54
7.41
7.40
7.38
7.37
7.36
7.35
7.32
7.31
7.31
7.30
7.29
7.28
7.27
7.27
7.26
7.19
7.18
7.18
7.16
7.15
7.15
7.14
7.13
7.13
4.58
4.55
4.46
4.43
1.46
2.00
2.10
1.20
4.03
2.28
2.30
10.17
f1 (ppm)

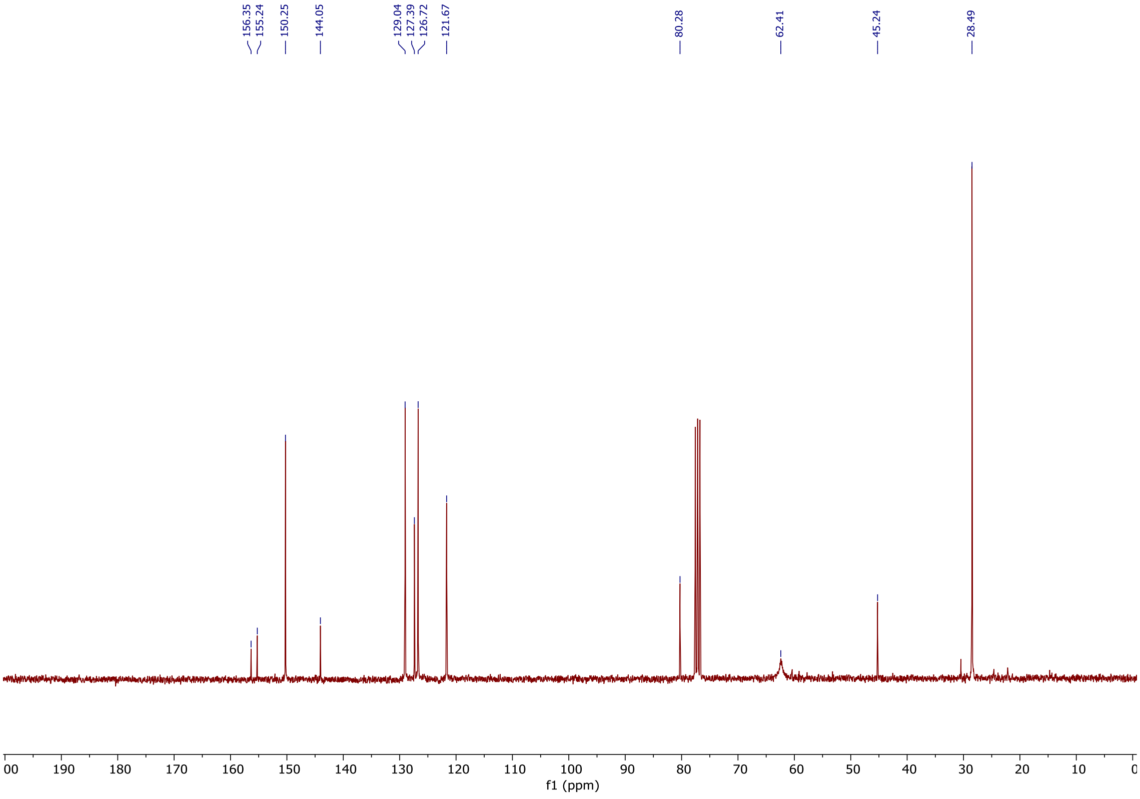
156.35
155.24
150.25
144.05
129.04
127.39
126.72
121.67
80.28
62.41
45.24
28.49
f1 (ppm)

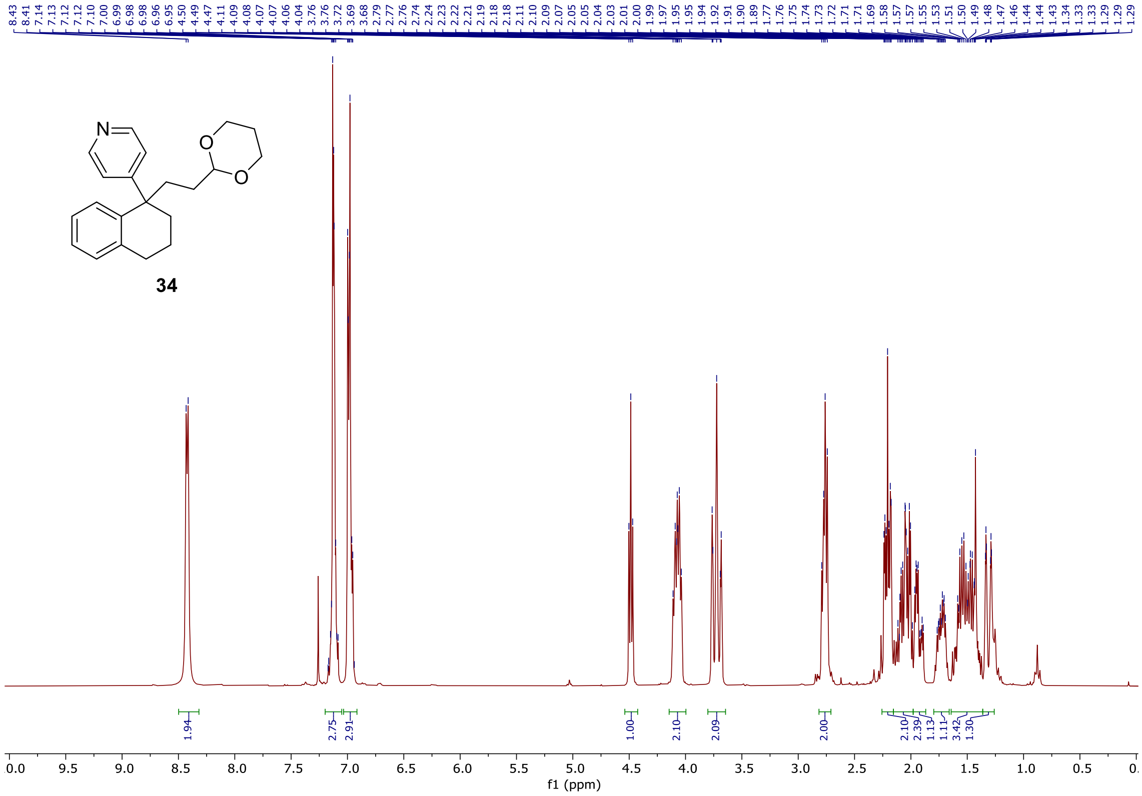

8.43 8.41 7.14 7.13 7.12 7.12 7.10 7.00 6.99 6.98 6.98 6.96 6.95 4.50 4.49 4.47 4.11 4.09 4.08 4.07 4.07 4.06 4.04 3.76 3.76 3.72 3.69 3.68 2.79 2.77 2.76 2.74 2.24 2.23 2.22 2.21 2.19 2.18 2.11 2.10 2.09 2.07 2.05 2.04 2.03 2.01 2.00 1.99 1.97 1.95 1.95 1.94 1.92 1.91 1.90 1.89 1.77 1.76 1.75 1.74 1.73 1.72 1.71 1.69 1.58 1.57 1.57 1.55 1.53 1.51 1.50 1.49 1.48 1.47 1.46 1.44 1.44 1.43 1.34 1.34 1.33 1.33 1.29 1.29
N
O
O
34
1.94
2.75
2.91
1.00
2.10
2.09
2.00
2.10
2.39
1.13
1.11
3.42
1.30
0.0 9.5 9.0 8.5 8.0 7.5 7.0 6.5 6.0 5.5 5.0 4.5 4.0 3.5 3.0 2.5 2.0 1.5 1.0 0.5 0.
f1 (ppm)

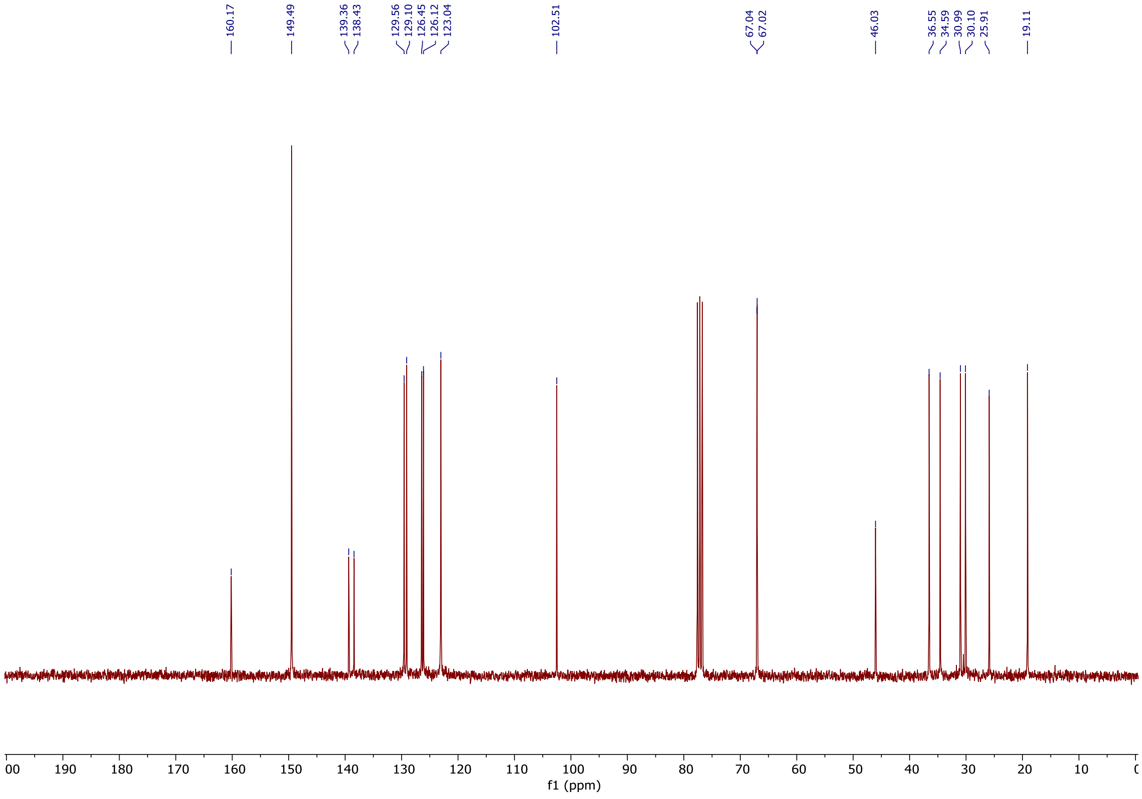

160.17
149.49
139.36 138.43
129.56 129.10 126.45 126.12 123.04
102.51
67.04 67.02
46.03
36.55 34.59 30.99 30.10 25.91
19.11
00 190 180 170 160 150 140 130 120 110 100 90 80 70 60 50 40 30 20 10
f1 (ppm)

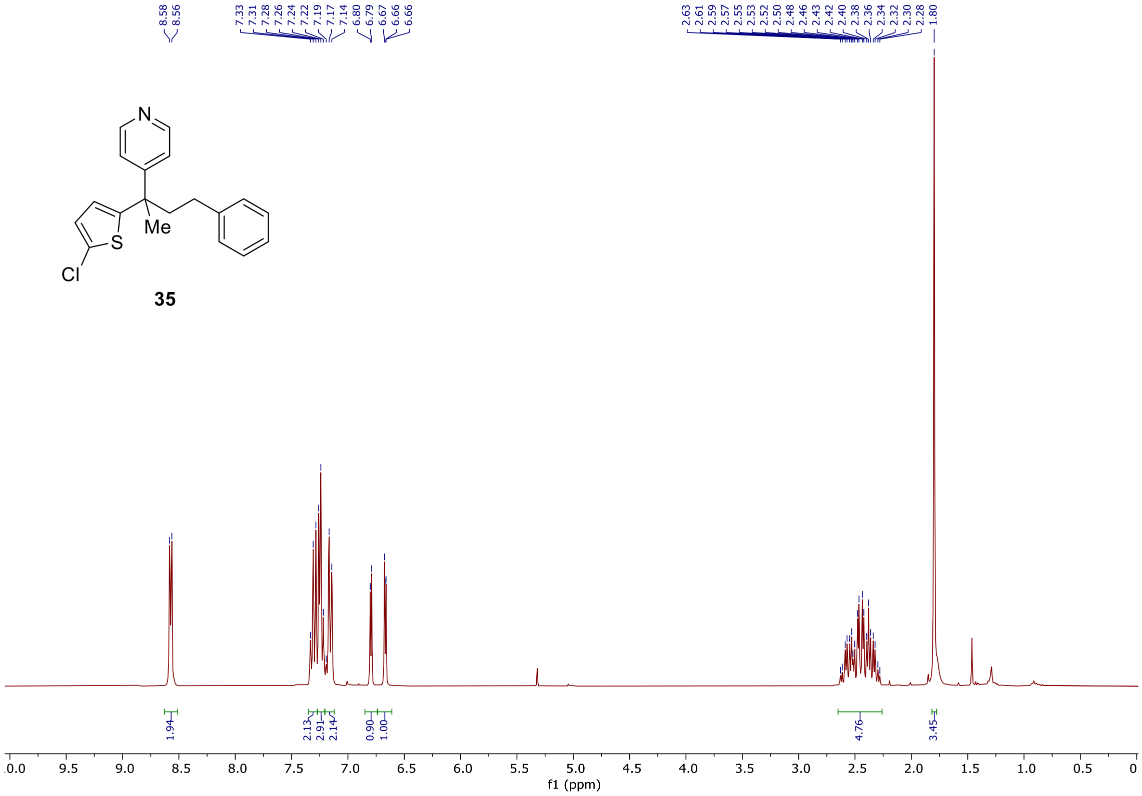
8.58
8.56
7.33
7.31
7.28
7.26
7.24
7.22
7.19
7.17
7.14
6.80
6.79
6.67
6.66
6.66
2.63
2.61
2.59
2.57
2.55
2.53
2.52
2.50
2.48
2.46
2.43
2.42
2.40
2.38
2.36
2.34
2.32
2.30
2.28
1.80
N
S
Me
Cl
35
1.94
2.13
2.91
2.14
0.90
1.00
4.76
3.45
f1 (ppm)

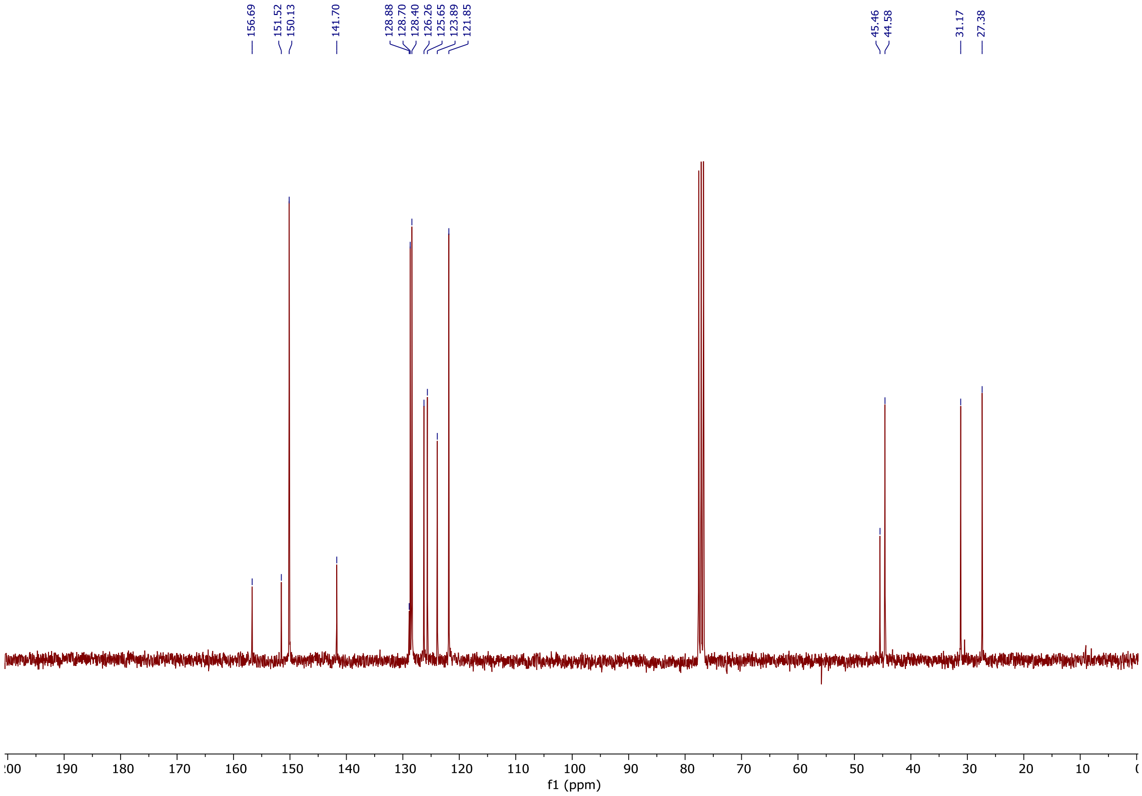
156.69
151.52
150.13
141.70
128.88
128.70
128.40
126.26
125.65
123.89
121.85
45.46
44.58
31.17
27.38
f1 (ppm)

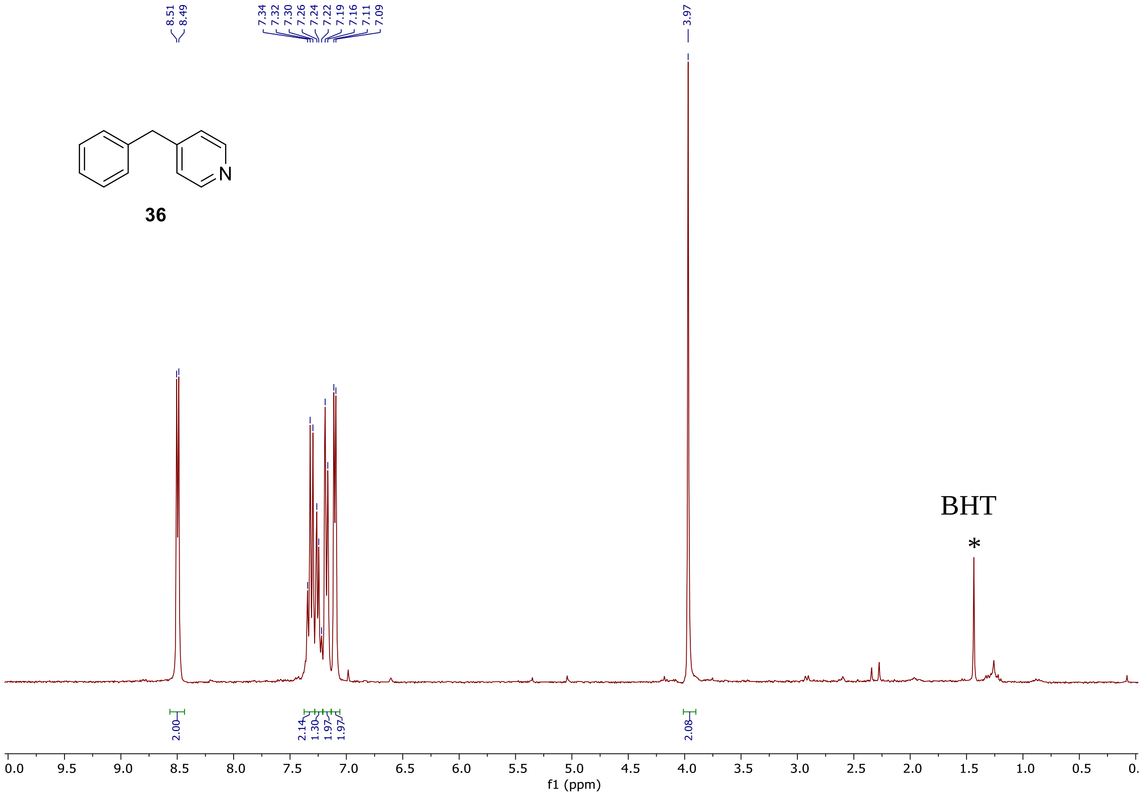
8.51
8.49
7.34
7.32
7.30
7.26
7.24
7.22
7.19
7.16
7.11
7.09
3.97
N
36
BHT
*
2.00
2.14
1.30
1.97
1.97
2.08
10.0 9.5 9.0 8.5 8.0 7.5 7.0 6.5 6.0 5.5 5.0 4.5 4.0 3.5 3.0 2.5 2.0 1.5 1.0 0.5 0.
f1 (ppm)

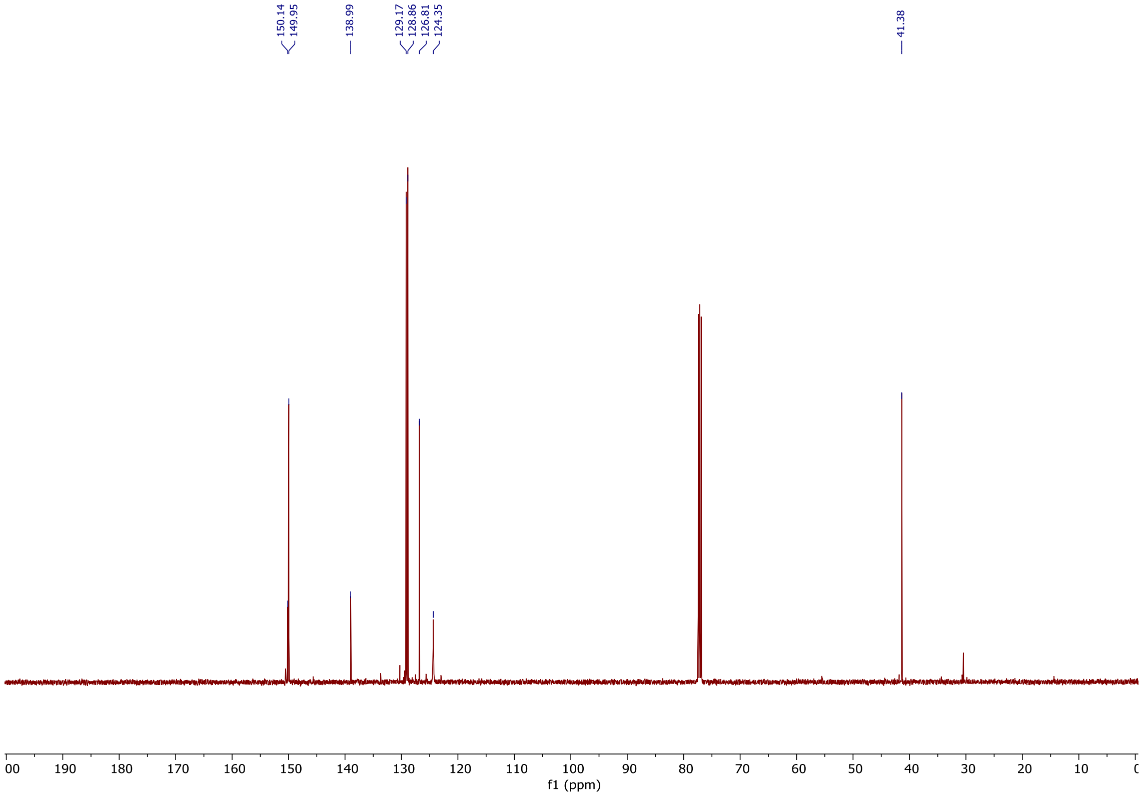
150.14
149.95
138.99
129.17
128.86
126.81
124.35
41.38
00 190 180 170 160 150 140 130 120 110 100 90 80 70 60 50 40 30 20 10 0
f1 (ppm)

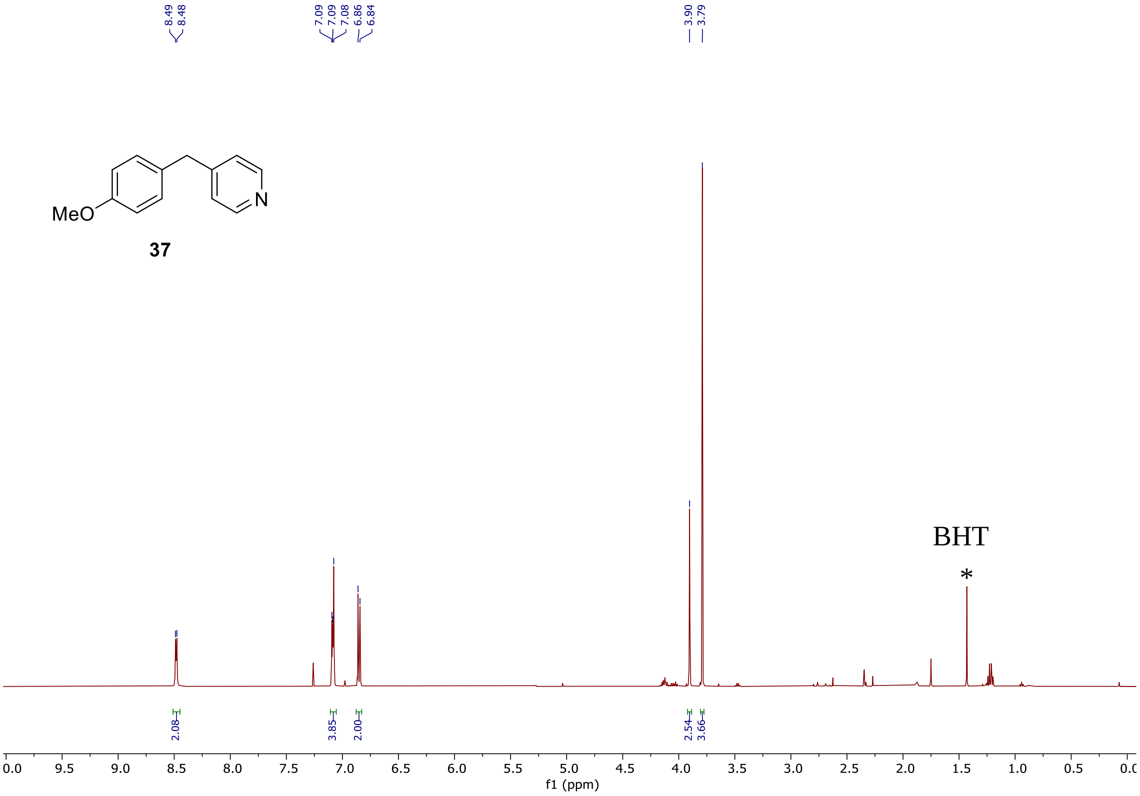

8.49
8.48
7.09
7.09
7.08
6.86
6.84
3.90
3.79
MeO
N
37
BHT
*
2.08
3.85
2.00
2.54
3.66
f1 (ppm)

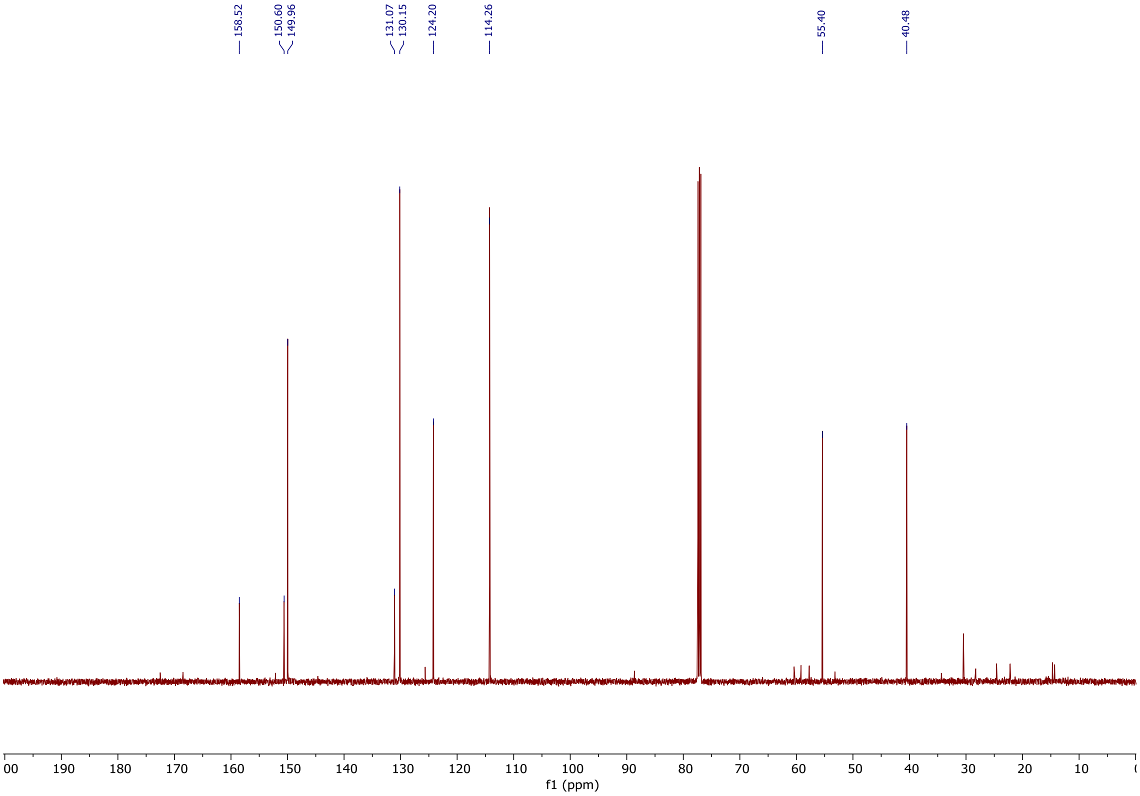

158.52
150.60
149.96
131.07
130.15
124.20
114.26
55.40
40.48
f1 (ppm)

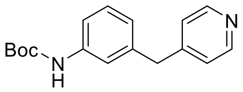
Boc
N
H
N

38

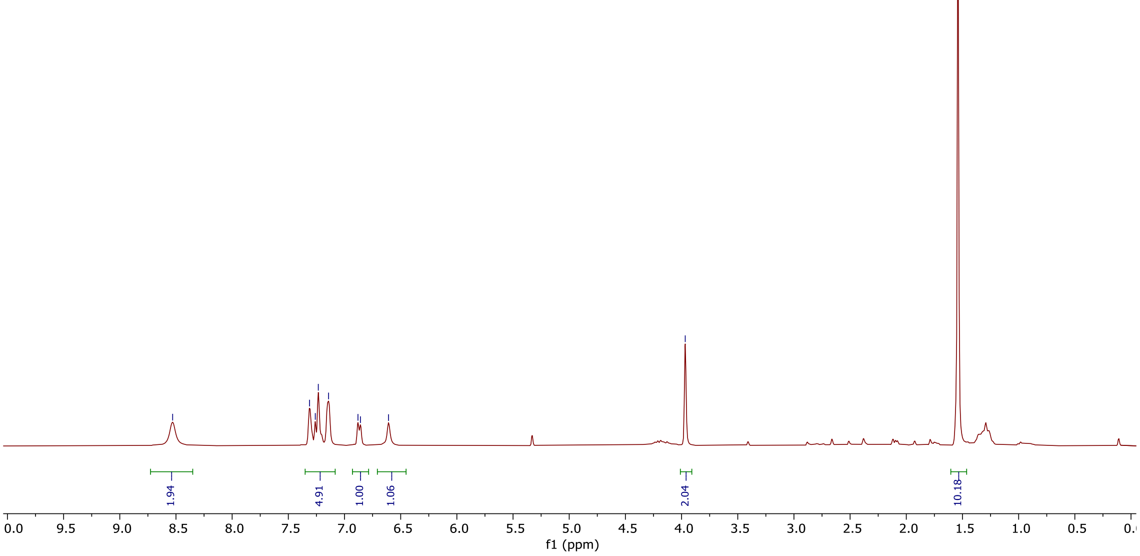
8.53
7.31
7.26
7.23
7.14
6.88
6.86
6.61
3.97
1.54
1.54
1.94
4.91
1.00
1.06
2.04
10.18
f1 (ppm)

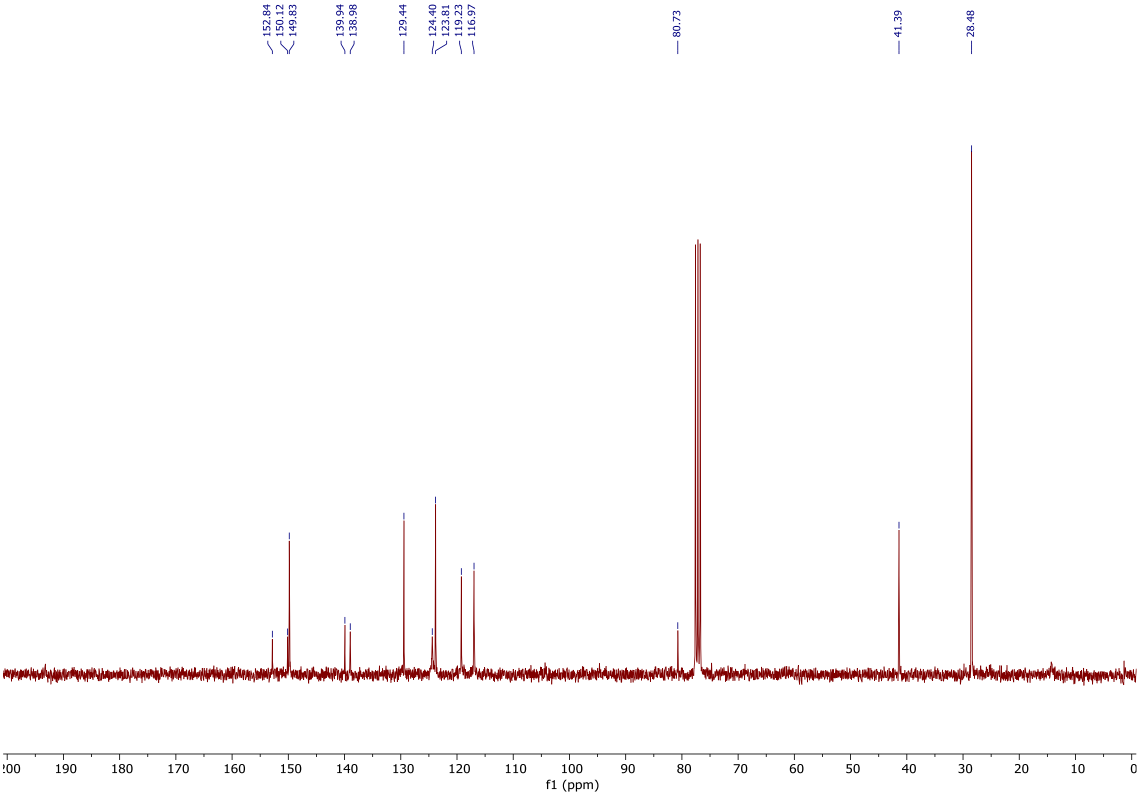
152.84
150.12
149.83
139.94
138.98
129.44
124.40
123.81
119.23
116.97
80.73
41.39
28.48
f1 (ppm)

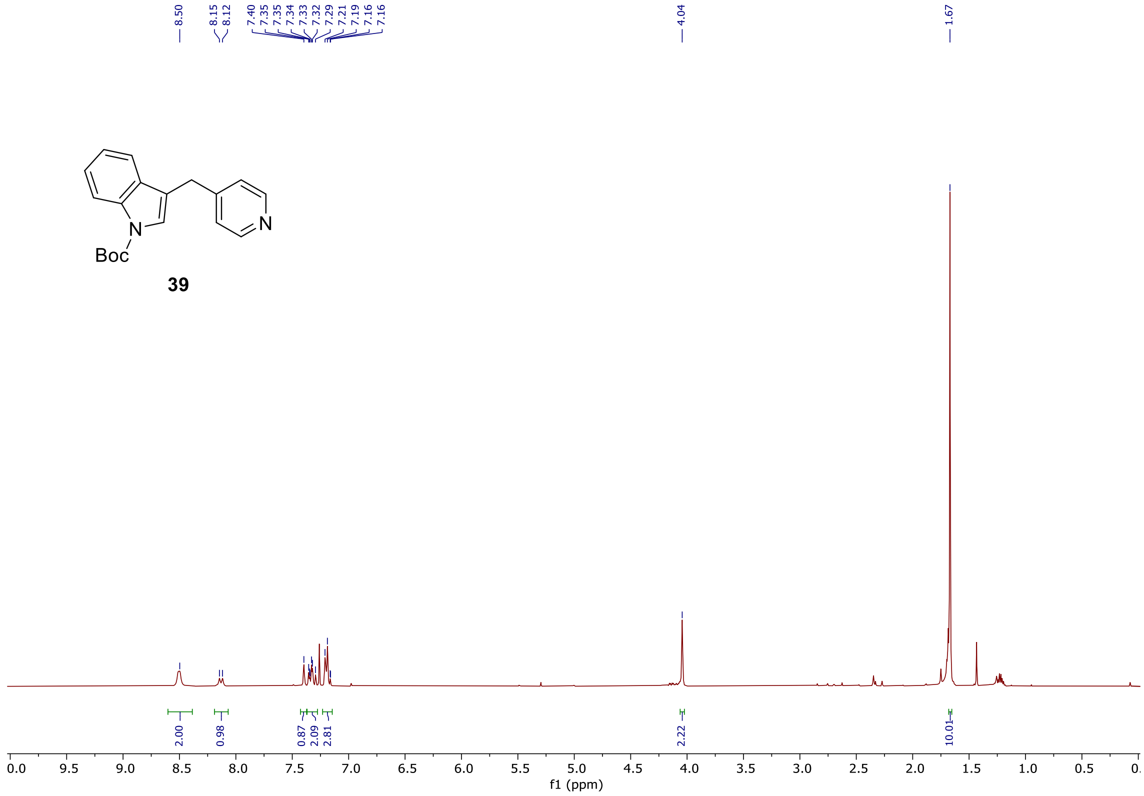
N
N
Boc
8.50
8.15
8.12
7.40
7.35
7.35
7.34
7.33
7.32
7.29
7.21
7.19
7.16
7.16
4.04
1.67
2.00
0.98
0.87
2.09
2.81
2.22
10.01
f1 (ppm)

39

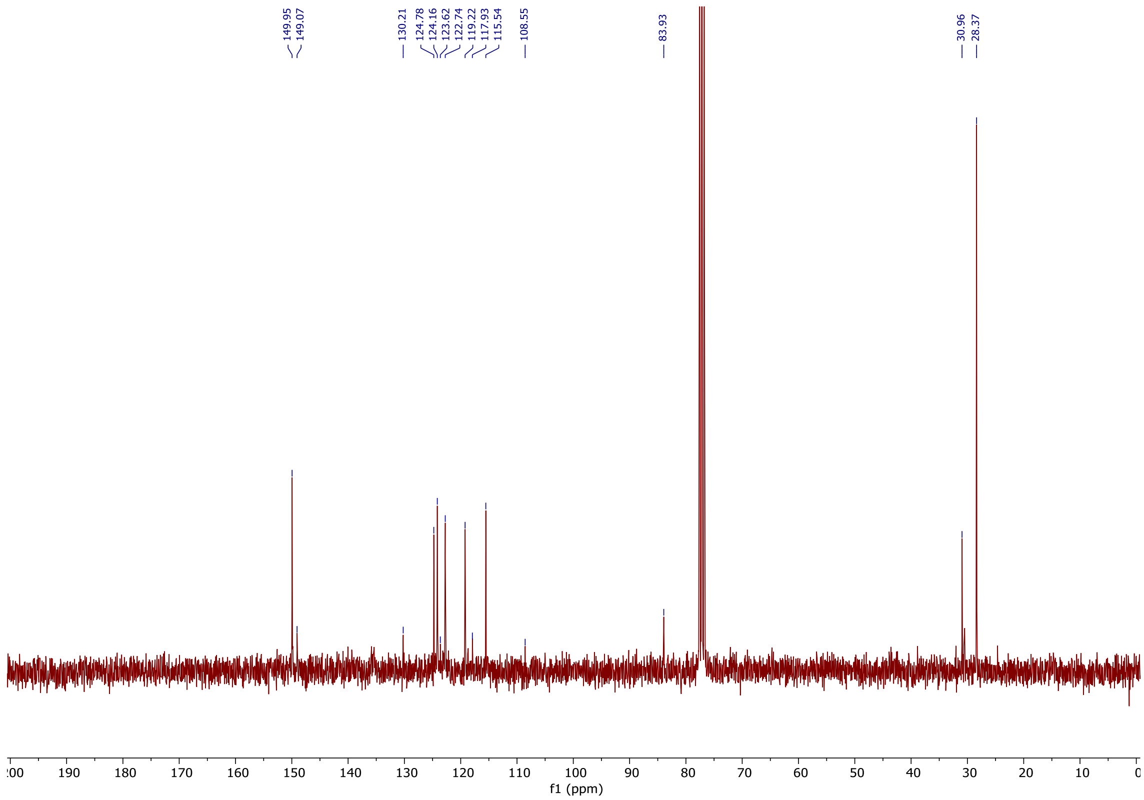
149.95
149.07
130.21
124.78
124.16
123.62
122.74
119.22
117.93
115.54
108.55
83.93
30.96
28.37
f1 (ppm)

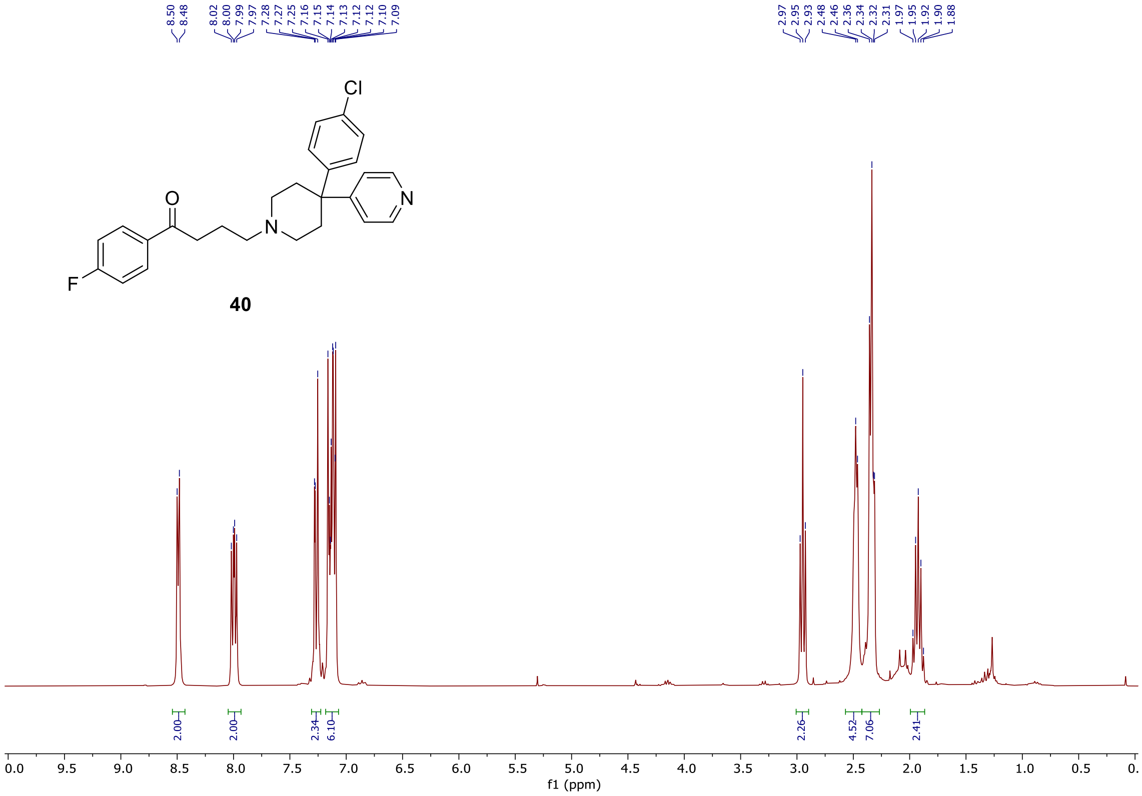
8.50
8.48
8.02
8.00
7.99
7.97
7.28
7.27
7.25
7.16
7.15
7.14
7.13
7.12
7.12
7.10
7.09
2.97
2.95
2.93
2.48
2.46
2.36
2.34
2.32
2.31
1.97
1.95
1.92
1.90
1.88
Cl
O
N
N
F
40
2.00
2.00
2.34
6.10
2.26
4.52
7.06
2.41
f1 (ppm)

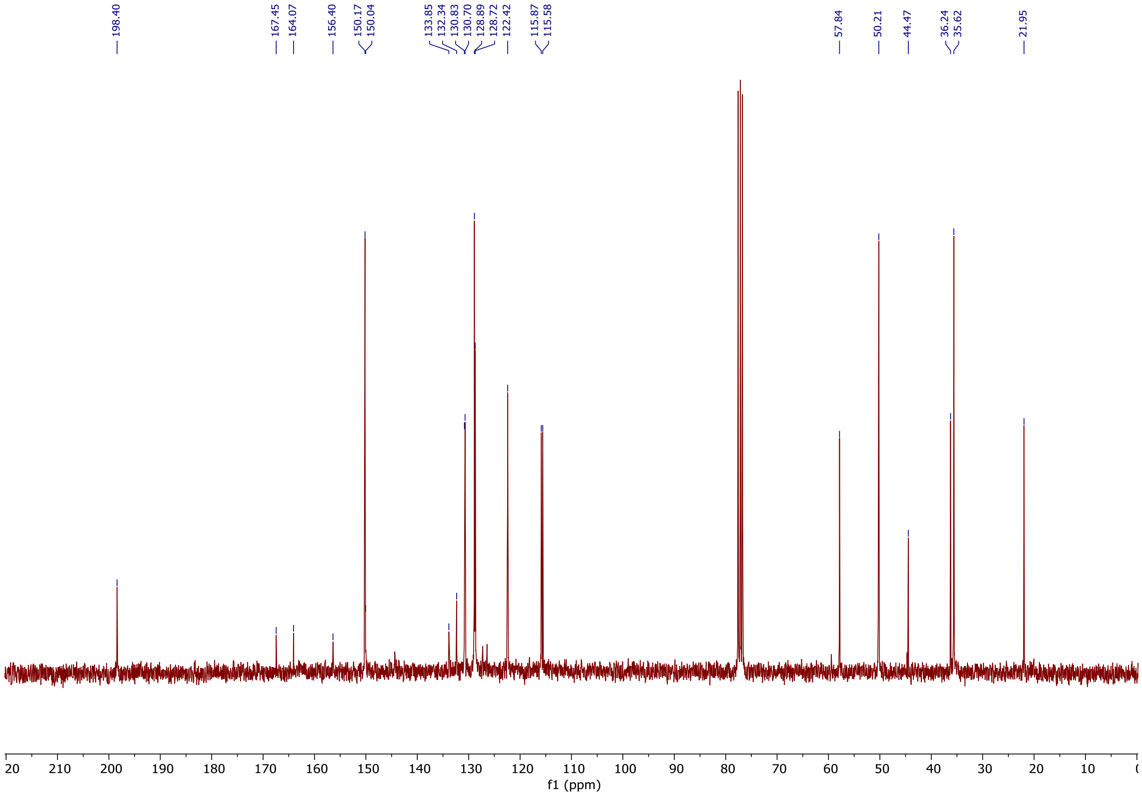
198.40
167.45
164.07
156.40
150.17
150.04
133.85
132.34
130.83
130.70
128.89
128.72
122.42
115.87
115.58
57.84
50.21
44.47
36.24
35.62
21.95
f1 (ppm)

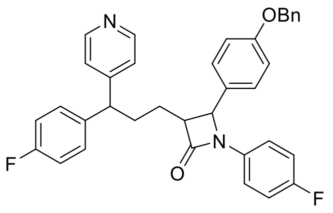
OBn
N
F
O
F

41

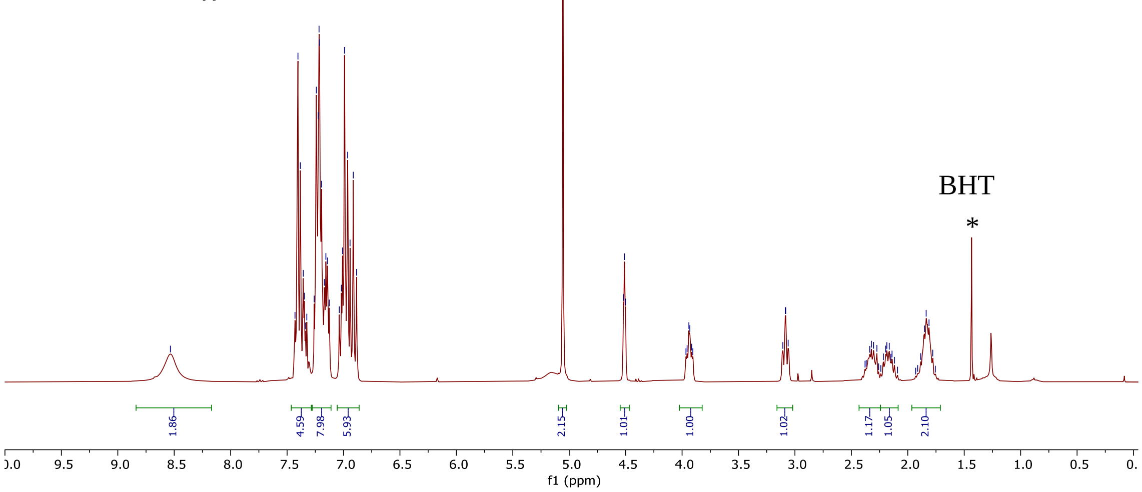
8.53
7.43
7.40
7.38
7.36
7.35
7.34
7.33
7.26
7.24
7.22
7.22
7.21
7.19
7.17
7.16
7.14
7.13
7.04
7.02
7.01
6.99
6.96
6.94
6.91
6.88
5.06
4.52
4.51
4.50
3.97
3.96
3.94
3.93
3.91
3.90
3.11
3.09
3.08
3.06
2.38
2.37
2.34
2.32
2.30
2.27
2.26
2.24
2.22
2.20
2.19
2.16
2.15
2.14
2.12
2.09
1.93
1.91
1.89
1.85
1.84
1.81
1.78
1.76
BHT
*
1.86
4.59
7.98
5.93
2.15
1.01
1.00
1.02
1.17
1.05
2.10
f1 (ppm)

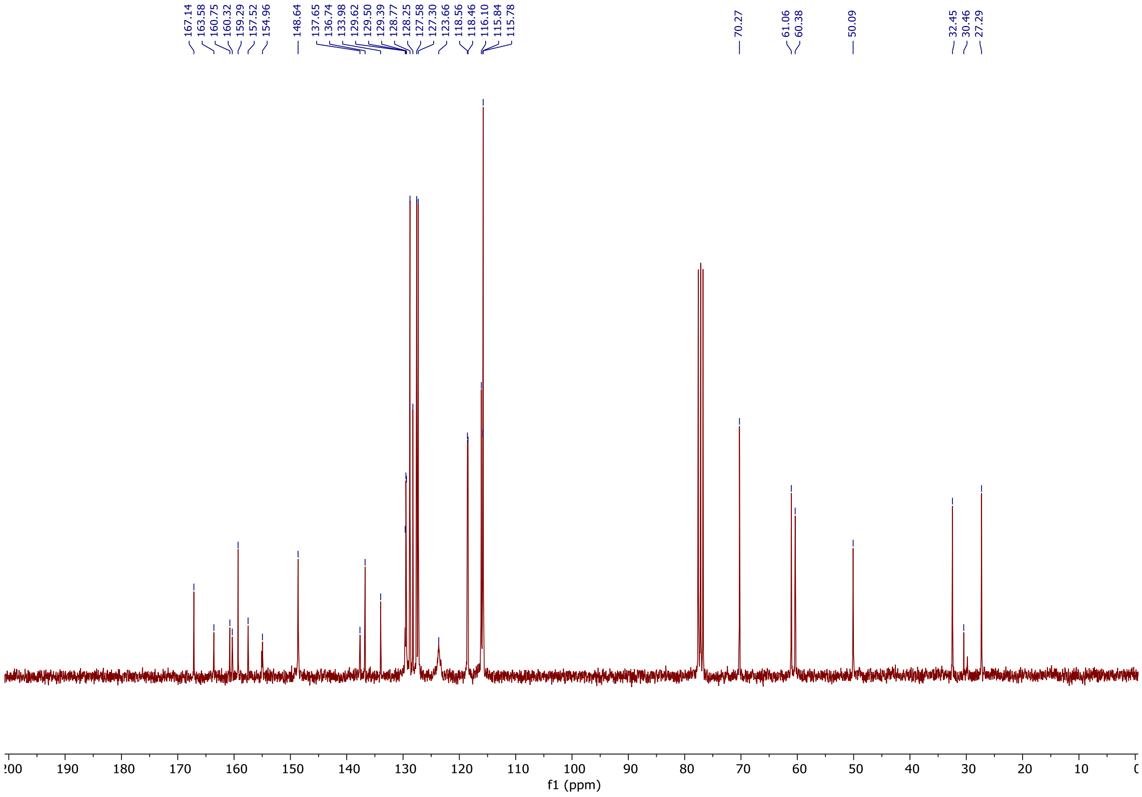
167.14
163.58
160.75
160.32
159.29
157.52
154.96
148.64
137.65
136.74
133.98
129.62
129.50
129.39
128.77
128.25
127.58
127.30
123.66
118.56
118.46
116.10
115.84
115.78
70.27
61.06
60.38
50.09
32.45
30.46
27.29
f1 (ppm)

42 (*trans:cis* 5.3:1)

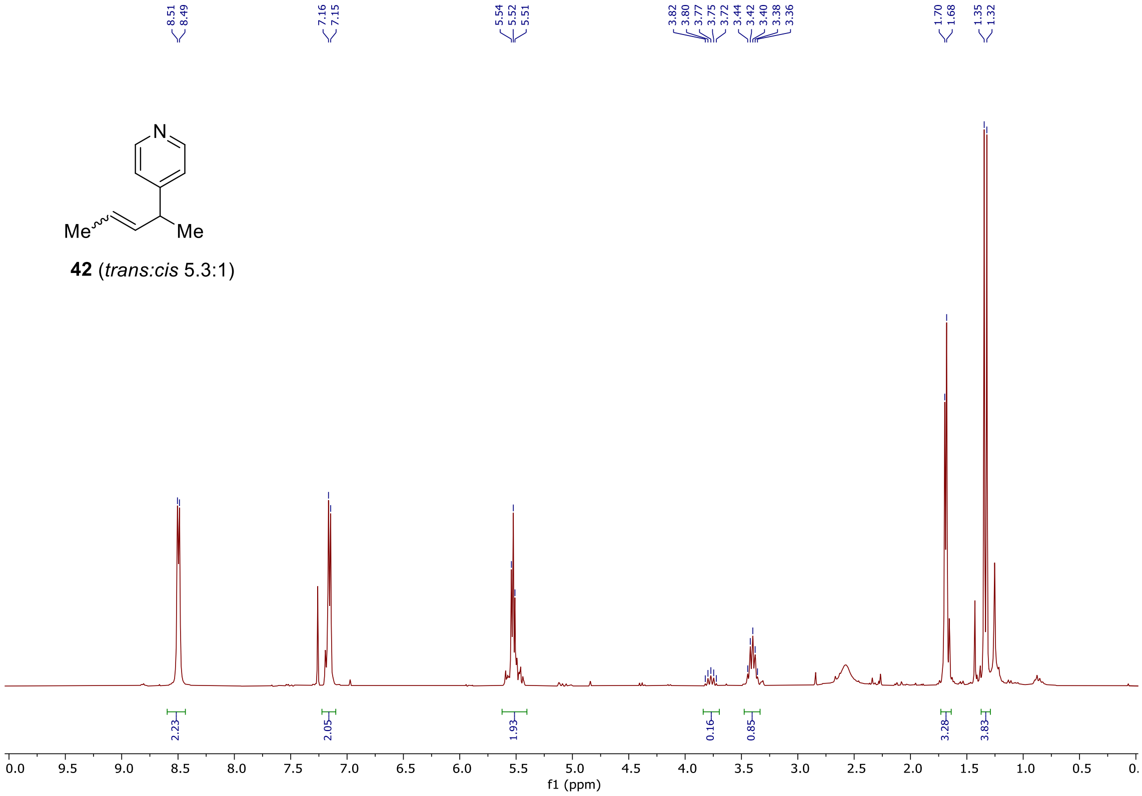

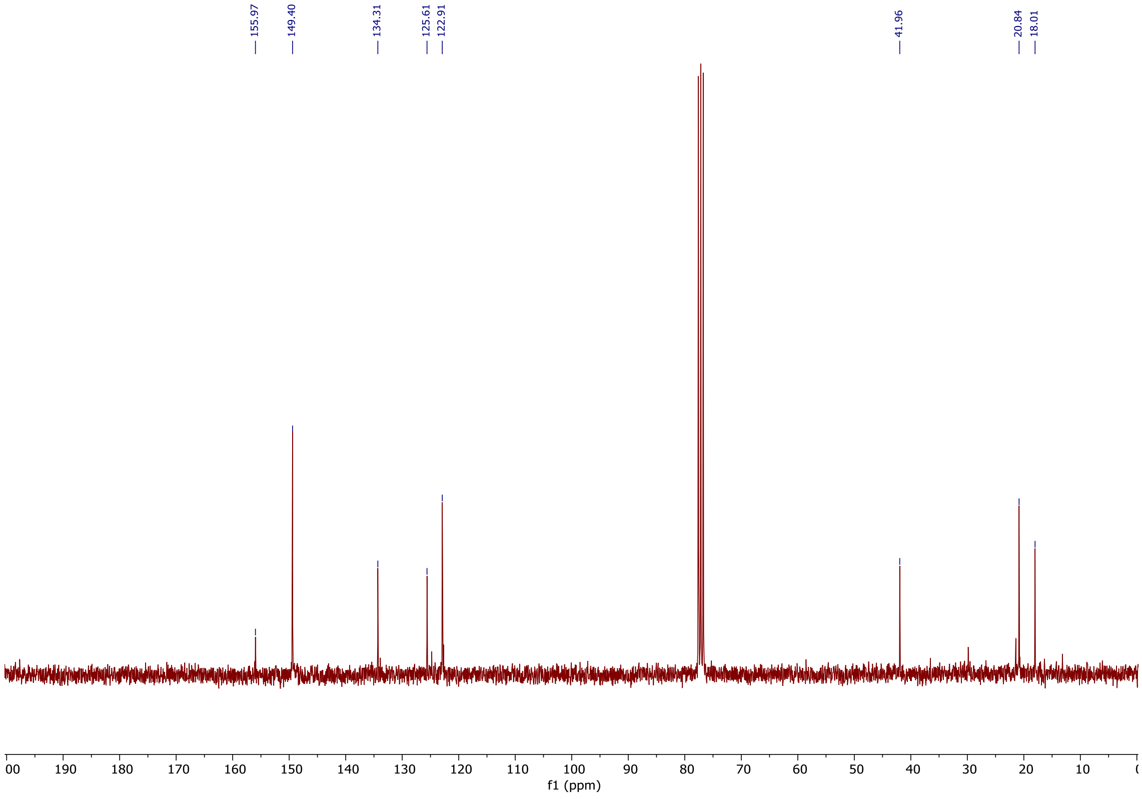

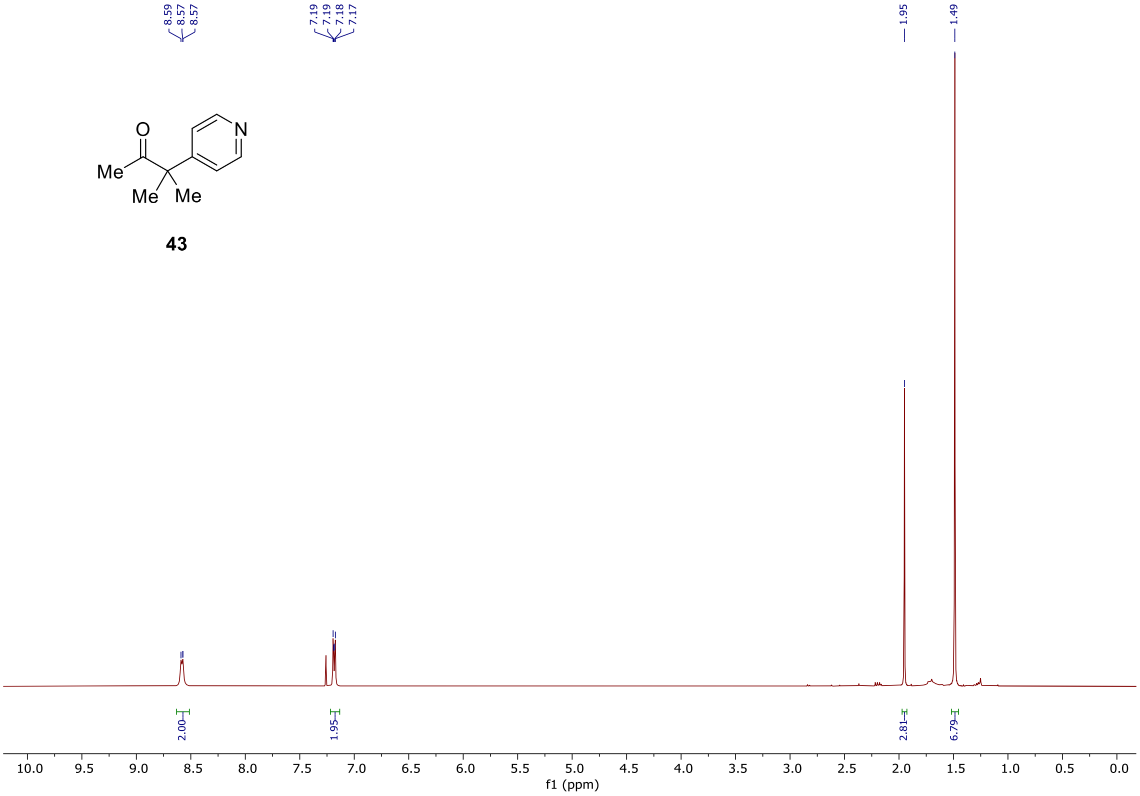

8.59
8.57
8.57
7.19
7.19
7.18
7.17
1.95
1.49
O
N
Me
Me
Me
2.00
1.95
2.81
6.79
10.0 9.5 9.0 8.5 8.0 7.5 7.0 6.5 6.0 5.5 5.0 4.5 4.0 3.5 3.0 2.5 2.0 1.5 1.0 0.5 0.0
f1 (ppm)

43

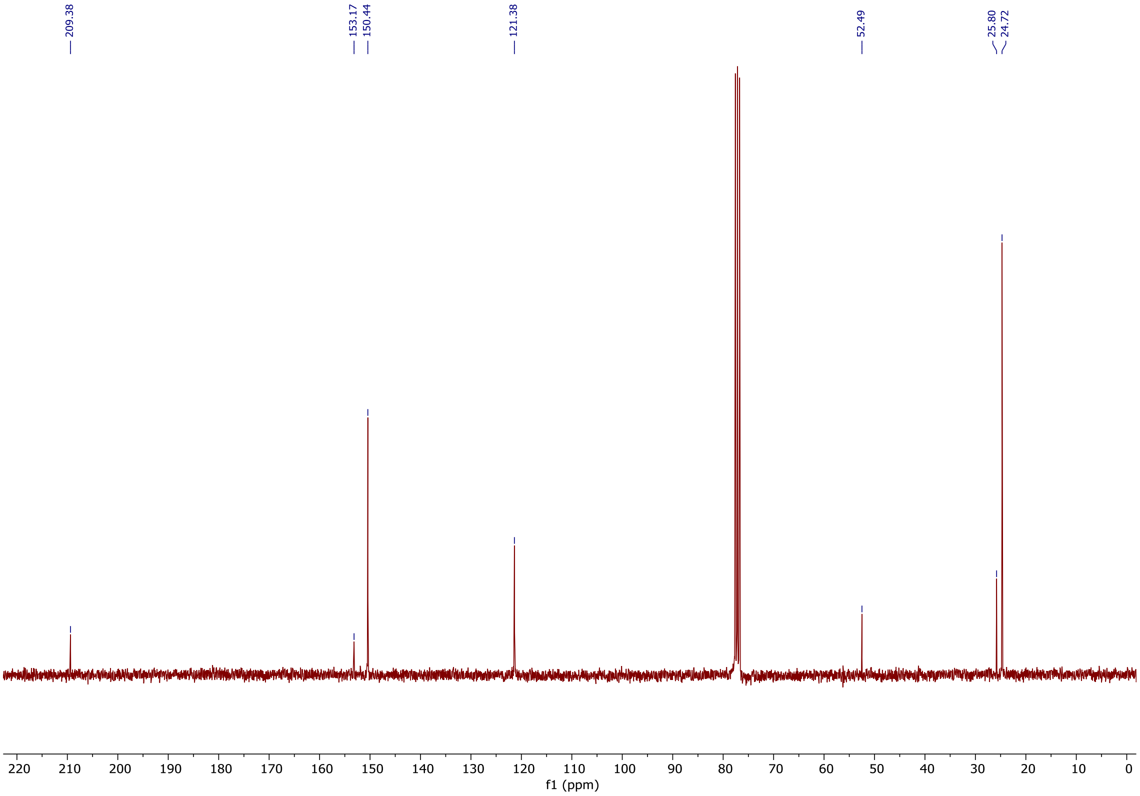

209.38
153.17
150.44
121.38
52.49
25.80
24.72
220 210 200 190 180 170 160 150 140 130 120 110 100 90 80 70 60 50 40 30 20 10 0
f1 (ppm)

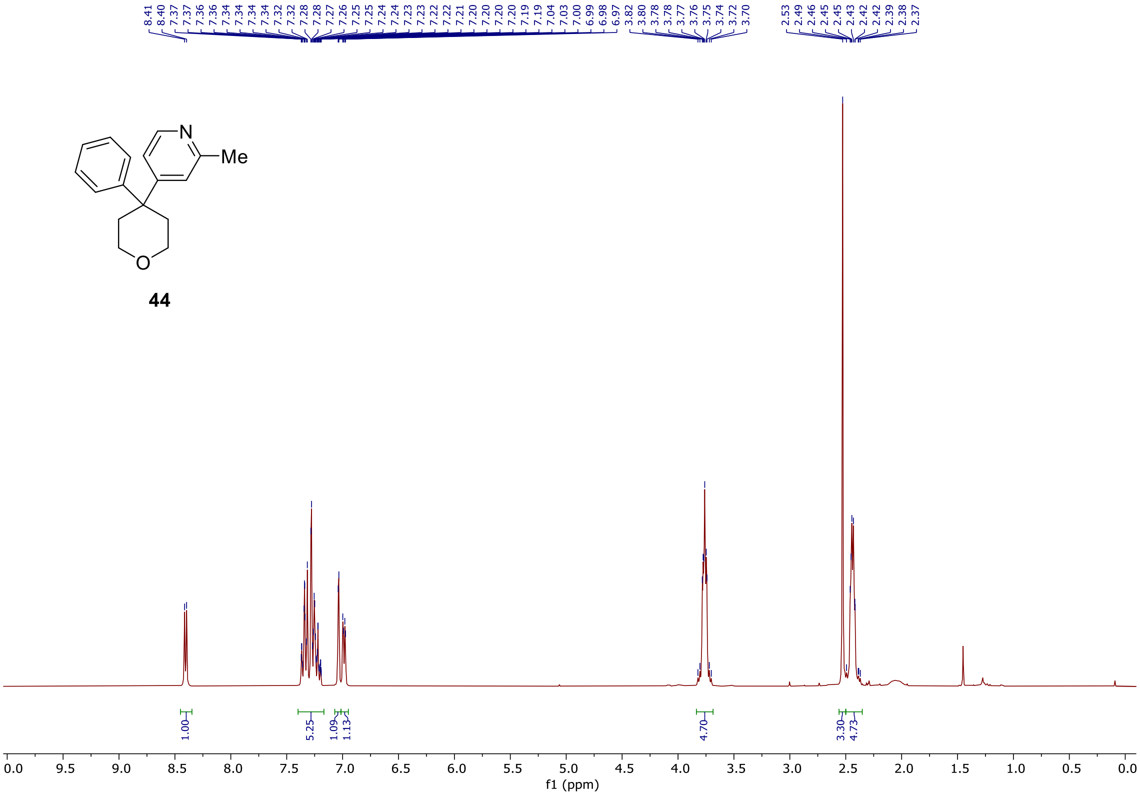
8.41
8.40
7.37
7.37
7.36
7.34
7.34
7.34
7.32
7.32
7.28
7.28
7.27
7.26
7.25
7.24
7.24
7.23
7.23
7.22
7.22
7.21
7.20
7.20
7.20
7.19
7.19
7.04
7.03
7.00
6.99
6.98
6.97
3.82
3.80
3.78
3.77
3.76
3.75
3.74
3.72
3.70
2.53
2.49
2.46
2.45
2.45
2.43
2.42
2.42
2.39
2.38
2.37
N
Me
O
44
1.00
5.25
1.09
1.13
4.70
3.30
4.73
f1 (ppm)

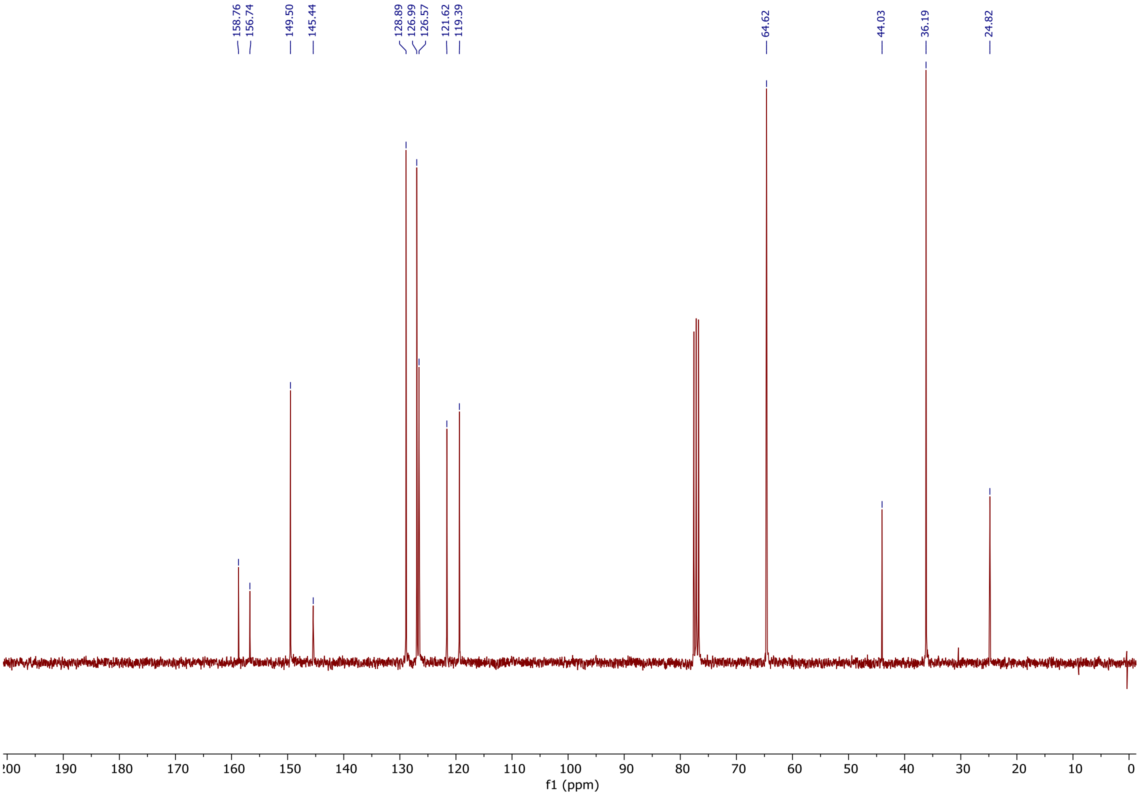
158.76
156.74
149.50
145.44
128.89
126.99
126.57
121.62
119.39
64.62
44.03
36.19
24.82
f1 (ppm)

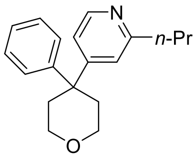
N
n-Pr
O

45

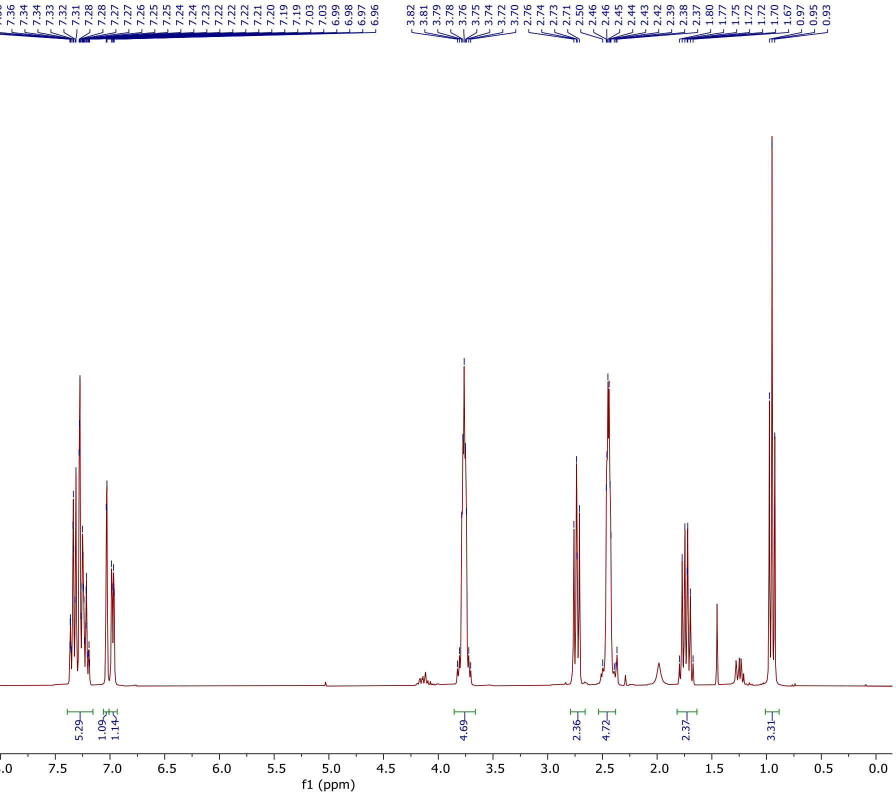
8.44
8.42
7.37
7.36
7.36
7.34
7.34
7.33
7.32
7.31
7.28
7.28
7.27
7.27
7.26
7.25
7.25
7.24
7.24
7.23
7.22
7.22
7.22
7.21
7.20
7.19
7.19
7.03
7.03
6.99
6.98
6.97
6.96
3.82
3.81
3.79
3.78
3.76
3.75
3.74
3.72
3.70
2.76
2.74
2.73
2.71
2.50
2.46
2.46
2.45
2.44
2.43
2.42
2.39
2.38
2.37
1.80
1.77
1.75
1.72
1.72
1.70
1.67
0.97
0.95
0.93
1.00
5.29
1.09
1.14
4.69
2.36
4.72
2.37
3.31
10.0
9.5
9.0
8.5
8.0
7.5
7.0
6.5
6.0
5.5
5.0
4.5
4.0
3.5
3.0
2.5
2.0
1.5
1.0
0.5
0.0
f1 (ppm)

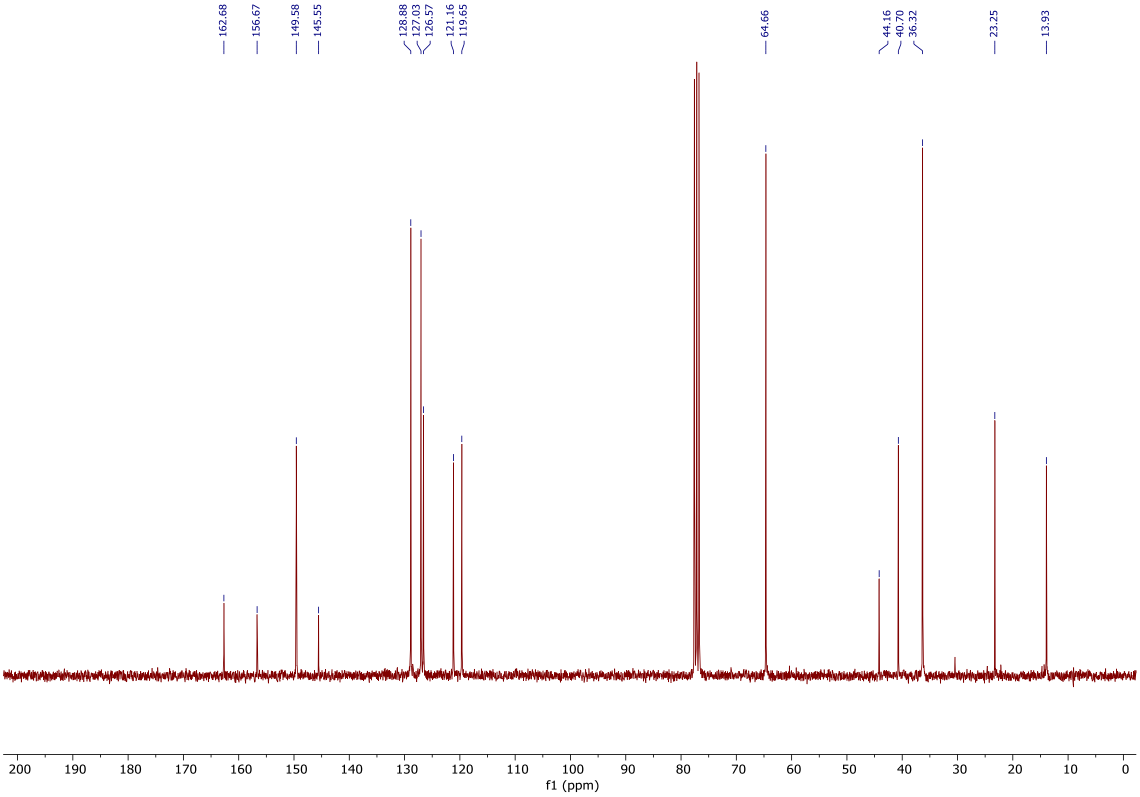
162.68
156.67
149.58
145.55
128.88
127.03
126.57
121.16
119.65
64.66
44.16
40.70
36.32
23.25
13.93
200
190
180
170
160
150
140
130
120
110
100
90
80
70
60
50
40
30
20
10
0
f1 (ppm)

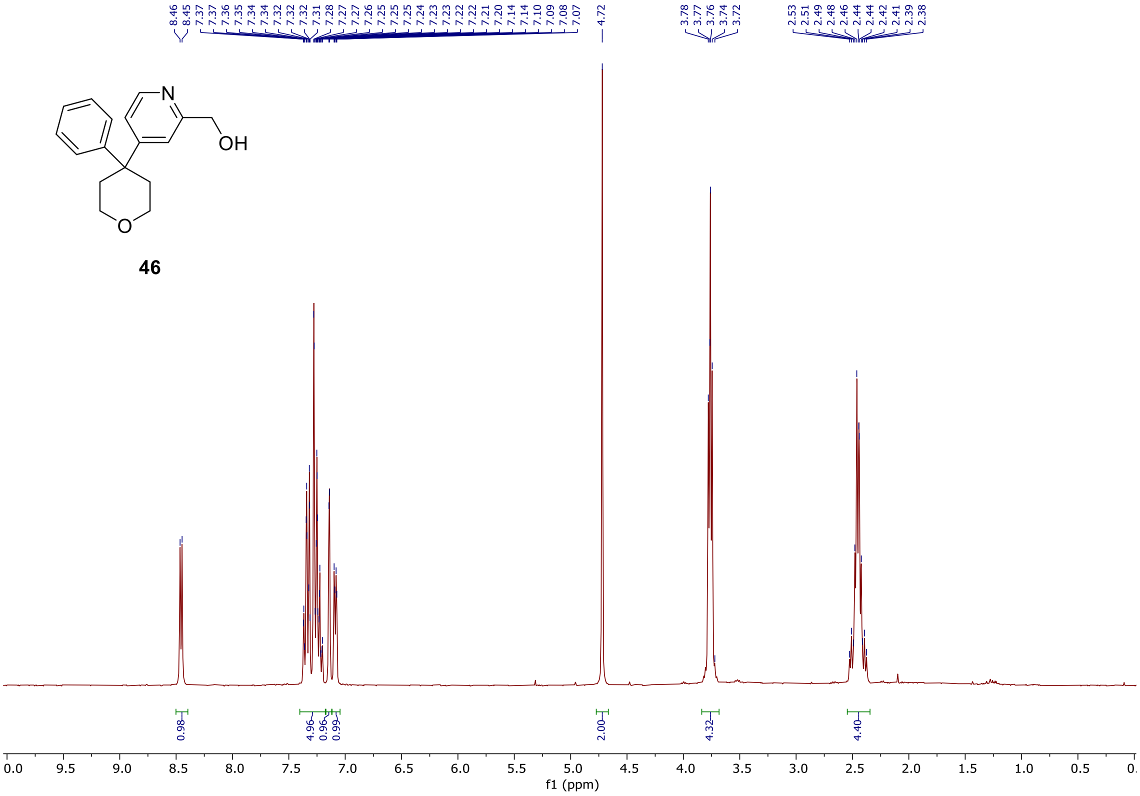
N
OH
O
46
8.46
8.45
7.37
7.37
7.36
7.35
7.34
7.34
7.34
7.32
7.32
7.32
7.32
7.31
7.28
7.27
7.27
7.26
7.25
7.25
7.25
7.24
7.24
7.23
7.23
7.22
7.22
7.21
7.21
7.20
7.14
7.14
7.10
7.09
7.08
7.07
4.72
3.78
3.77
3.76
3.74
3.72
2.53
2.51
2.49
2.48
2.46
2.44
2.44
2.42
2.41
2.39
2.38
0.98
4.96
0.96
0.99
2.00
4.32
4.40
f1 (ppm)

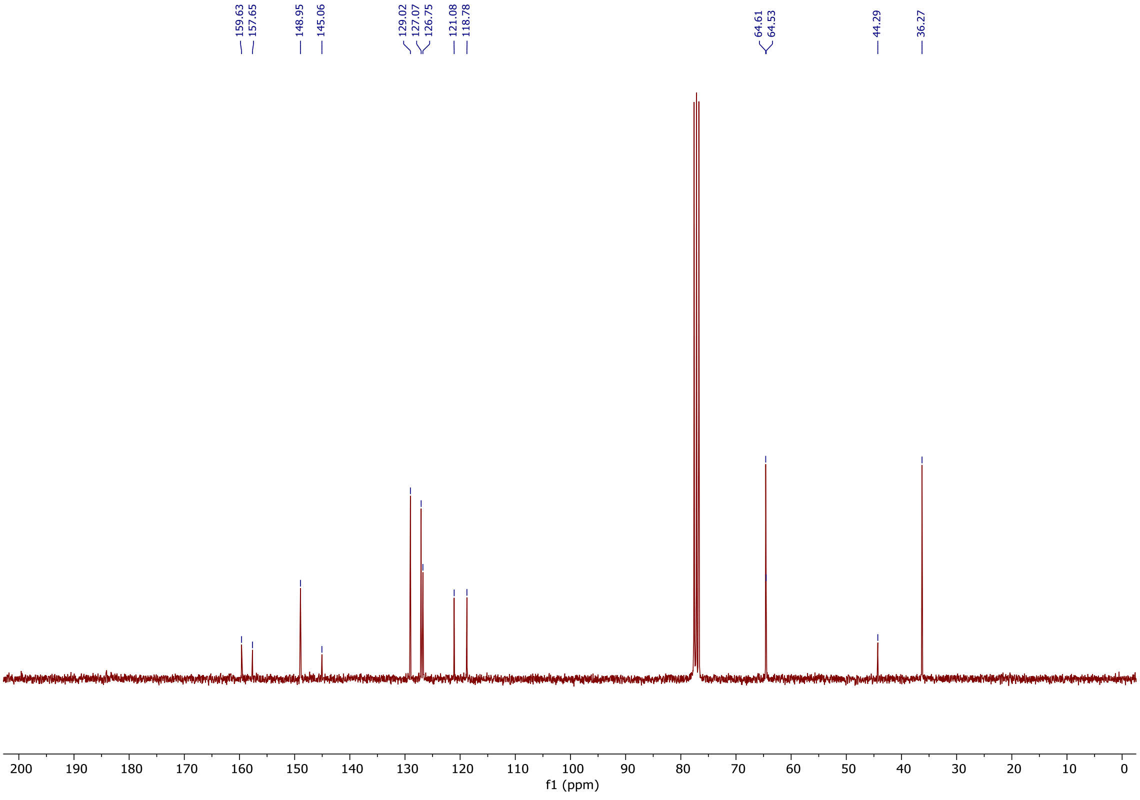
159.63
157.65
148.95
145.06
129.02
127.07
126.75
121.08
118.78
64.61
64.53
44.29
36.27
f1 (ppm)

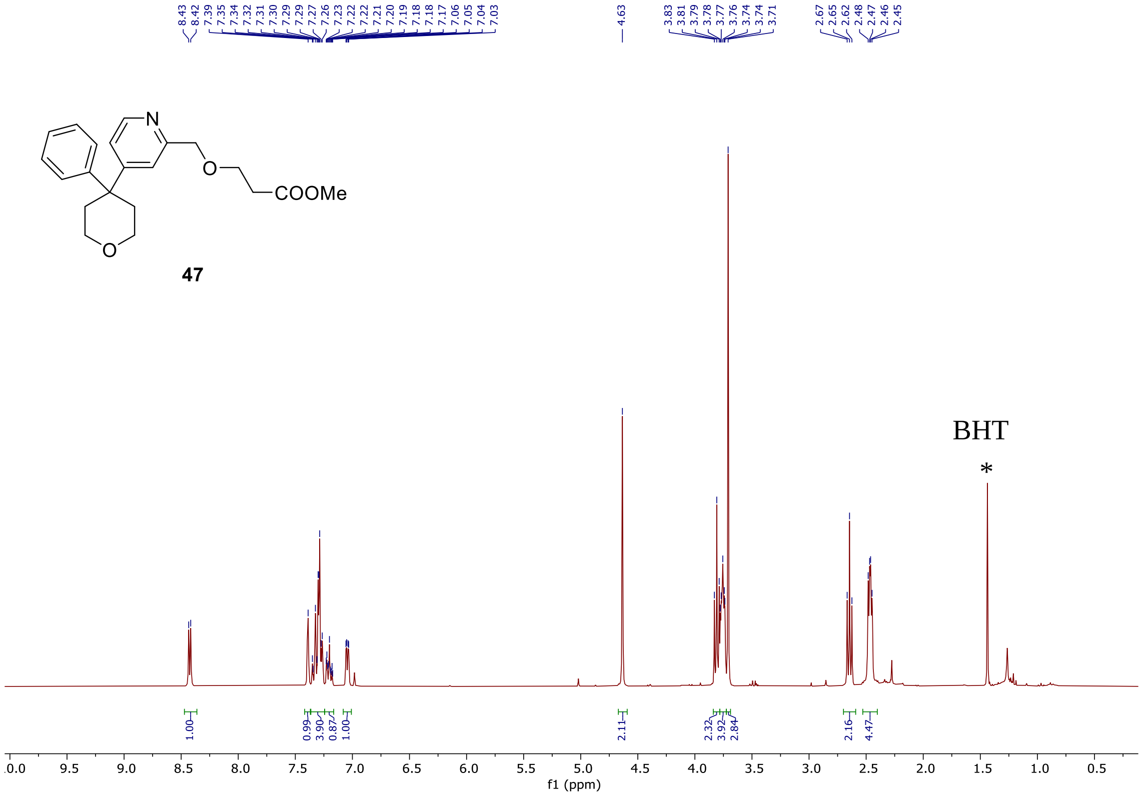

47
COOMe
BHT
f1 (ppm)

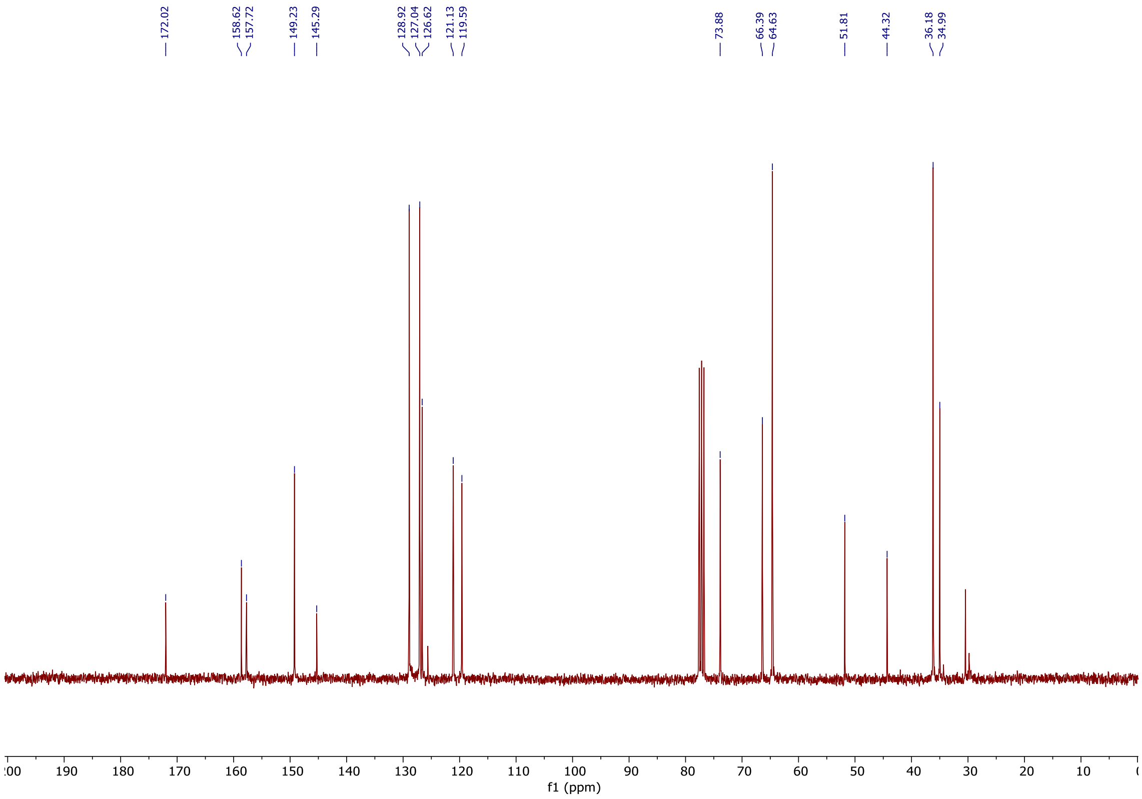

f1 (ppm)

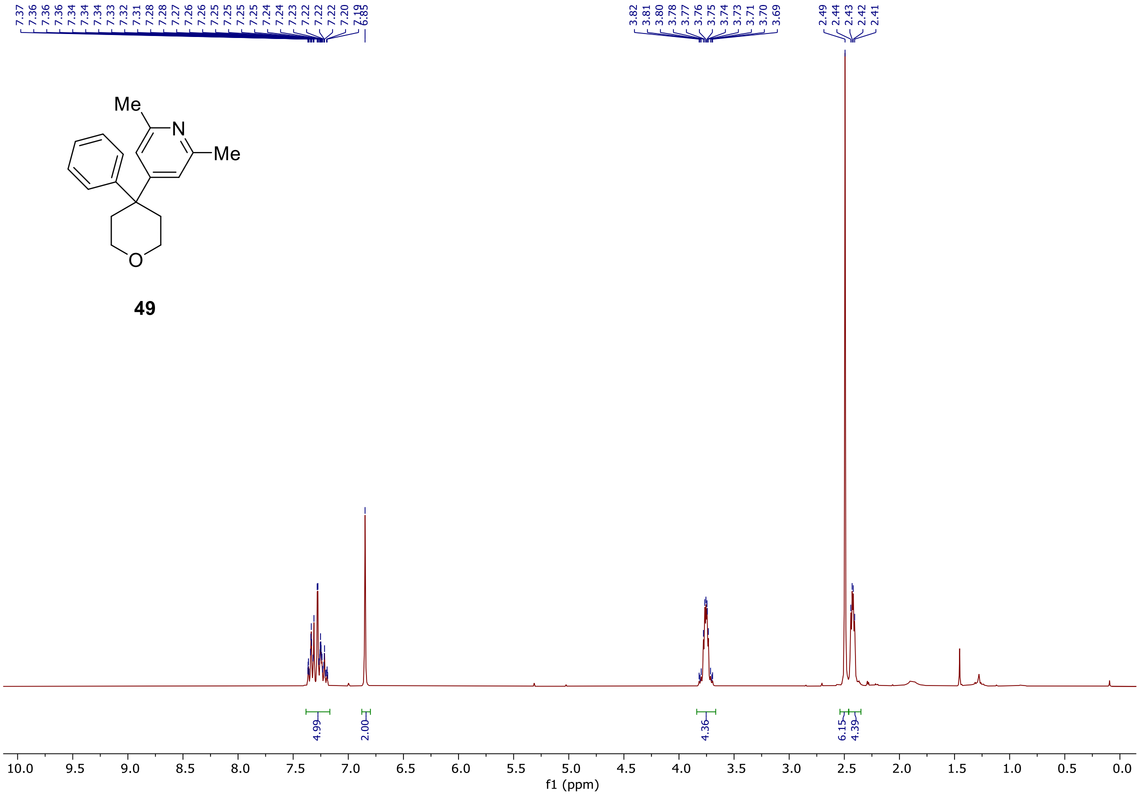
7.37
7.36
7.36
7.34
7.34
7.34
7.33
7.32
7.31
7.28
7.28
7.27
7.26
7.26
7.25
7.25
7.25
7.24
7.24
7.23
7.22
7.22
7.22
7.20
7.19
6.85
3.82
3.81
3.80
3.78
3.77
3.76
3.75
3.74
3.73
3.71
3.70
3.69
2.49
2.44
2.43
2.42
2.41
Me
N
Me
O
49
4.99
2.00
4.36
6.15
4.39
f1 (ppm)

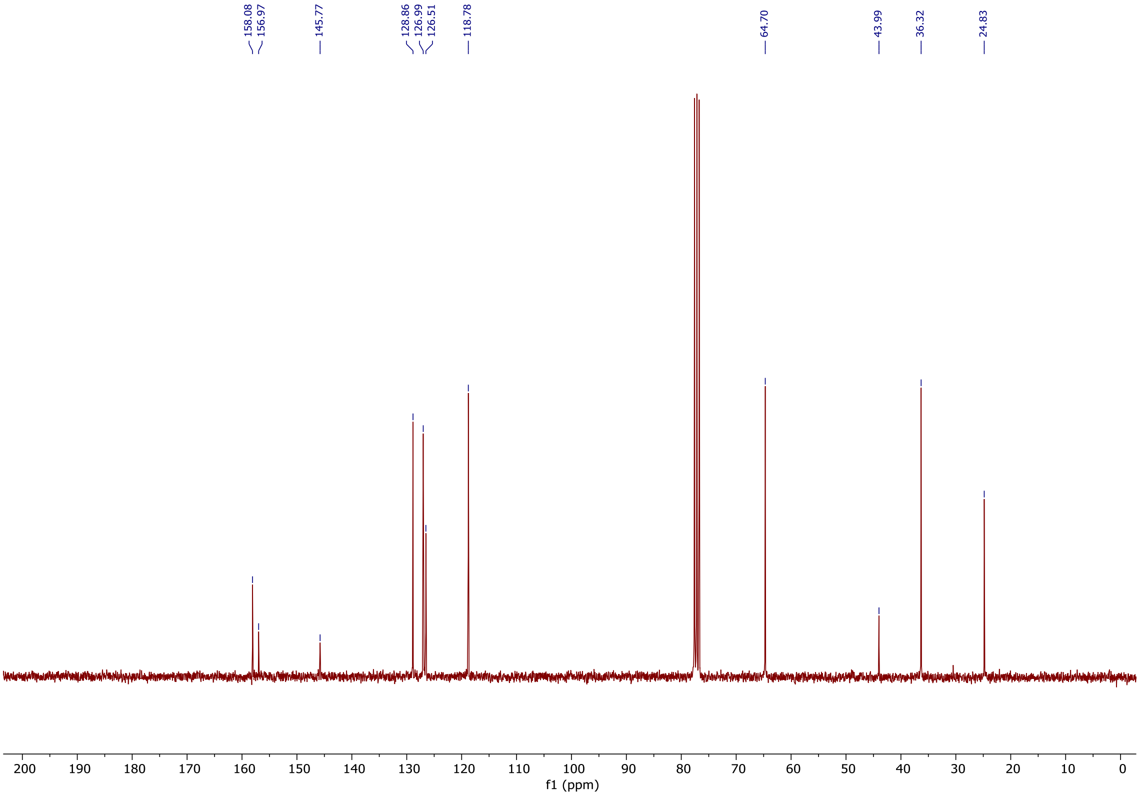
158.08
156.97
145.77
128.86
126.99
126.51
118.78
64.70
43.99
36.32
24.83
f1 (ppm)

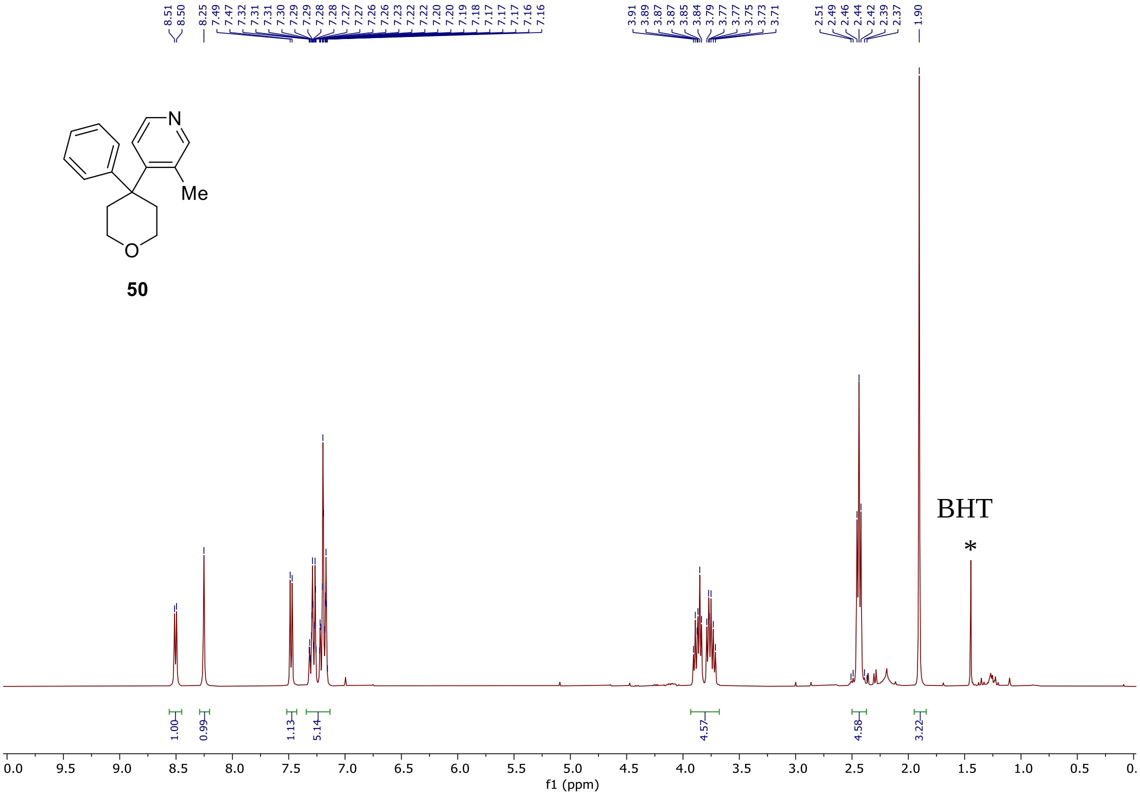

50

8.51
8.50
8.25
7.49
7.47
7.32
7.31
7.31
7.30
7.29
7.29
7.28
7.28
7.27
7.27
7.26
7.26
7.23
7.22
7.22
7.20
7.20
7.19
7.18
7.17
7.17
7.17
7.16
7.16
3.91
3.89
3.87
3.87
3.85
3.84
3.79
3.77
3.77
3.75
3.73
3.71
2.51
2.49
2.46
2.44
2.42
2.39
2.37
1.90
BHT
*
1.00
0.99
1.13
5.14
4.57
4.58
3.22
0.0
9.5
9.0
8.5
8.0
7.5
7.0
6.5
6.0
5.5
5.0
4.5
4.0
3.5
3.0
2.5
2.0
1.5
1.0
0.5
0.
f1 (ppm)

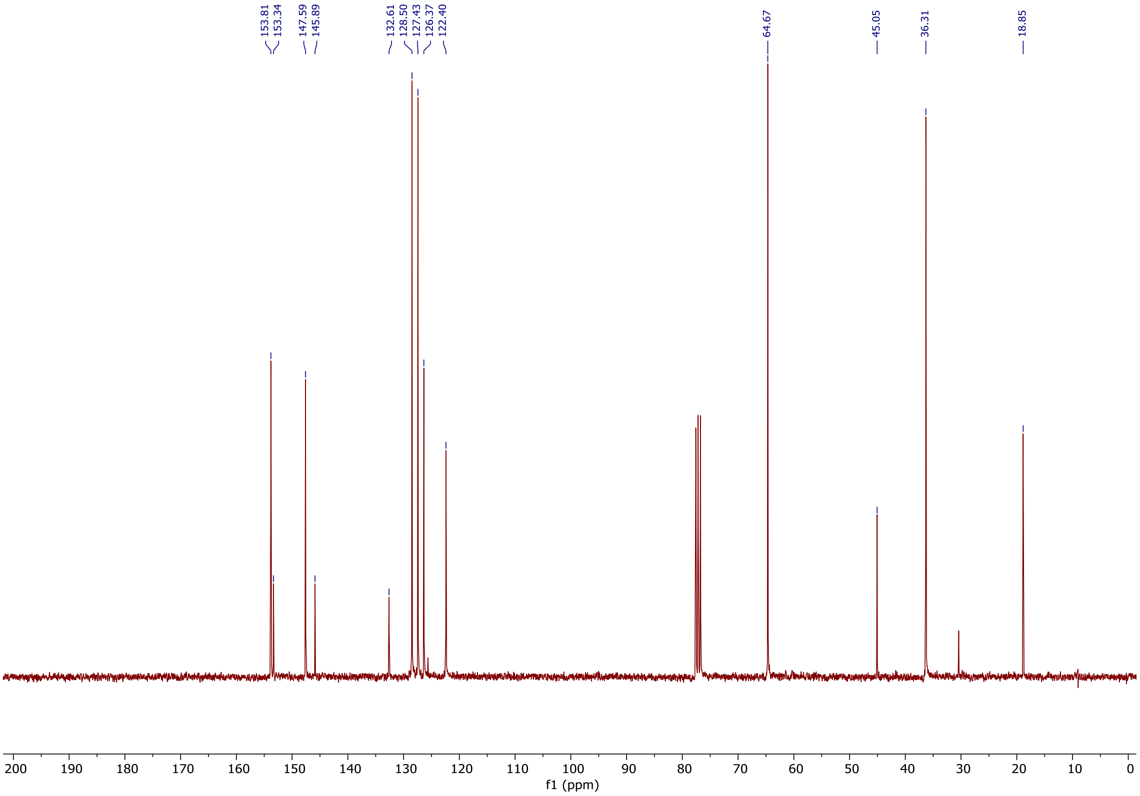

153.81
153.34
147.59
145.89
132.61
128.50
127.43
126.37
122.40
64.67
45.05
36.31
18.85
200
190
180
170
160
150
140
130
120
110
100
90
80
70
60
50
40
30
20
10
0
f1 (ppm)

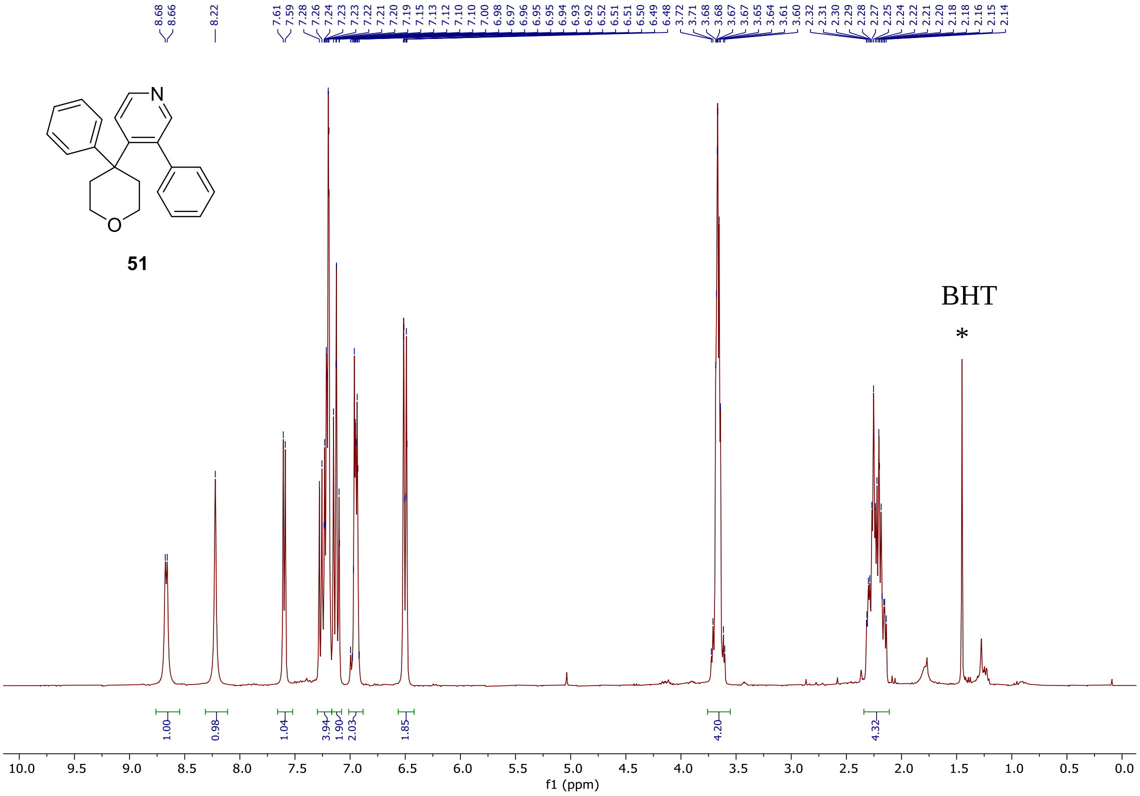

8.68
8.66
8.22
7.61
7.59
7.28
7.26
7.24
7.23
7.23
7.22
7.21
7.20
7.19
7.15
7.13
7.12
7.12
7.10
7.10
7.00
6.98
6.97
6.96
6.95
6.95
6.94
6.93
6.92
6.52
6.51
6.51
6.50
6.49
6.48
3.72
3.71
3.68
3.68
3.67
3.67
3.65
3.64
3.61
3.60
2.32
2.31
2.30
2.29
2.28
2.27
2.25
2.24
2.22
2.21
2.20
2.18
2.16
2.15
2.14
N
O
51
BHT
*
1.00
0.98
1.04
3.94
1.90
2.03
1.85
4.20
4.32
10.0 9.5 9.0 8.5 8.0 7.5 7.0 6.5 6.0 5.5 5.0 4.5 4.0 3.5 3.0 2.5 2.0 1.5 1.0 0.5 0.0
f1 (ppm)

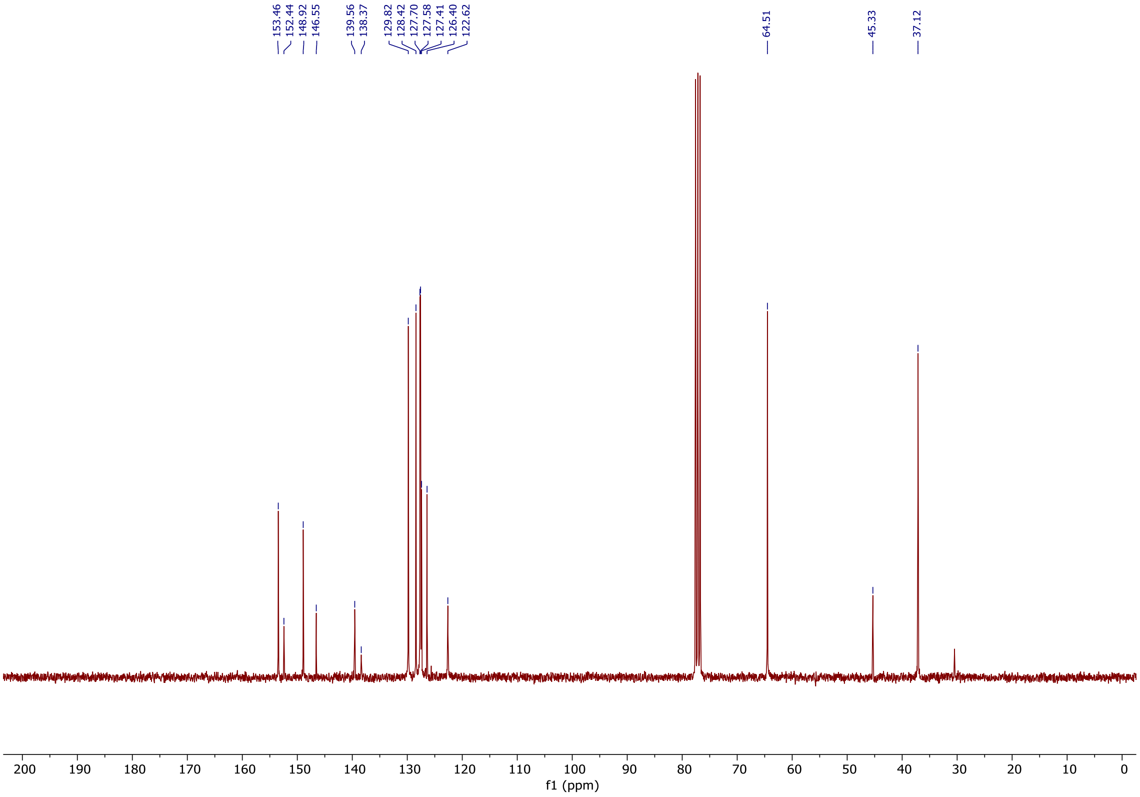

153.46
152.44
148.92
146.55
139.56
138.37
129.82
128.42
127.70
127.58
127.41
126.40
122.62
64.51
45.33
37.12
200 190 180 170 160 150 140 130 120 110 100 90 80 70 60 50 40 30 20 10 0
f1 (ppm)

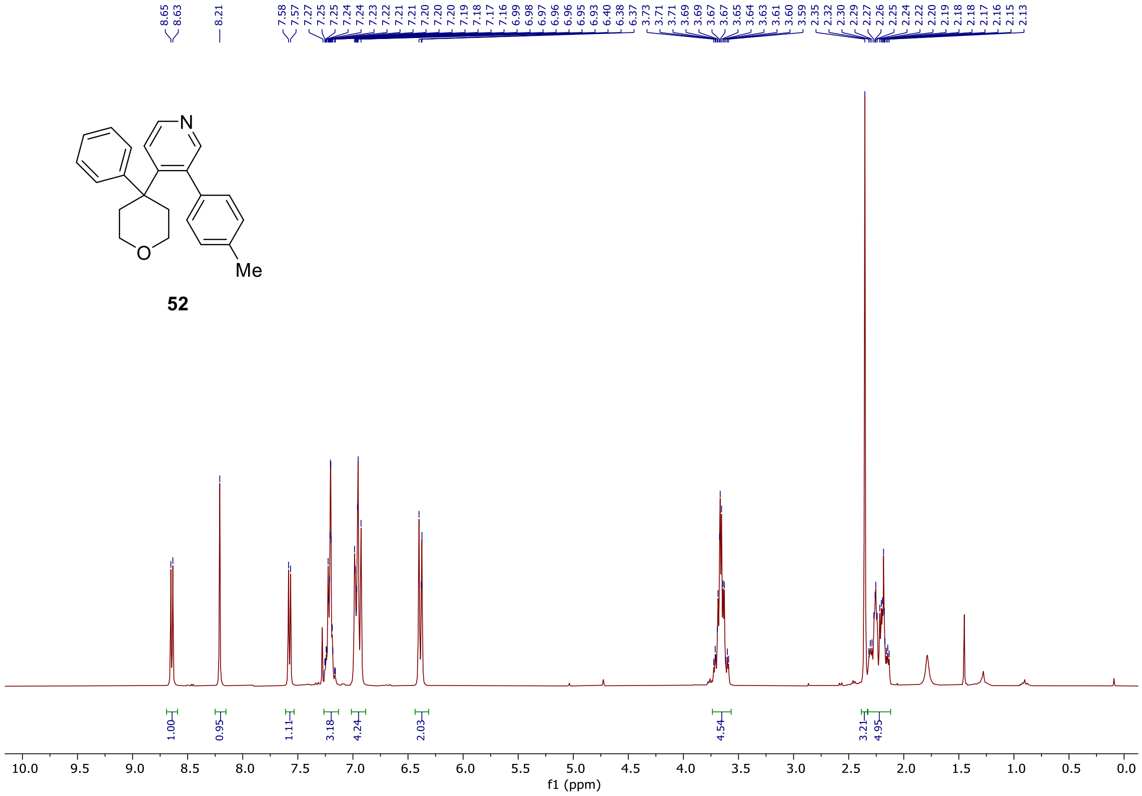
8.65
8.63
8.21
7.58
7.57
7.27
7.25
7.25
7.24
7.24
7.23
7.22
7.21
7.21
7.20
7.20
7.20
7.19
7.18
7.17
7.16
6.99
6.98
6.97
6.96
6.96
6.95
6.93
6.40
6.38
6.37
3.73
3.71
3.69
3.69
3.67
3.67
3.65
3.64
3.63
3.61
3.60
3.59
2.35
2.32
2.30
2.29
2.27
2.26
2.25
2.24
2.22
2.20
2.19
2.18
2.18
2.17
2.16
2.15
2.13
N
O
Me
52
1.00
0.95
1.11
3.18
4.24
2.03
4.54
3.21
4.95
10.0
9.5
9.0
8.5
8.0
7.5
7.0
6.5
6.0
5.5
5.0
4.5
4.0
3.5
3.0
2.5
2.0
1.5
1.0
0.5
0.0
f1 (ppm)

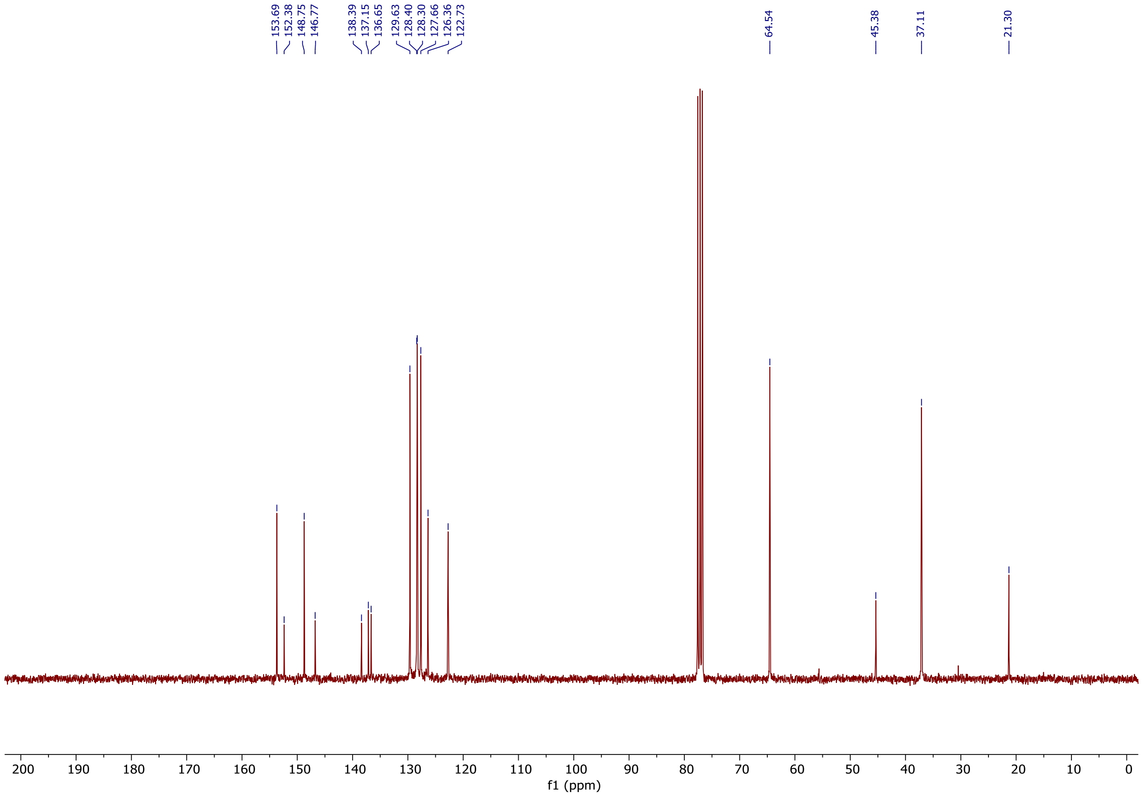
153.69
152.38
148.75
146.77
138.39
137.15
136.65
129.63
128.40
128.30
127.66
126.36
122.73
64.54
45.38
37.11
21.30
200
190
180
170
160
150
140
130
120
110
100
90
80
70
60
50
40
30
20
10
0
f1 (ppm)

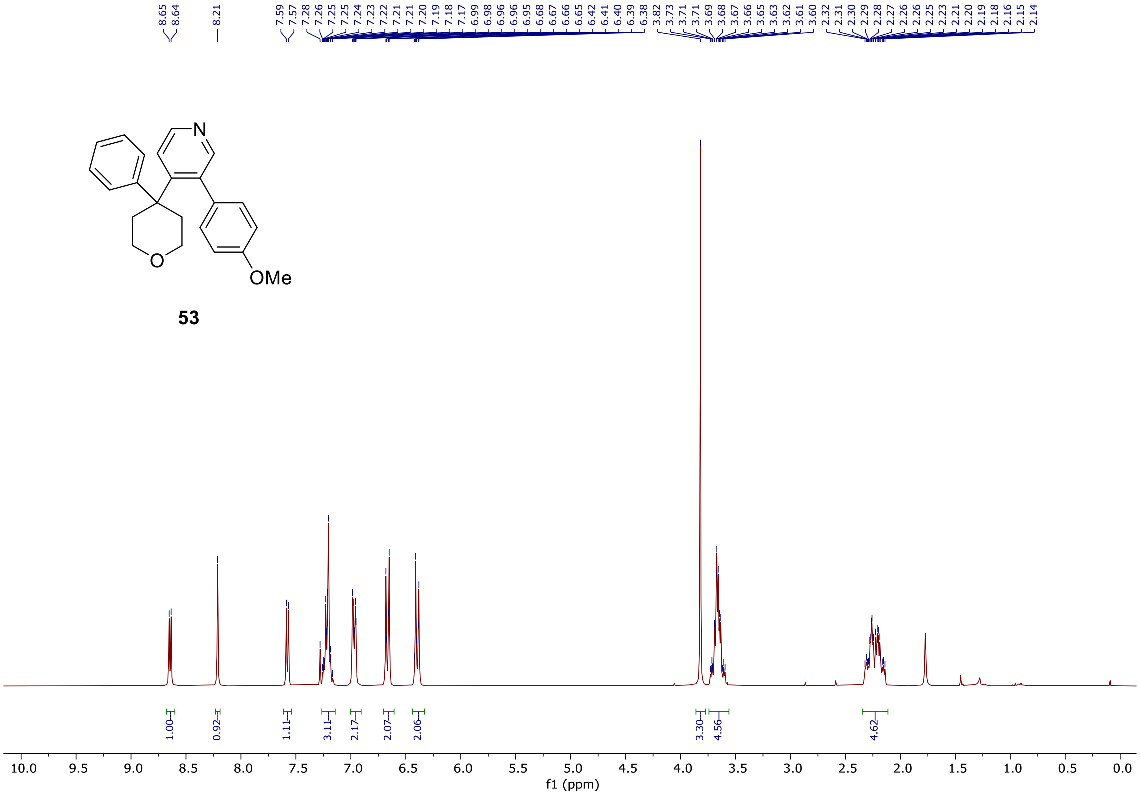

N
O
OMe
53
8.65
8.64
8.21
7.59
7.57
7.28
7.26
7.25
7.25
7.24
7.23
7.22
7.21
7.21
7.20
7.19
7.18
7.17
6.99
6.98
6.96
6.96
6.95
6.68
6.67
6.66
6.65
6.42
6.41
6.40
6.39
6.38
3.82
3.73
3.71
3.71
3.69
3.68
3.67
3.66
3.65
3.63
3.62
3.61
3.60
2.32
2.31
2.30
2.29
2.28
2.27
2.26
2.25
2.23
2.21
2.20
2.19
2.18
2.16
2.15
2.14
1.00
0.92
1.11
3.11
2.17
2.07
2.06
3.30
4.56
4.62
10.0 9.5 9.0 8.5 8.0 7.5 7.0 6.5 6.0 5.5 5.0 4.5 4.0 3.5 3.0 2.5 2.0 1.5 1.0 0.5 0.0
f1 (ppm)

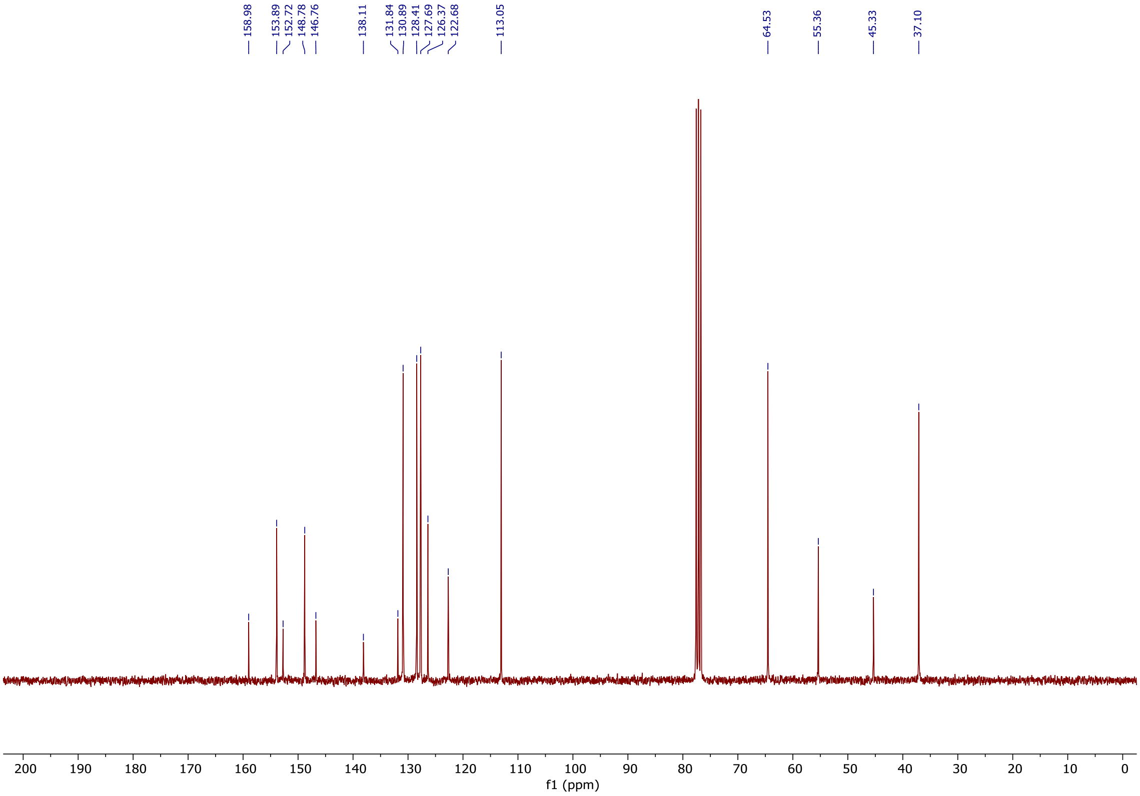

158.98
153.89
152.72
148.78
146.76
138.11
131.84
130.89
128.41
127.69
126.37
122.68
113.05
64.53
55.36
45.33
37.10
200 190 180 170 160 150 140 130 120 110 100 90 80 70 60 50 40 30 20 10 0
f1 (ppm)

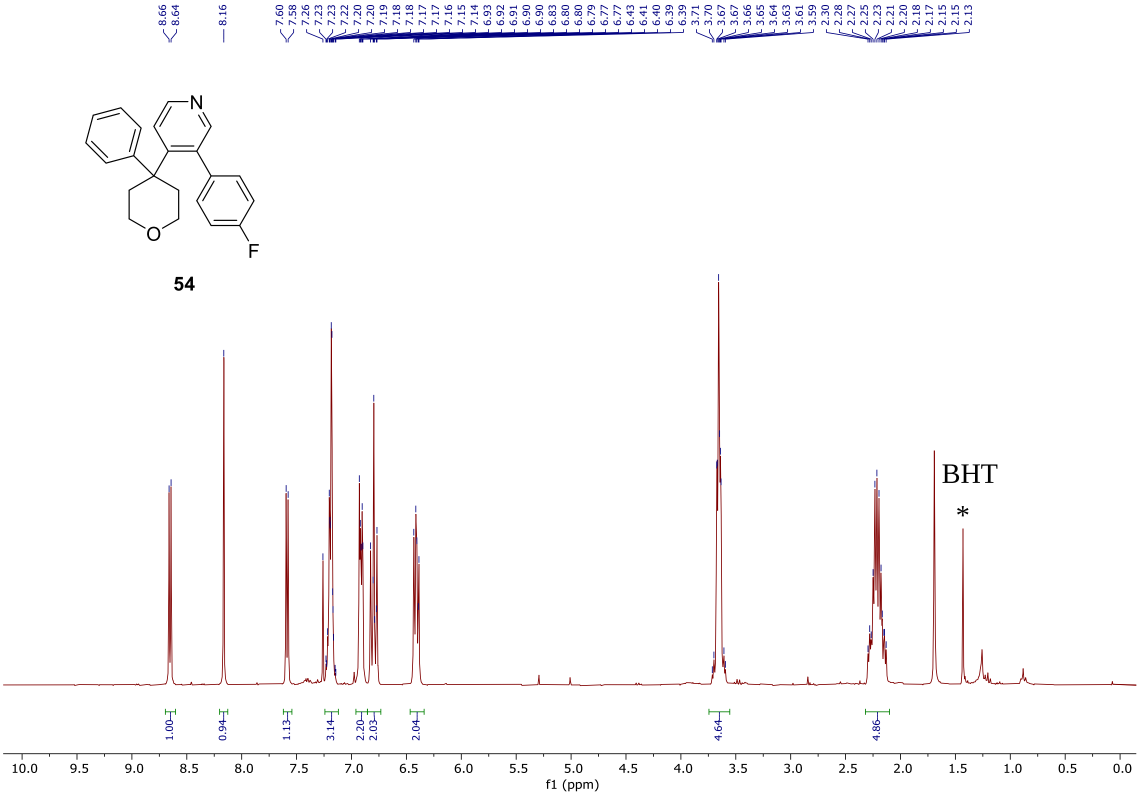
8.66 8.64
8.16
7.60 7.58 7.26 7.23 7.23 7.22 7.20 7.20 7.19 7.18 7.18 7.17 7.17 7.16 7.15 7.14 6.93 6.92 6.91 6.90 6.90 6.83 6.80 6.80 6.79 6.77 6.77 6.43 6.41 6.40 6.39 6.39
3.71 3.70 3.67 3.67 3.66 3.65 3.64 3.63 3.61 3.59
2.30 2.28 2.27 2.25 2.23 2.21 2.20 2.18 2.17 2.15 2.13
N
O
F
BHT
*
1.00 0.94 1.13 3.14 2.20 2.03 2.04 4.64 4.86
10.0 9.5 9.0 8.5 8.0 7.5 7.0 6.5 6.0 5.5 5.0 4.5 4.0 3.5 3.0 2.5 2.0 1.5 1.0 0.5 0.0
f1 (ppm)

54

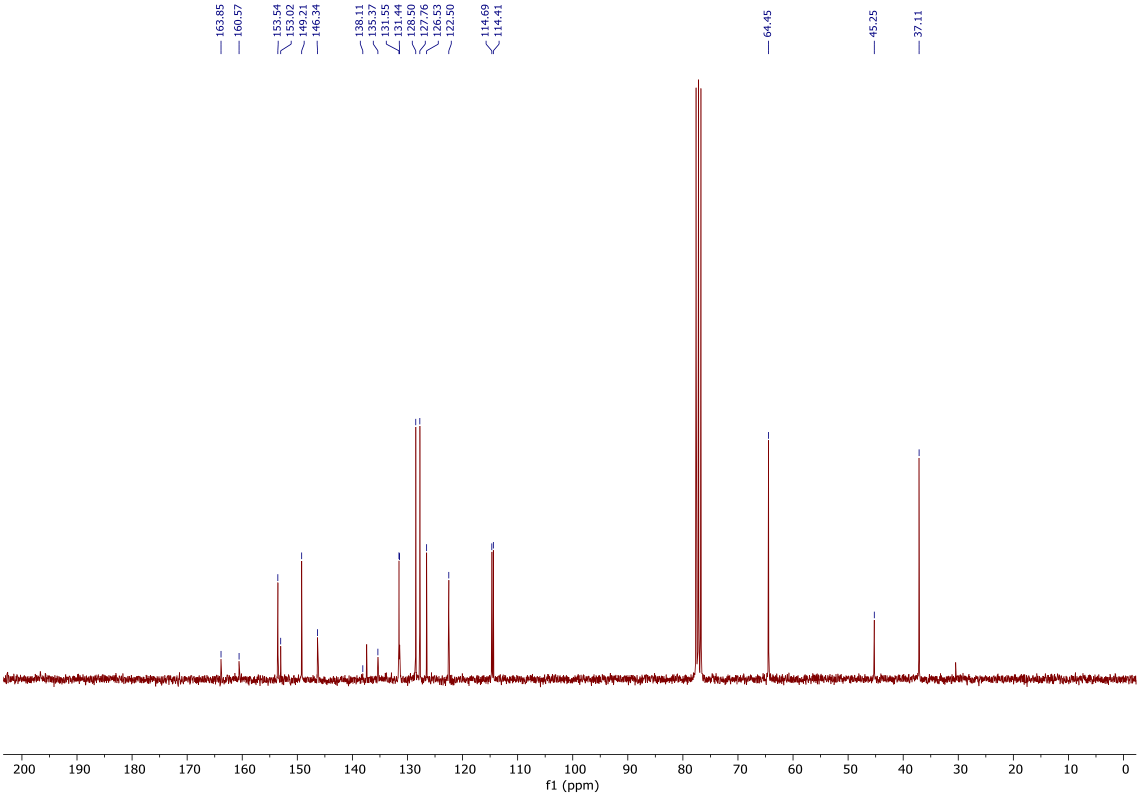
163.85
160.57
153.54 153.02 149.21 146.34
138.11 135.37 131.55 131.44 128.50 127.76 126.53 122.50
114.69 114.41
64.45
45.25
37.11
200 190 180 170 160 150 140 130 120 110 100 90 80 70 60 50 40 30 20 10 0
f1 (ppm)

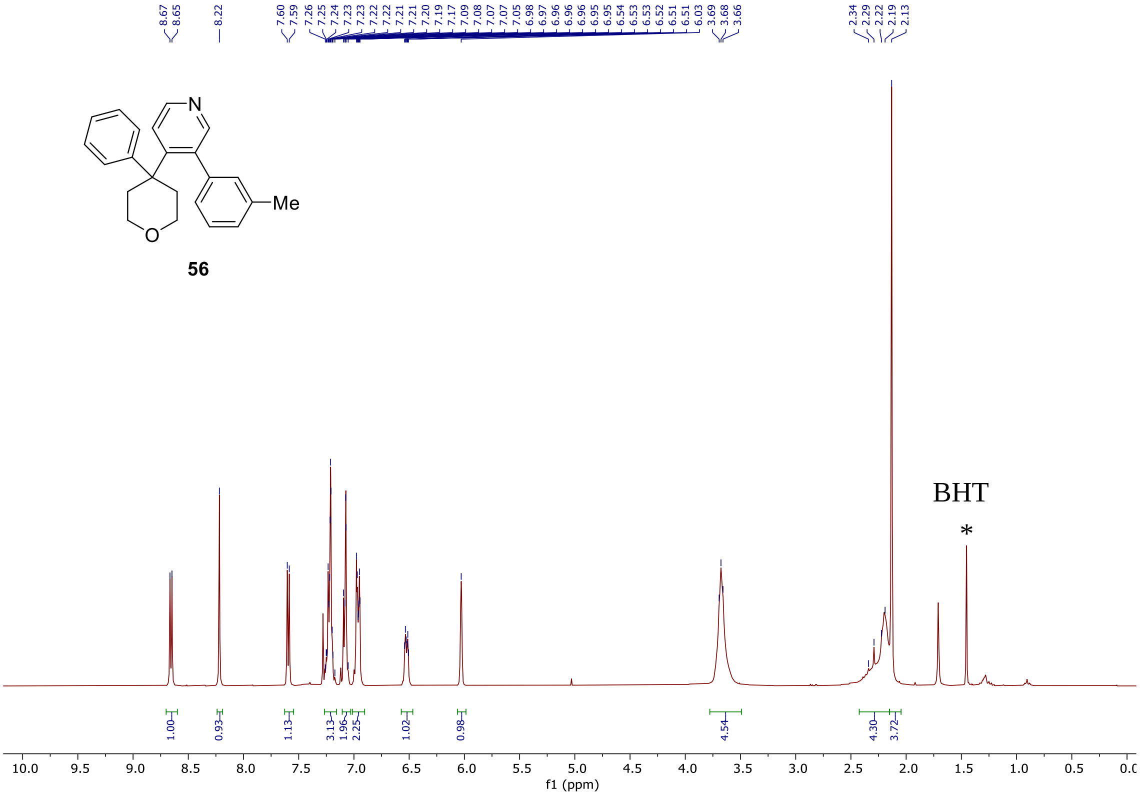
8.67
8.65
8.22
7.60
7.59
7.26
7.25
7.24
7.23
7.23
7.22
7.22
7.21
7.21
7.20
7.19
7.17
7.09
7.08
7.07
7.07
7.05
6.98
6.97
6.96
6.96
6.96
6.95
6.95
6.54
6.53
6.53
6.52
6.51
6.51
6.03
3.69
3.68
3.66
2.34
2.29
2.22
2.19
2.13
N
Me
O
56
BHT
*
1.00
0.93
1.13
3.13
1.96
2.25
1.02
0.98
4.54
4.30
3.72
10.0 9.5 9.0 8.5 8.0 7.5 7.0 6.5 6.0 5.5 5.0 4.5 4.0 3.5 3.0 2.5 2.0 1.5 1.0 0.5 0.0
f1 (ppm)

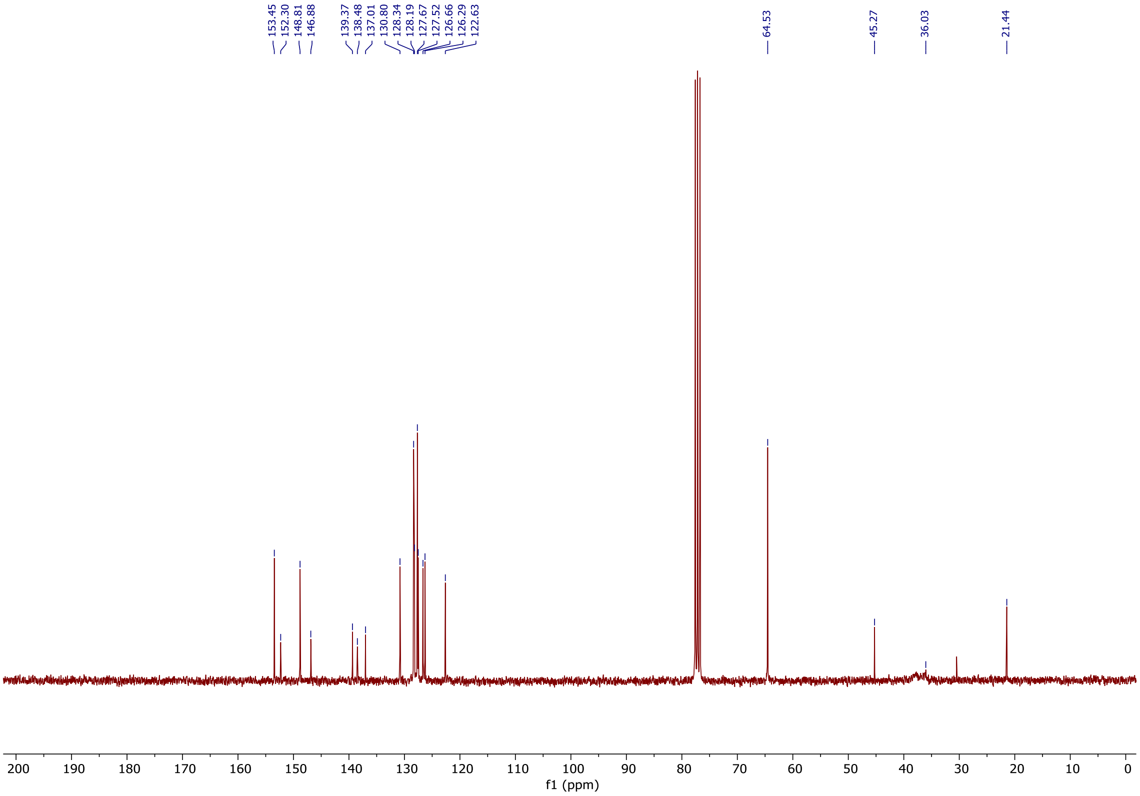
153.45
152.30
148.81
146.88
139.37
138.48
137.01
130.80
128.34
128.19
127.67
127.52
126.66
126.29
122.63
64.53
45.27
36.03
21.44
200 190 180 170 160 150 140 130 120 110 100 90 80 70 60 50 40 30 20 10 0
f1 (ppm)

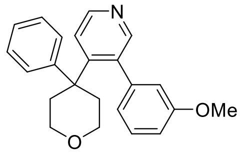

57

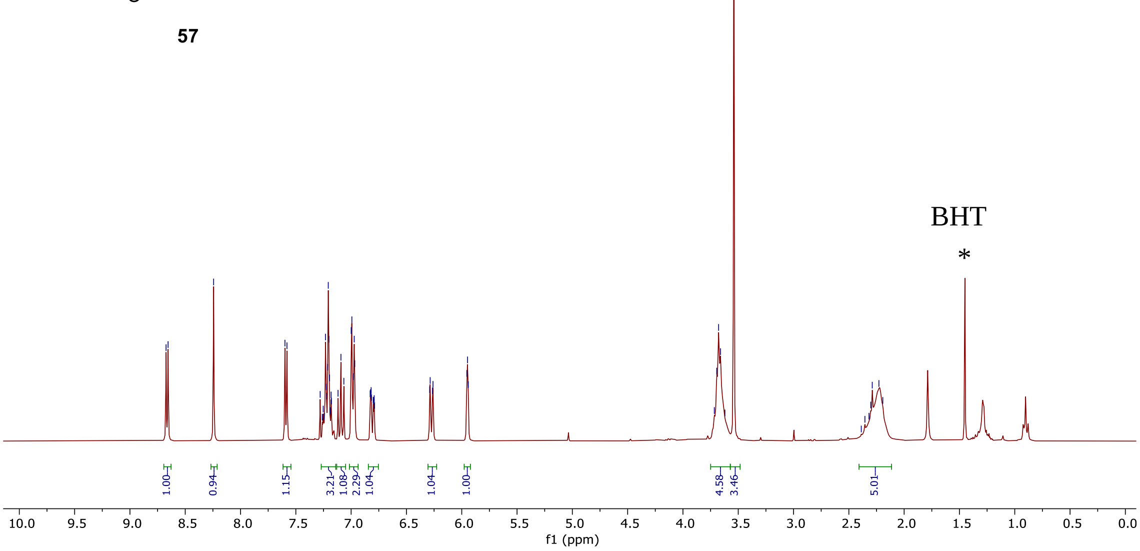
8.67
8.66
8.24
7.60
7.58
7.28
7.26
7.25
7.25
7.23
7.23
7.22
7.21
7.21
7.20
7.20
7.19
7.18
7.18
7.12
7.09
7.07
7.00
6.99
6.98
6.97
6.97
6.83
6.82
6.82
6.82
6.80
6.80
6.79
6.79
6.29
6.29
6.26
6.26
5.95
5.95
5.94
3.72
3.69
3.68
3.66
3.62
3.54
2.39
2.35
2.32
2.30
2.29
2.23
2.19
BHT
*
1.00
0.94
1.15
3.21
1.08
2.29
1.04
1.04
1.00
4.58
3.46
5.01
10.0 9.5 9.0 8.5 8.0 7.5 7.0 6.5 6.0 5.5 5.0 4.5 4.0 3.5 3.0 2.5 2.0 1.5 1.0 0.5 0.0
f1 (ppm)

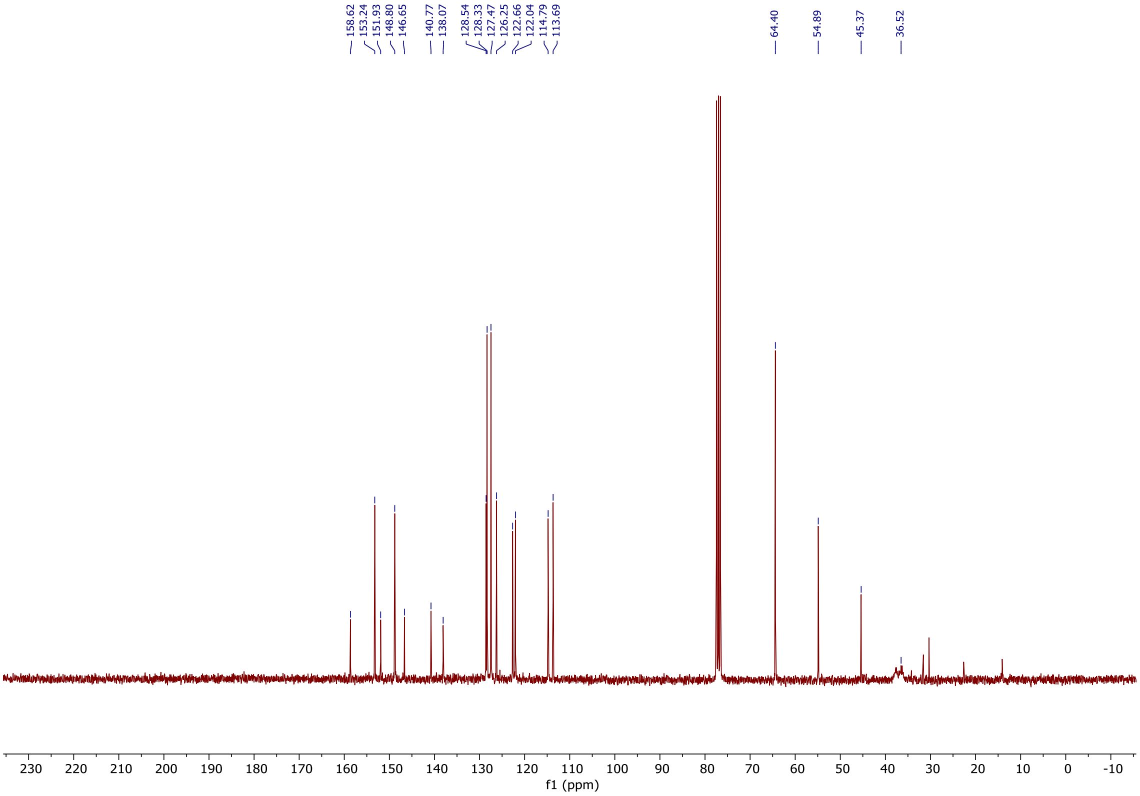
158.62
153.24
151.93
148.80
146.65
140.77
138.07
128.54
128.33
127.47
126.25
122.66
122.04
114.79
113.69
64.40
54.89
45.37
36.52
230 220 210 200 190 180 170 160 150 140 130 120 110 100 90 80 70 60 50 40 30 20 10 0 -10
f1 (ppm)

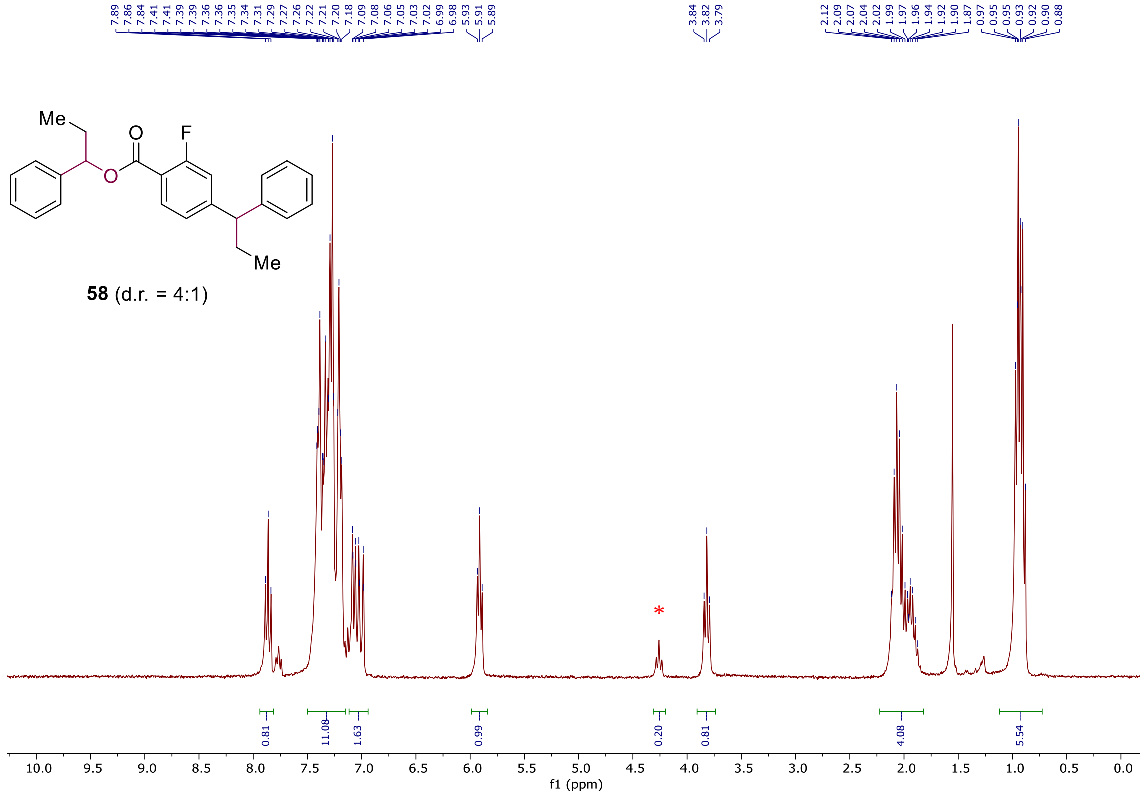

7.89 7.86 7.84 7.41 7.41 7.39 7.39 7.36 7.35 7.34 7.31 7.29 7.27 7.26 7.22 7.21 7.20 7.18 7.09 7.08 7.06 7.05 7.03 7.02 6.99 6.98 5.93 5.91 5.89
3.84 3.82 3.79
2.12 2.09 2.07 2.04 2.02 1.99 1.97 1.96 1.94 1.92 1.90 1.87 0.97 0.95 0.95 0.93 0.92 0.90 0.88
Me
O
F
O
Me
58 (d.r. = 4:1)
*
0.81 11.08 1.63 0.99 0.20 0.81 4.08 5.54
10.0 9.5 9.0 8.5 8.0 7.5 7.0 6.5 6.0 5.5 5.0 4.5 4.0 3.5 3.0 2.5 2.0 1.5 1.0 0.5 0.0
f1 (ppm)

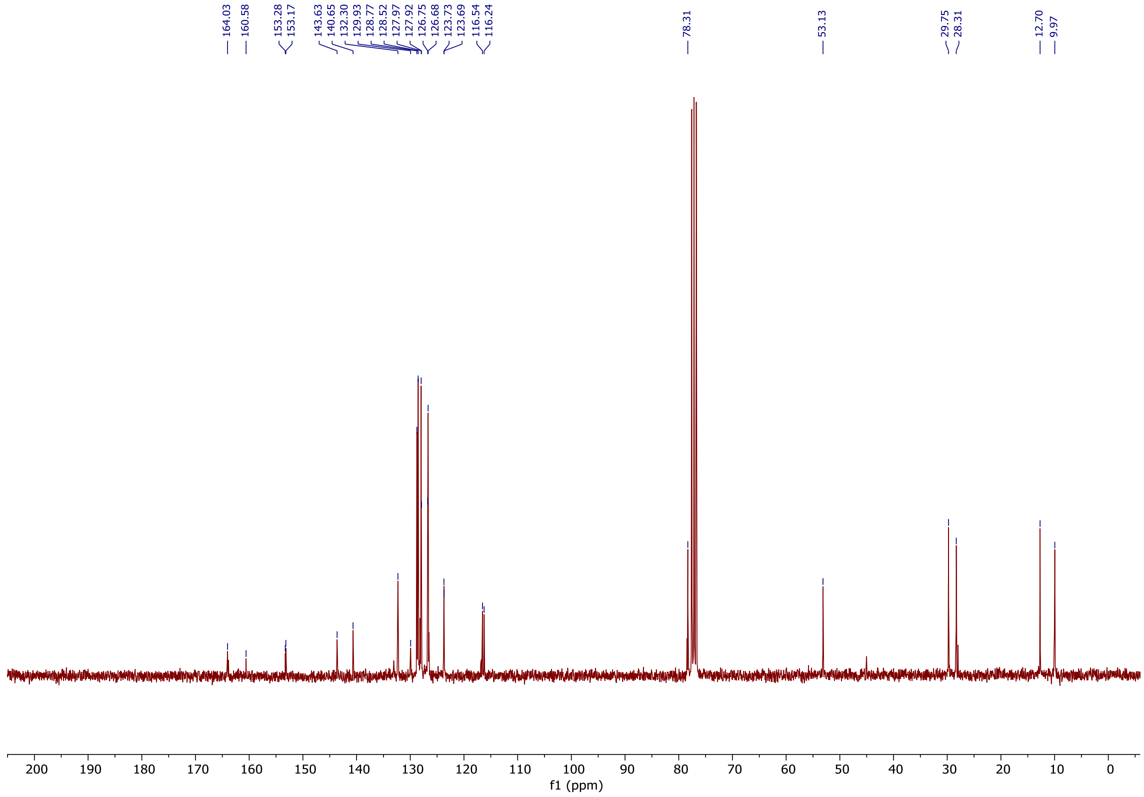

164.03 160.58 153.28 153.17 143.63 140.65 132.30 129.93 128.77 128.52 127.97 127.92 126.75 126.68 123.73 123.69 116.54 116.24
78.31
53.13
29.75 28.31
12.70 9.97
200 190 180 170 160 150 140 130 120 110 100 90 80 70 60 50 40 30 20 10 0
f1 (ppm)
